# New method for the mathematical derivation of the ventilatory anaerobic threshold: a retrospective study

**DOI:** 10.1186/s13102-019-0122-z

**Published:** 2019-06-24

**Authors:** Hirotaka Nishijima, Kazuyuki Kominami, Kazuo Kondo, Masatoshi Akino, Masayuki Sakurai

**Affiliations:** 1 Cardiology, Sapporo Ryokuai Hospital, 6-30 Kitanao 1-1, Kiyota-ku, Sapporo, 004-0861 Japan; 2 Cardiac Rehabilitation, Sapporo Ryokuai Hospital, 6-30 Kitano 1-1, Kiyota-ku, Sapporo, 004-0861 Japan; 3 Cardiac Rehabilitation, Hokko Memorial Hospital, 1-6 Kita-27 Higashi-8, Higashiku, Sapporo, 065-0027 Japan; 4 Cardiology, Hokko Memorial Hospital, 1-6 Kita-27 Higashi-8, Higashiku, Sapporo, 065-0027 Japan

**Keywords:** V-slope, Exponential fitting, Cardiac rehabilitation

## Abstract

**Background:**

Ventilatory anaerobic threshold (VAT) is a useful submaximal measure of exercise tolerance; however, it must be visually determined. We developed a new mathematical method to objectively determine VAT.

**Methods:**

We employed two retrospective population data sets (A/B). Data A (from 128 healthy subjects, patients with cardiovascular risk factors, and cardiac subjects at institution A, who underwent symptom-limited cardiopulmonary exercise testing) were used to develop the method. Data B (from 163 cardiac patients at institution B, who underwent pre−/post-rehabilitation submaximal exercise testing) were used to apply the developed method. VAT (by V-slope) was visually determined (vVAT), assuming that the pre-VAT segment is parallel to the respiratory exchange ratio (R) = 1 line.

**Results:**

First, from data A, exponential fitting of ramp V-slope data yielded the equation *y = ba*^x^, where *a* is the slope of the exponential function: a smaller value signified a less steep curve, representing less VCO_2_ against VO_2_. Next, a tangential line parallel to *R* = 1 was drawn. The x-axis value of the contact point was the derived VAT, termed the expVAT (VCO_2_) (calculated as LN (1/[b*LN(*a*)]/LN(*a*). This point represents an instantaneous ΔVCO_2_/ΔVO_2_ of 1.0. Second, in a similar way, the relation of VO2 vs. VE (minute ventilation) was fitted exponentially. The tangent line that crosses zero was drawn and the x-axis value was termed expVAT (VE) (calculated as 1/LN(*a*). For data A, the correlation coefficients (r) of vVAT versus VAT (CO_2_), and VAT (VE) were 0.924 and 0.903, respectively (*p* < 0.001), with no significant difference between mean values with the limits of agreement (1.96*SD of the pair difference) being ±276 and ± 278 mL/min, respectively. expVAT (VCO_2_) and expVAT (VE) significantly correlated with VO_2_peak (*r* = 0.971, *r* = 0.935, *p* < 0.001). For data B, after cardiac rehabilitation, expVAT (CO_2_) and exp. (VE) (mL/min) increased from 641 ± 185 to 685 ± 201 and from 696 ± 182 to 727 ± 209, respectively (*p* < 0.001, *p* < 0.008), while vVAT increased from 673 ± 191 to 734 ± 226 (*p* < 0.001). During submaximal testing, expVAT (VCO_2_) underestimated VAT, whereas expVAT (VE) did not.

**Conclusions:**

Two new mathematically-derived estimates to determine VAT are promising because they yielded an objective VAT that significantly correlated with VO_2_peak, and detected training effect as well as visual VAT did.

**Electronic supplementary material:**

The online version of this article (10.1186/s13102-019-0122-z) contains supplementary material, which is available to authorized users.

## Background

Ventilatory anaerobic threshold (VAT) and maximal oxygen uptake (VO_2_max) are two commonly used parameters during cardiopulmonary exercise testing (CPX) [1, 2] that provide physiological and metabolic data that are unobtainable with routine clinical exercise testing involving only electrocardiographic and blood pressure measurements and clinical signs and symptoms. VO_2_max is currently the gold standard for measuring exercise tolerance; VAT, also an index of exercise tolerance, has a singular characteristic not shared by VO_2_max. VAT is visually observed online during exercise testing before the symptom-limited maximal point. Furthermore, it closely correlates with VO_2_max, the gold standard measure of exercise tolerance. Additionally, the exercise protocol does not require maximal testing. However, as it requires visual determination, it is ultimately a subjective measurement. In a scientific investigation employing VAT, the detection of VAT must be made in a blinded manner.

Several computerized algorithms to objectively detect VAT have been developed [3]; however, they have not been widely used. Cardiopulmonary exercise systems generally include computer programs to detect VAT, although their validity has never been tested. It has been reported that there is significant variability in VAT determination among varying programs [3].

In this study, we describe a simple, new method to mathematically derive VAT that does not require computer programs and its successful application in assessing the effects of cardiac rehabilitation.

## Methods

### Study design and population

This retrospective study aimed to develop a new method for the objective determination of VAT. It is based on data sets from two institutions: symptom-limited exercise testing data from institution A and pre- and post- cardiac rehabilitation submaximal exercise testing data from institution B. The objective method of determining VAT was developed using data from institution A. The developed method was then applied to the data from institution B. The mean age of the 128 subjects at institution A (population A) was 52 ± 21 years. This population consisted of 67 healthy subjects, 20 patients with cardiovascular risk factors (hypertension, diabetes mellitus, and hyperlipidemia) who were medication, and 41 patients with various cardiovascular diseases (Table 1). The New York Heart Association (NYHA) functional class of the patients was determined as either I or II, although the classification had not been coded. As a rule, those with NYHA class III or higher did not undergo exercise testing at either institution. Although not a clinical one, we employed the Weber-Janicki classification of exercise tolerance for cardiac patients [4]. The mean age of the 163 patients undergoing cardiac rehabilitation at institution B (population B) was 63 ± 10 years (Table 1). CPX was performed upon entry to the cardiac rehabilitation program, and follow-up testing was done after 3–6 months (mean, 4.5 ± 0.9 months). In 52 patients of the total 163, a hospital-based program (requiring outpatient visits) was not feasible and a home exercise program was offered. Exercise was prescribed at the intensity of the visually determined VAT (vVAT), lasting about 30 min, primarily using stationary bikes or treadmills. Bodyweight resistance exercise was also offered. A total of about 1 h of exercise consisted of working bouts and resting periods. The home program initially consisted of walking for 10 min at the level of VAT, eventually extending to 30 min/day.

**Table 1:** Demographic and clinical characteristics (populations A and B)

	Population A healthy (*n* = 67)	Population A with CV risks (*n* = 20)	Population A cardiac (*n* = 41)	Population B cardiac (*n* = 163)
Age, y	37 ± 20*	59 ± 11	67 ± 9	63 ± 10
Sex (male/female)	48/19	20/0	26/15	141/22
Body weight, kg	62 ± 11*	72 ± 7	59 ± 9	66.1 ± 10.9
Body mass index, kg/m^2^	22.1 ± 3.0*	25.8 ± 2.8	23.3 ± 3.1	24.7 ± 3.2
Heart disease etiology: ischemic (%)	NA	NA	76	84
Weber class: A/B/C/D^†^	NA	NA		
Using VO2peak (n)			7/12/20/2	NA
Using VAT (n)			1/16/18/6	11/40/66/29
Medications (n)	NA			
ACE or ARB		9	16	91
Diuretic		2	7	51
Beta-blocker		0	17	72
Inotropics		0	0	4
Ca channel blocker		17	7	35
Anti-lipidemic		3	16	85
Anti-diabetic		2	4	42

*p-Value was significant at least at < 0.05 by 1-way analysis of variance

^†^Weber-Janicki functional classification: Using VO2peak, based on VO2peak; Using VAT, based on the ventilatory anaerobic threshold

*CV* cardiovascular, *ACE* angiotensin-converting enzyme inhibitor, *ARB* angiotensin II receptor blocker, *Ca* calcium

### CPX

CPX was performed using a stationary bicycle and a breath-by-breath gas analyzer (AE-300S; Minato Ikagaku, Tokyo, Japan). The exercise testing protocol was similar at both institutions; work rates varied from 5 to 25 W/min, preceded by a 2-min warm-up stage. As a general rule at institution A, symptom-limited maximal exercise testing was performed; however, for the purpose of this study, only data sets of exercise tests in which it was documented that the subject was unable to continue pedaling at a specified rate were selected. Cardiologists directly supervised tests. No cardiopulmonary exercise data, such as peak heart rate (HR), VO_2_, or respiratory exchange rate (R), were reviewed for the inclusion. At institution B, a submaximal exercise protocol was routinely adopted. Physical therapists who were certified by the Japanese Association of Cardiac Rehabilitation administered exercise tests and in-house cardiologists were always available when needed. Exercise was generally stopped shortly after the test administrator saw online that the VAT had appeared. In the rehabilitation study of institution B, 127 patients underwent pre−/post-exercise testing under the same 10 W/min ramp protocol, with 13 patients under the same ramp protocol (5 or 15 W/min) and 23 patients under different ramp protocols. In this group (institution B), the last highest value of physiological measurement, such as HR or VO_2_, was described as the highest HR or VO_2_; this was not the conventional HRpeak or VO_2peak_ under the symptom-limited exercise testing. For the perceived rate of exertion, the Borg scale (/20) was used. Although both scales of dyspnea and leg fatigue were recorded, the higher of the two scales was used for analysis in this study.

All subjects completed a written informed consent to exercise tests.

### Visual determination of VAT

The VAT was determined according to the V-slope method [1], because we concur with Wasserman et al. [1] that this graphical method depicts the most basic and direct metabolic energy relation during exercise: VO_2_ vs. VCO_2_ (VCO_2_: as a presumed result of the buffered lactic acid produced), from which V-slope VAT is to be detected. This is not affected by factors other than those that produce VCO_2_, such as individual ventilatory sensitivity to CO_2_ and the presence of mechanical limitation to ventilation such as chronic obstructive pulmonary disease [1], which may alter the relation: VO_2_ vs. VE. It seems that most researchers employ a method of breakpoint detection with two lines (pre- and post-segments, S1 and S2), as reported by Beaver et al. [5], to determine VAT. We have found that a variation proposed by Sue et al. [6] offers easier VAT detection. This method proposes that the pre-VAT segment of the V-slope (S1) is parallel to the respiratory exchange ratio (R) = 1 line, although this is primarily based on empirical observations. Once this parallel line is visually set (S1), the post-VAT segment (S2) is simply seen as breaking off from this parallel line. When more than one such parallel lines were observed, the first one was always taken as S1, the end of which is the VAT. The VAT thus determined best agreed with the lactate threshold in the study we previously reported [7]. Ventilatory equivalent and end-tidal gas concentration plots were also utilized, primarily for the detection of hyperventilation, which breaks the V-slope leftward and may be interpreted as a VAT point. The details of our VAT detection method have been reported with actual examples in a previous study [8] and in this study Additional file 1. The initial part of Additional file 1 also includes explanations of our VAT determination technique using sample graphs. The VAT values that several test administrators recorded at the time of testing were not used in this research analysis. All VAT determinations were performed independently by one investigator. VAT determinations before and after rehabilitation were performed in a completely blinded manner; the independent controller assigned a random number to each graph. The reliability of VAT determination (population A: symptom-limited) by the investigator whose reading was adopted in this study was ±129 mL/min according to the limits of agreement (LoA), with two cases of failed detection. This compared well with the report of Myers et al. (±207 mL/min). [9]. In the submaximal series, VAT was not detected in 17 cases before rehabilitation and in 8 cases after rehabilitation.

Our detection of VAT according to the ventilatory equivalent has been unsatisfactory in the past; the non-detection rate had been higher than that using the V-slope. However, in this investigation, we determined the ventilatory equivalent VAT (veqVAT) in population A for the purpose of comparison. The veqVAT was not detected in 10 cases.

### Mathematical estimation of objective VAT

**Fig. 1:**
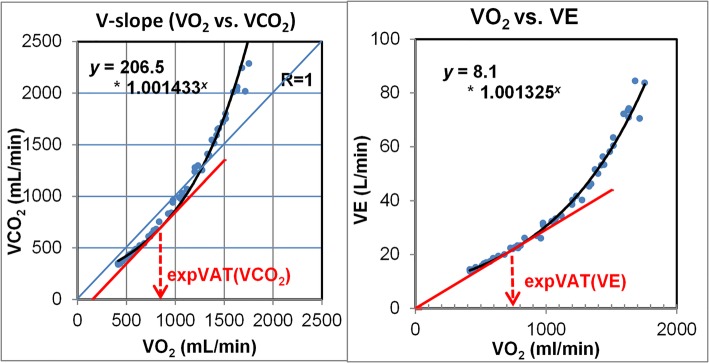
(left) Diagram showing the method of determination of expVAT (VCO_2_), and (right) expVAT (VE). expVAT (VCO_2_), VAT derived from exponential fitting of the V-slope (VCO2 vs. VO2). expVAT (VE), VAT derived from exponential fitting of the relation VO_2_ vs. VE (p) (30 y/o healthy female weighing 57 kg).

First, we performed exponential curve fitting on each V-slope by using ramp exercise data (excluding resting and warm-up data). The function was expressed as *y = ba*^*x*^. Subsequently, the straight tangent line parallel to *R* = 1 was drawn by using differential calculus. This was done based on the framework of our visual method of detecting VAT, as previously described [8]. The x-axis value of the contact point of the exponential curve and the tangent parallel line is the derived VAT (Fig. 1: left), and this was termed expVAT (VCO_2_). The final expVAT value was simply calculated according to the following equation: LN (1/[b*LN(*a*)]) divided by LN(*a*), where LN is the natural logarithm. All 128 graphs demonstrating exponentially fitted curves for raw data points are included in Additional file 2. The derivation of this equation is detailed in Additional file 3: Supplementary Methods 1. The coefficient of the exponential function “*a*” represents the overall steepness of the V-slope, with a smaller value representing a higher VAT. The physiological meaning of the slope equaling 1.0 is interesting because at that point on the fitted exponential V-slope, the instantaneous ΔVCO_2_/ΔVO_2_ is 1.0.

In view of the increasing use of oxygen uptake efficiency slope (OUES), which is calculated as a slope of VO_2_/log-transformed minute ventilation (VE) [10], we also derived VAT based on the conventional VO_2_ vs. VE relation. This relation was initially used by Wasserman et al. [1] for detecting VAT, and subsequently replaced by the VO_2_ vs. VCO_2_ relation (V-slope method). A variation of this method, the ventilatory equivalent method, is still used as an alternative/supplement to the V-slope method; a rise of VE/VO_2_ from the horizontal line is taken as a VAT. The horizontal line as a baseline for VE/VO_2_ mathematically assumes that on the VO_2_ vs. VE graph, a line that crosses the origin is the reference line. Accordingly, after we performed an exponential fitting of the VO_2_ (x-axis) vs. VE (y-axis) relation, we drew the tangential line to the exponential curve that crosses the origin (0, 0) of the graph (Fig. 1: right). The x-value of the contact point was termed the expVAT (VE), corresponds to the point of the minimum (nadir) value of VE/VO2, which some investigators use as VAT [11]. The final equation for calculation is simply 1/LN(*a*), the inverse of LN(*a*); a small value representing a less steep slope yields a larger VAT.

### OUES of VE and VCO_2_

We also calculated the OUES, as our method has a similarity to this method. OUES uses a logarithmic transformation [10]. Our method uses an exponential function, which is the inverse function of the logarithm. OUES is based on the linear relation between log-transformed VE (x-axis) and non-transformed VO_2_ (y-axis), in contrast to the more conventional relation of VO_2_ (x-axis) vs. VE (y-axis). As the V-slope method of VAT is fitted as an exponential relation of VO_2_ (x-axis) vs. VCO2 (y-axis) in this study, we plotted the relation of log-transformed VCO_2_ (x-axis) vs. VO_2_ (y-axis) for comparison. Taking the logarithm of VCO_2_ and applying a linear equation will provide a similar index to the original OUES. To prevent confusion, we called this parameter OUES (VCO_2_) and the original OUES as OUES (VE).

Occasionally, the fitting of original data by exponential function was visually poor, with poor linear correlation coefficient (r) between the original and fitted values owing to insufficient number of data or artifacts such as hyperventilation. We excluded these by applying the outlier detection method of Smirnov-Grubbs on the value of r; 4 plots for population A and 7 plots for population B were excluded for further analysis. The details are given in Additional file 3: Supplementary Methods 2.

### Sensitivity analysis

The first sensitivity analysis aimed to assess whether not exercising to a symptomatic maximum influences the calculated value of derived VAT and OUES; the first 75% of data points of the exercise duration were used and compared with the full data values (Population A). The second sensitivity analysis was performed to assess how far beyond vVAT the exercise had to be extended before the methods of expVAT (VCO_2_) and expVAT (VE) became effective for detecting pre- and post-rehabilitation changes (Population B). Two analyses were performed: 1) when the range of data was limited only up to the point of vVAT, and 2) when it was extended beyond to the point of vVAT + 100 mL/min VO_2_ (approximately the limits of agreement for intra-individual VAT determinations). Additionally, as it was mathematically evident that exponential fitting could be performed and expVATs could be calculated even when the V-slope data were limited to the range equal to vVAT or lower (pre-VAT slope), a linear regression analysis was performed for this data segment to detect the data range in which it was unlikely to detect valid expVATs (Population A) .

Additionally, comparisons of vVAT, expVAT (VCO_2_), and expVAT (VE) in each subject group in population A (healthy, those with CV risks, cardiac subjects) were performed to assess the consistency of these values across groups.

### Statistical analysis

Results are given as mean ± standard deviation. The means of the two groups were compared using the Student’s t-test. Paired data were compared using the paired t-test. Correlations between variables were assessed using Pearson correlation coefficients (r). The comparison of the three groups was performed using a one-way analysis of variance (ANOVA) followed by post-hoc test (Bonferroni). Correlated groups were assessed using repeat ANOVA. When assessing the agreement between the two methods, we employed the limits of agreement (LoA) [12], as in cases of repeat determinations of vVAT, and the agreement of vVAT vs. expVAT. When describing LoA in the text, 1.96*SD of the difference of relevant pairs of data was used. Two correlated correlation coefficients (r) were compared using Williams’s procedure [13]. Outlier detection was performed using the Smirnov-Grubbs method (at *p* = 0.001). When the exponentially fitted slopes of the V-slope and the VO_2_ vs. VE slope were compared, both VCO_2_ and VE values were converted to the percentage of each peak value and expressed as a slope (%peak) because the units of VCO_2_ and VE differed. Residuals were also calculated from the %peak values and expressed as the root mean square deviation (RMSD, analogous to the standard deviation of residuals).

The percentage of age-adjusted maximal HR was calculated as peak HR/(220-age) × 100. Relative VAT (%) was calculated as (VAT/VO_2_peak) × 100.

Statistical analyses including curve fitting were performed with Statistics for Excel 2015 (Social Survey Research Information Co., Tokyo, Japan).

The research protocol was approved by the institutional review boards of Sapporo Ryokuai Hospital and Hokko Memorial Hospital. The corresponding author had full access to all the data in the study and takes responsibility for its integrity and the data analysis.

## Results

The demographic and clinical characteristics of the two populations (A and B) are summarized in Table 1.

**Fig. 2:**
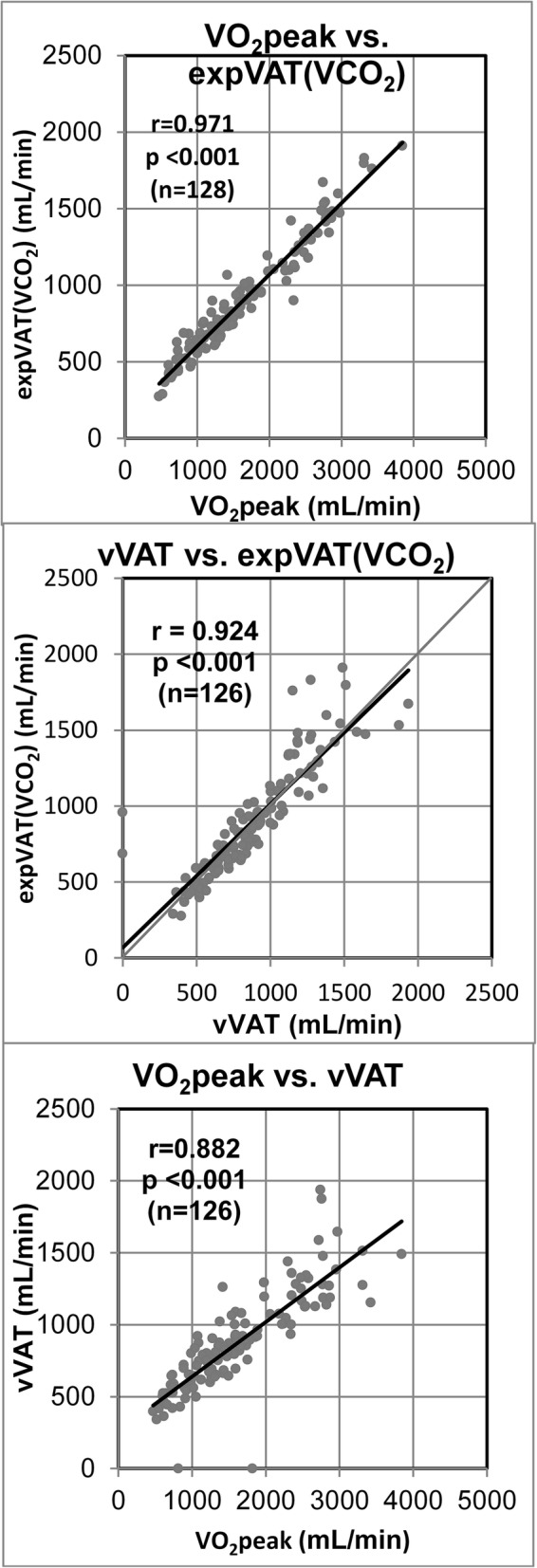
Relation between (top) VO_2_peak vs. expVAT (VCO_2_), (middle) vVAT vs. expVAT (VCO_2_), and (bottom) VO_2_peak vs. vVAT. Abbreviations are the same as in Fig. 1.

**Fig. 3:**
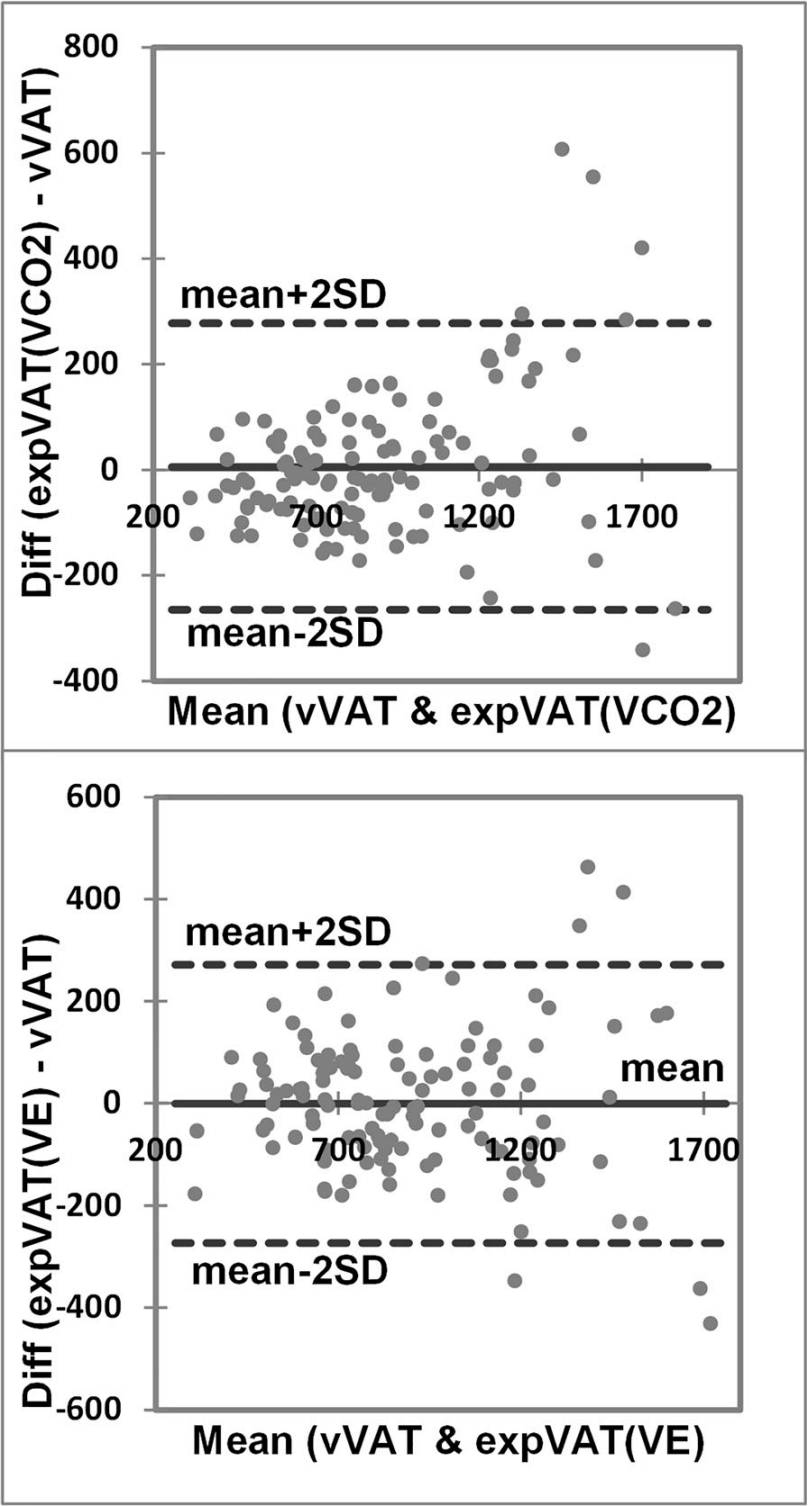
Bland and Altman plot between vVAT and expVAT. (top) expVAT (VCO2) and (bottom) expVAT (VE).

The basic results of CPX and derived values including expVAT (VCO_2_), expVAT (VE), OUES (VE), and OUES (VCO_2_) are summarized in Table 2 (maximal) and Table 3 (submaximal, pre/post cardiac rehabilitation). In the maximal exercise study (institution A), expVAT (VCO_2_) significantly correlated with both VO_2_peak (*r* = 0.971, *p* < 0.001) and vVAT (*r* = 0.924, *p* < 0.001) (Fig. 2: top, middle). The correlation (r) between VO_2_peak and vVAT was 0.882 (*p* < 0.001) (Fig. 2: bottom). The expVAT (VE) also significantly correlated with both VO_2_peak (*r* = 0.932, *p* < 0.001) and vVAT (*r* = 0.903, *p* < 0.001). Interestingly, the correlation coefficient between expVAT (VCO_2_) and VO_2_peak was significantly better than that of vVAT vs. expVAT (VCO_2_) (*p* < 0.01). The correlation between expVAT (VE) and VO_2_peak was also significantly better than that of expVAT (VE) vs. vVAT (*p* = 0.041). The LoA between expVAT (VCO2) vs. vVAT, and that of expVAT (VE) vs, vVAT were ± 276 and ± 278 mL/min (Fig. 3), respectively, which were considerably higher than the intra-individual reliability of ±129 mL/min of our institution (see Methods: visual determination of VAT). Additionally, the variation in agreement was larger at higher VATs.

**Table 2:** Cardiopulmonary exercise and derived variables of population A (maximal exercise)

	Healthy subjects	Patients with CV risk factors	Cardiac subjects
Peak work rate, W	170 ± 65*	121 ± 36	71 ± 28
Ramp duration, s	550 ± 139	572 ± 132	494 ± 148
Peak Borg scale	16.7 ± 1.3*	16.8 ± 1.4	15.4 ± 1.6
Peak HR, beats/min	164 ± 23*	134 ± 27	117 ± 19
%Predicted peak HR	90 ± 9.5*	83 ± 13	77 ± 12
VO_2_peak, mL/min	2031 ± 728*	1581 ± 413	973 ± 323
VO_2_peak, mL/kg/min	32.2 ± 9.2*	22.0 ± 6.0	16.0 ± 4.0
Peak R	1.18 ± 0.09	1.15 ± 0.09	1.14 ± 0.10
vVAT, mL/min	1042 ± 310*	916 ± 237	629 ± 169
vVAT, mL/min/kg	16.7 ± 4.1*	12.6 ± 3.2	10.4 ± 1.9
expVAT (VCO_2_), mL/min	1085 ± 353*	874 ± 199	589 ± 168
expVAT (VCO_2_), mL/min/kg	17.2 ± 4.2*	12.1 ± 2.7	9.7 ± 2.0
Exponential coefficient	1.0012 ± 0.0003*	1.0013 ± 0.0002	1.0020 ± 0.0006
expVAT (VE), mL/min	1053 ± 295*	884 ± 203	634 ± 176
expVAT (VE), mL/min/kg	16.7 ± 3.4*	12.2 ± 2.8	10.4 ± 2.3
Exponential coefficient	1.0010 ± 0.0003*	1.0011 ± 0.0002	1.0017 ± 0.0006
OUES (VCO_2_), mL/min	2036 ± 599*	1725 ± 345	1197 ± 326
OUES (VE), mL/min	2386 ± 679*	1988 ± 451	1413 ± 404

*p-Value was significant at least at < 0.05 by 1-way analysis of variance.

*HR* heart rate, *VO*_*2*_ oxygen uptake, *R* respiratory exchange rate, *vVAT* visual ventilatory anaerobic threshold: expVAT (VCO_2_), exponential VAT based on VO_2_ vs. VCO_2_; expVAT (VE), exponential VAT based on VO_2_ vs. VE; OUES (VCO_2_), oxygen uptake efficiency slope based on logVCO_2_ vs. VO_2_; OUES (VE), oxygen uptake efficiency slope based on logVE vs. VO_2_

**Table 3:** Correlation matrix of exercise tolerance variables (population A)

	VO_2_peak	vVAT	expVAT (VCO_2_)	expVAT (VE)	OUES (VCO_2_)	OUES (VE)
VO_2_peak		0.882	0.971	0.932	0.968	0.935
vVAT	0.882		0.924	0.903	0.914	0.897
expVAT (VCO_2_)	0.971	0.924		0.970	0.987	0.970
expVAT (VE)	0.932	0.903	0.970		0.970	0.998
OUES (VCO_2_)	0.968	0.914	0.987	0.970		0.971
OUES (VE)	0.935	0.897	0.970	0.998	0.971	

All r values are significant at *p* < 0.001. Abbreviations are the same as in Table 2

The correlations among VO_2_peak, vVAT, expVAT (VCO_2_), expVAT (VE), OUES (VCO_2_), and OUES (VE) were all excellent, with r values ranging from 0.882 to 0.998. The correlation matrix among these variables is shown in Table 3. Derived values such as expVAT and OUES tended to have better correlation with vO2peak than vVAT. There were no significant differences in the mean values among vVAT, expVAT (CO_2_), and expVAT (VE) (*p* = 0.712, by repeat ANOVA).

The mean expVAT (VCO_2_) and VAT (VE) represented 57.3 ± 8.2% and 57.6 ± 11.4% of VO_2_peak, respectively, whereas vVAT represented 58.5 ± 12.7% of VO_2_peak (%relative VAT). Both %relative expVAT (VCO_2_) and %relative vVAT significantly decreased as VO_2_peak increased, although with a higher VO_2_peak, %relative VAT tended to plateau at about 50% (Additional file 3). As the calculation formula shows, the coefficient of the fitted exponential equation “*a*” (slope) was the primary factor in determining the value of the derived VAT; a smaller value represented a slower exponential slope, which signified a higher VAT. The coefficient *a* was inversely correlated with expVAT (VCO_2_) and expVAT (VE) (*r* = 0.842 and *r* = 0.842, *p* < 0.001 and *p* < 0.001, respectively).

The mean veqVAT (1049 ± 414 mL/min) was significantly higher than either vVAT, expVAT (VCO_2_), or expVAT (VE) (*p* < 0.001).

Comparison of the two age−/sex-matched groups (21 healthy vs. 21 cardiac subjects) showed that the mean VO_2_peak, vVAT, expVAT (VCO_2_), expVAT (VE), OUES (VCO_2_), and OUES (VE) were all significantly different between the groups (Additional file 3: Table S1).

The results of the submaximal exercise protocol for pre- and post-cardiac rehabilitation (institution B) are summarized in Table 4.

**Table 4:** Cardiopulmonary exercise and derived variables of population B (submaximal exercise: before/after cardiac rehabilitation)

	Before	After	*p*-Value
Body weight, kg	66.1 ± 10.9	66.7 ± 10.9	0.008
Highest work rate, W^a^	66 ± 20	82 ± 24	< 0.001
Ramp exercise duration, s	360 ± 107	438 ± 121	< 0.001
Highest Borg scale^a^, /20	14.1 ± 1.7	14.7 ± 1.9	< 0.001
Highest HR^a^, beats/min	109 ± 18	116 ± 18	< 0.001
Highest VO_2_^a^, mL/min	971 ± 285	1146 ± 341	< 0.001
Highest VO_2_^a^, mL/min/kg	14.6 ± 3.4	17.0 ± 4.0	< 0.001
Highest R^a^	1.06 ± 0.10	1.09 ± 0.11	< 0.001
vVAT, mL/min	673 ± 191	734 ± 226	< 0.001
vVAT, mL/min/kg	10.1 ± 2.4	10.8 ± 2.8	< 0.001
expVAT (VCO_2_), mL/min	641 ± 185	685 ± 201	< 0.001
expVAT (VCO_2_), mL/min/kg	9.6 ± 2.3	10.2 ± 2.4	< 0.001
Exponential coefficient, “*a*”	1.0018 ± 0.0004	1.0017 ± 0.0004	0.001
expVAT (VE), mL/min	696 ± 182	727 ± 209	0.008
expVAT (VE), mL/min/kg	10.6 ± 2.7	10.9 ± 2.7	0.074
Exponential coefficient, “*a*”	1.0015 ± 0.0004	1.0014 ± 0.0004	0.011
OUES (VCO_2_), mL/min	1306 ± 327	1385 ± 355	< 0.001
OUES (VE), mL/min	1513 ± 398	1610 ± 467	< 0.001

^a^As the exercise protocol was submaximal, the last value was simply designated as the highest value (see the “CPX” subsection in Methods). Abbreviations are the same as in Table 2

The vVAT, expVAT (CO_2_), expVAT (VE), OUES (CO_2_), and OUES (VE) each improved significantly after cardiac rehabilitation, although the mean detected pre/post difference was largest with vVAT, followed by expVAT (VCO_2_) and then expVAT (VE). The mean expVAT (VCO_2_) was significantly less than the mean vVAT at both pre- and post-rehabilitation by about 7.2 ± 14.7% (*p* < 0.001) and 6.8 ± 11.5% (*p* < 0.001), respectively. The mean expVAT (VE) did not differ significantly from the mean vVAT at both pre- and post-rehabilitation, although the sensitivity to detect changes was less than that of expVAT (VCO2). As seen in Table 3, the coefficients “*a*” (slope) was not as sensitive as the calculated values of VAT to detect changes (*p*-values not as good). Additionally, the correlations among vVAT, expVAT (CO_2_), expVAT (VE), OUES (VE), and OUES (VCO_2_) were excellent both at pre- and post-rehabilitation, with r values ranging from 0.799 to 0.976 and from 0.873 to 0.994, respectively (Additional file 3: Table S2).

### Sensitivity analysis

An assessment of 75% of the data (population A) showed that expVAT (VCO_2_) significantly decreased by 7.4 ± 7.1% compared with that of the full data (*p* = 0.001, from 893 ± 359 to 816 ± 301 mL/min), whereas expVAT (VE) did not show a significant change (*p* = 0.125, from 895 ± 310 to 885 ± 292 mL/min). An assessment of 75% of OUES (VCO_2_) data significantly decreased by 8.2 ± 5.9% compared with the full data value (*p* < 0.001, from 1719 ± 615 to 1562 ± 520 mL/min), whereas the 75% of OUES (VE) data decreased by only 2.1 ± 7.9% (from 2019 ± 716 to 1962 ± 670 mL/min, *p* < 0.01).

When the analyzed range of data was limited to that of vVAT and lower (population B), significant changes were not detected from pre- to post-expVAT (VCO2) or expVAT (VE). When the data range was extended beyond to vVAT + 100 mL/min VO_2_, expVAT (VCO2) registered a significant change (*p* = 0.013), with VAT (VE) showing a tendency (*p* = 0.070) from pre- to post-rehabilitation. Additional file 3: Table S3 shows the details**)**.

The linear regression of the pre-VAT slope yielded a mean slope of 0.901 ± 0.102 (mean ± SD). The 95% upper range is 1.105 (0.901 + 0.204). Therefore, below this level of linear slope, it may be considered that VAT has not been reached with the particular exercise test and expVAT should not be calculated.

Additionally, comparisons of the three modes of VATs (vVAT, expVAT (VCO_2_), expVAT (VE)) were performed in each subject group in population A (healthy, those with CV risks, and cardiac subjects). In healthy subjects, a significant difference was observed between the mean vVAT and expVAT (VCO_2_) (*p* = 0.038). In cardiac subjects, a significant difference was observed between the mean vVAT and expVAT (VCO_2_) (*p* = 0.007) and between expVAT (VCO_2_) and expVAT (VE) (*p* = 0.004). The absolute mean differences were relatively small, at about 40 mL/min in each case. The main reasons for these discrepancies were primarily the differences in the configuration of the V-slope and the slope for VO_2_ vs. VE in each group, affecting the degree of exponential fitting. These details are presented in the Additional file 3.

## Discussion

We developed a new objective method of estimating VAT: expVAT (VCO2) and expVAT (VE). expVAT (of VCO2 and VE) significantly correlated with VO_2_peak and vVAT. Furthermore, the developed method detected changes in VAT after cardiac rehabilitation, as well as vVAT did. There have been many attempts to objectively determine VAT using mathematical or computer algorithms [14–24]. Seven studies used breakpoint detection with two or more linear functions, three used polynomial functions [16, 17], one used a computerized cumulative sum method to detect a change in the data trend [23], and only one used an exponential model [15]. Many of these methods as well as others have been integrated into computer programs and tested by Ekkekakis et al. [3]. The computed VAT values varied widely from each other, with the correlation coefficients among them ranging from 0.363 to 0.967. Unfortunately, the authors did not compare the computed values with either vVAT or VO_2_max (or VO_2_peak).

As far as we could ascertain, only one study used an exponential model [15]. That study used VE/VO_2_ (ventilatory equivalent of O_2_), rather than VE itself, on the y-axis and VO_2_ on the x-axis. The study subjects included 45 active males. The exponential formula was as follows: y = *e*^bx + a^/x. Differentiating it yielded the minimum value of y (:VE/VO_2_), whereas x (:VAT) = 1/b. Theoretically, the values from that study should be identical to our values. The differences between the previous study and our study are as follows: our method has been applied to the most basic VAT relation: both V-slope (VO_2_ vs. VCO_2_) and the relation VO_2_ vs. VE, without transformation of data. We applied the method in a much larger population (*n* = 128), including cardiac patients who were young or old and male or female individuals. We also applied the method, with success, to detect changes in exercise tolerance before and after intervention (before and after cardiac rehabilitation).

None of these computer programs seem to have been widely used or available for use to date. One of the main reasons might be the unavailability of those computer programs to clinicians or researchers in general. Most CPX systems seem to include a computer program for detecting VAT; however, the exact algorithm has not been made public. Furthermore, no large-scale study comparing mathematically computed VAT’s with that using the visual method that has been published.

As an objective means to estimate exercise tolerance, the OUES has been increasingly used [10]. The close correlation with VO_2_peak has been noted. OUES has also been successfully used to detect exercise tolerance changes after cardiac rehabilitation [25]. At present, OUES generates a new value (VO_2_/log-transformed VE) not resembling the commonly-used values such as VO_2_peak or VAT; however, in the future, this value may become commonly adopted. Our method is unique because it generates an estimated value of VAT from the exponentially fitted V-slope.

Our method preserves the original relation of VO_2_ vs. VCO_2_ (V-slope) or VE, as proposed by Wasserman et al. [1]. We curve-fitted the slope by using the exponential function because the exponential model of lactate increase during exercise has been proposed [26] and the increase in buffered acid has been associated with both VCO_2_ and VE increase. The resulting exponential function yields *y* = *b*^*ax*^, where “*a*” is the slope of the V-slope, analogous to the slope of OUES. This described a characteristic of each V-slope and represented a parameter of exercise tolerance; a smaller “*a*” representing a less steep slope signified a larger value of VAT; less CO_2_ was produced with increasing VO_2_. We calculated VAT from the exponentially fitted V-slope based on our visual method of detecting VAT [8]. As we utilized a parallel line to the *R* = 1 line to detect VAT on a V-slope as proposed by Sue et al. [6], we hypothesized in a similar fashion that the parallel tangent line to the exponentially fitted V-slope might yield a close estimate of VAT, if not exactly a deflection point.

The expVAT showed a close correlation with OUES. The two methods detected changes after cardiac rehabilitation equally well; they are mathematically related. The VO_2_ vs. VE relation is the inverse function of the VE vs. VO_2_ relation. The VO_2_ vs. VE relation is fitted well with the exponential function, and the relation VE vs. VO_2_ is fitted with the logarithmic function, which is the inverse function of the exponential function.

As our derivation shows, this is not a method attempting to detect a deflection point of two lines or slopes (S1 and S2). Rather, it represents a certain midway point toward VO_2_peak. The result shows it is more closely related to VO_2_peak than to vVAT. The expVAT (VCO2) point is where the V-slope (exponentially fitted) equals the instantaneous ΔVCO_2_/ΔVO_2_ of 1.0 (“instantaneous R” as contrasted with the conventional R, which is an average R to that point). Therefore, it has an important physiological basis in the framework of exercise energy metabolism: it is not merely a convenient technical point to estimate VAT.

The expVAT (VE) represents the point where the ratio of VE to VO2 (ventilatory equivalent of O2) changes from a decreasing to an increasing pattern. The mean expVAT (VE) agreed with the mean expVAT (VCO_2_). This was somewhat against our expectation as the expVAT (VCO2) required the hypothesis of parallel shifting of S1 to *R* = 1 (rightward shift of V-slope) [8], whereas expVAT (VE) did not. We cannot explain why these two methods nearly agreed. Although VE changes may physiologically follow VCO_2_ changes, the sensitivity to CO_2_ may vary individually and also according to the presence of ventilatory limitations, such as in chronic obstructive pulmonary disease [5, 6]. Furthermore, particularly during the lighter phase of incremental exercise, VE is significantly influenced by a reduction in the dead space/tidal volume ratio, as VE does not equal alveolar ventilation. All these factors may differentially influence expVAT (VE).

The LoA of about 270 mL/min is probably too large for expVAT to replace vVAT. In addition, a variation in agreement was larger in subjects with a higher VO_2_peak, which appeared to be a stepwise linear pattern of VCO_2_ increase in these subjects (see Additional file 3, section 8 for details). However, the correlation coefficient (r) between expVAT and vVAT was excellent and the group distinction between normal and cardiac subjects could be made. expVAT detected changes in exercise tolerance after cardiac rehabilitation as well as vVAT. Therefore, it seems that expVAT may be used as an objective index of exercise tolerance when group means are compared, or when post intervention changes are evaluated.

vVAT on the V-slope is an important variable to watch during CPX because it provides visualization of a metabolic breakpoint online during an exercise test, albeit a subjective one. For personnel administering an exercise test, it is easy to see online whether the exercising subject has passed a metabolic breakpoint (exactly at what point may not be practically important). It is much easier to see that the subject has passed a breakpoint than to pinpoint the exact breakpoint.

### Study limitations

As our study does not include athletes as subjects, we do not know whether our method could estimate the VAT or training effects for this population. Our data also do not include those of cardiac patients with NYHA III; therefore, we do not know whether our method can be applied to patients with severe functional disability. Retrospective data of cardiac rehabilitation were utilized to examine whether changes in expVAT would parallel changes in vVAT, which were determined in a blinded fashion. The study was not purported to assess the unbiased effects of cardiac rehabilitation that we practice at our hospital; the study was not a randomized controlled trial. The effects were assessed with only submaximal testing and VAT. Maximal exercise studies are needed to confirm whether expVAT also parallels changes in VO_2_peak during cardiac rehabilitation.

The expVAT (VCO_2_) and OUES (VCO_2_) calculated using 75% of the data exhibited a mean 7–8% decrease compared with those calculated using the full data. However, in the submaximal exercise study before and after rehabilitation, expVAT (VCO_2_) performed as well as vVAT did. Therefore, in comparing pre−/post-intervention changes, the lack of a symptomatic maximum may not be too important, as far as exercise protocols are approximately similar in the sense that the exercise passed beyond a visually identifiable VAT point, but not necessarily reaching symptomatic maximum. This issue requires further studies. On the other hand, VE-derived measures, such as expVAT (VE) and OUES (VE), showed only a small or insignificant decrease when 75% of the data were used. In addition, expVAT (VCO_2_) indicates an exponential fitting problem, particularly in subjects with a higher exercise tolerance. This may signify that these VE-derived measures are superior to those derived from VCO_2_. However, as stated earlier, the VO_2_ vs. VCO_2_ relation and the VO_2_ vs. VE relation do not represent physiologically identical relations; it is entirely possible that the overall agreement between expVAT (VCO_2_) and expVAT (VE) is somewhat coincidental. This aspect may need to be studied in broader range of populations.

Finally, even if we constructed a perfect computer program that exactly locates a VAT point on the basis of a threshold model, it would still be a subjective one as long as there is variation in the individually determined threshold (vVAT). Only the consistency of individual VAT determinations would improve, whereas a systemic difference would remain. Therefore, it would not be an unbiased universal value. Indexes such as expVAT and OUES are largely determined in the context of the whole curve of VCO_2_ or VE vs. VO_2_ and the slope characteristics of the whole curve appear to determine exercise tolerance. In that sense expVAT and OUES will not be influenced by individual differences in the threshold visually. Despite not being free from personal bias, vVAT is a useful clinical tool because we believe people usually agree on whether a certain point on the curve (wherever it exactly is) is past a threshold. In addition, it is indispensable as an on-line metabolic monitor during exercise testing because vVAT may be determined quite independently of the whole curve.

## Conclusions

In summary, we have developed a new objective mathematical method to estimate VAT that does not require a computer software program. The estimated VAT showed a high correlation with vVAT and VO_2_peak during maximal exercise. It also detected changes after cardiac rehabilitation as well as vVAT did.

## Additional files

Additional file 1: Determination of vVAT in all 128 cases. (PPTX 2642 kb)

Additional file 2: Exponential fitting of all 128 cases. (PPTX 2984 kb)

Additional file 3: **Supplementary Methods-1**. Derivation of computation formula of expVAT. **Supplementary Methods-2.** Detection of outliers on exponential fitting. **Supplementary figure.** Description of data: Relation of VO2peak vs. %relative expVAT. **Table S1.** Age−/sex-matched comparisons of CPX variables. **Table S2.** Correlation matrix of CPX variables in Population B. **Table S3.** Sensitivity analysis. **Supplementary material:** Comparison of three modes of VAT in each subject group (Population A). (DOCX 1058 kb)

## Data Availability

The datasets generated and analyzed during the current study are available in the Figshare repository, DOI: 10.6084/m9.figshare.7607414

## Abbreviations

ACE: Angiotensin-converting enzyme
ANOVA: Analysis of variance
ARB: Angiotensin II receptor blocker
BMI: Body mass index
CPX: Cardiopulmonary exercise testing
expVAT (VCO_2_): Calculated VAT based on exponential fitting of V-slope (VO_2_ vs. VCO_2_)
expVAT (VE): Calculated VAT based on exponential fitting of VO_2_ vs. VE relation
HR: Heart rate
IQR: Interquartile range
NYHA: New York Heart Association classification
OUES: Oxygen uptake efficiency slope
R: Respiratory exchange rate
RQ: Respiratory quotient
RR: Respiration rate
RtShift: Rightward shift of V-slope
S1: Pre-VAT slope of the V-slope or the S1 itself
S2: Post-VAT slope of the V-slope or the S2 itself
SBP: Systolic blood pressure
VAT: Ventilatory anaerobic threshold expressed in ml/min VO_2_
VCO_2_: Minute carbon dioxide production
VE: Minute ventilation
VO_2_: Minute oxygen uptake
VO_2_peak: Peak oxygen uptake
V-slope: Graphical representation of VO_2_ (mL/min, x-axix) vs. VCO_2_ (mL/min, y-axis)
vVAT: Visually determined ventilatory anaerobic threshold
W: Watt

## References

[1] Wasserman K, Hansen JE, Sue DY, Stringer WW, Sietsema KE, Sun XG (2012). Principles of Exercise Testing and Interpretation: Including Pathophysiology and Clinical Application.

[2] Matsumura N, Nishijima H, Kojima S, Hashimoto F, Minami M, Yasuda H (1983). Determination of anaerobic threshold for assessment of functional state in patients with chronic heart failure. Circulation..

[3] Ekkekakis P, Lind E, Hall EE, Petruzzello SJ (2008). Do regression-based computer algorithms for determining the ventilatory threshold agree?. J Sports Sci..

[4] Janicki JS, Weber KT, McElroy PA (1988). Use of the cardiopulmonary exercise test to evaluate the patient with chronic heart failure. Eur Heart J..

[5] Beaver WL, Wasserman K, Whipp BJ (1986). A new method for detecting anaerobic threshold by gas exchange. J Appl Physiol..

[6] Sue DY, Wasserman K, Moricca RB, Casaburi R (1988). Metabolic acidosis during exercise in patients with chronic obstructive pulmonary disease. Use of the V-slope method for anaerobic threshold determination. Chest..

[7] Kominami K, Nishijima H, Imahashi K, Katsuragawa T, Murakami M, Yonezawa K (2015). Very early lactate threshold in healthy young men as related to oxygen uptake kinetics. Medicine (Baltimore)..

[8] Nishijima H, Kondo K, Yonezawa K, Hashimoto H, Sakurai M (2017). Quantification and physiological significance of the rightward shift of the V-slope during incremental cardiopulmonary exercise testing. BMC Sports Sci Med Rehabil..

[9] Myers J, Goldsmith RL, Keteyian SJ, Brawner CA, Brazil DA, Aldred H (2010). The ventilatory anaerobic threshold in heart failure: a multicenter evaluation of reliability. J Card Fail..

[10] Baba R, Nagashima M, Goto M (1996). Oxygen uptake efficiency slope: a new index of cardiorespiratory functional reserve derived from the relation between oxygen uptake and minute ventilation during incremental exercise. J Am Coll Cardiol..

[11] Mezzani A, Agostoni P, Cohen-Solal A, Corrà U, Jegier A, Kouidi E (2009). Standards for the use of cardiopulmonary exercise testing for the functional evaluation of cardiac patients: a report from the Exercise Physiology Section of the European Association for Cardiovascular Prevention and Rehabilitation. Eur J Cardiovasc Prev Rehabil..

[12] Bland JM, Altman DG (1986). Statistical methods for assessing agreement between two methods of clinical measurement. Lancet..

[13] Howell DC (2007). Statistical Methods for Psychology.

[14] Fukuba Y, Munaka M, Usui S, Sasahara H (1988). Comparison of objective methods for determining ventilatory threshold. Jpn J Physiol..

[15] Hagan RD, Smith MG (1984). Pulmonary ventilation in relation to oxygen uptake and carbon dioxide production during incremental load work. Int J Sports Med..

[16] Santos EL, Giannella-Neto A (2004). Comparison of computerized methods for detecting the ventilatory thresholds. Eur J Appl Physiol..

[17] Cheng B, Kuipers H, Snyder AC, Keizer HA, Jeukendrup A, Hesselink M (1992). A new approach for the determination of ventilatory and lactate thresholds. Int J Sports Med..

[18] Schneider DA, Phillips SE, Stoffolano S (1993). The simplified V-slope method of detecting the gas exchange threshold. Med Sci Sports Exerc..

[19] Zamunér AR, Catai AM, Martins LE, Sakabe D, Da Silva E (2013). Identification and agreement of first turn point by mathematical analysis applied to heart rate, carbon dioxide output and electromyography. Braz J Phys Ther..

[20] Orr GW, Green HJ, Hughson RL, Bennett GW (1982). A computer linear regression model to determine ventilatory anaerobic threshold. J Appl Physiol Respir Environ Exerc Physiol..

[21] Novais LD, Silva E, Simões RP, Sakabe DI, Martins LE, Oliveira L (2016). Anaerobic Threshold by Mathematical Model in Healthy and Post-Myocardial Infarction Men. Int J Sports Med..

[22] Dickstein K, Barvik S, Aarsland T, Snapinn S, Millerhagen J (1990). Validation of a computerized technique for detection of the gas exchange anaerobic threshold in cardiac disease. Am J Cardiol..

[23] Smith DA, O’Donnell TV (1984). The time course during 36 weeks’ endurance training of changes in Vo2 max. and anaerobic t + hreshold as determined with a new computerized method. Clin Sci (Lond)..

[24] Higa MN, Silva E, Neves VF, Catai AM, Gallo L, Silva de Sá MF (2007). Comparison of anaerobic threshold determined by visual and mathematical methods in healthy women. Braz J Med Biol Res..

[25] Van Laethem C, Van De Veire N, De Backer G (2007). Response of the oxygen uptake efficiency slope to exercise training in patients with chronic heart failure. Eur J Heart Fail..

[26] Anderson GS, Rhodes EC (1989). A review of blood lactate and ventilatory methods of detecting transition thresholds. Sports Med..

