# Supplementary material for: New method for the mathematical derivation of the ventilatory anaerobic threshold: a retrospective study
Source: BMC Sports Sci Med Rehabil. 2019 Jun 24;11:10. doi: 10.1186/s13102-019-0122-z (PMC6592010; doi:10.1186/s13102-019-0122-z)

## Slide 1
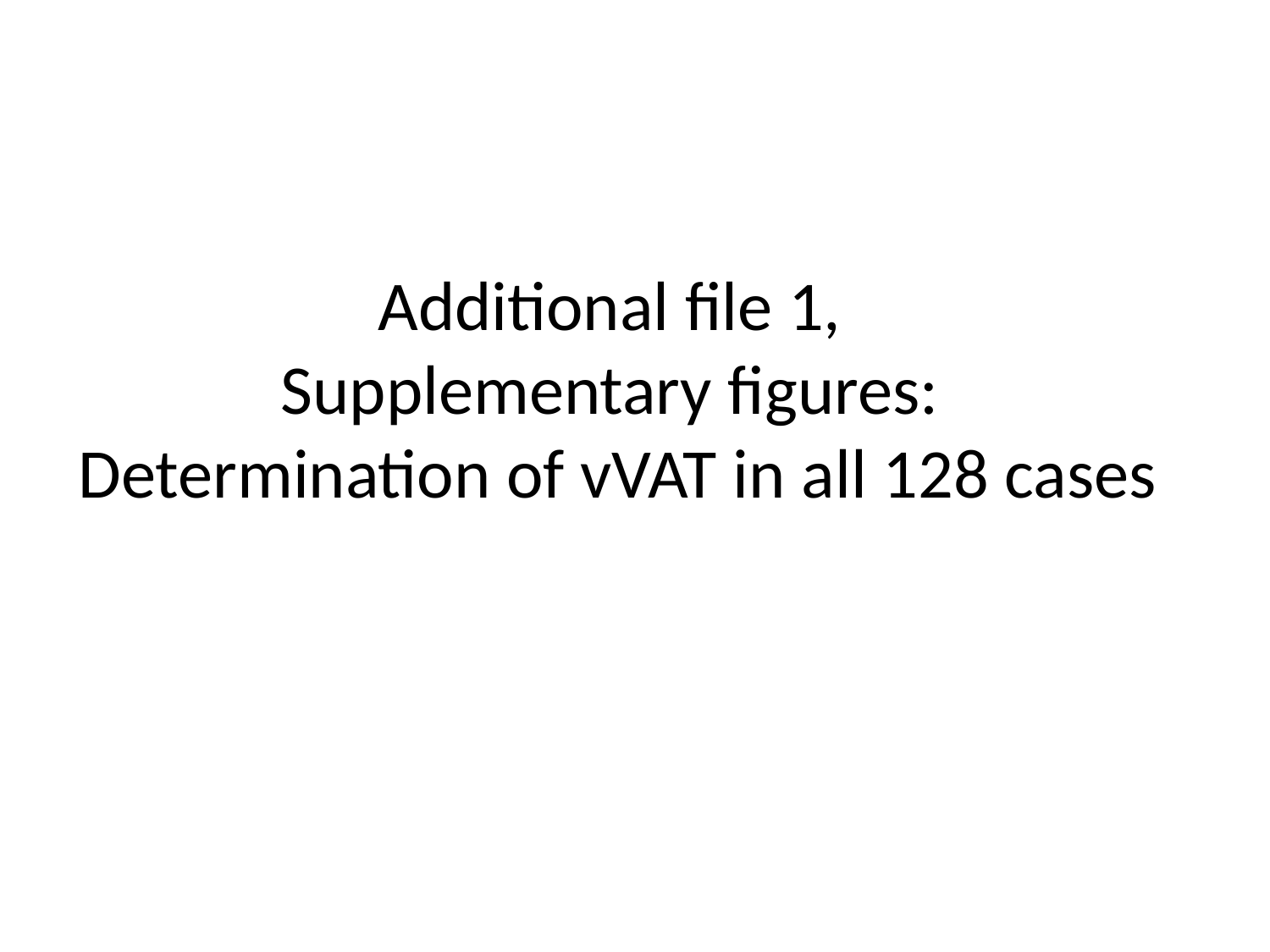

# Additional file 1, Supplementary figures: Determination of vVAT in all 128 cases

## Slide 2
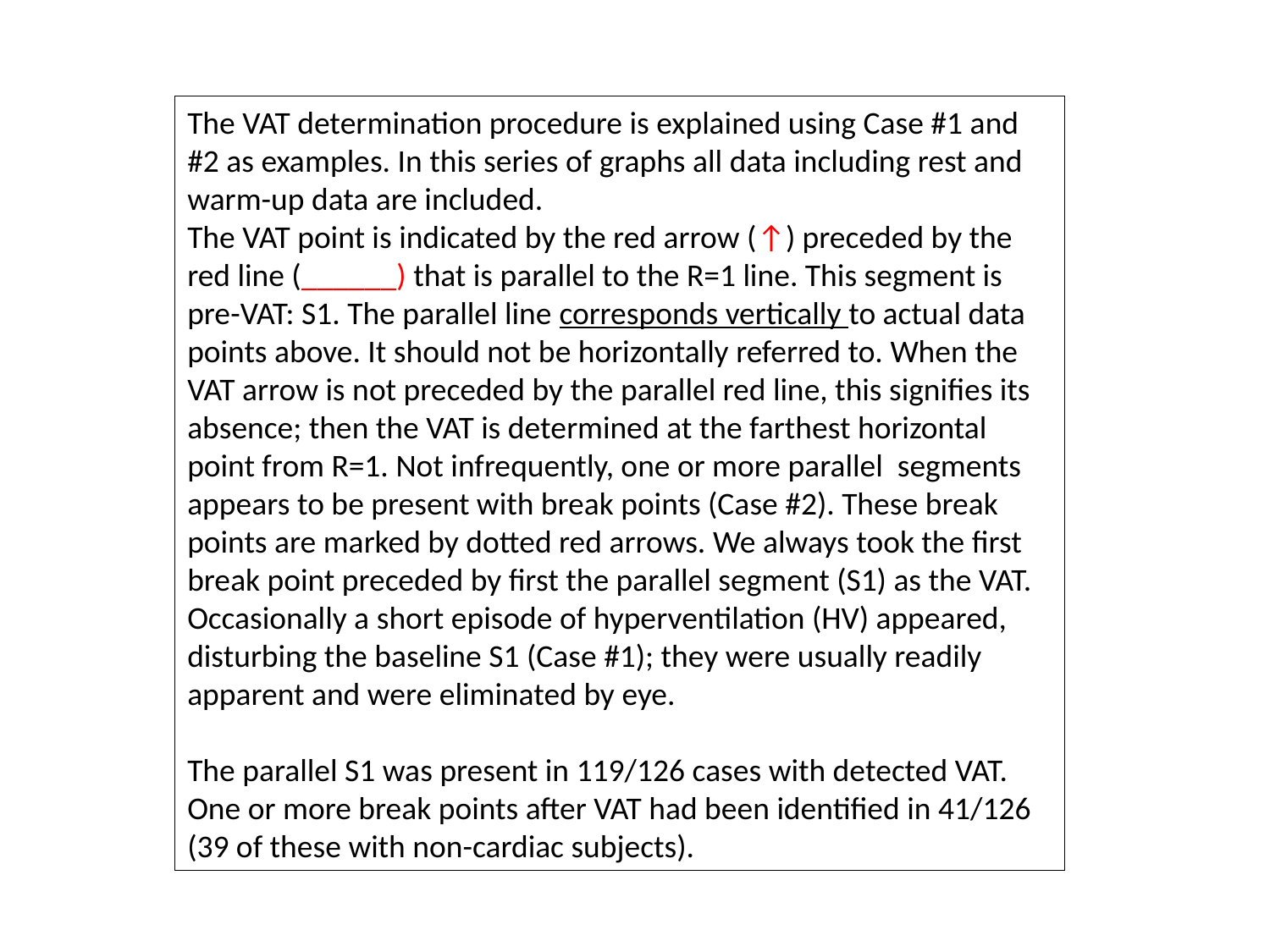

The VAT determination procedure is explained using Case #1 and #2 as examples. In this series of graphs all data including rest and warm-up data are included.
The VAT point is indicated by the red arrow (↑) preceded by the red line (______) that is parallel to the R=1 line. This segment is pre-VAT: S1. The parallel line corresponds vertically to actual data points above. It should not be horizontally referred to. When the VAT arrow is not preceded by the parallel red line, this signifies its absence; then the VAT is determined at the farthest horizontal point from R=1. Not infrequently, one or more parallel segments appears to be present with break points (Case #2). These break points are marked by dotted red arrows. We always took the first break point preceded by first the parallel segment (S1) as the VAT. Occasionally a short episode of hyperventilation (HV) appeared, disturbing the baseline S1 (Case #1); they were usually readily apparent and were eliminated by eye.
The parallel S1 was present in 119/126 cases with detected VAT. One or more break points after VAT had been identified in 41/126 (39 of these with non-cardiac subjects).

## Slide 3
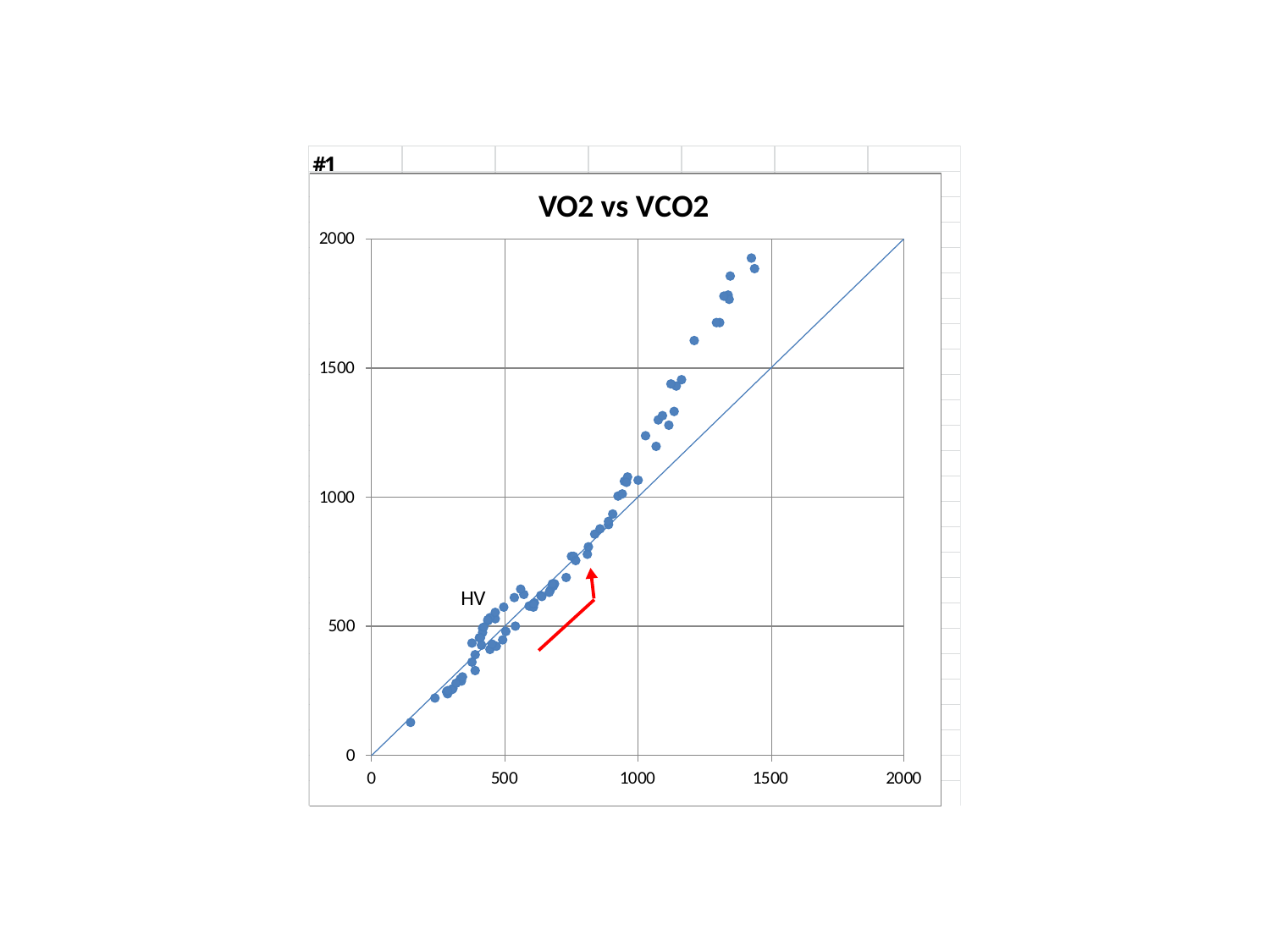

## Slide 4
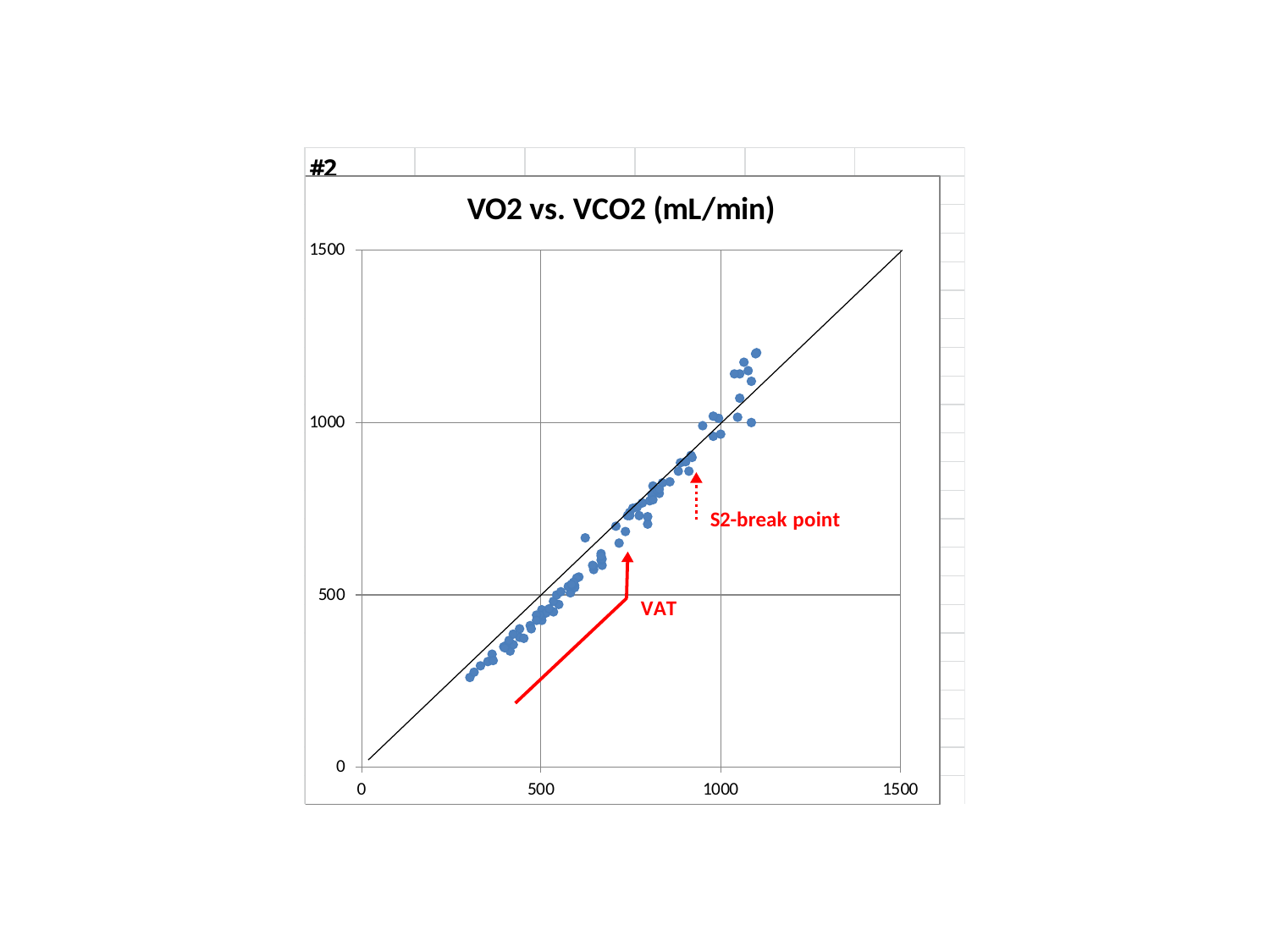

## Slide 5
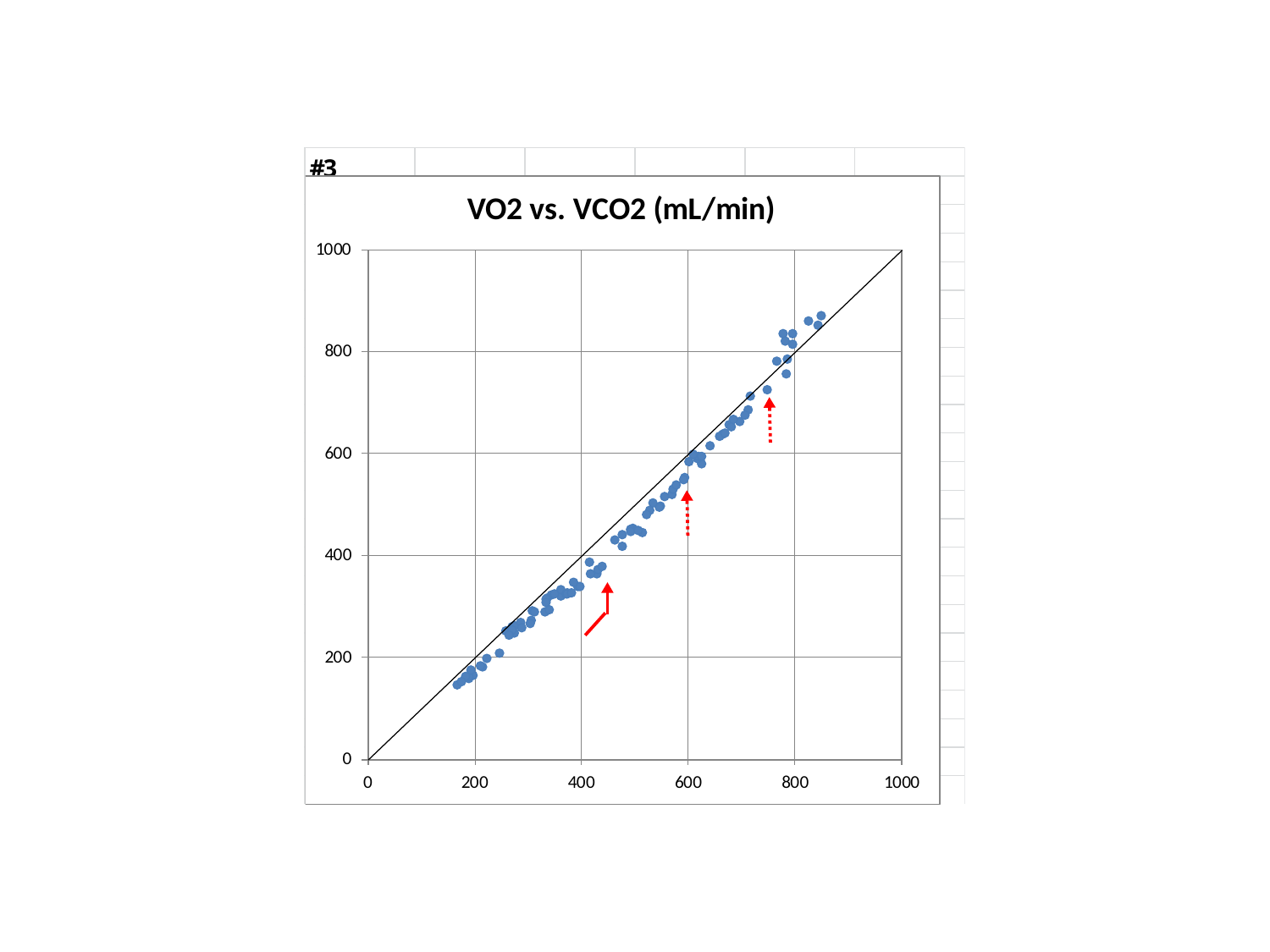

## Slide 6
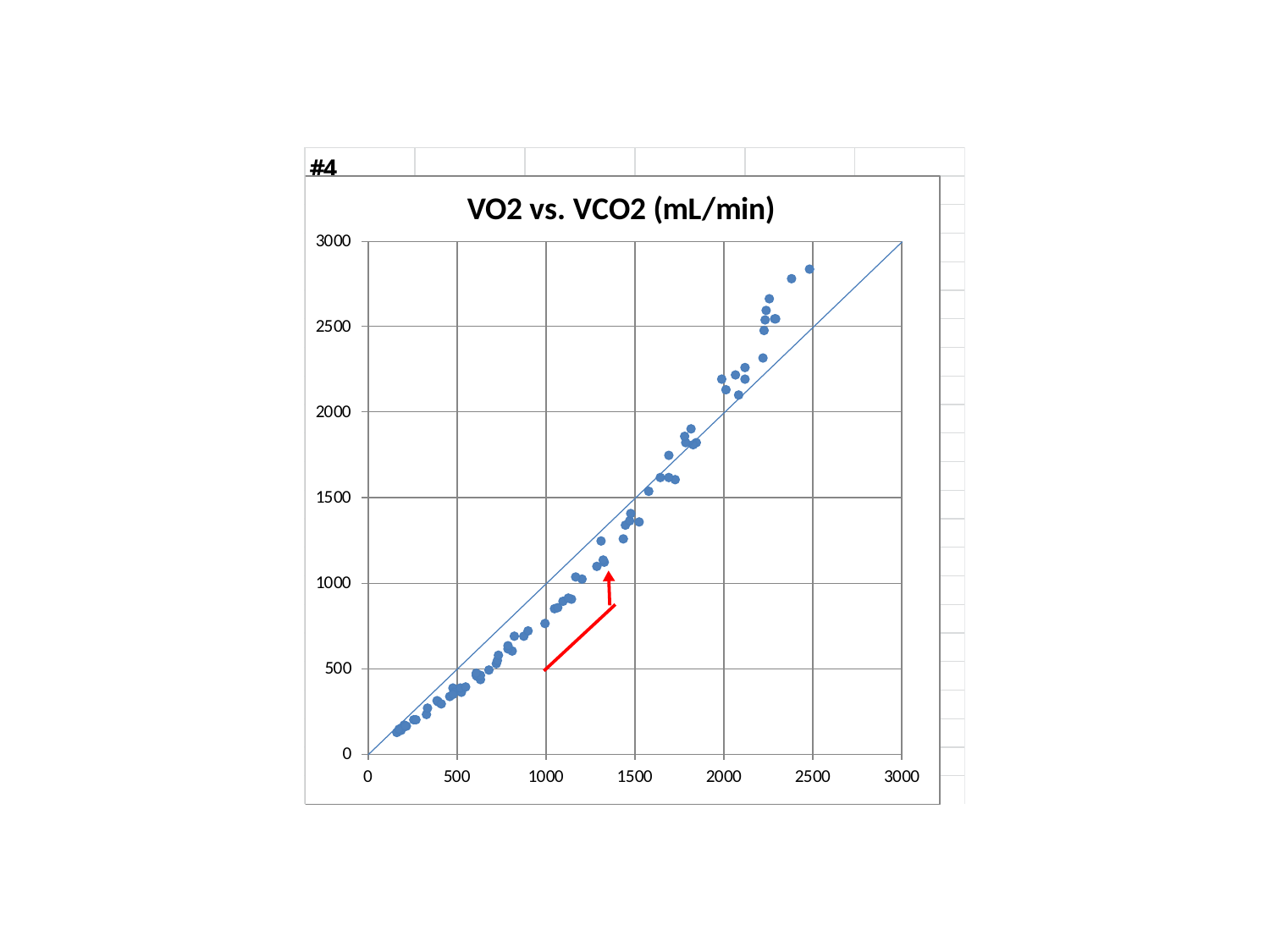

## Slide 7
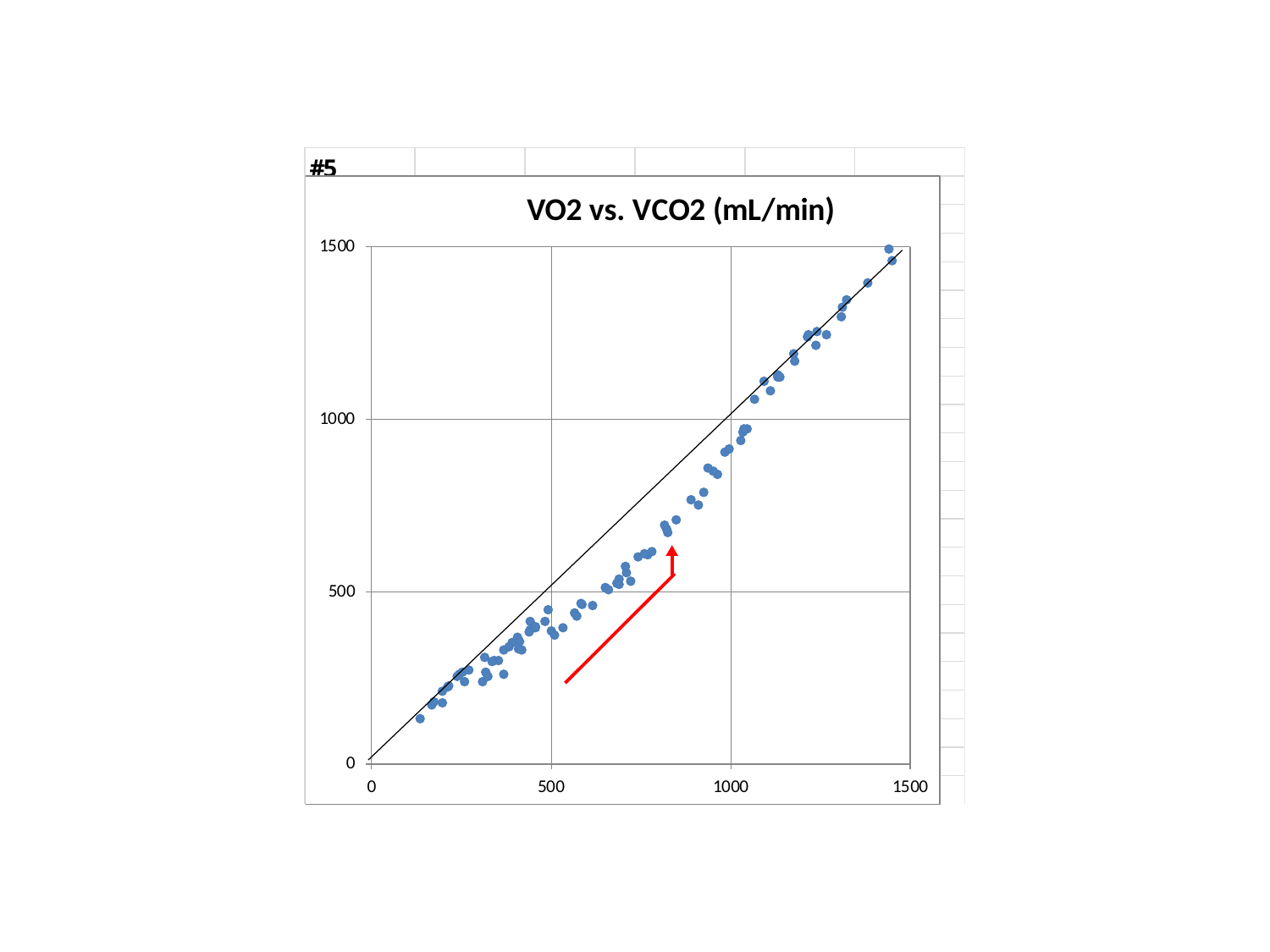

## Slide 8
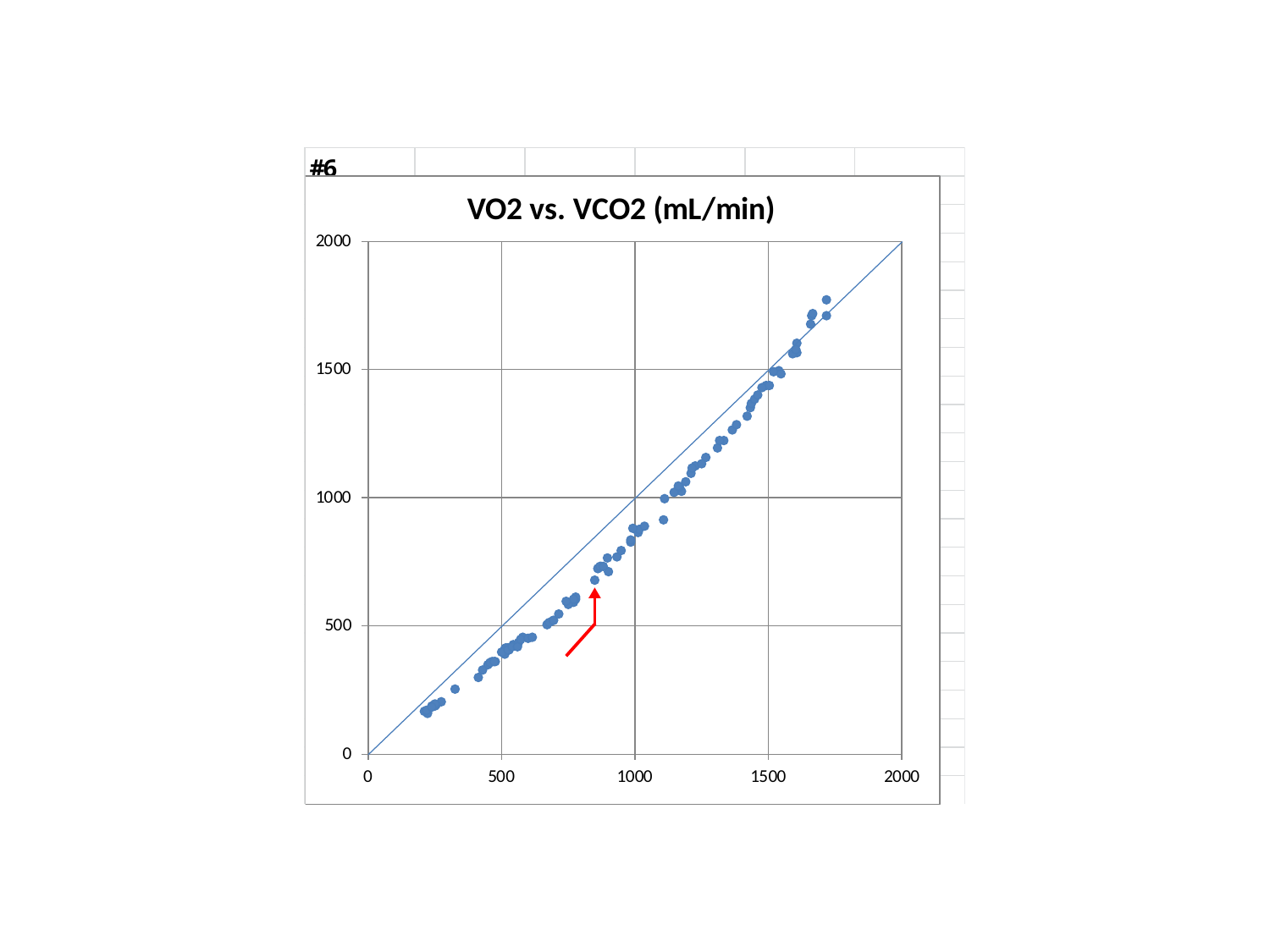

## Slide 9
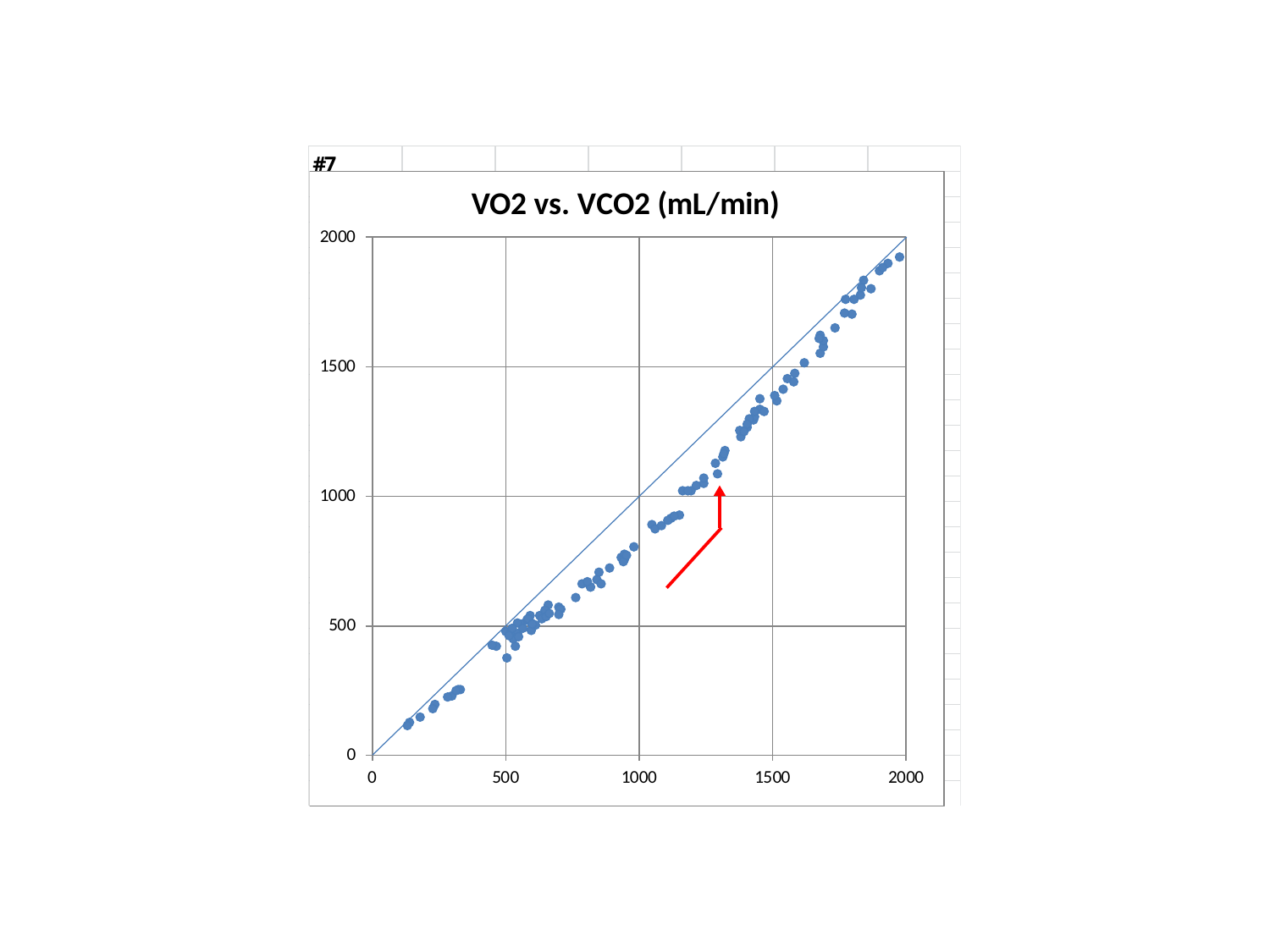

## Slide 10
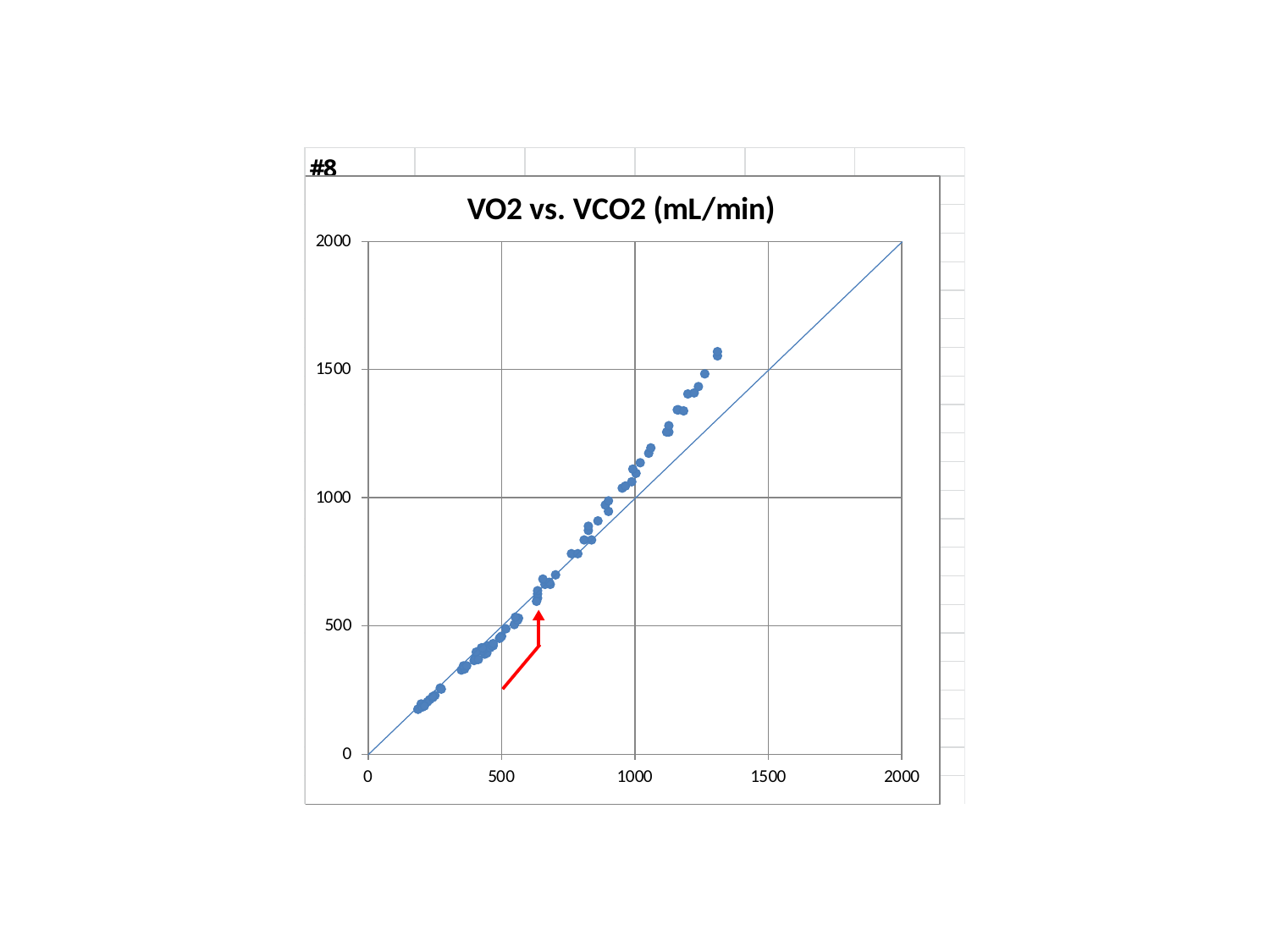

## Slide 11
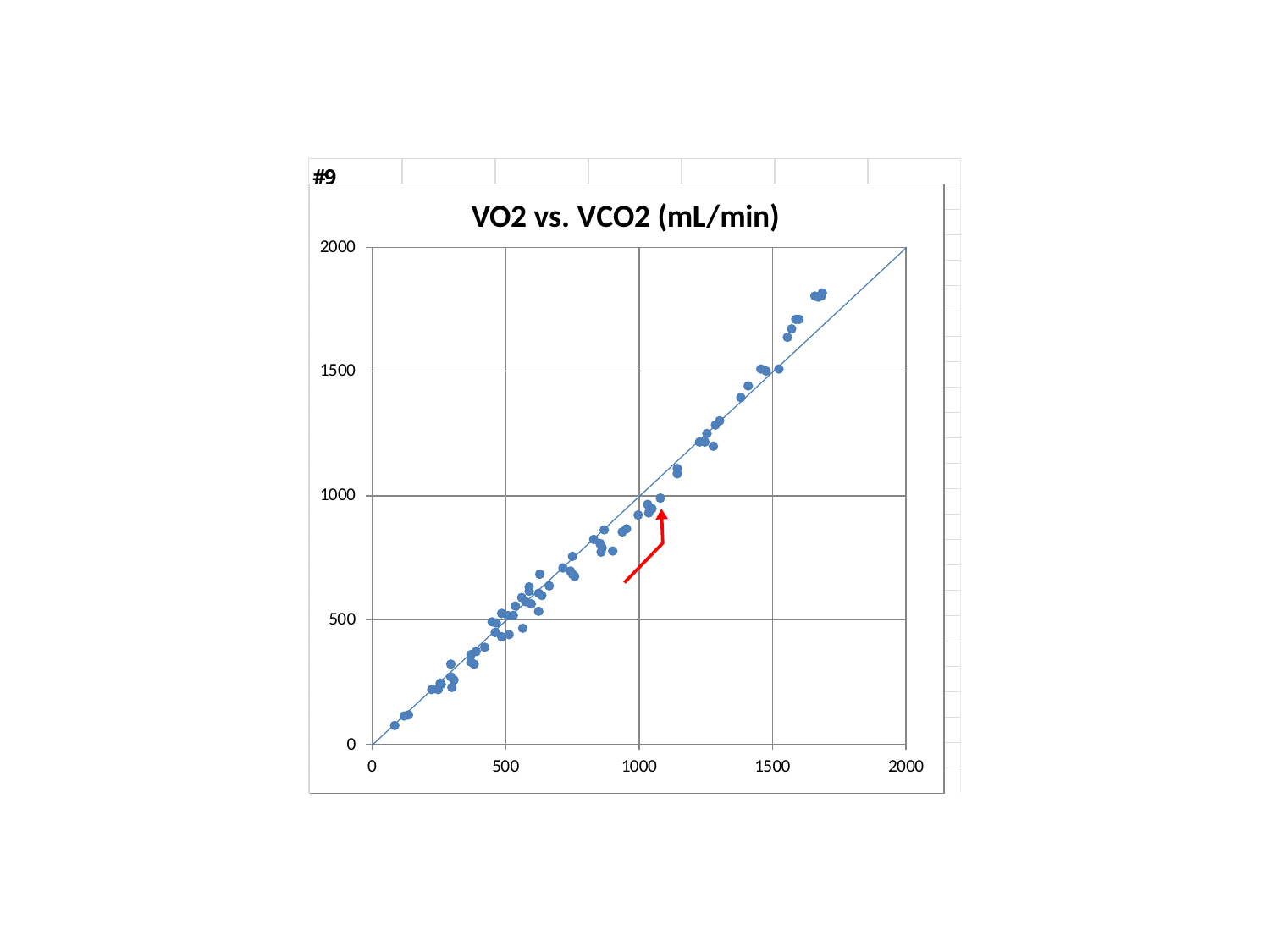

## Slide 12
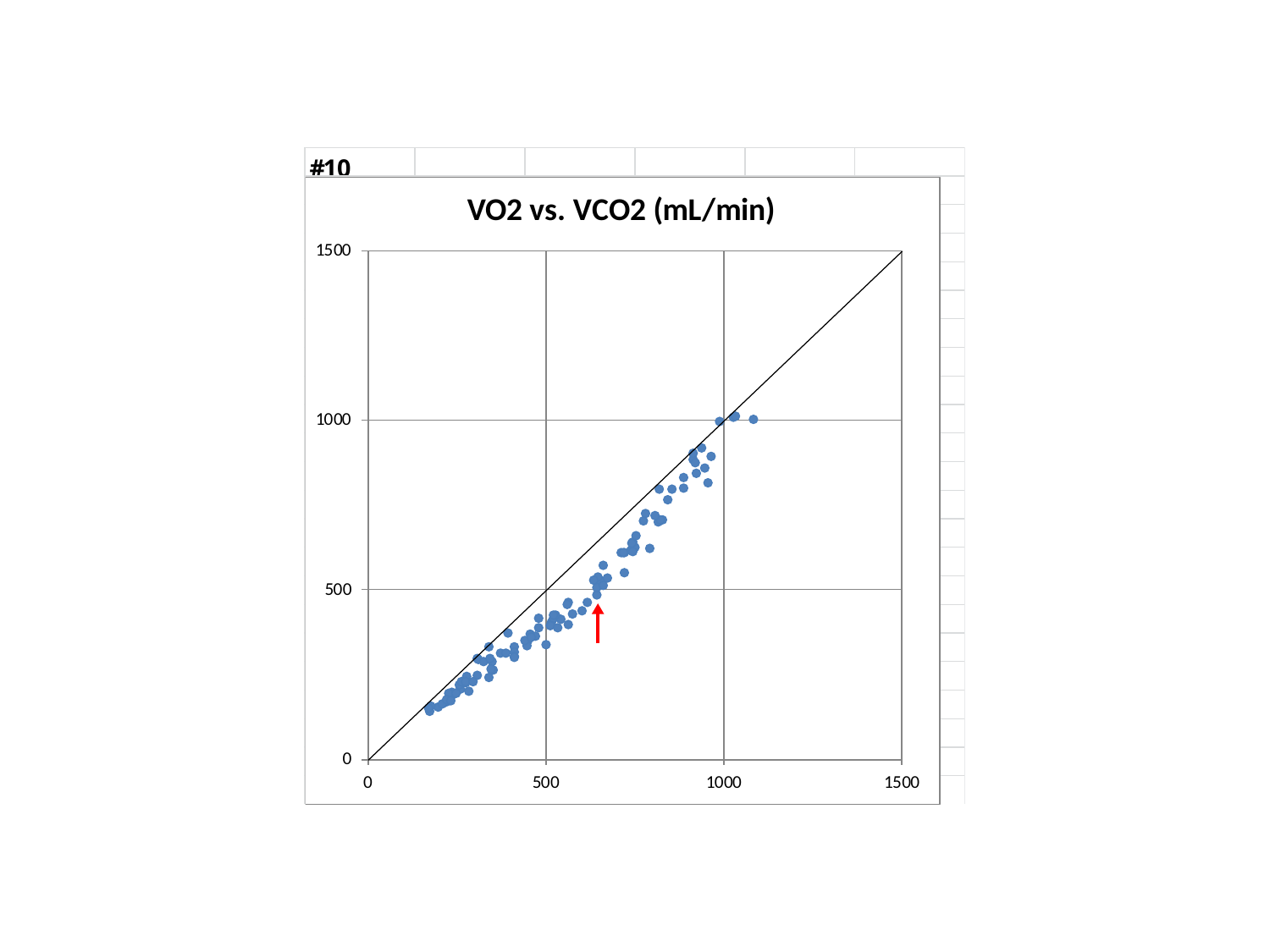

## Slide 13
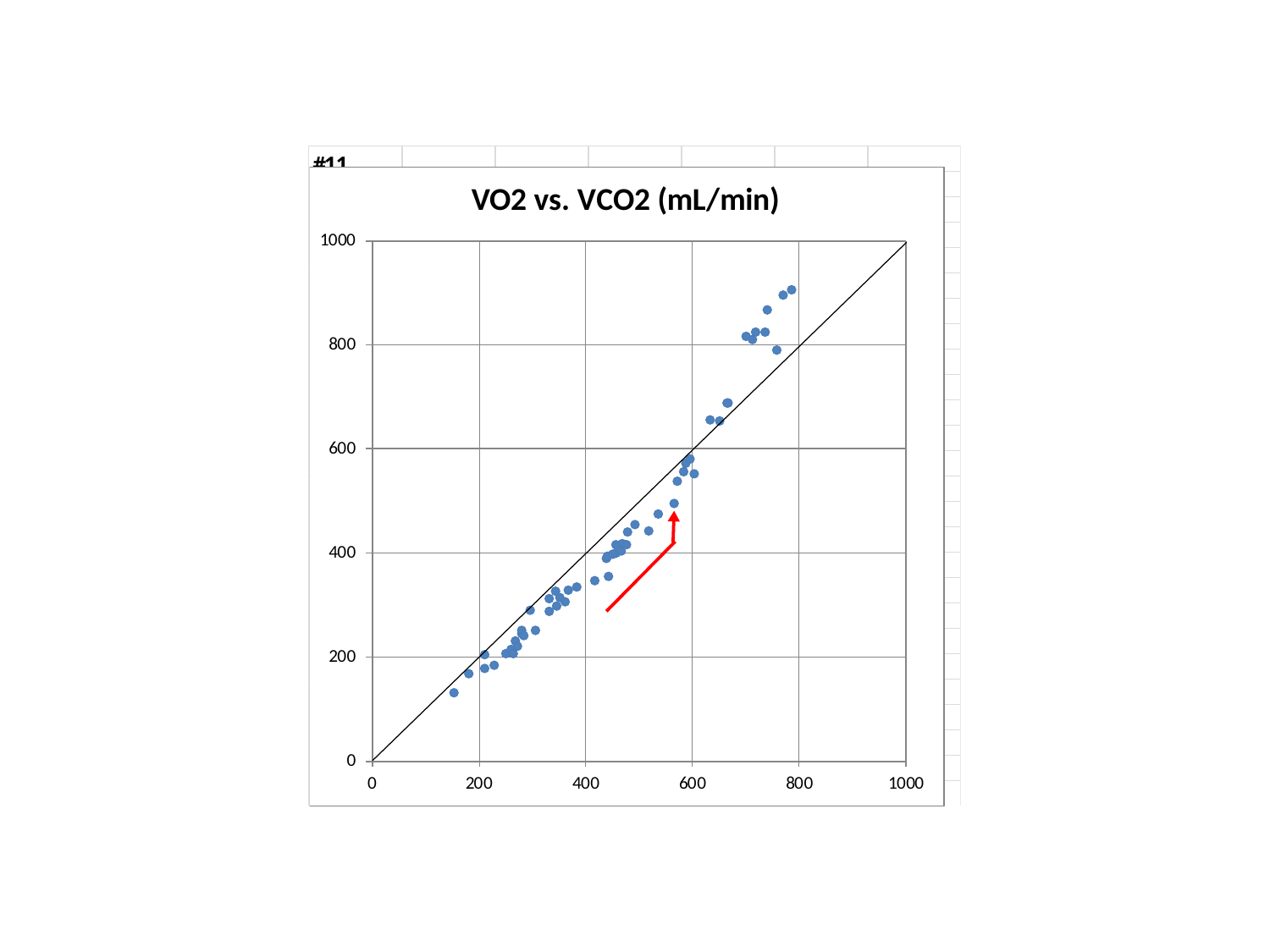

## Slide 14
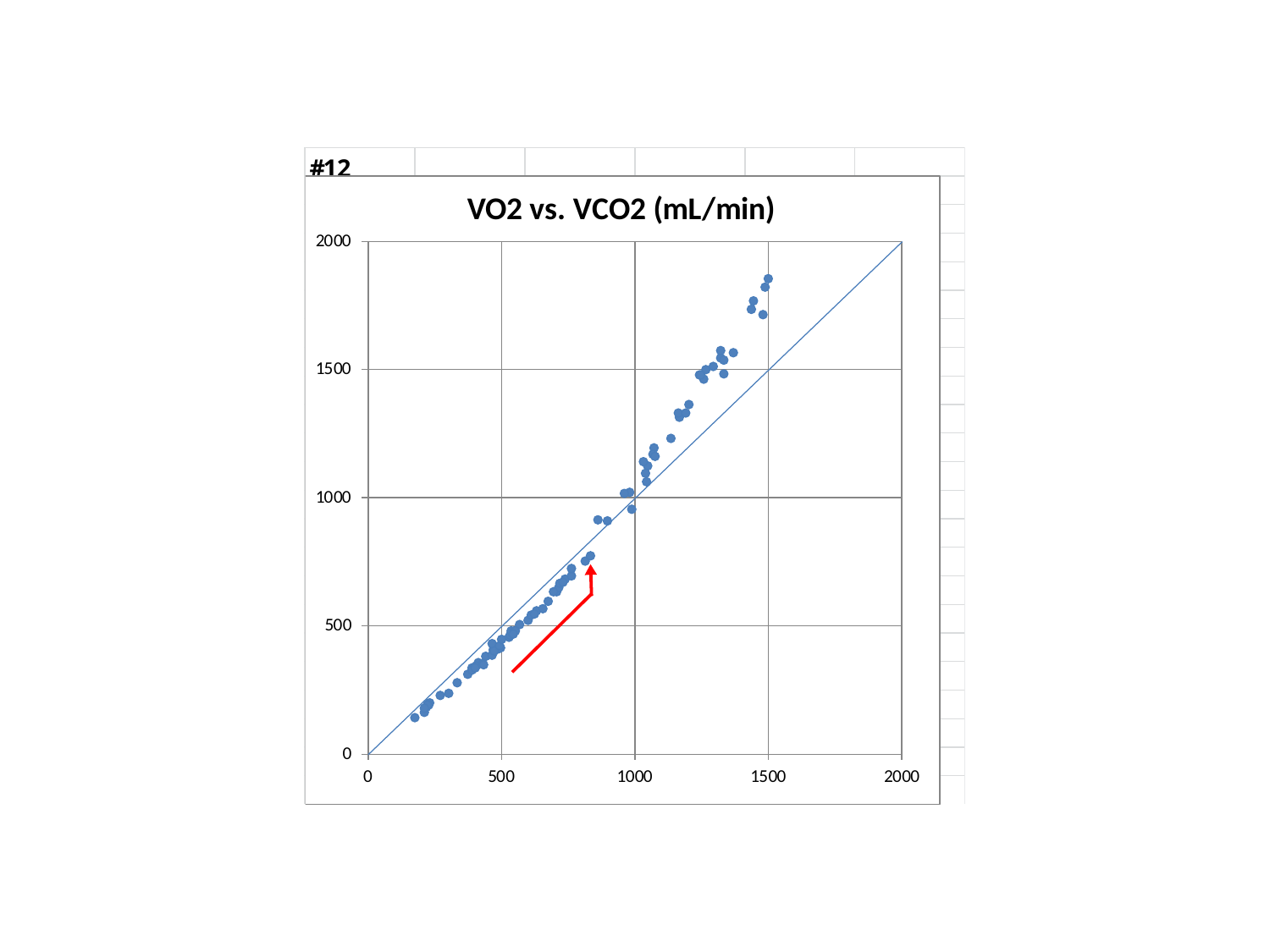

## Slide 15
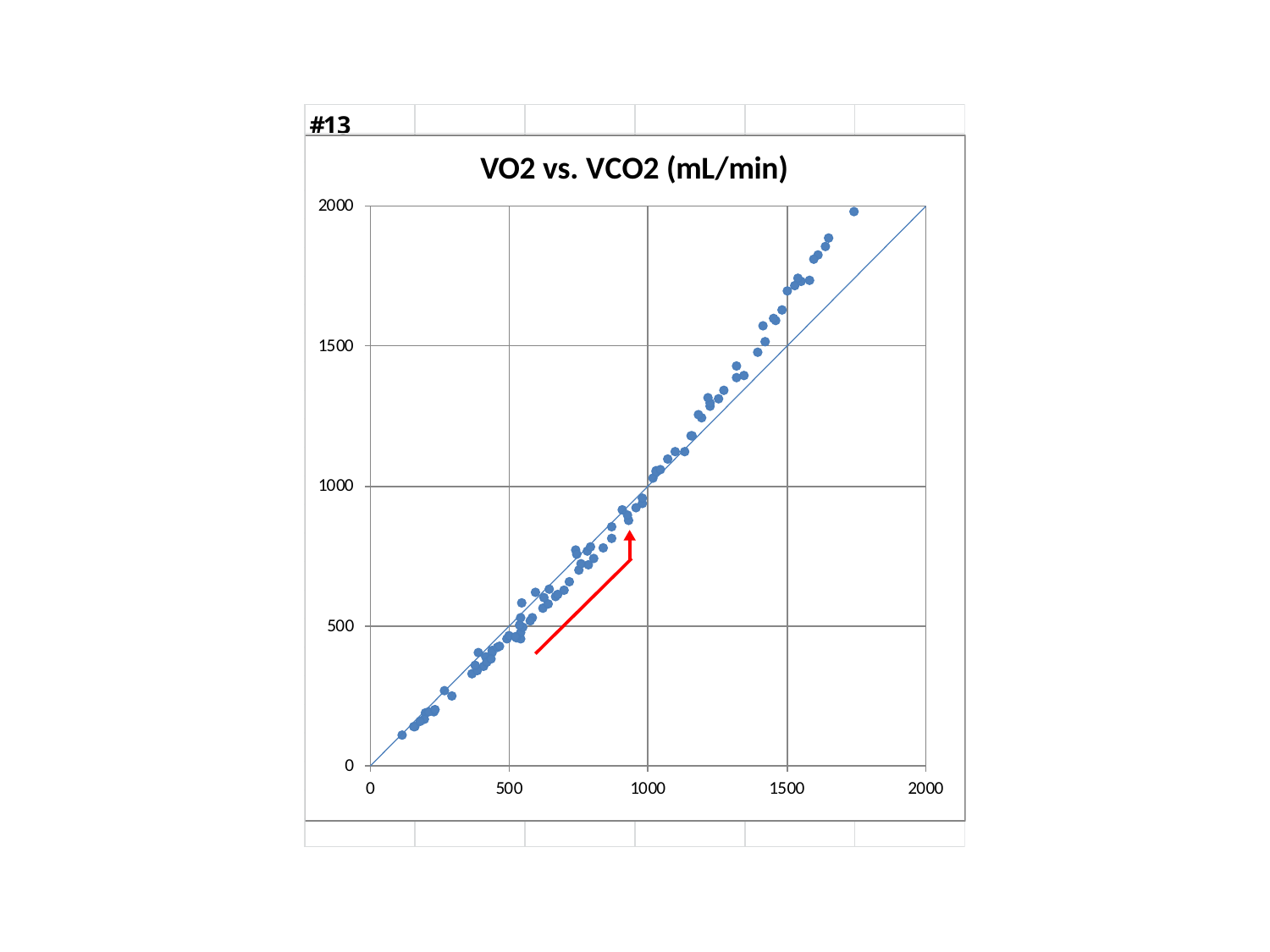

## Slide 16
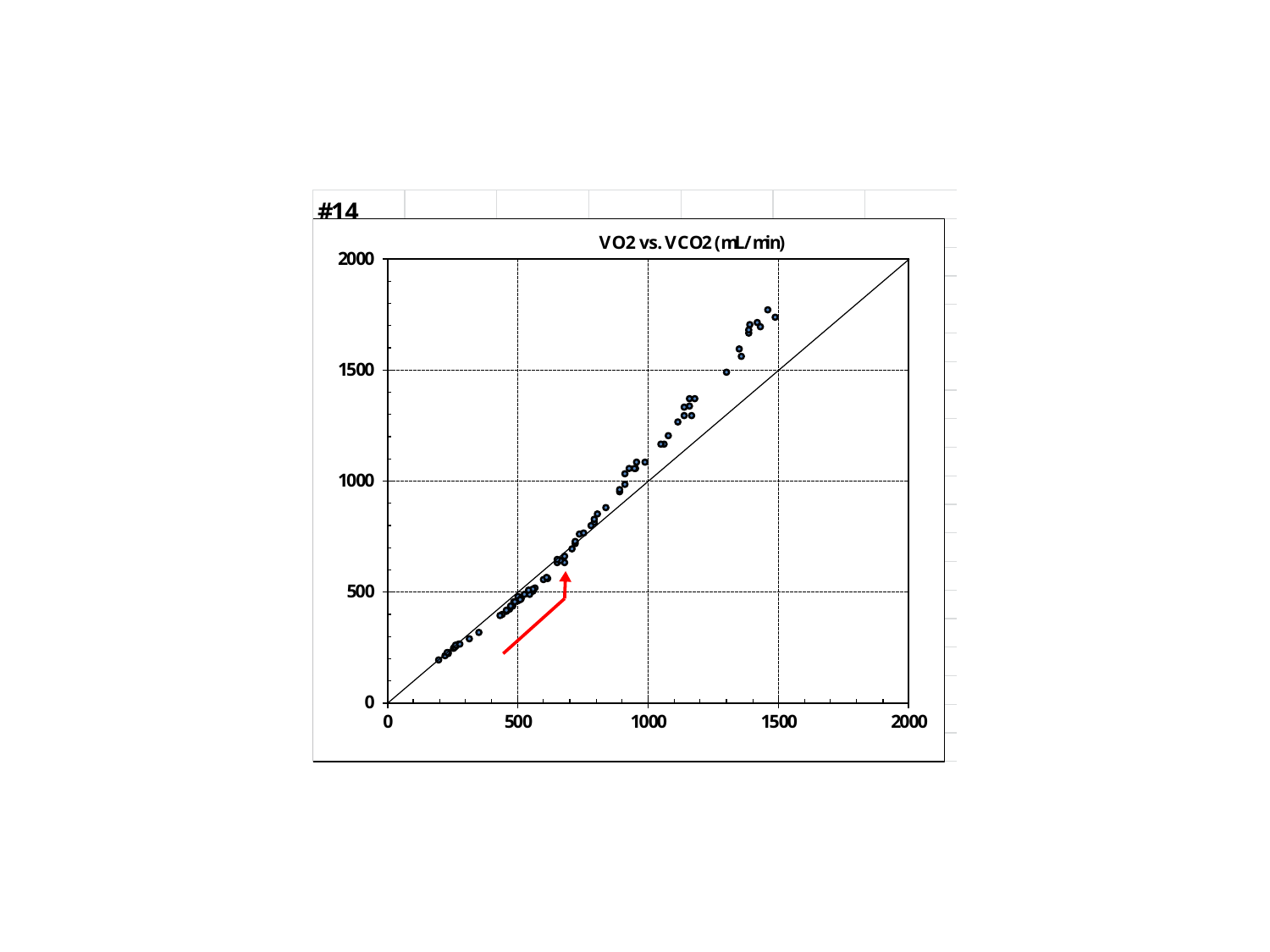

## Slide 17
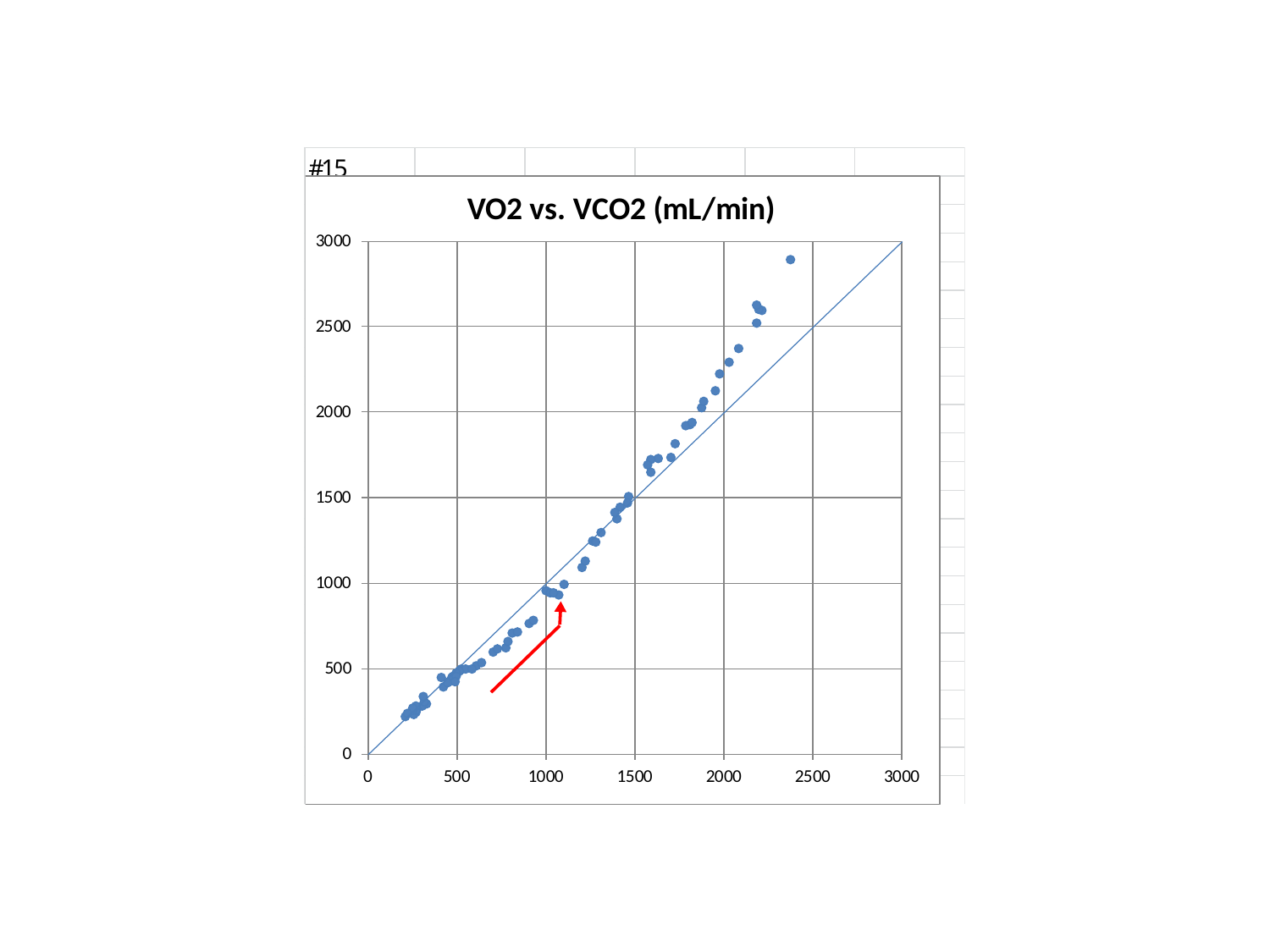

## Slide 18
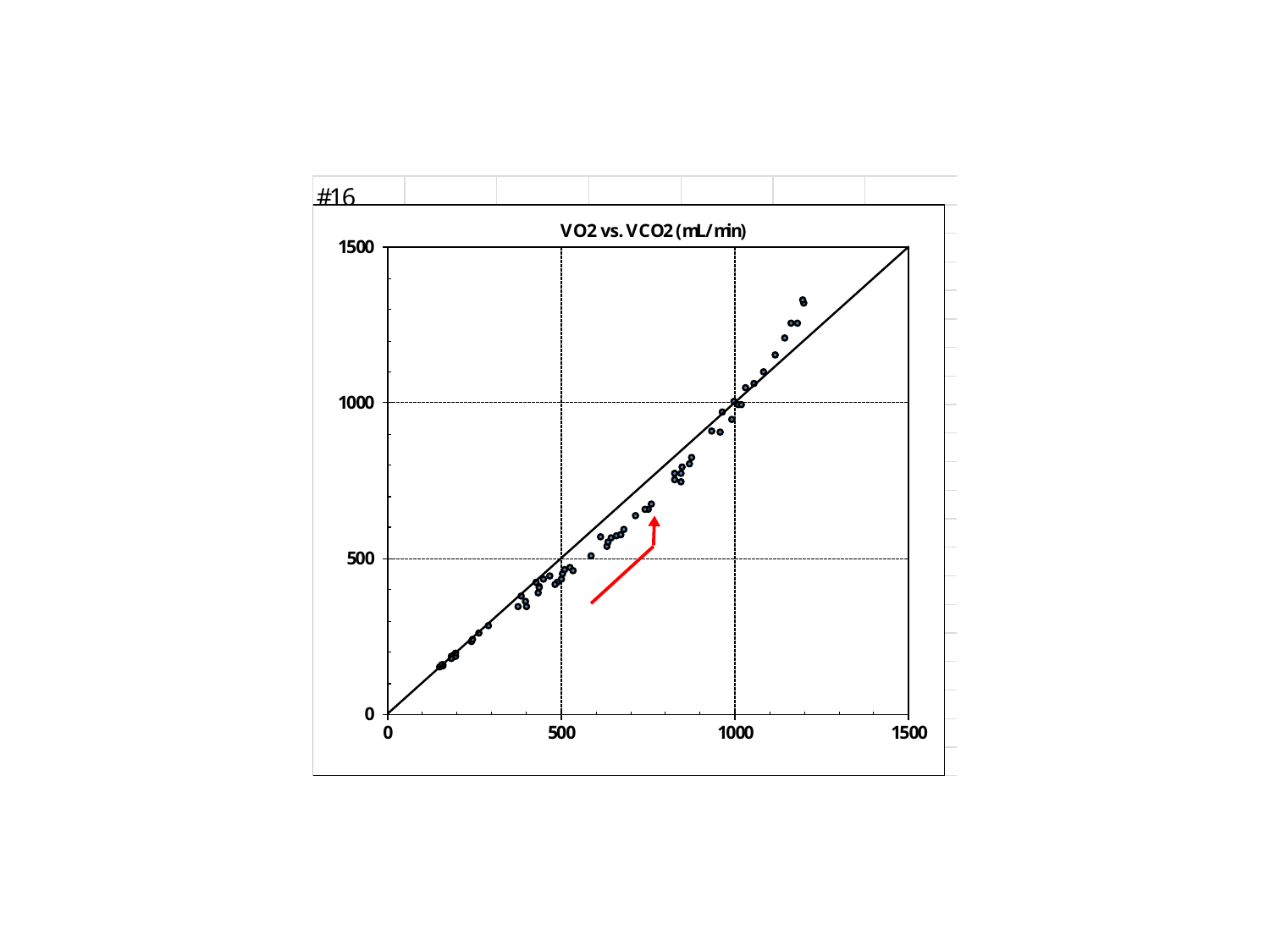

## Slide 19
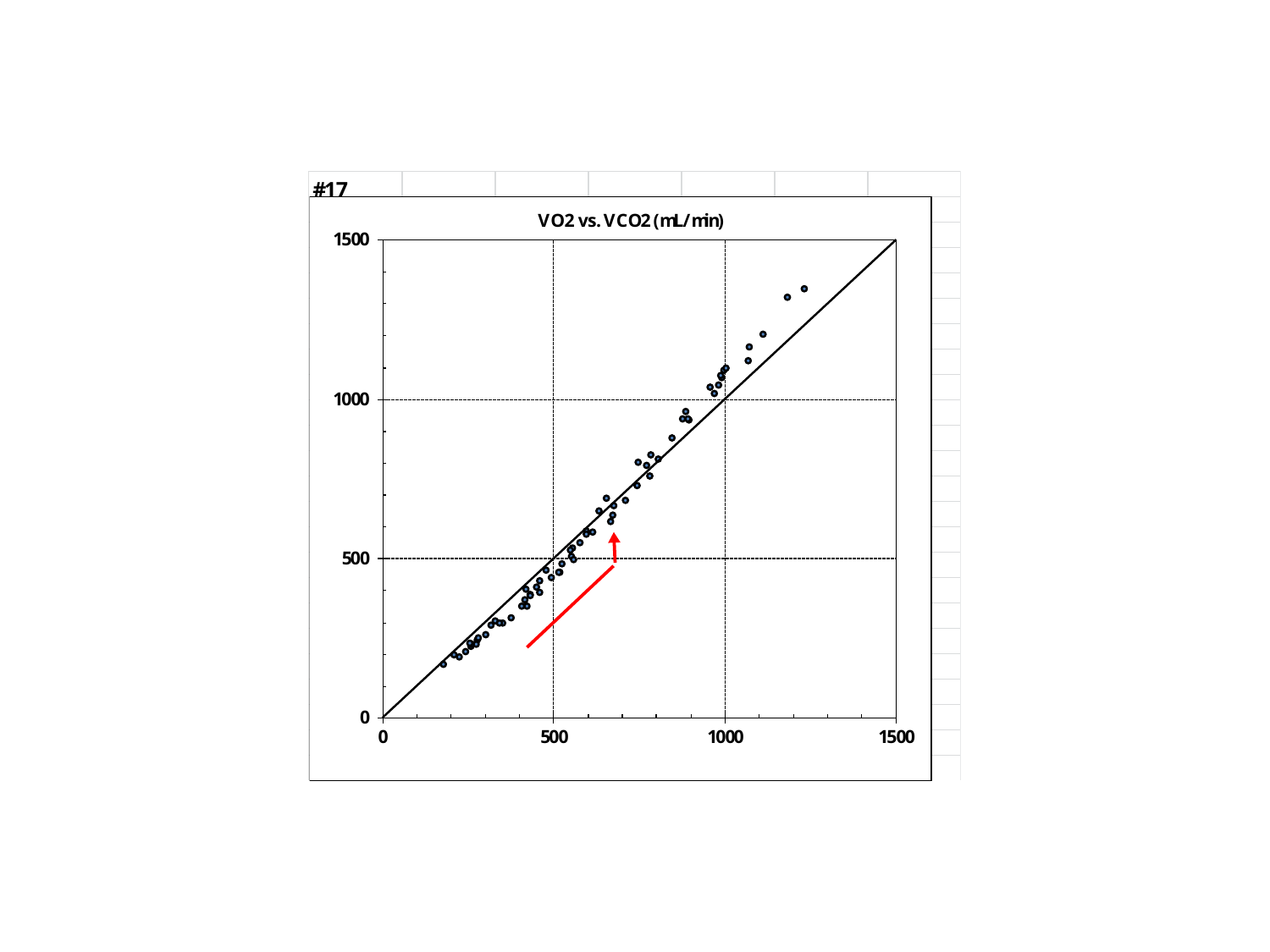

## Slide 20
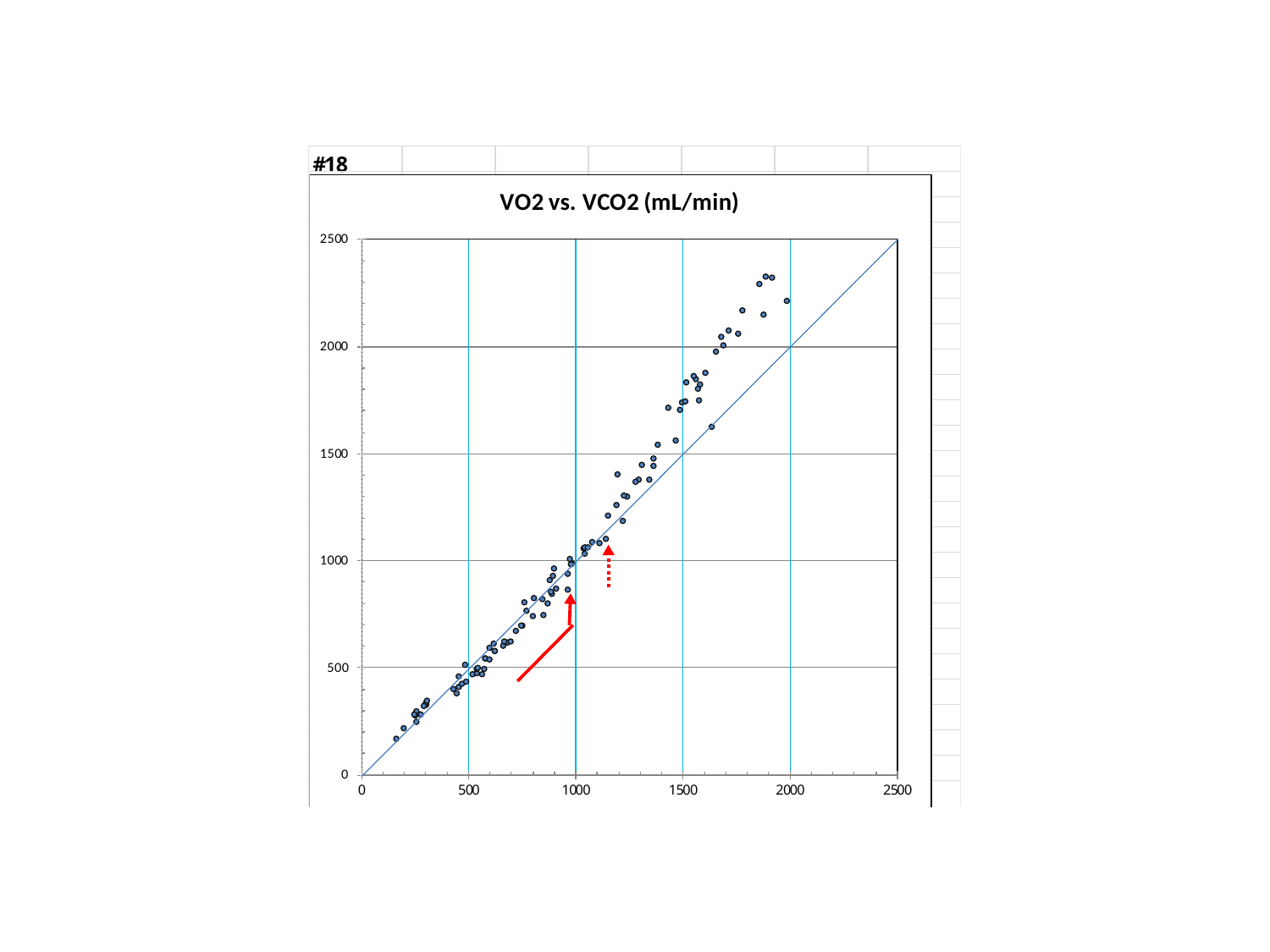

## Slide 21
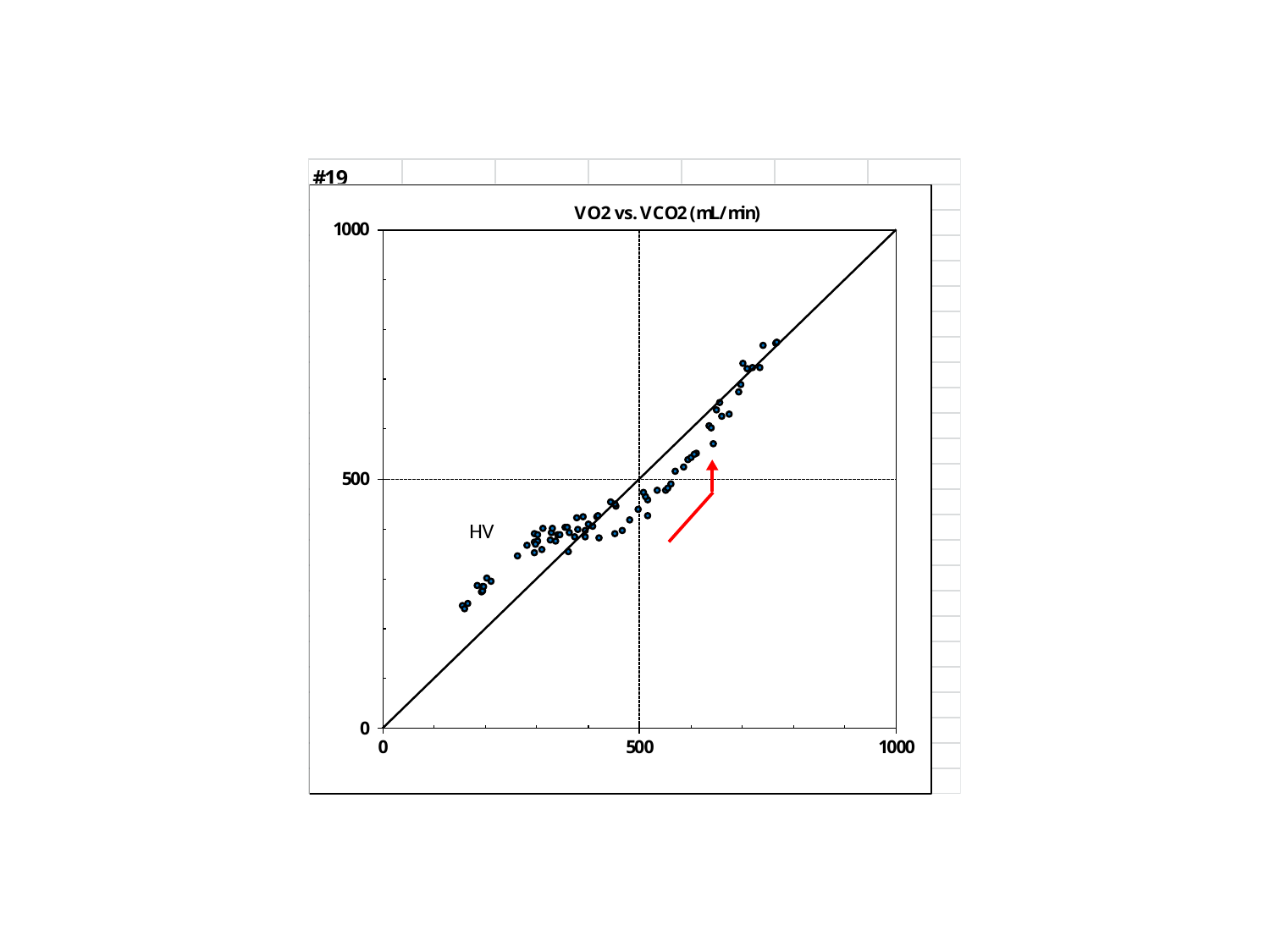

## Slide 22
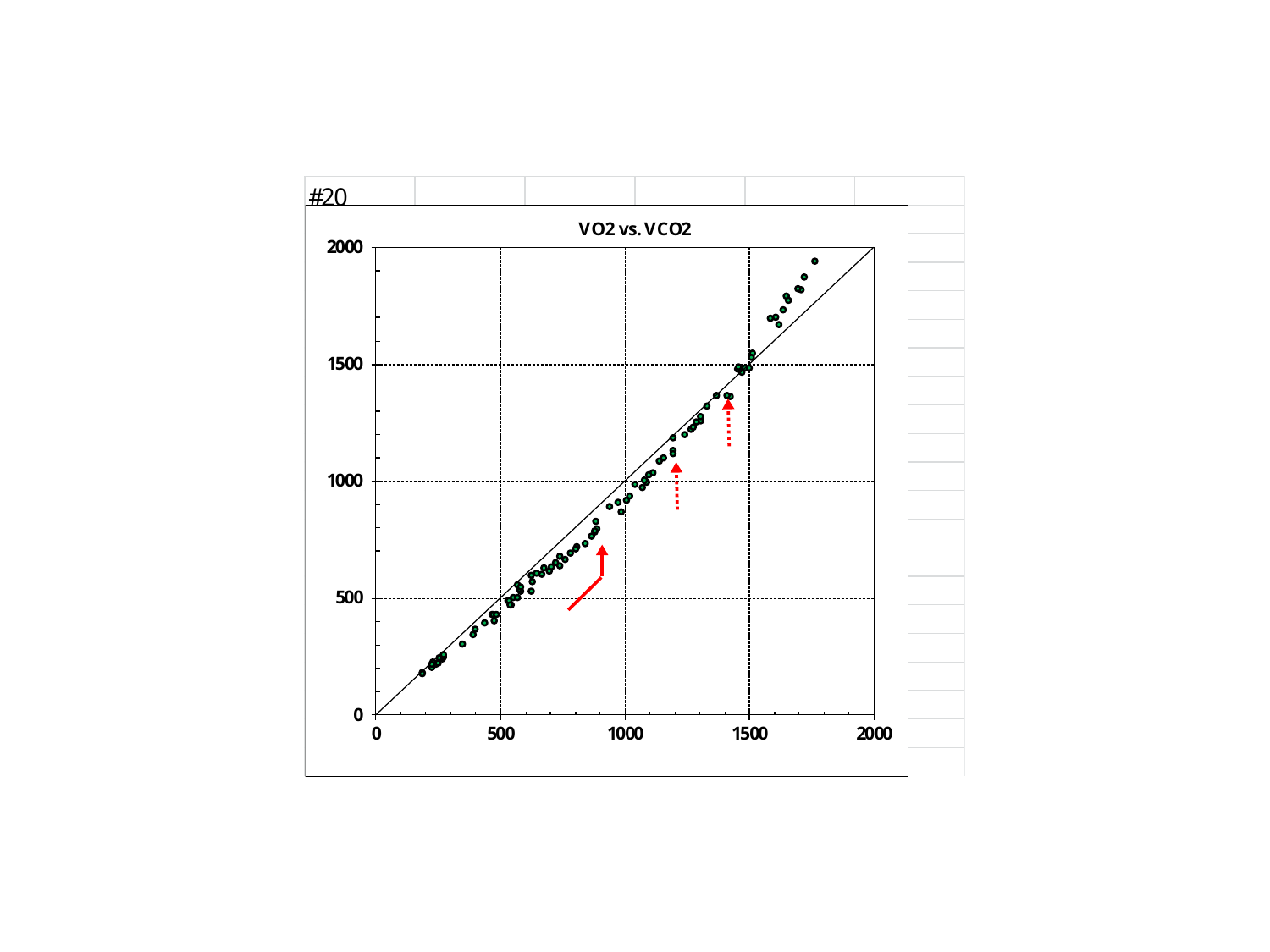

## Slide 23
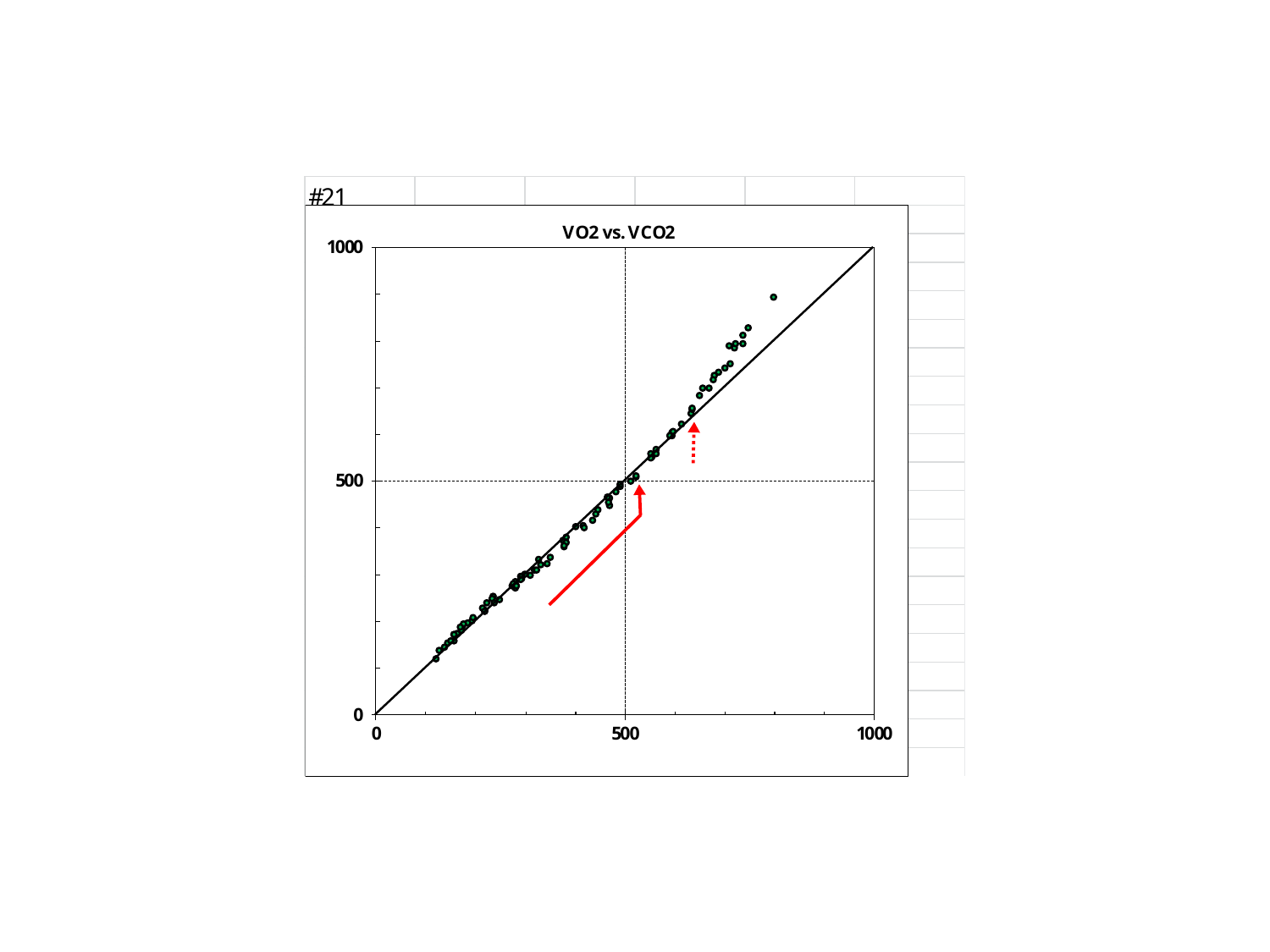

## Slide 24
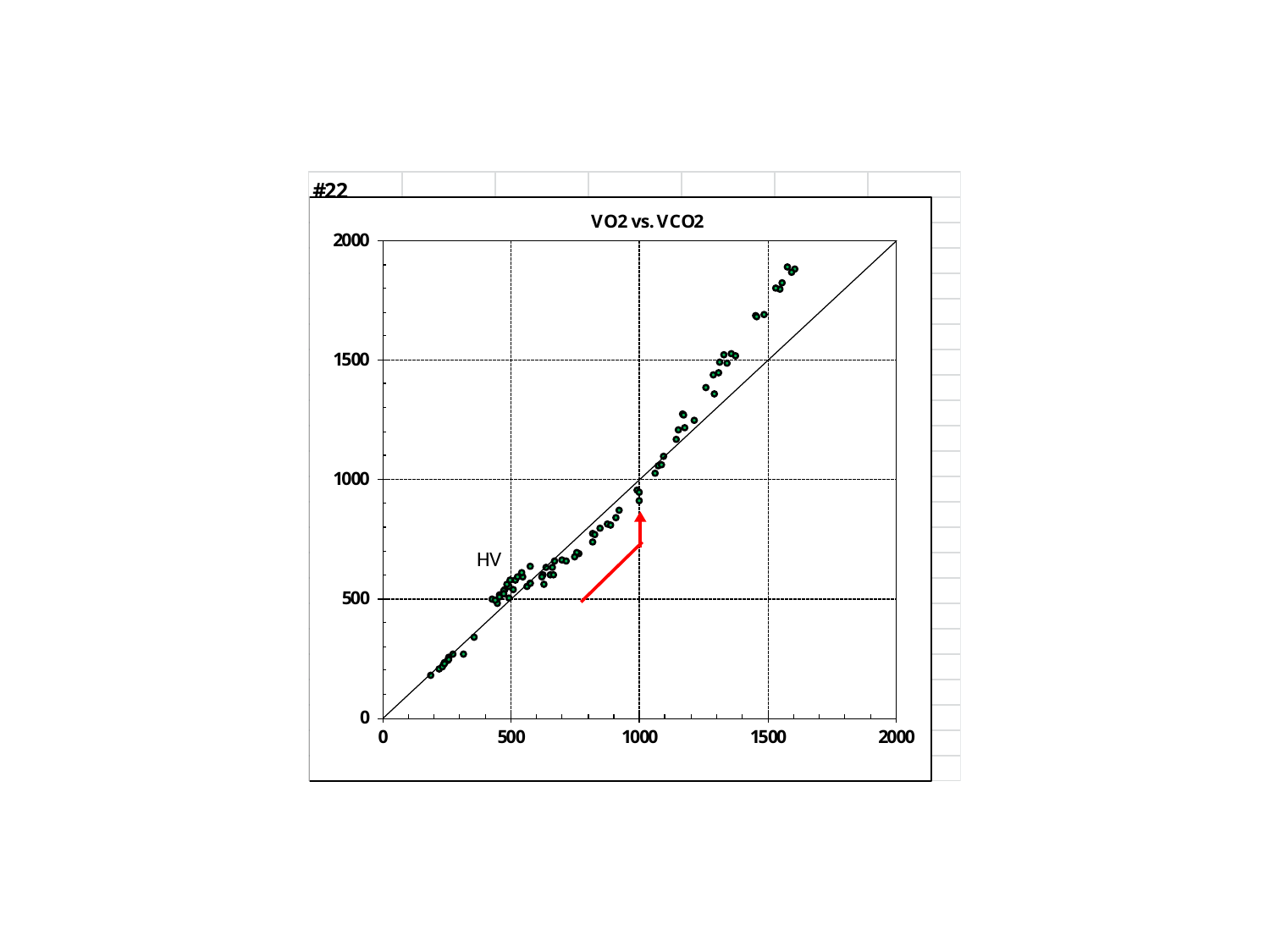

## Slide 25
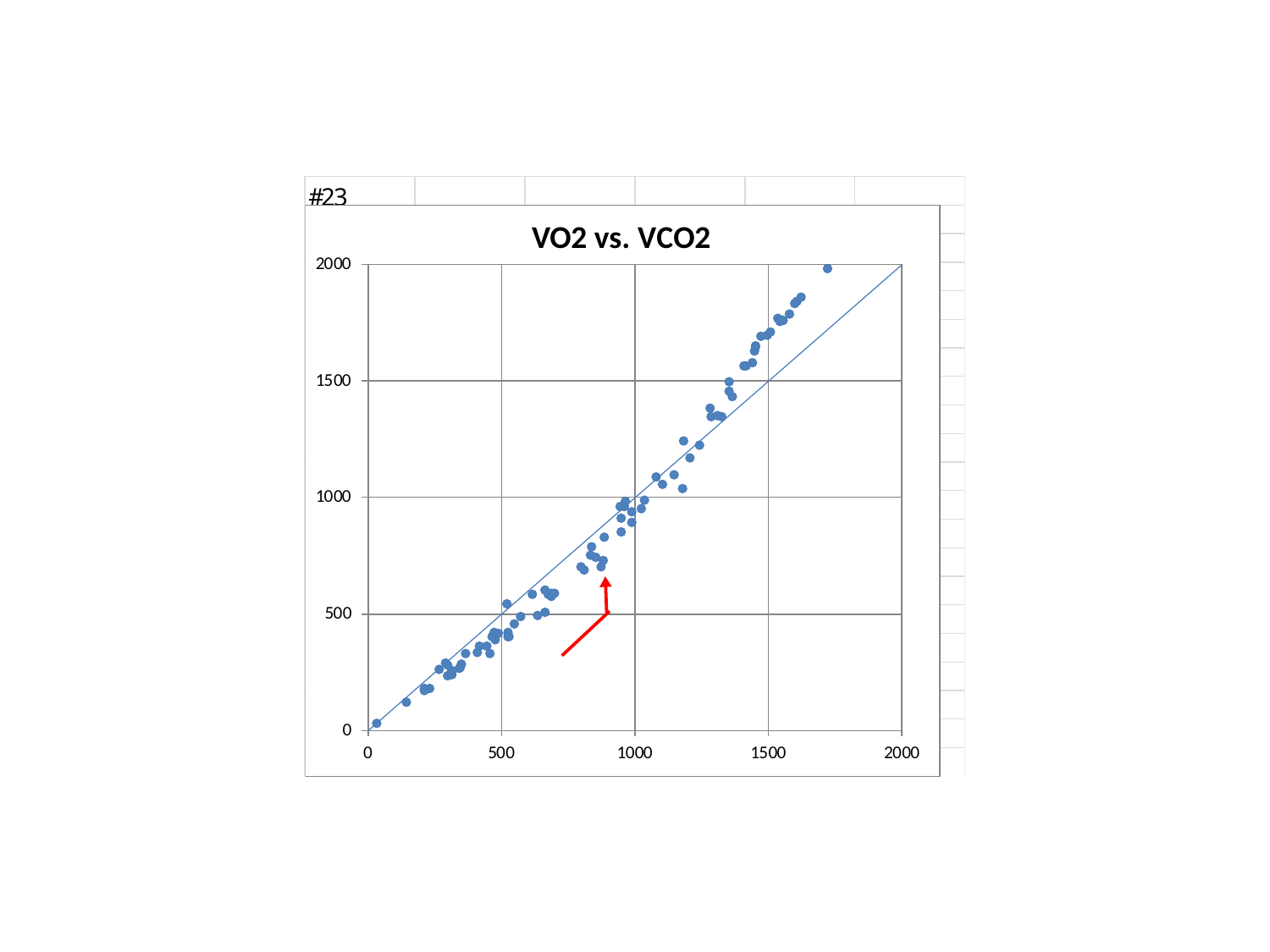

## Slide 26
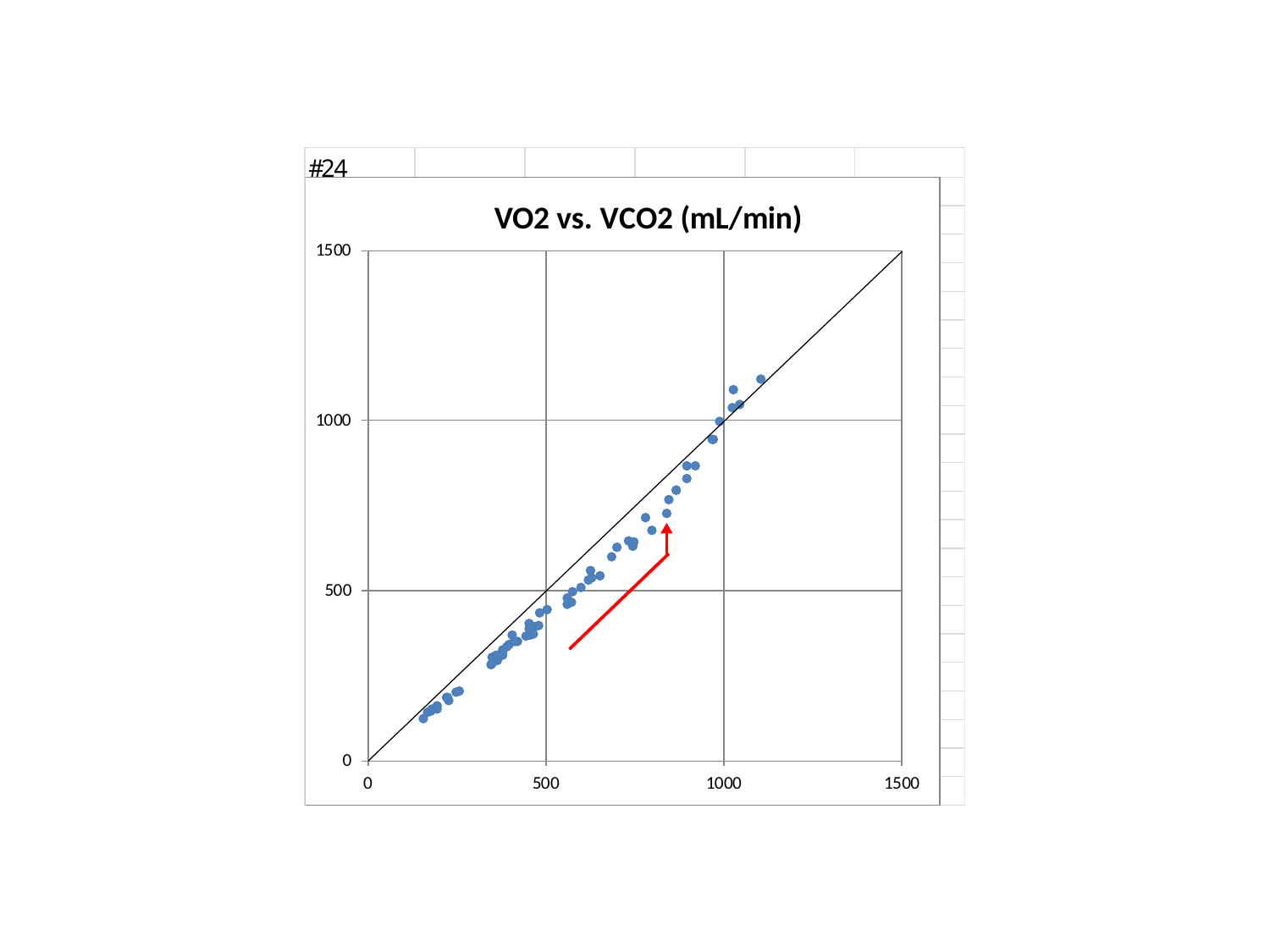

## Slide 27
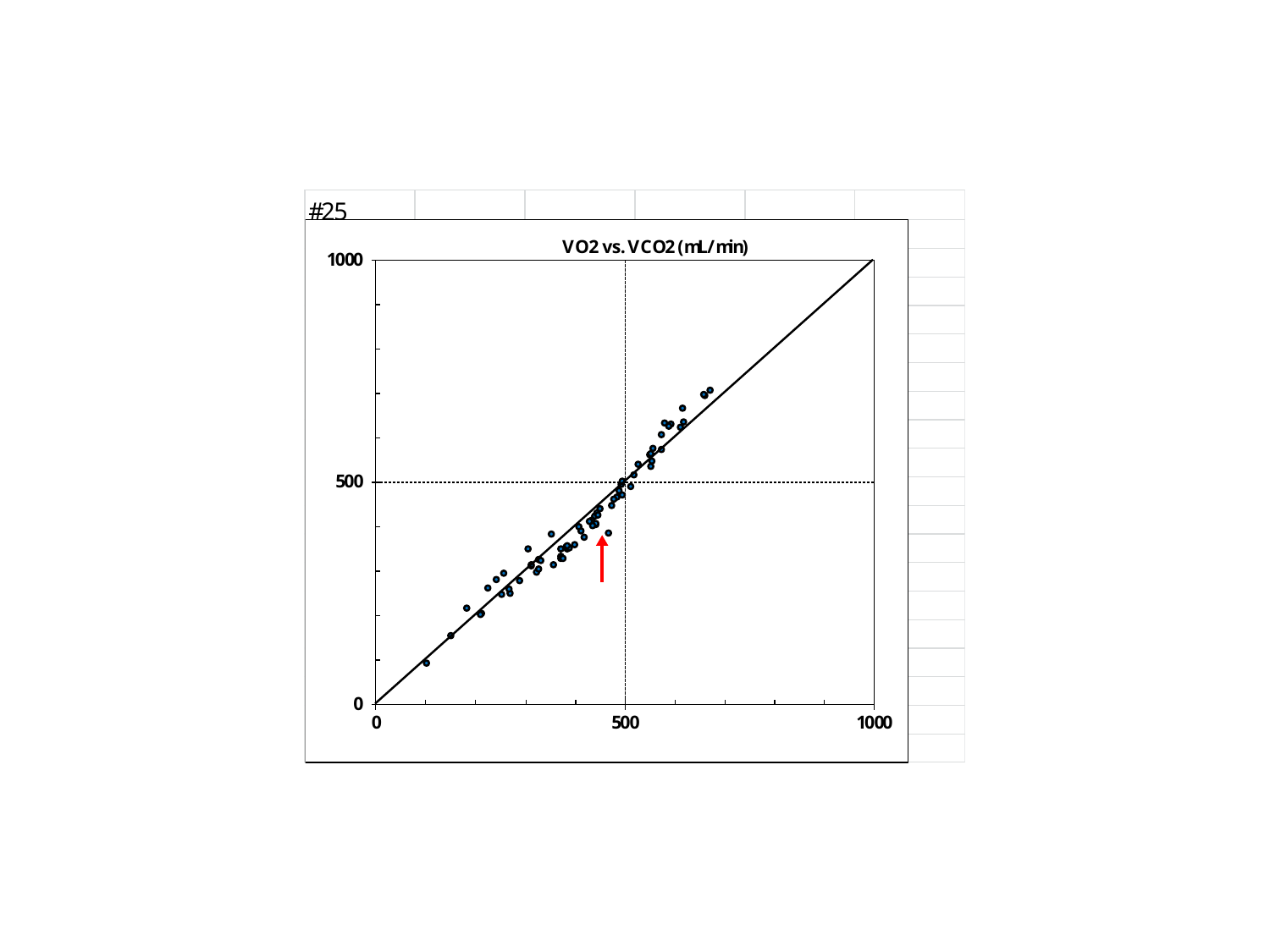

## Slide 28
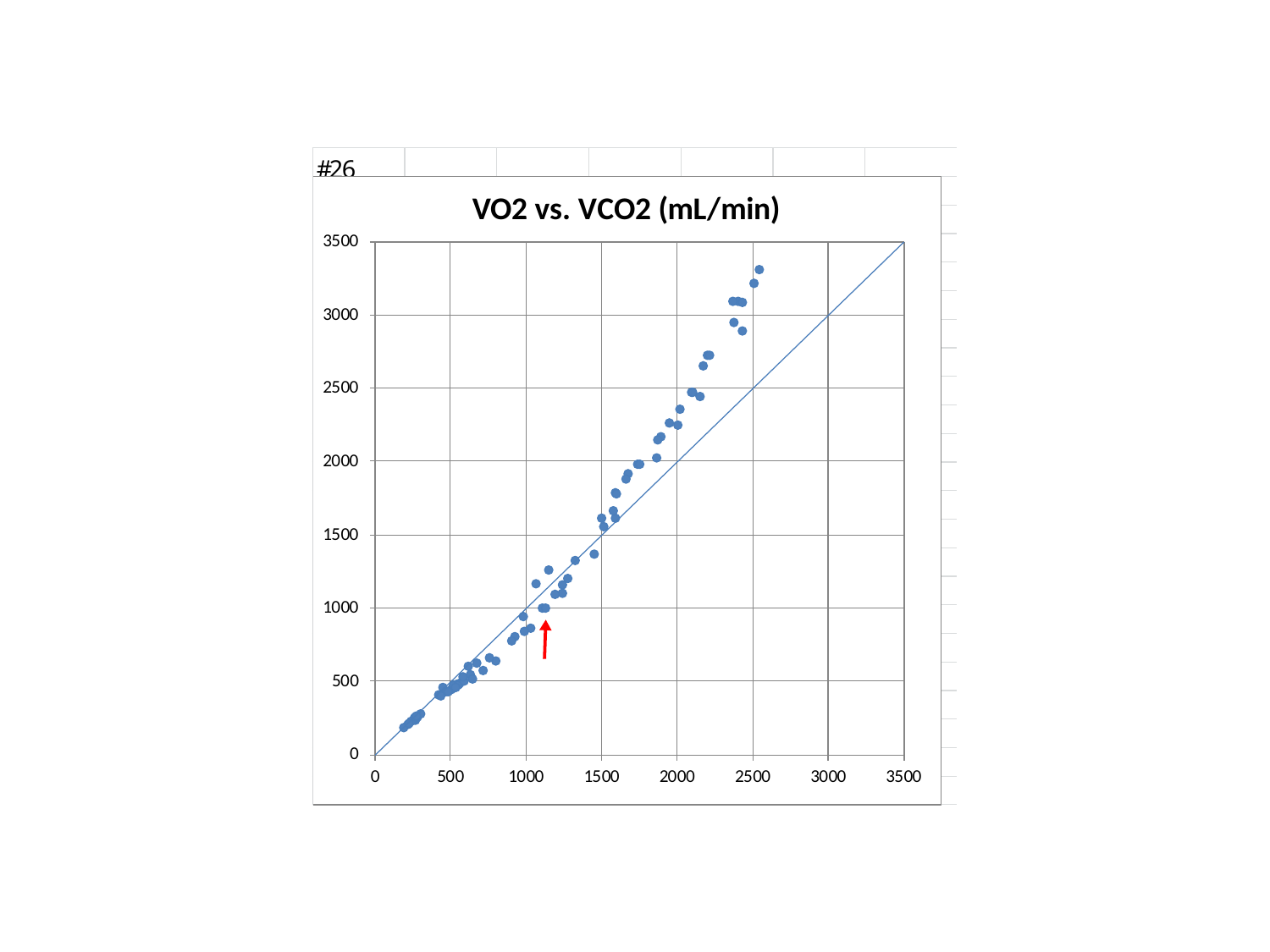

## Slide 29
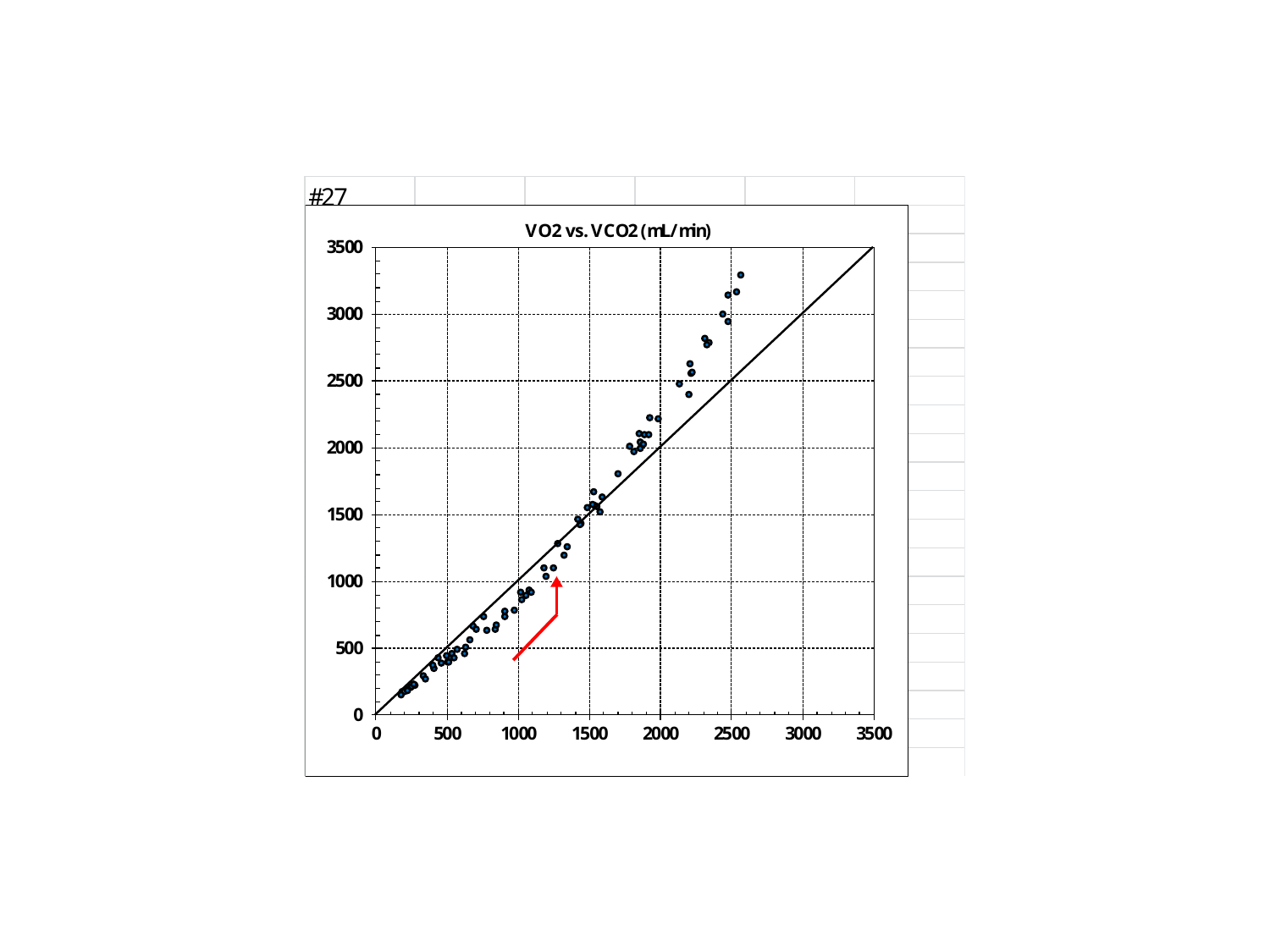

## Slide 30
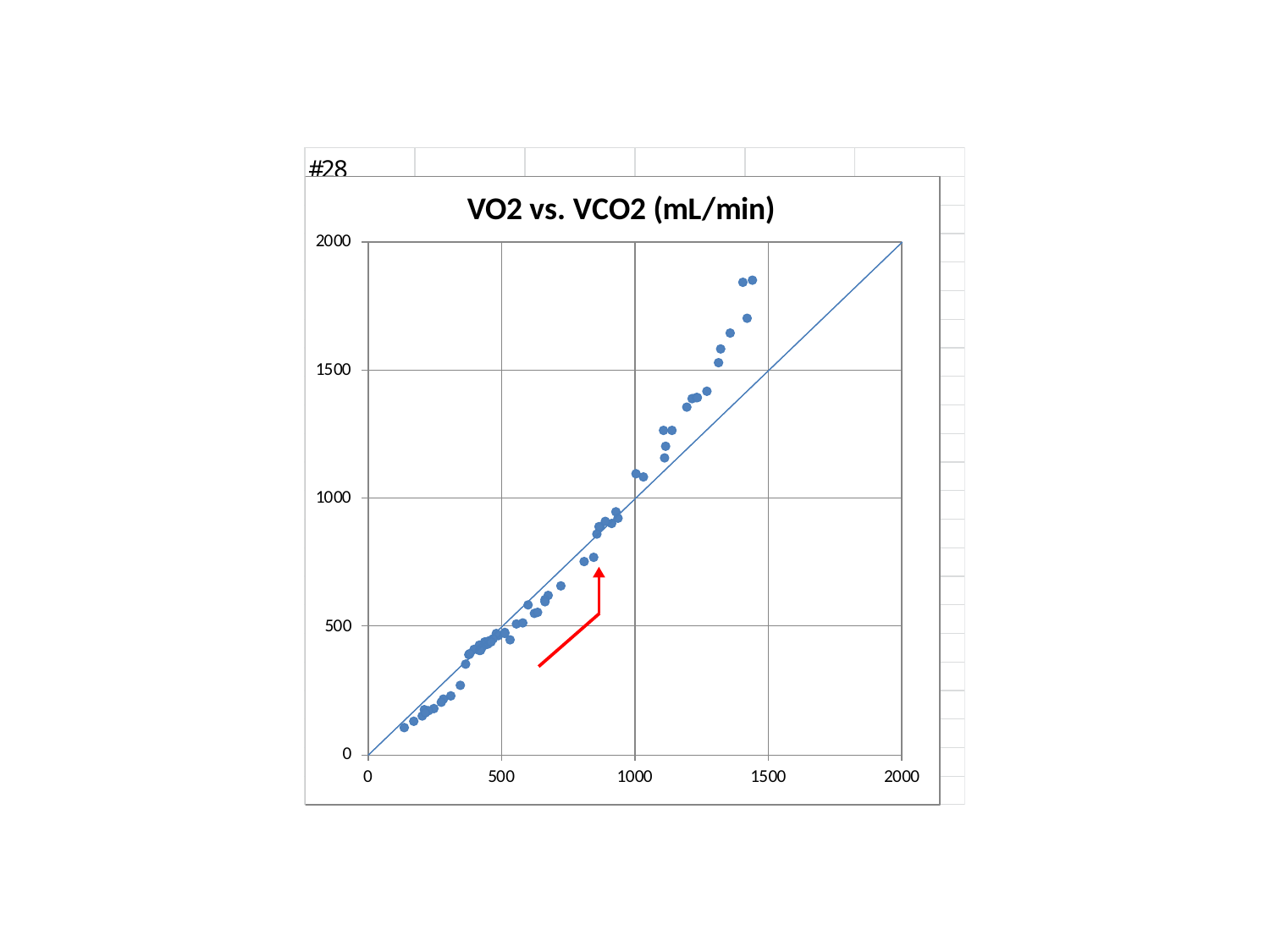

## Slide 31
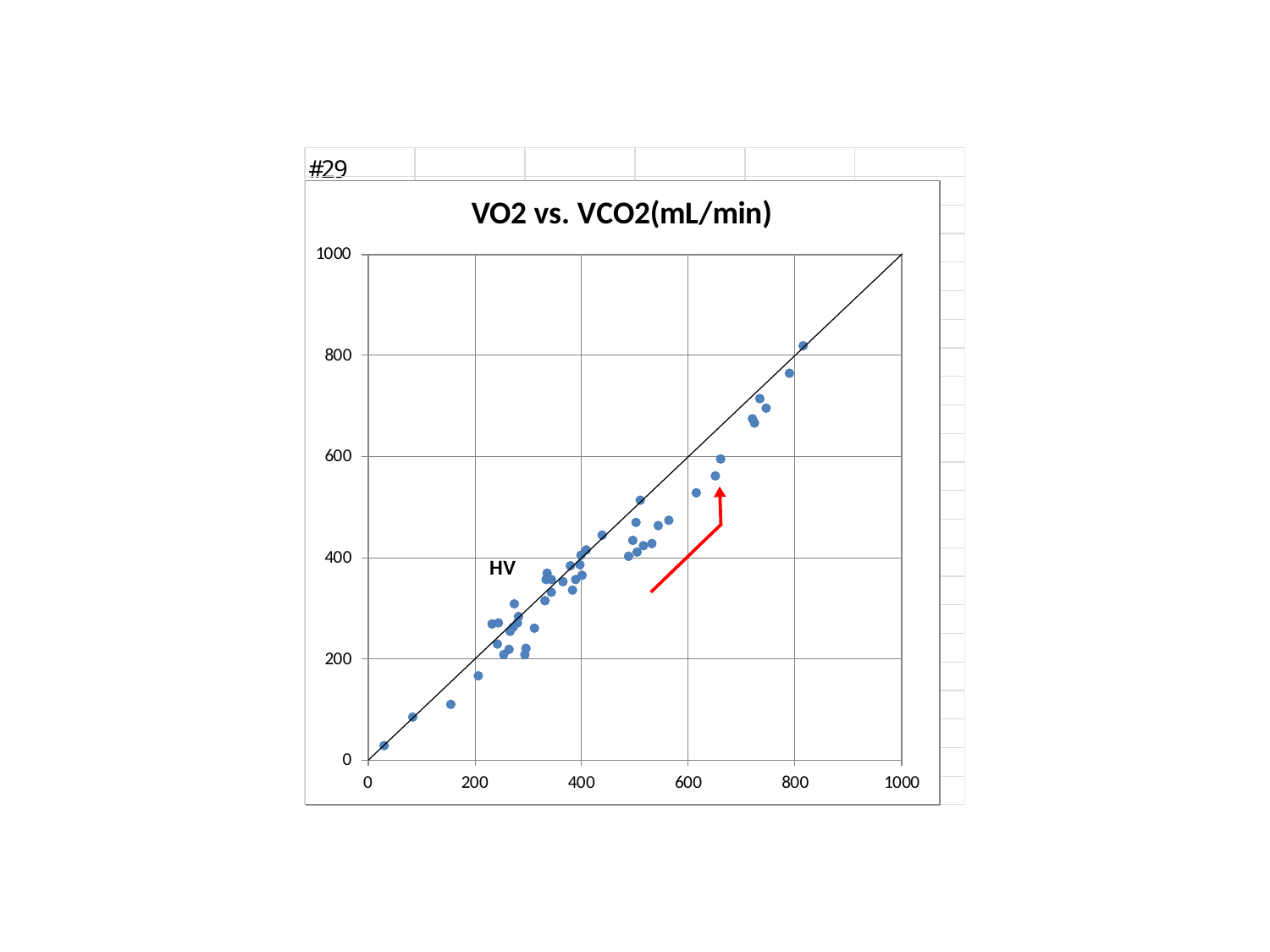

## Slide 32
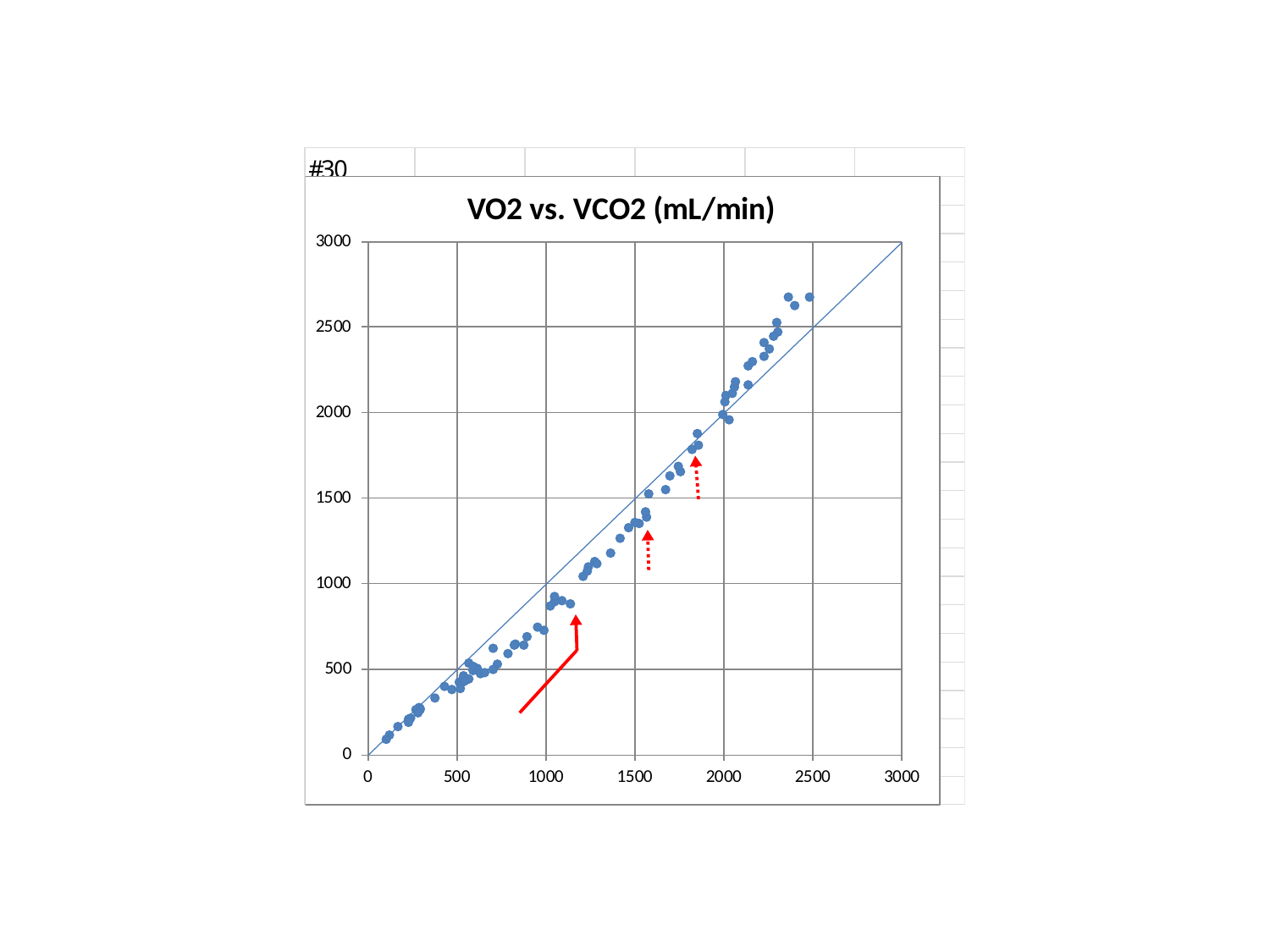

## Slide 33
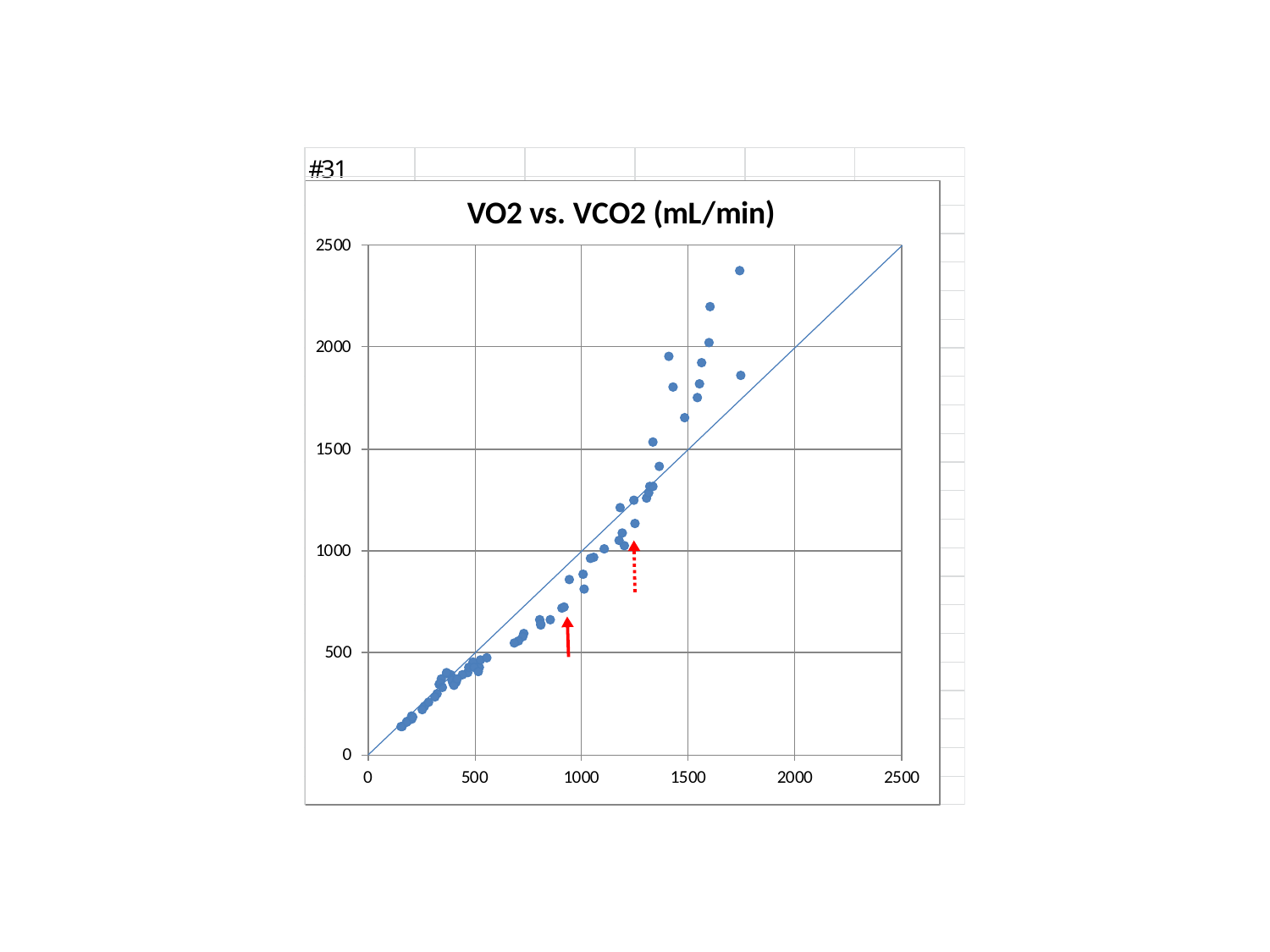

## Slide 34
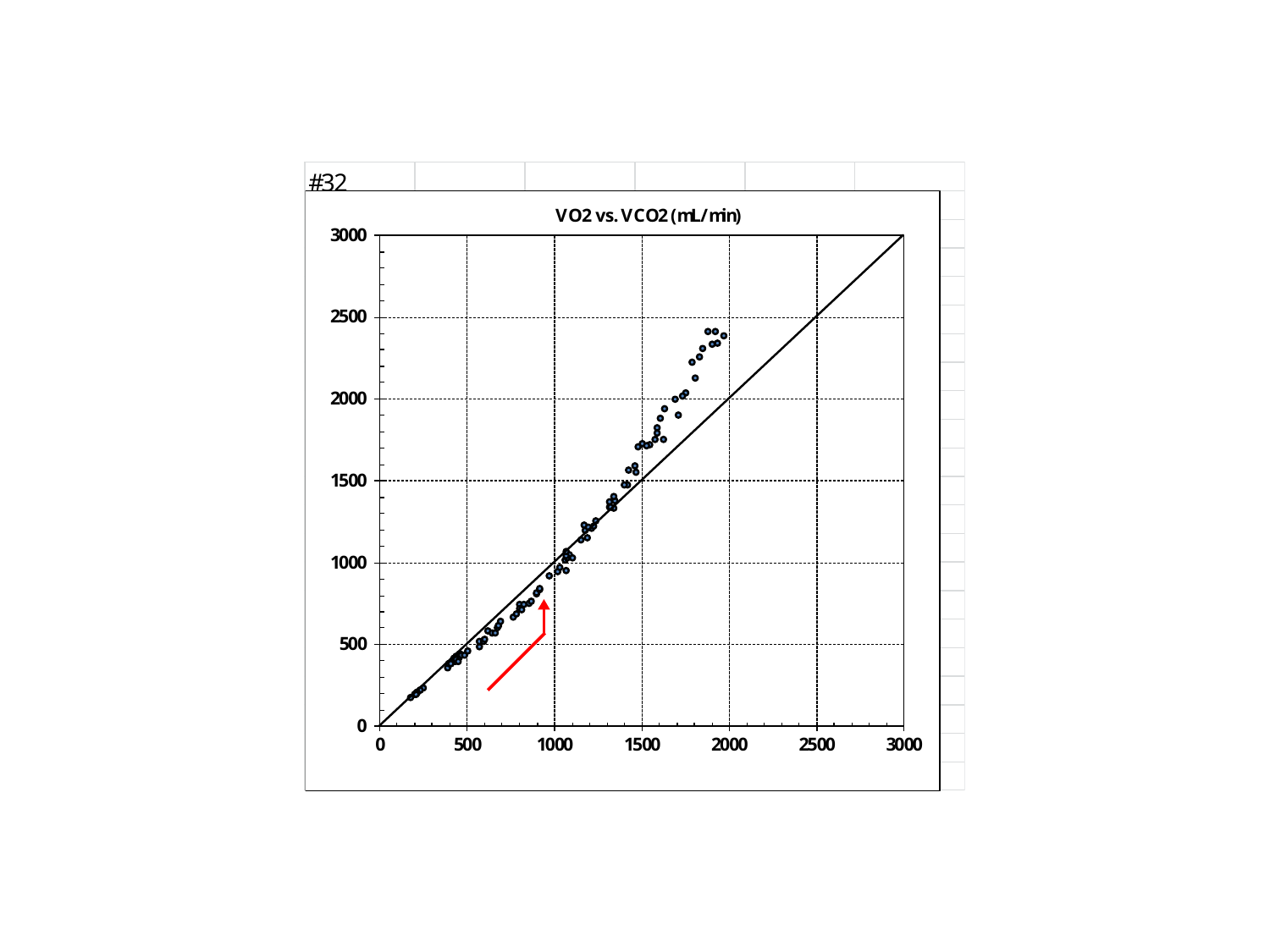

## Slide 35
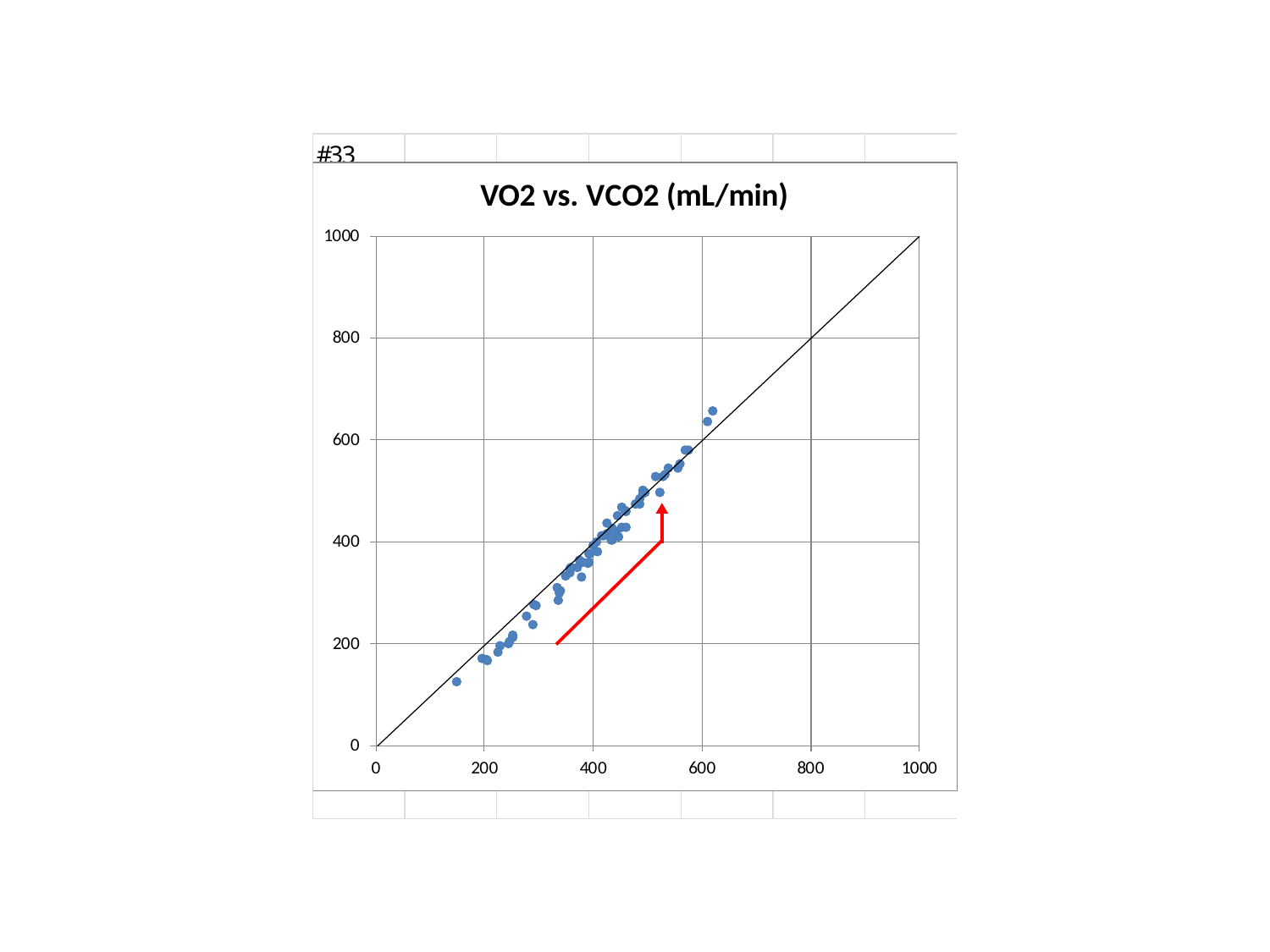

## Slide 36
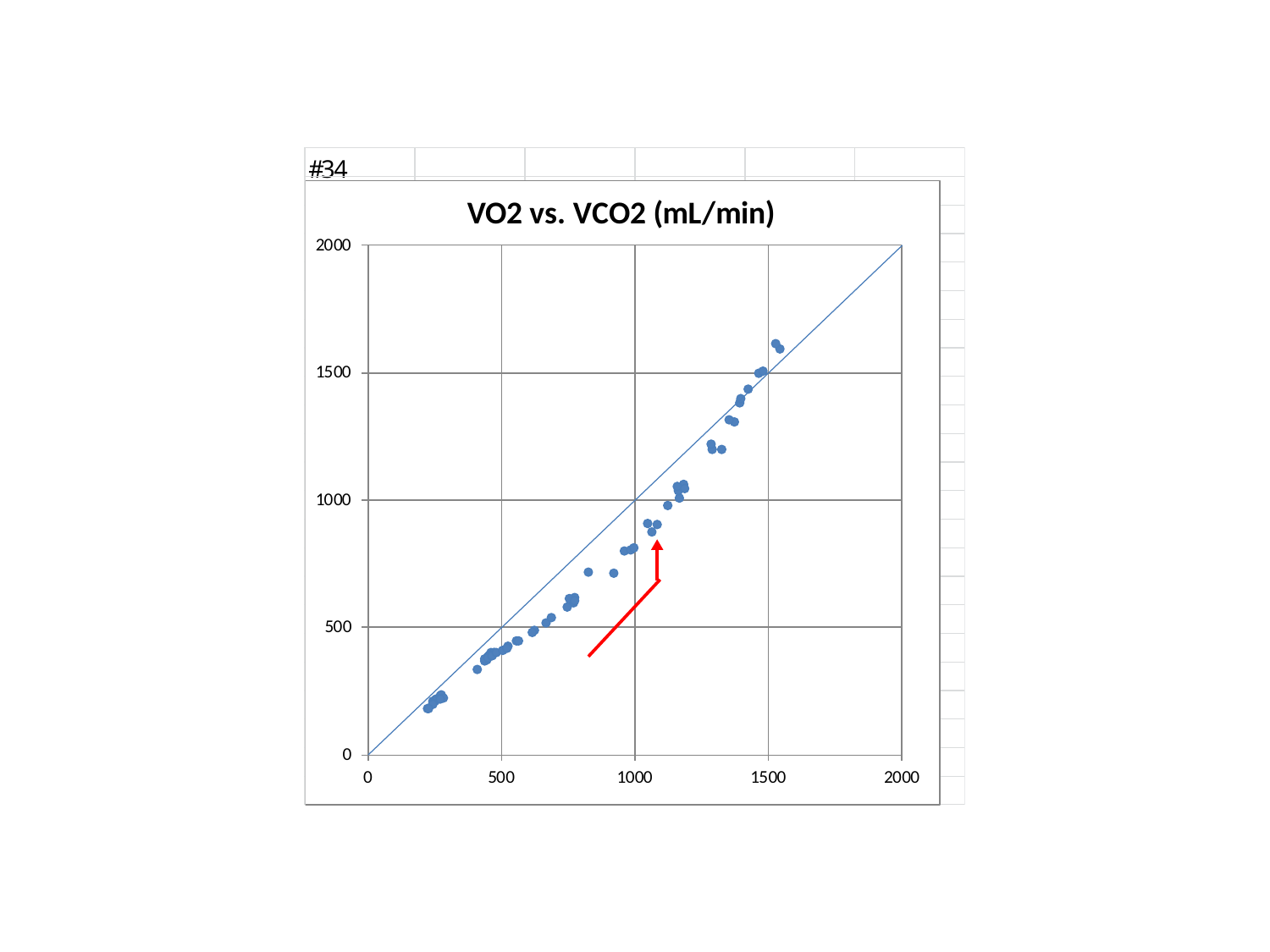

## Slide 37
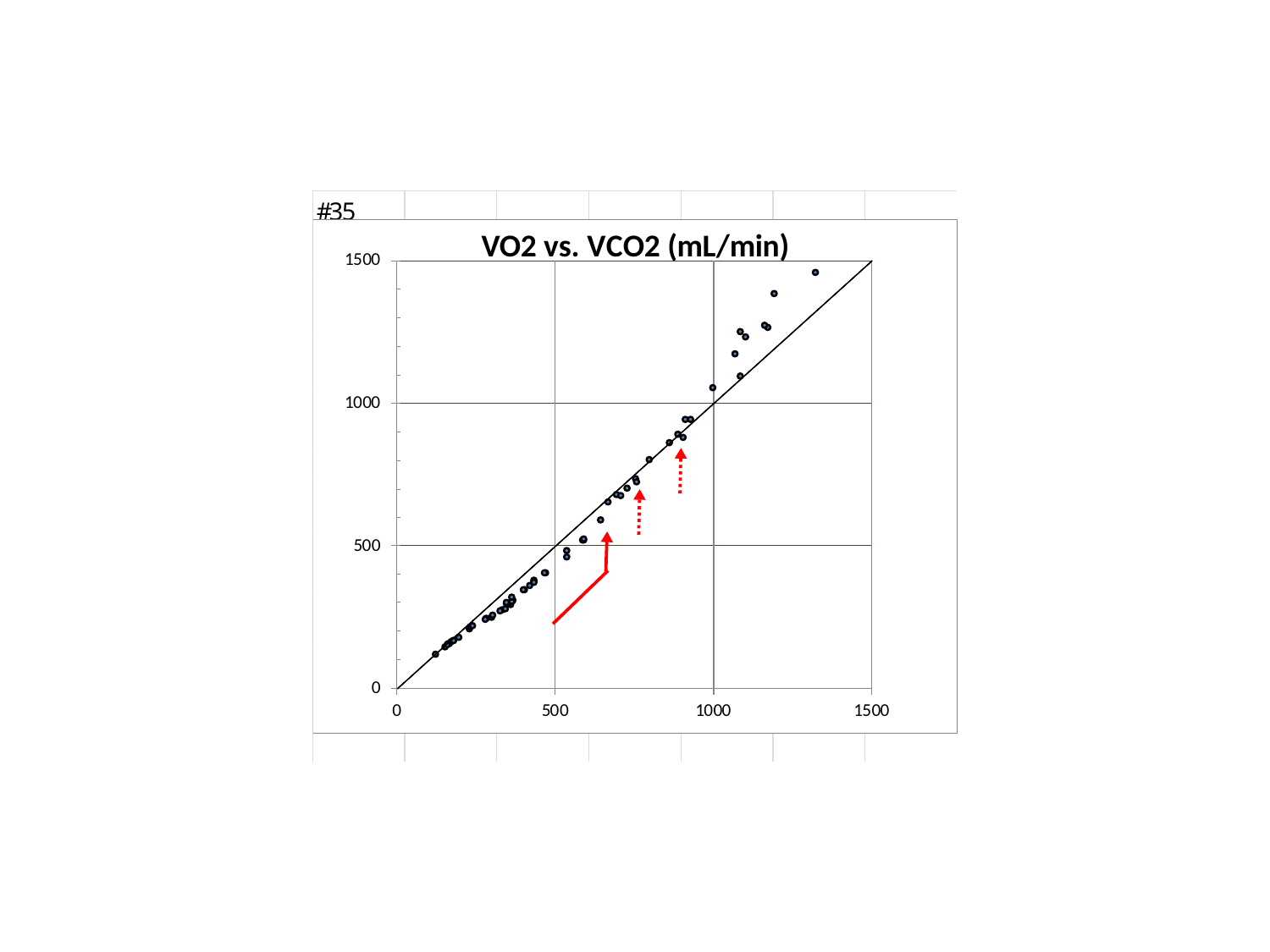

## Slide 38
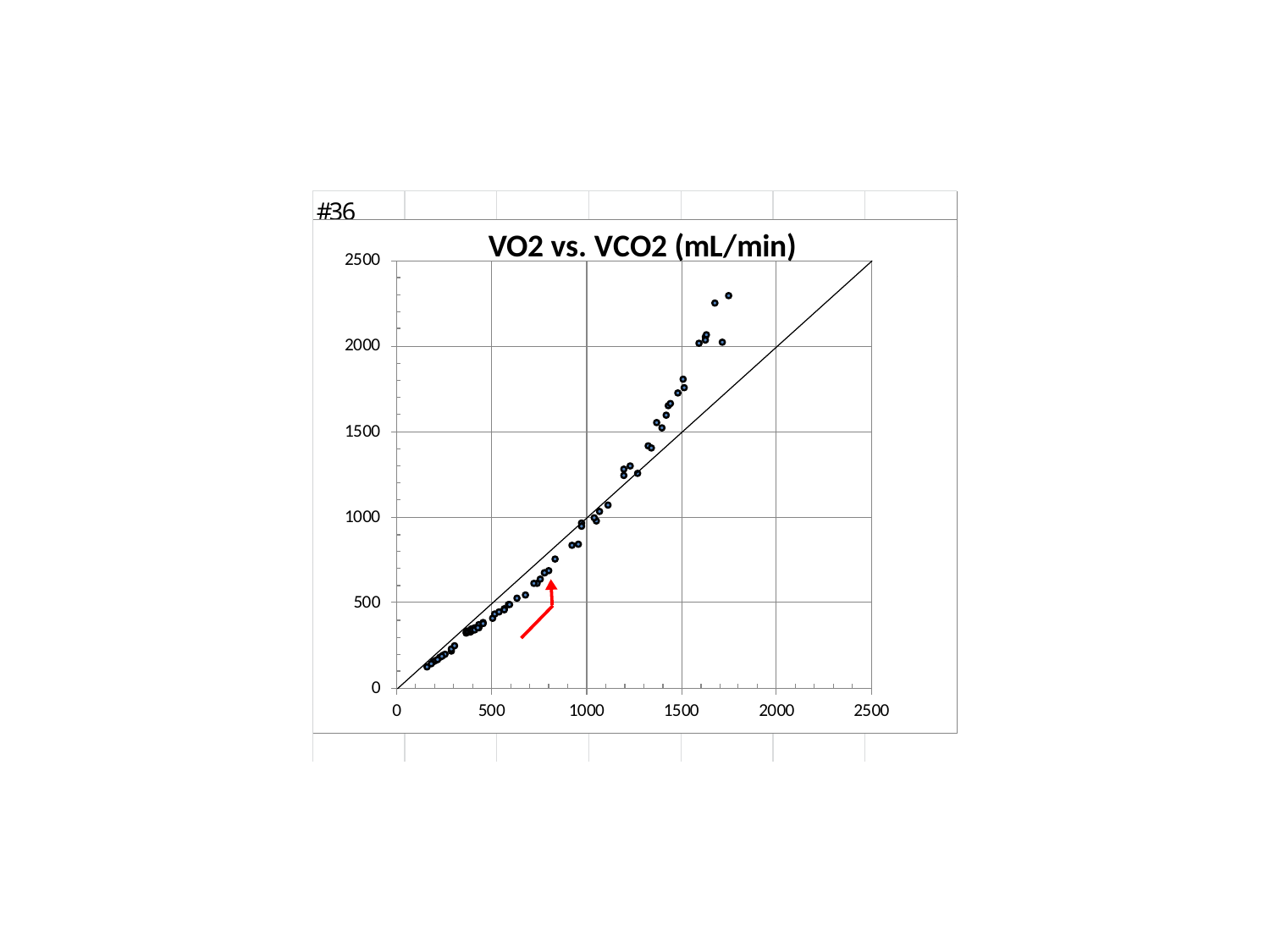

## Slide 39
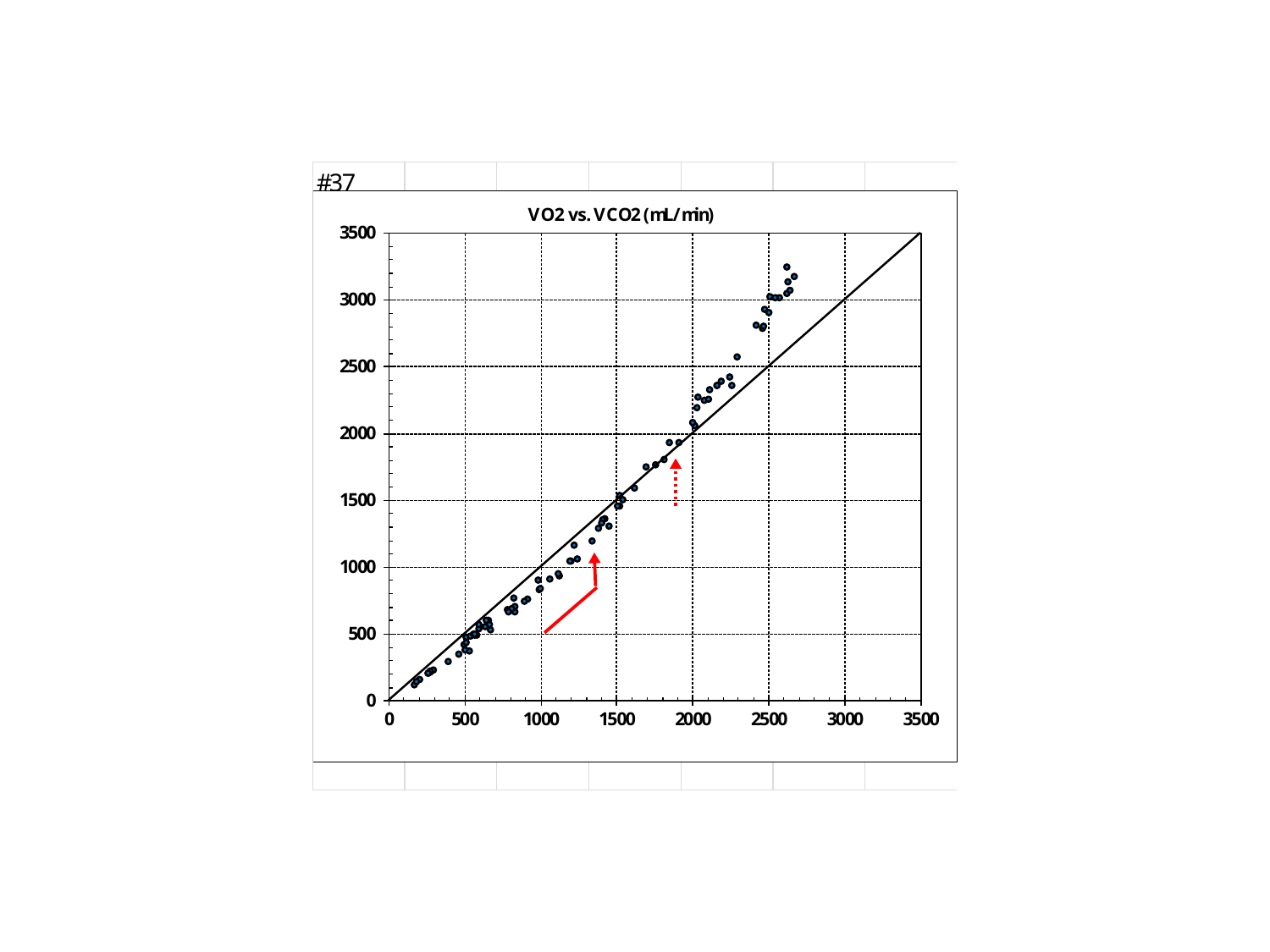

## Slide 40
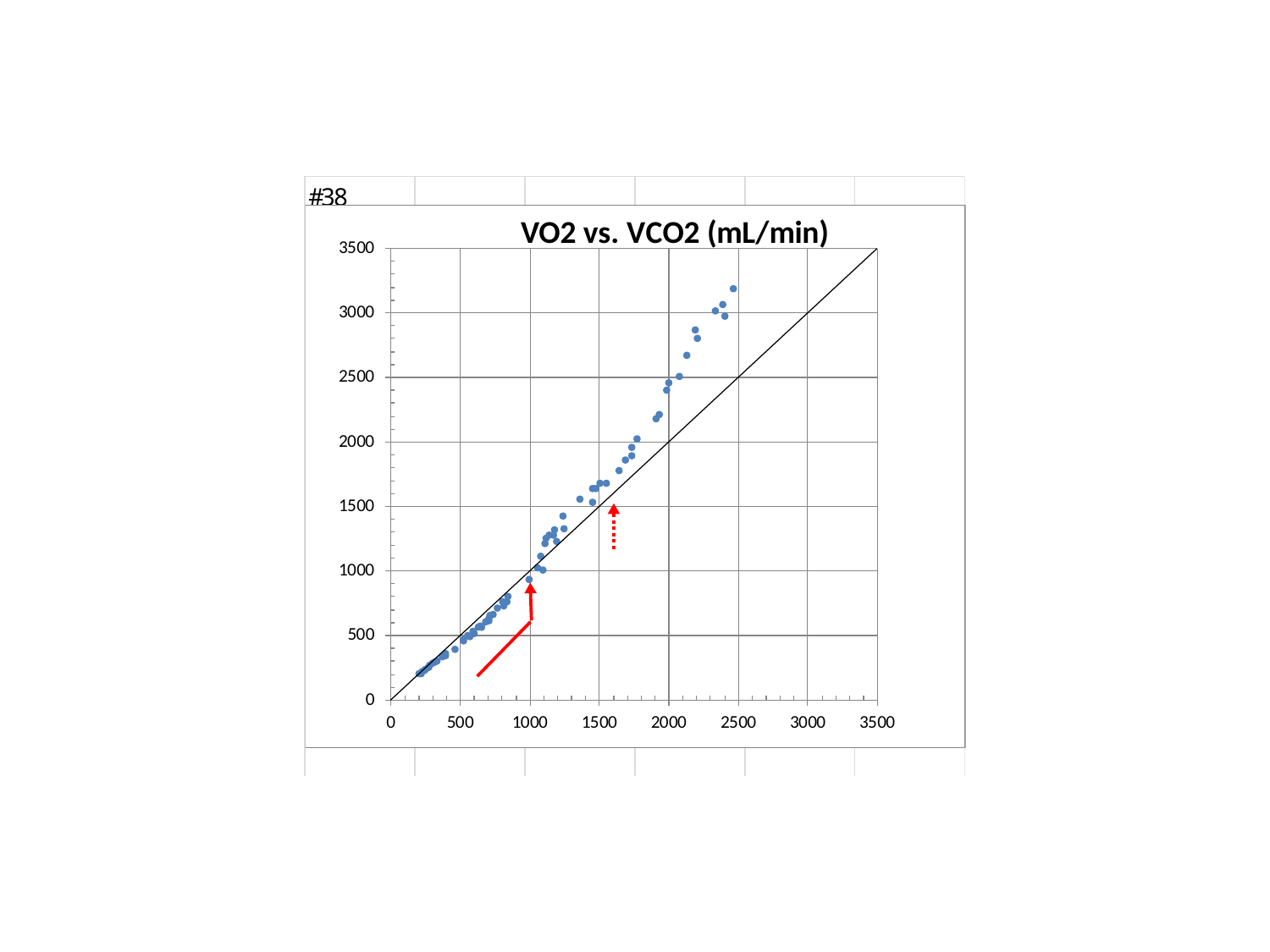

## Slide 41
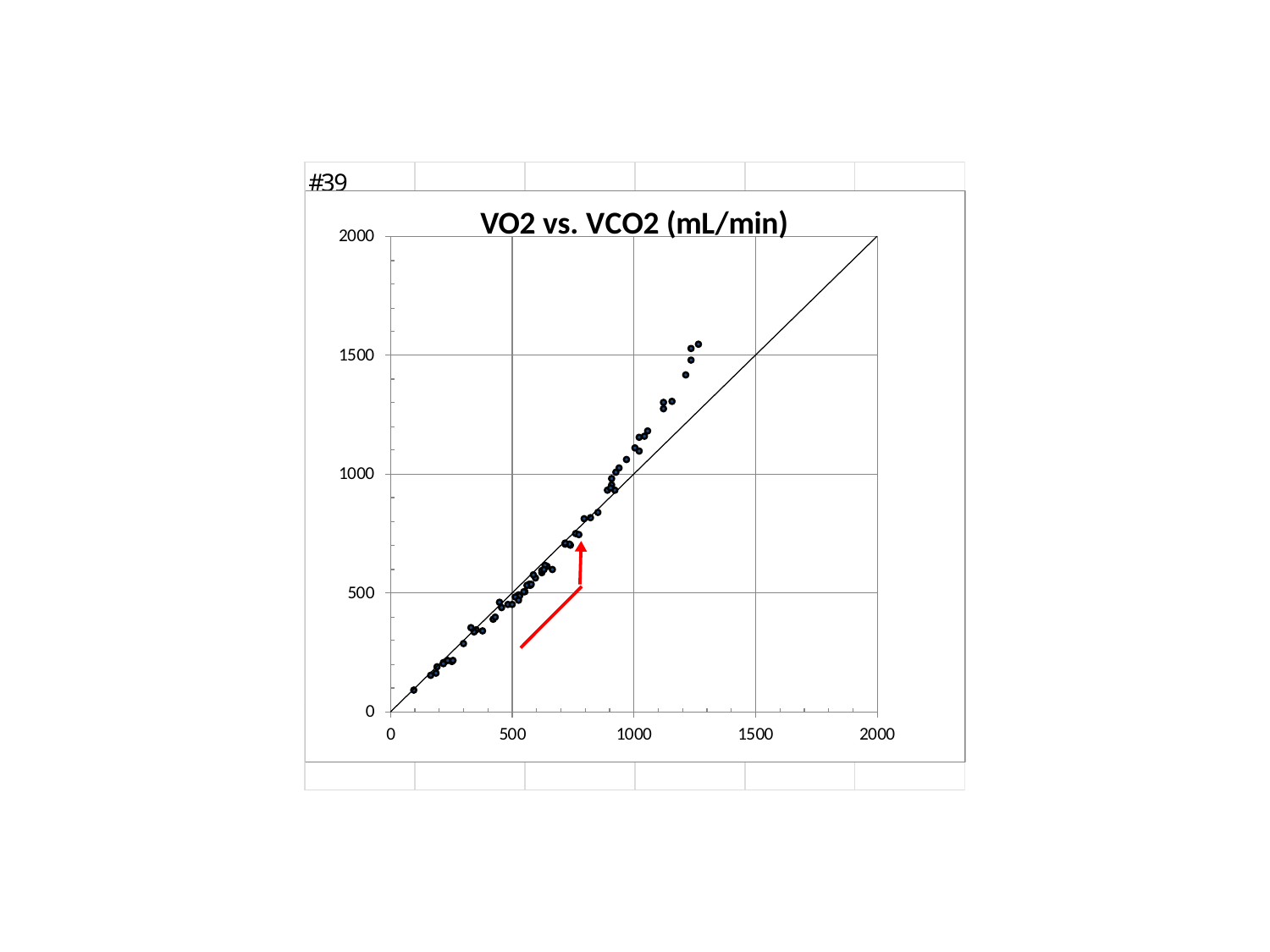

## Slide 42
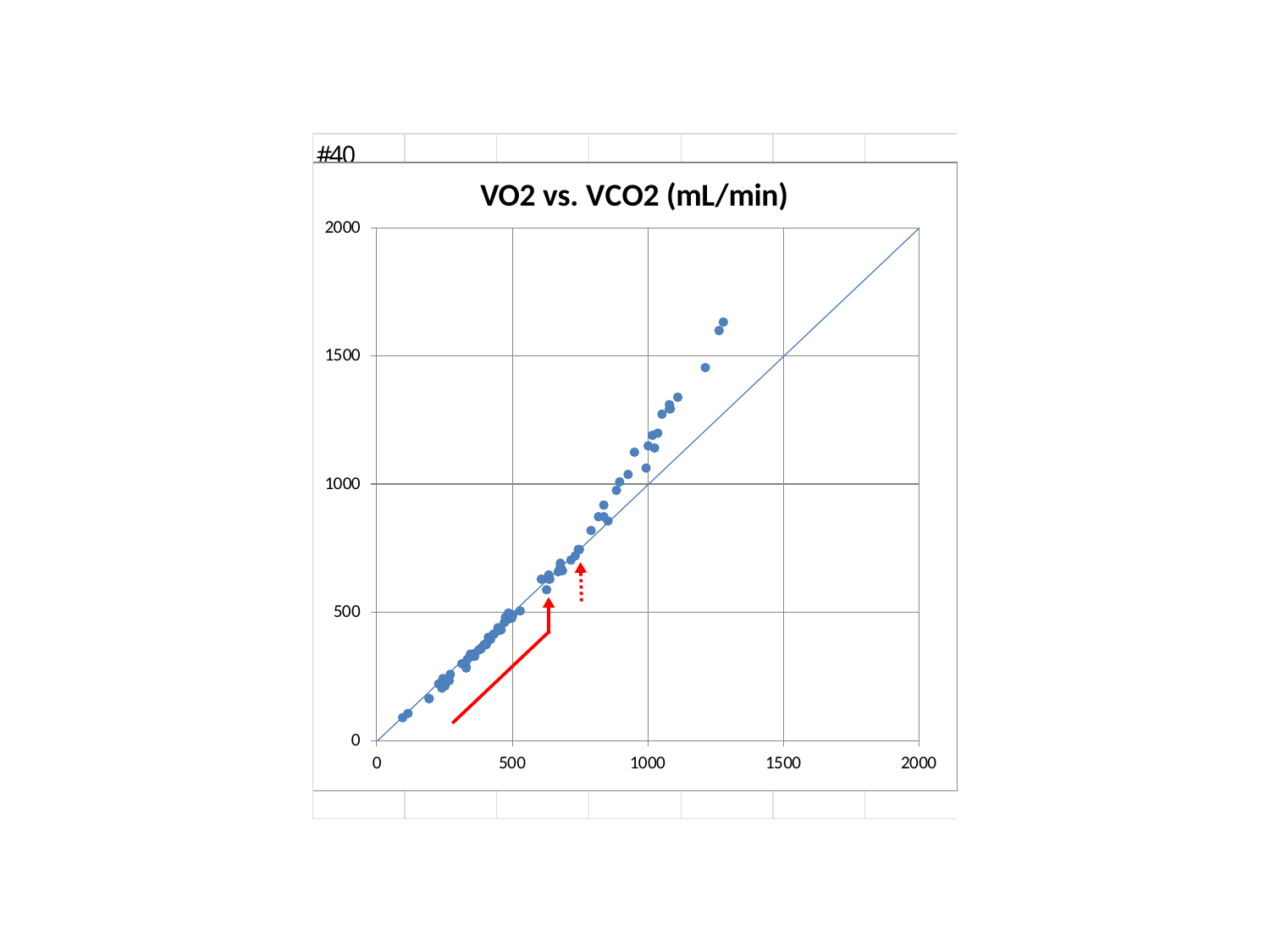

## Slide 43
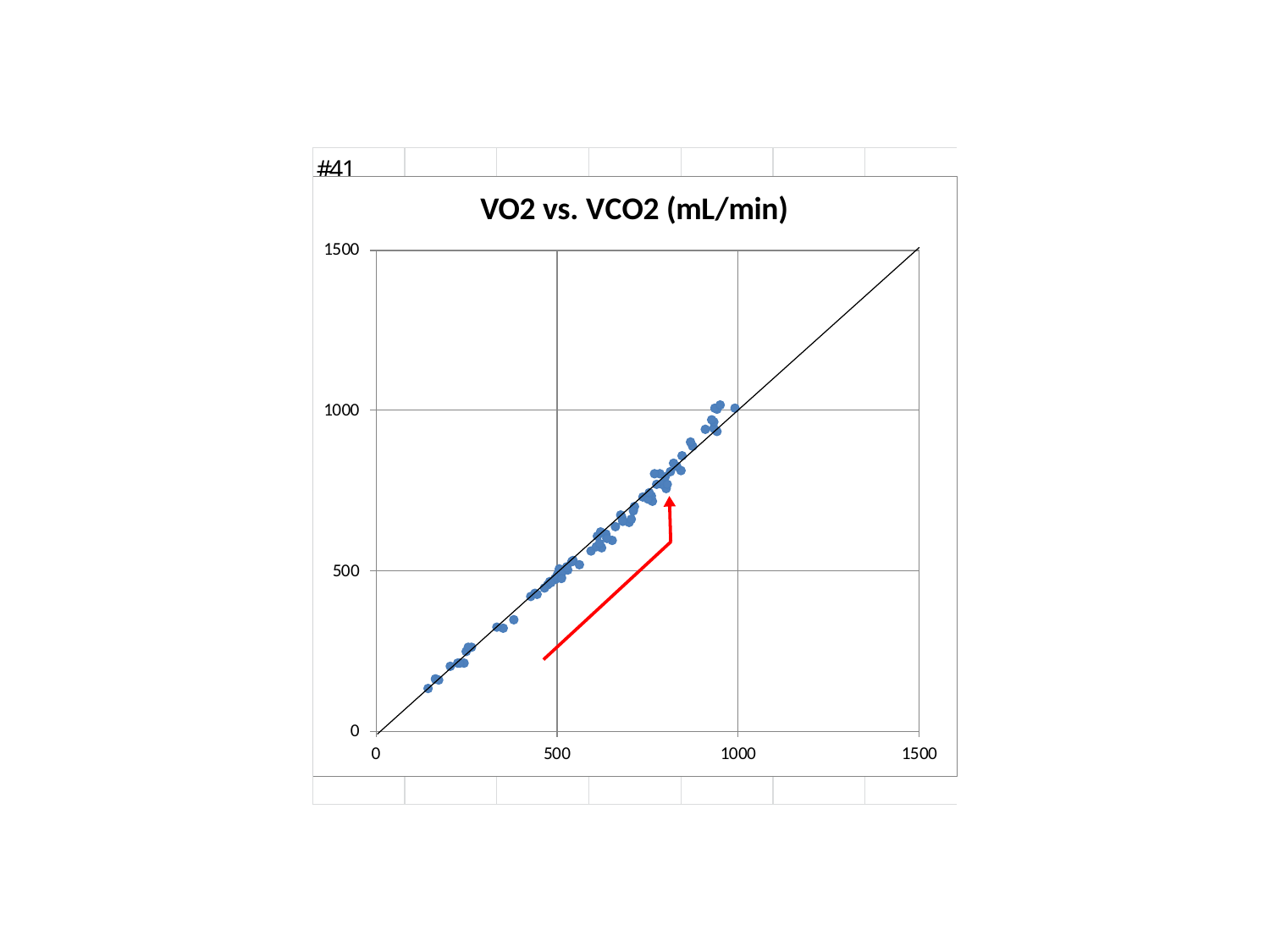

## Slide 44
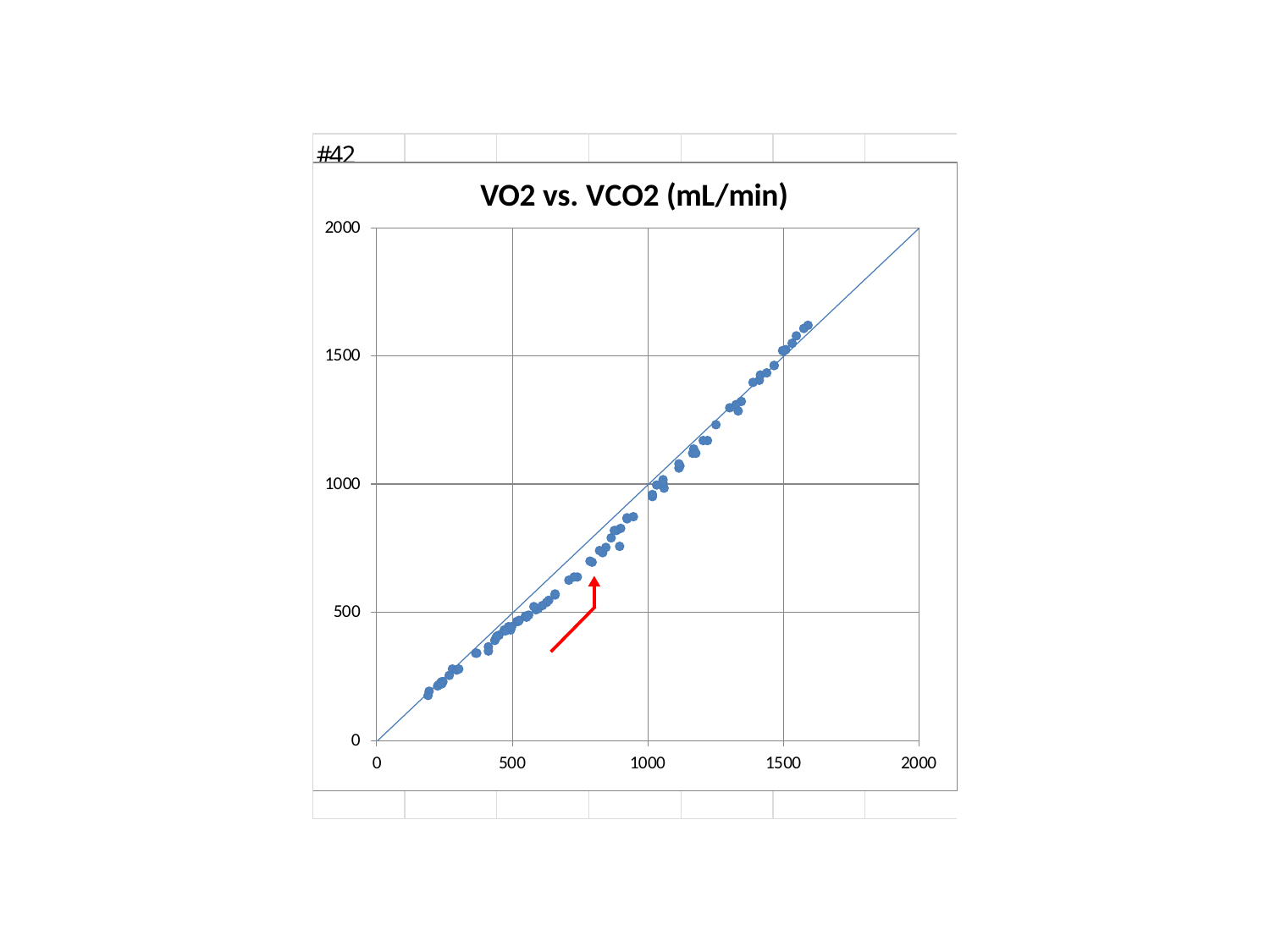

## Slide 45
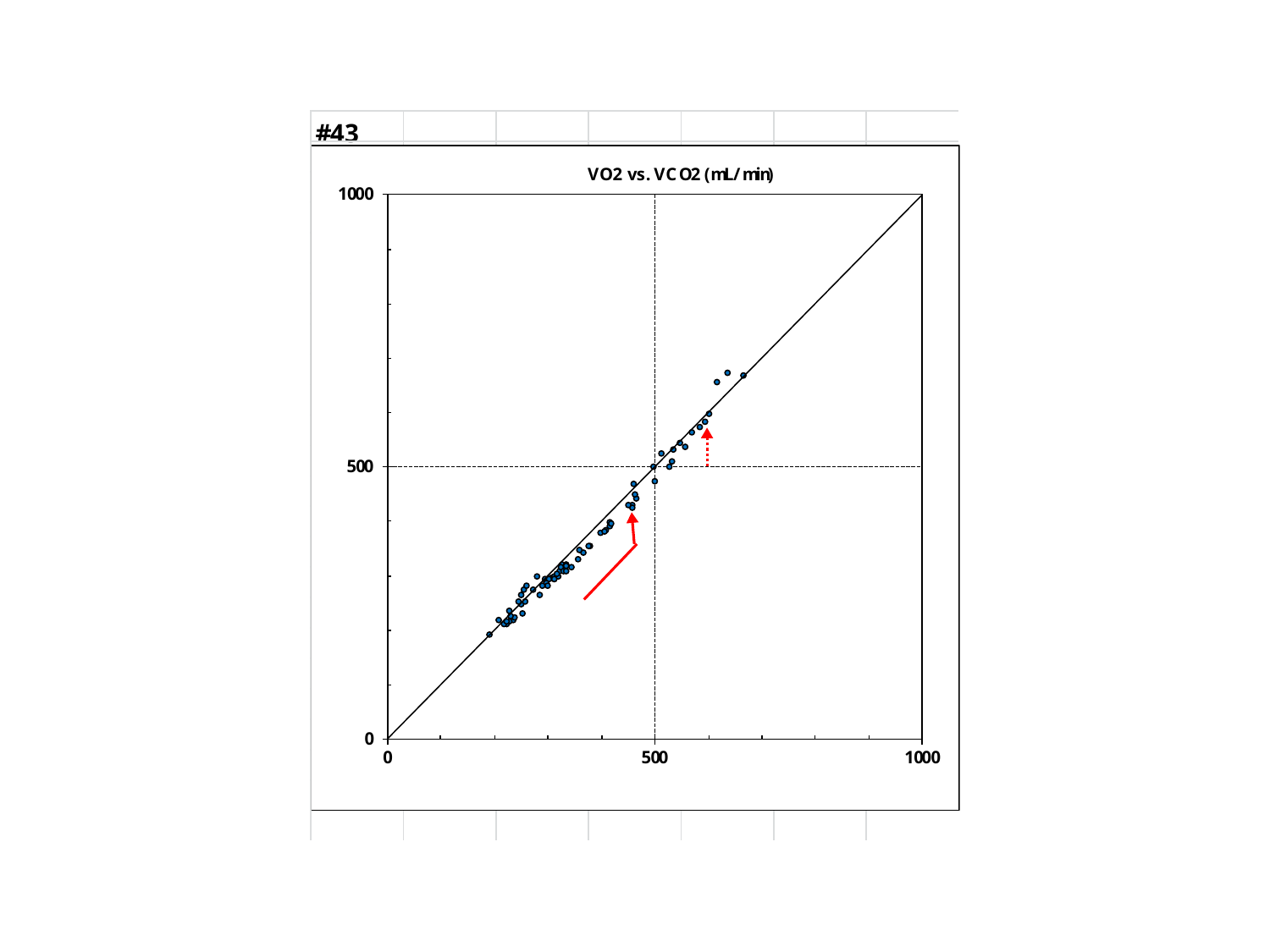

## Slide 46
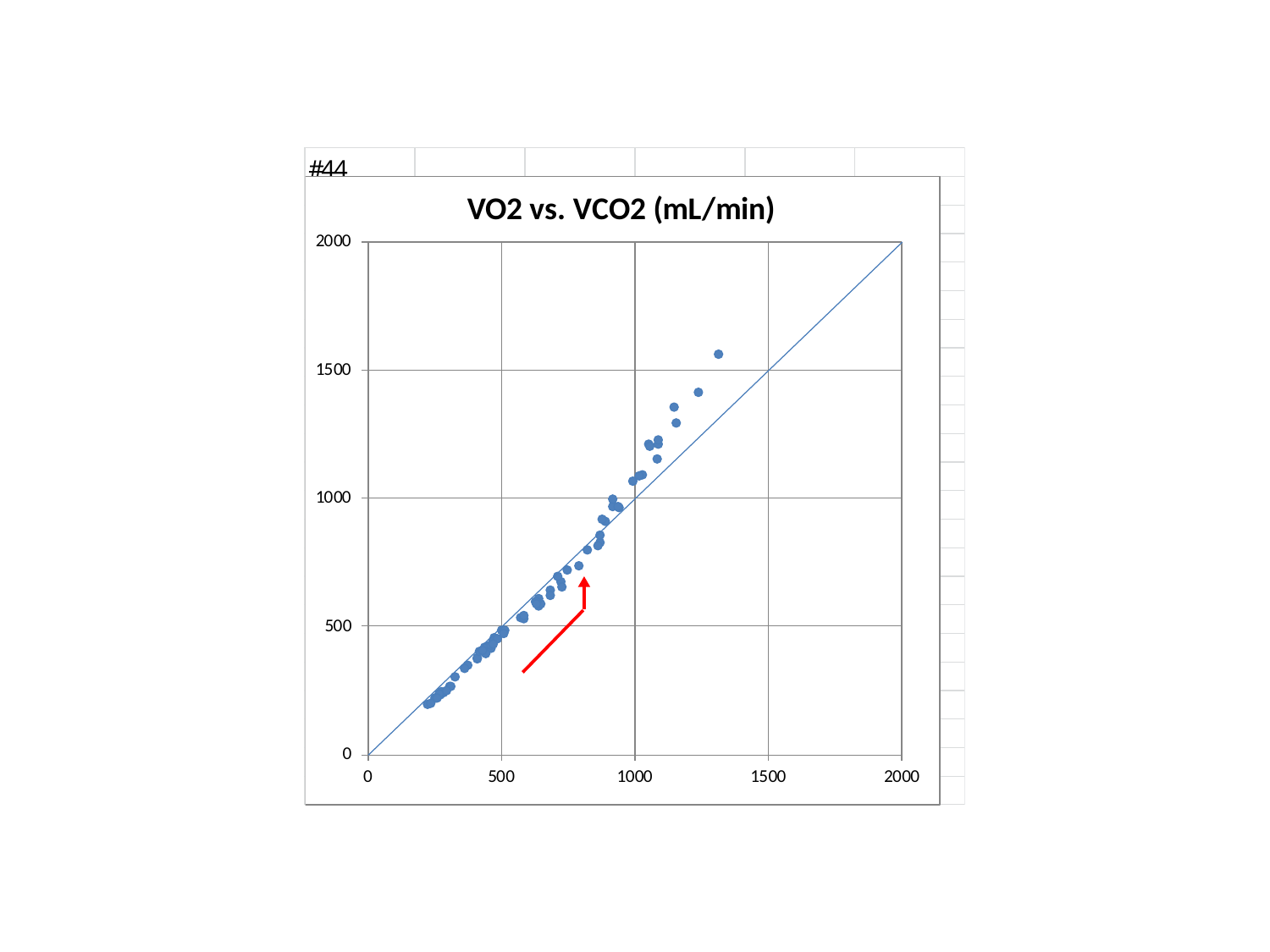

## Slide 47
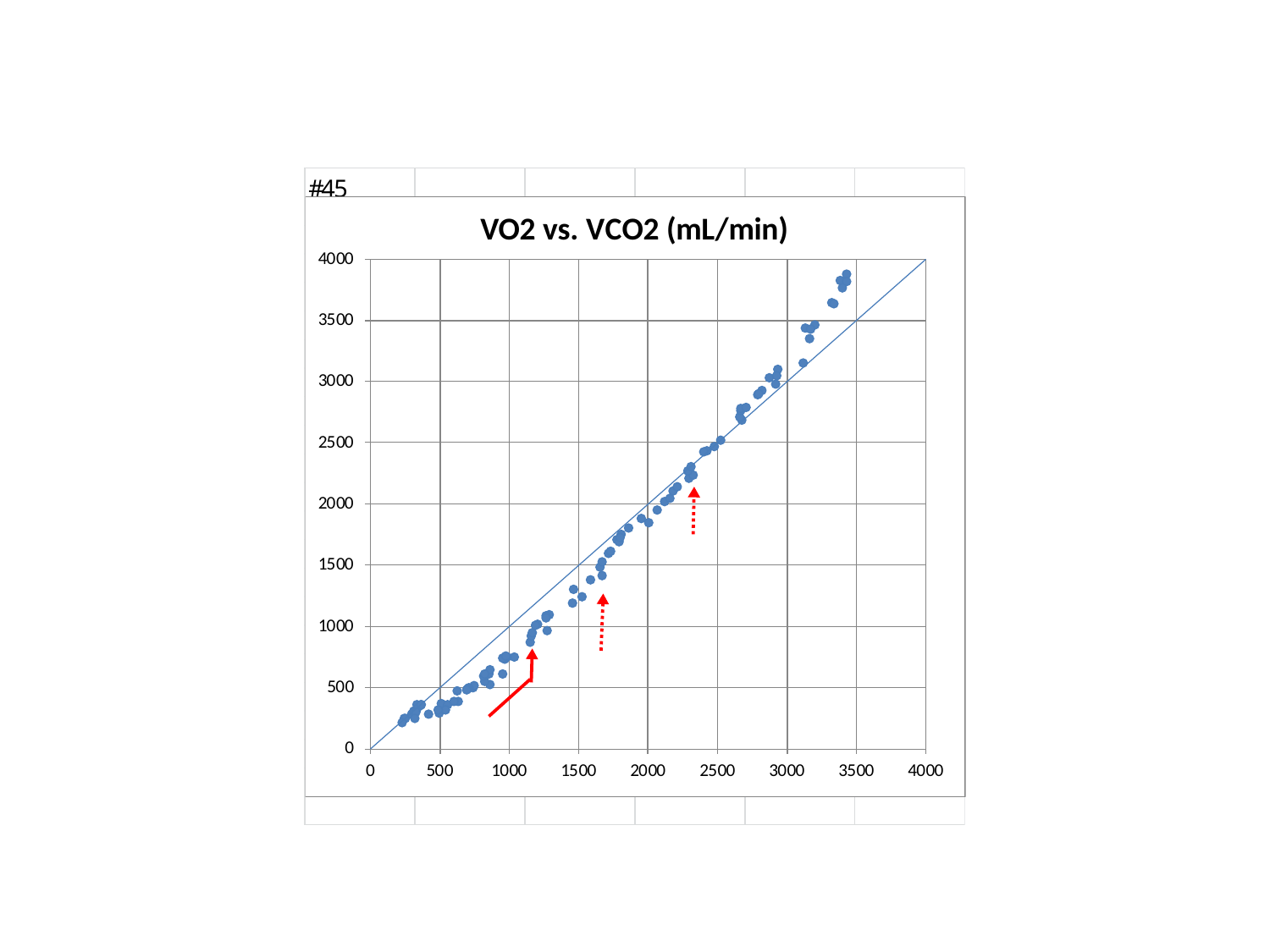

## Slide 48
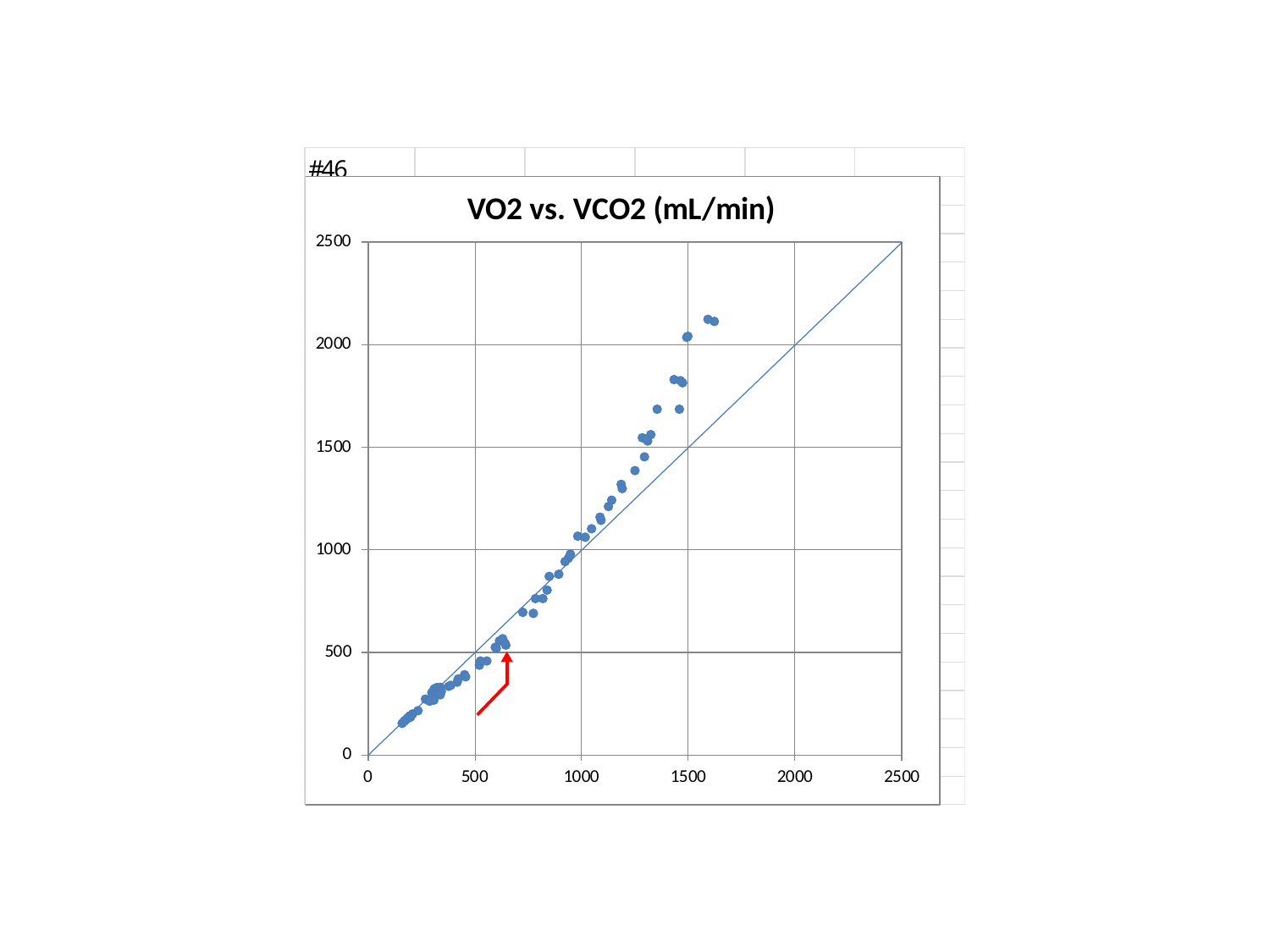

## Slide 49
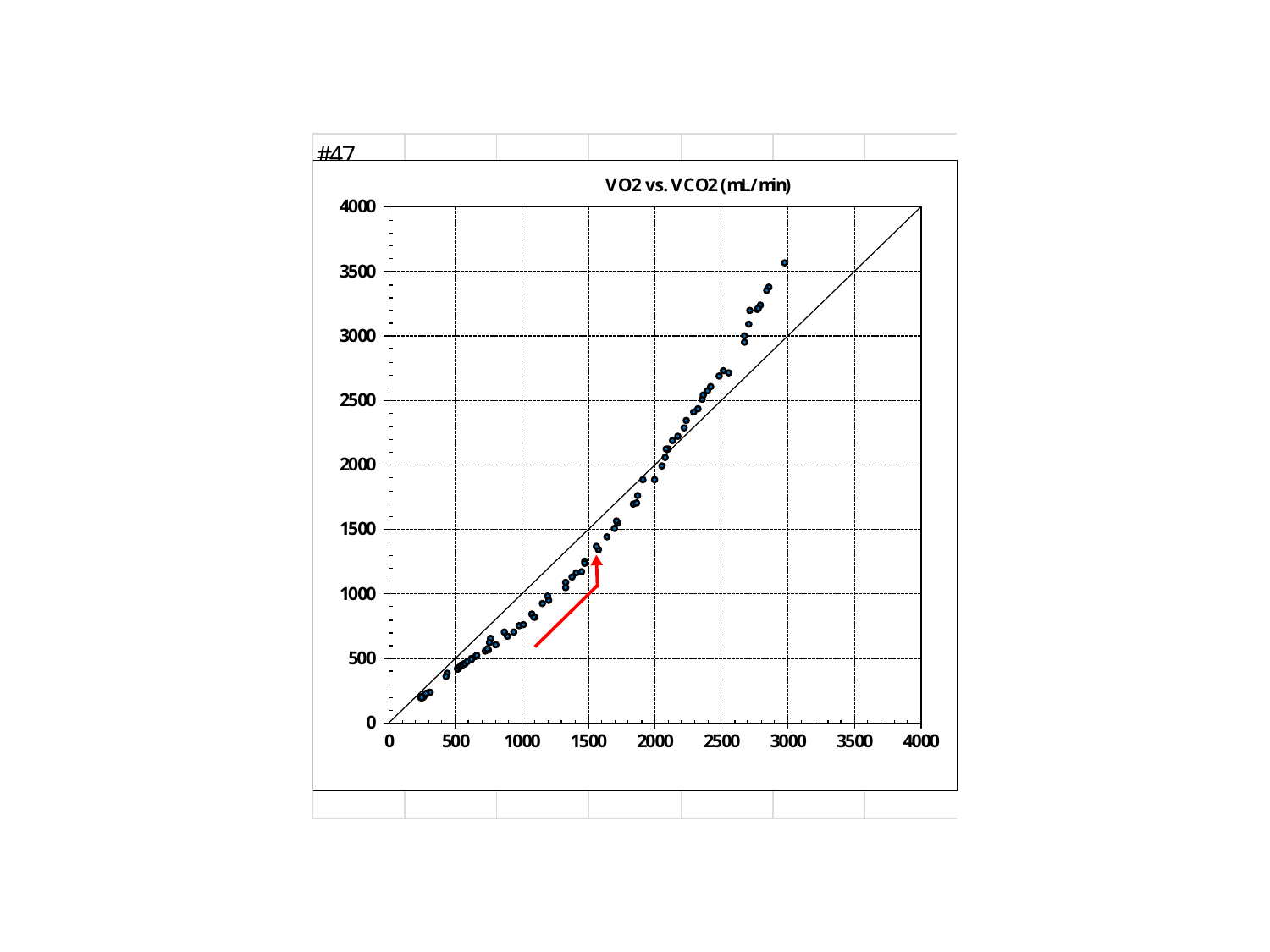

## Slide 50
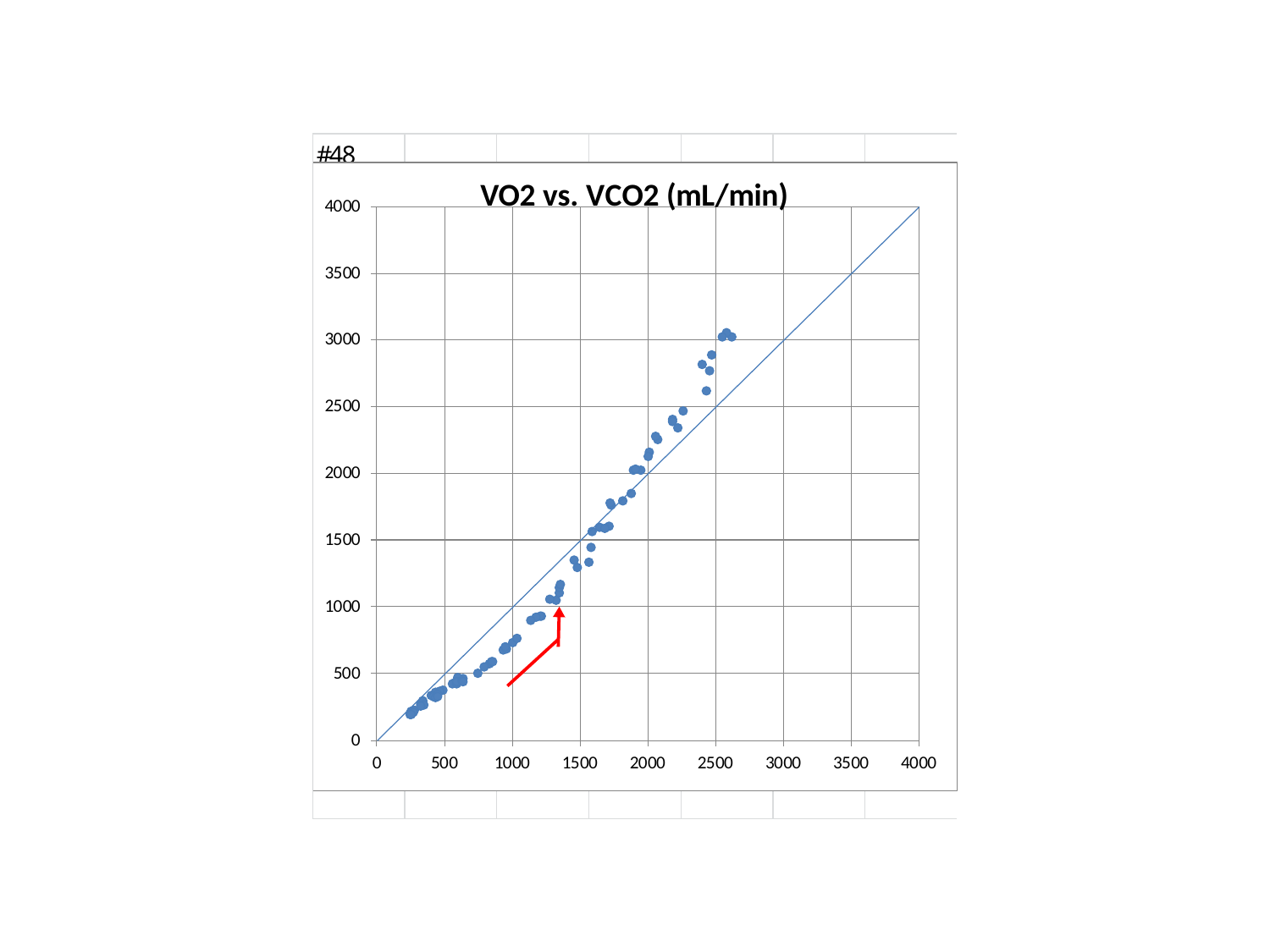

## Slide 51
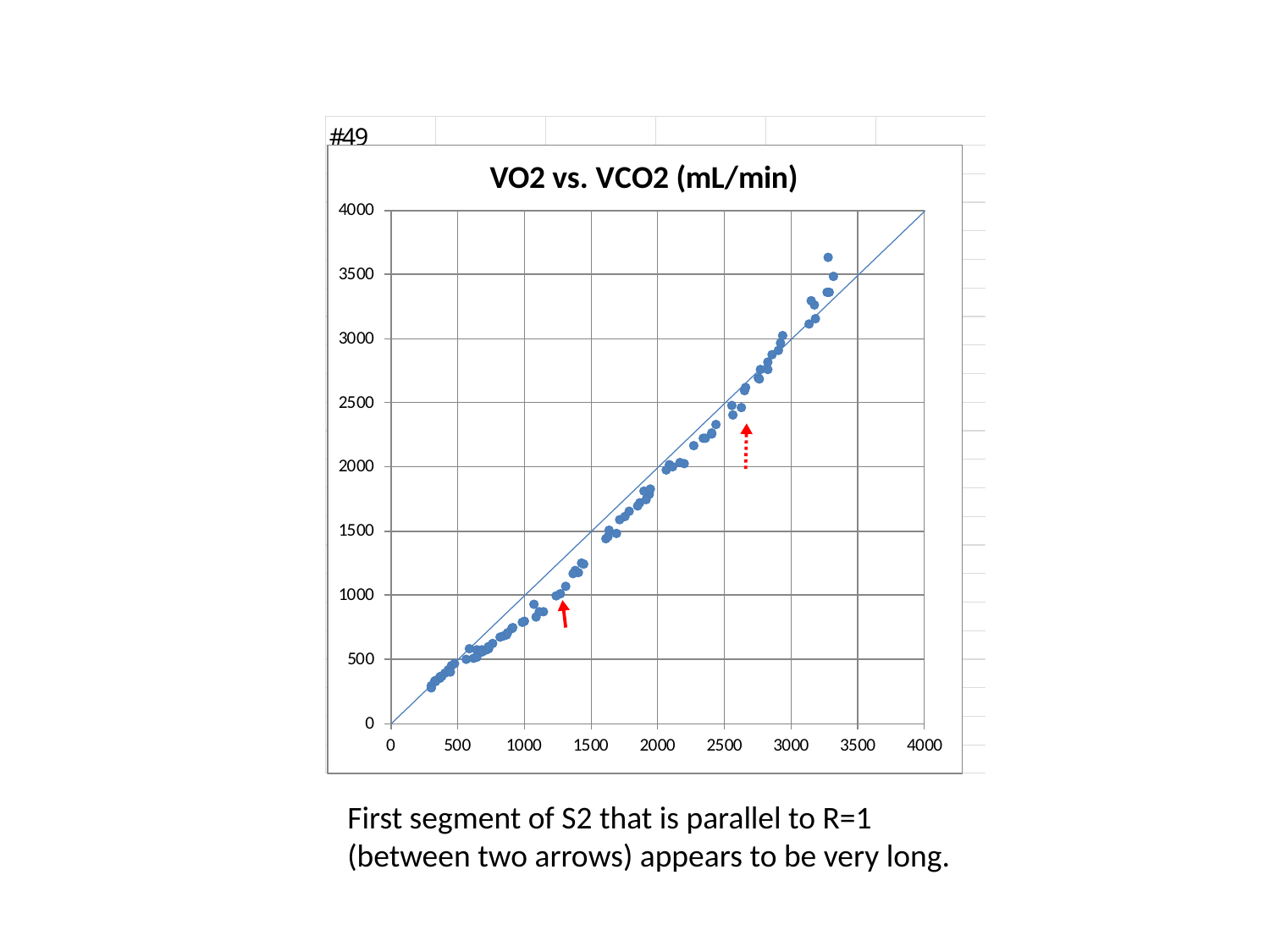

First segment of S2 that is parallel to R=1 (between two arrows) appears to be very long.

## Slide 52
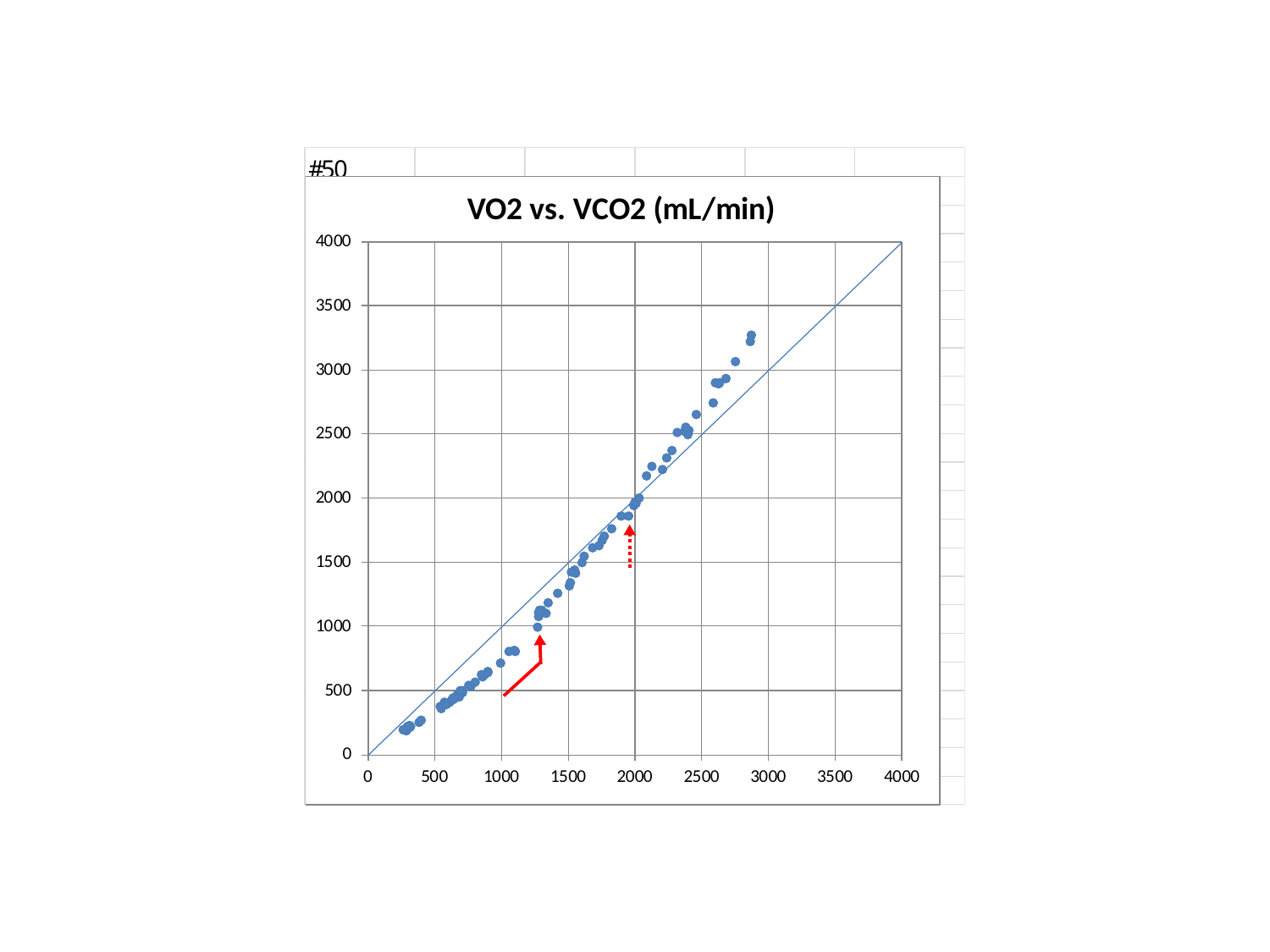

## Slide 53
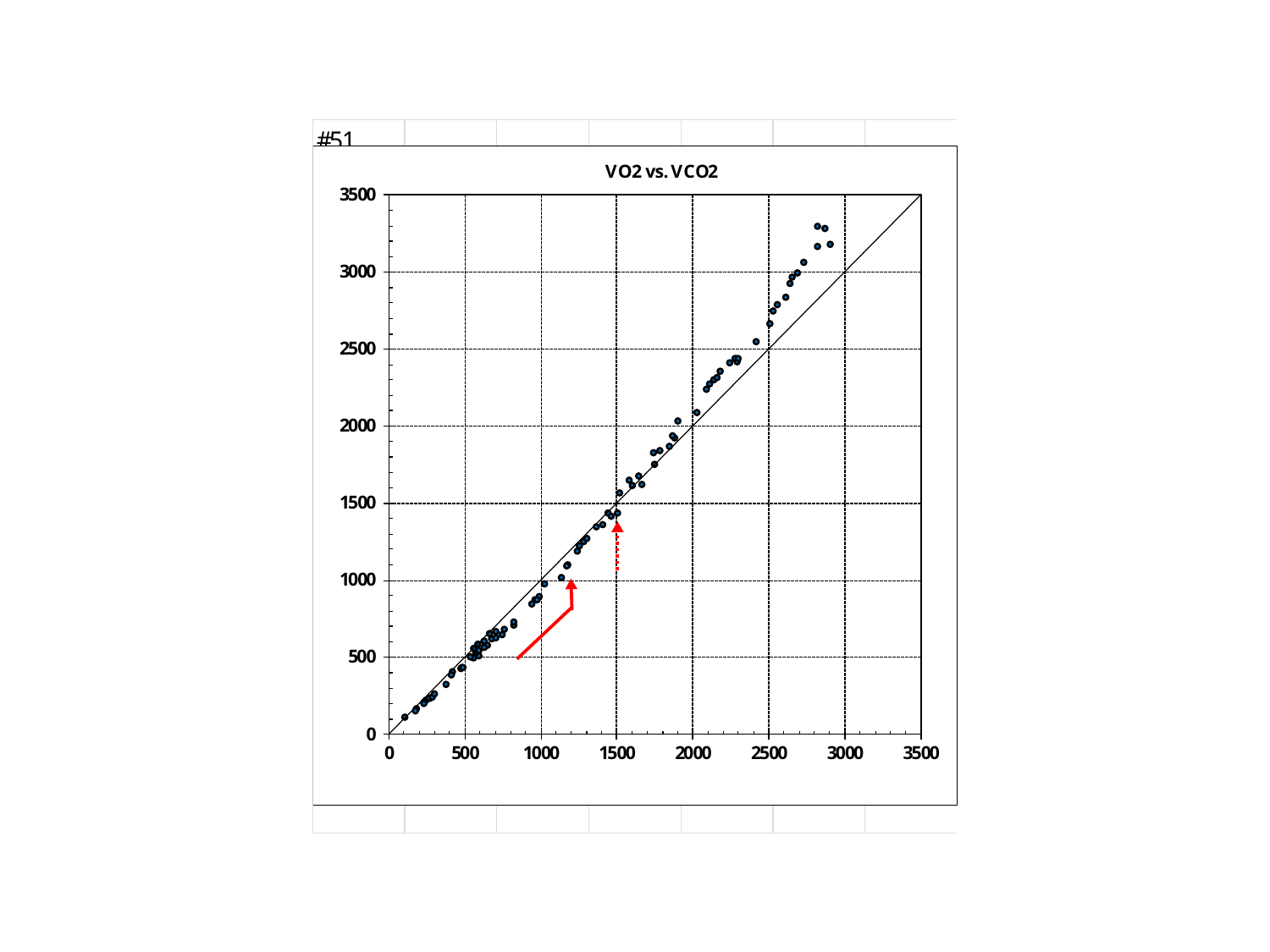

## Slide 54
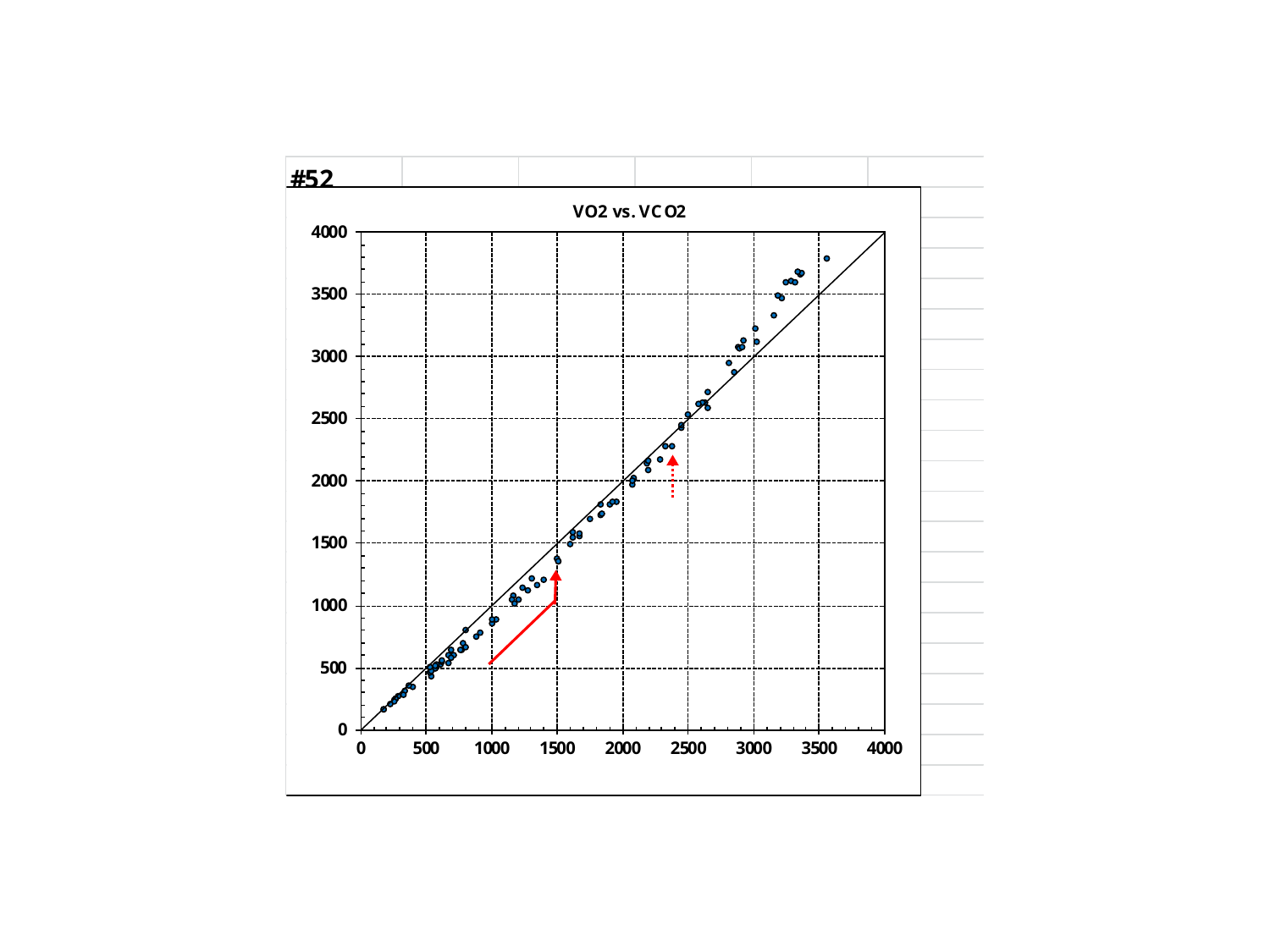

## Slide 55
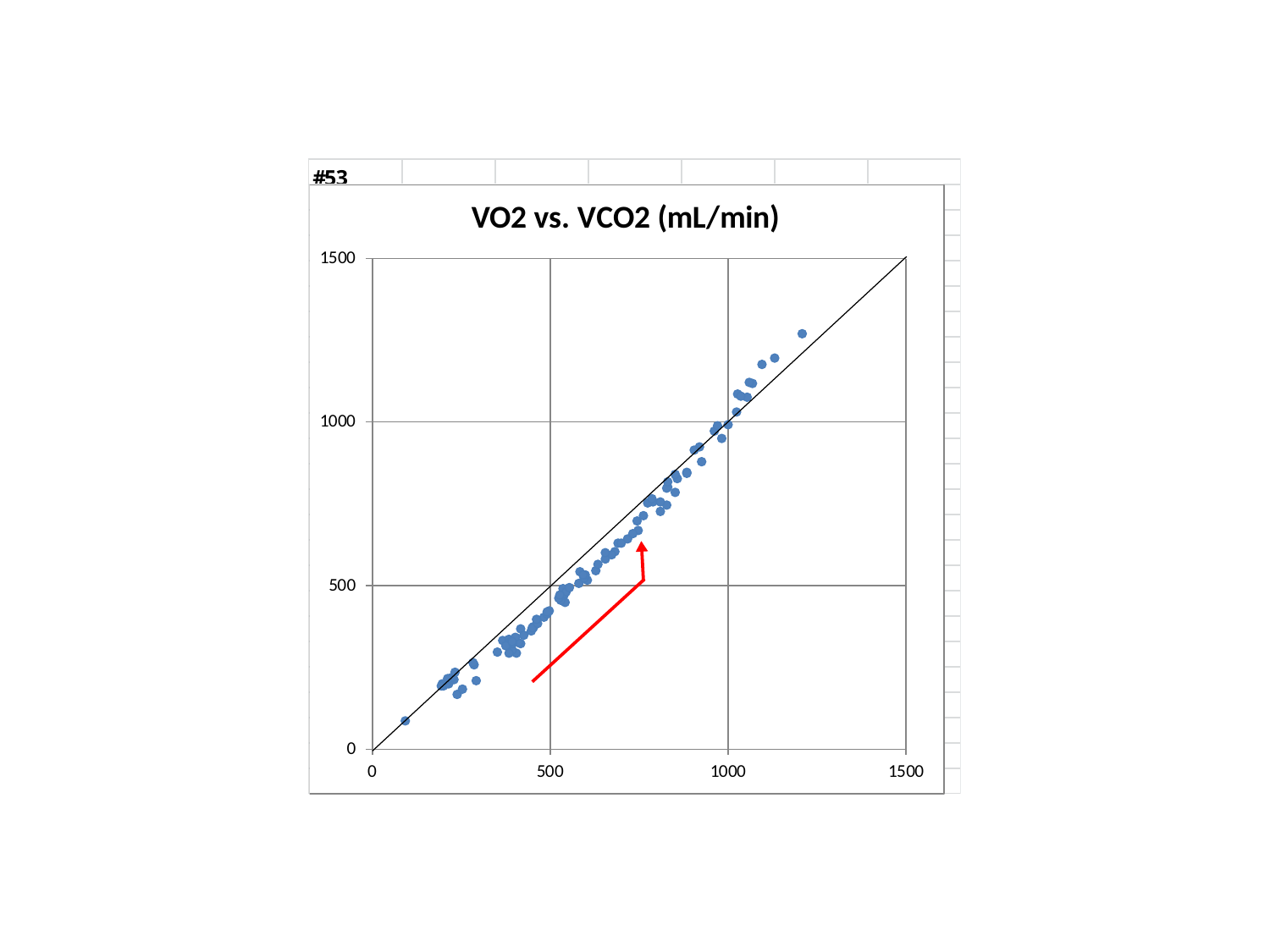

## Slide 56
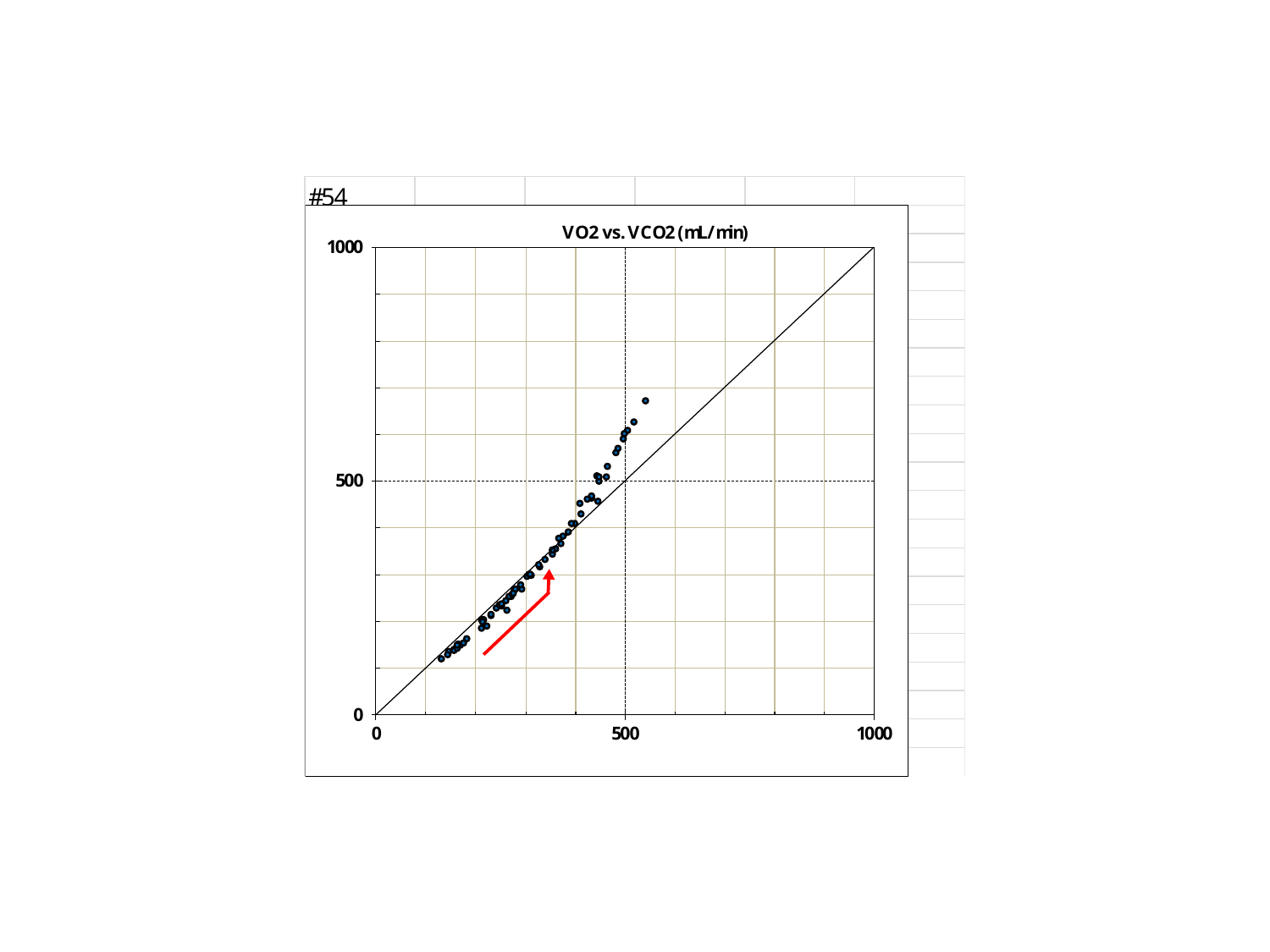

## Slide 57
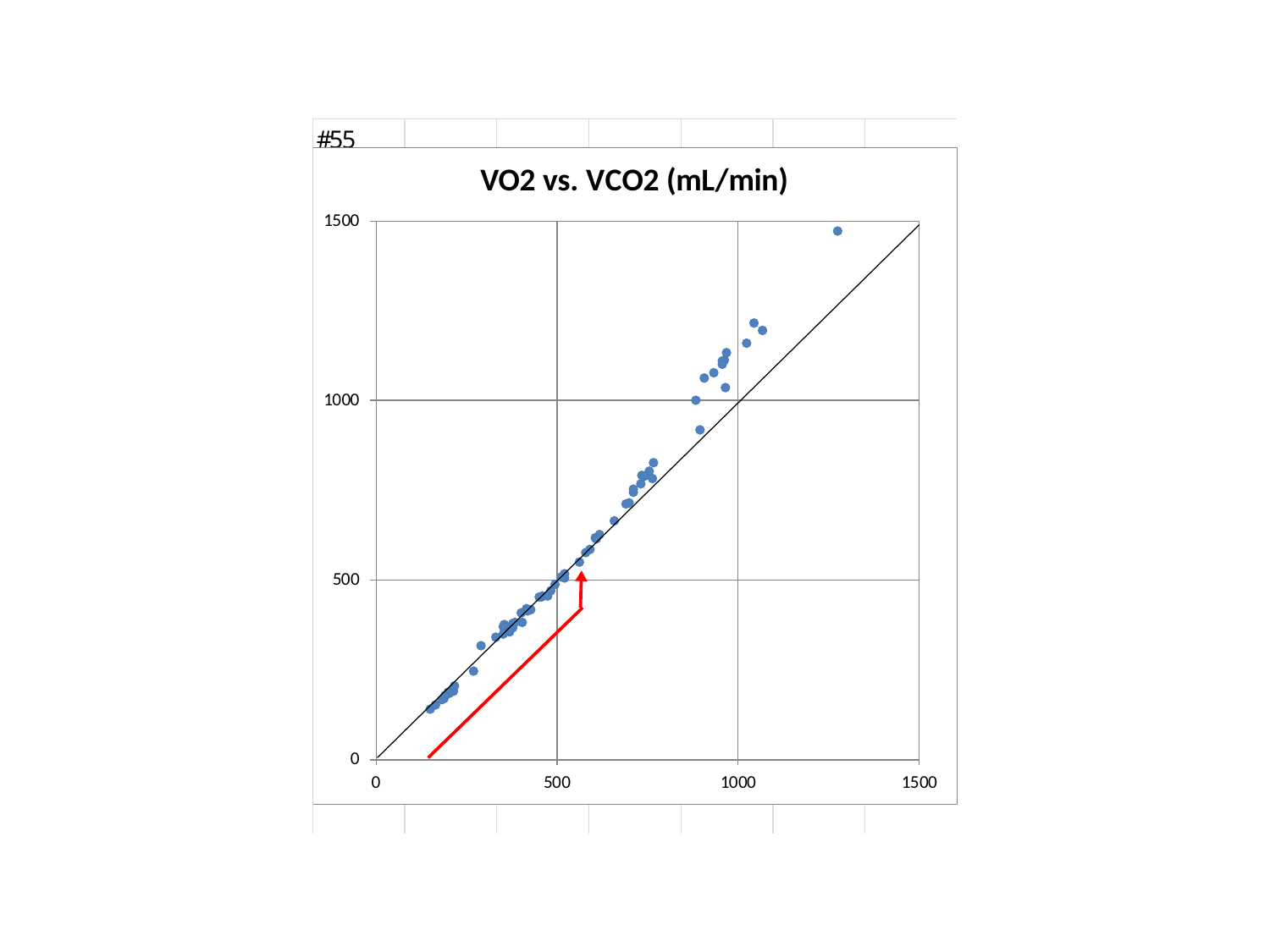

## Slide 58
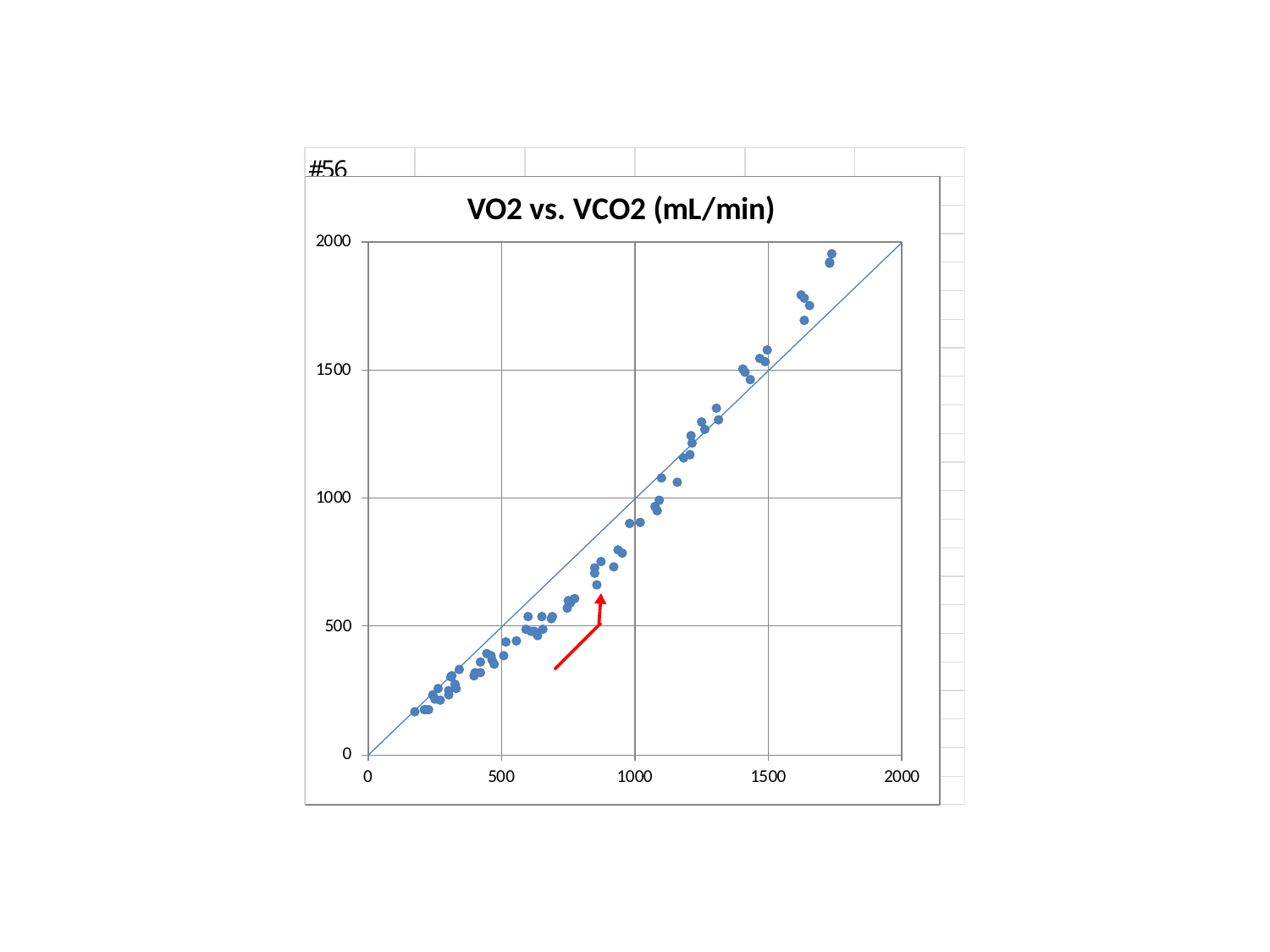

## Slide 59
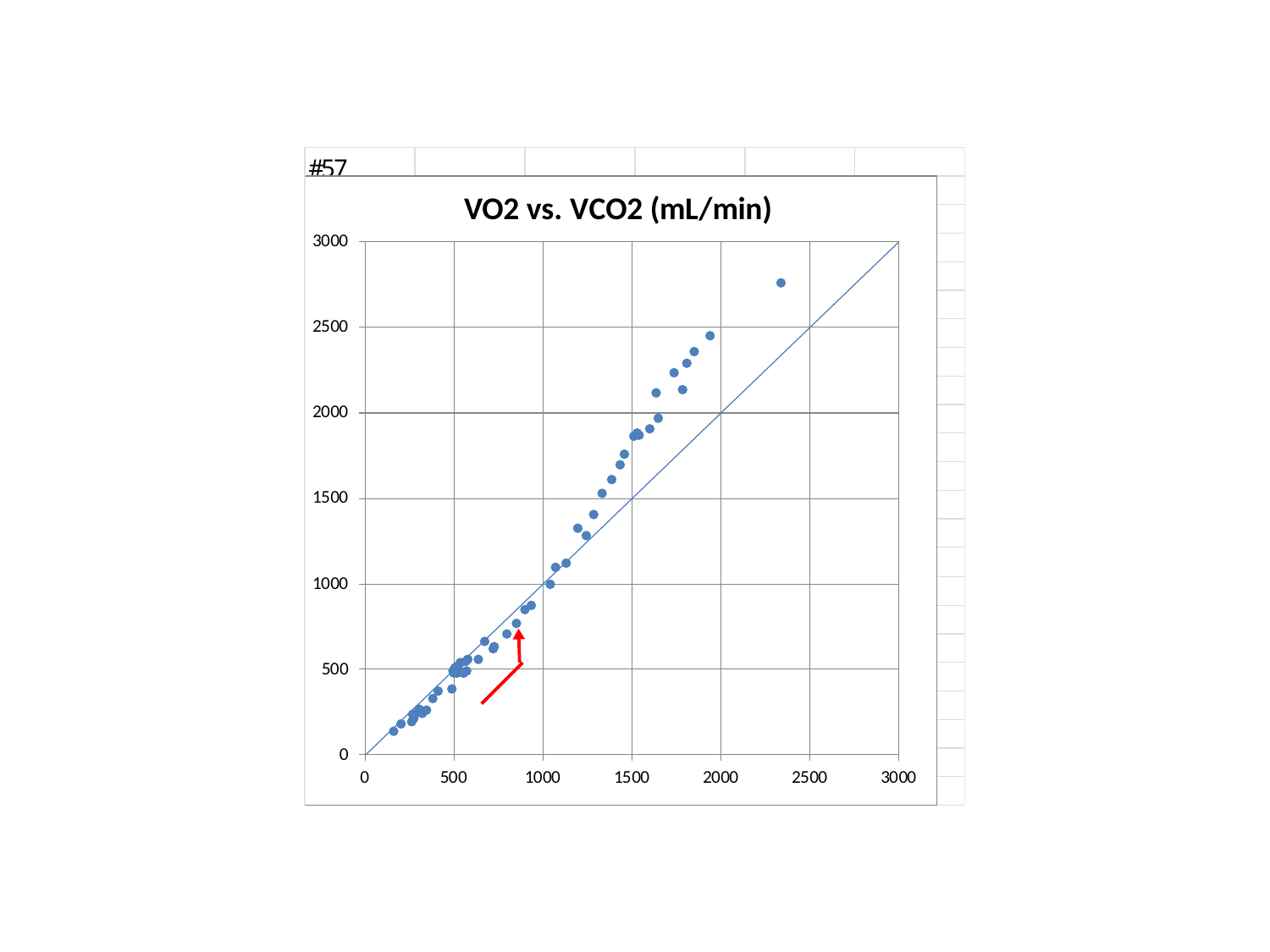

## Slide 60
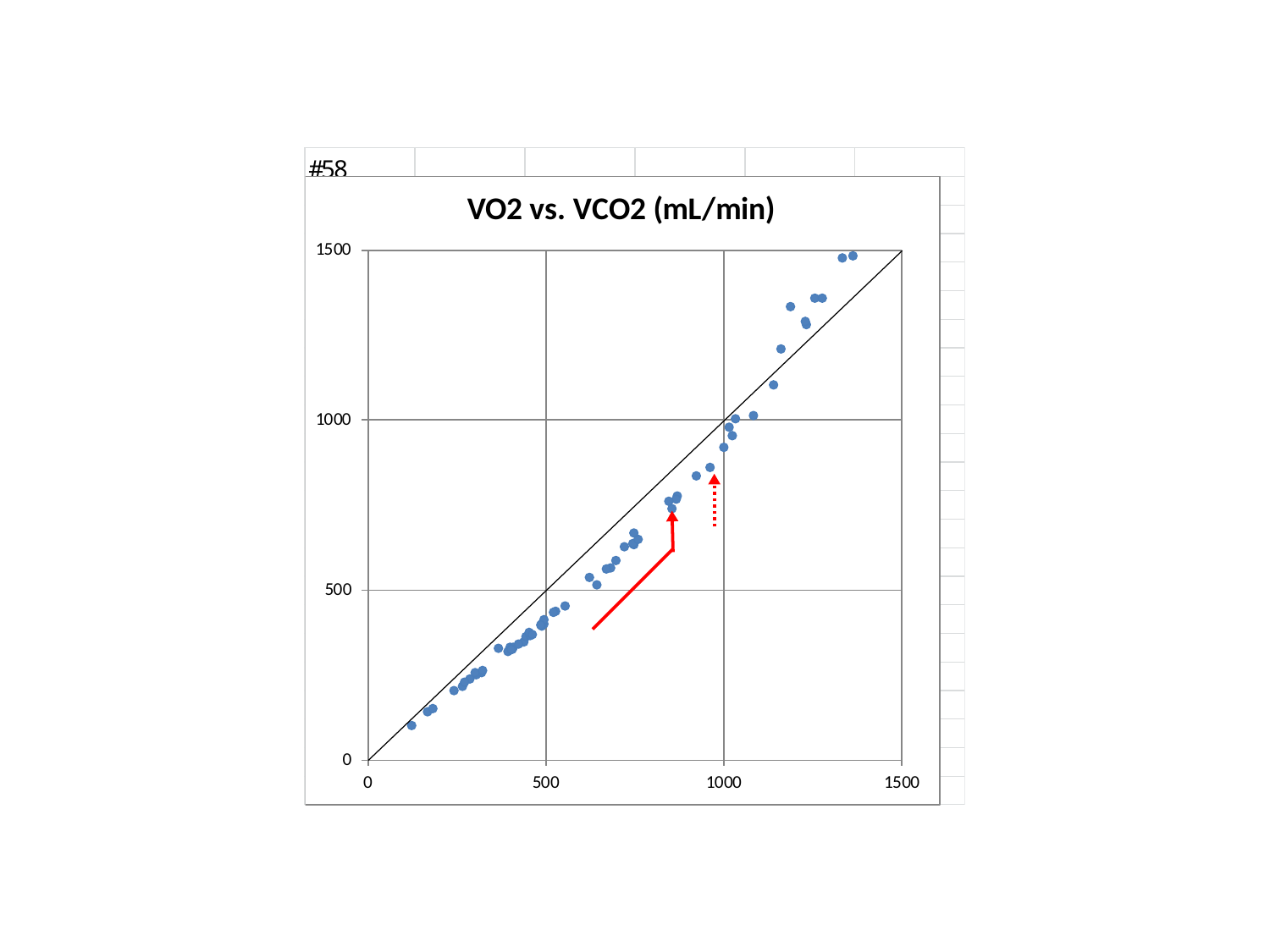

## Slide 61
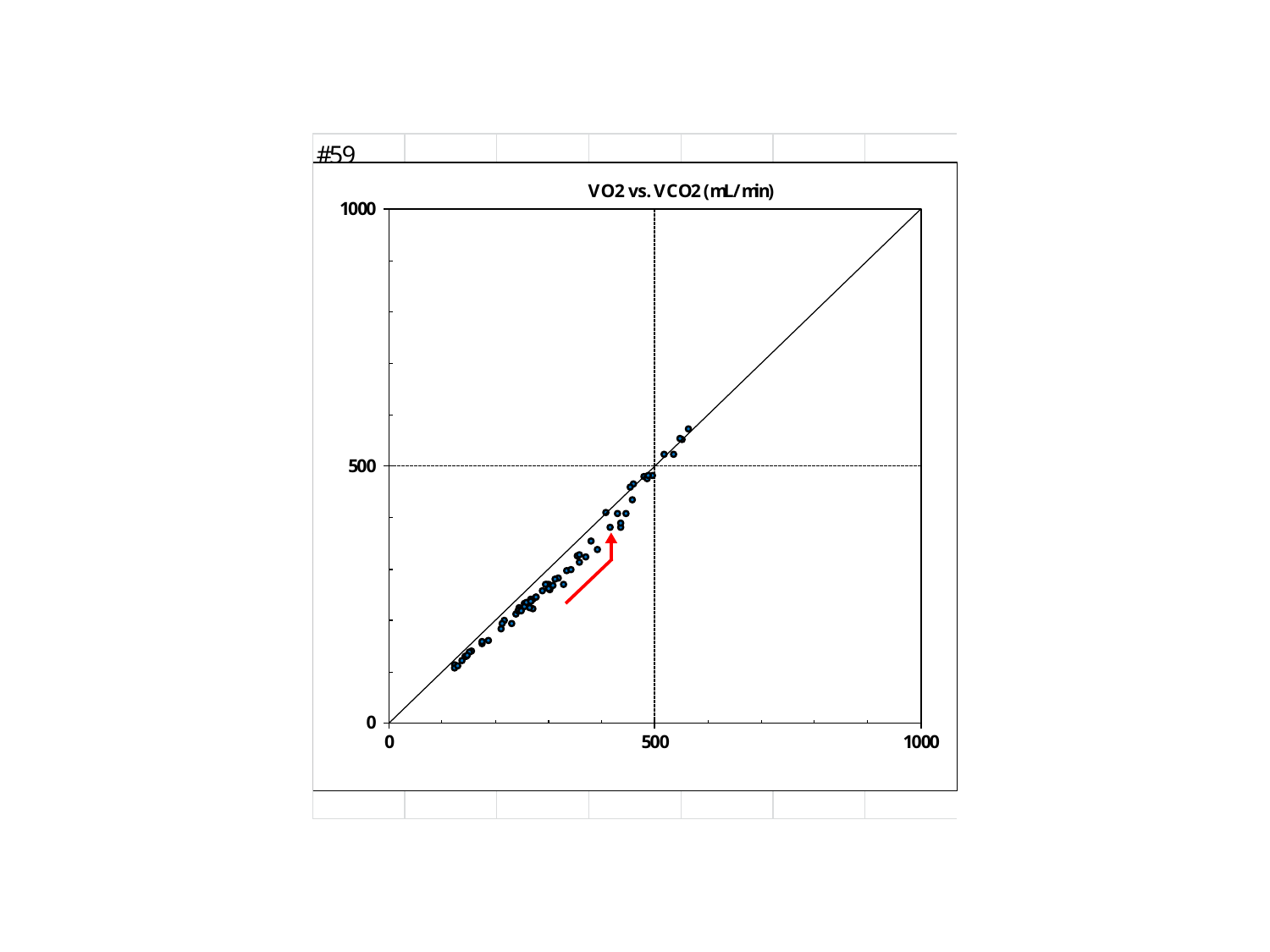

## Slide 62
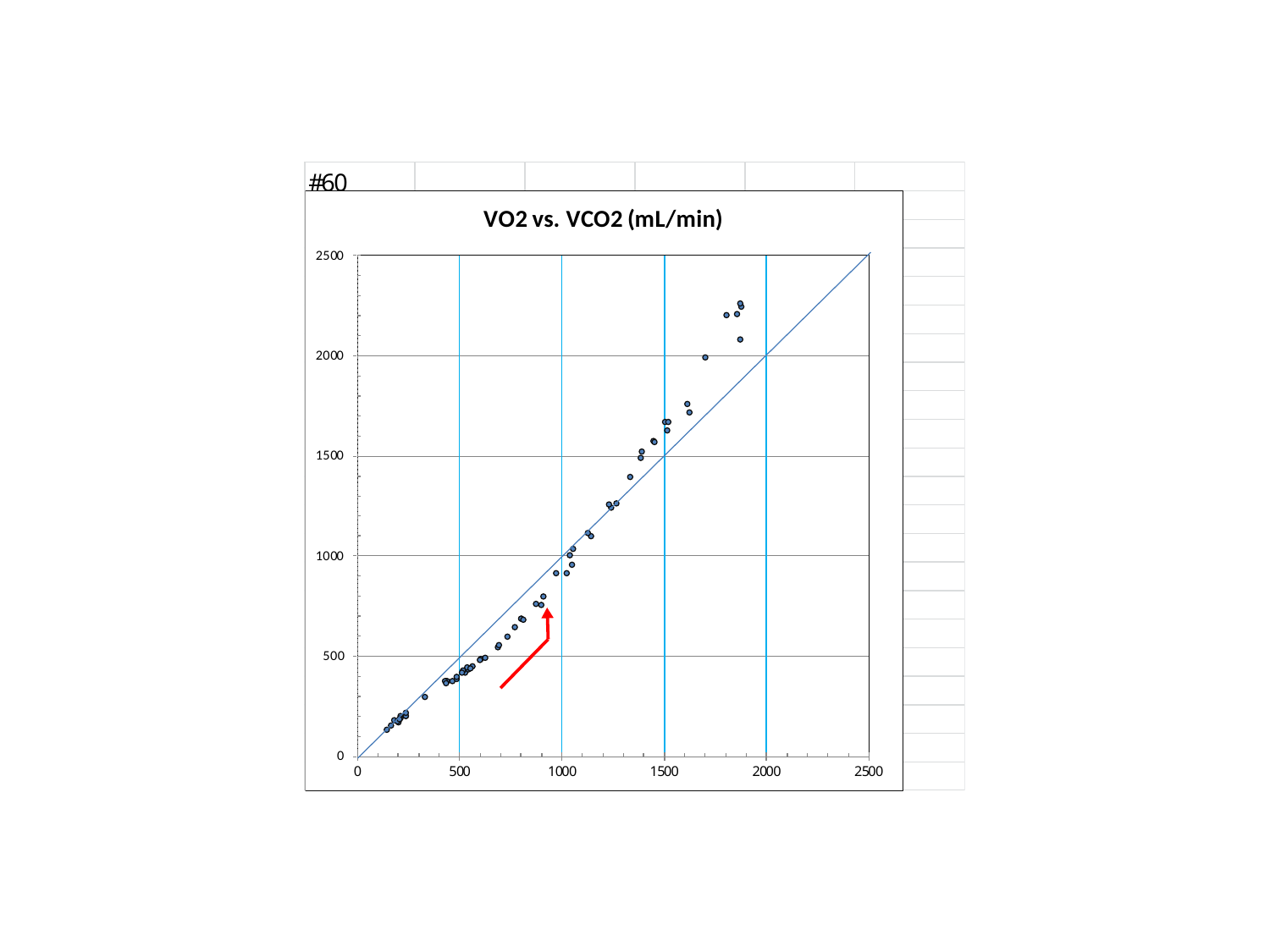

## Slide 63
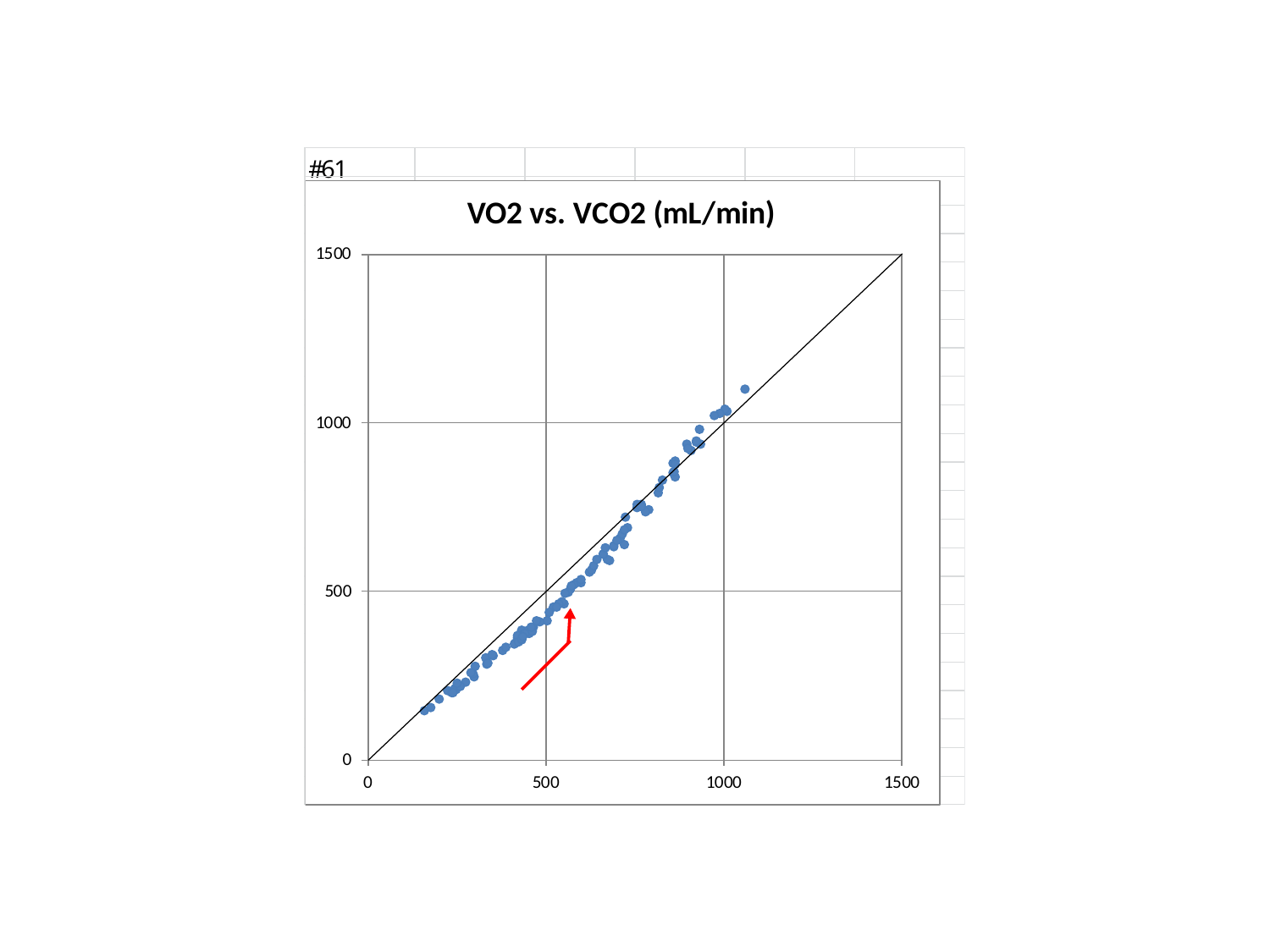

## Slide 64
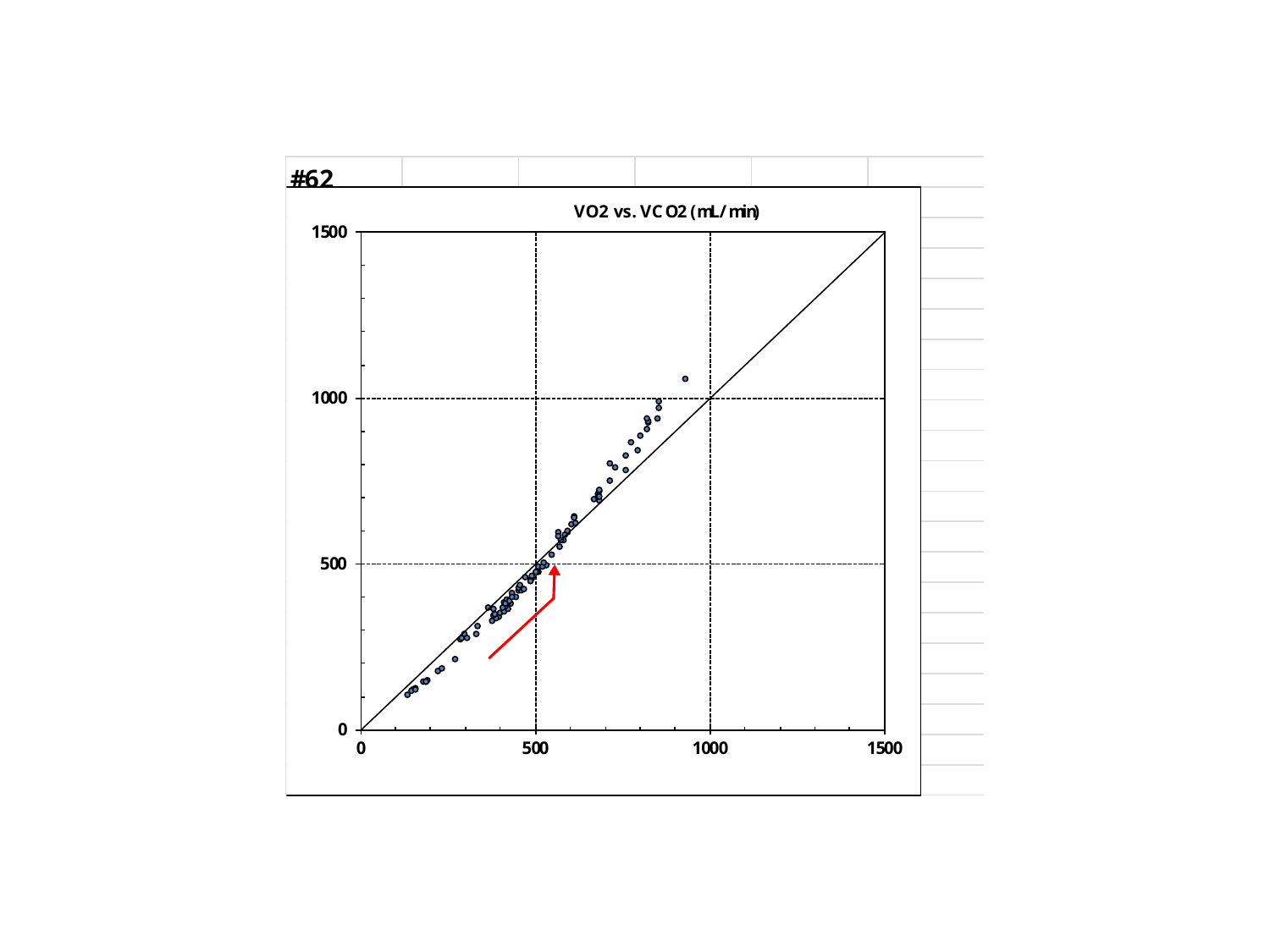

## Slide 65
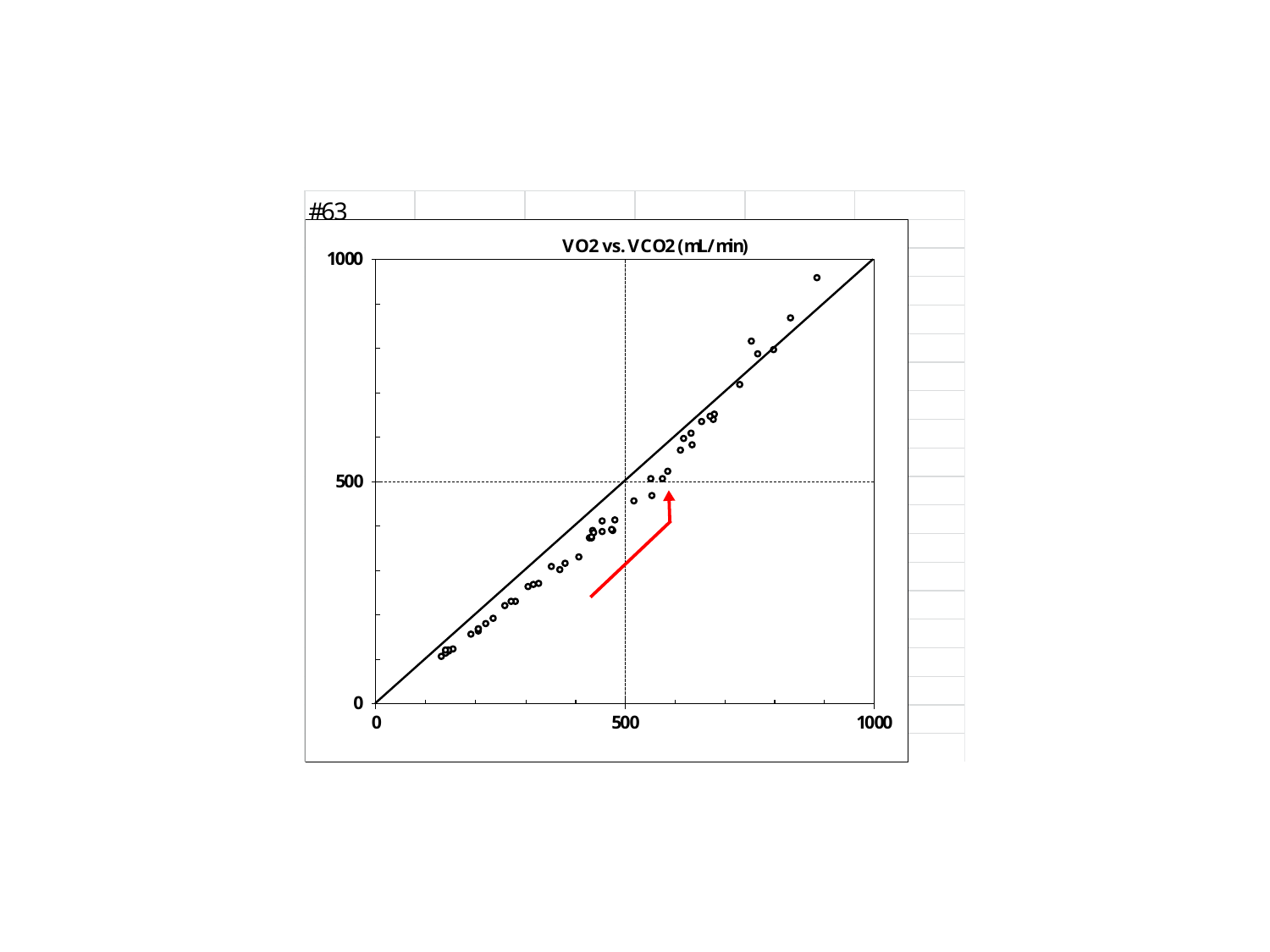

## Slide 66
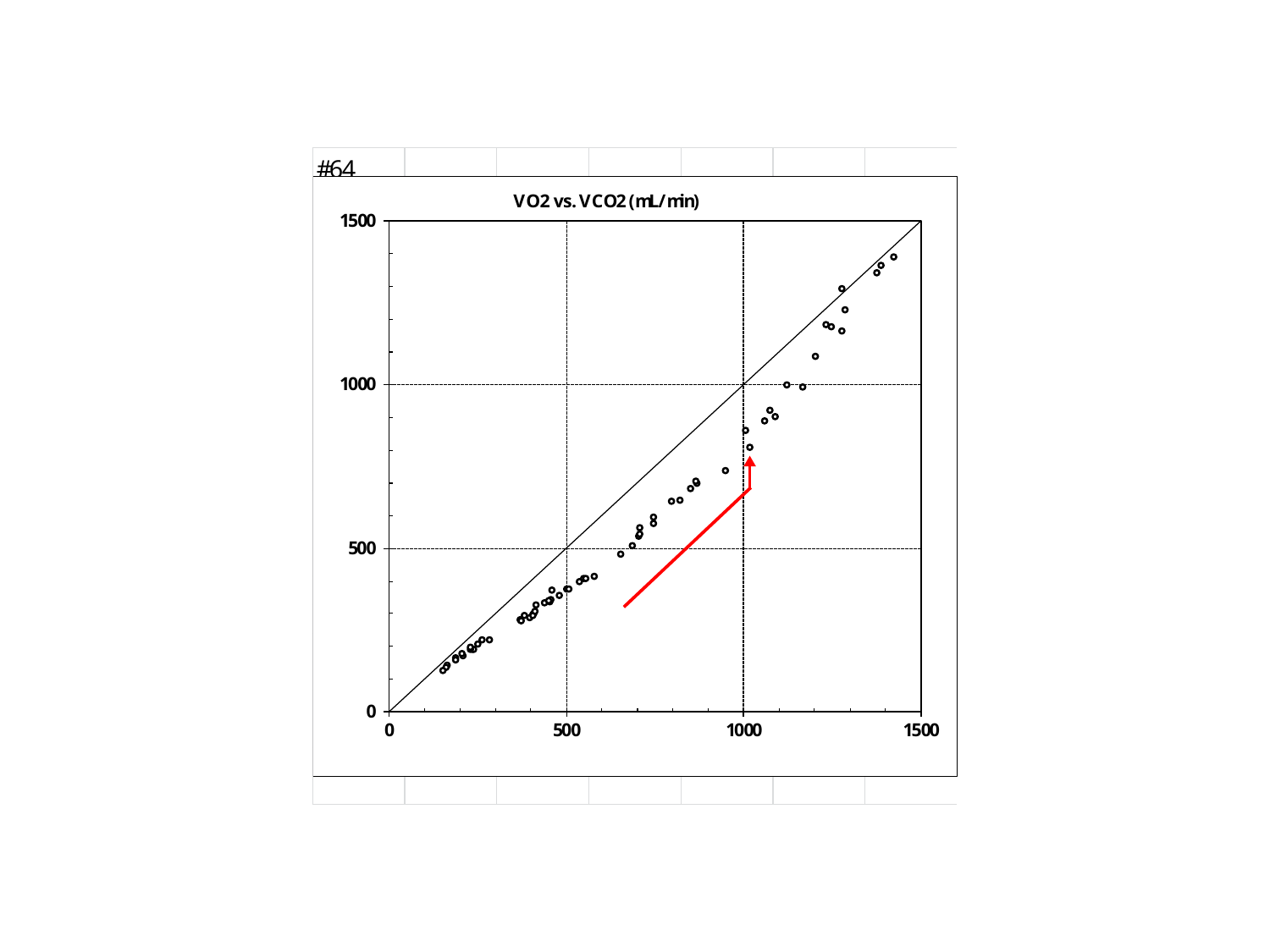

## Slide 67
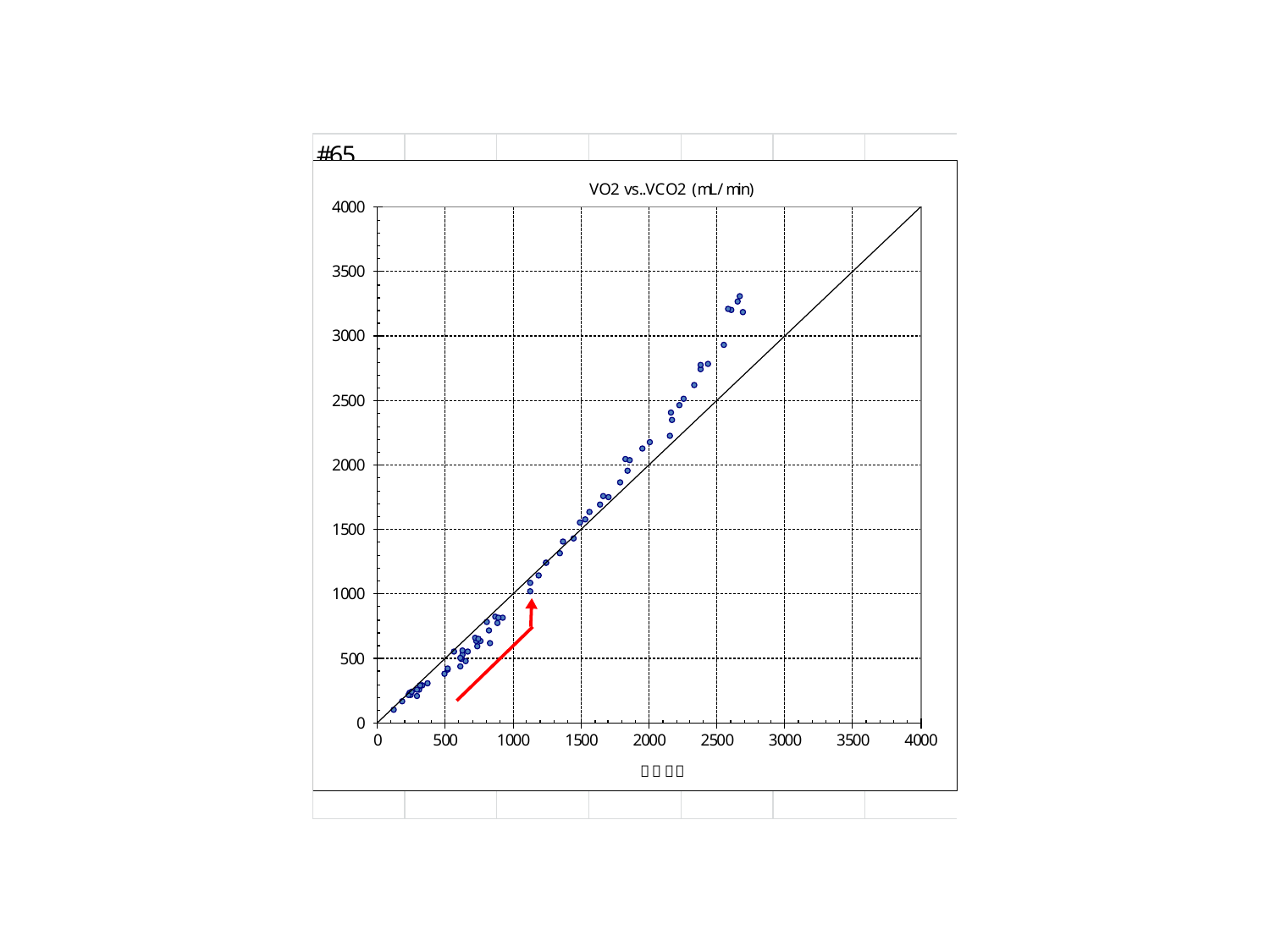

## Slide 68
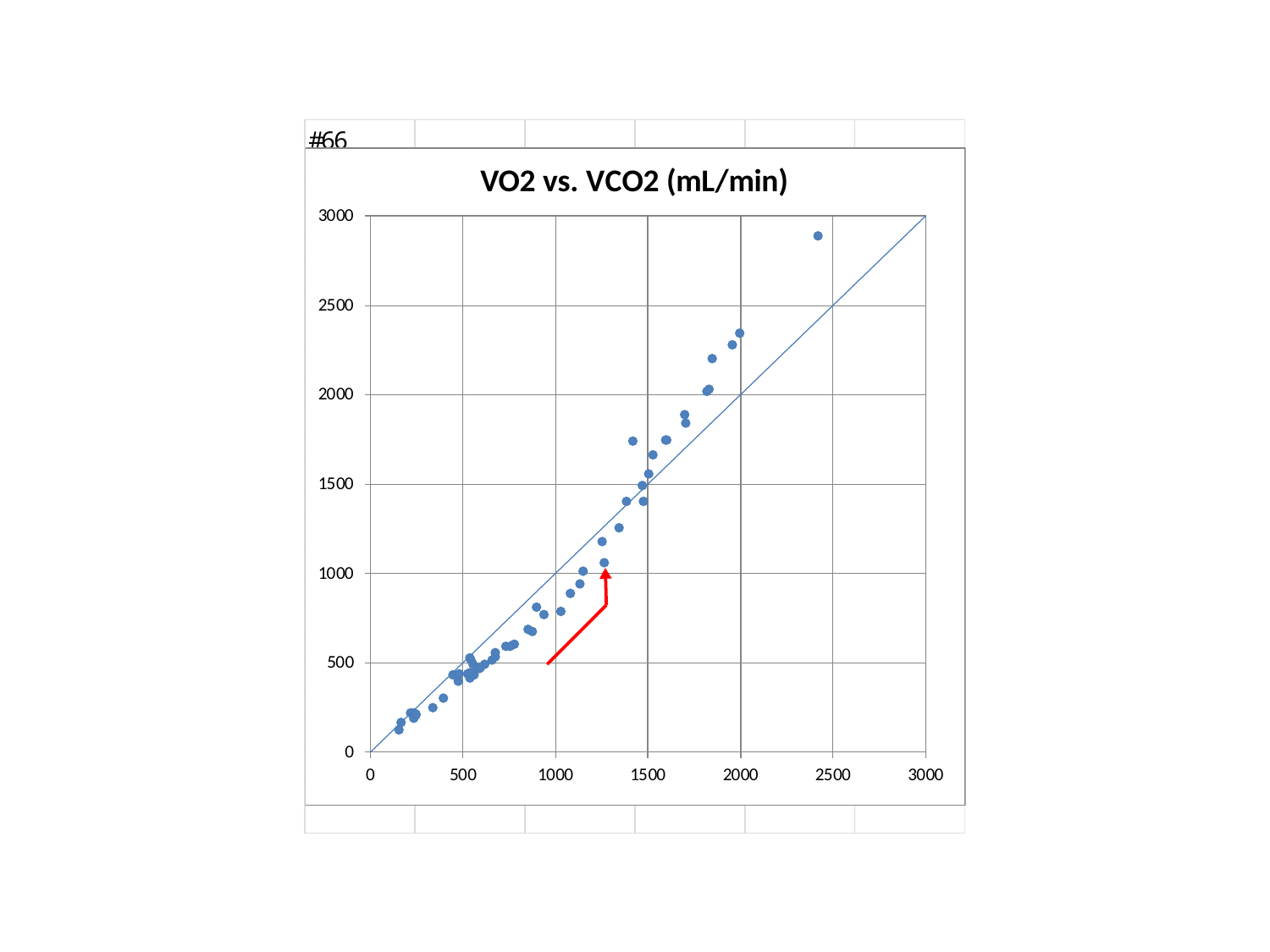

## Slide 69
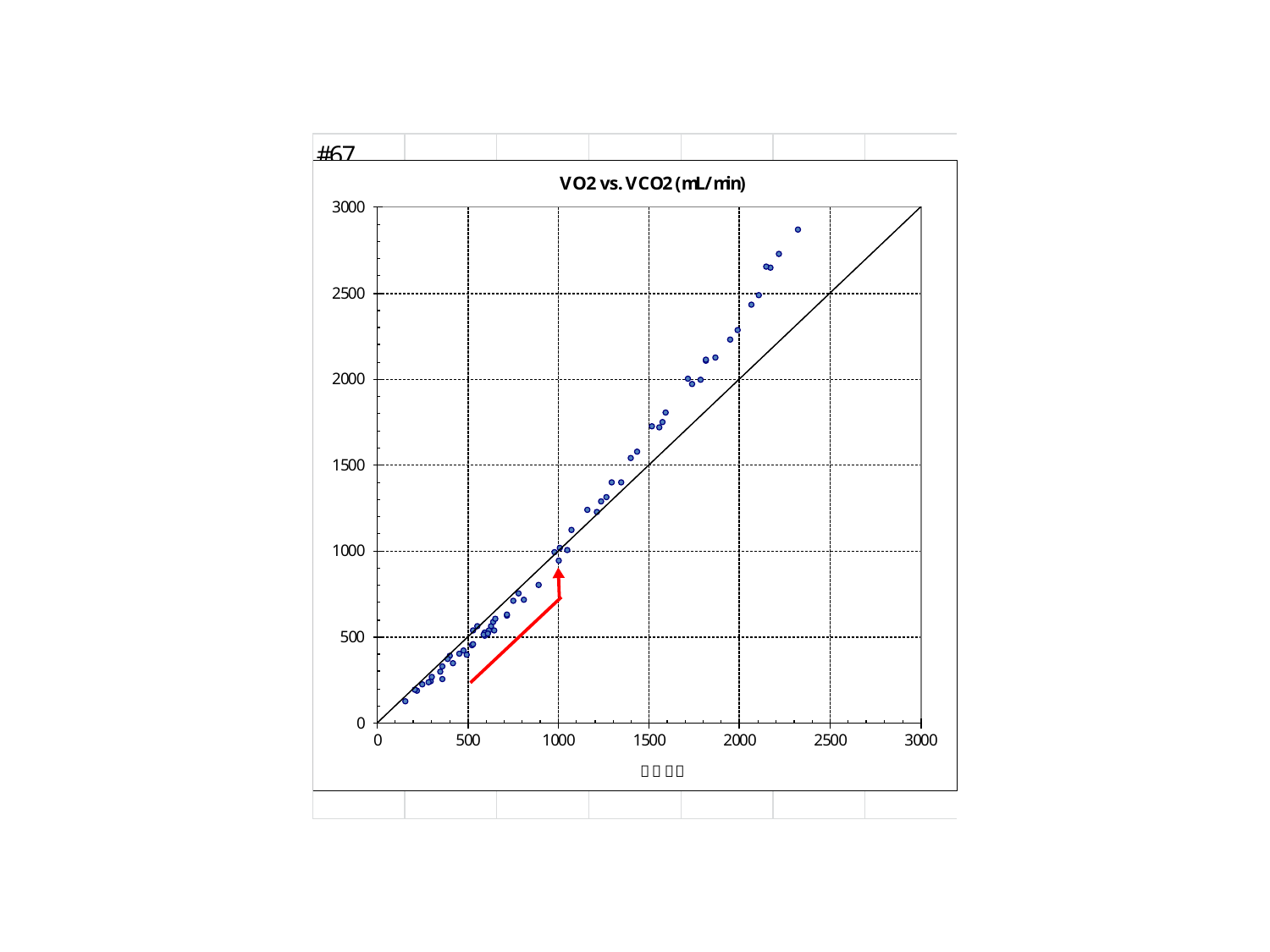

## Slide 70
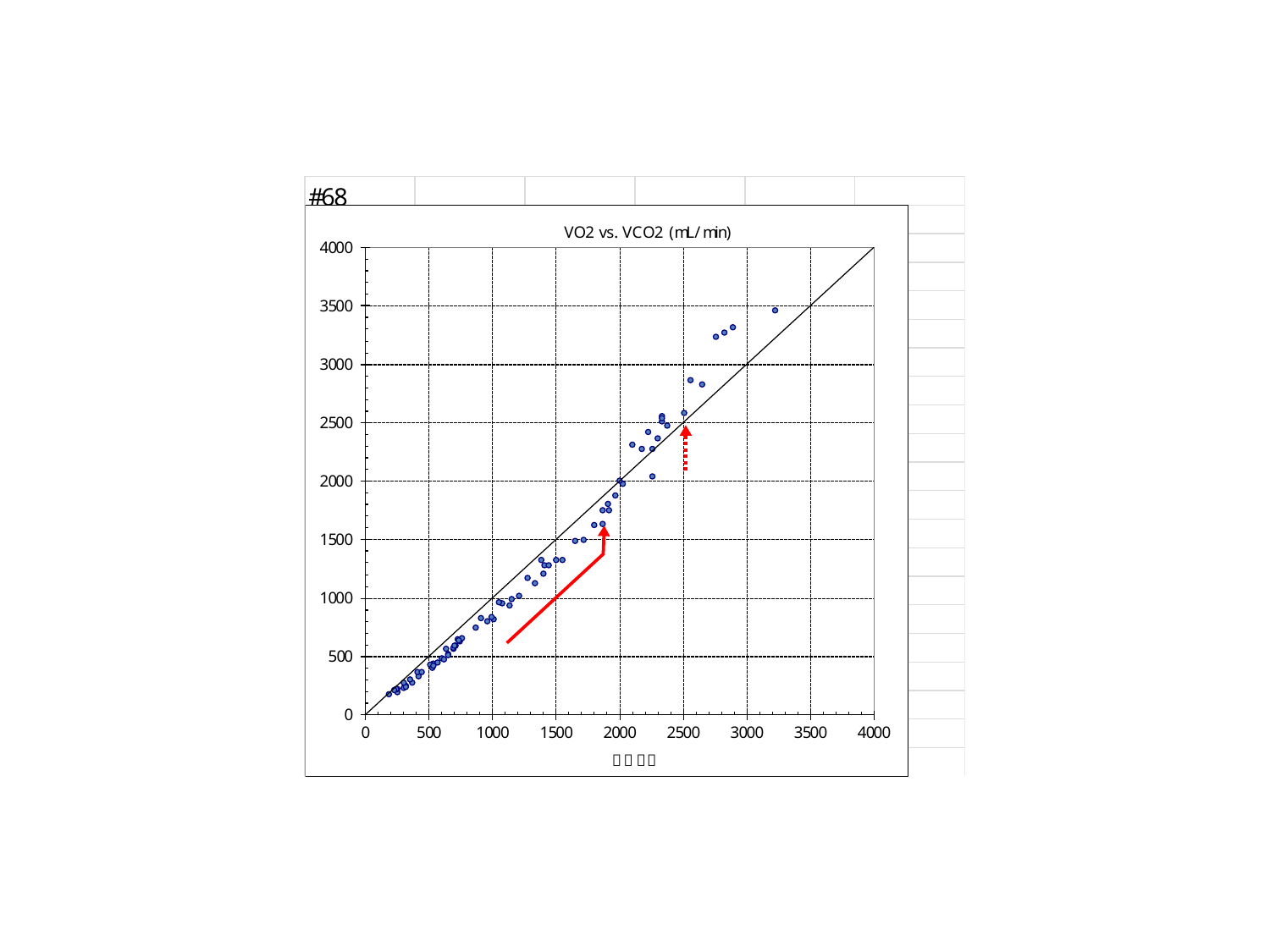

## Slide 71
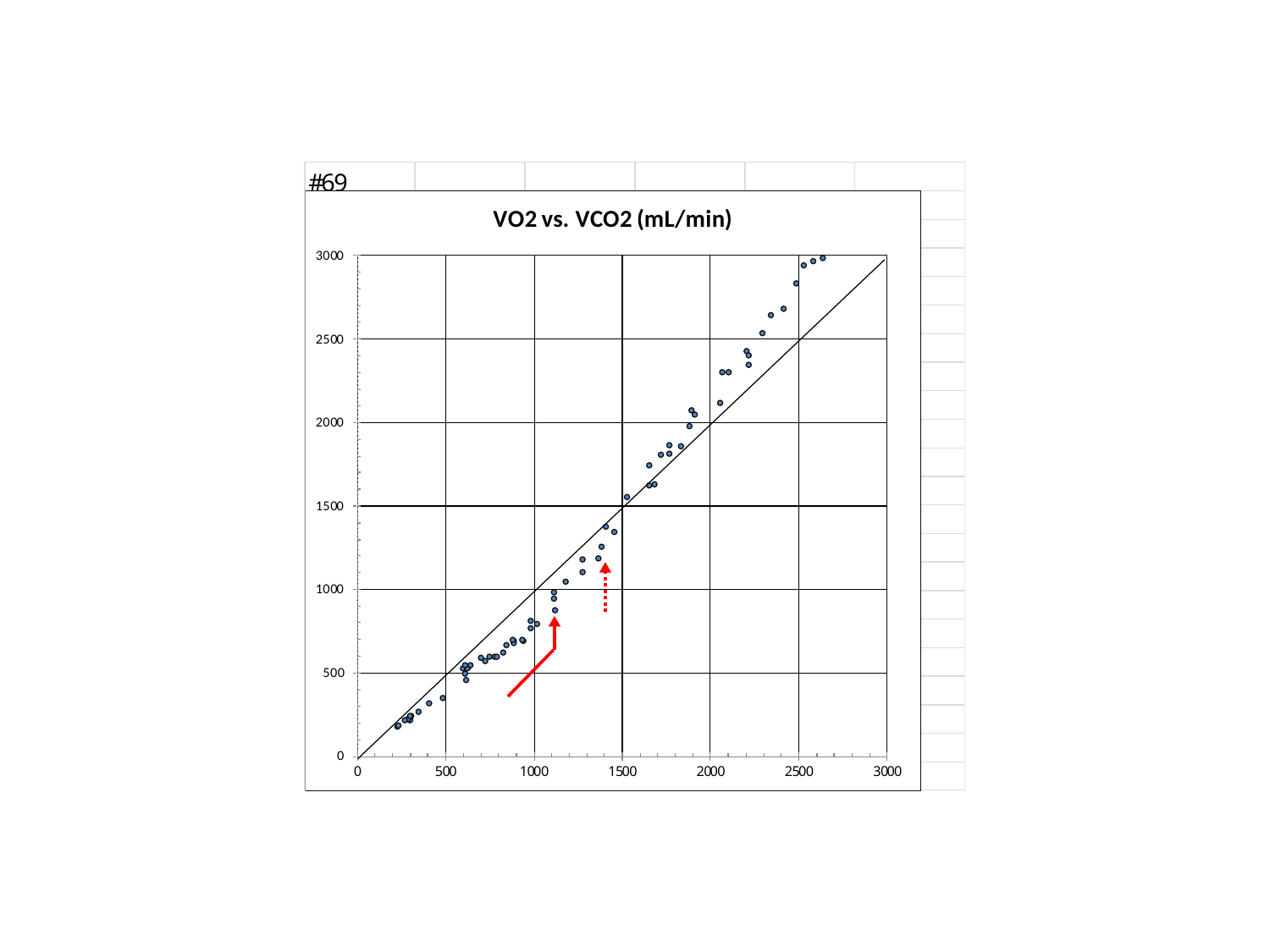

## Slide 72
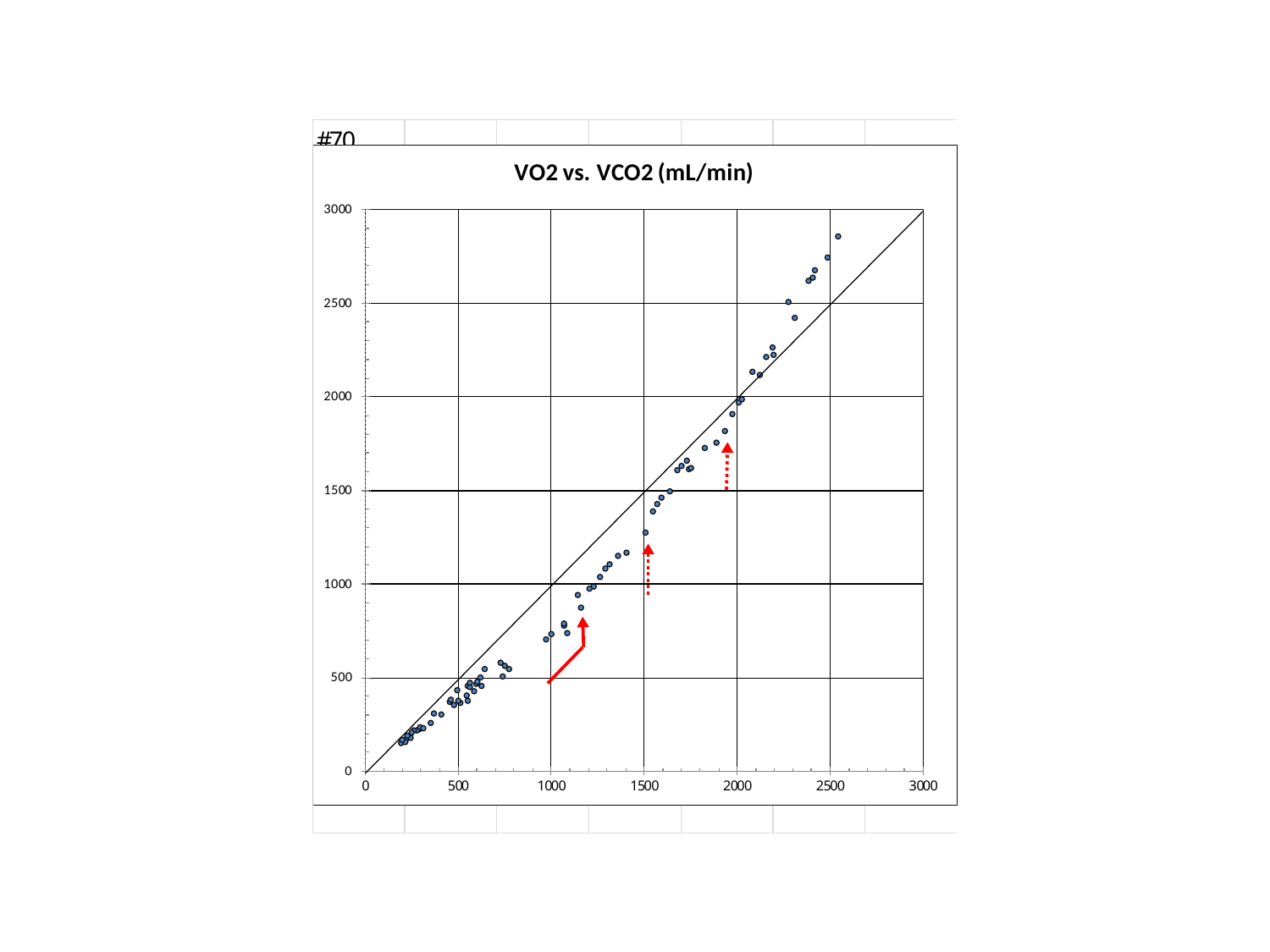

## Slide 73
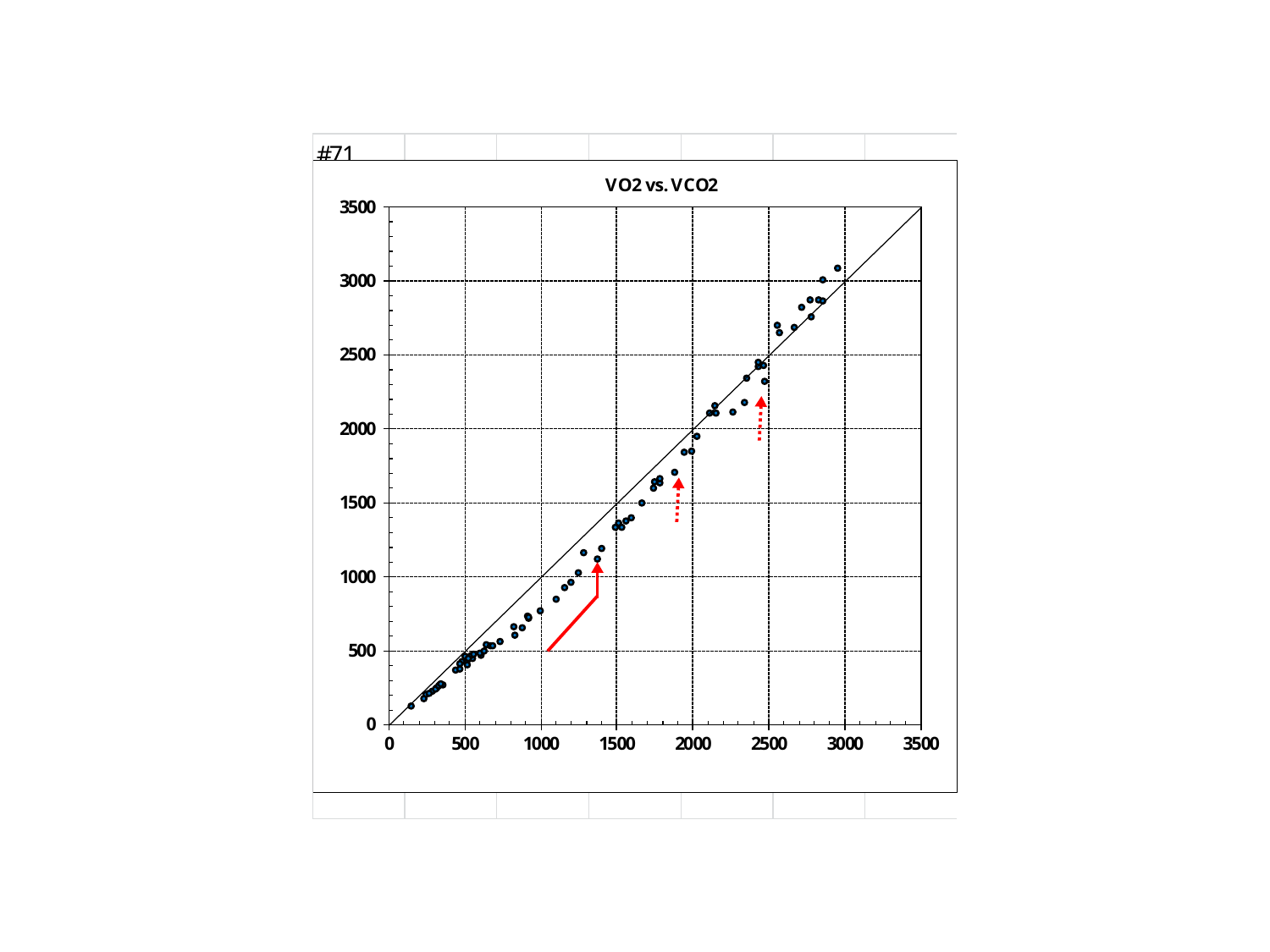

## Slide 74
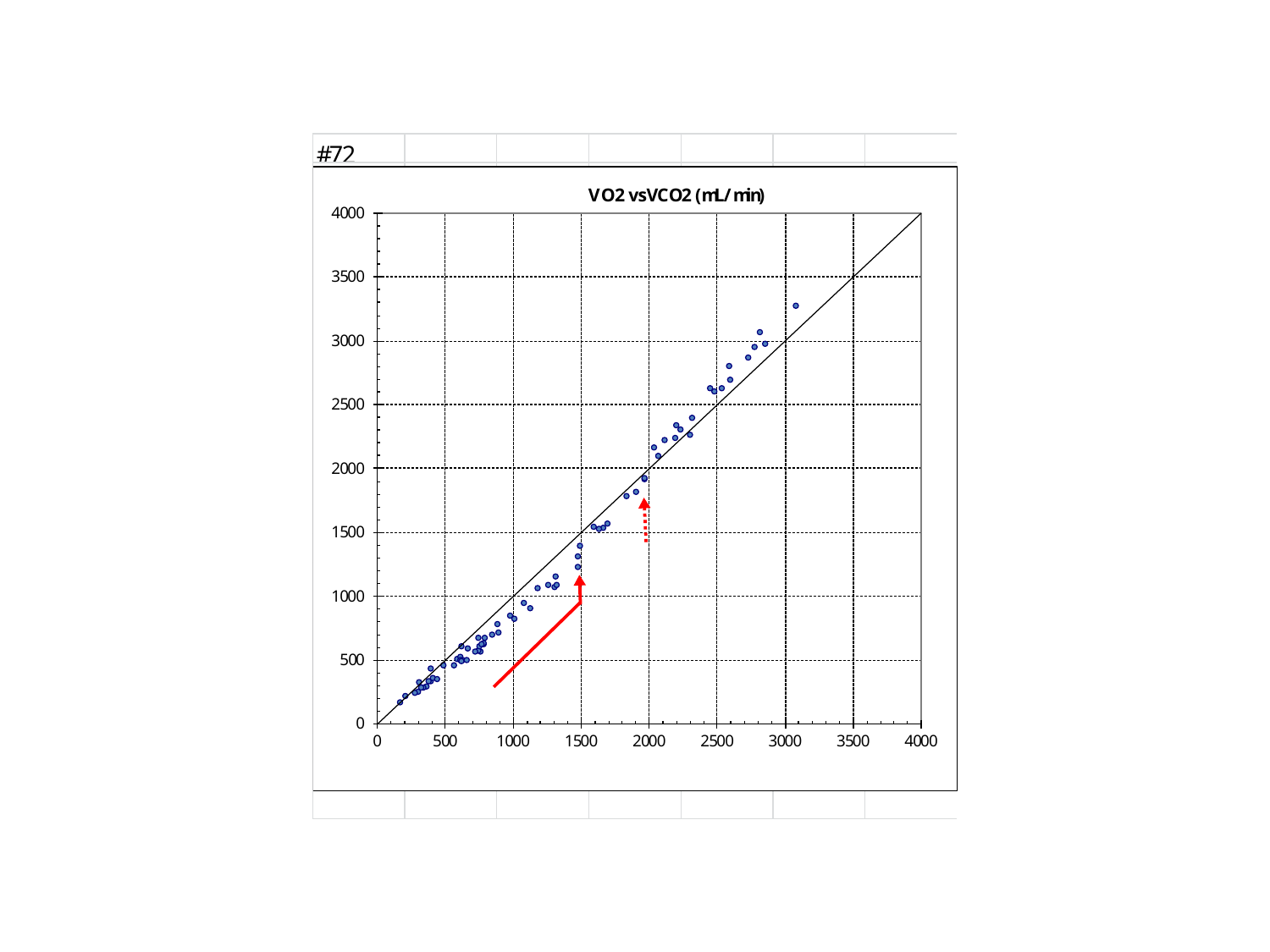

## Slide 75
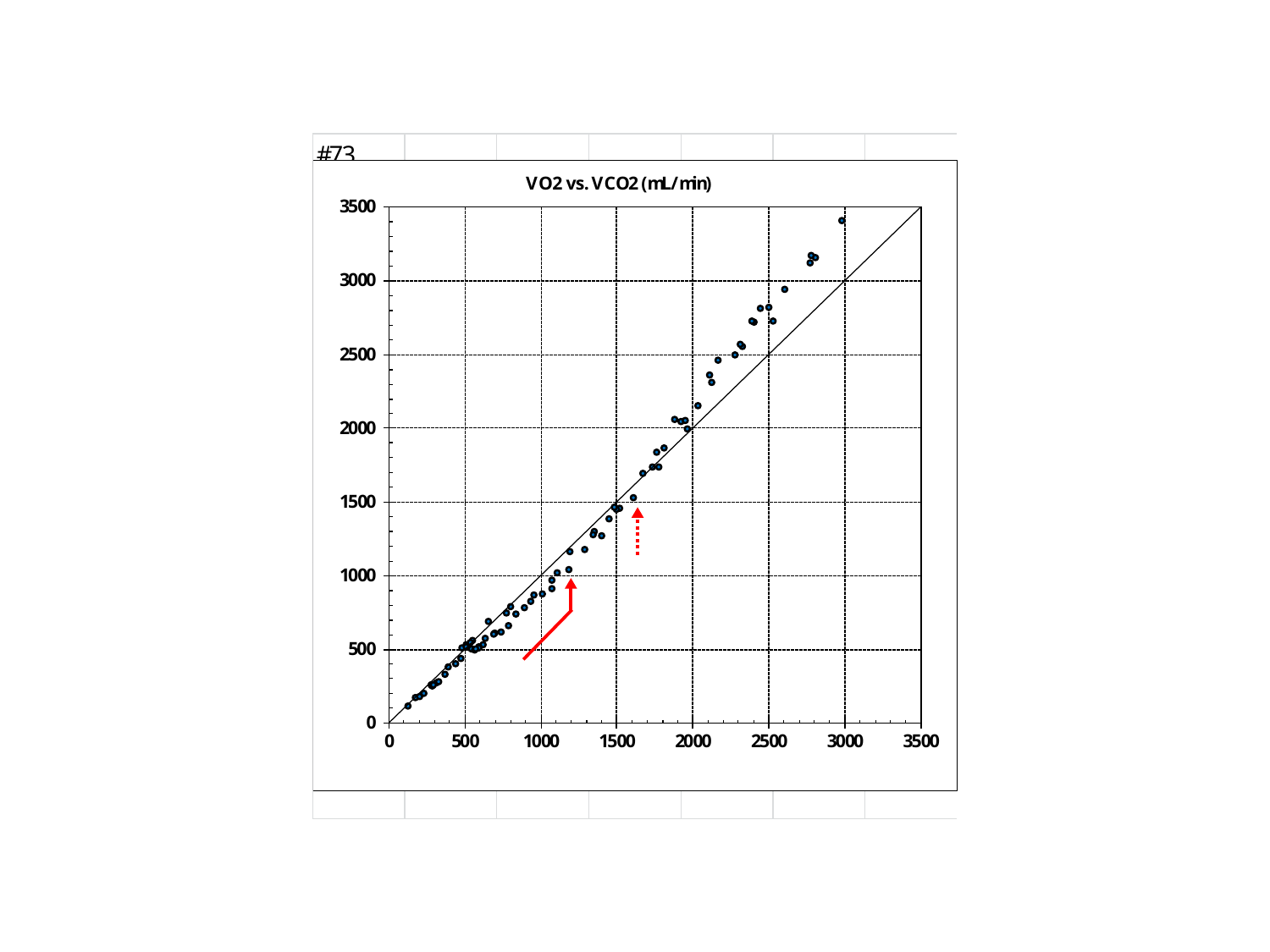

## Slide 76
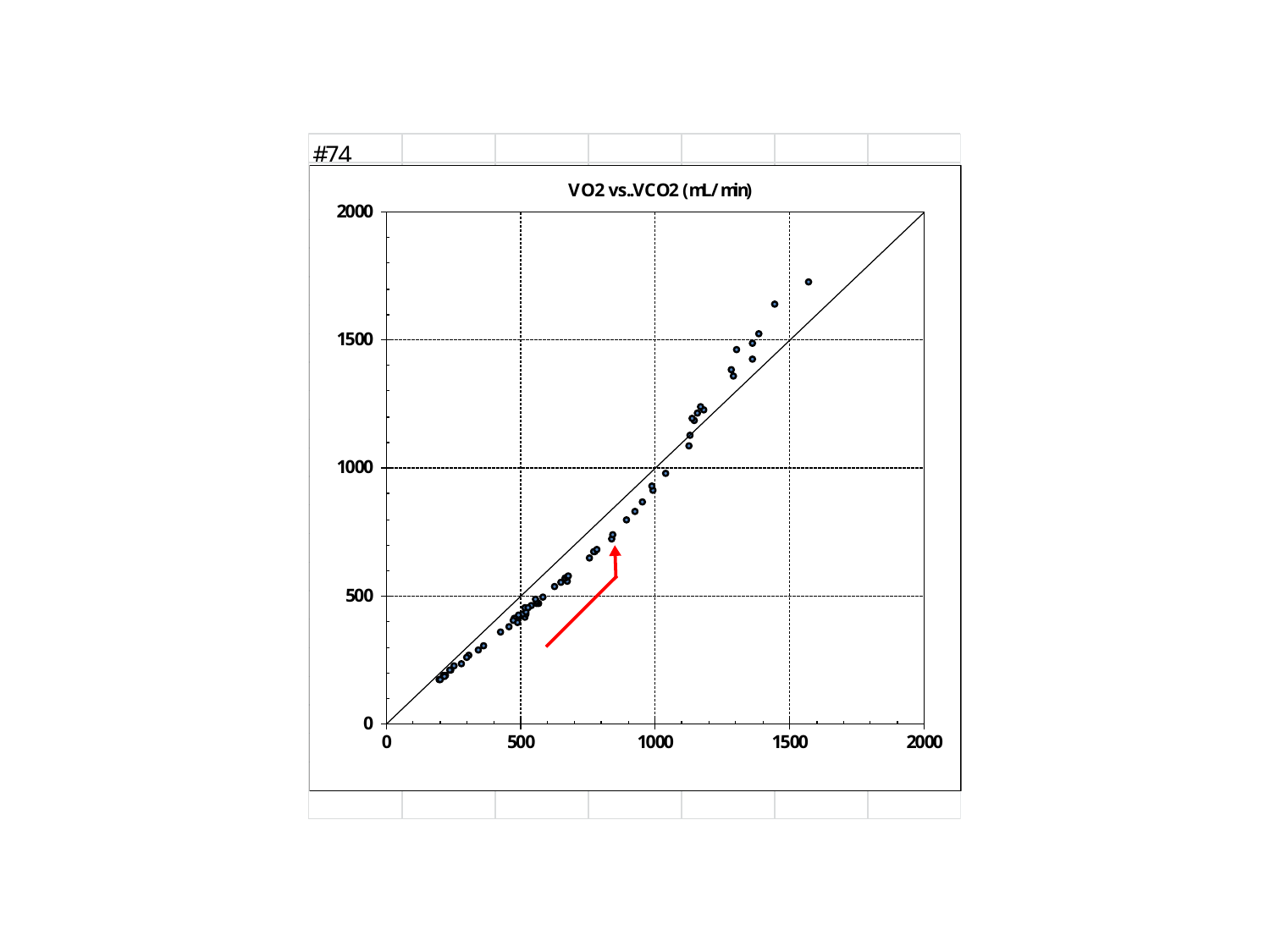

## Slide 77
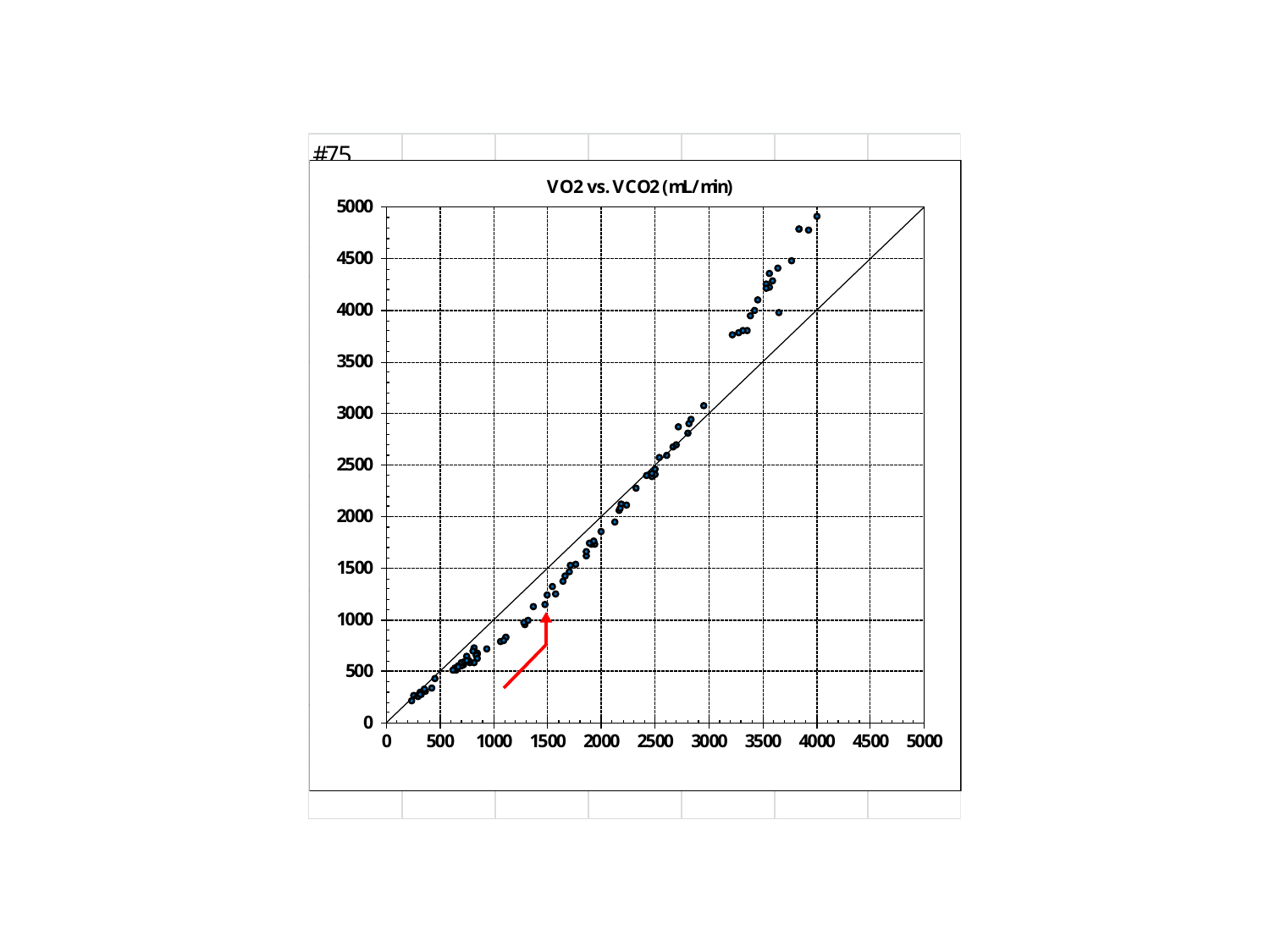

## Slide 78
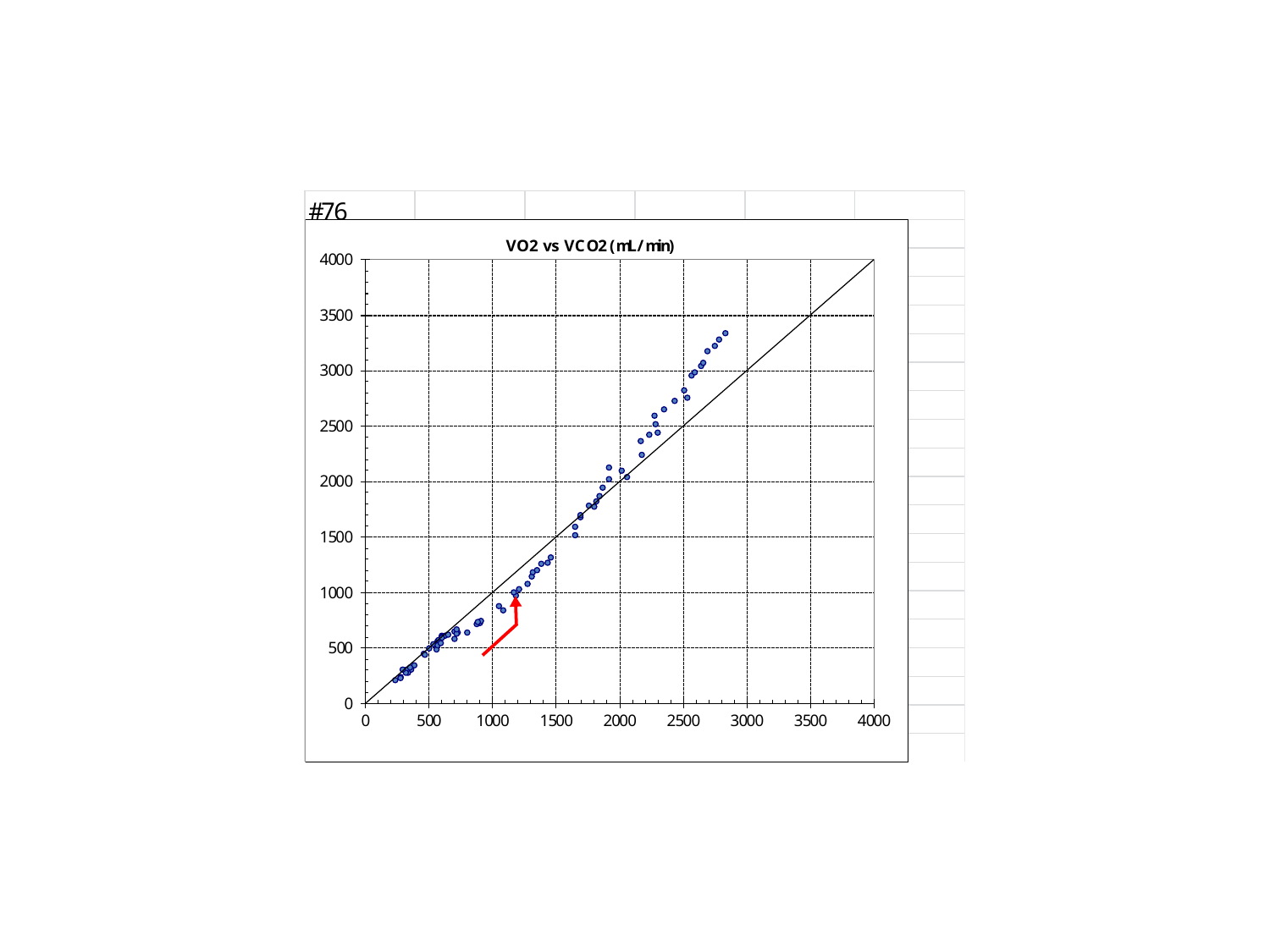

## Slide 79
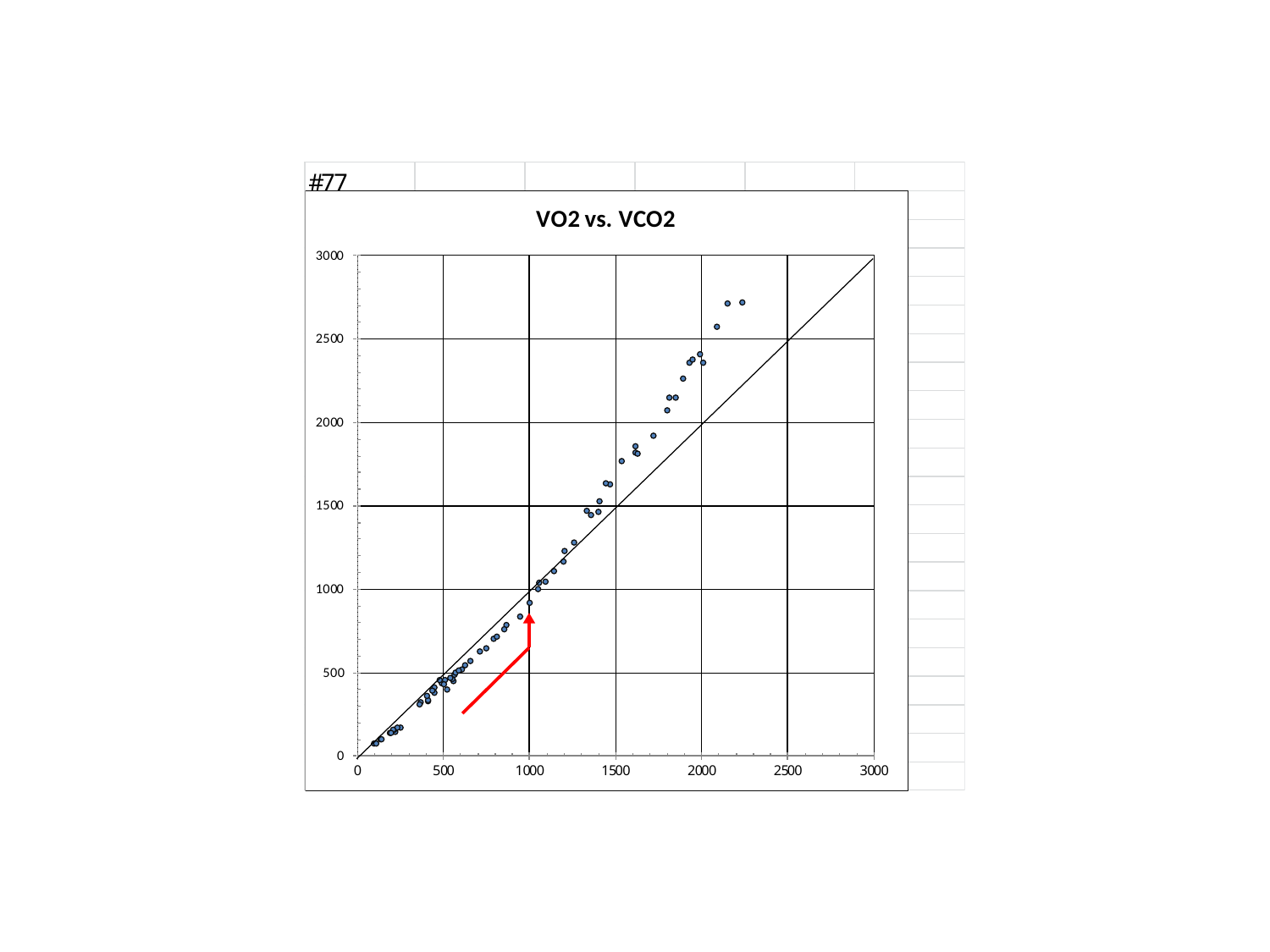

## Slide 80
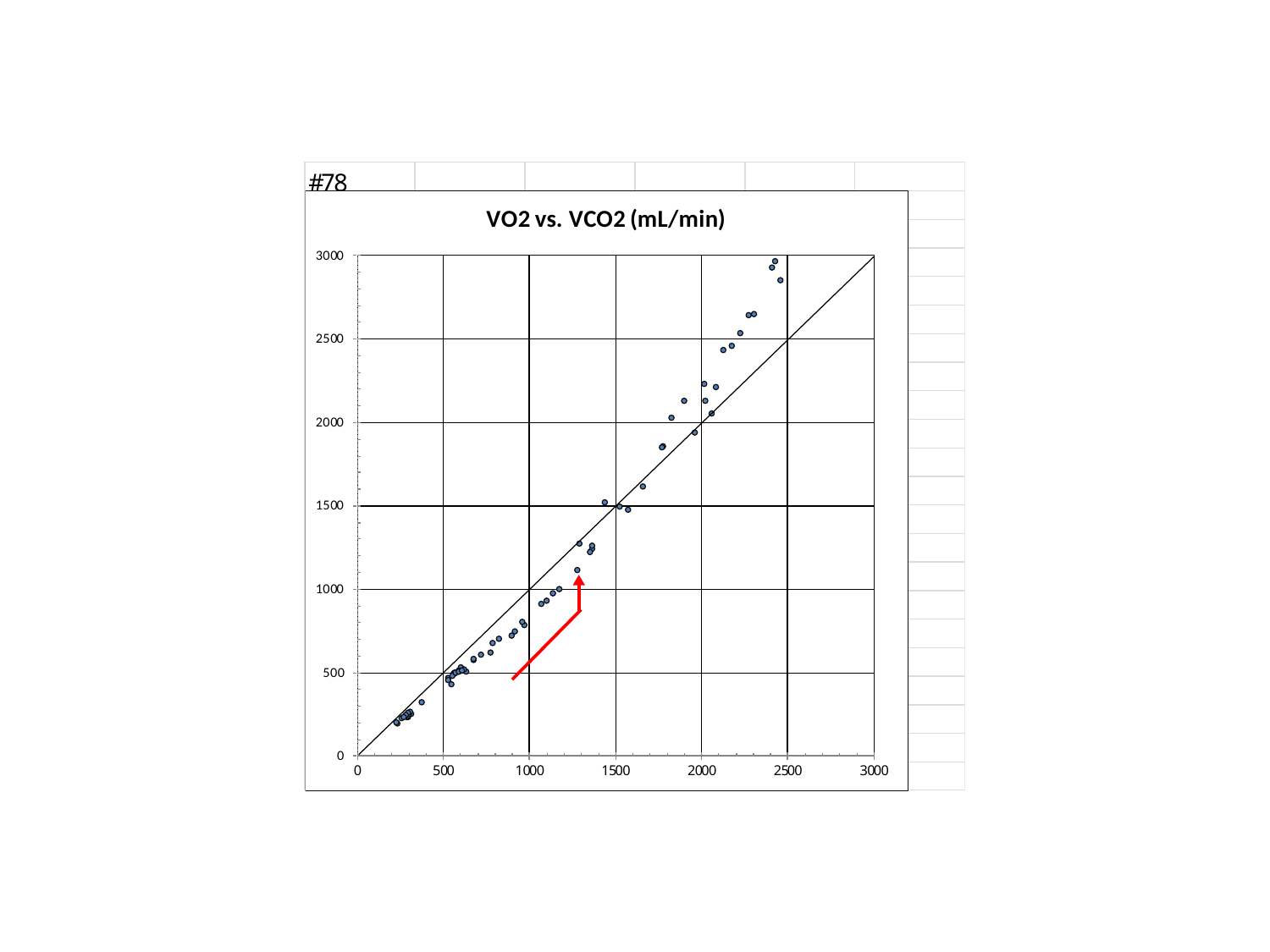

## Slide 81
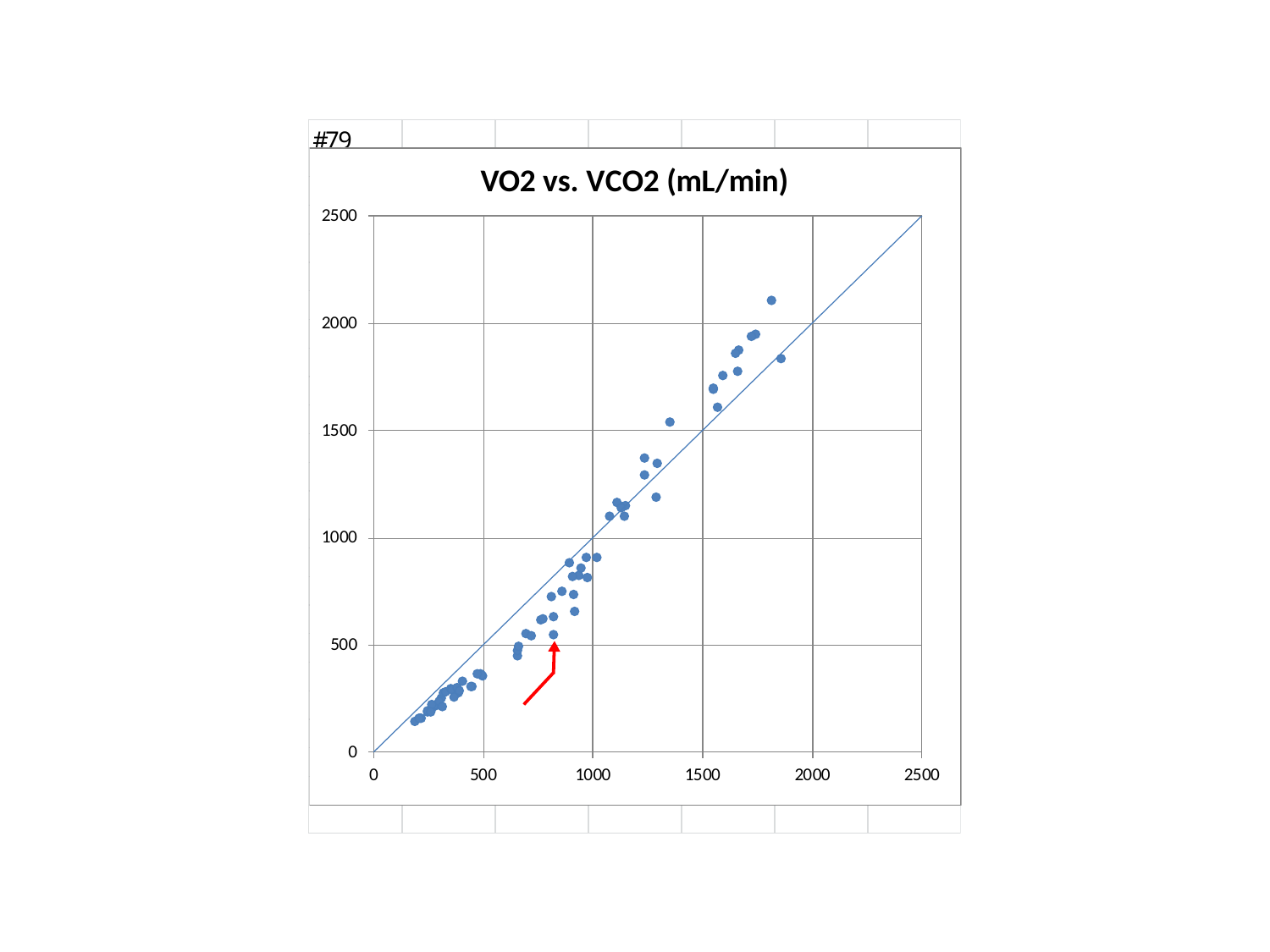

## Slide 82
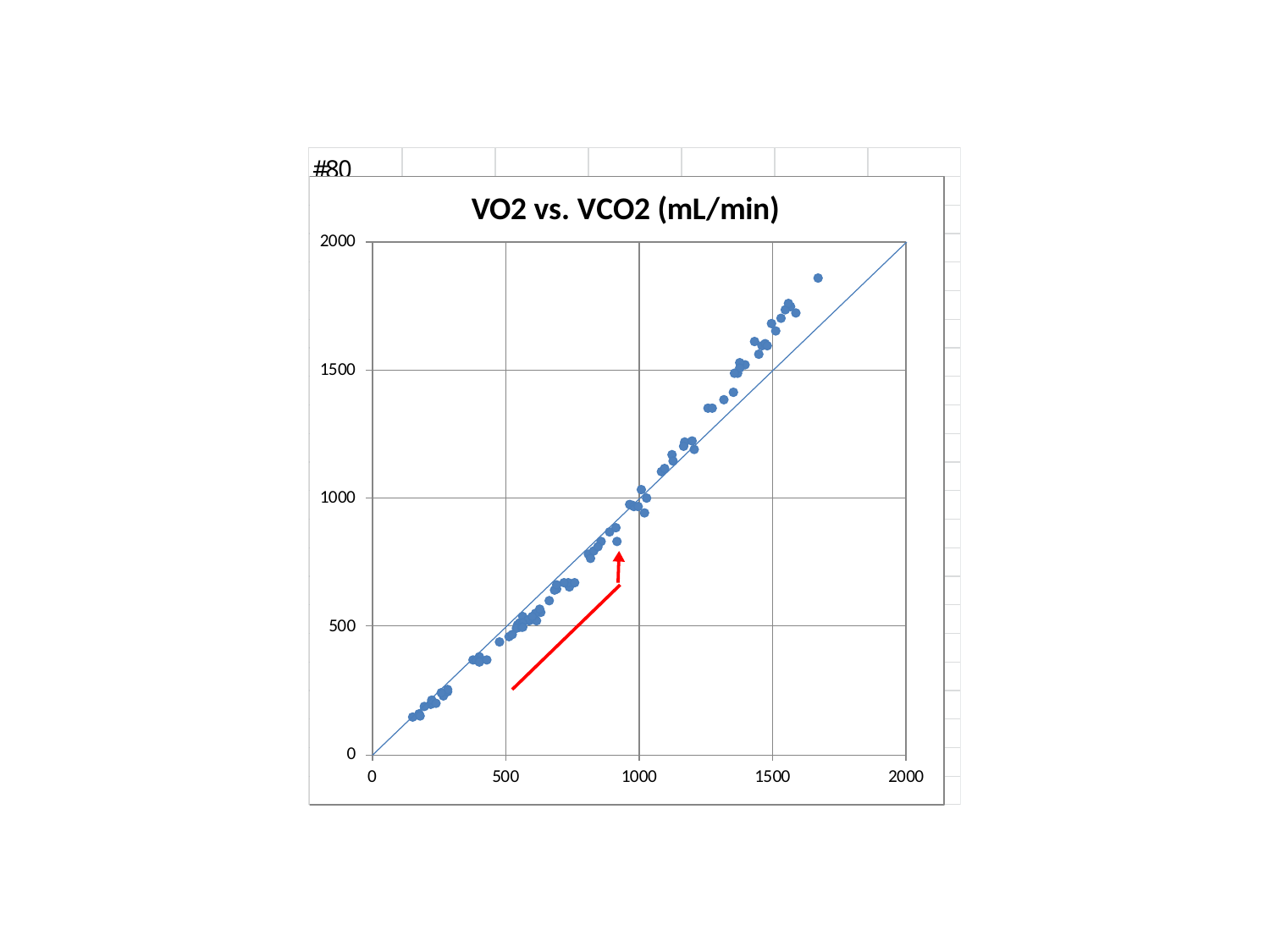

## Slide 83
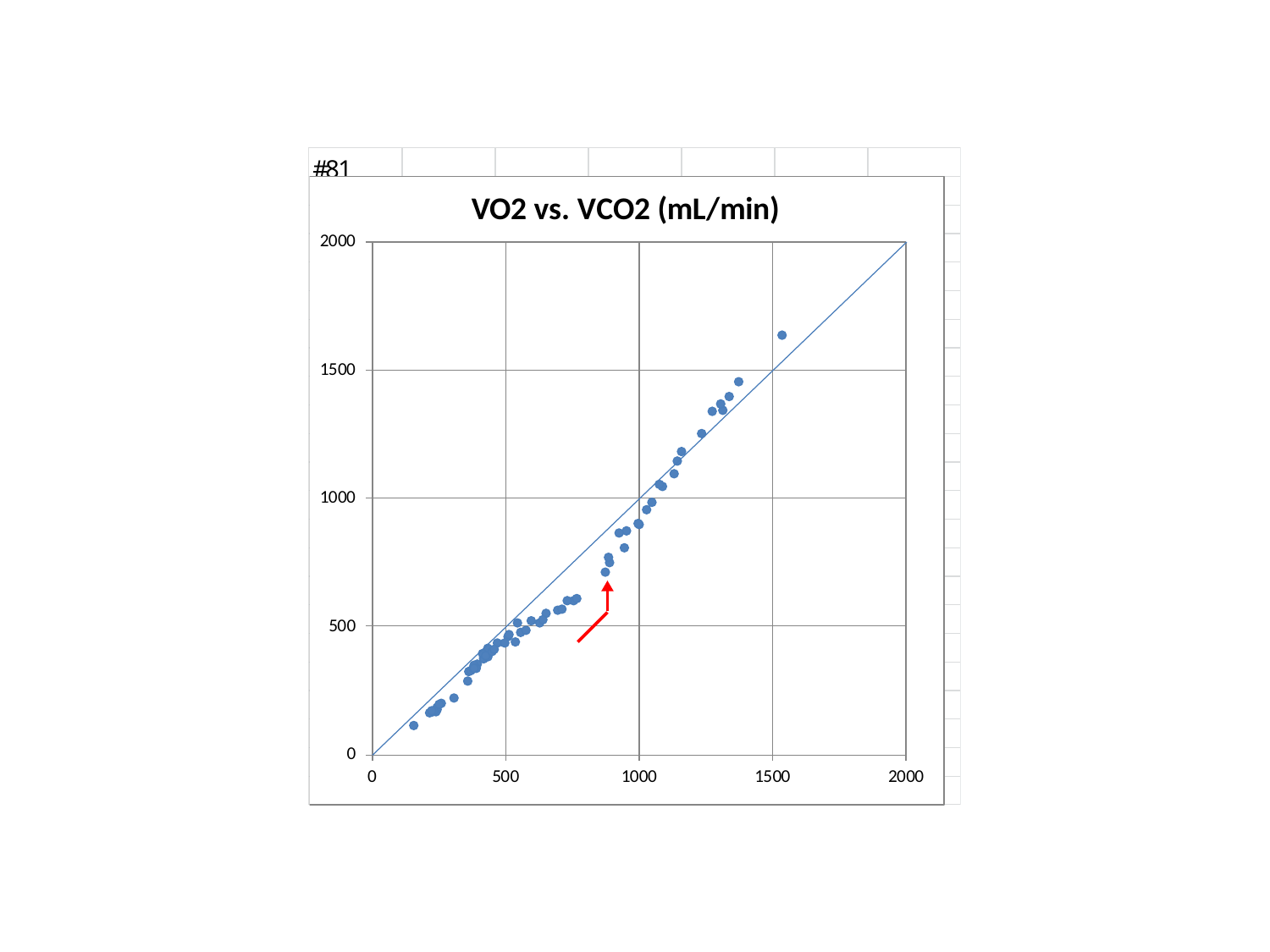

## Slide 84
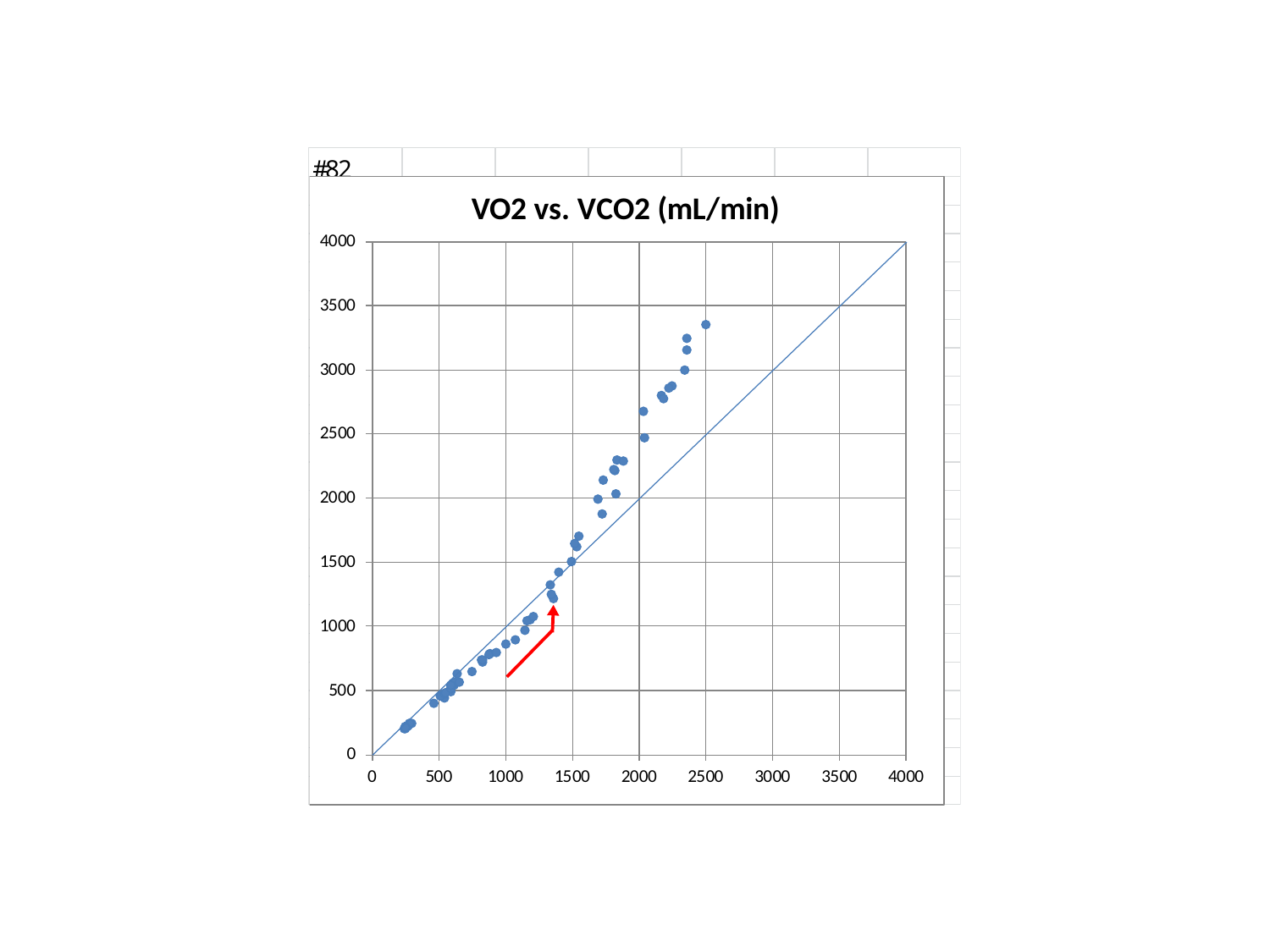

## Slide 85
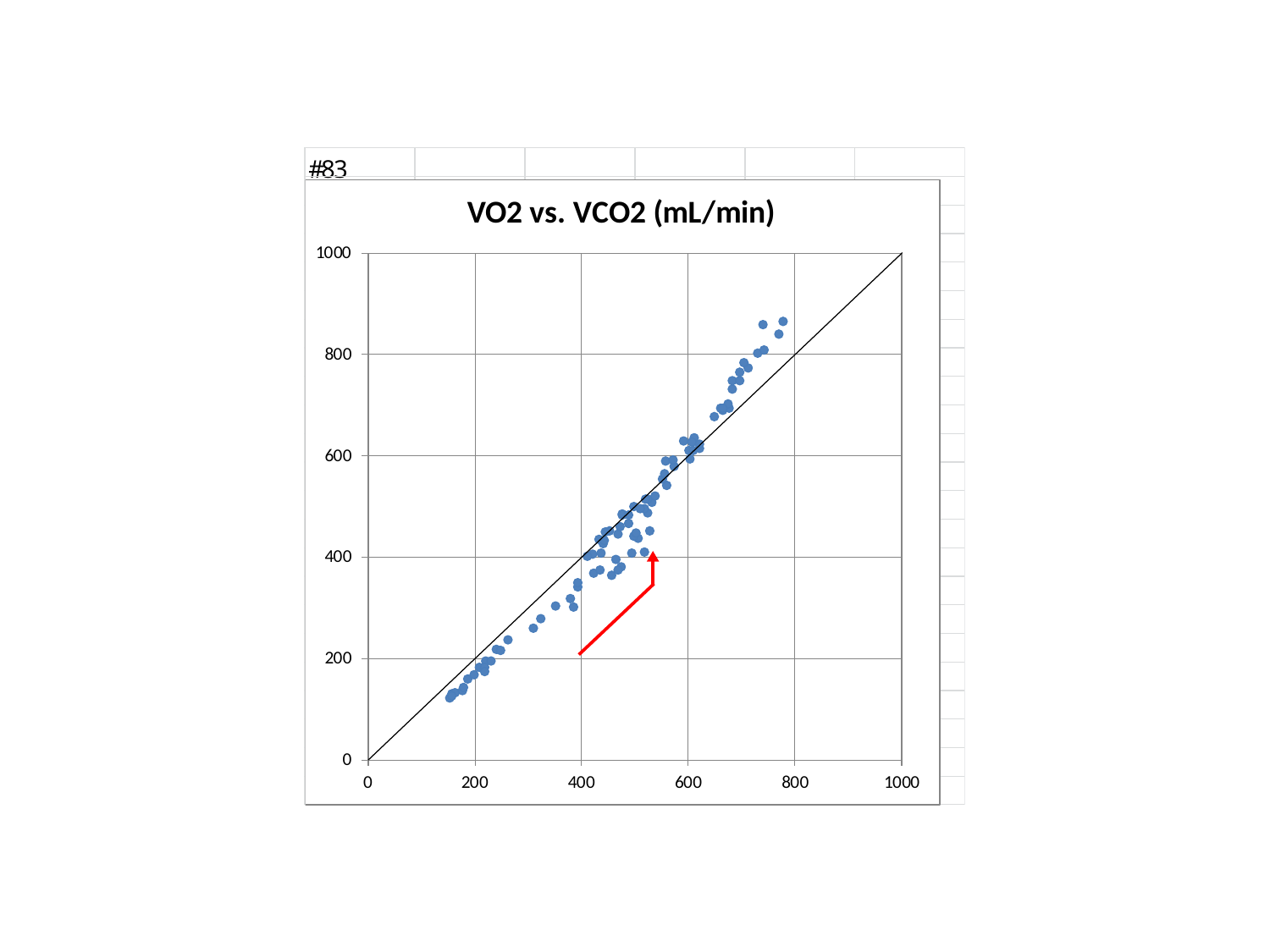

## Slide 86
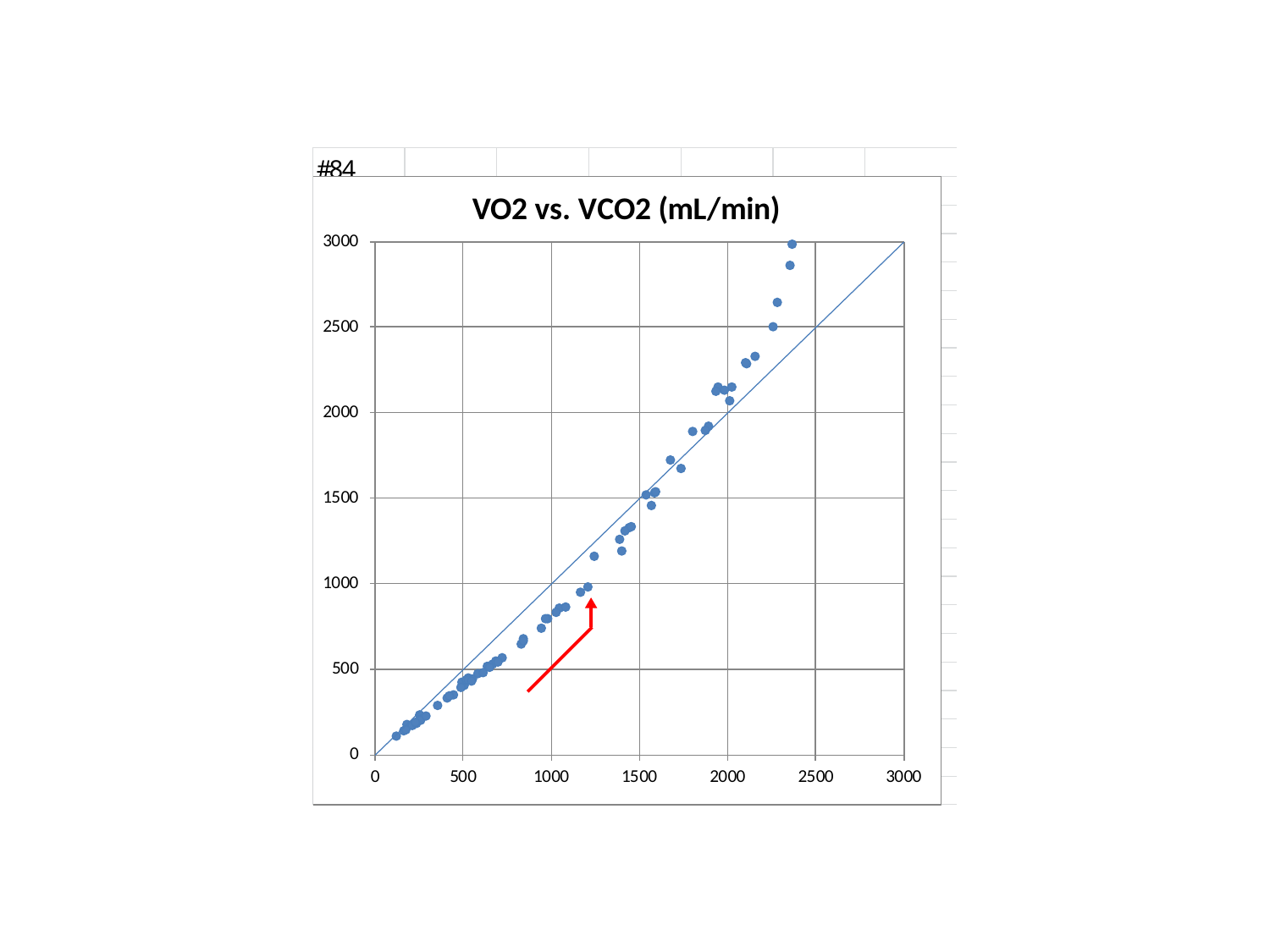

## Slide 87
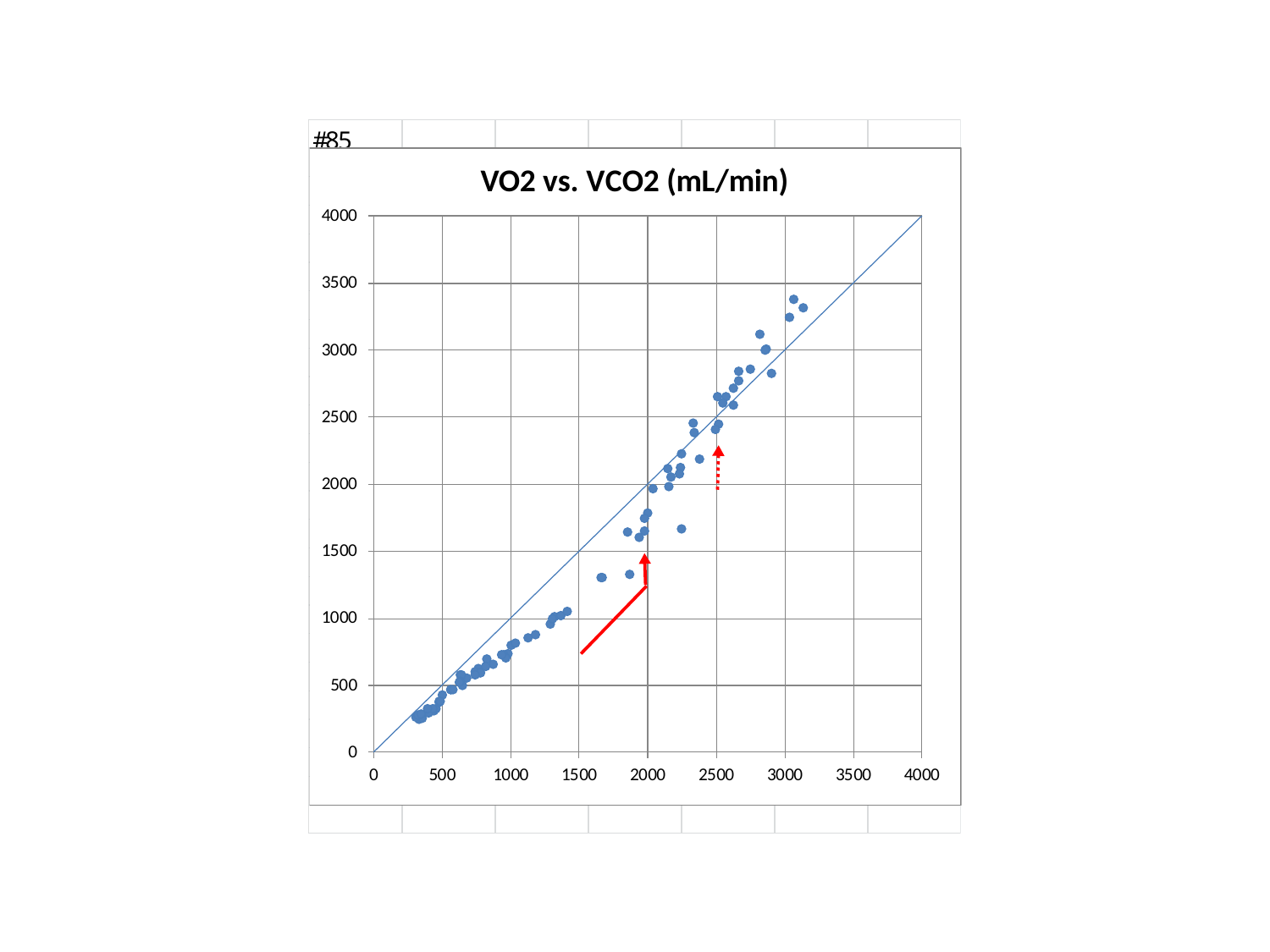

## Slide 88
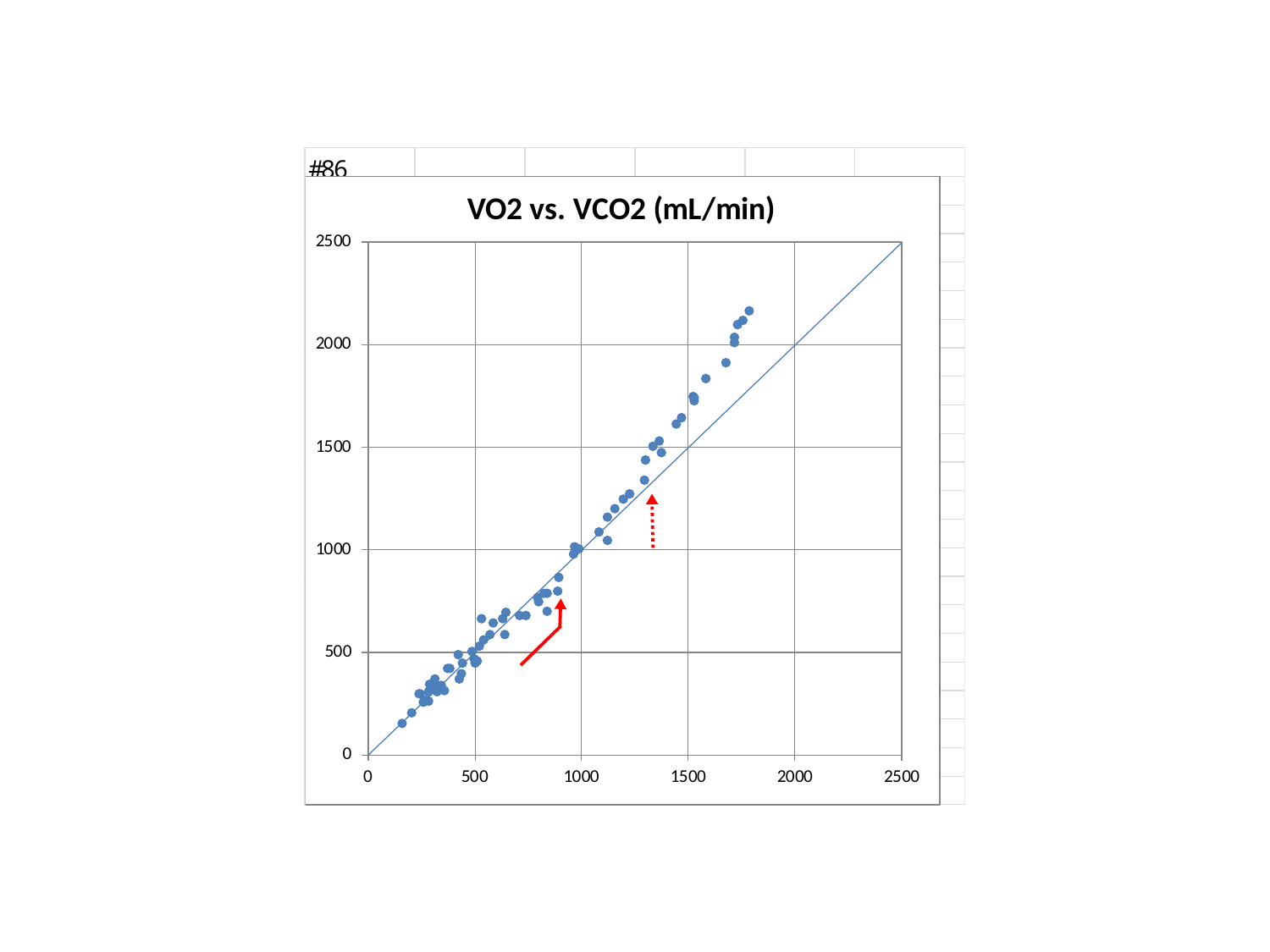

## Slide 89
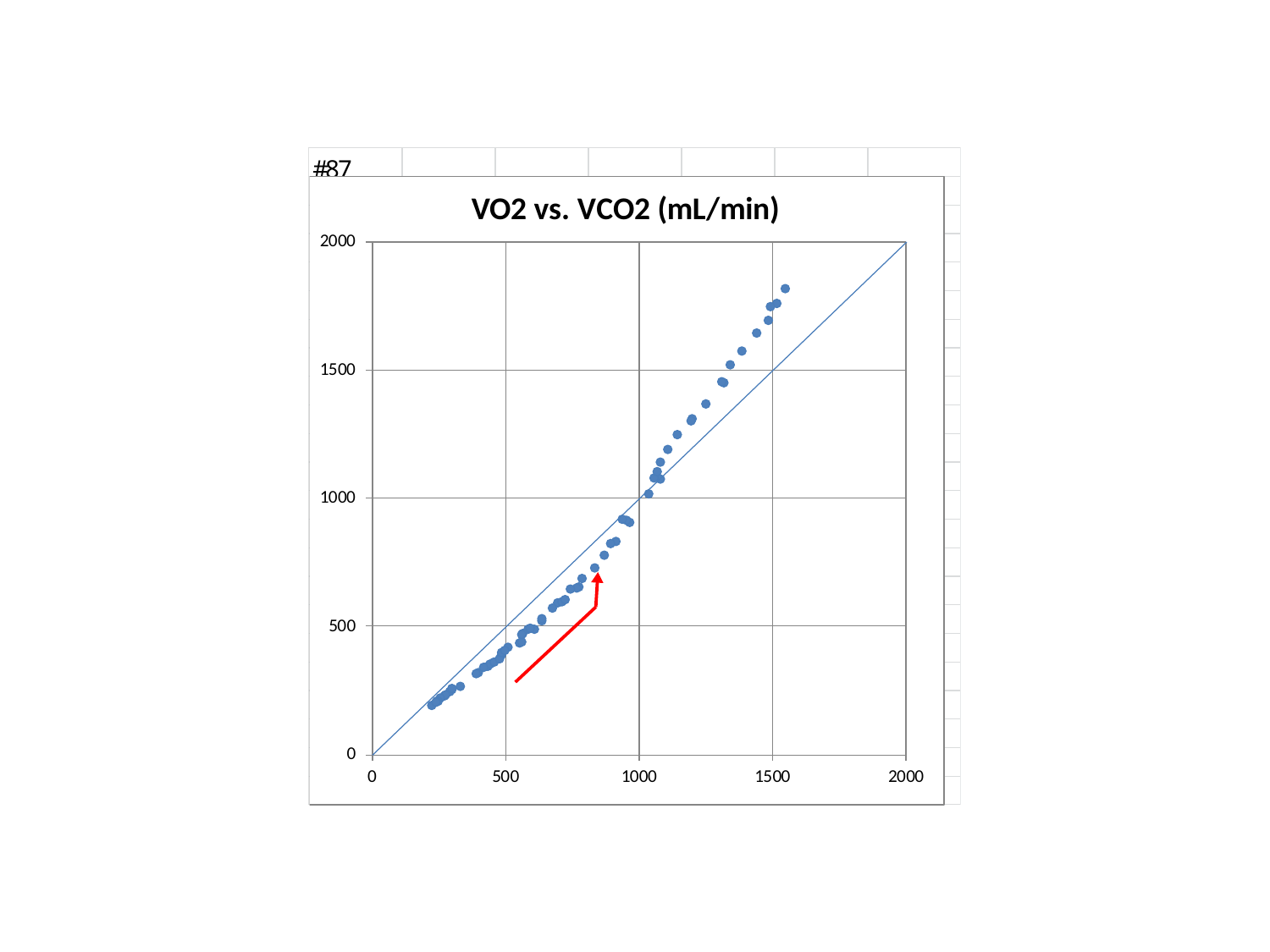

## Slide 90
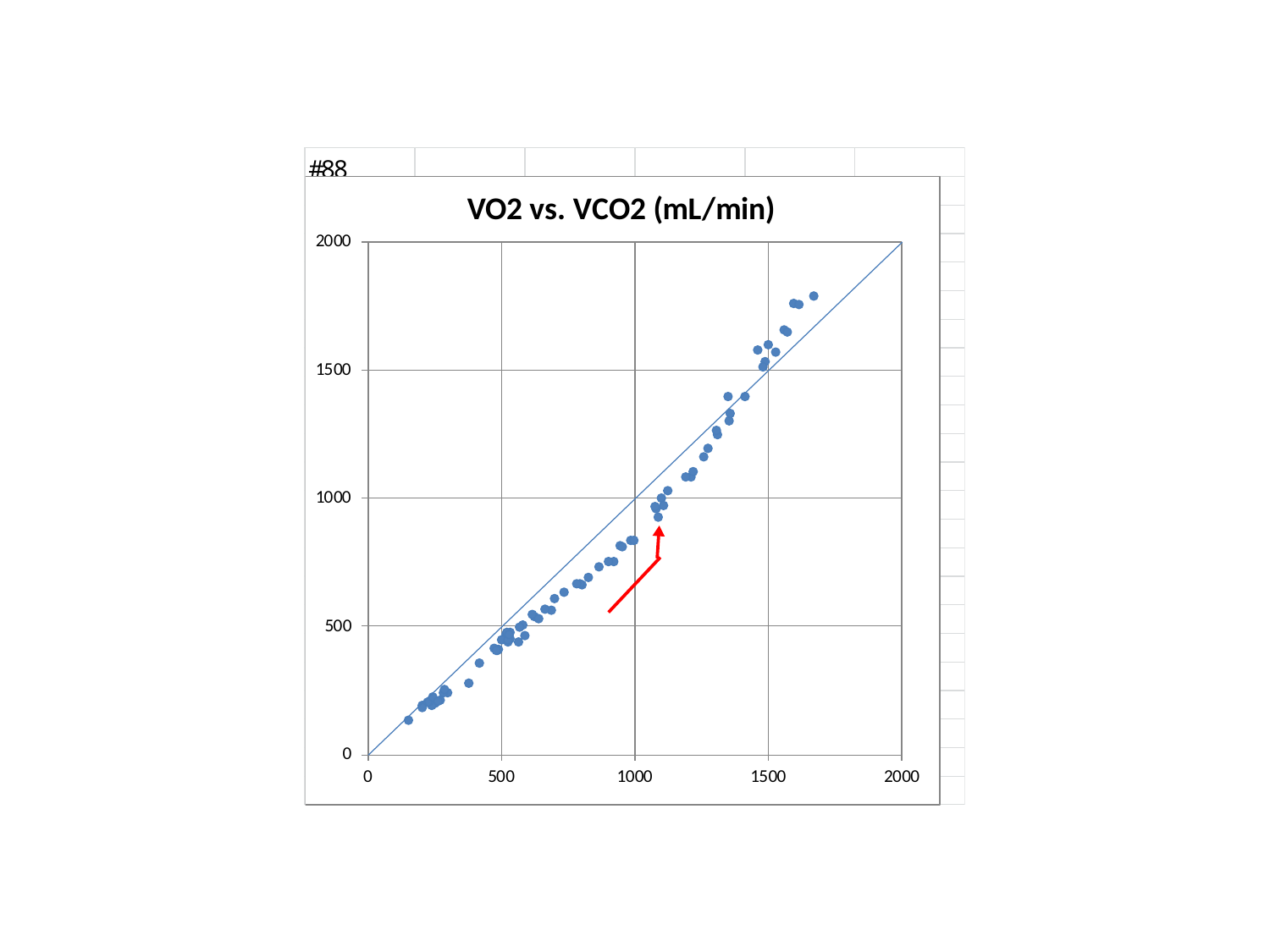

## Slide 91
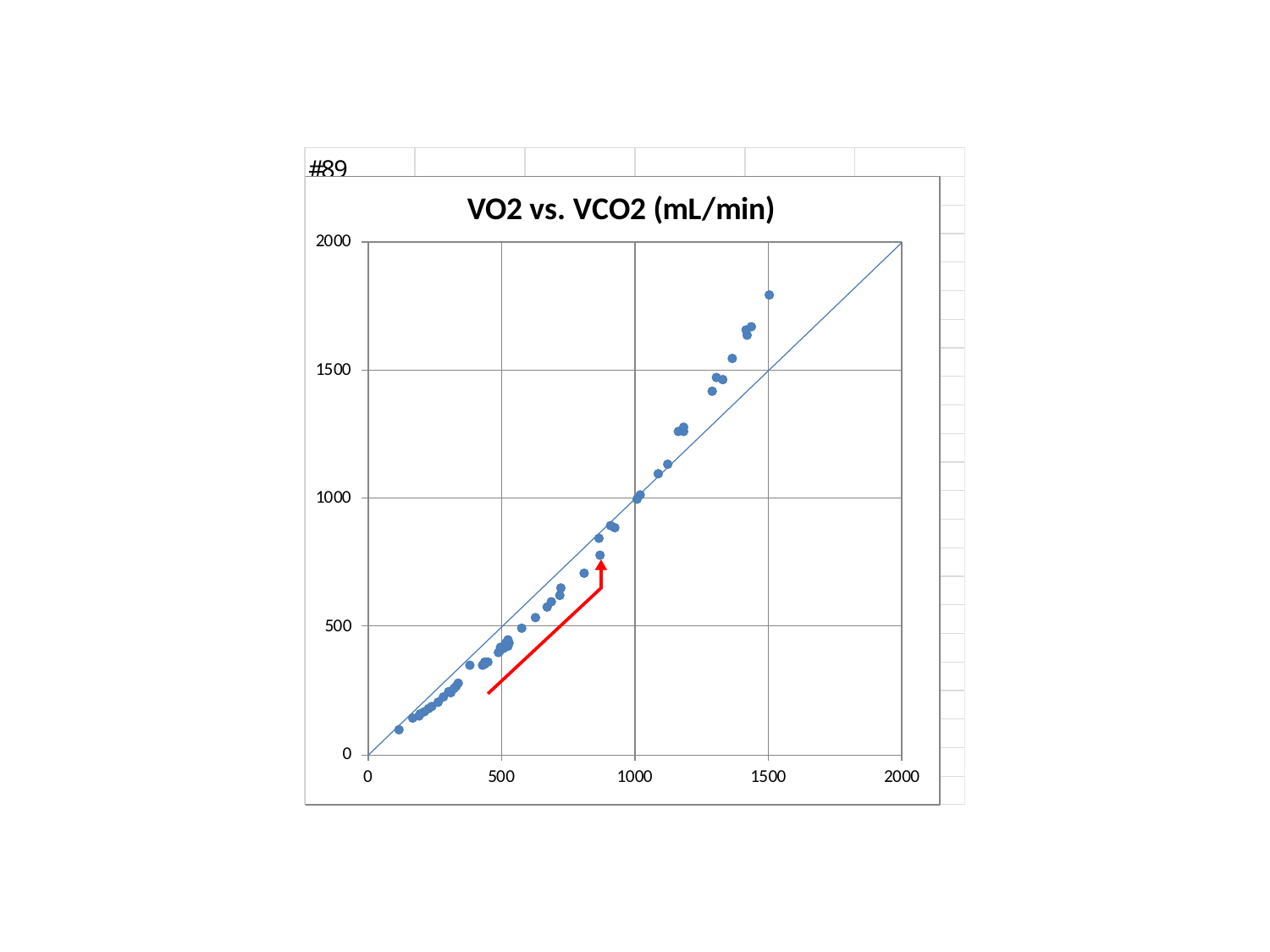

## Slide 92
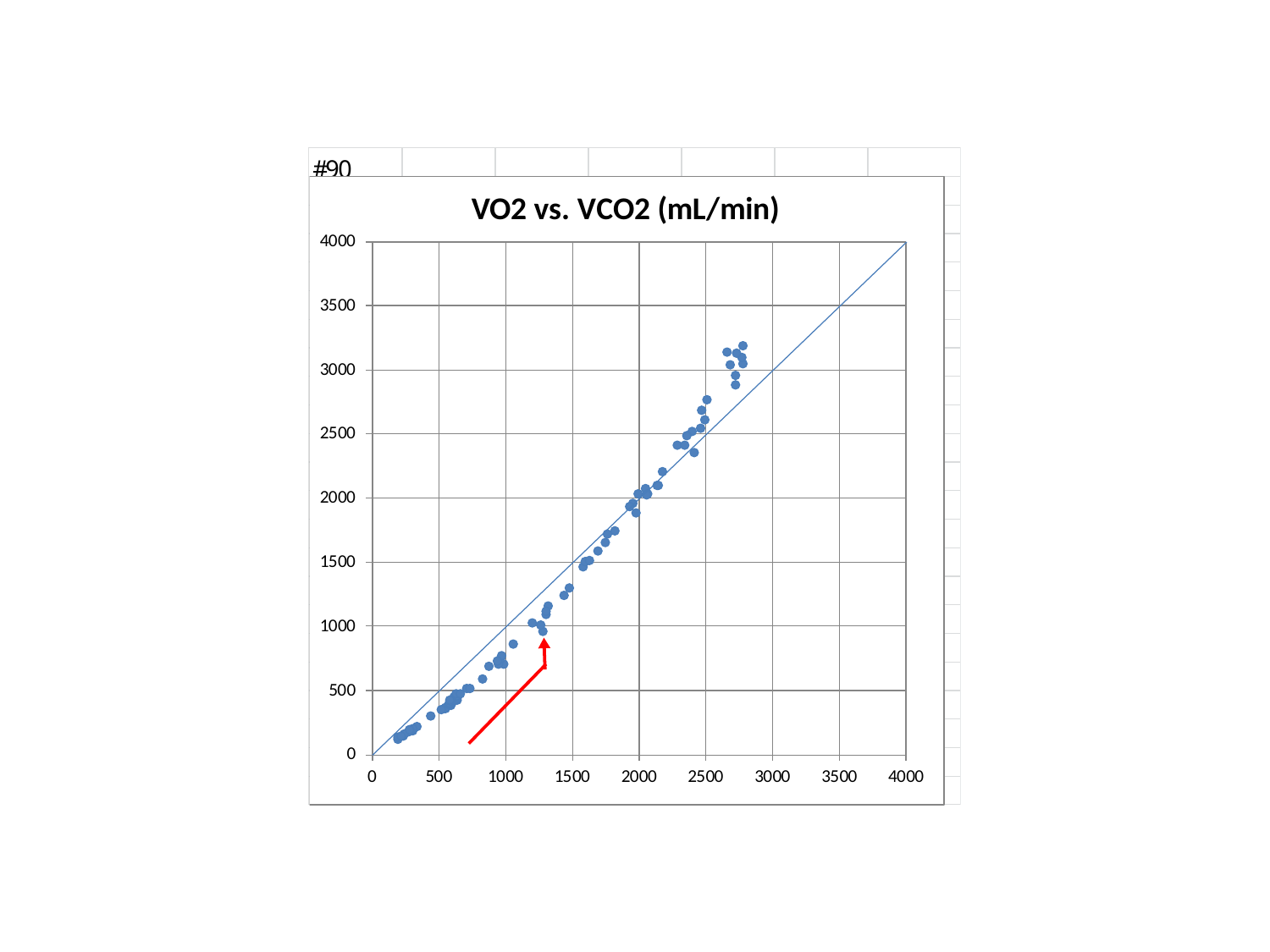

## Slide 93
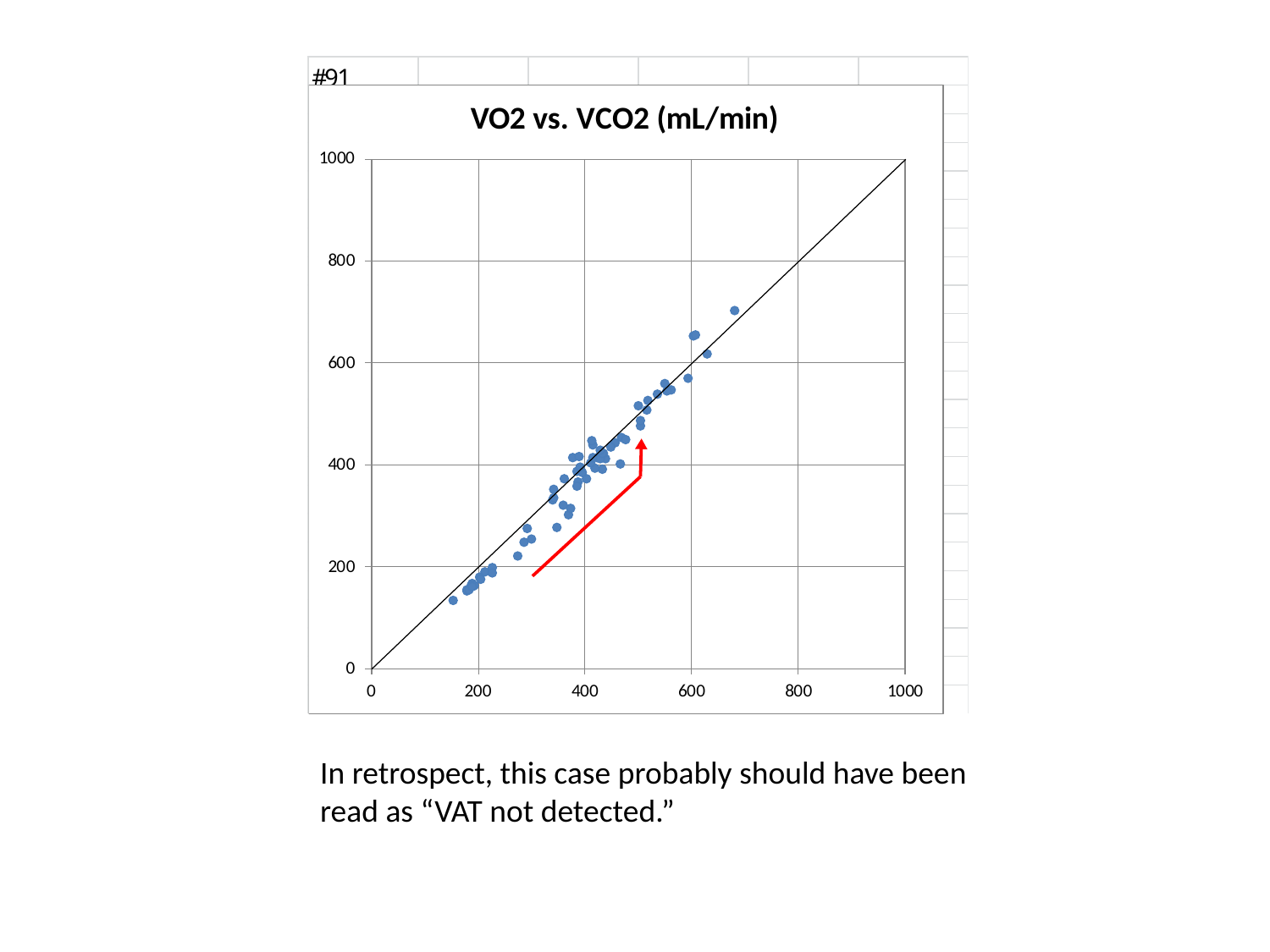

In retrospect, this case probably should have been read as “VAT not detected.”

## Slide 94
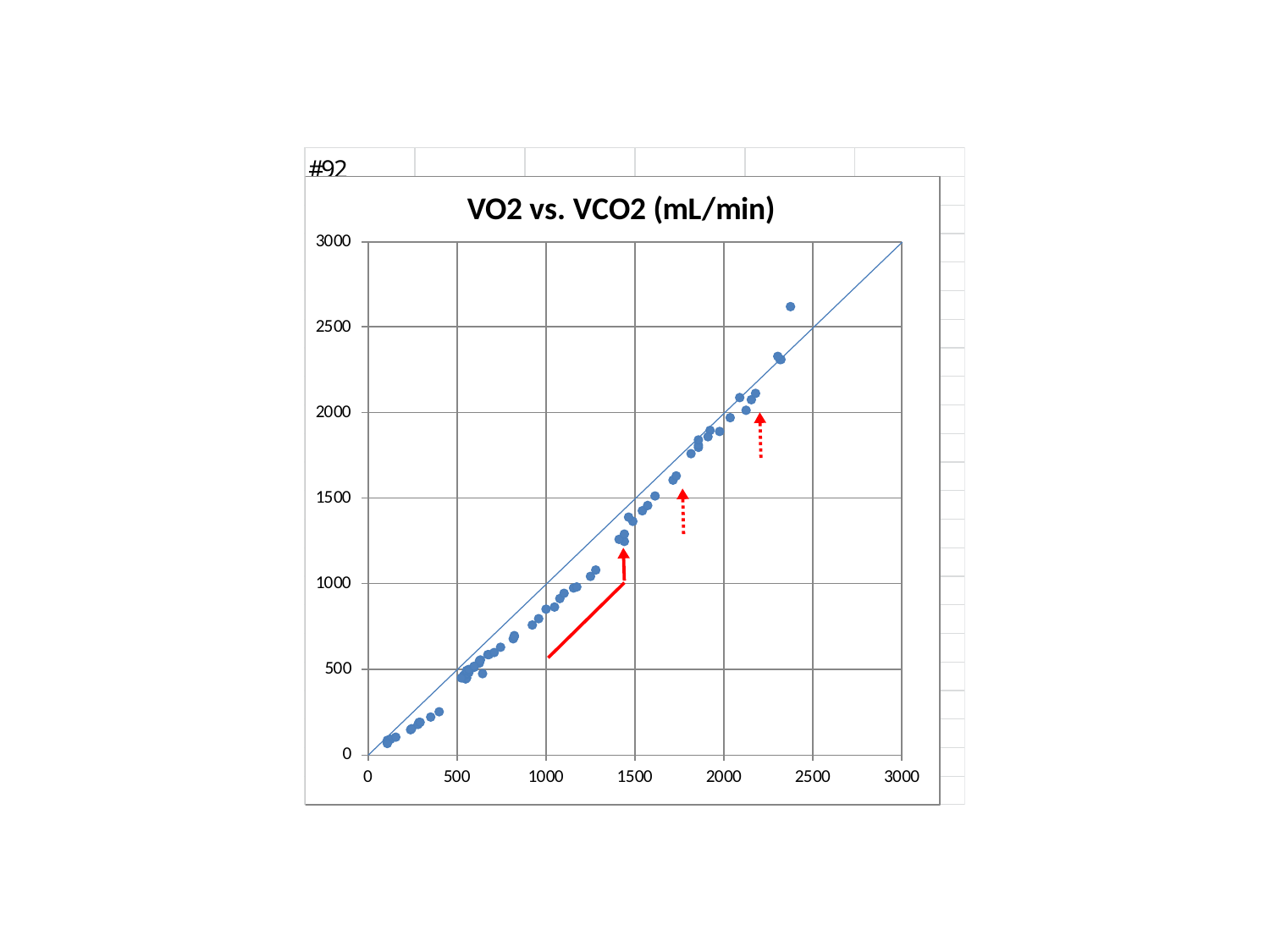

## Slide 95
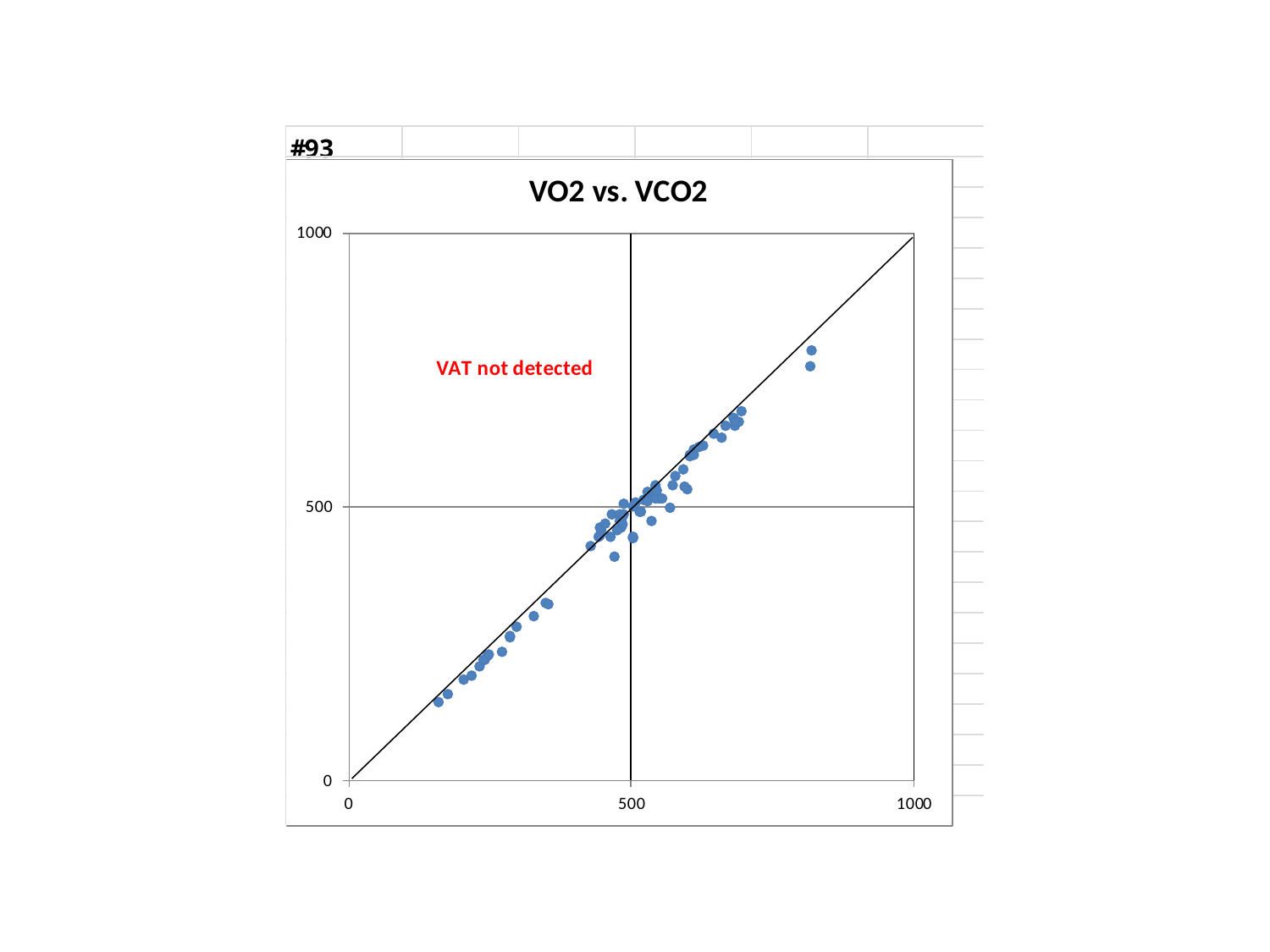

## Slide 96
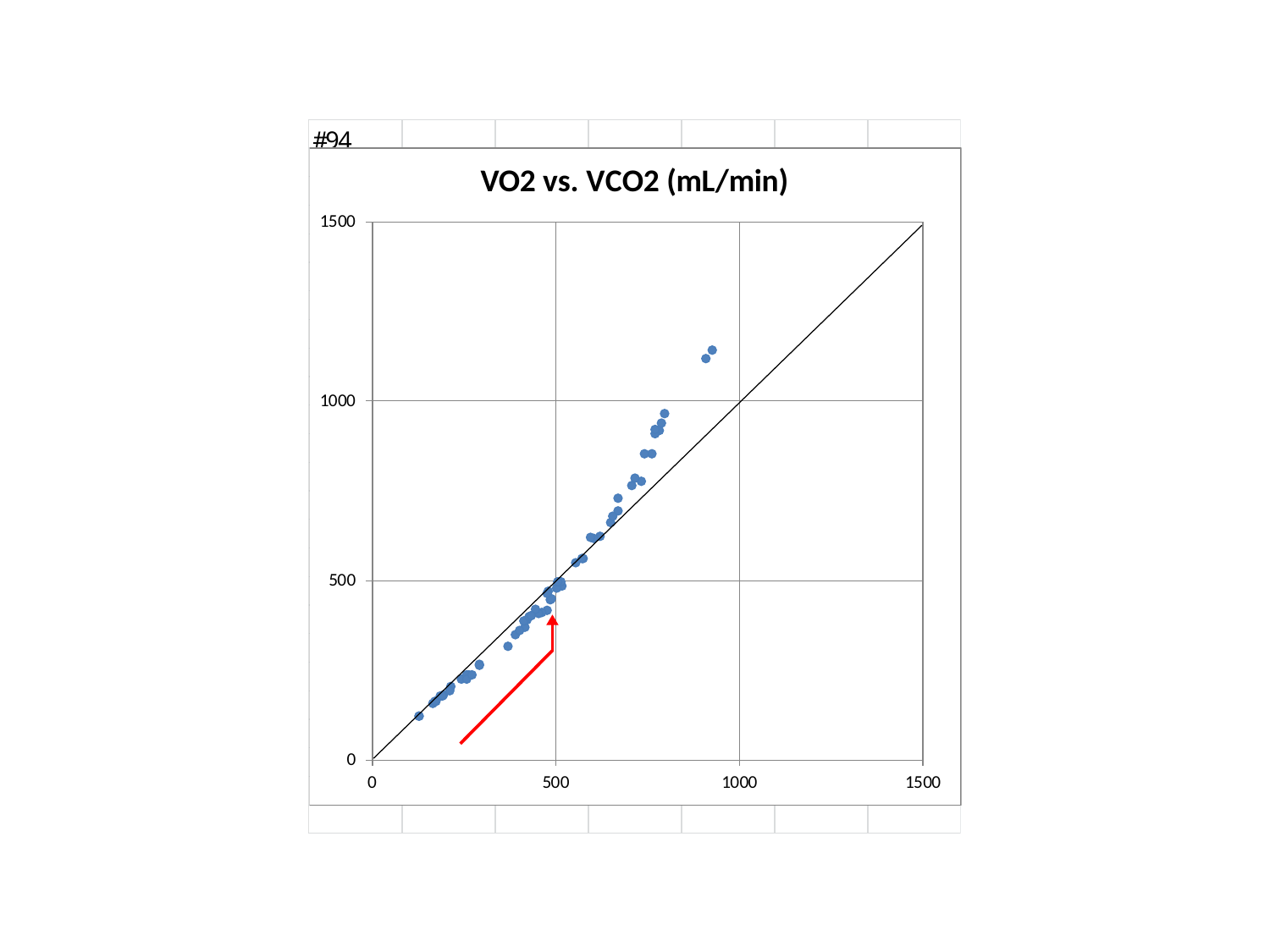

## Slide 97
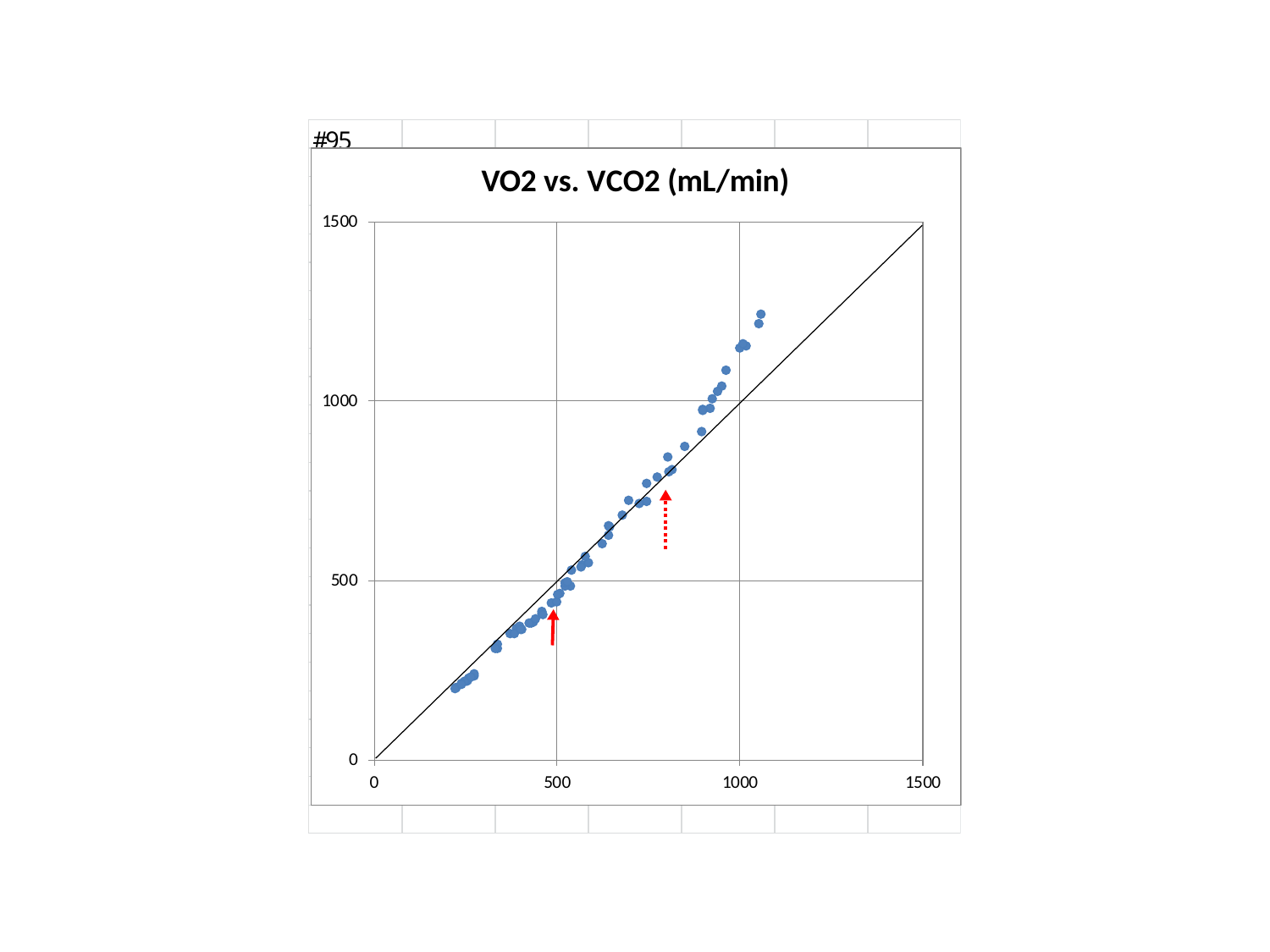

## Slide 98
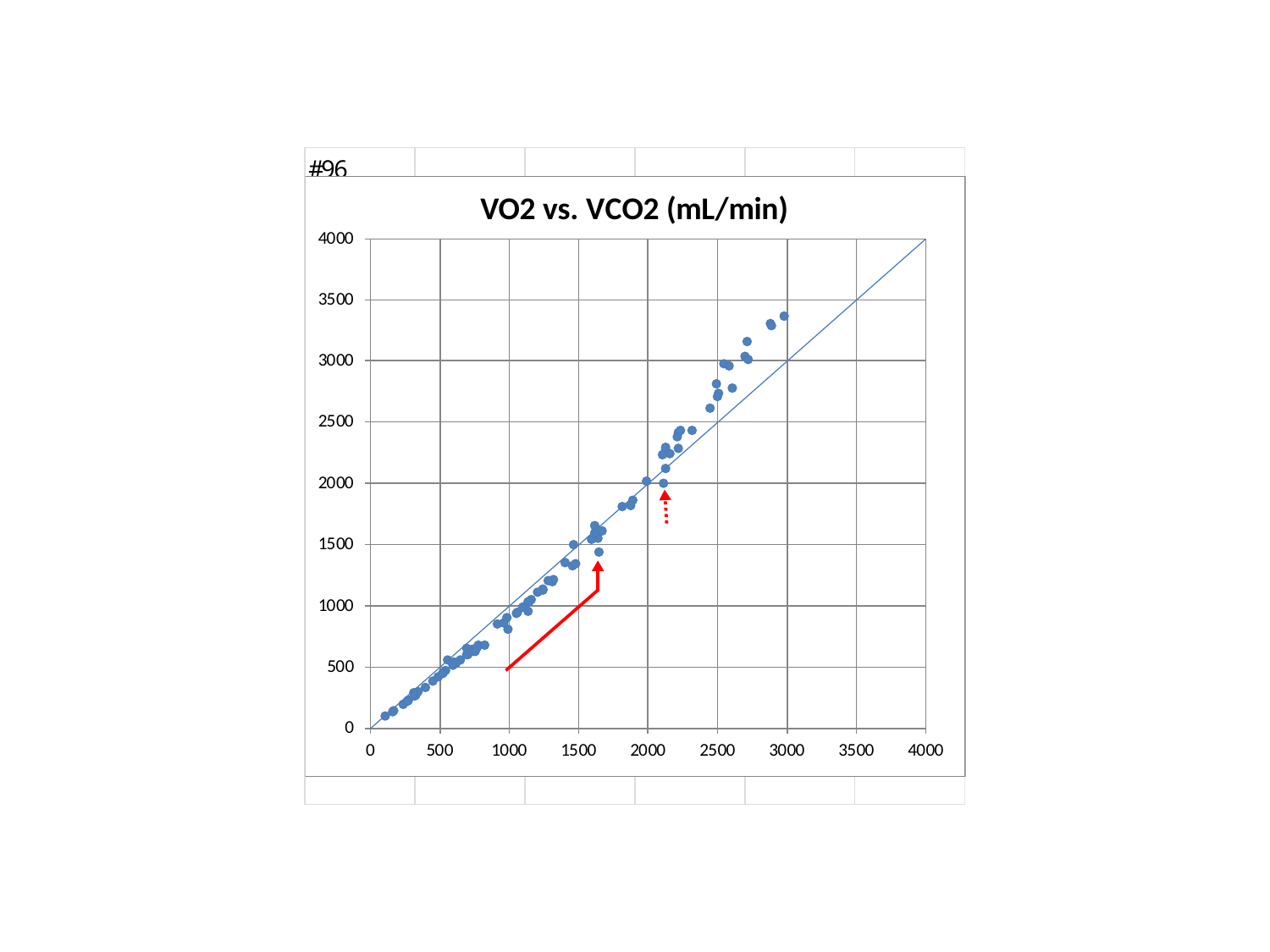

## Slide 99
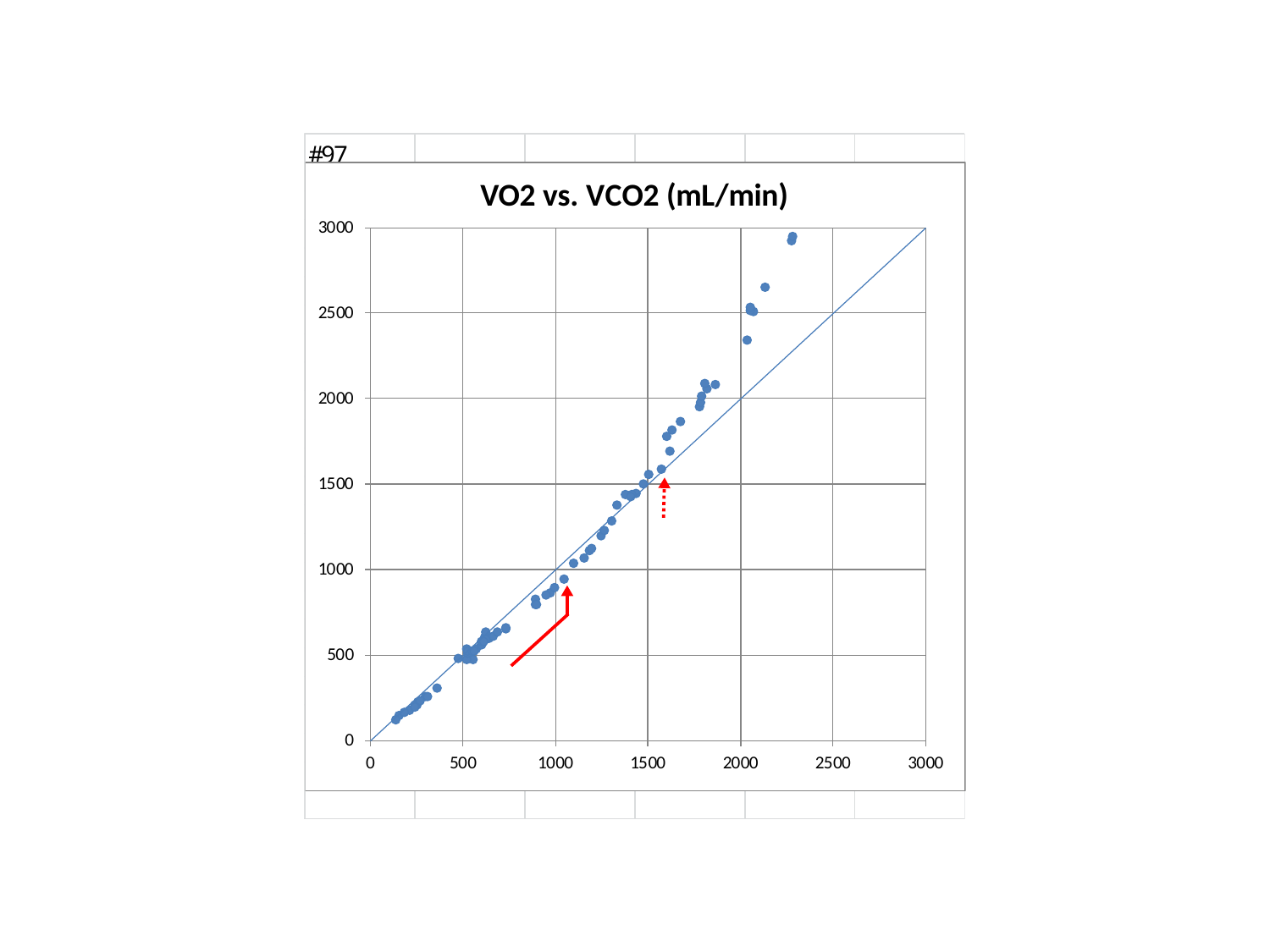

## Slide 100
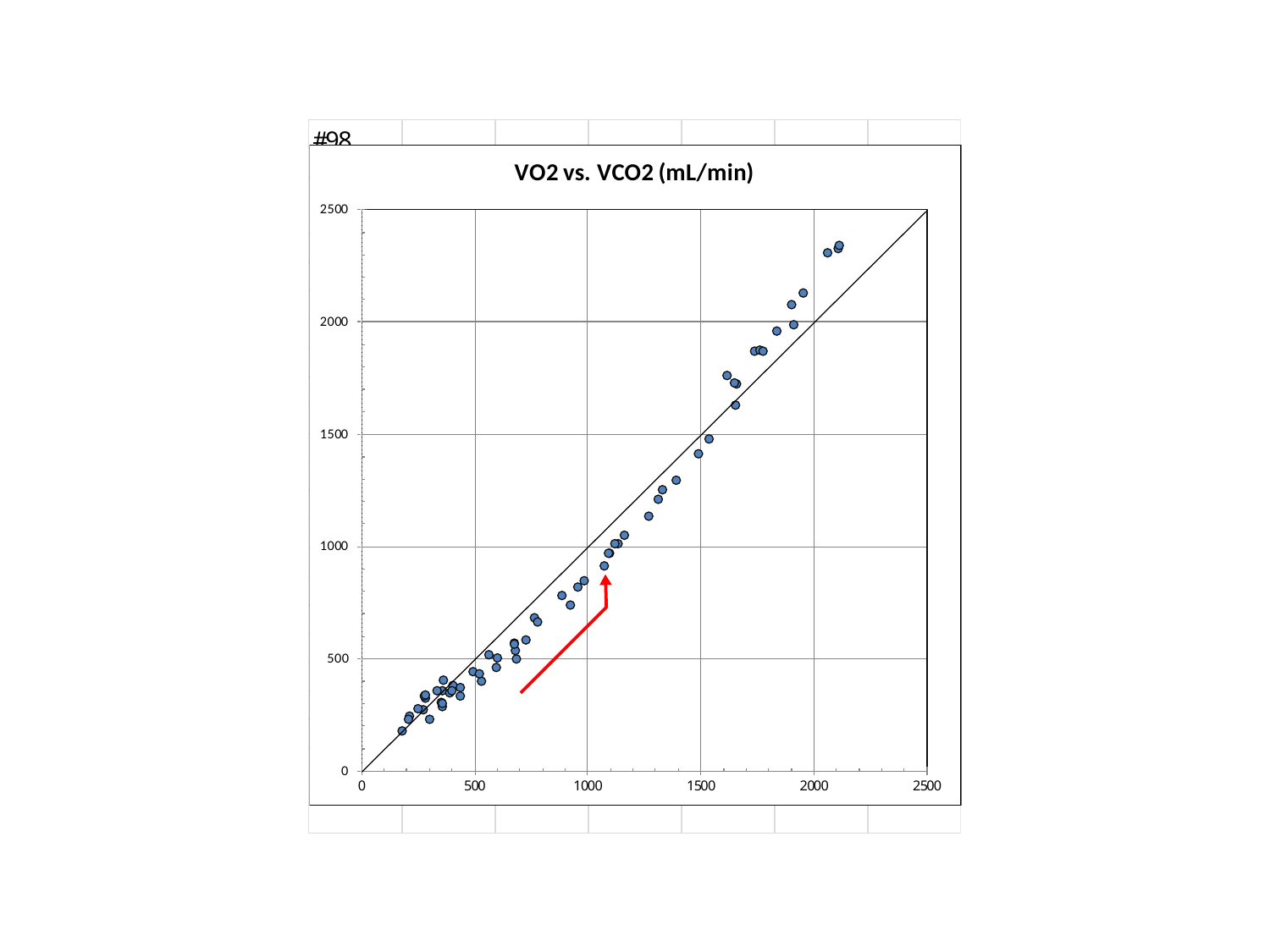

## Slide 101
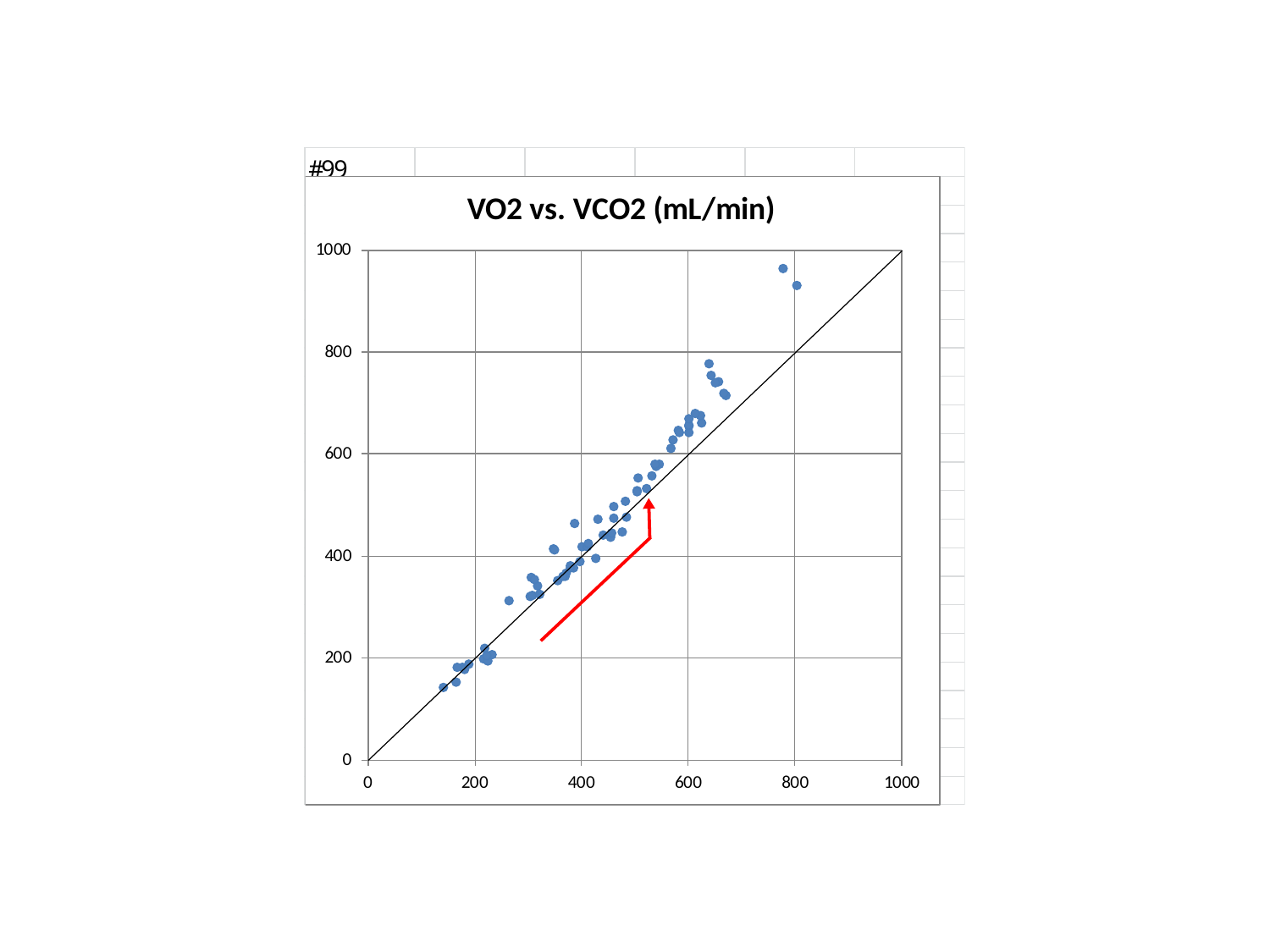

## Slide 102
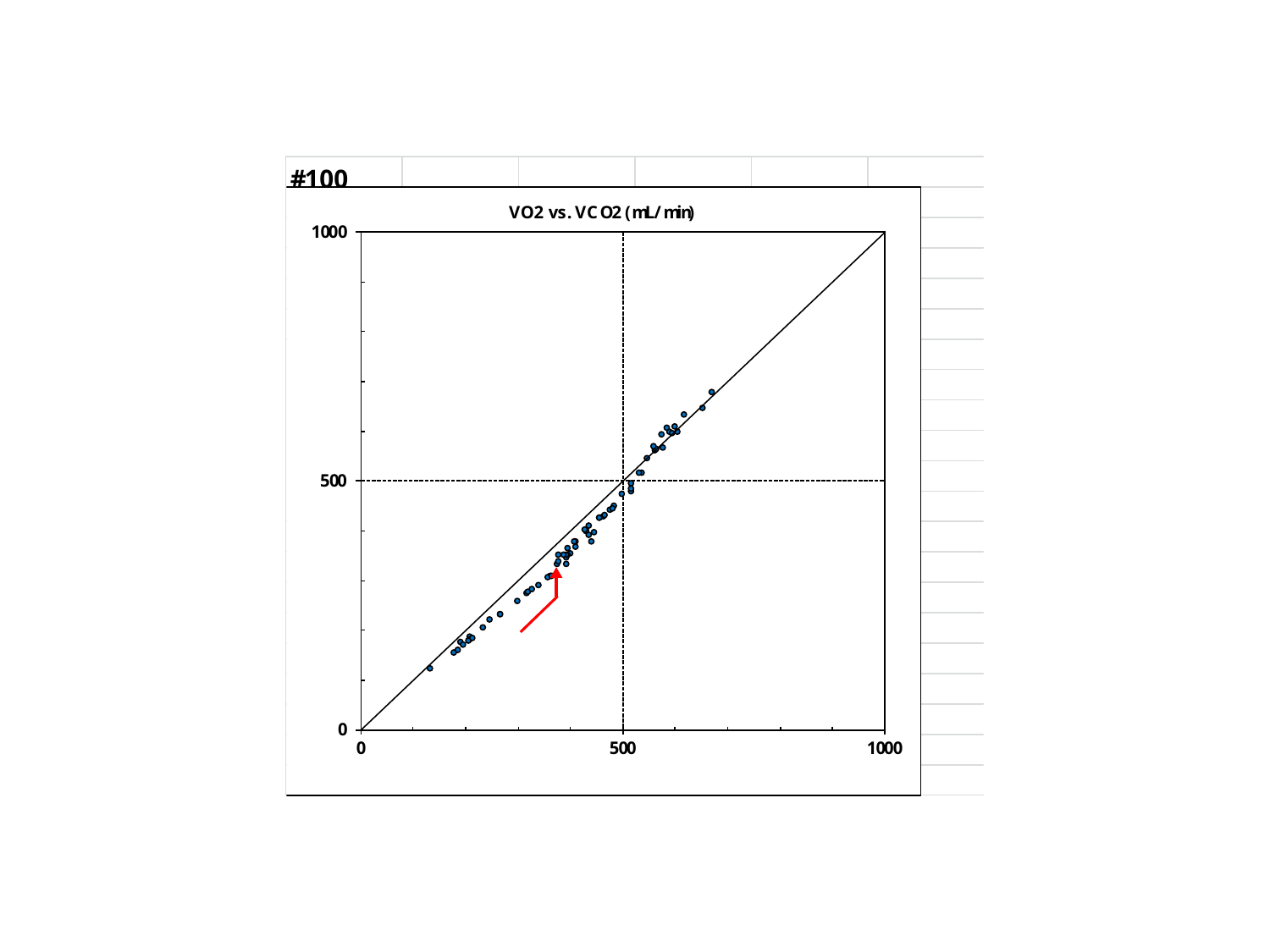

## Slide 103
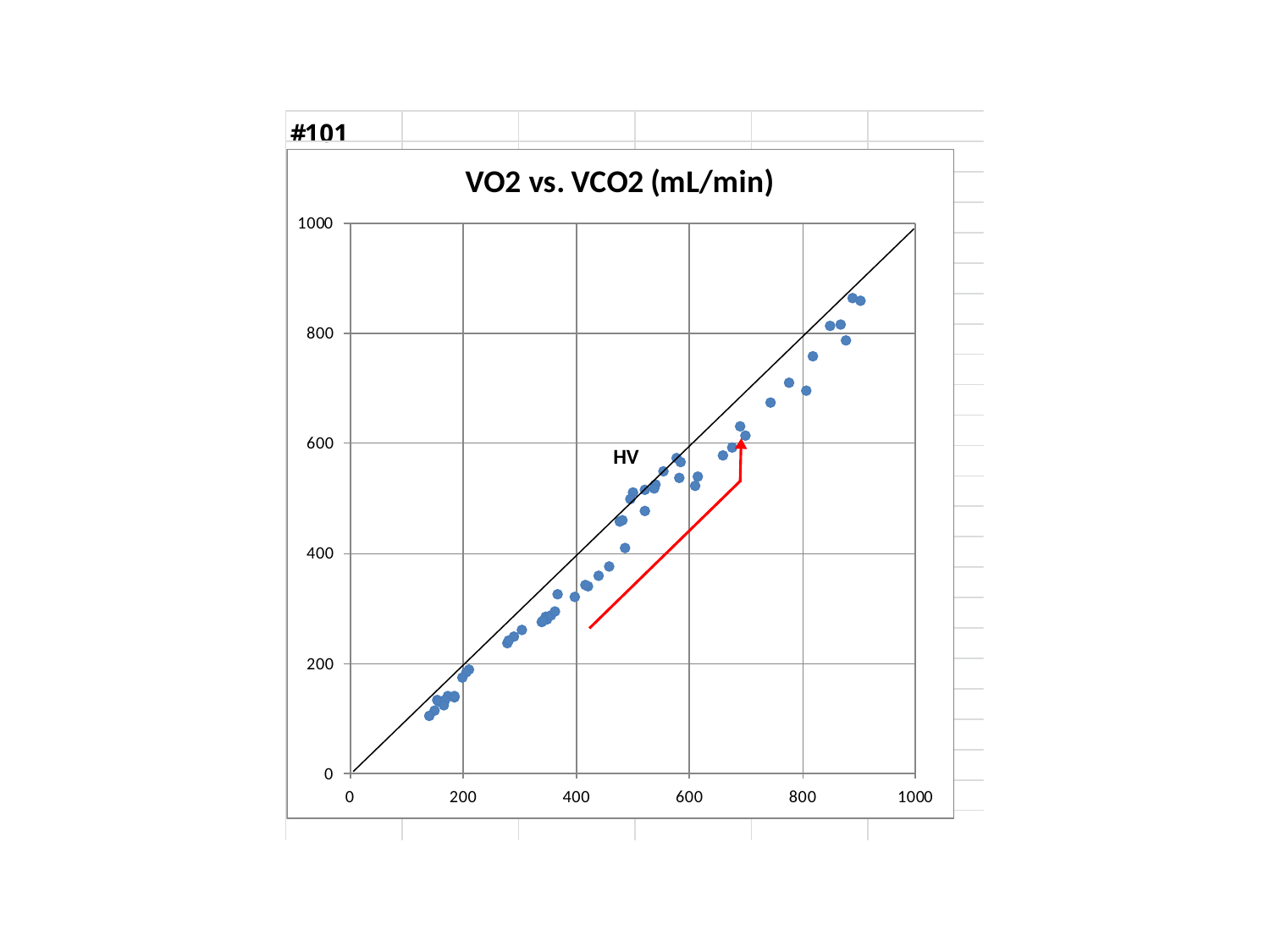

## Slide 104
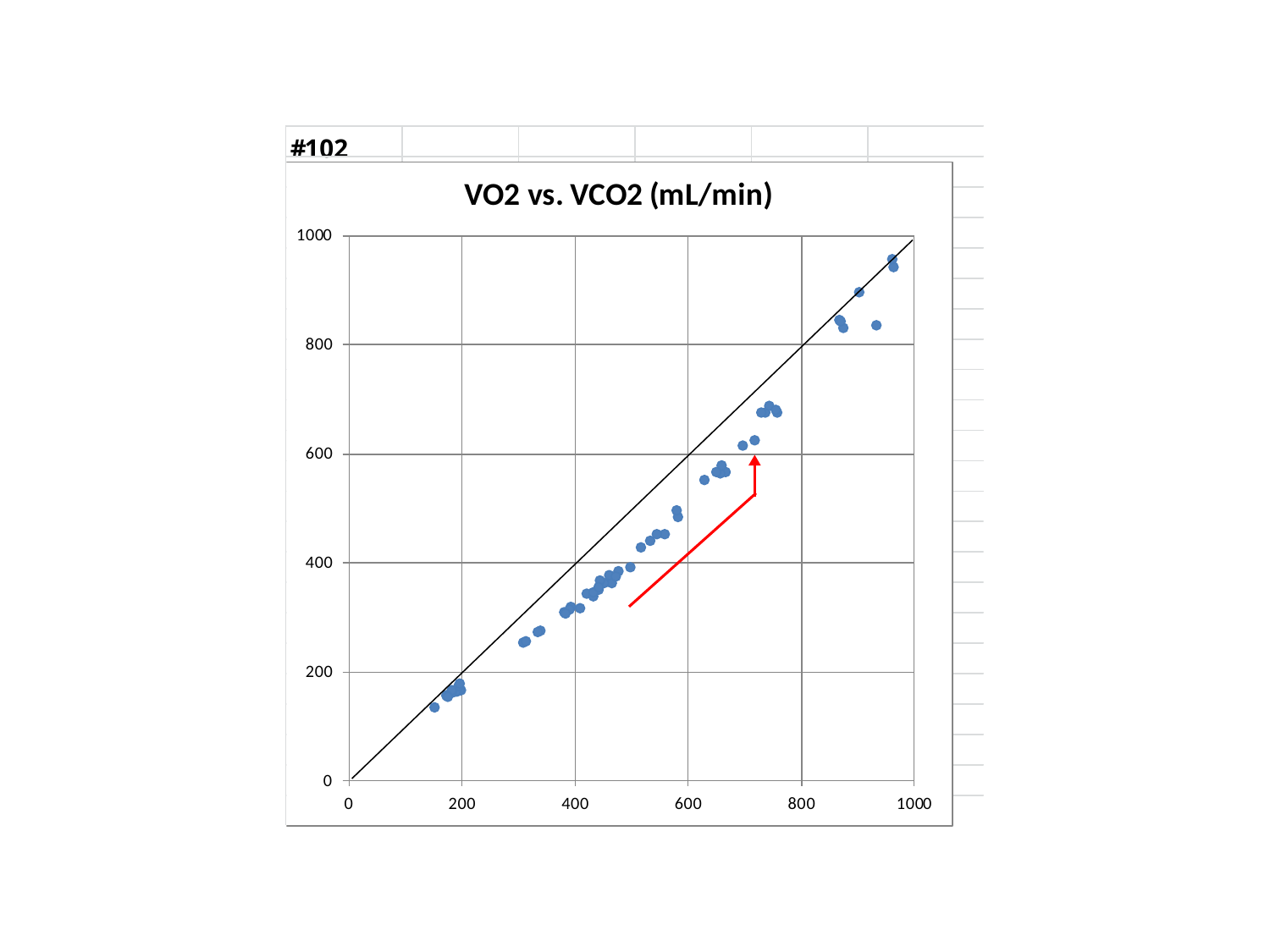

## Slide 105
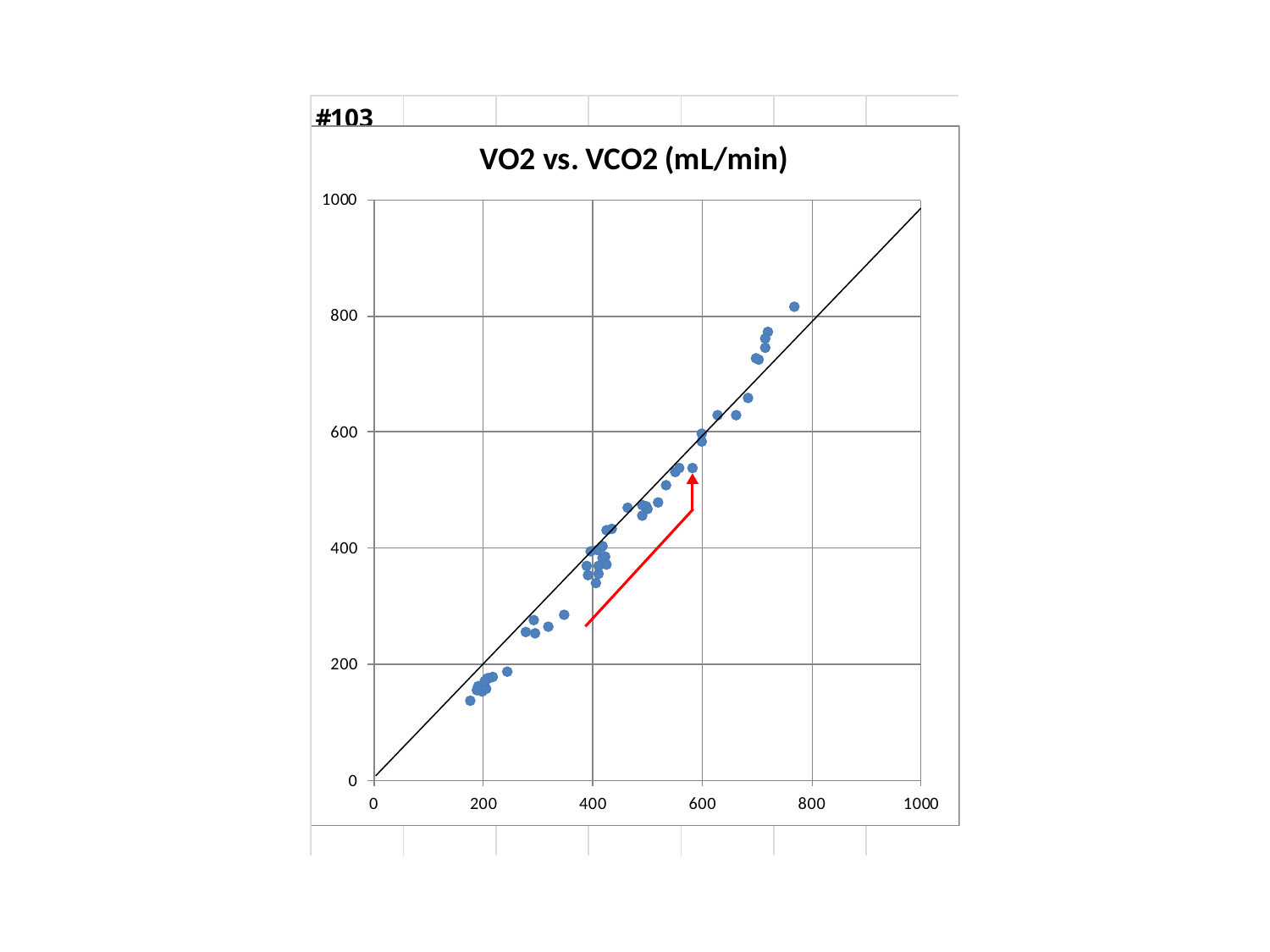

## Slide 106
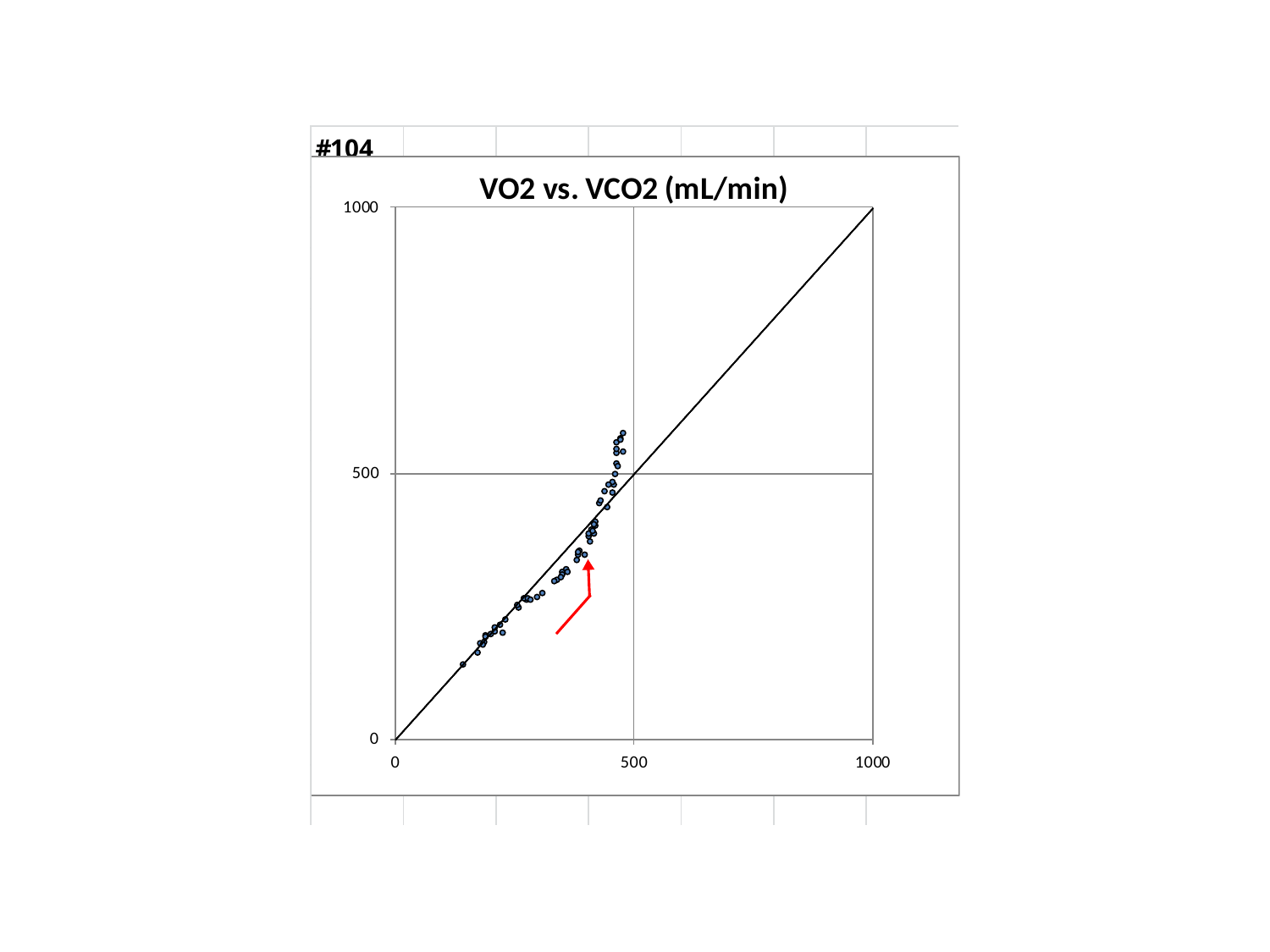

## Slide 107
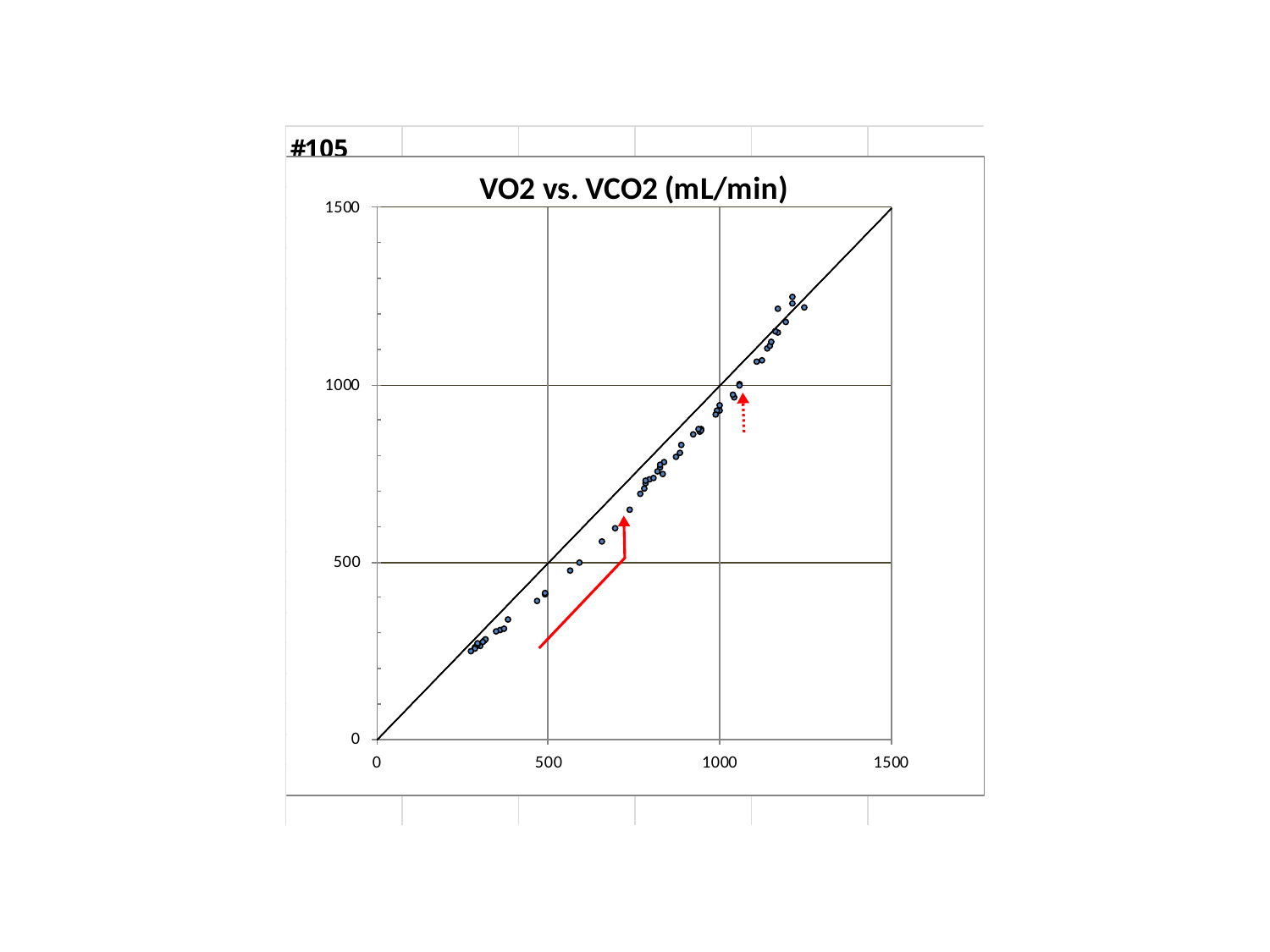

## Slide 108
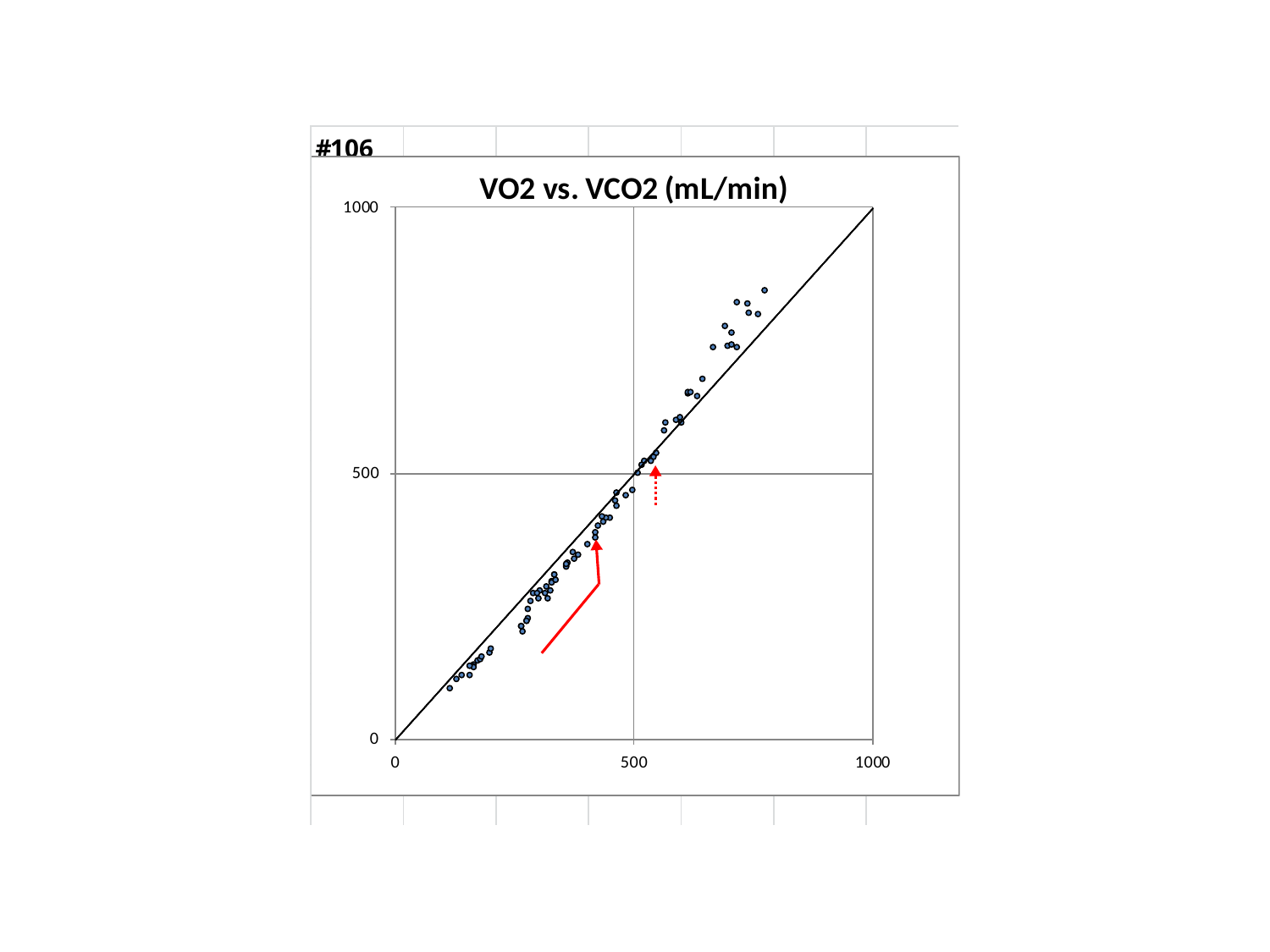

## Slide 109
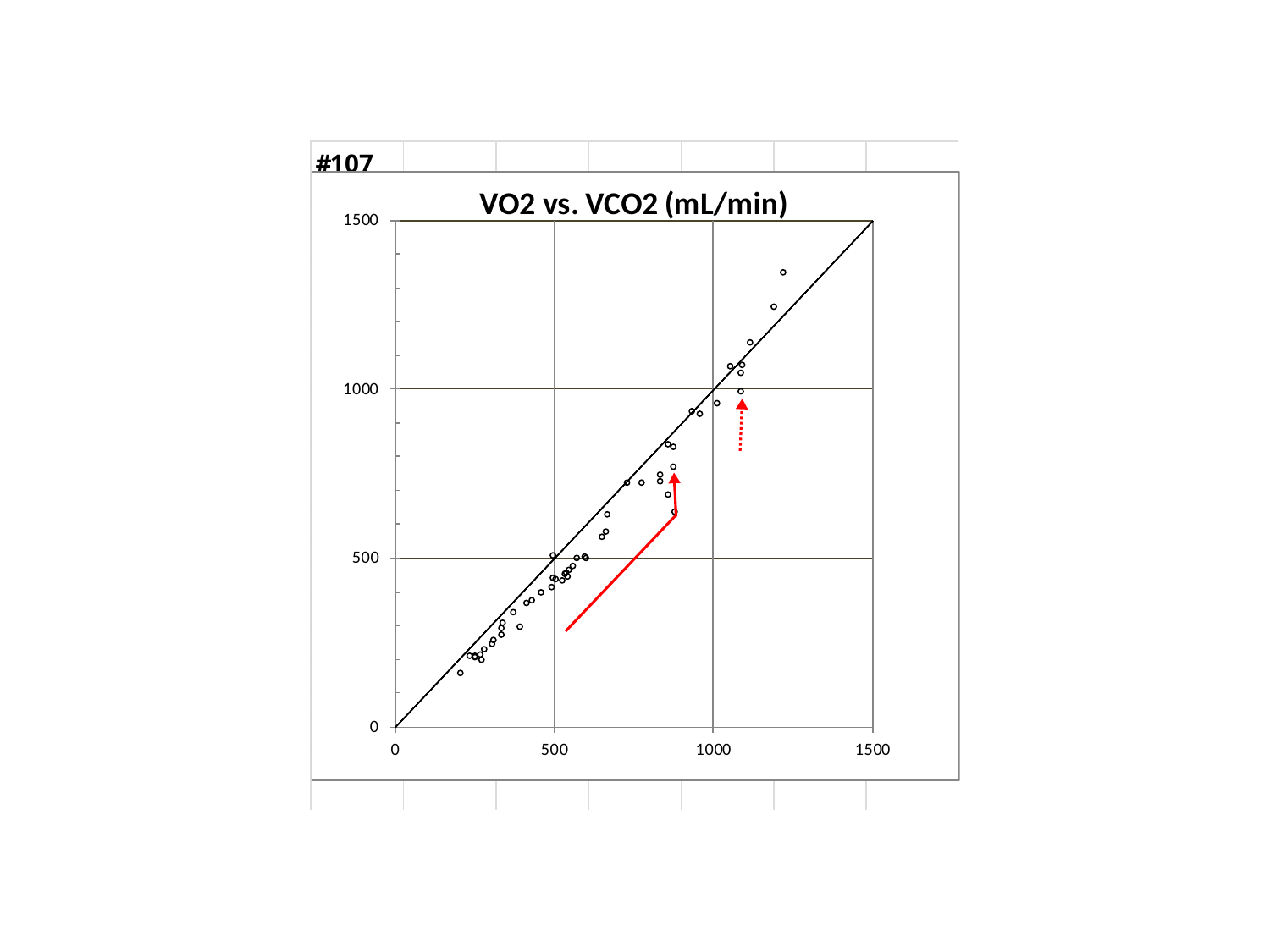

## Slide 110
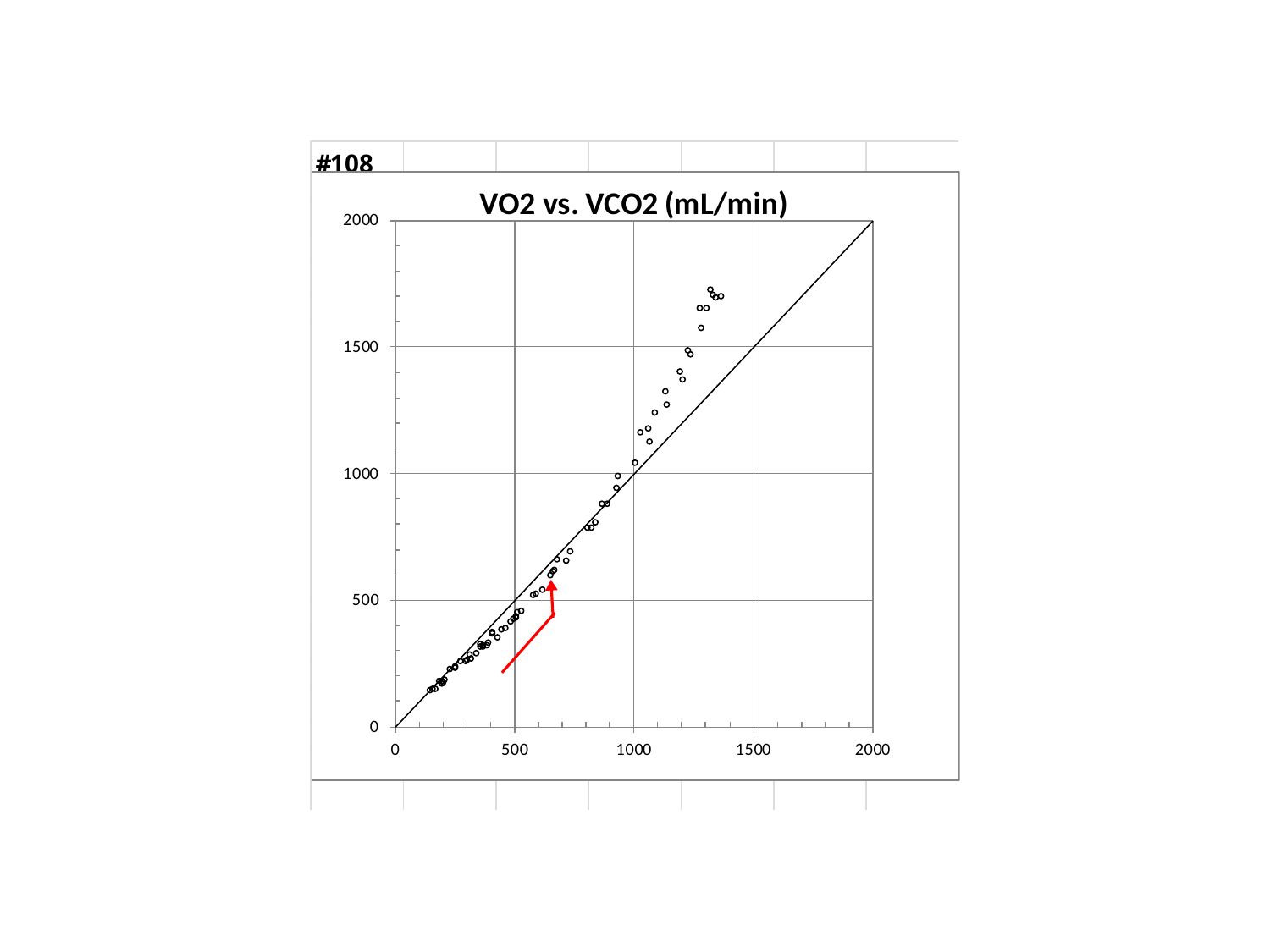

## Slide 111
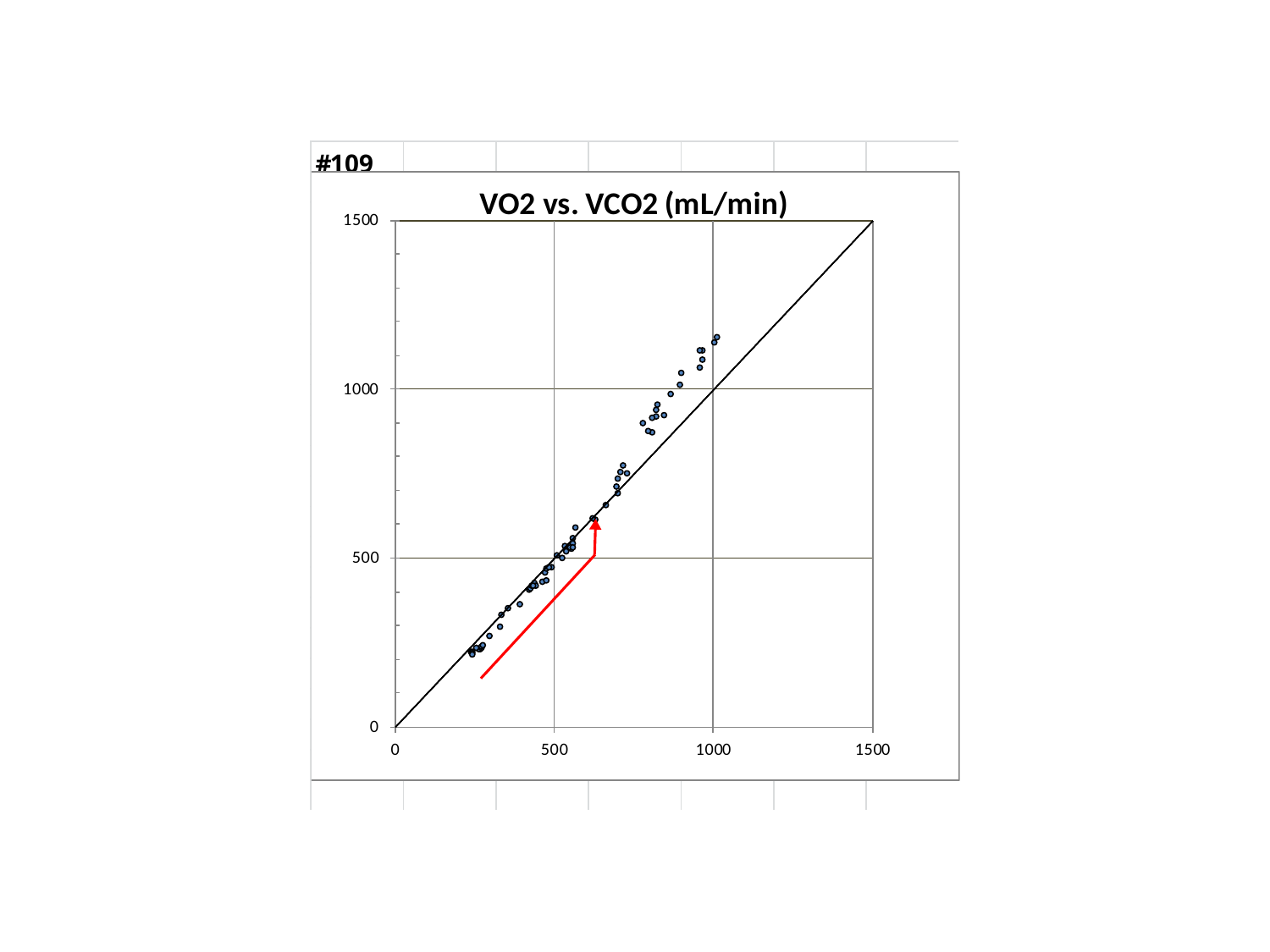

## Slide 112
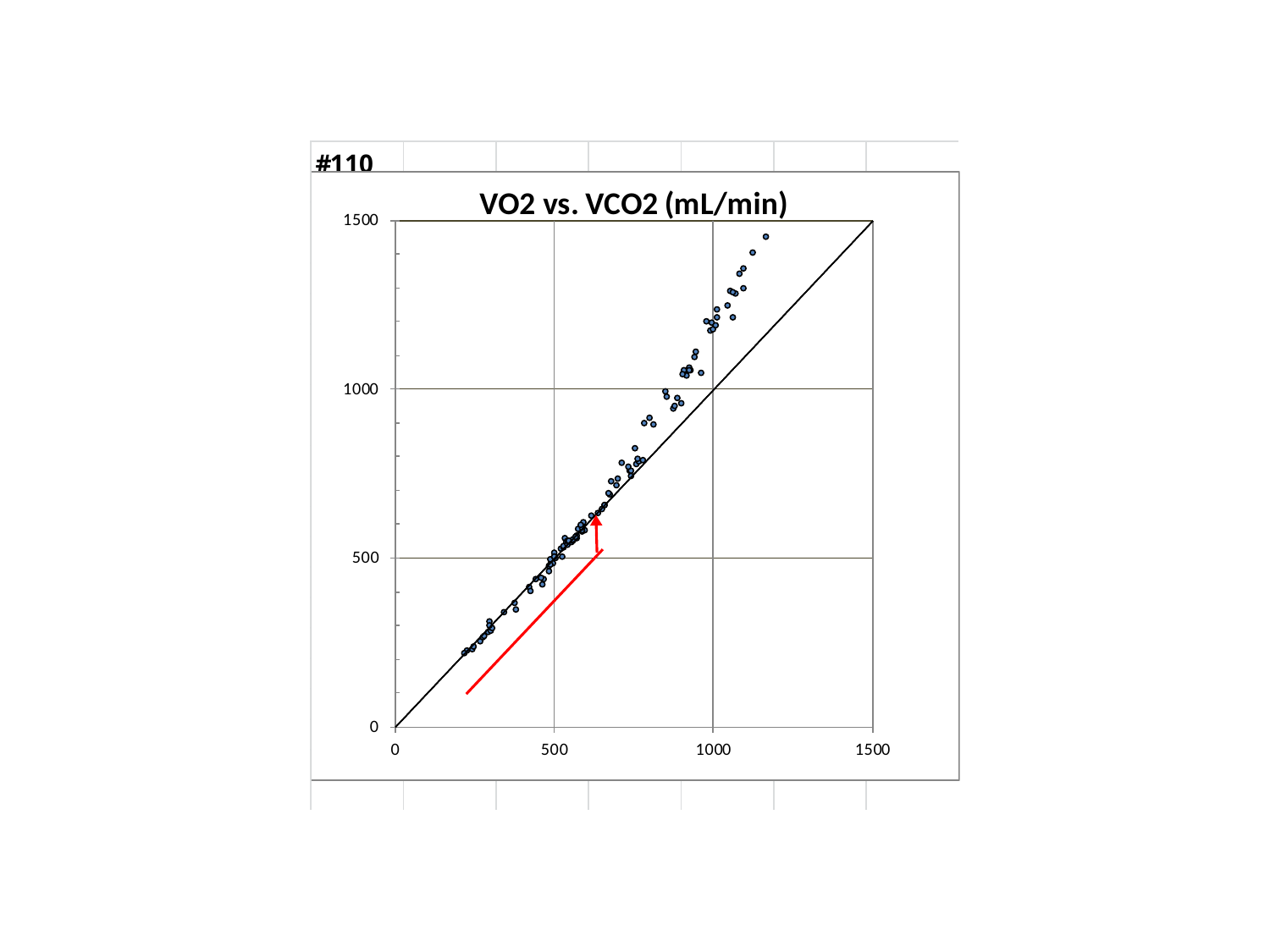

## Slide 113
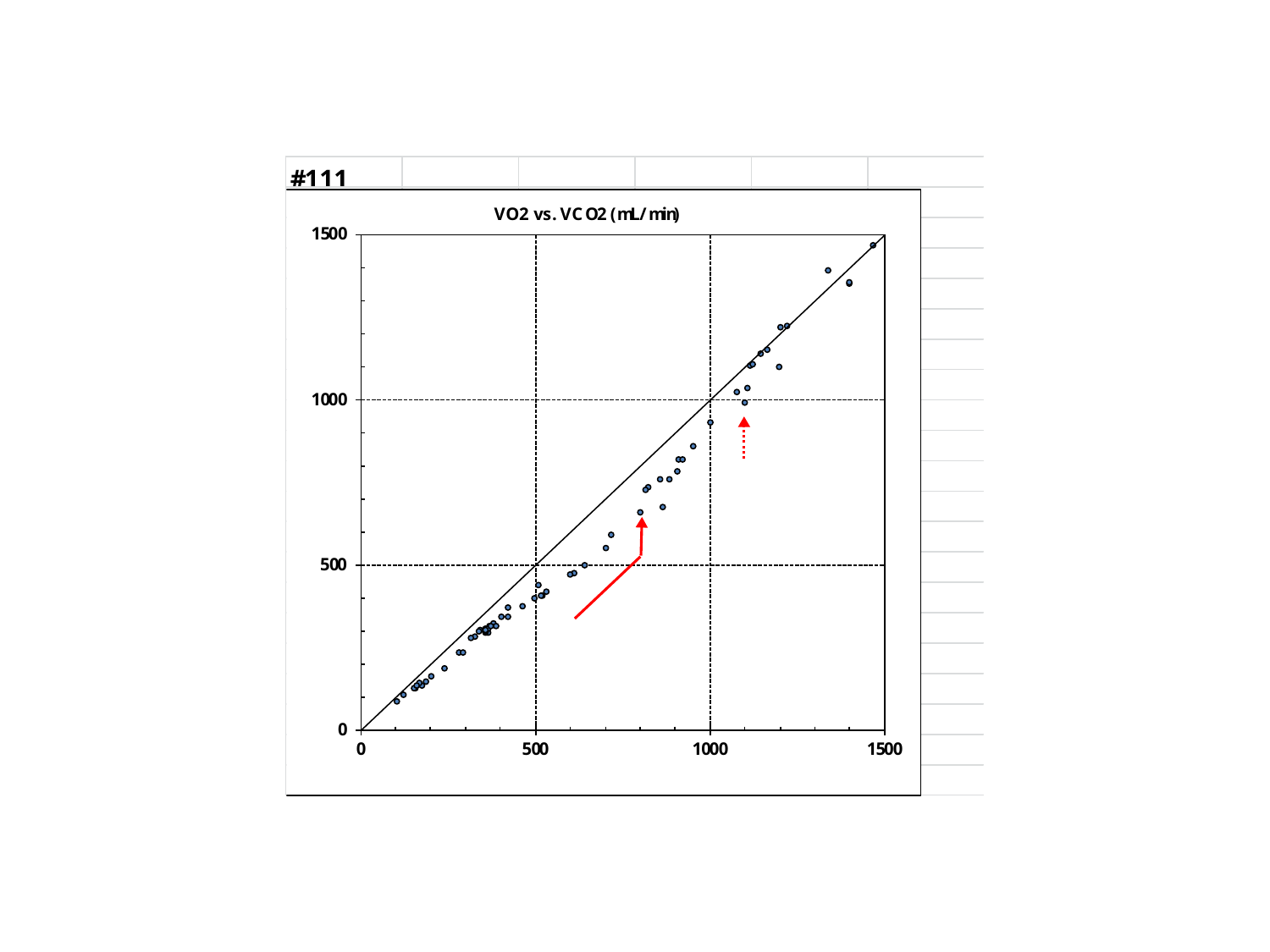

## Slide 114
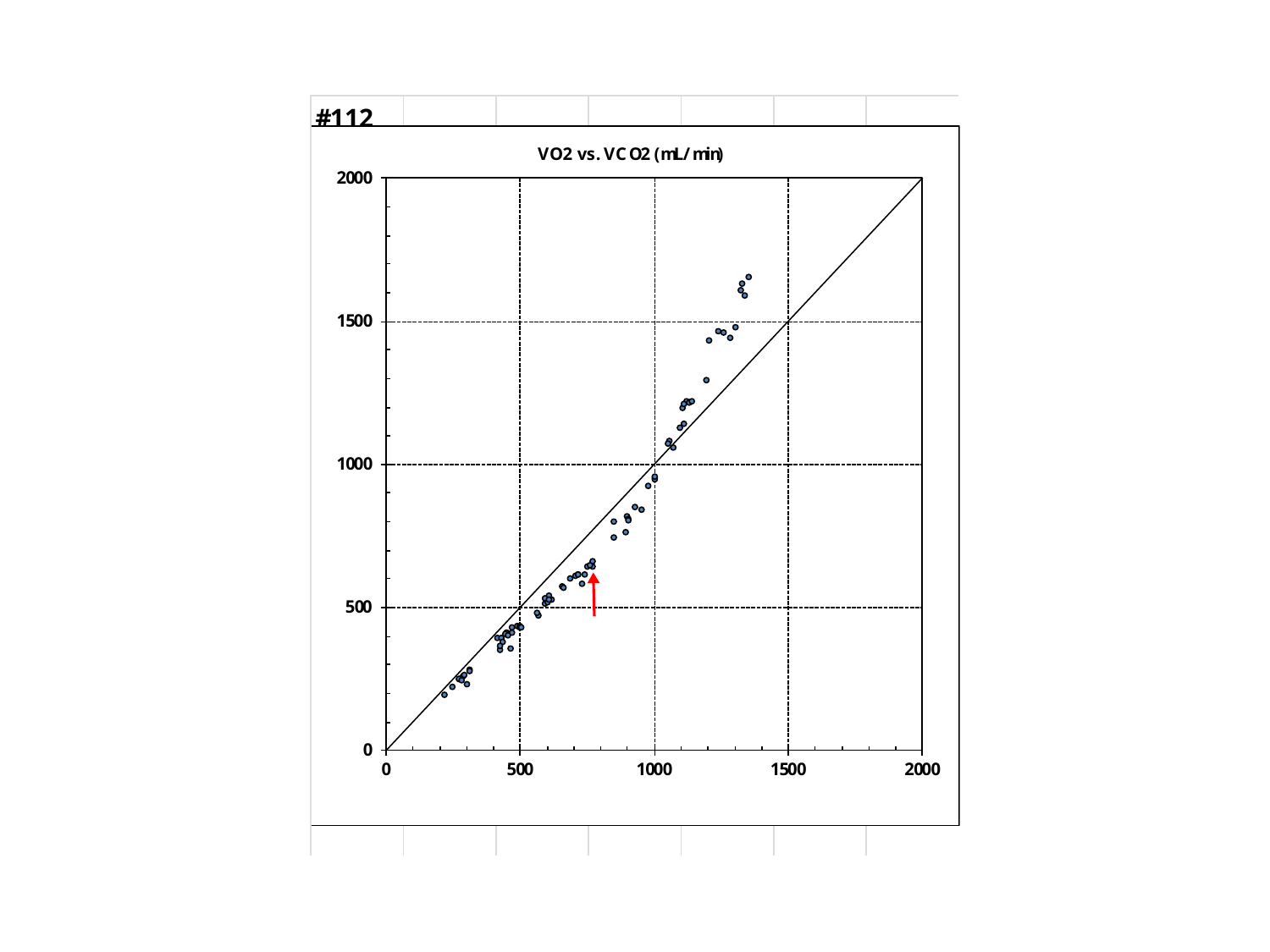

## Slide 115
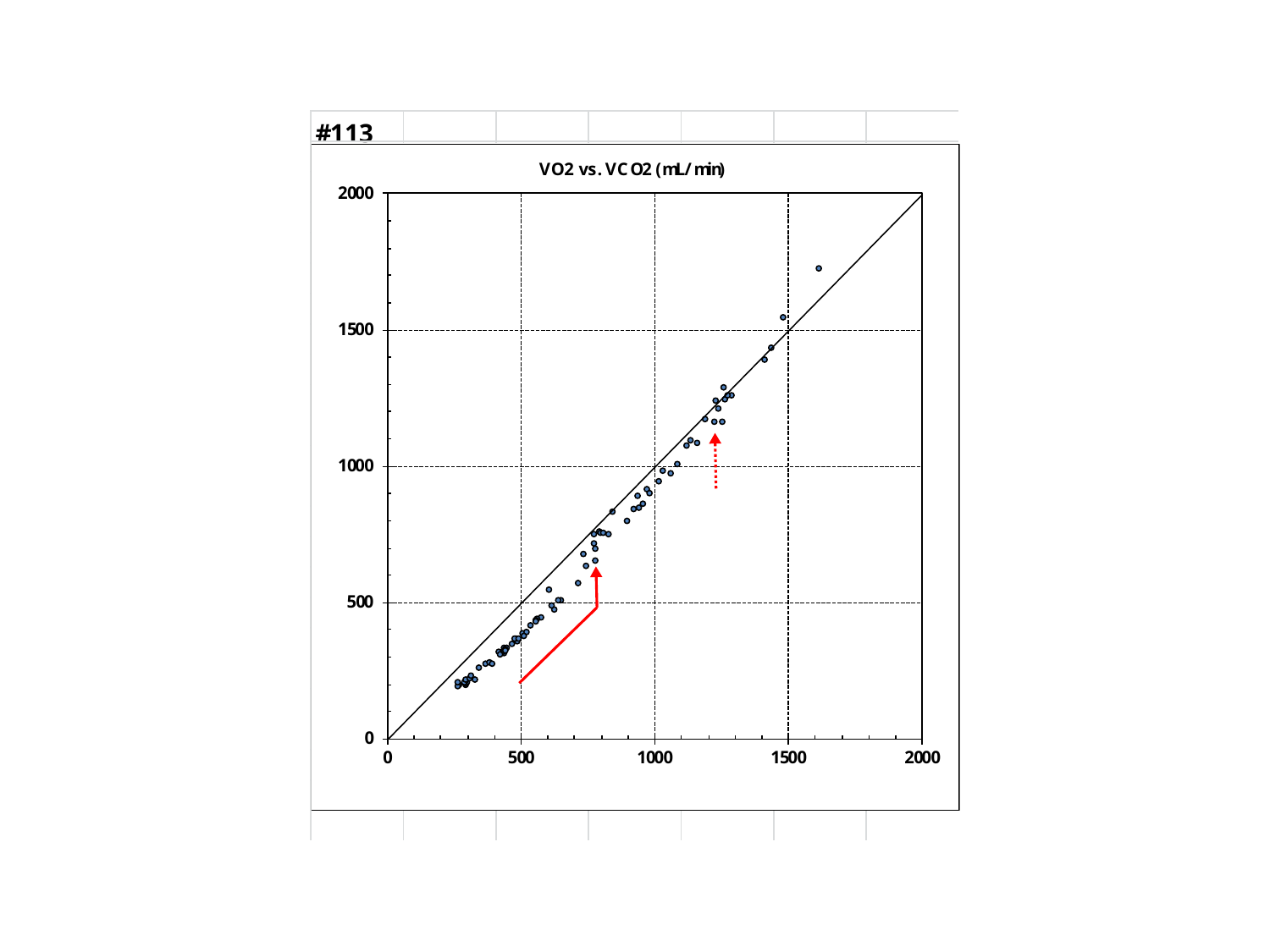

## Slide 116
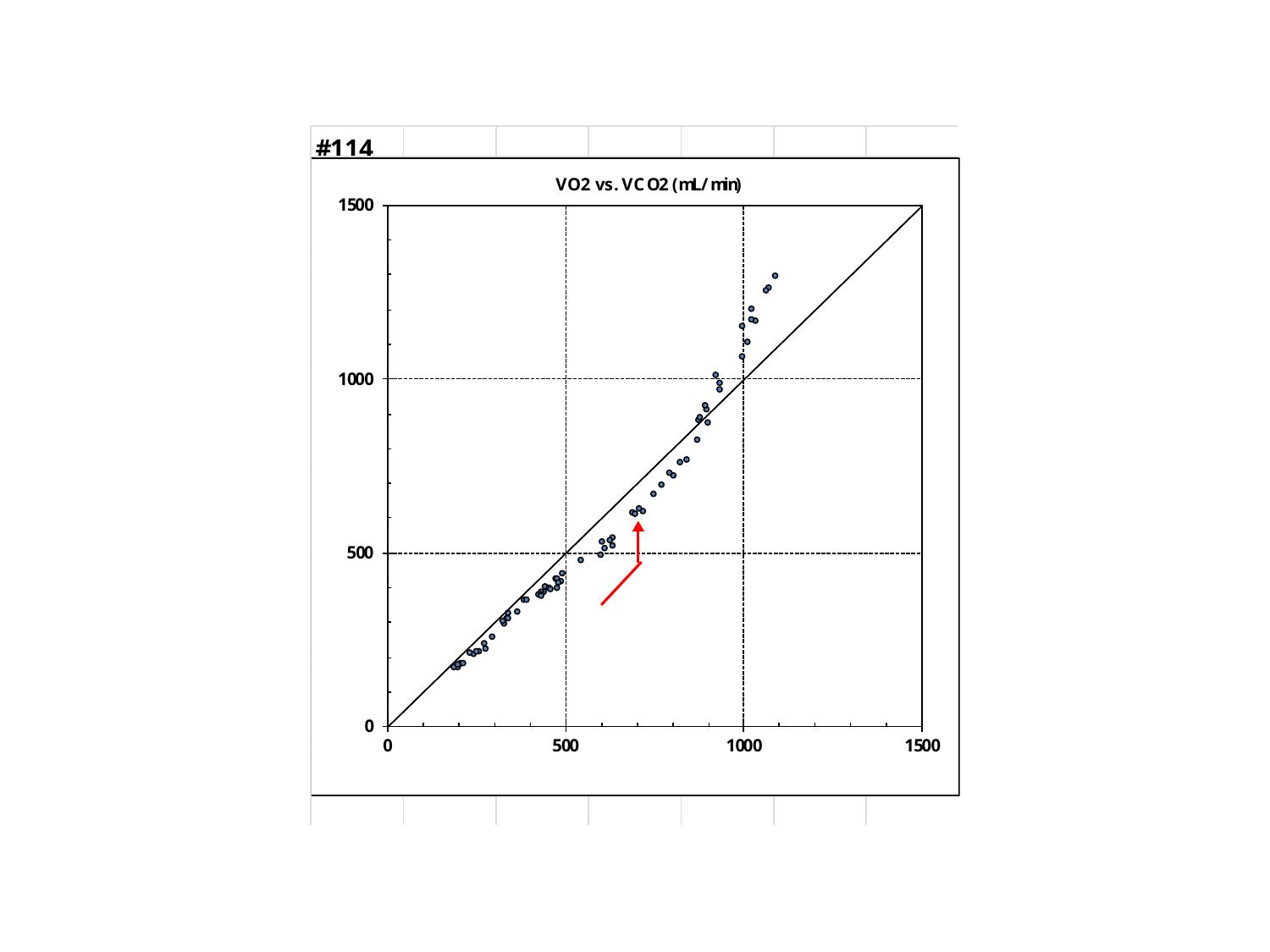

## Slide 117
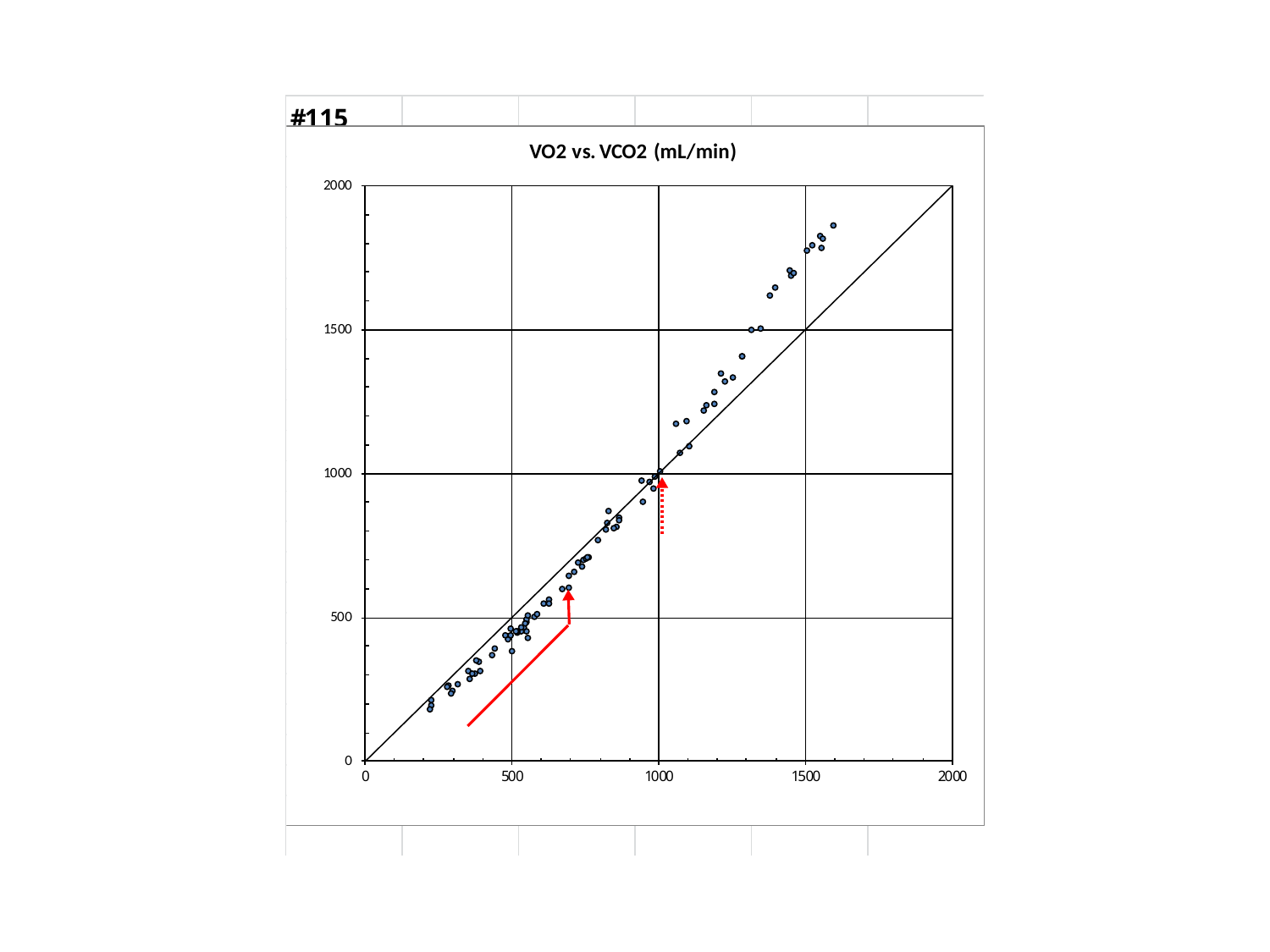

## Slide 118
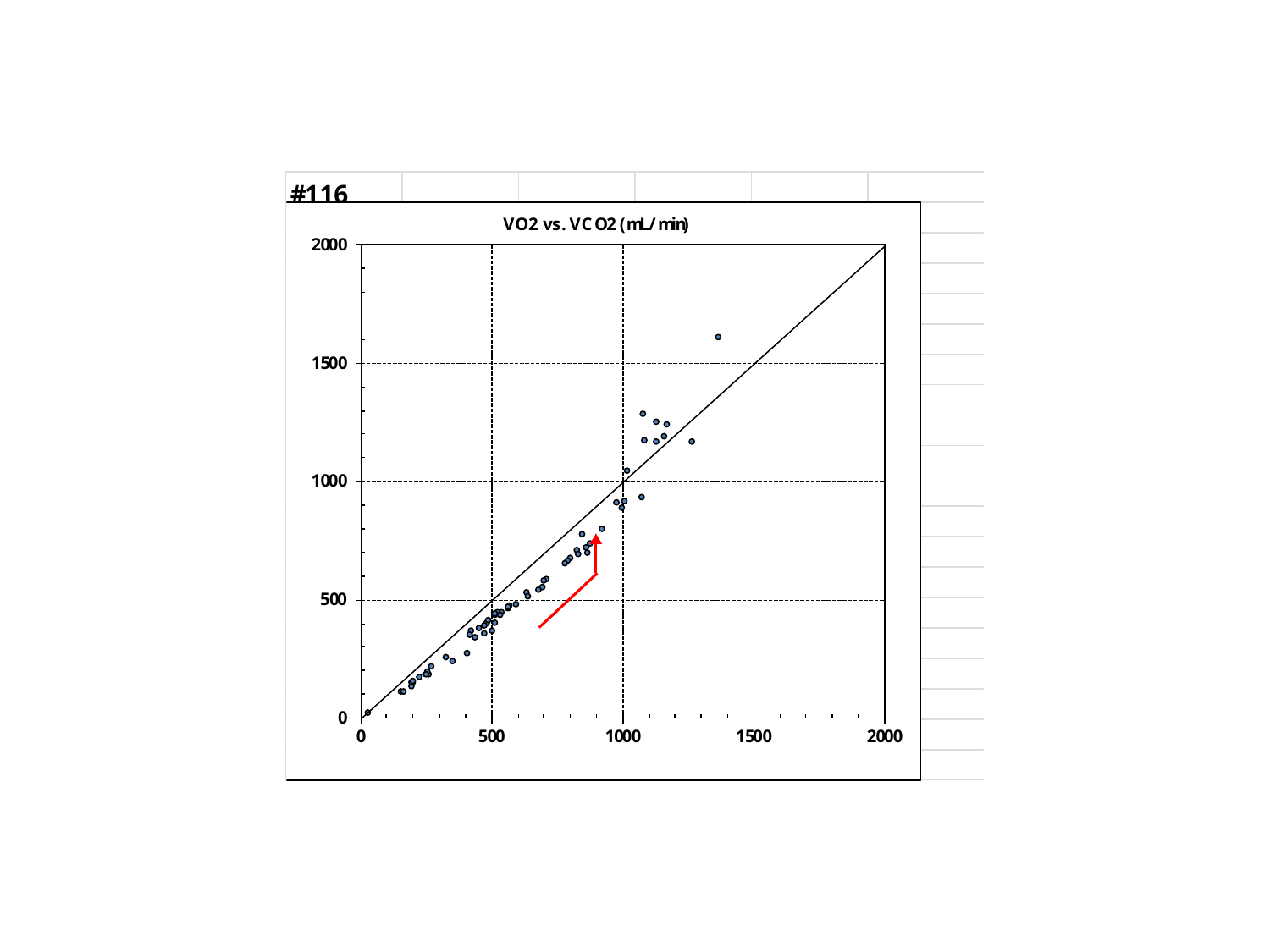

## Slide 119
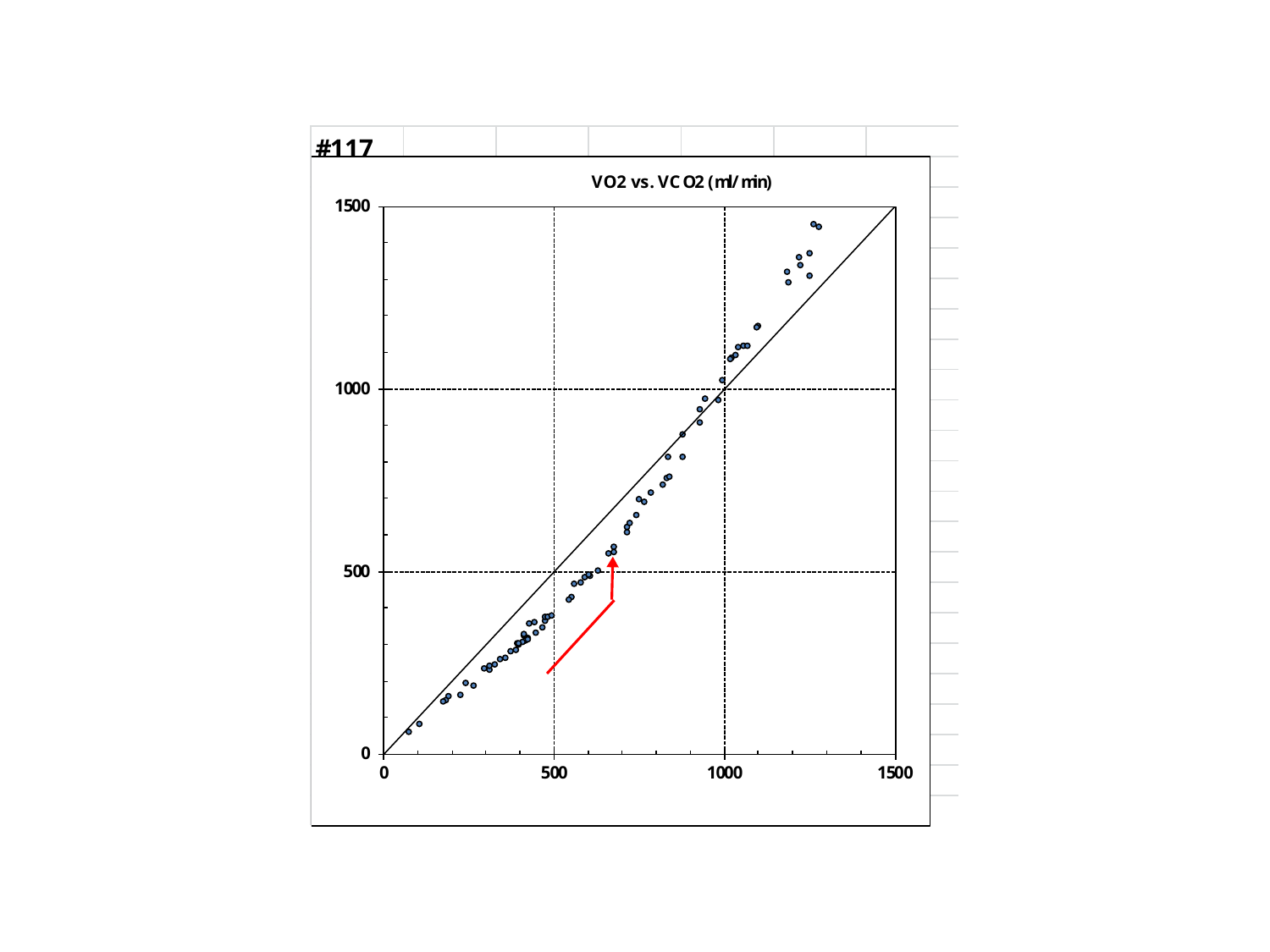

## Slide 120
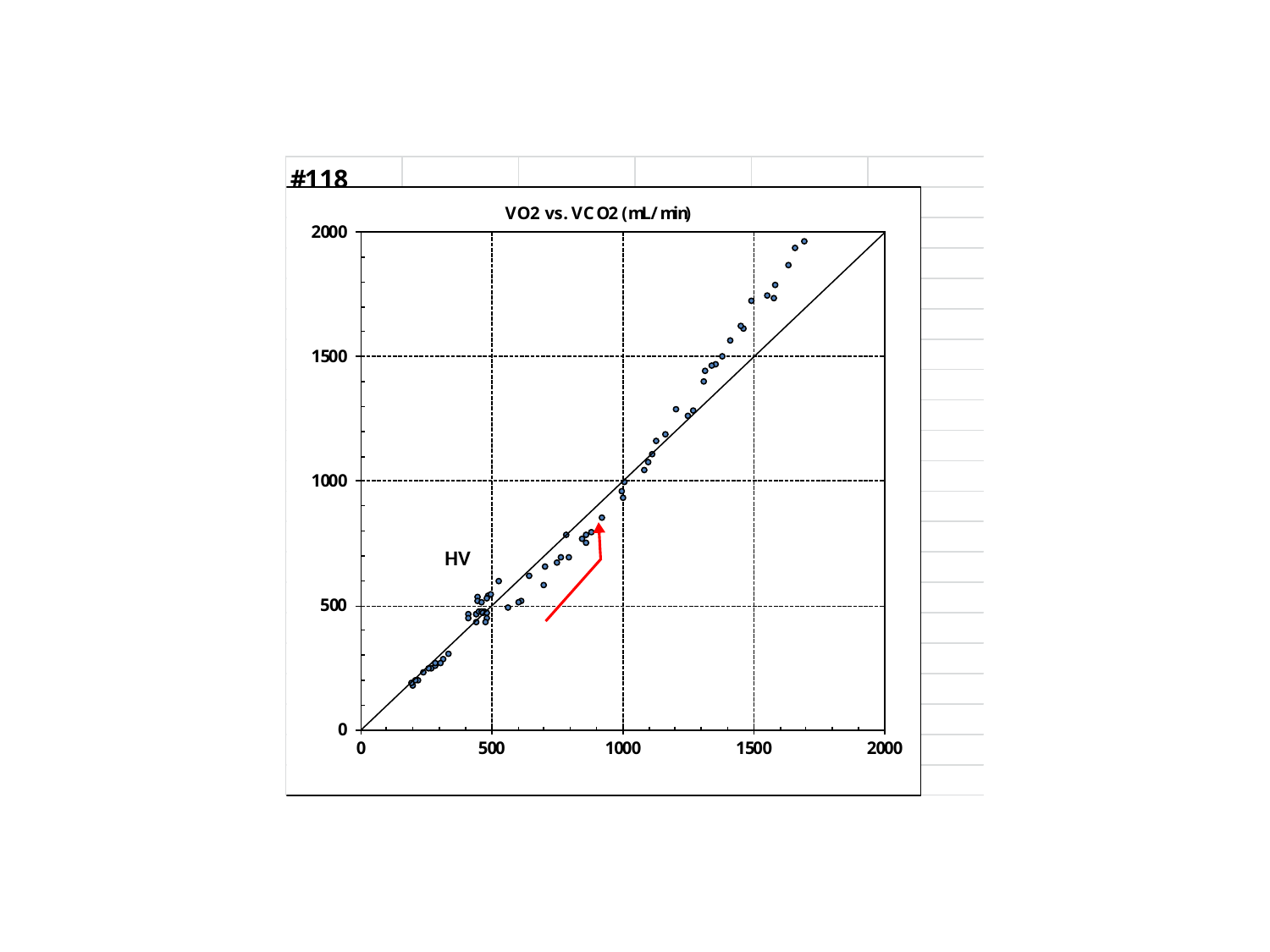

## Slide 121
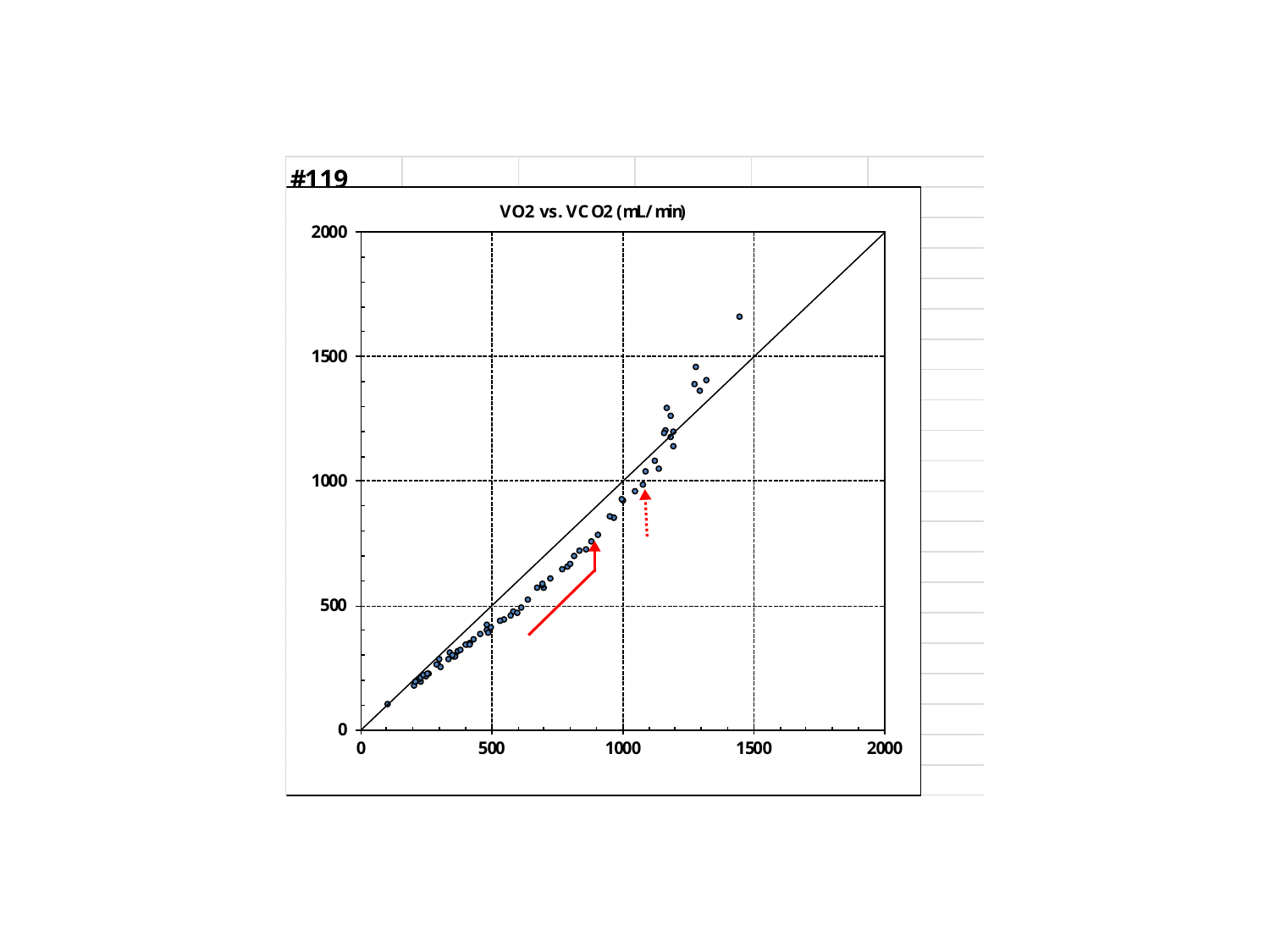

## Slide 122
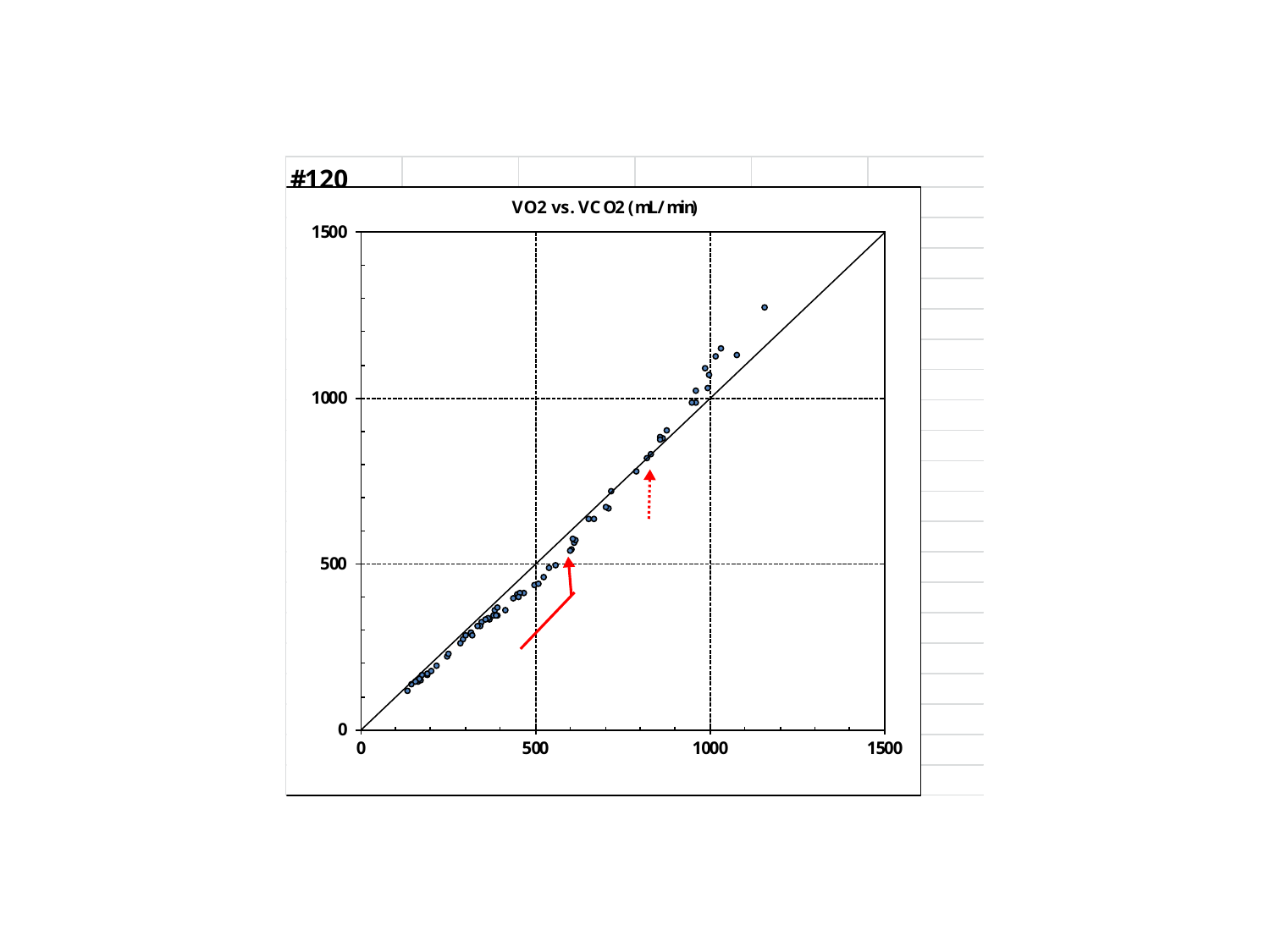

## Slide 123
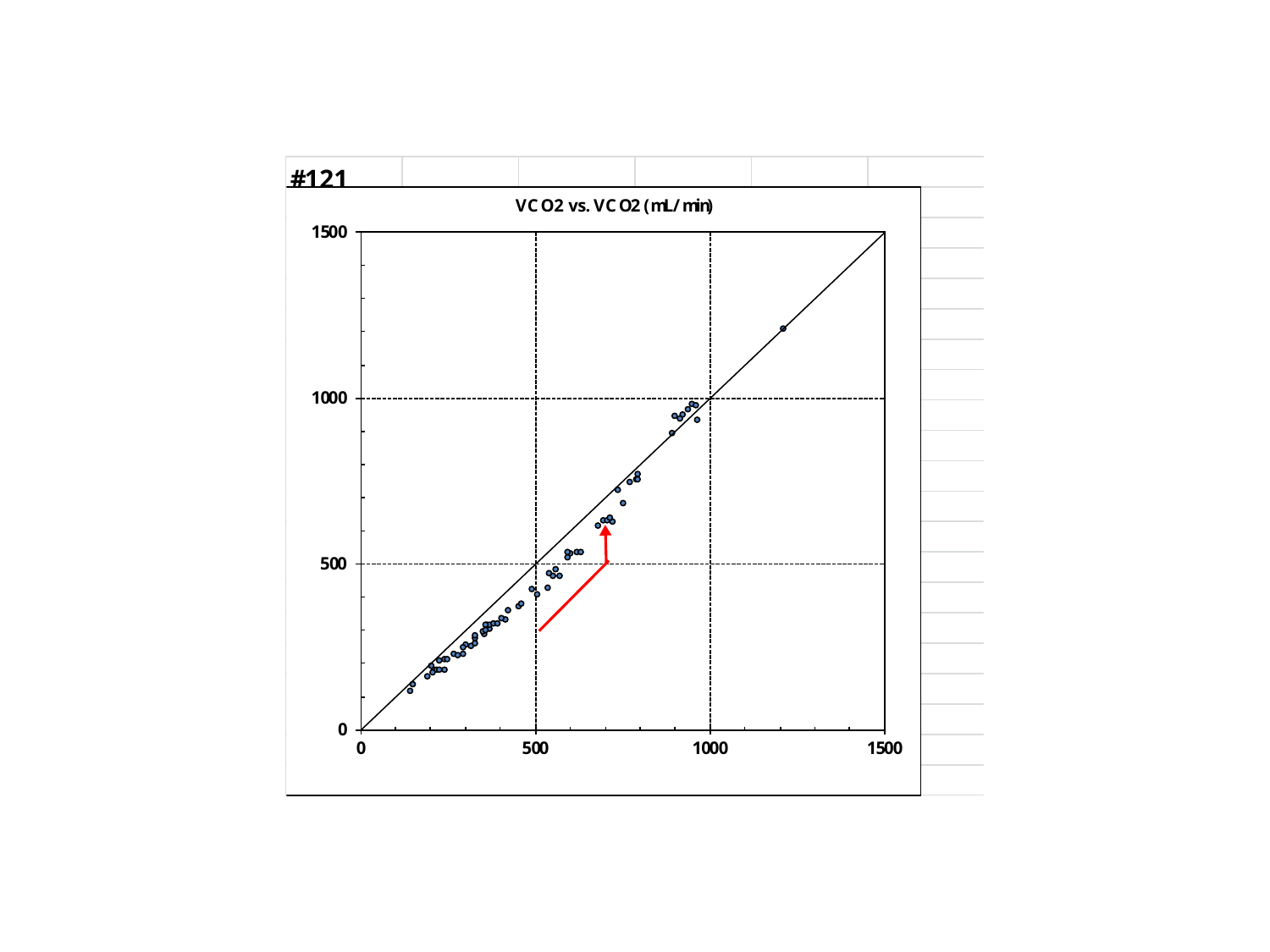

## Slide 124
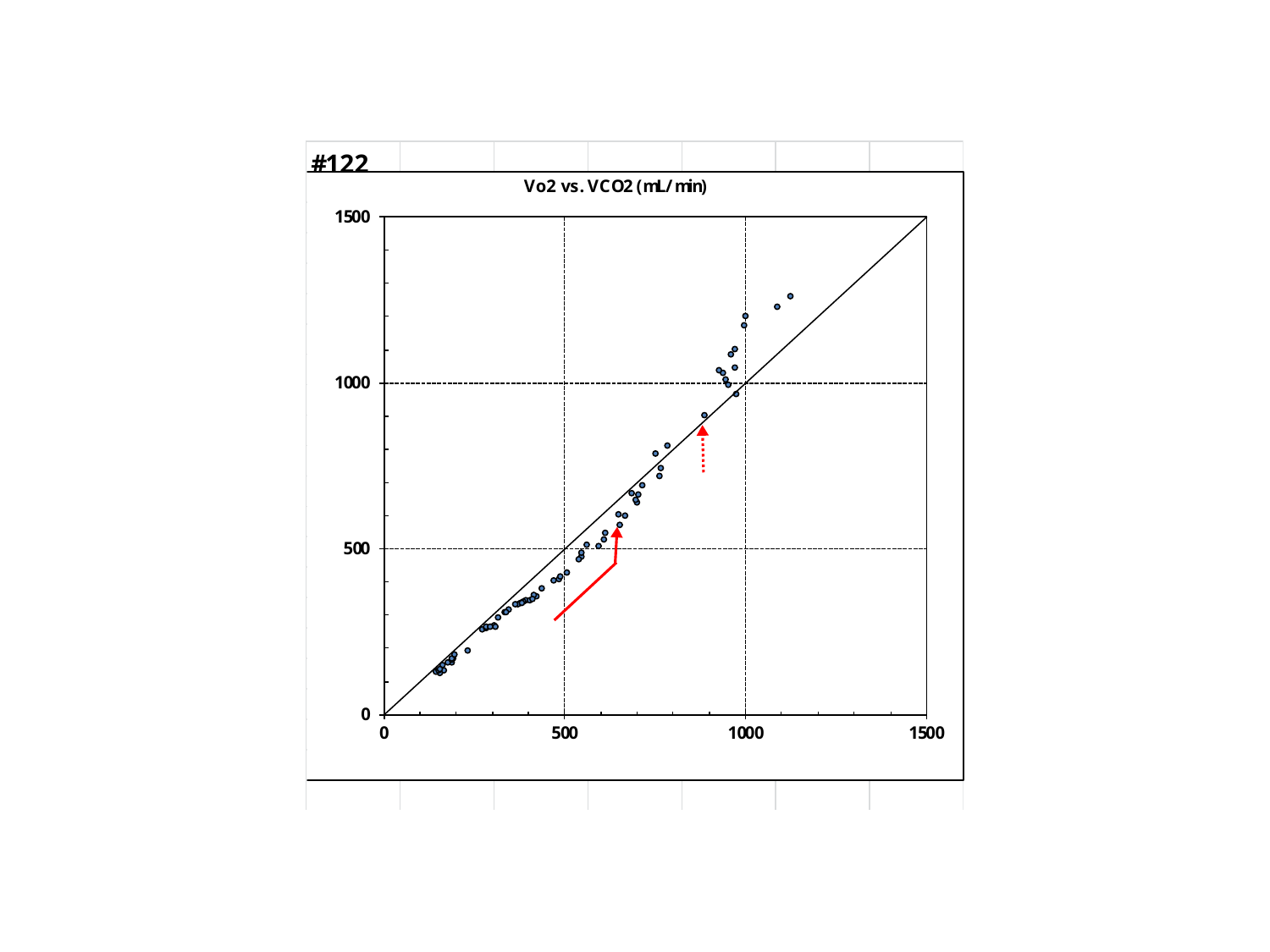

## Slide 125
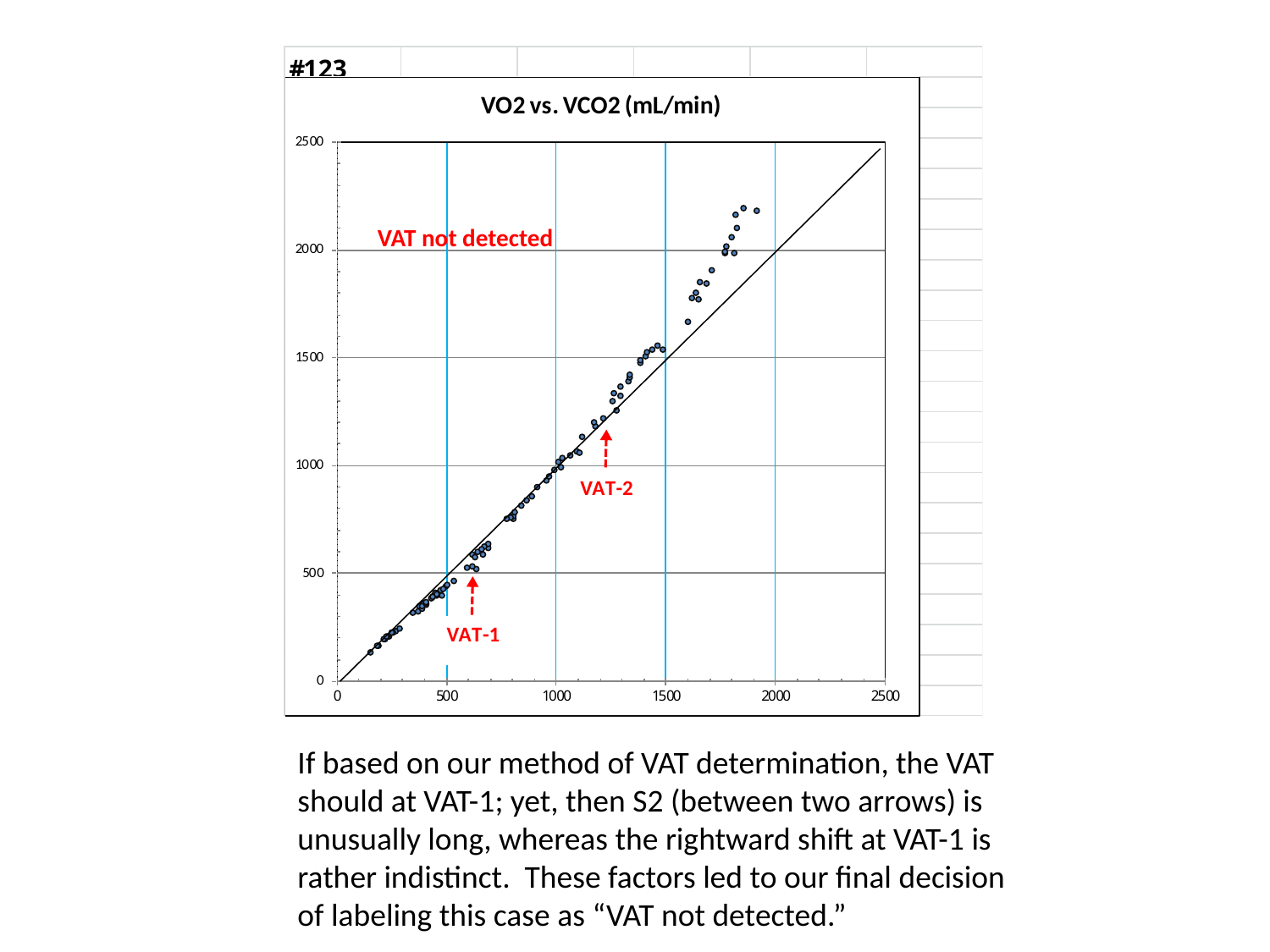

VAT not detected
If based on our method of VAT determination, the VAT should at VAT-1; yet, then S2 (between two arrows) is unusually long, whereas the rightward shift at VAT-1 is rather indistinct. These factors led to our final decision of labeling this case as “VAT not detected.”

## Slide 126
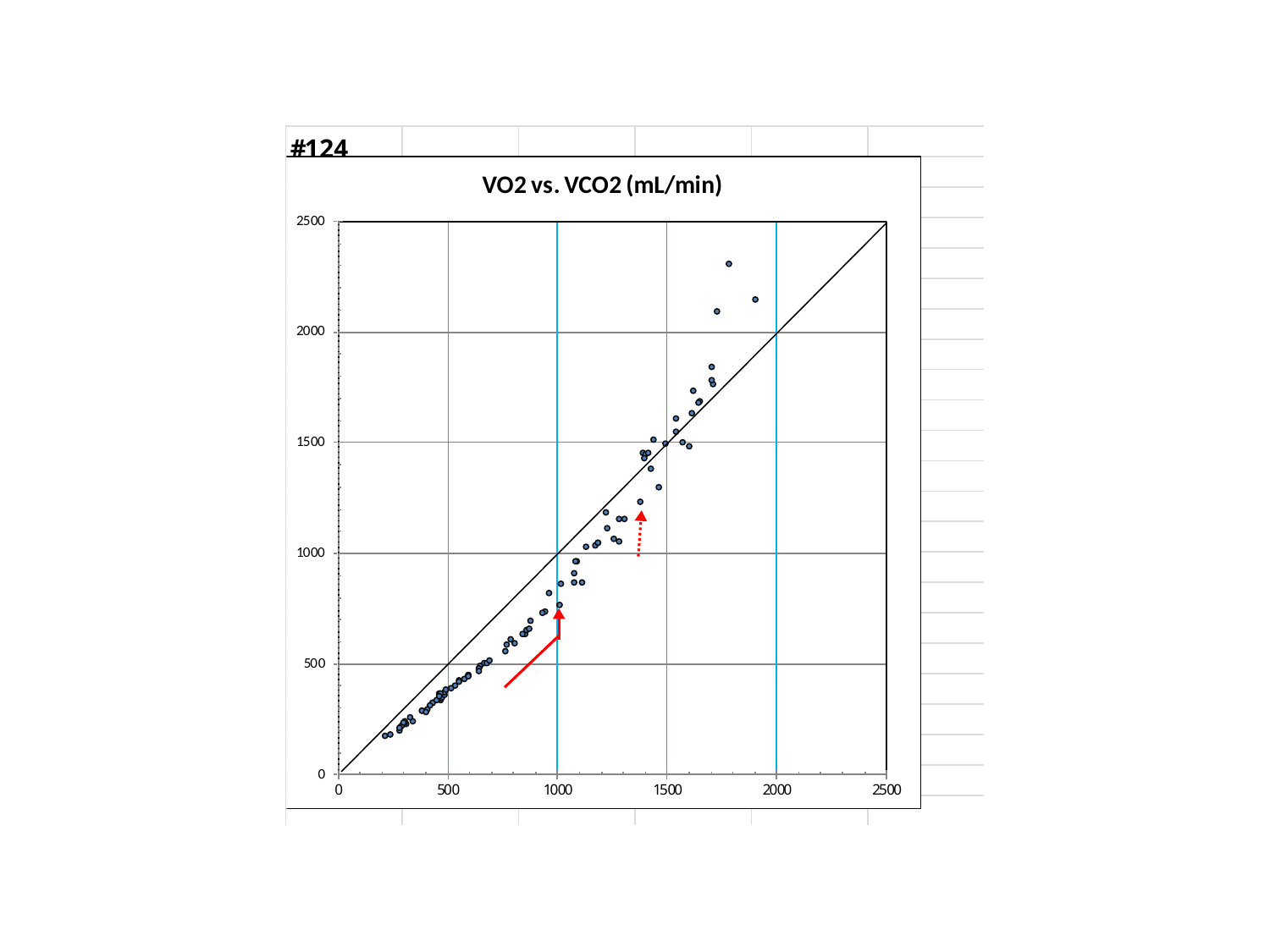

## Slide 127
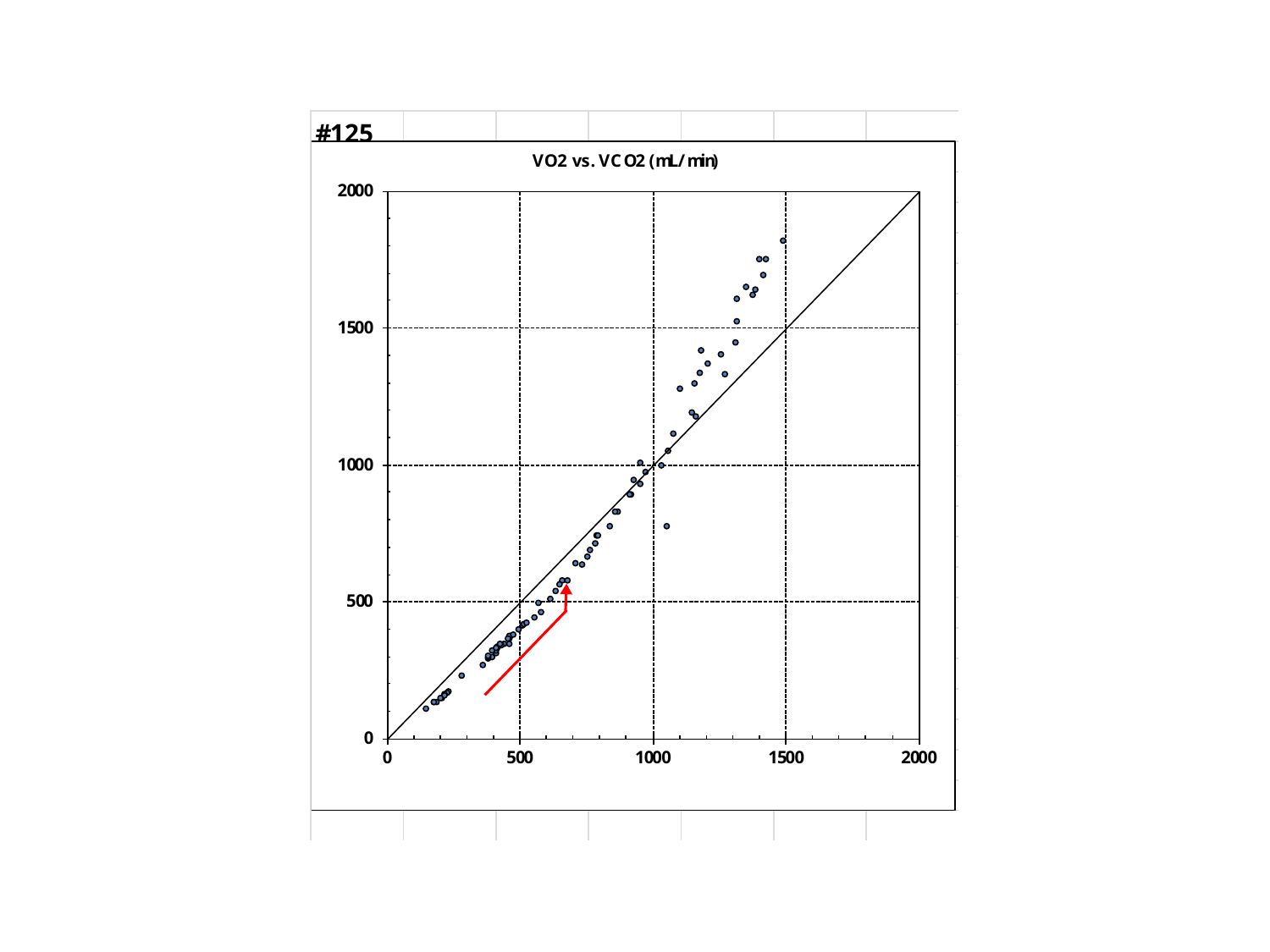

## Slide 128
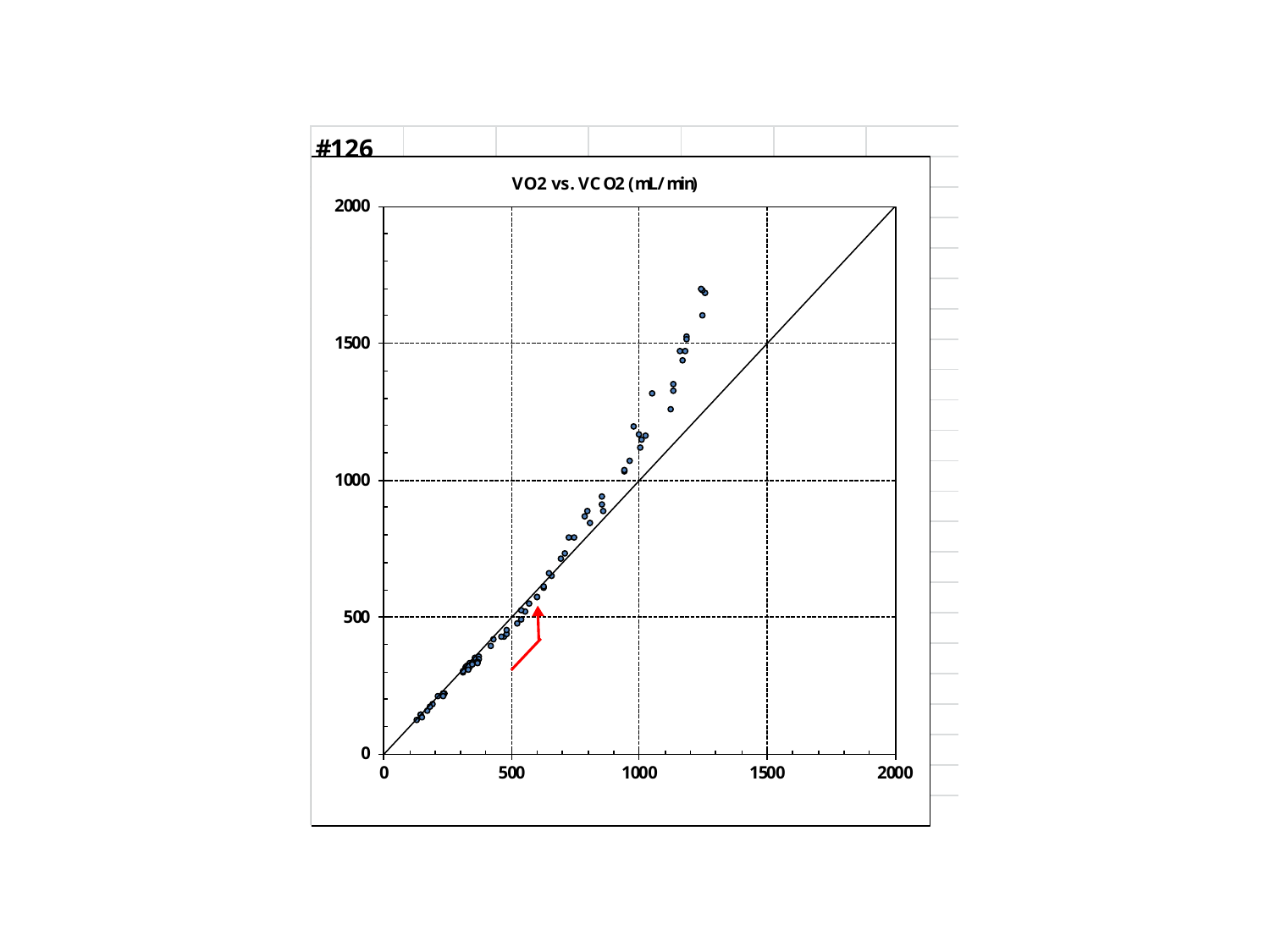

## Slide 129
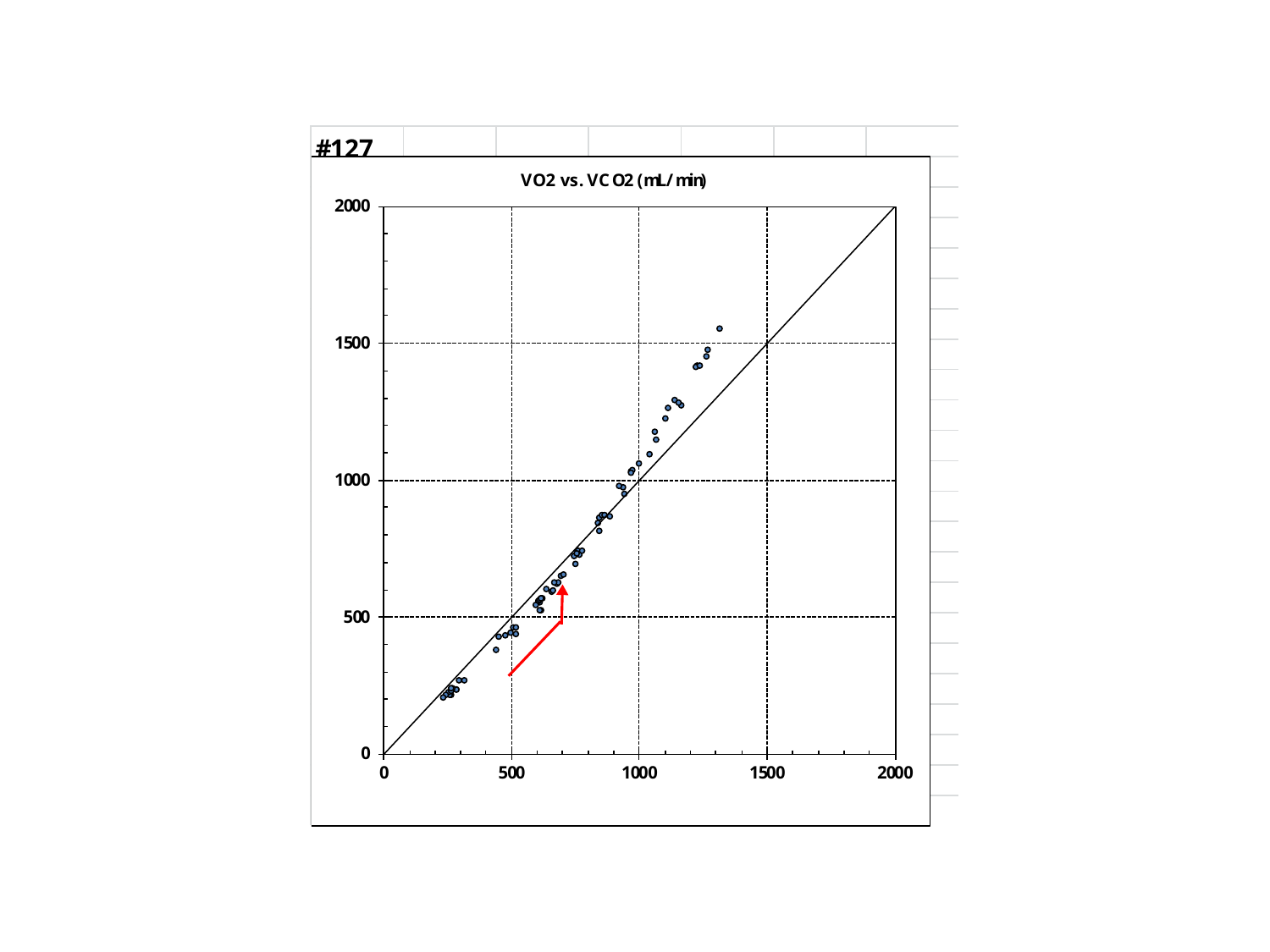

## Slide 130
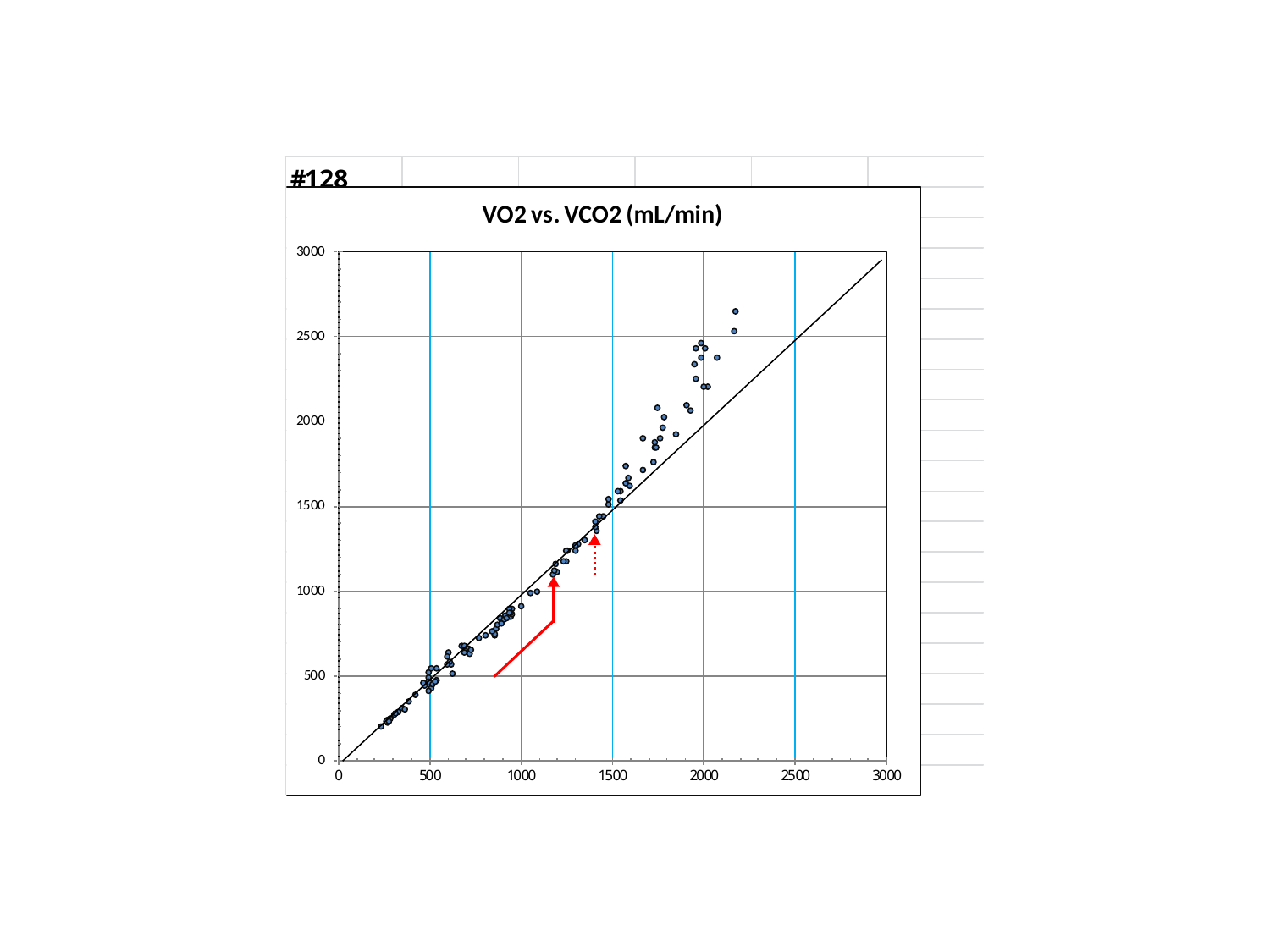

Supplement: Supplementary file 1 — Determination of vVAT in all 128 cases. (PPTX 2642 kb) [file 13102_2019_122_MOESM1_ESM.pptx]
