# Supplementary material for: New method for the mathematical derivation of the ventilatory anaerobic threshold: a retrospective study
Source: BMC Sports Sci Med Rehabil. 2019 Jun 24;11:10. doi: 10.1186/s13102-019-0122-z (PMC6592010; doi:10.1186/s13102-019-0122-z)

## Slide 1
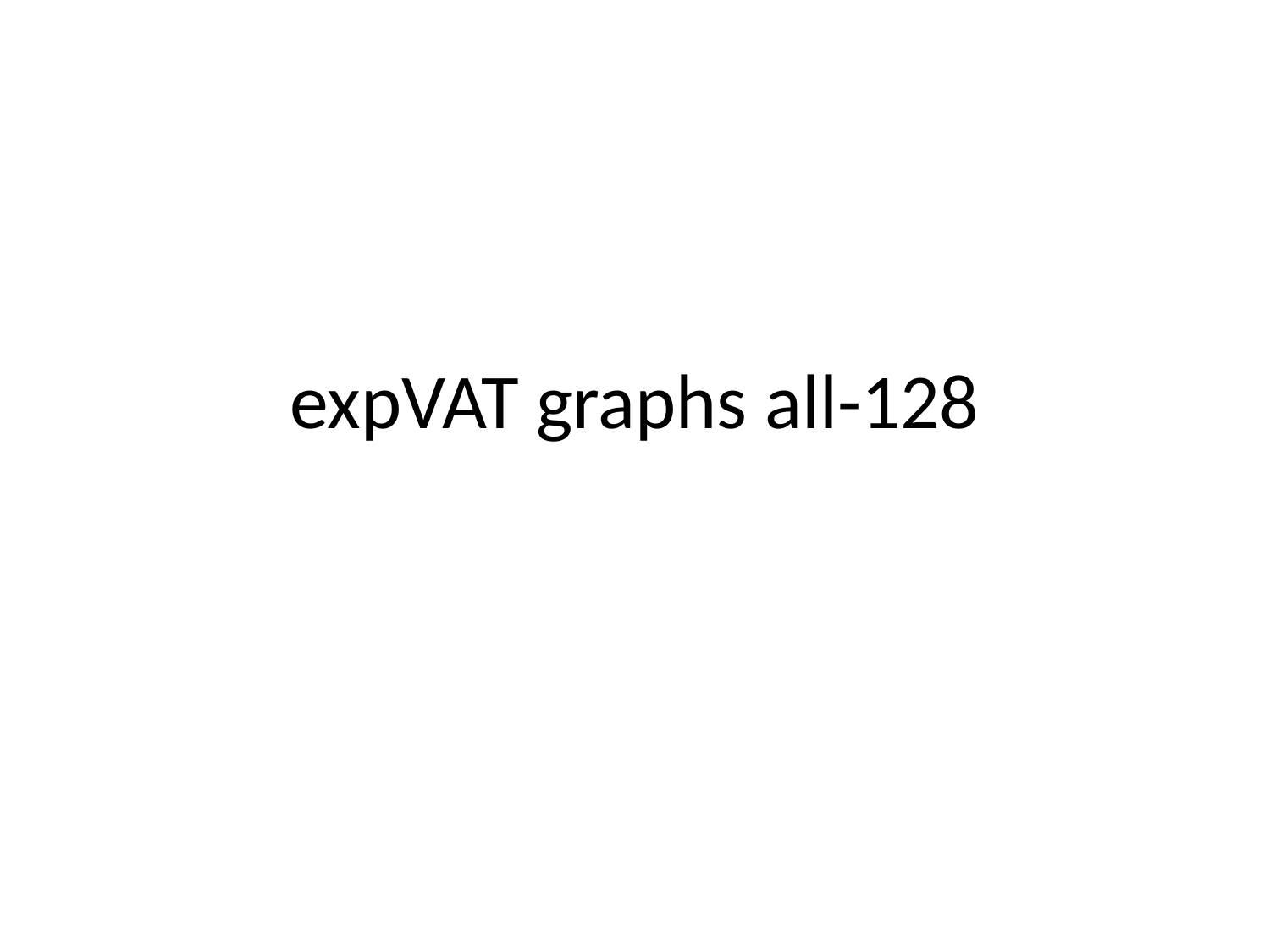

# expVAT graphs all-128

## Slide 2
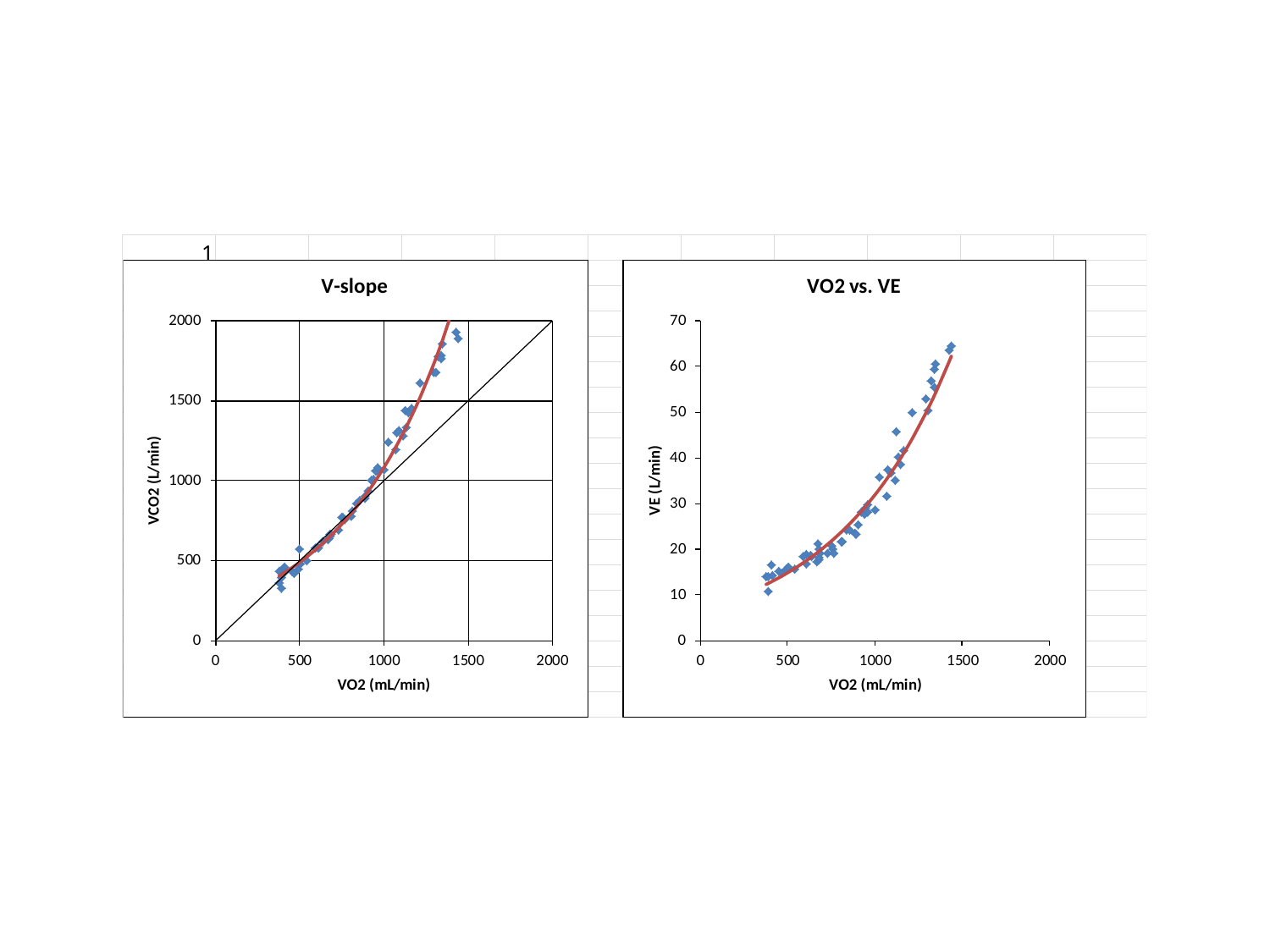

## Slide 3
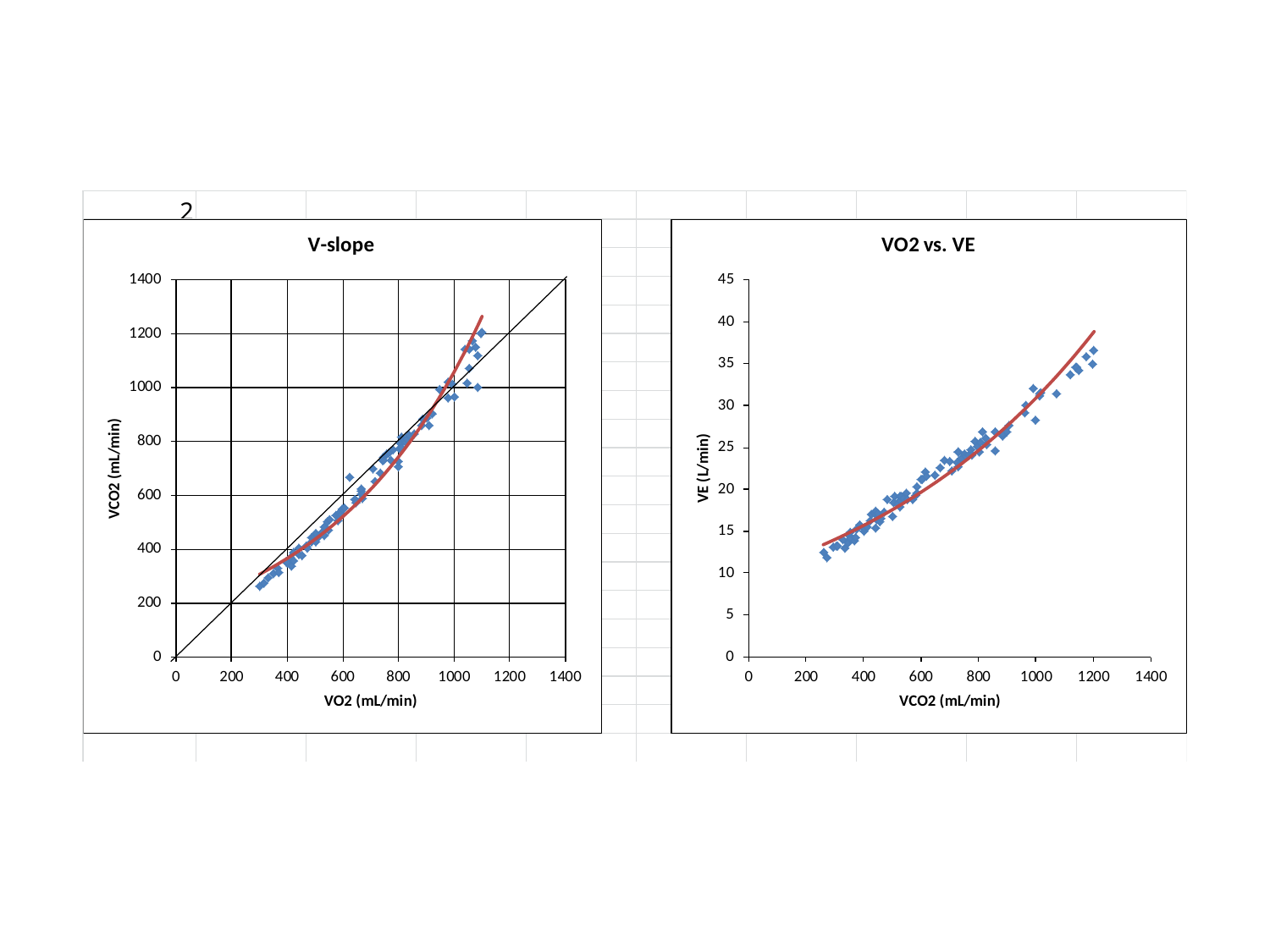

## Slide 4
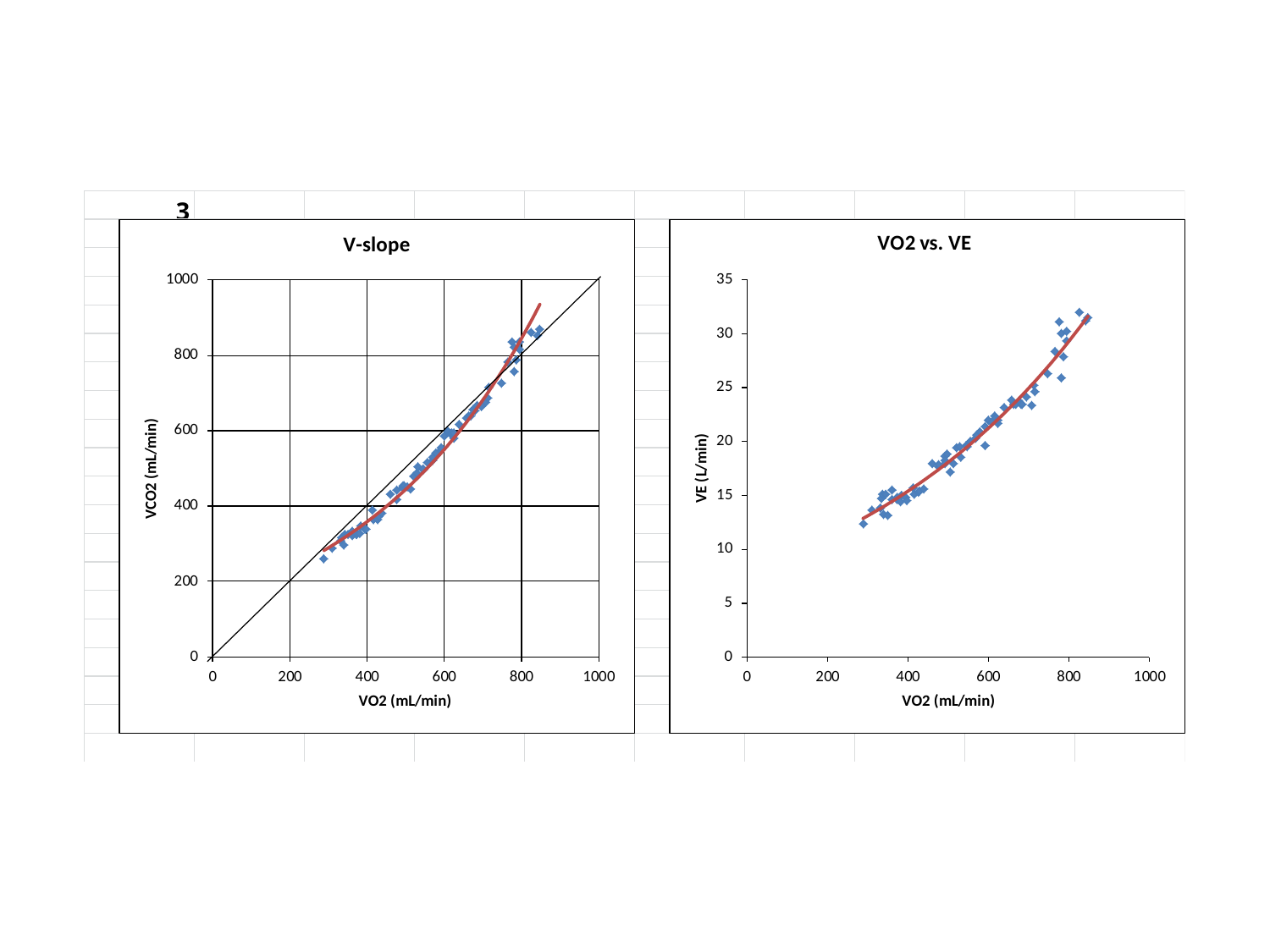

## Slide 5
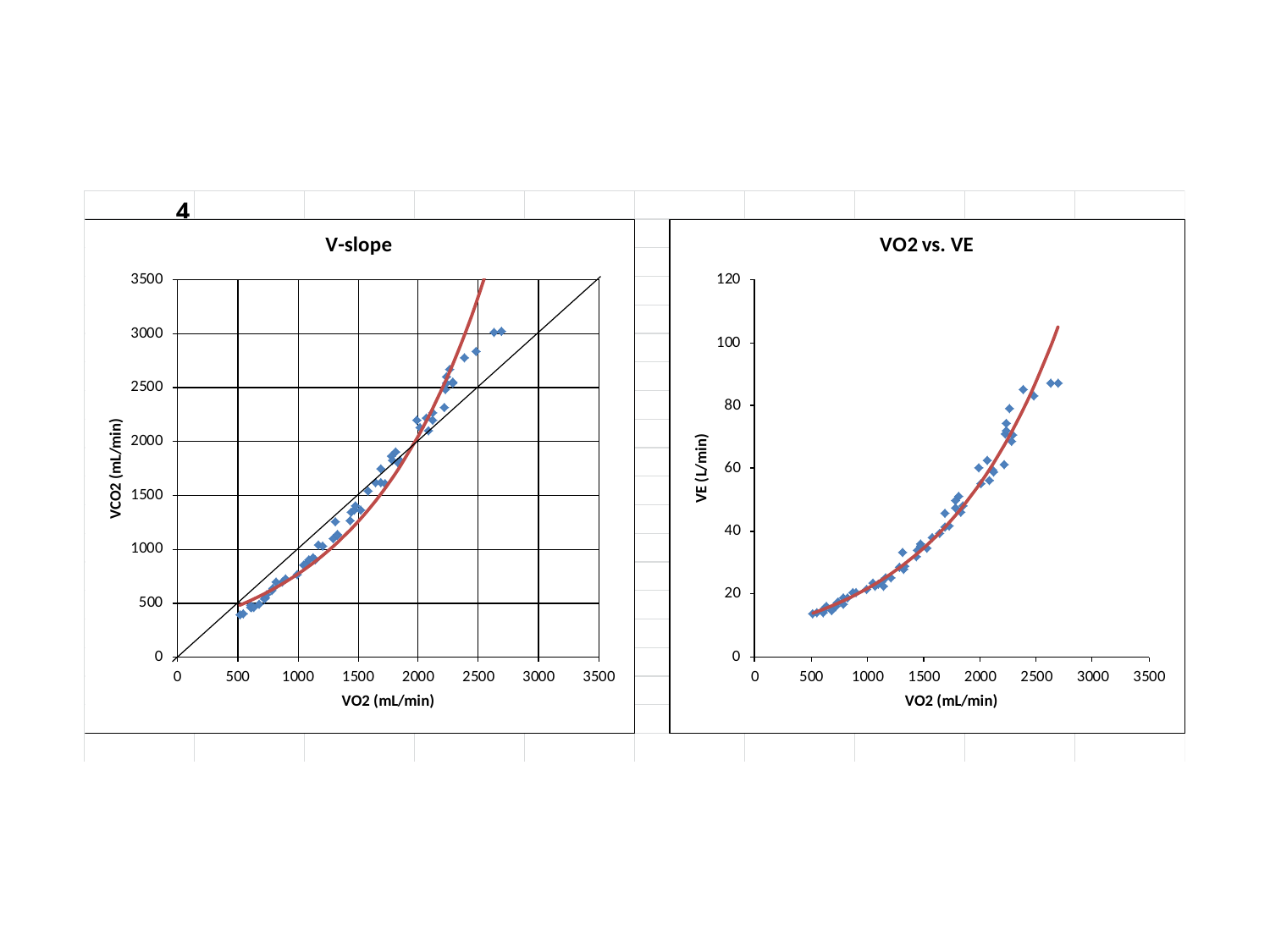

## Slide 6
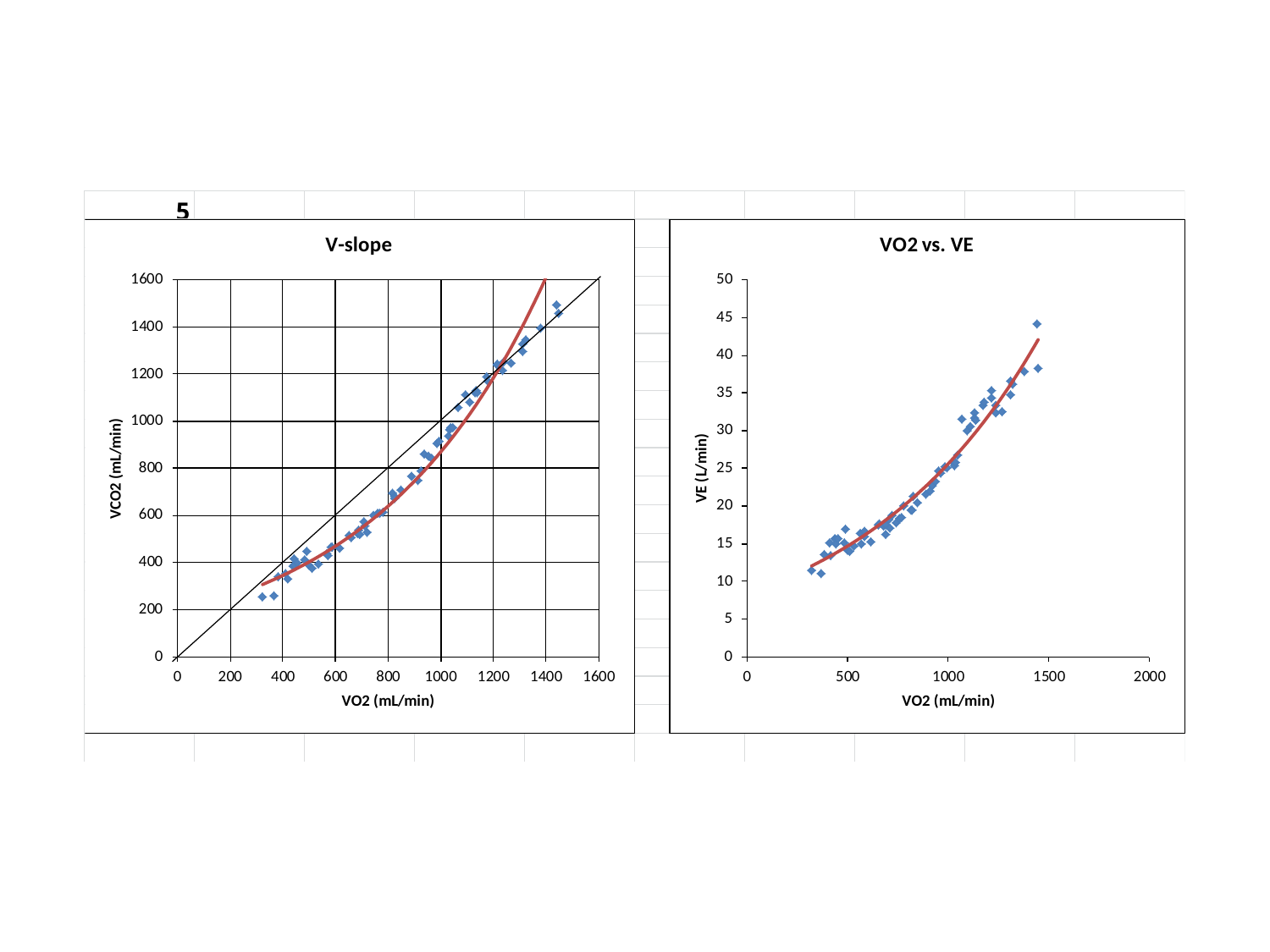

## Slide 7
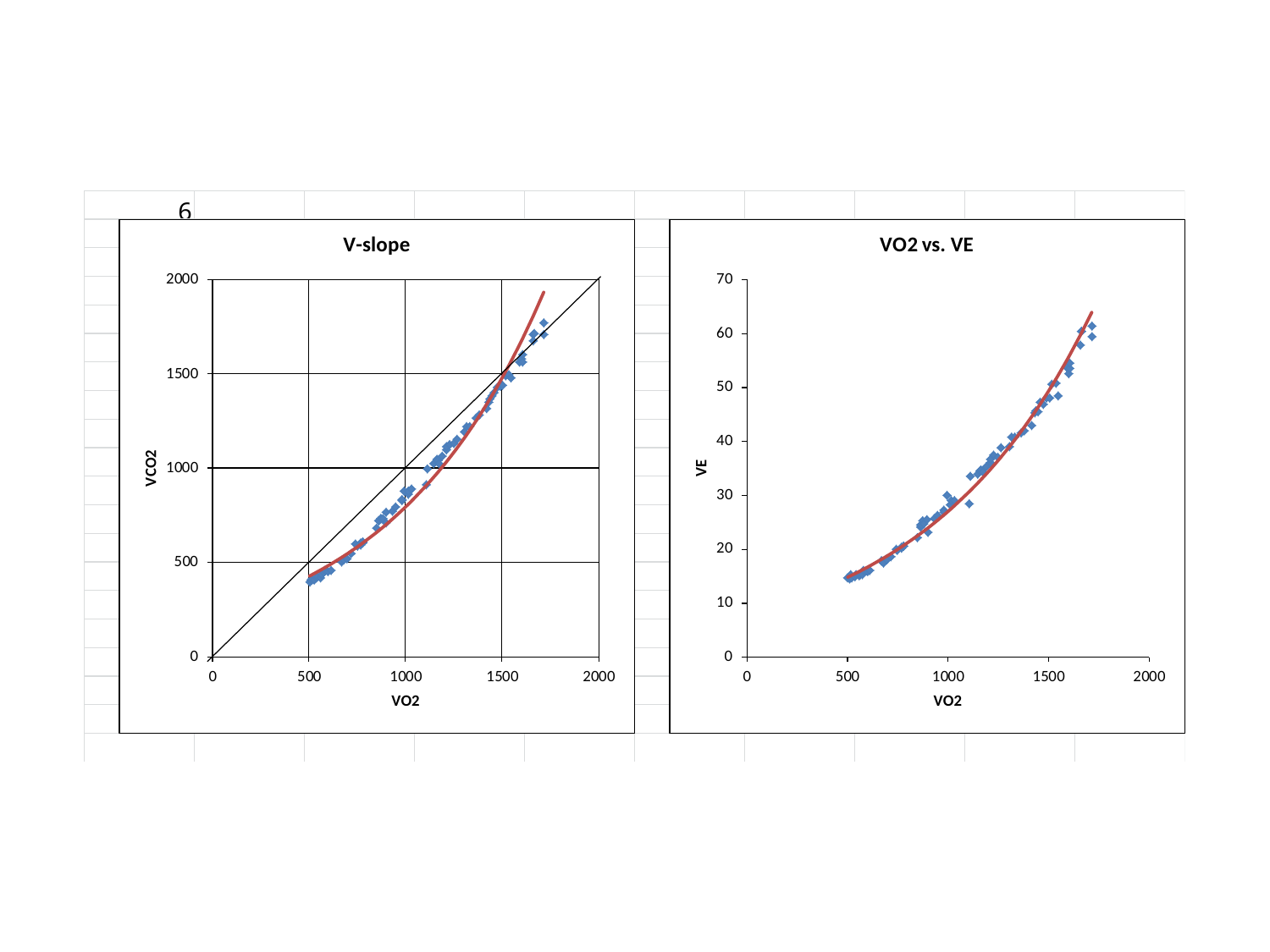

## Slide 8
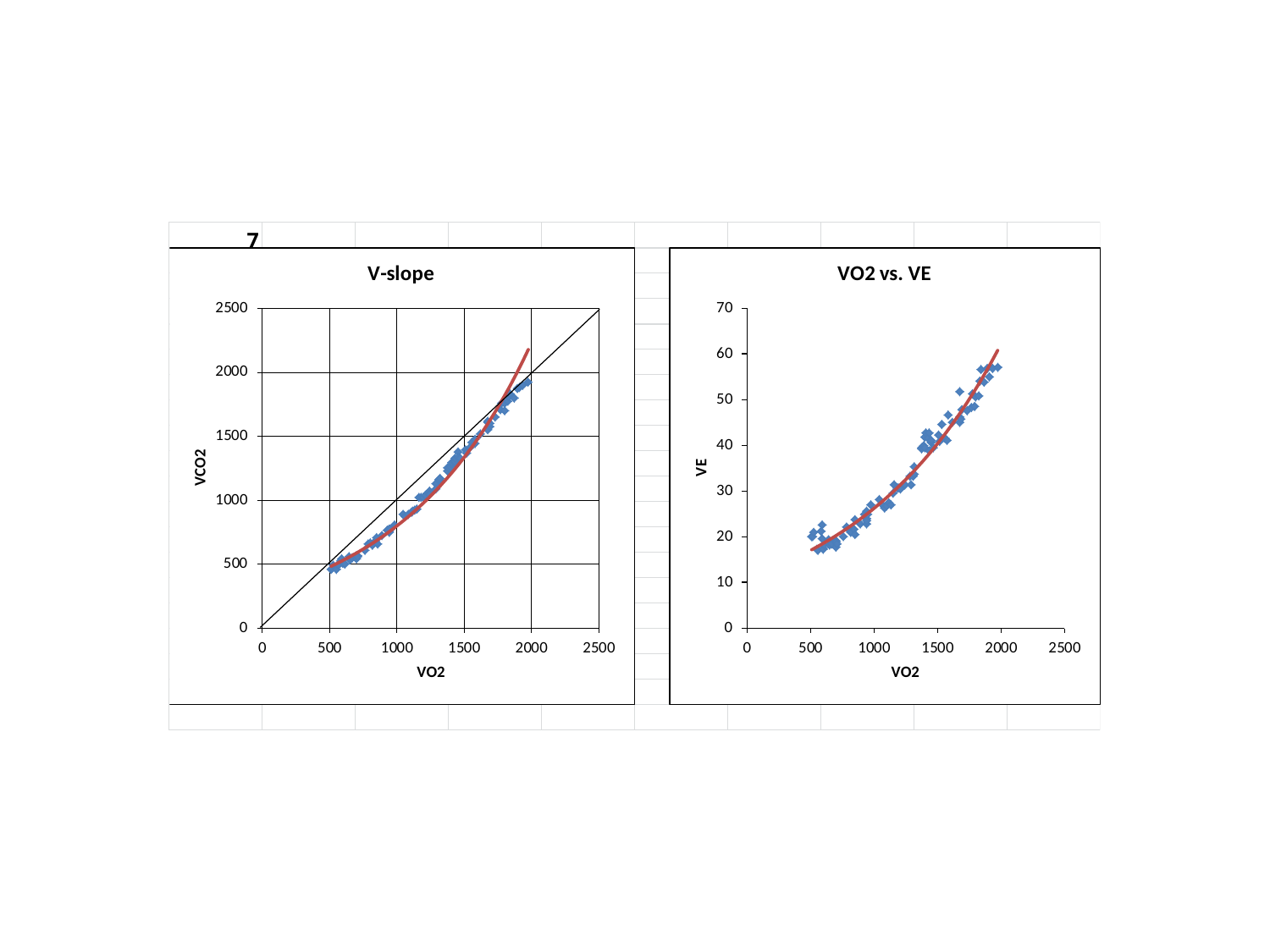

## Slide 9
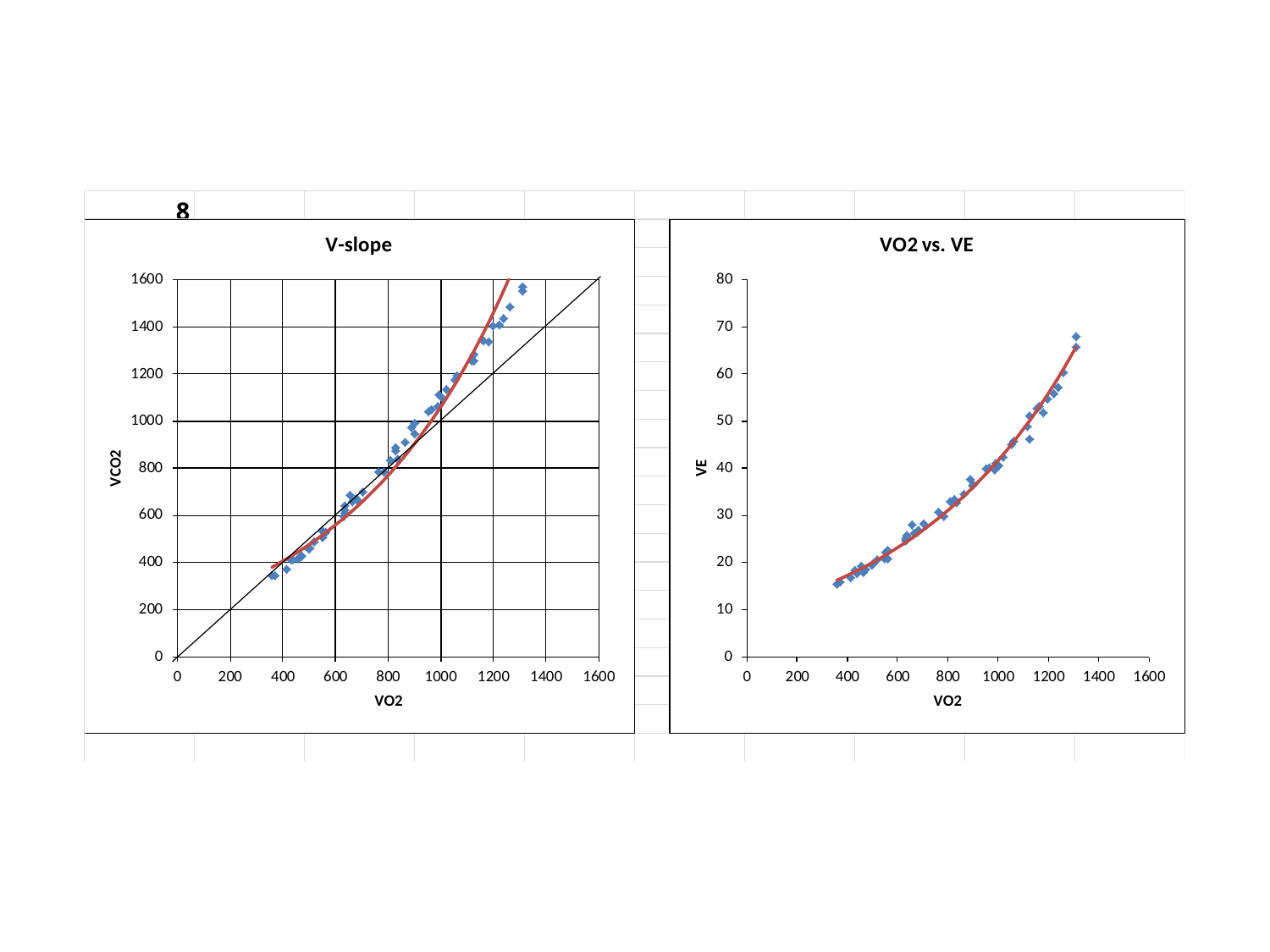

## Slide 10
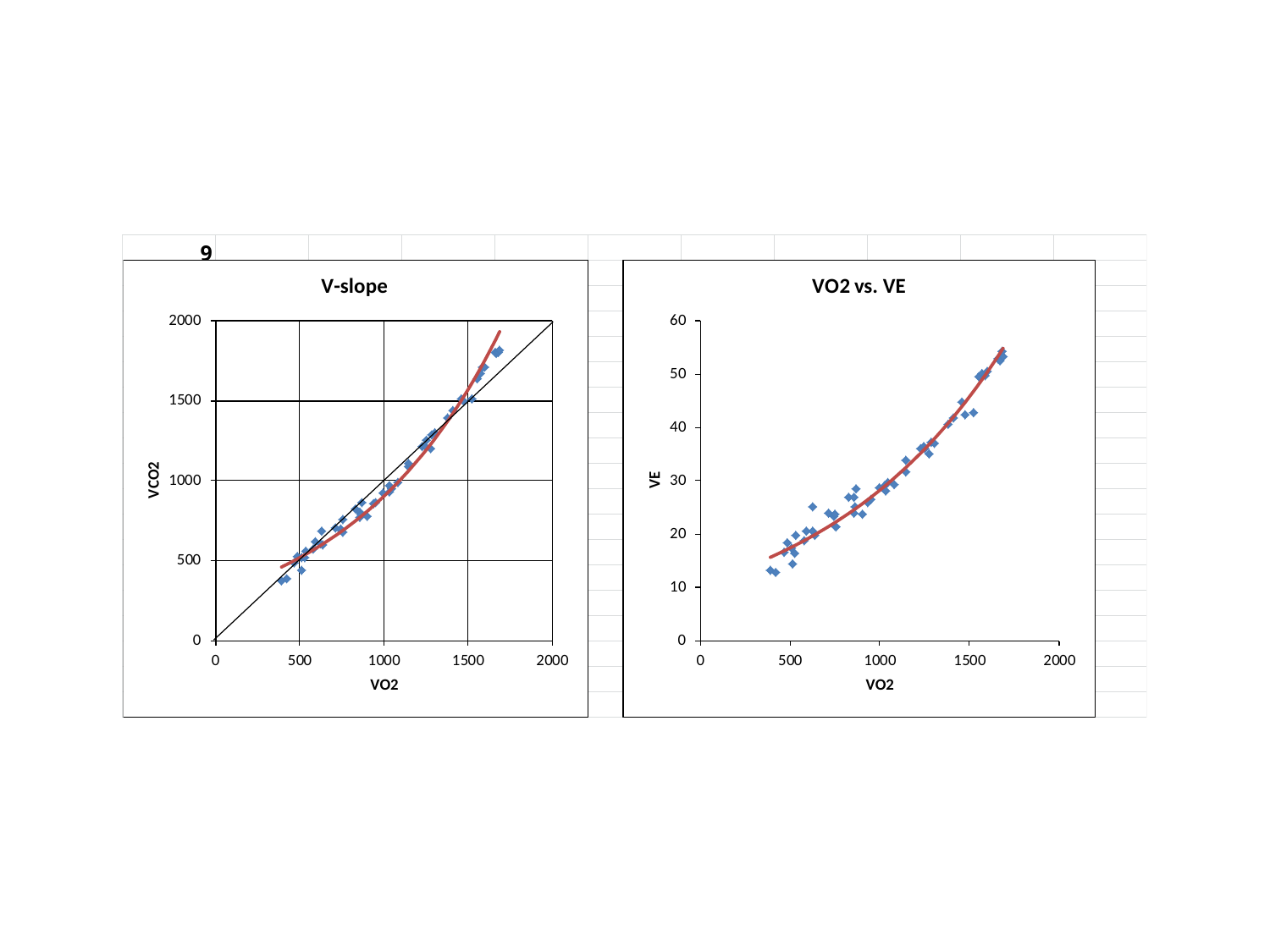

## Slide 11
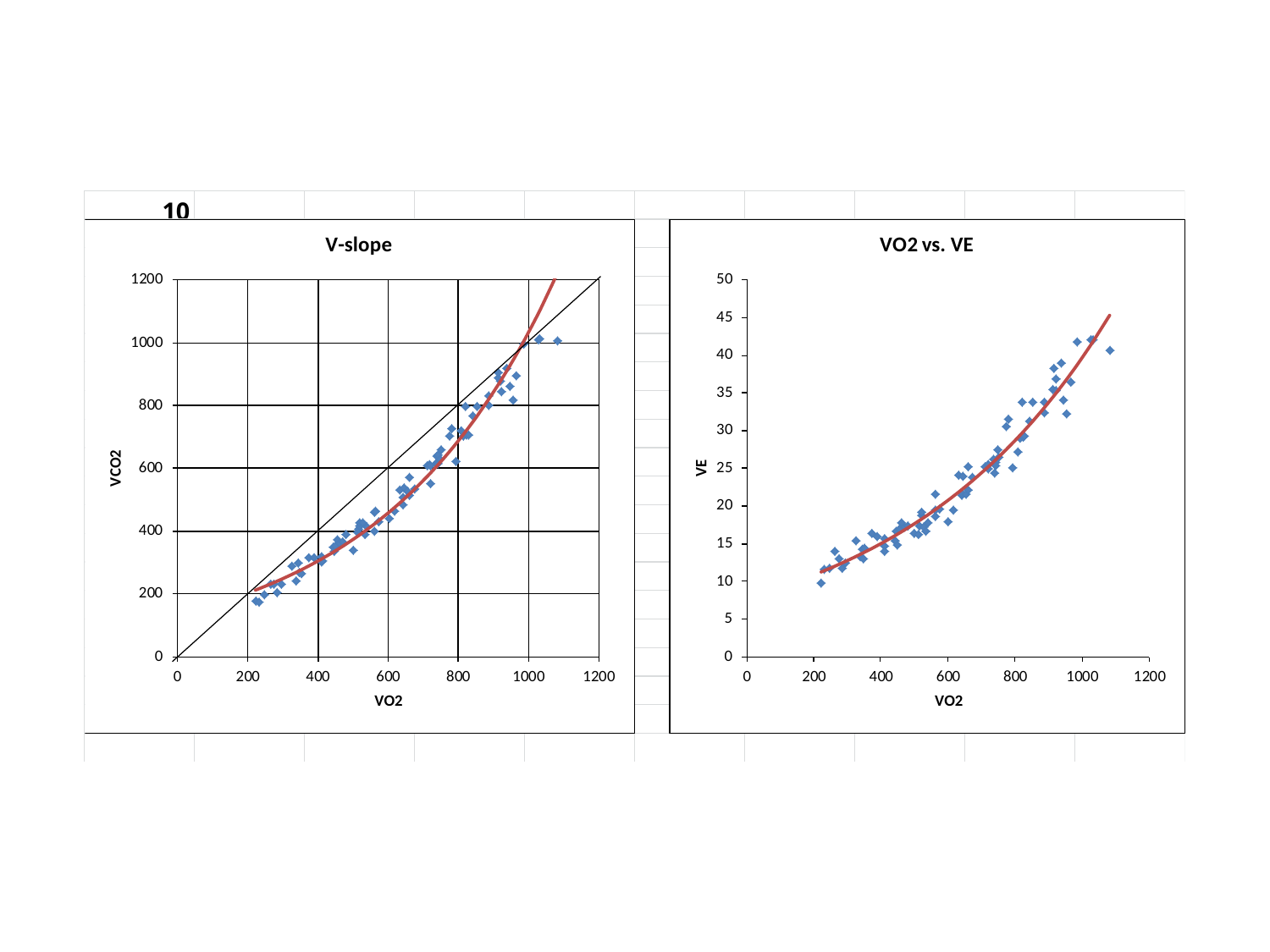

## Slide 12
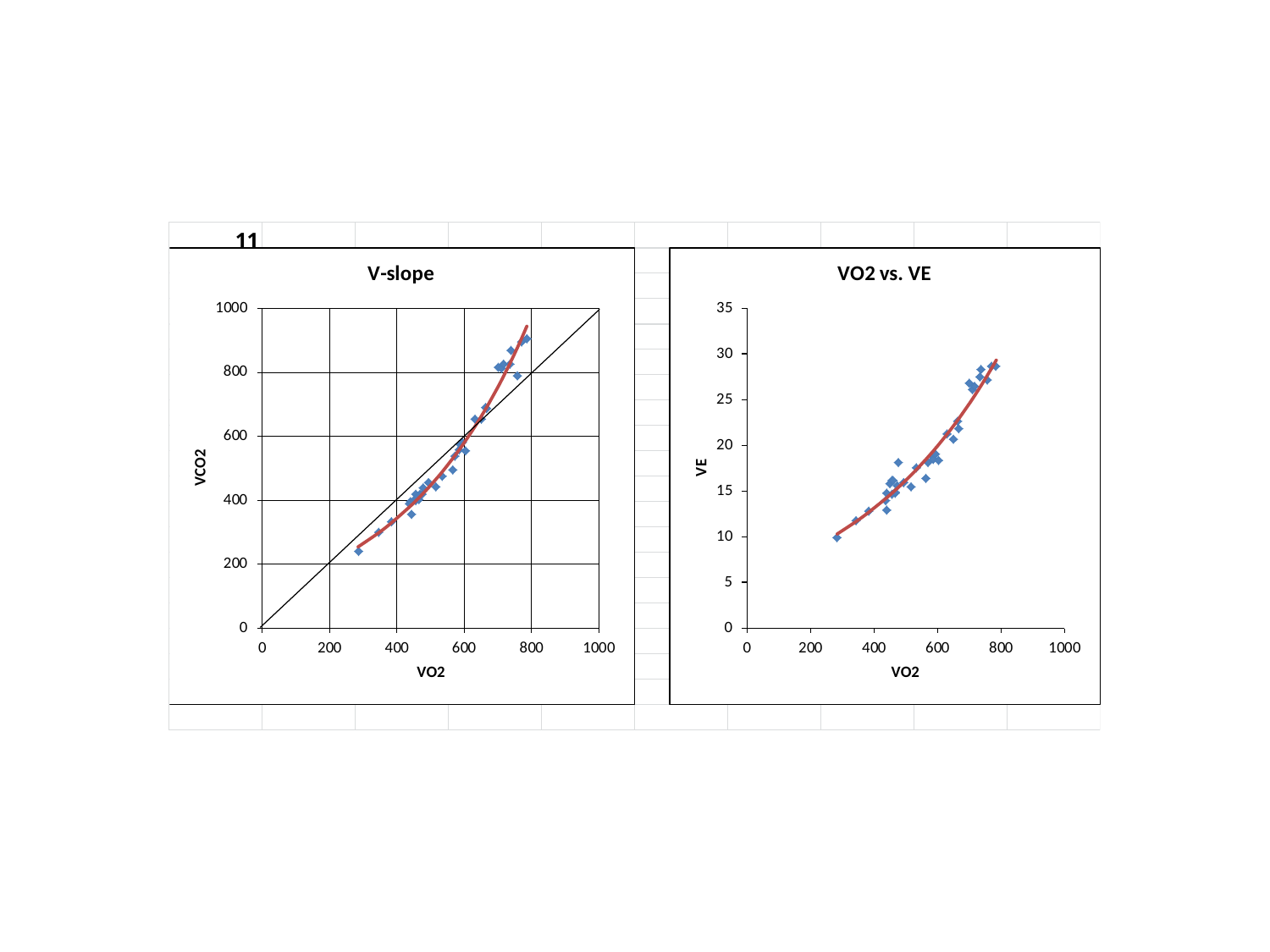

## Slide 13
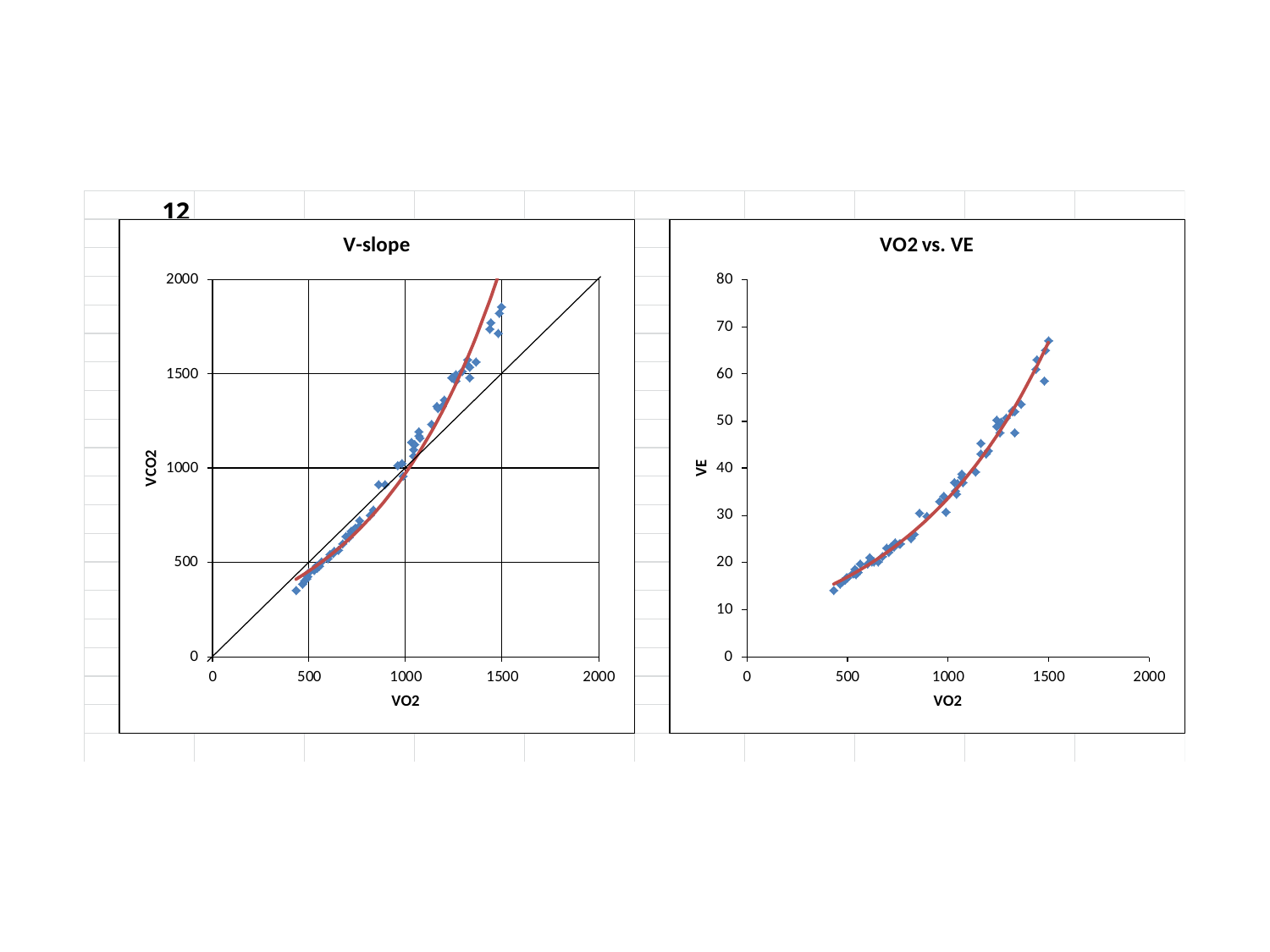

## Slide 14
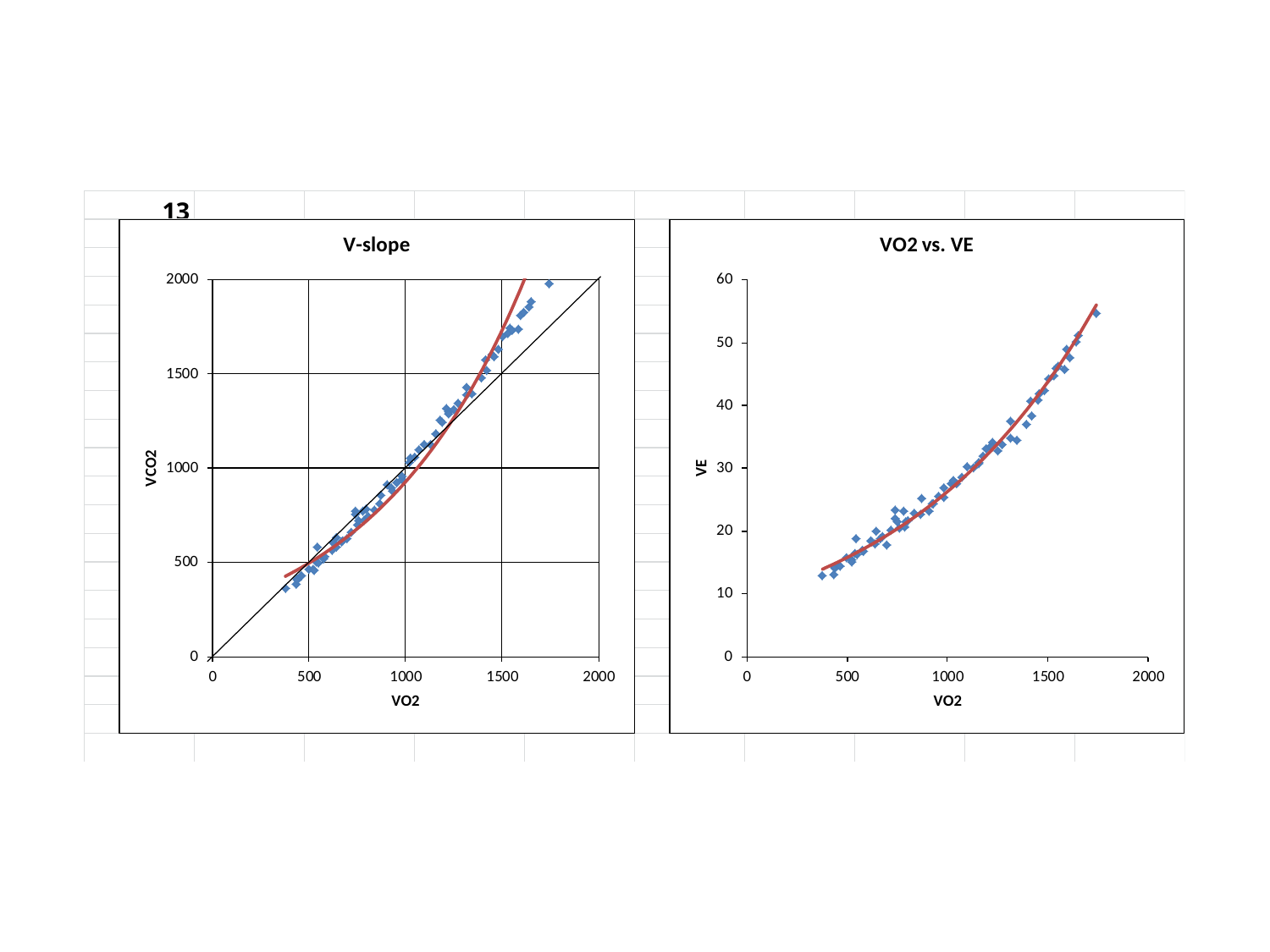

## Slide 15
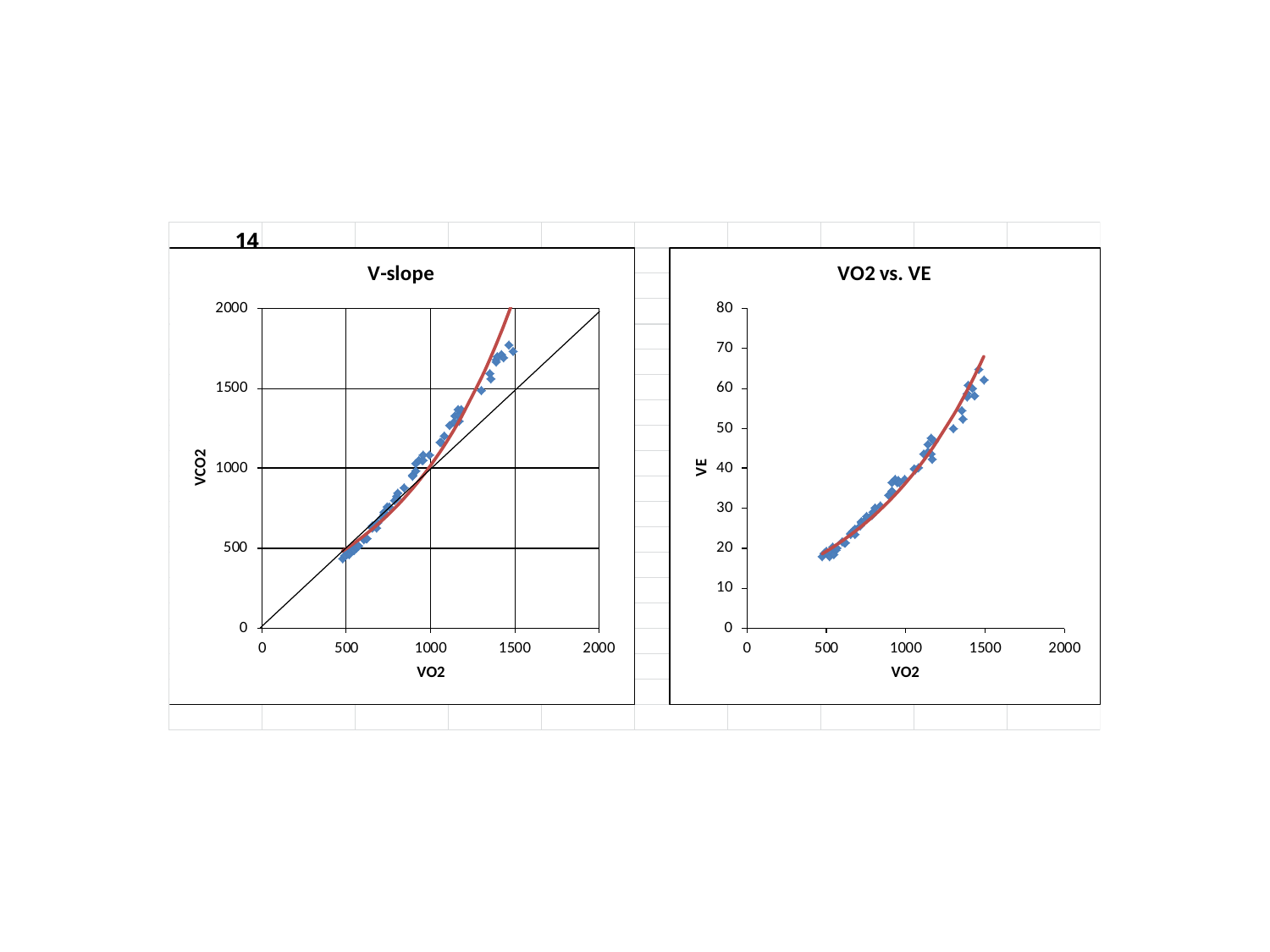

## Slide 16
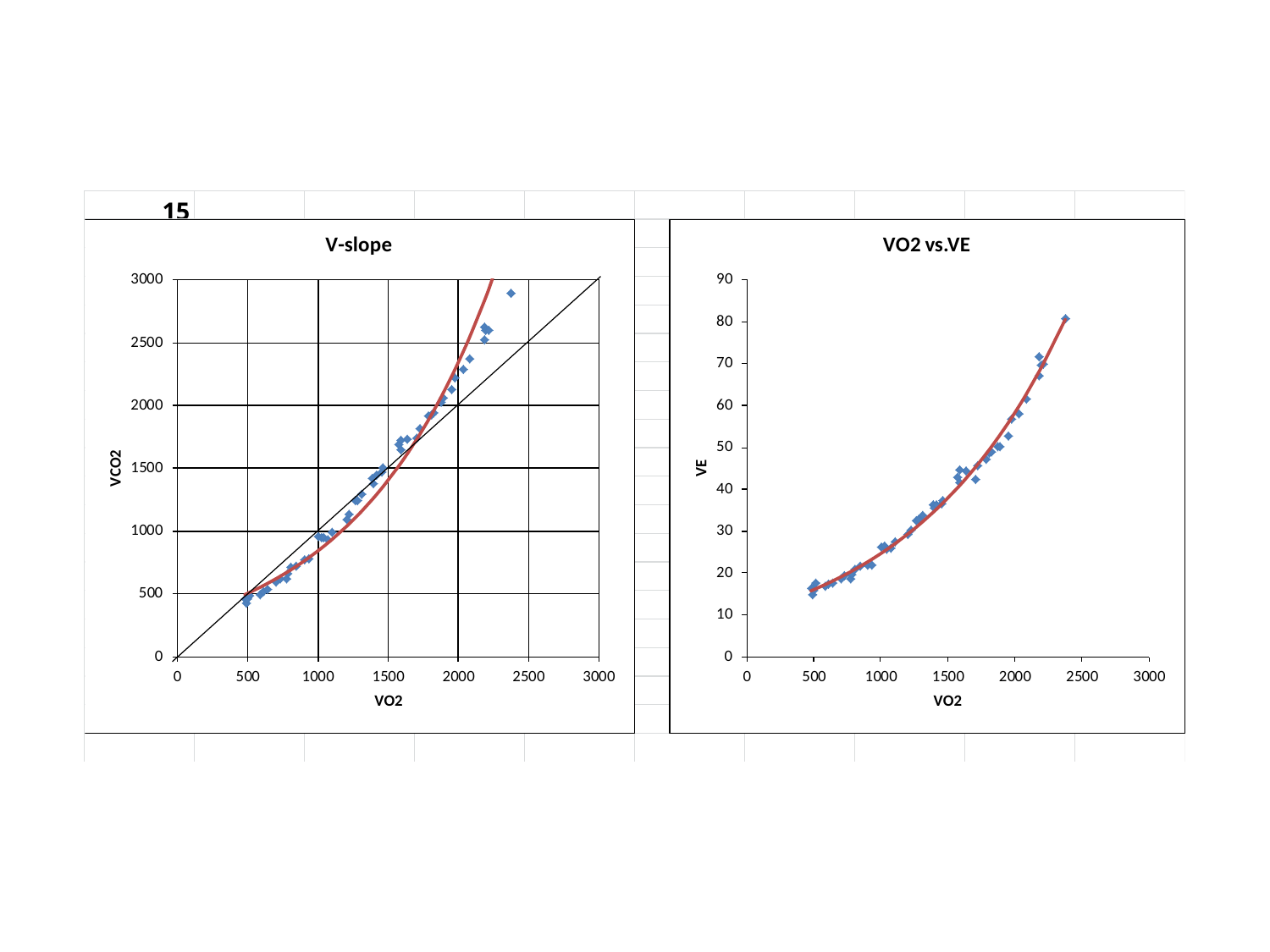

## Slide 17
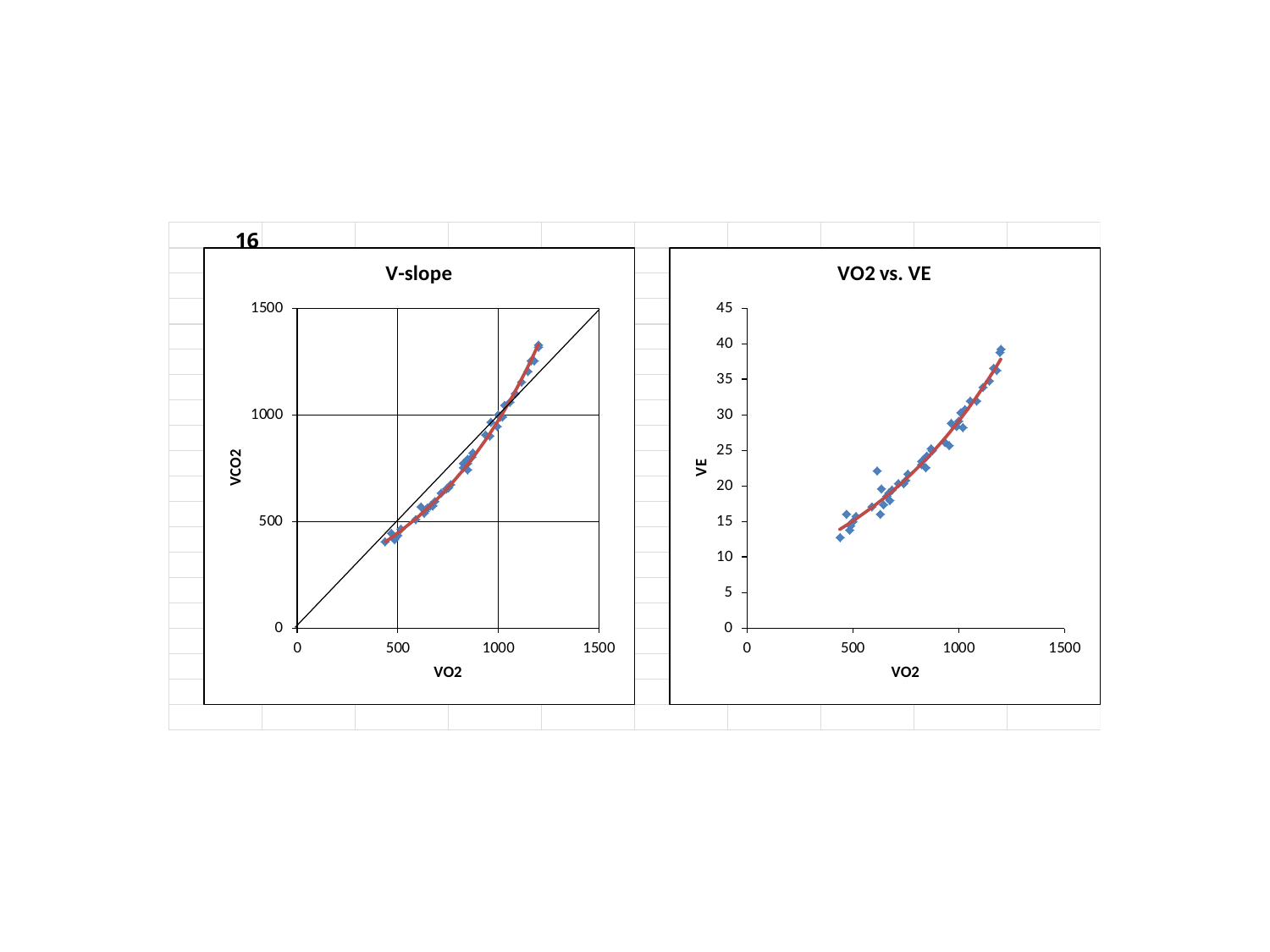

## Slide 18
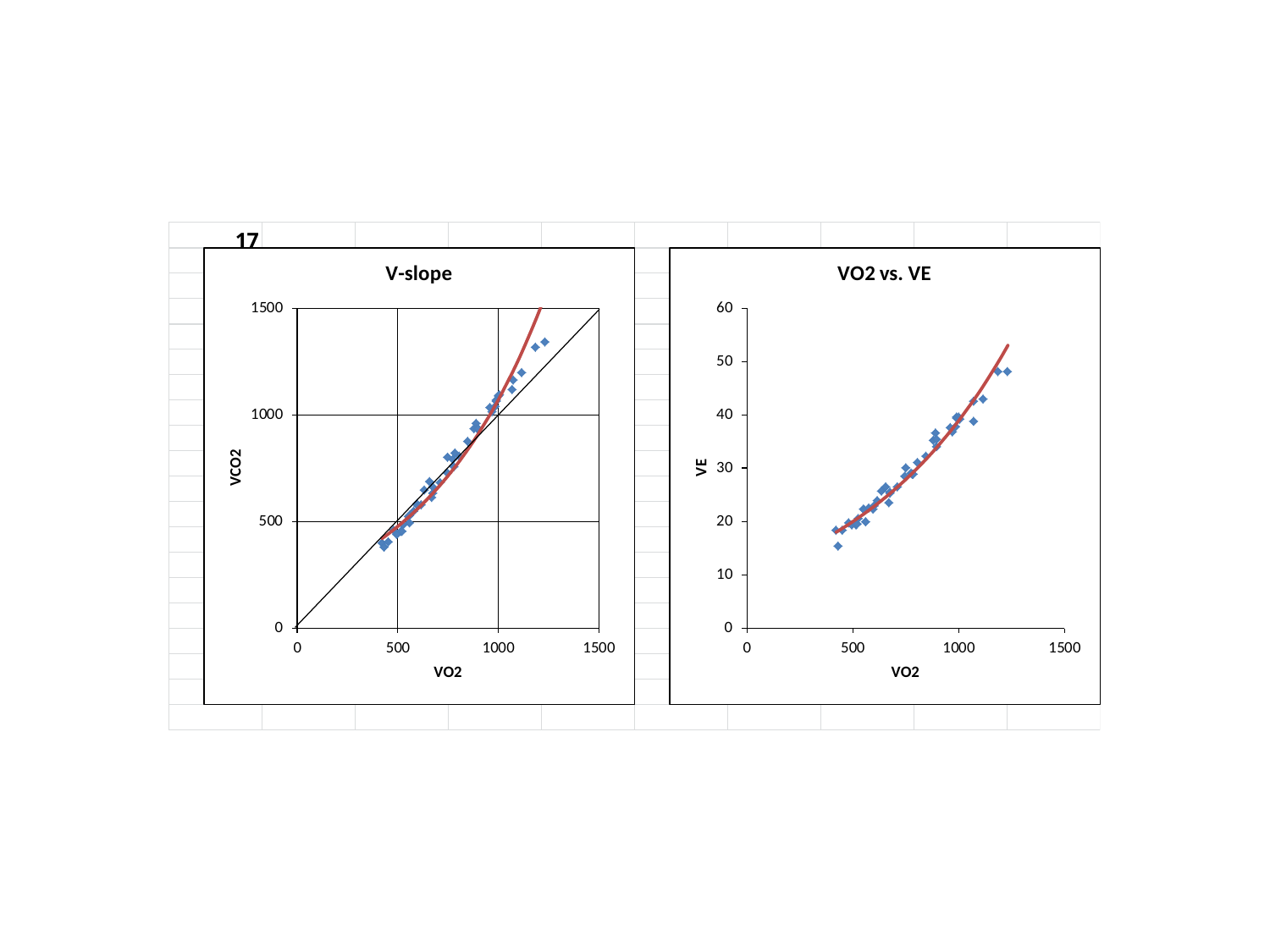

## Slide 19
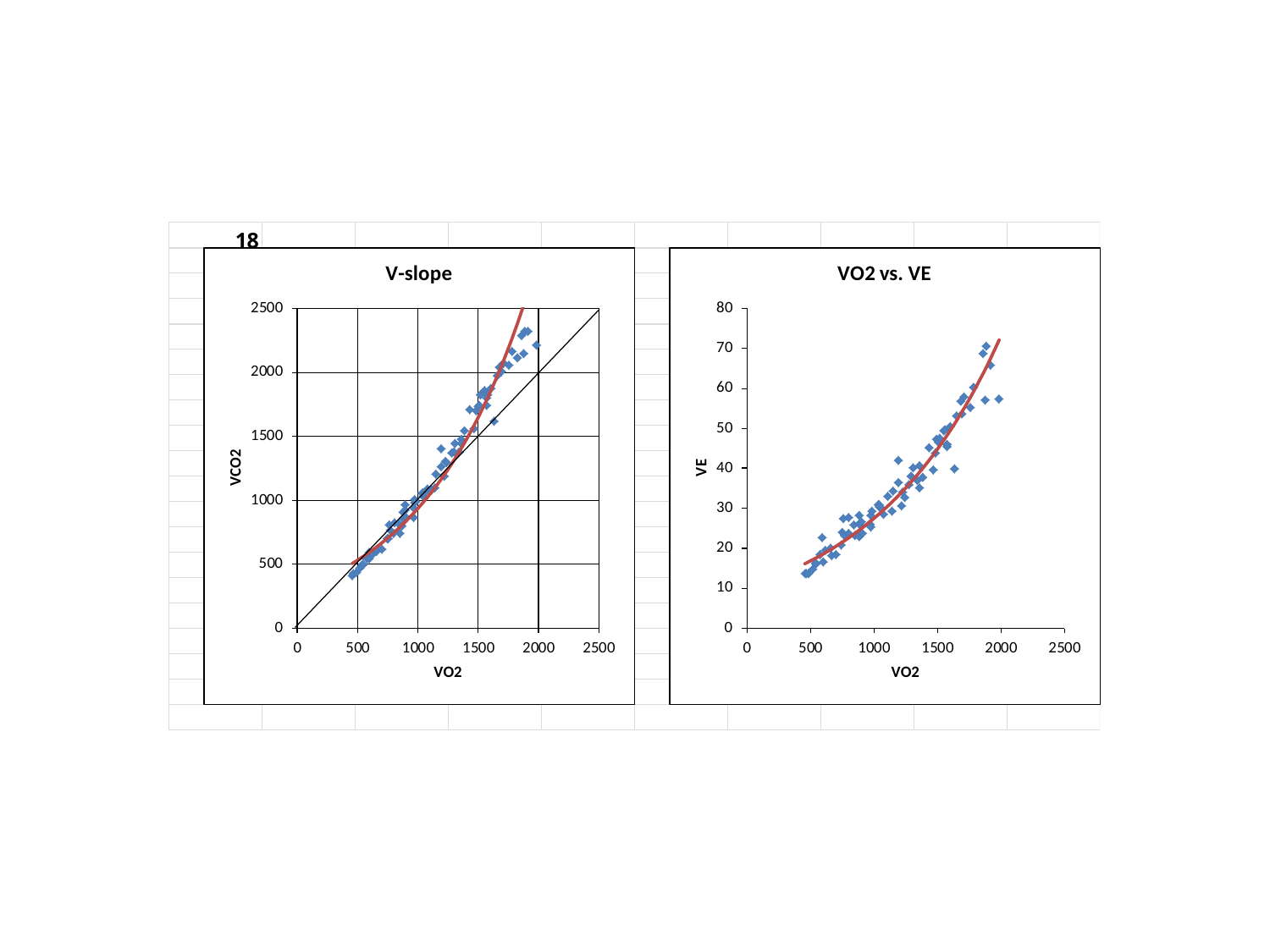

## Slide 20
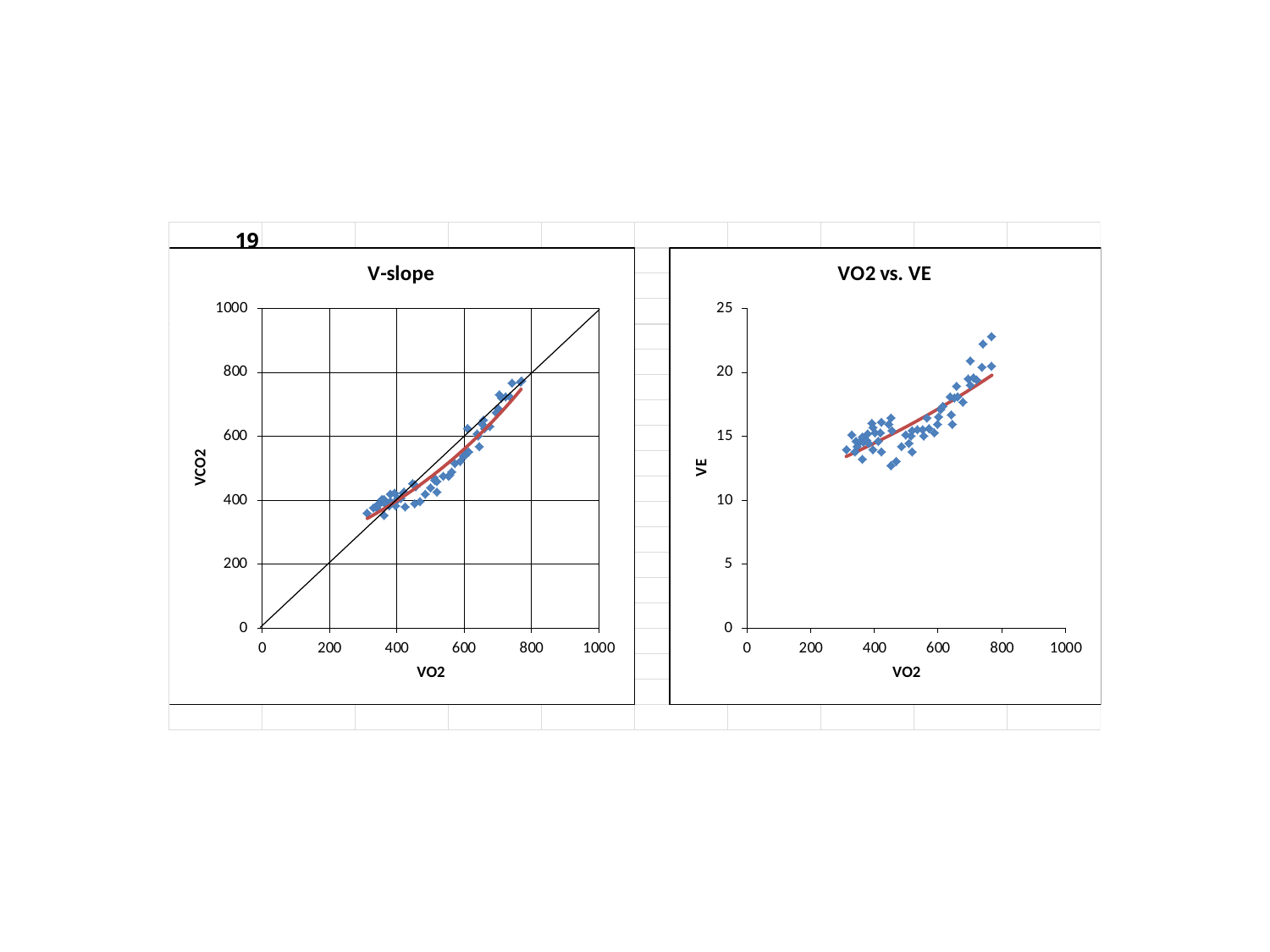

## Slide 21
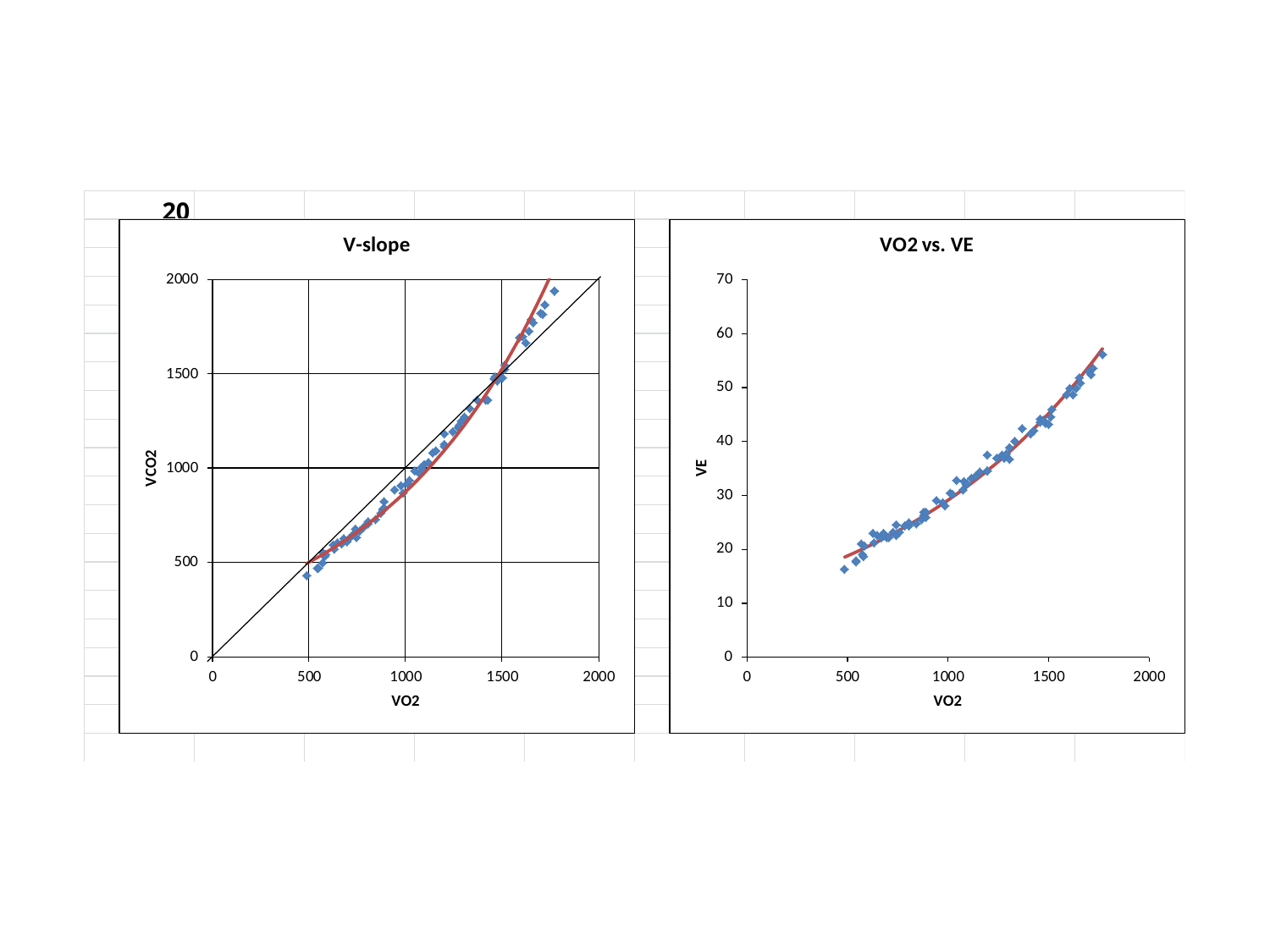

## Slide 22
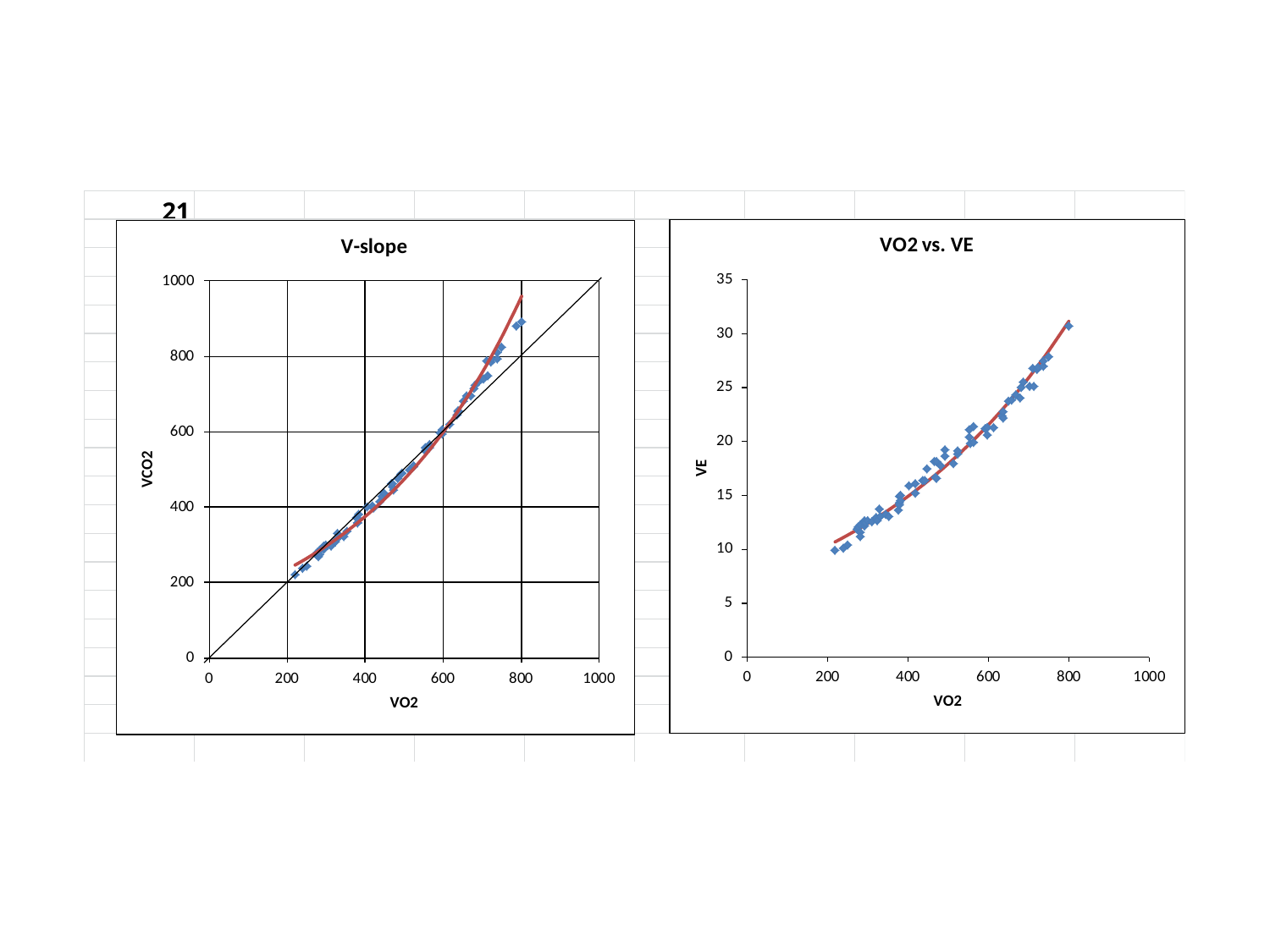

## Slide 23
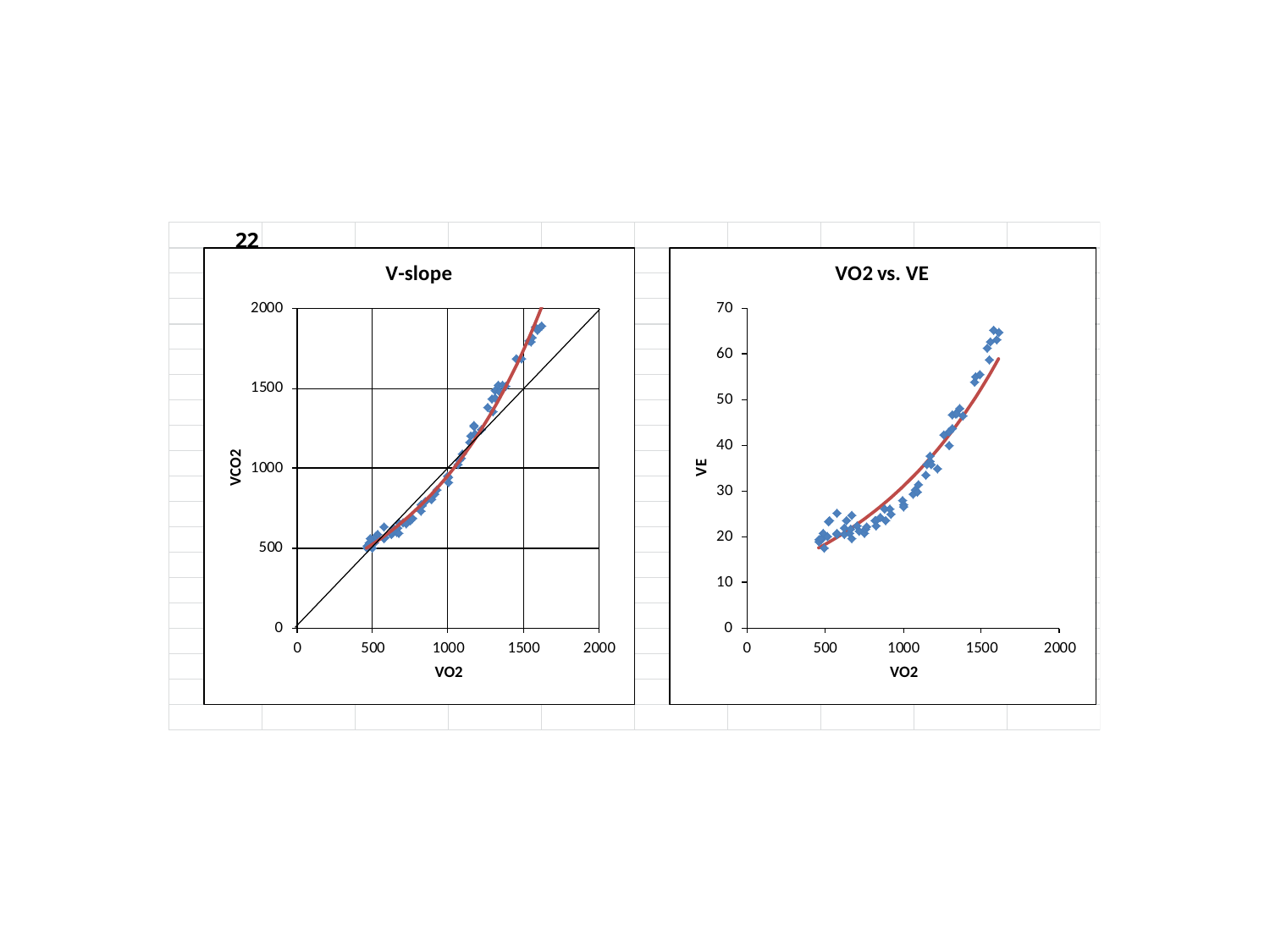

## Slide 24
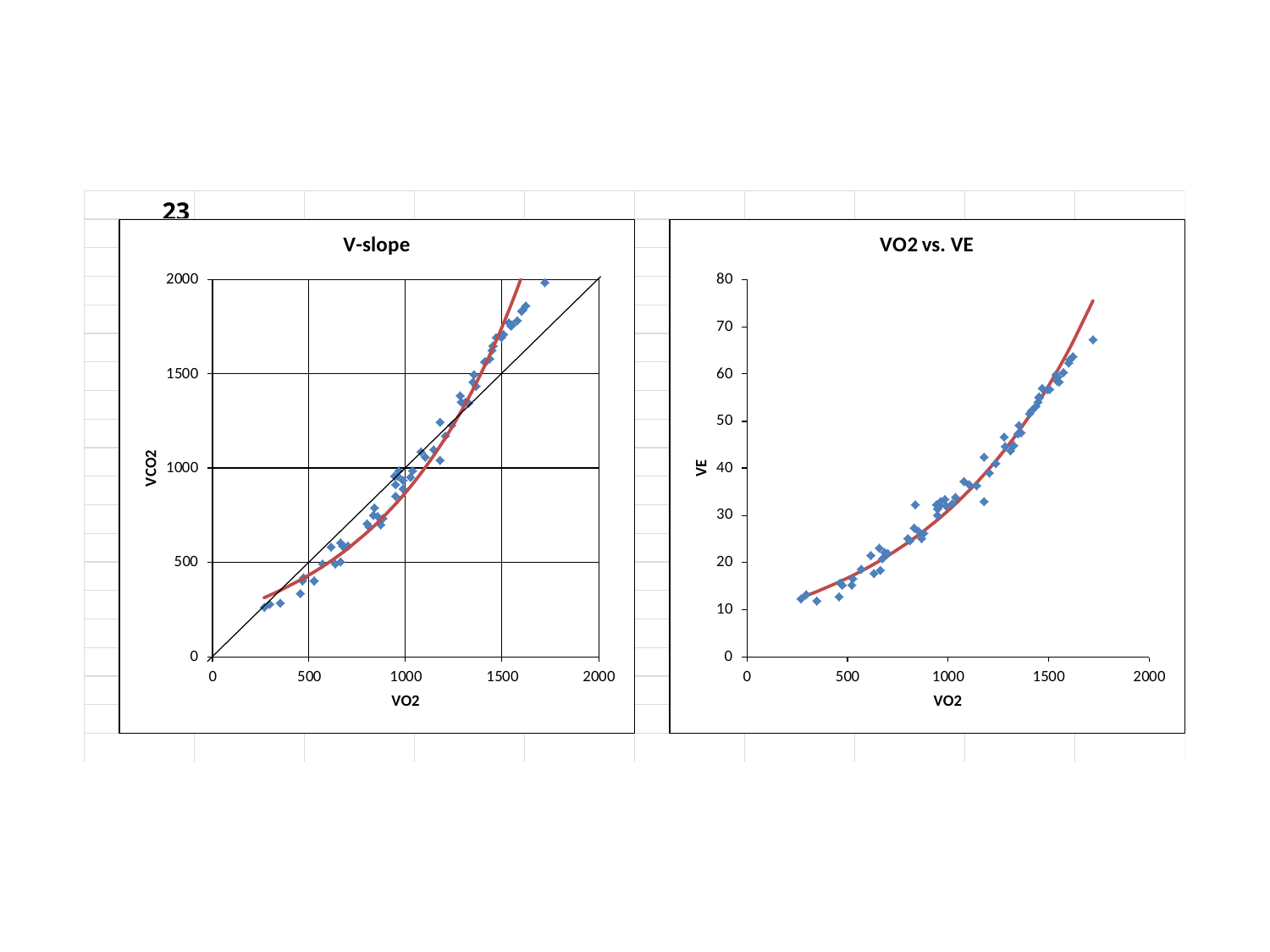

## Slide 25
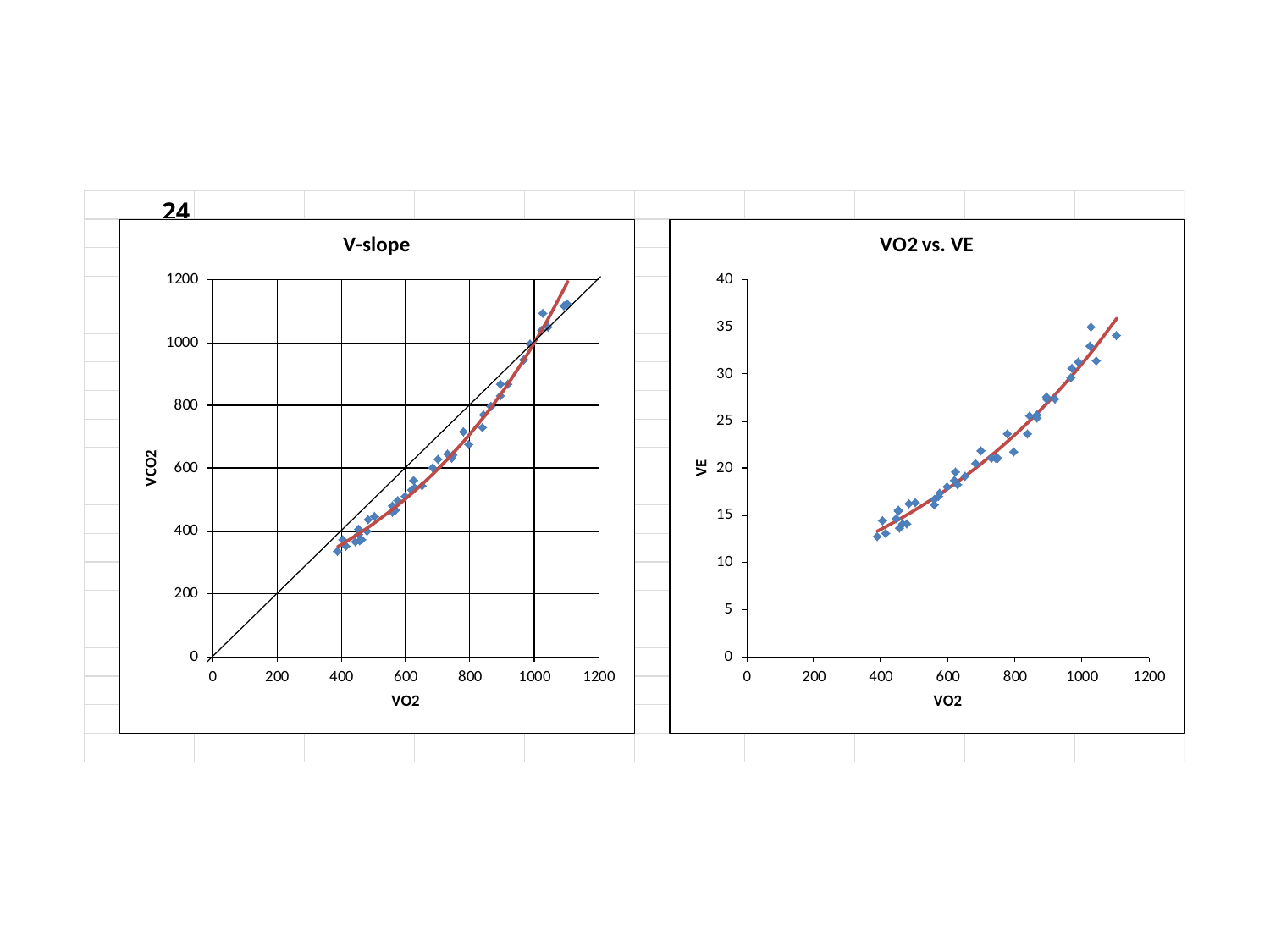

## Slide 26
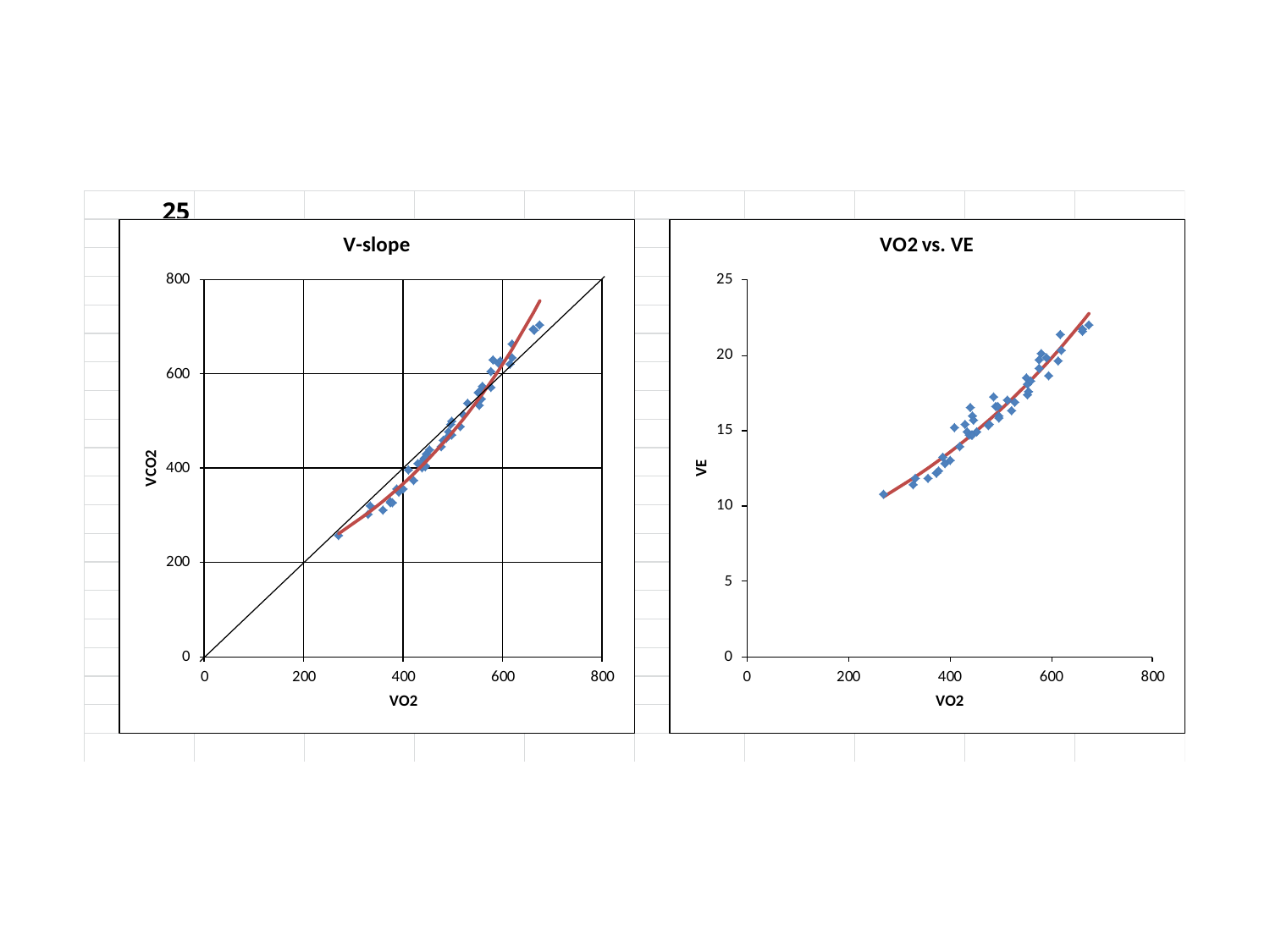

## Slide 27
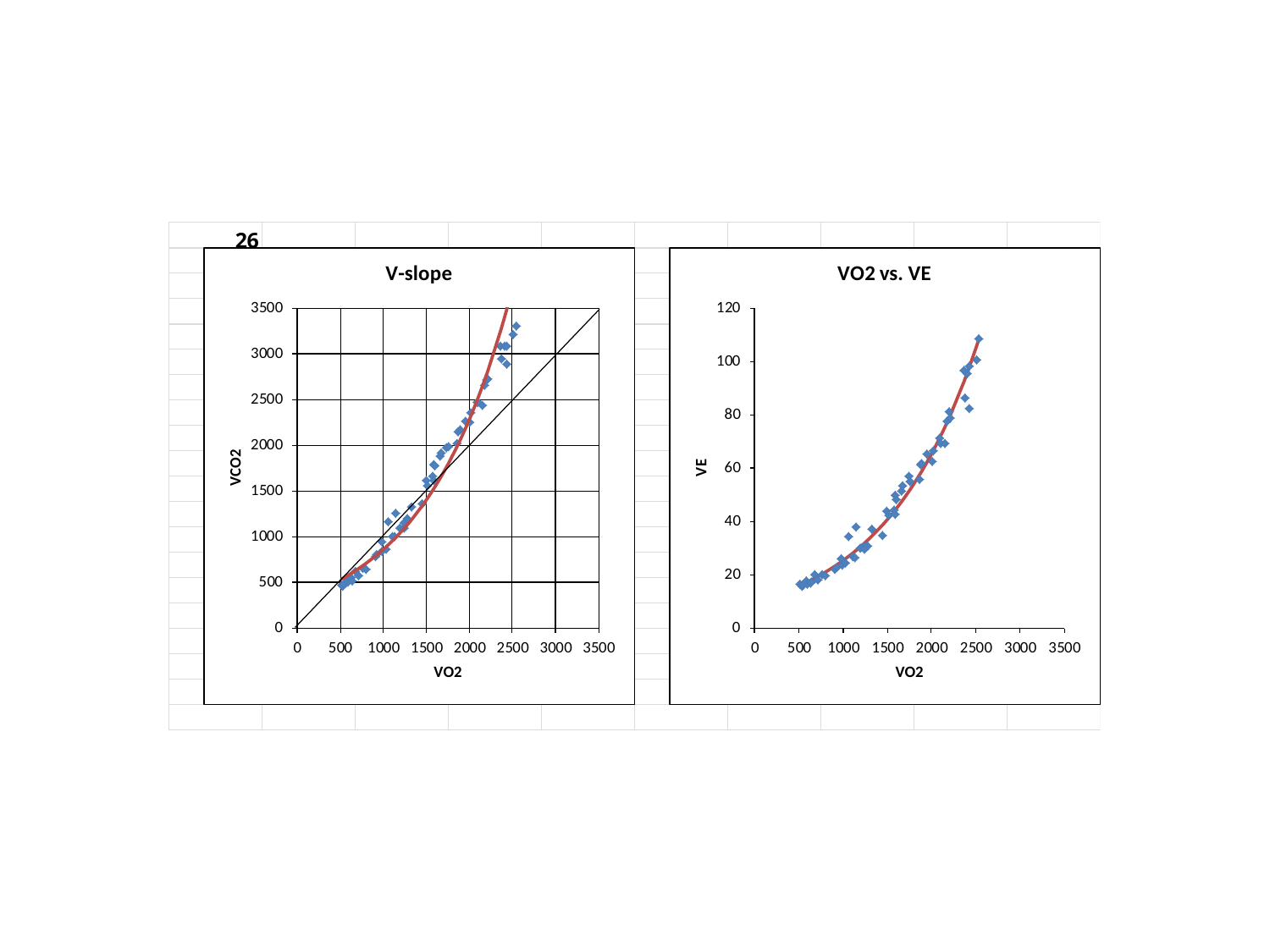

## Slide 28
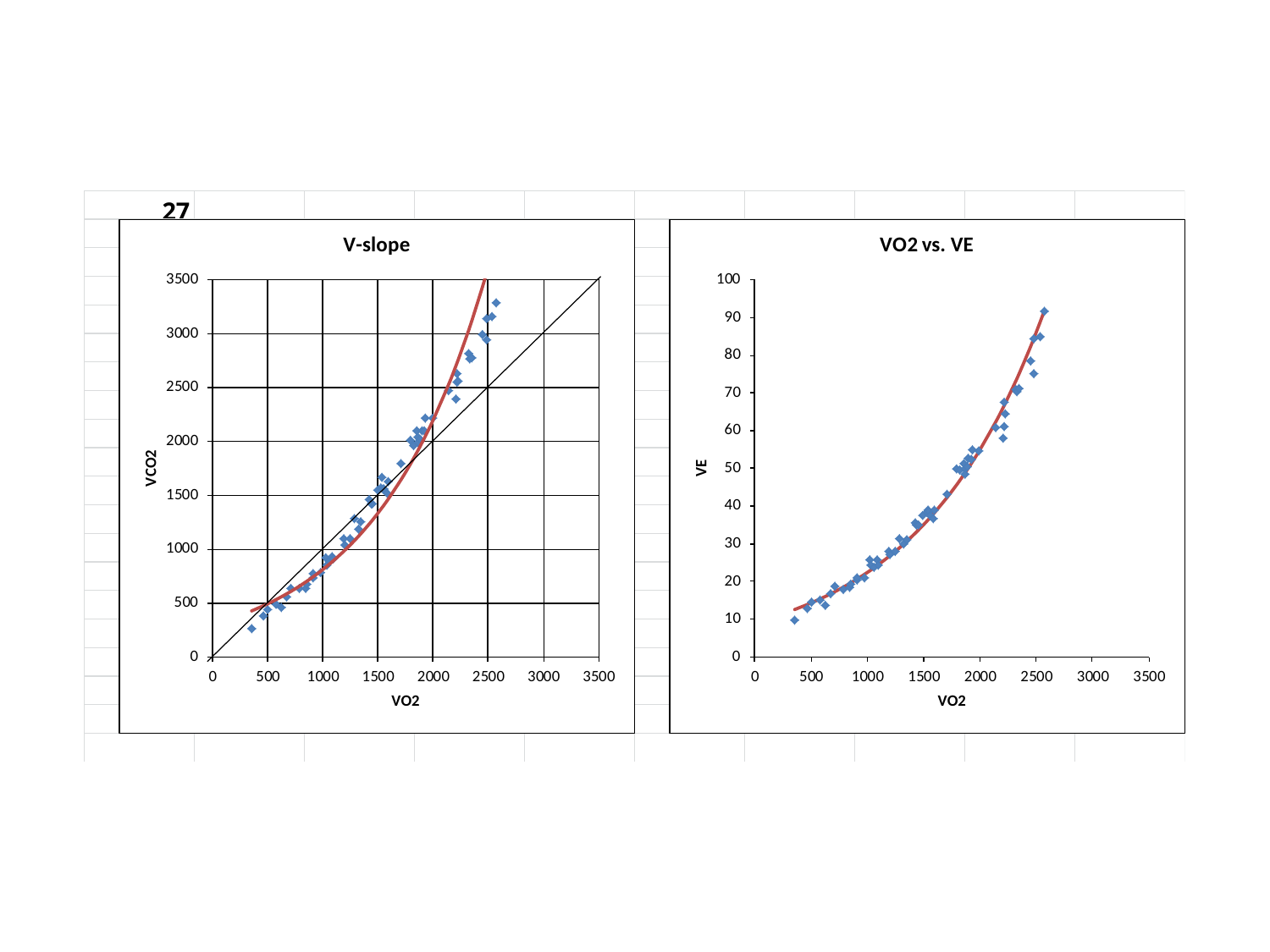

## Slide 29
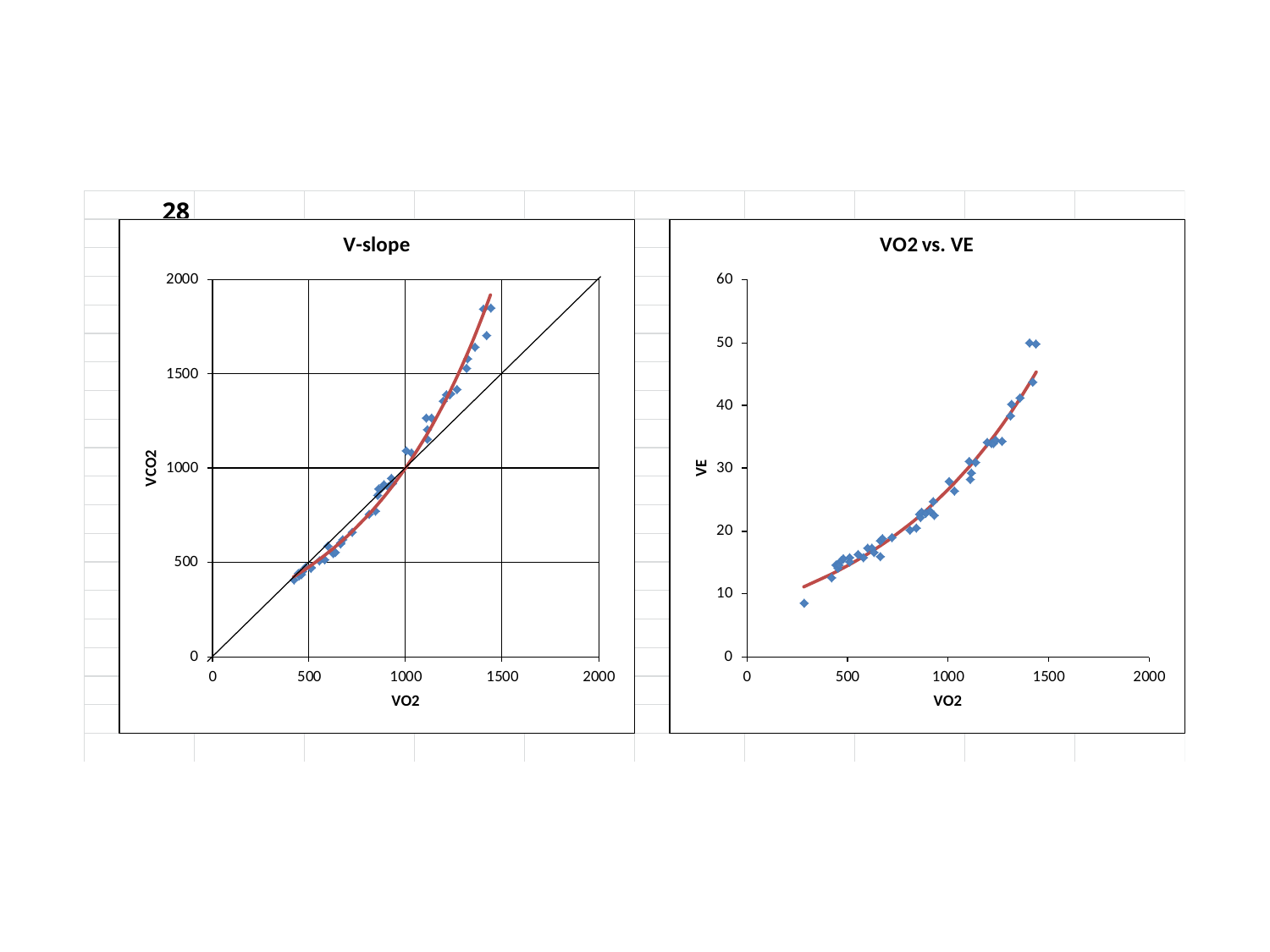

## Slide 30
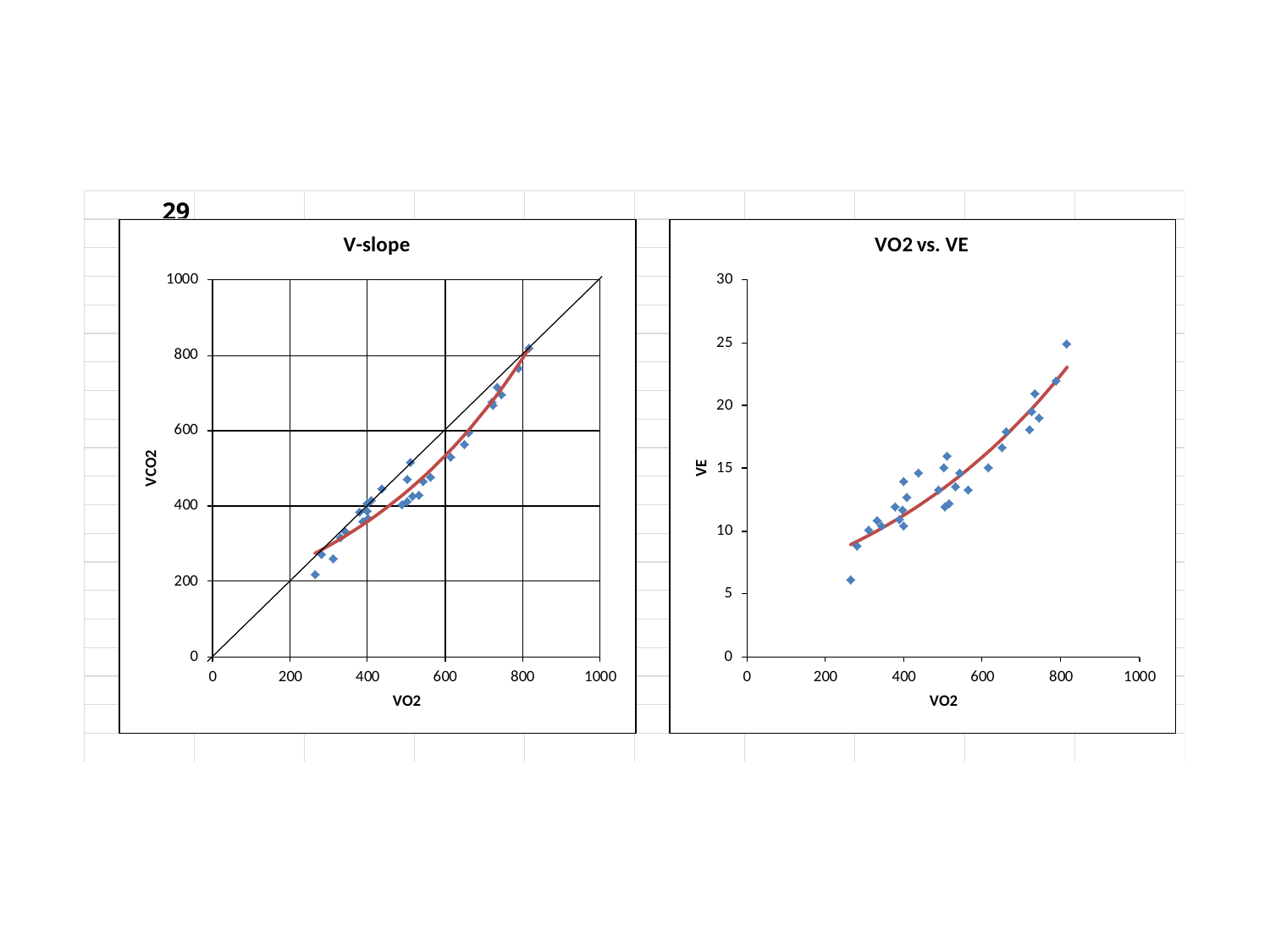

## Slide 31
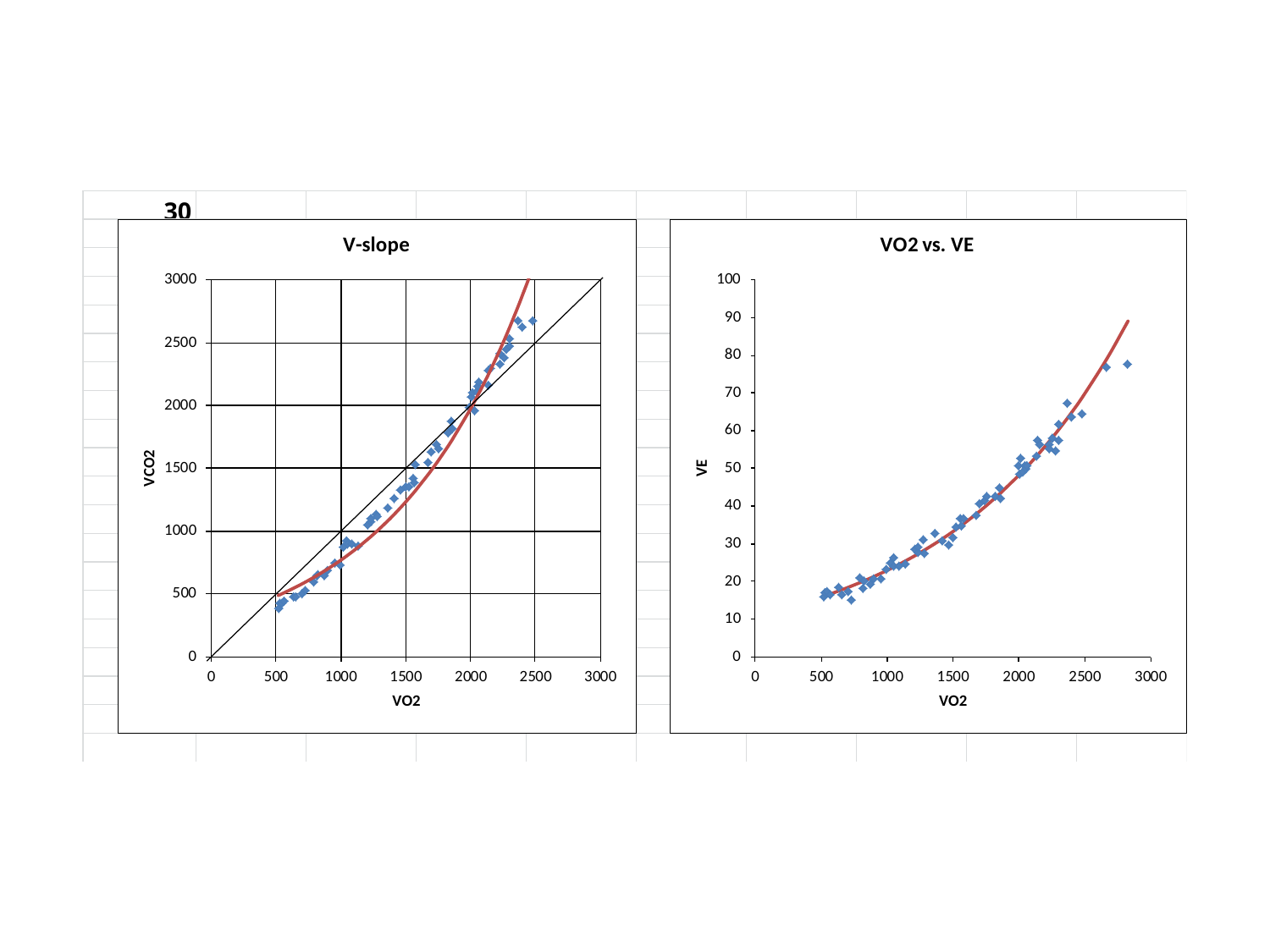

## Slide 32
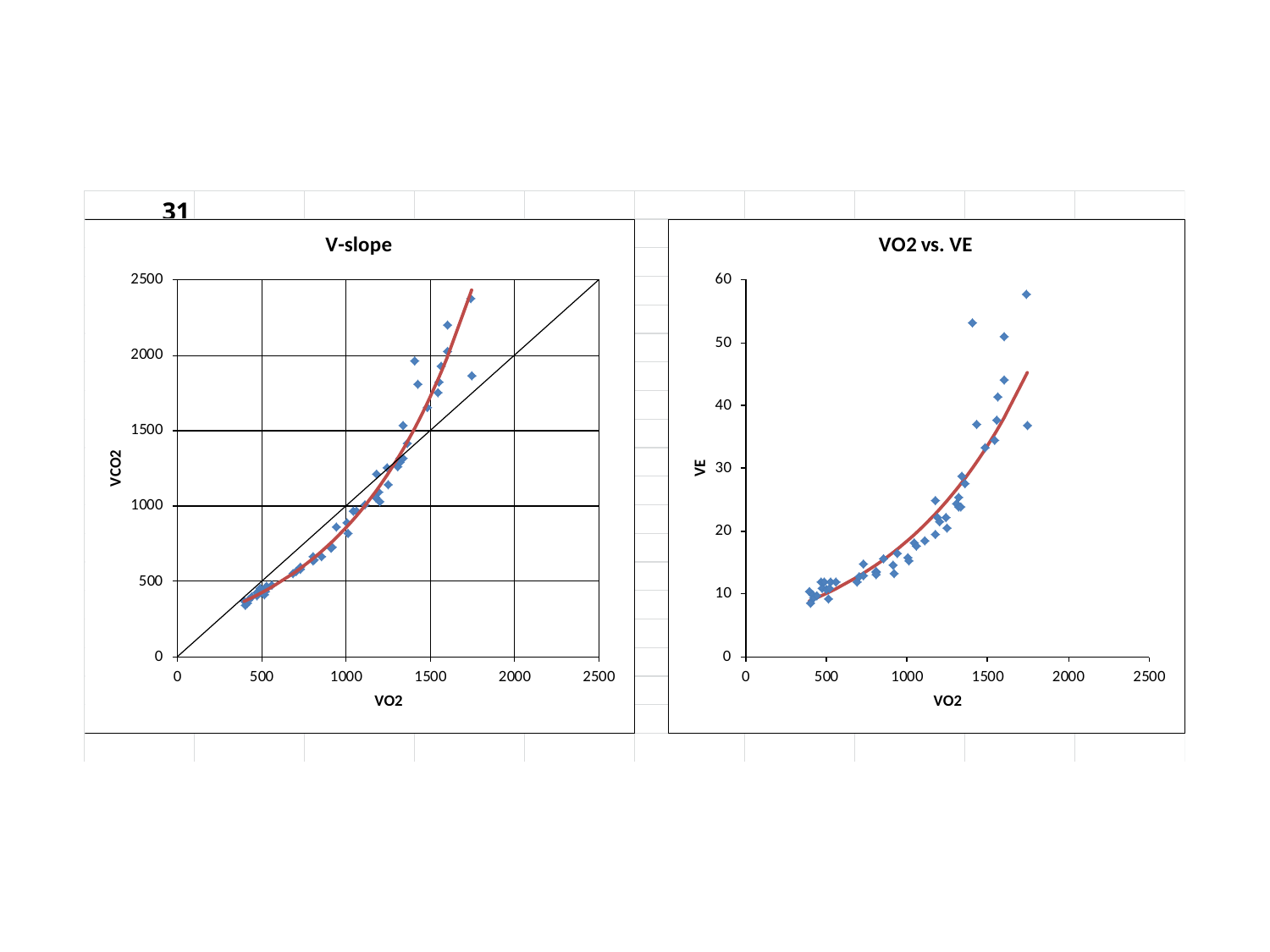

## Slide 33
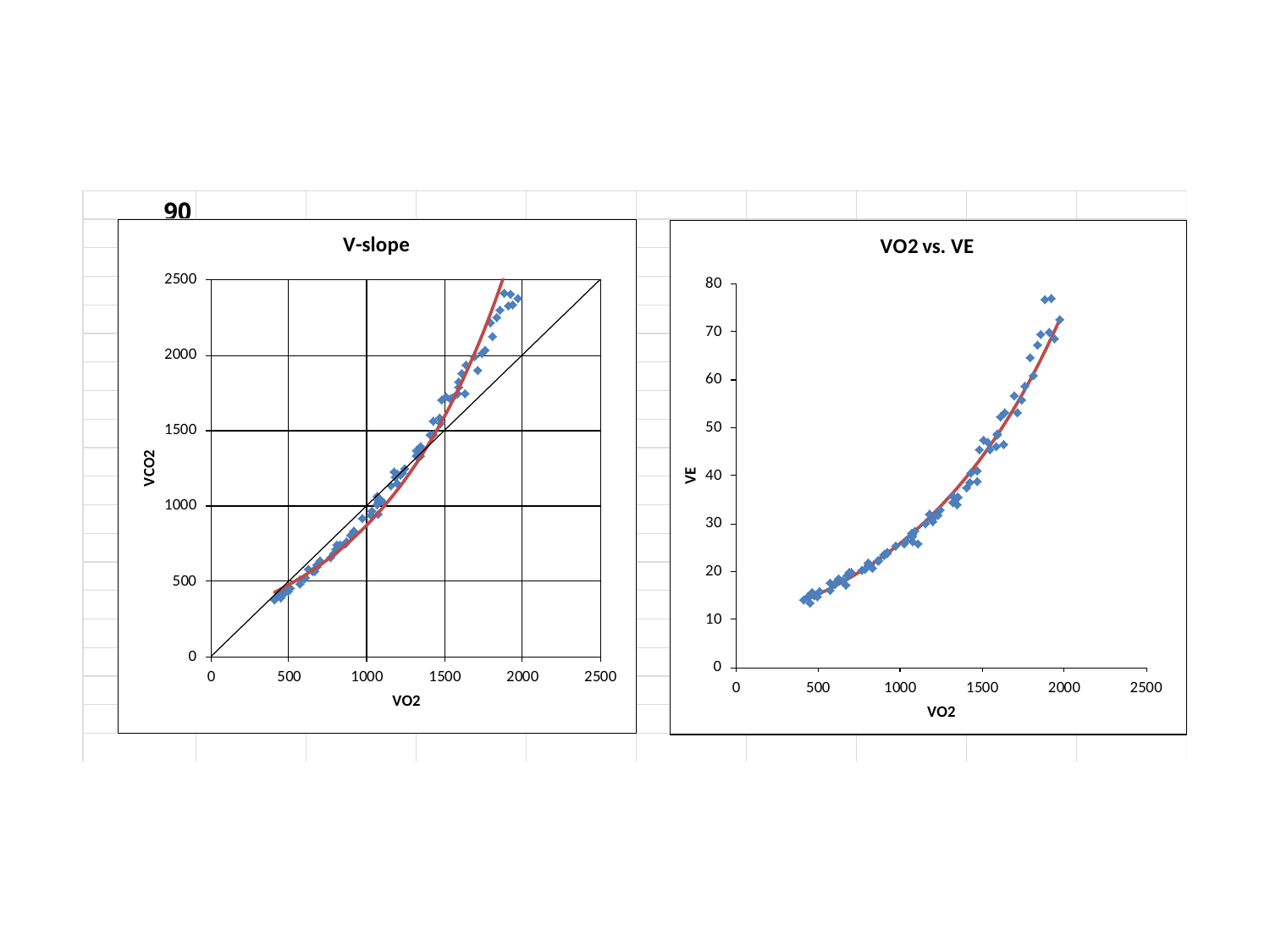

## Slide 34
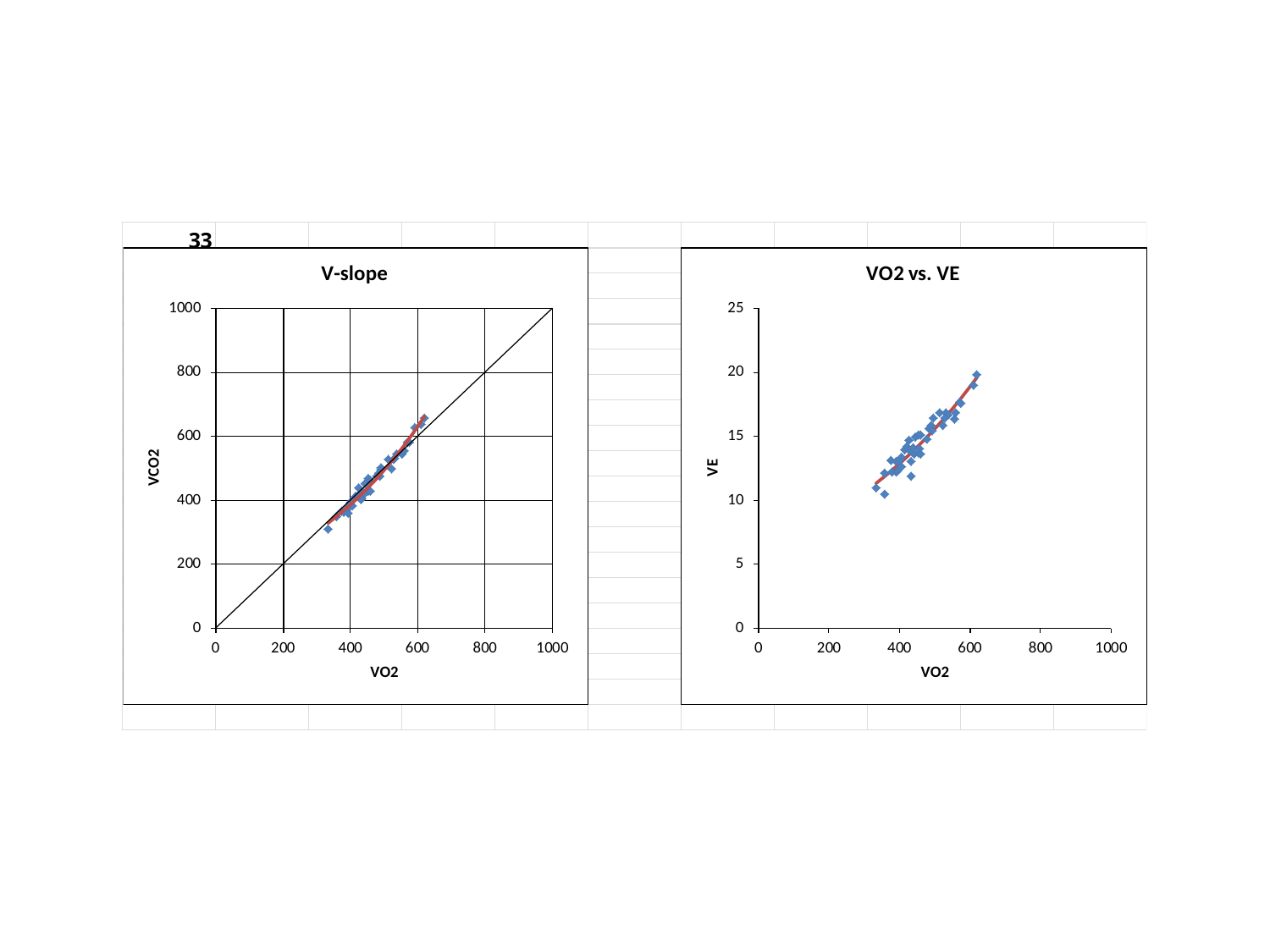

## Slide 35
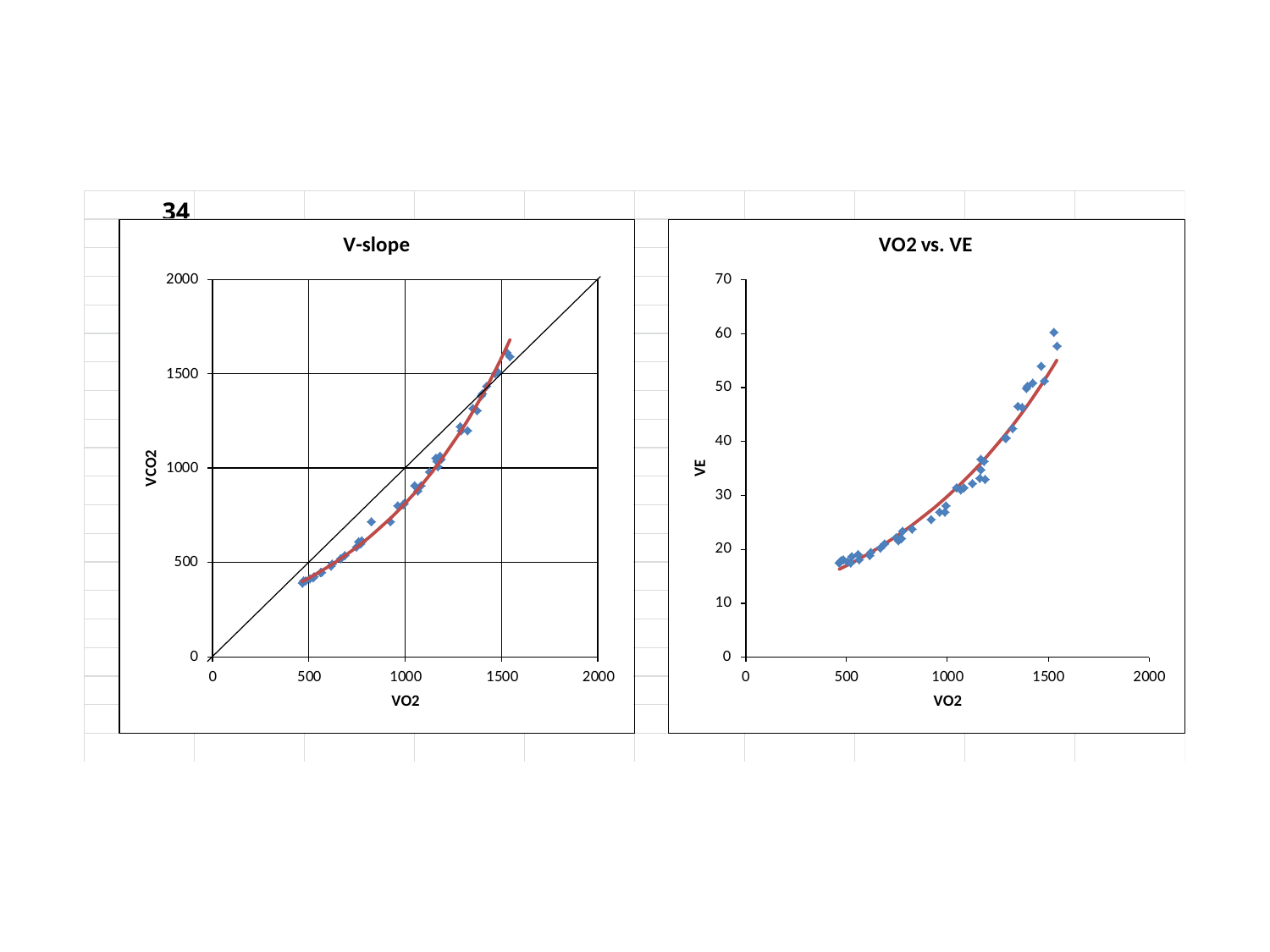

## Slide 36
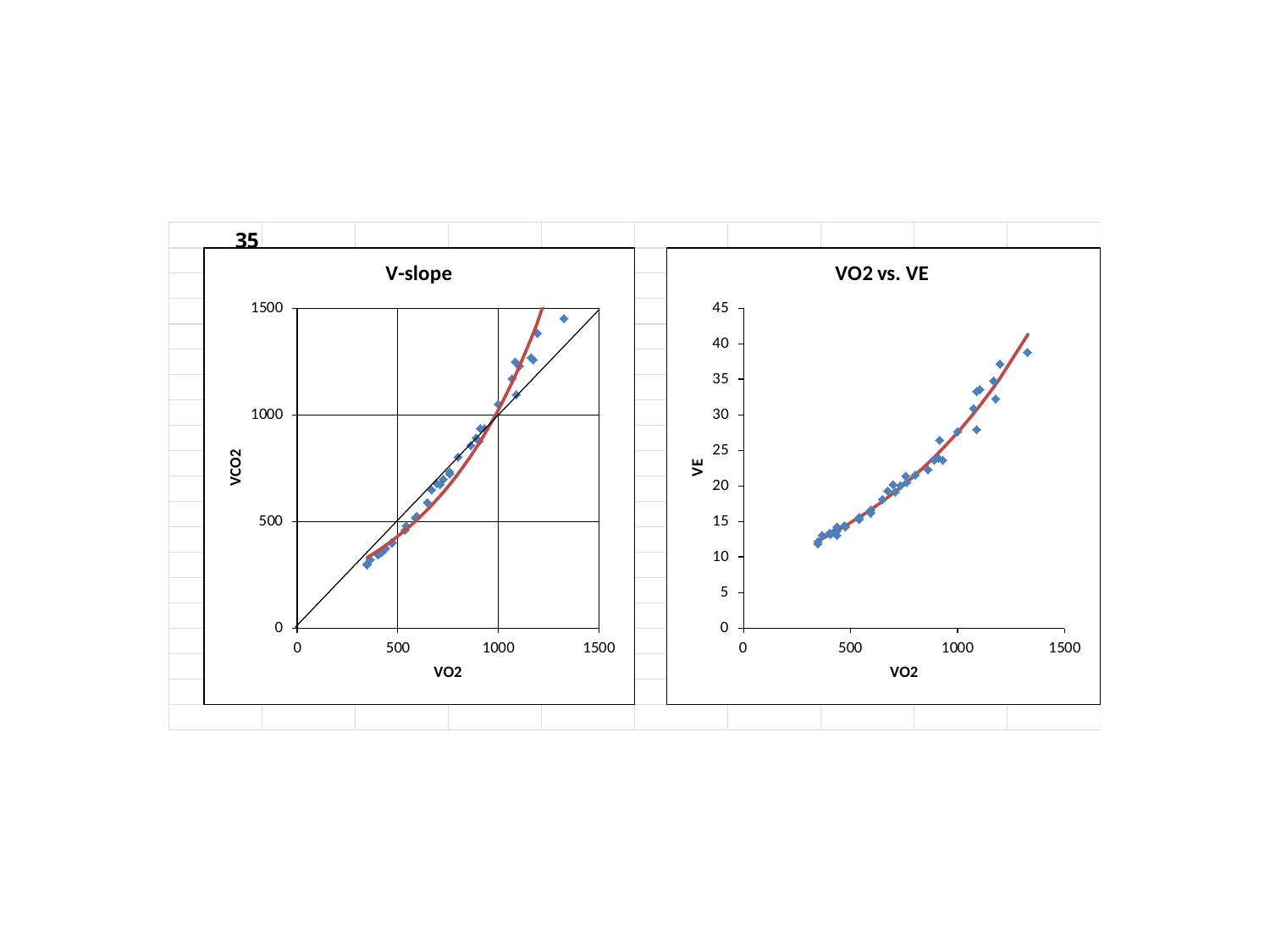

## Slide 37
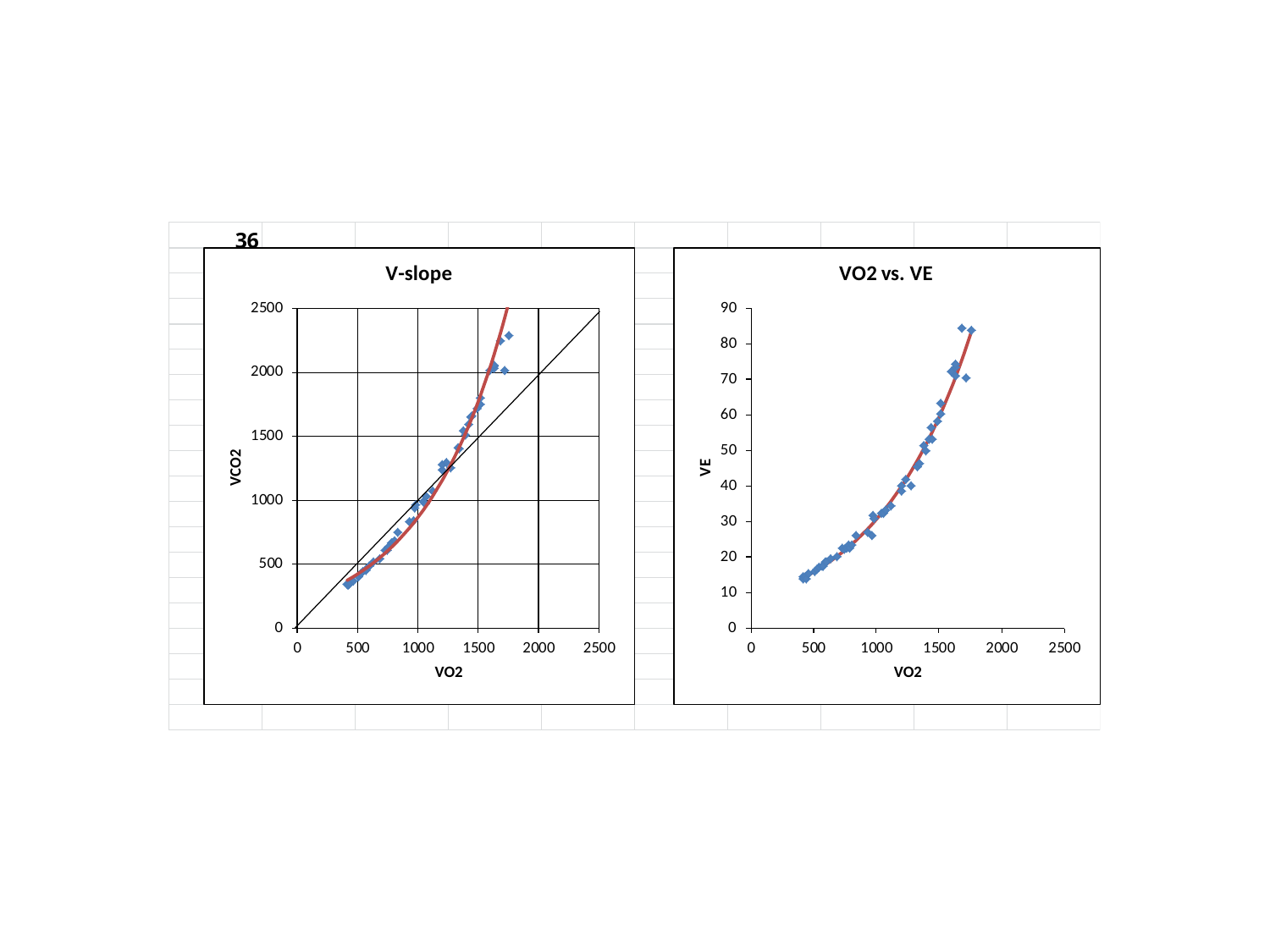

## Slide 38
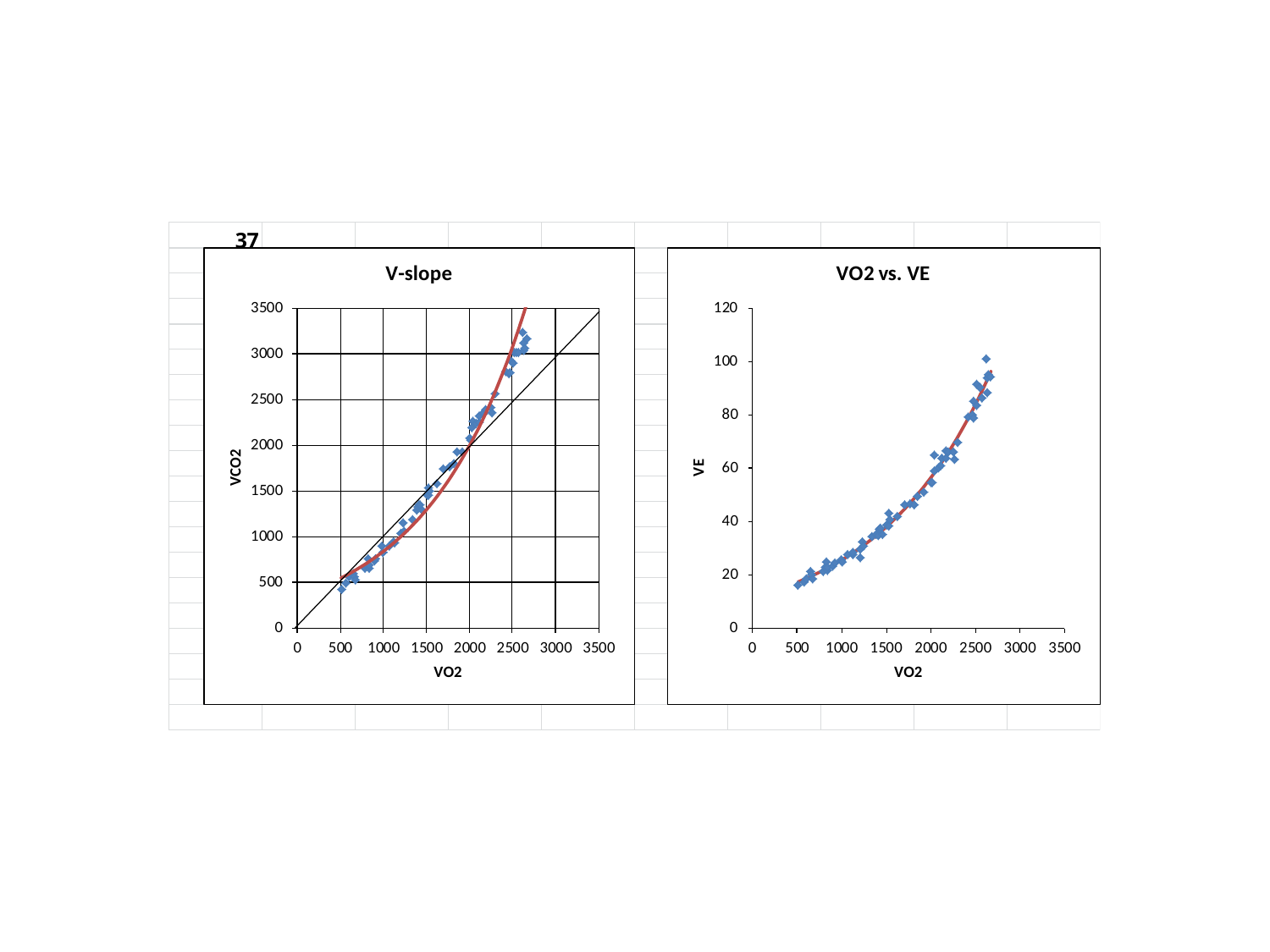

## Slide 39
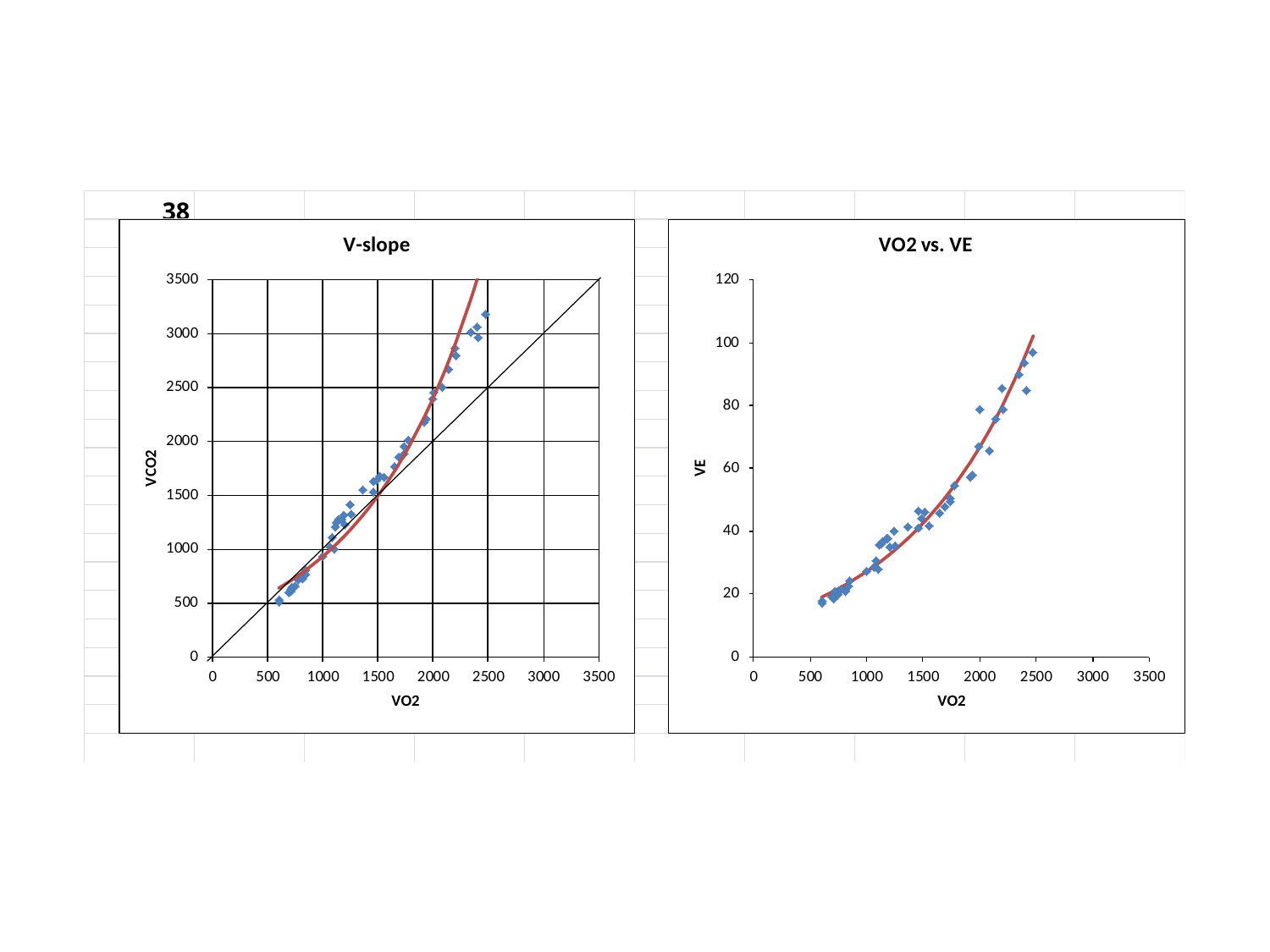

## Slide 40
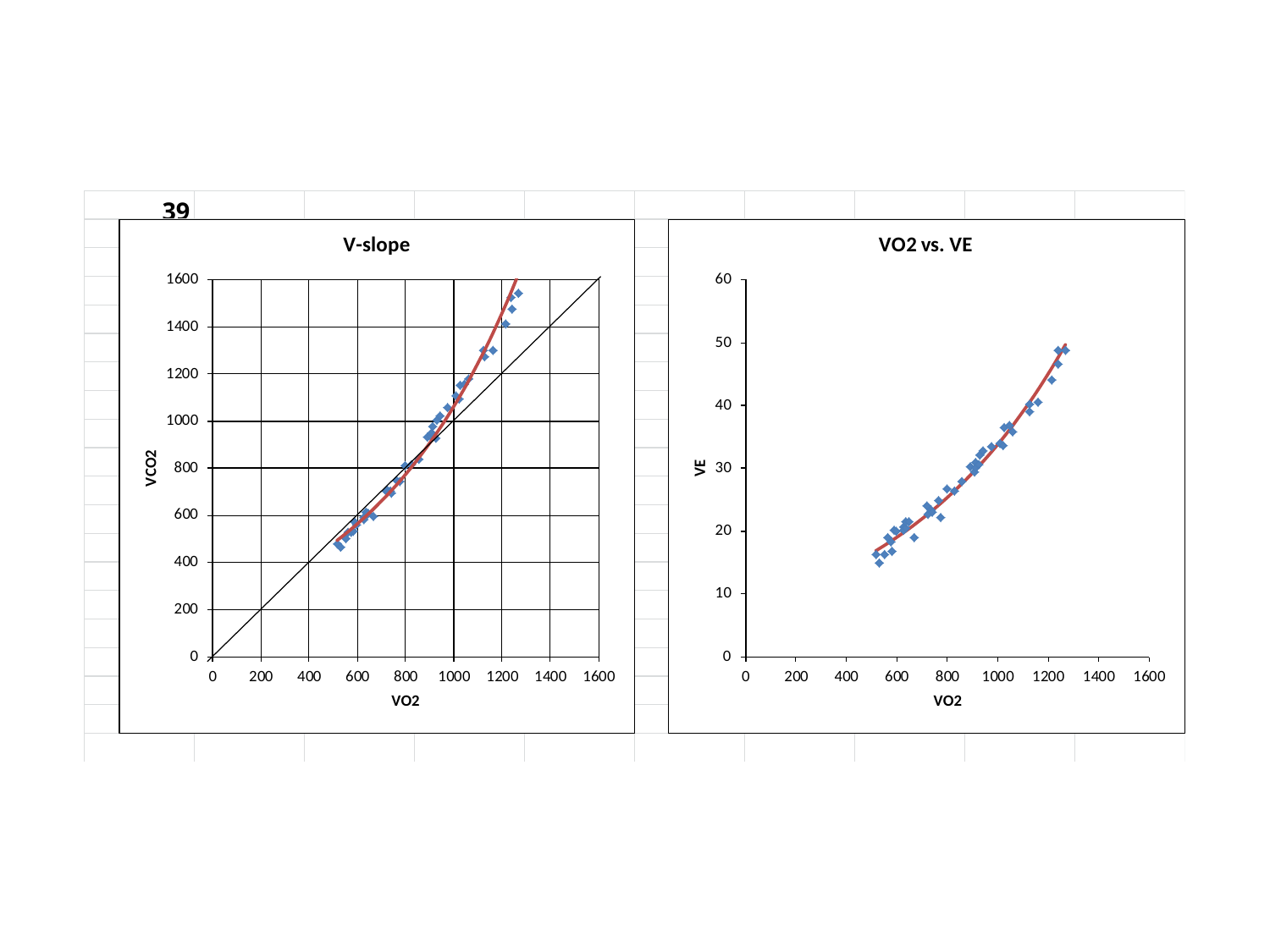

## Slide 41
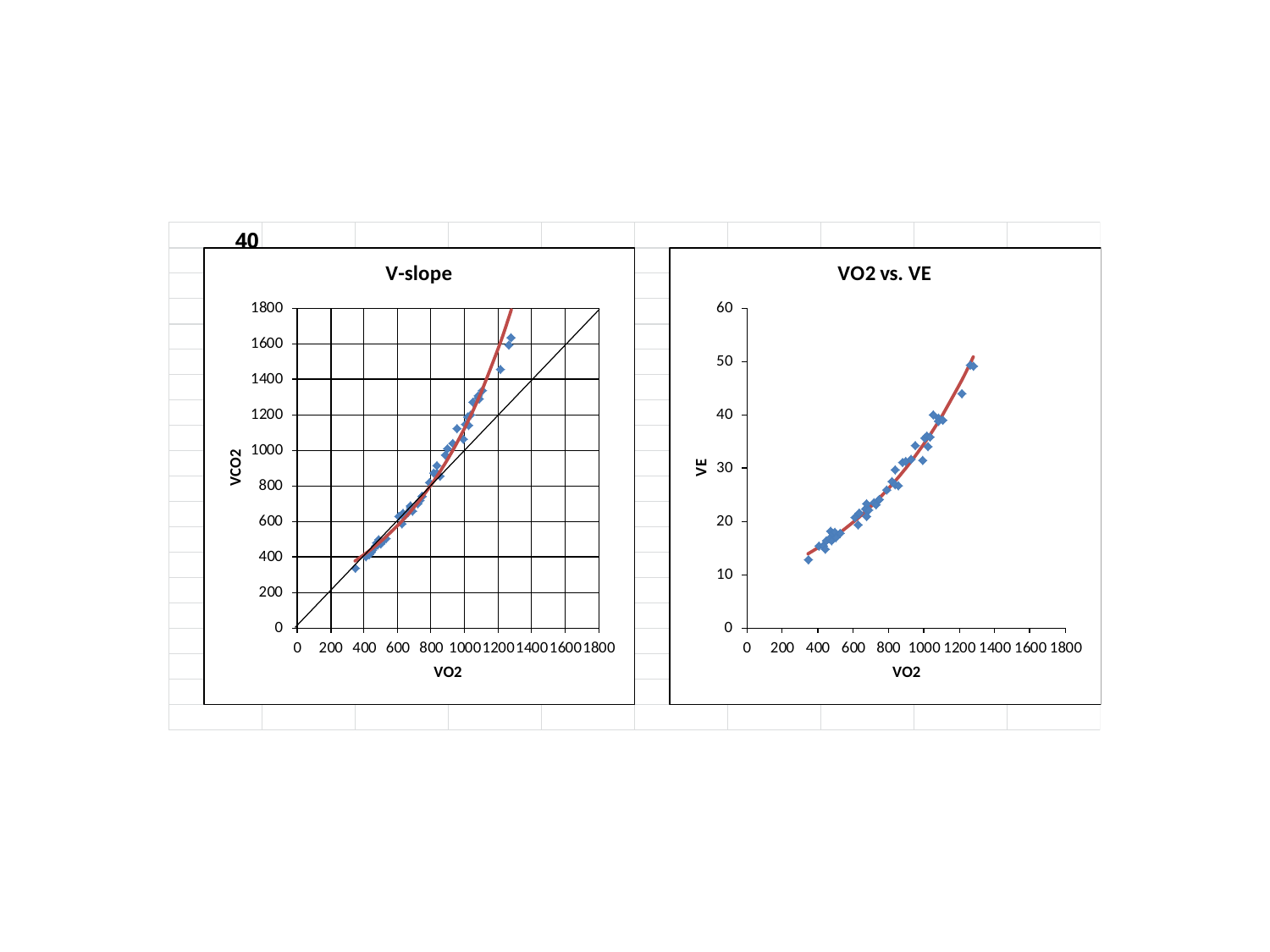

## Slide 42
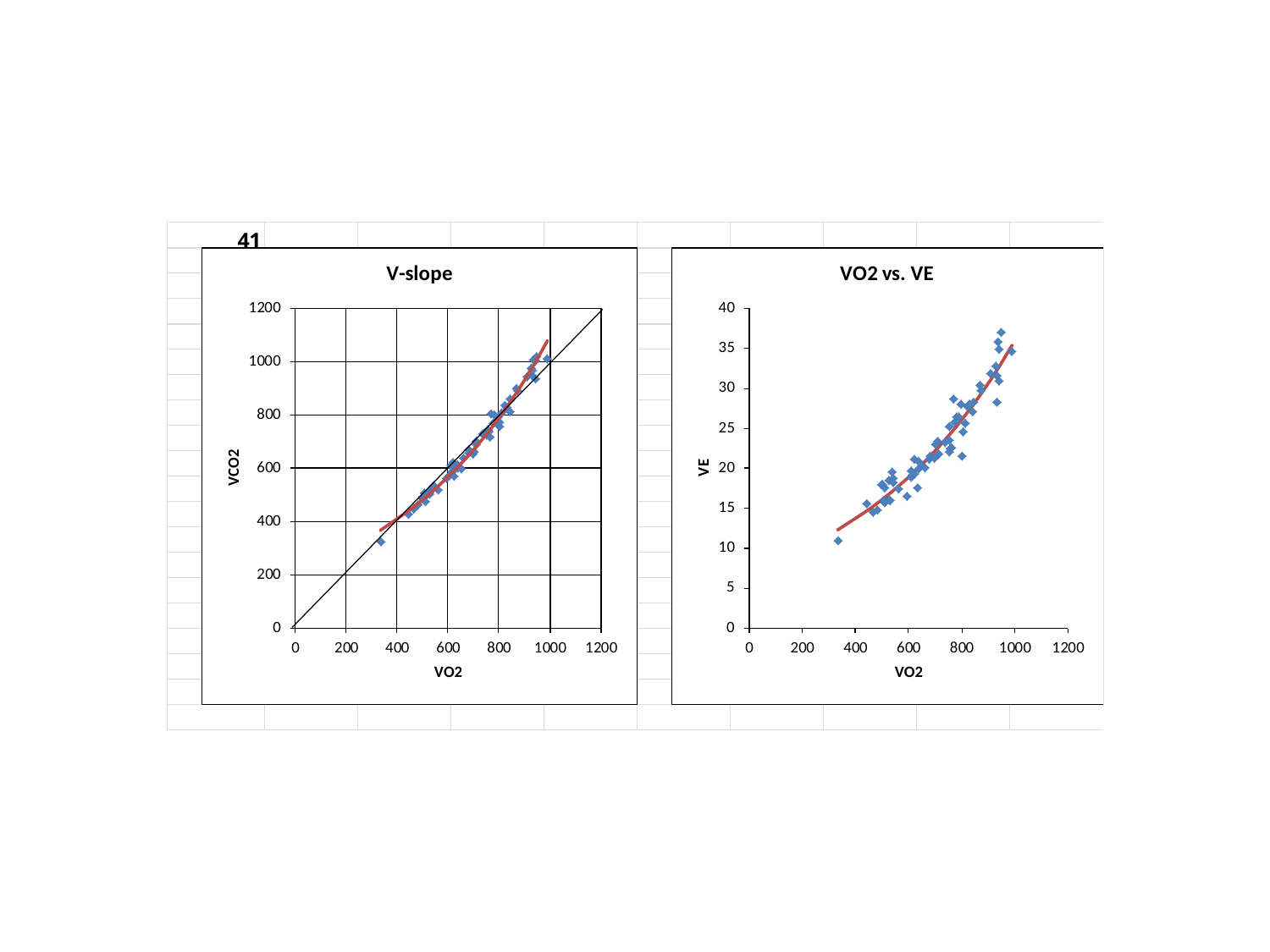

## Slide 43
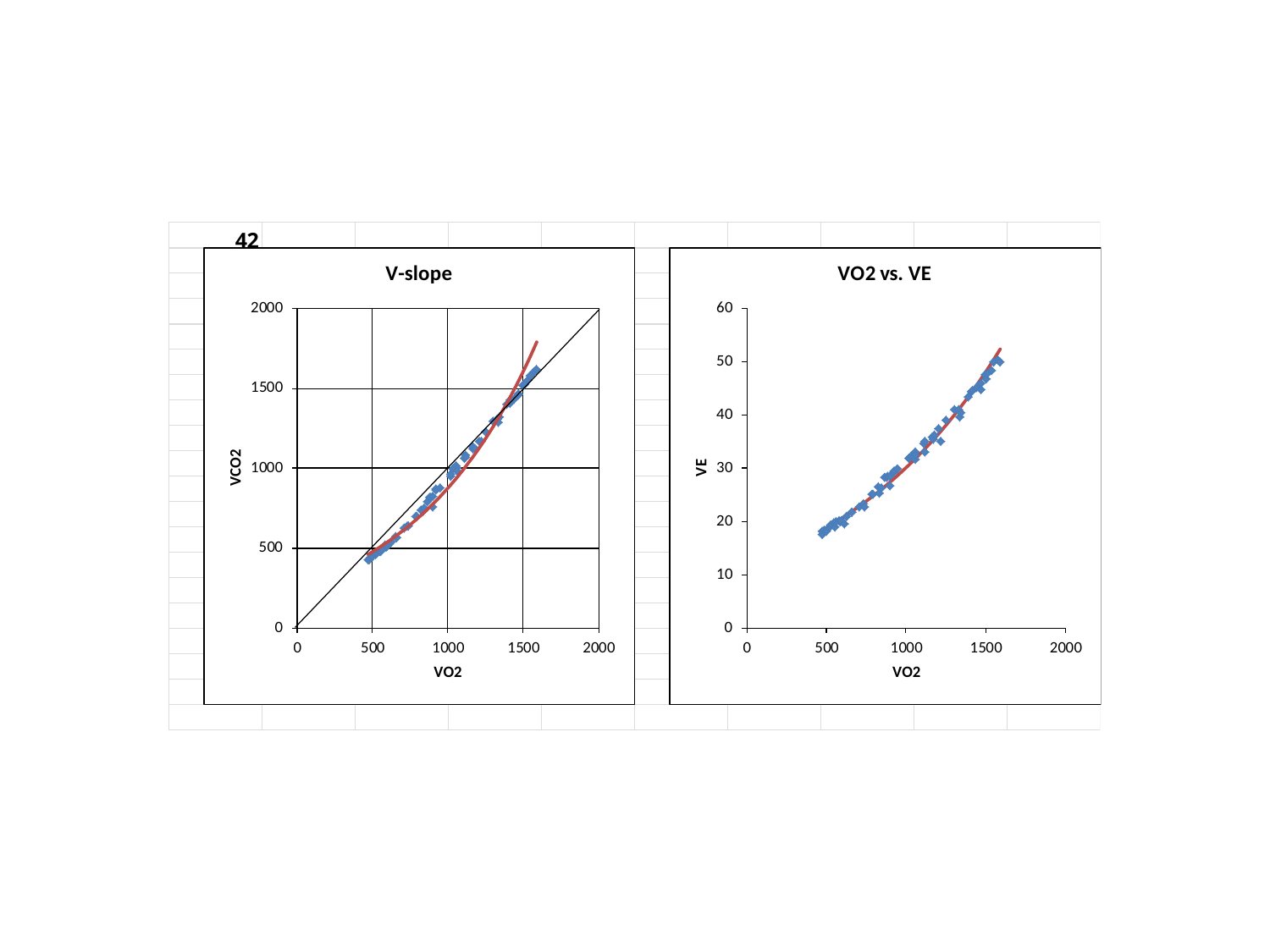

## Slide 44
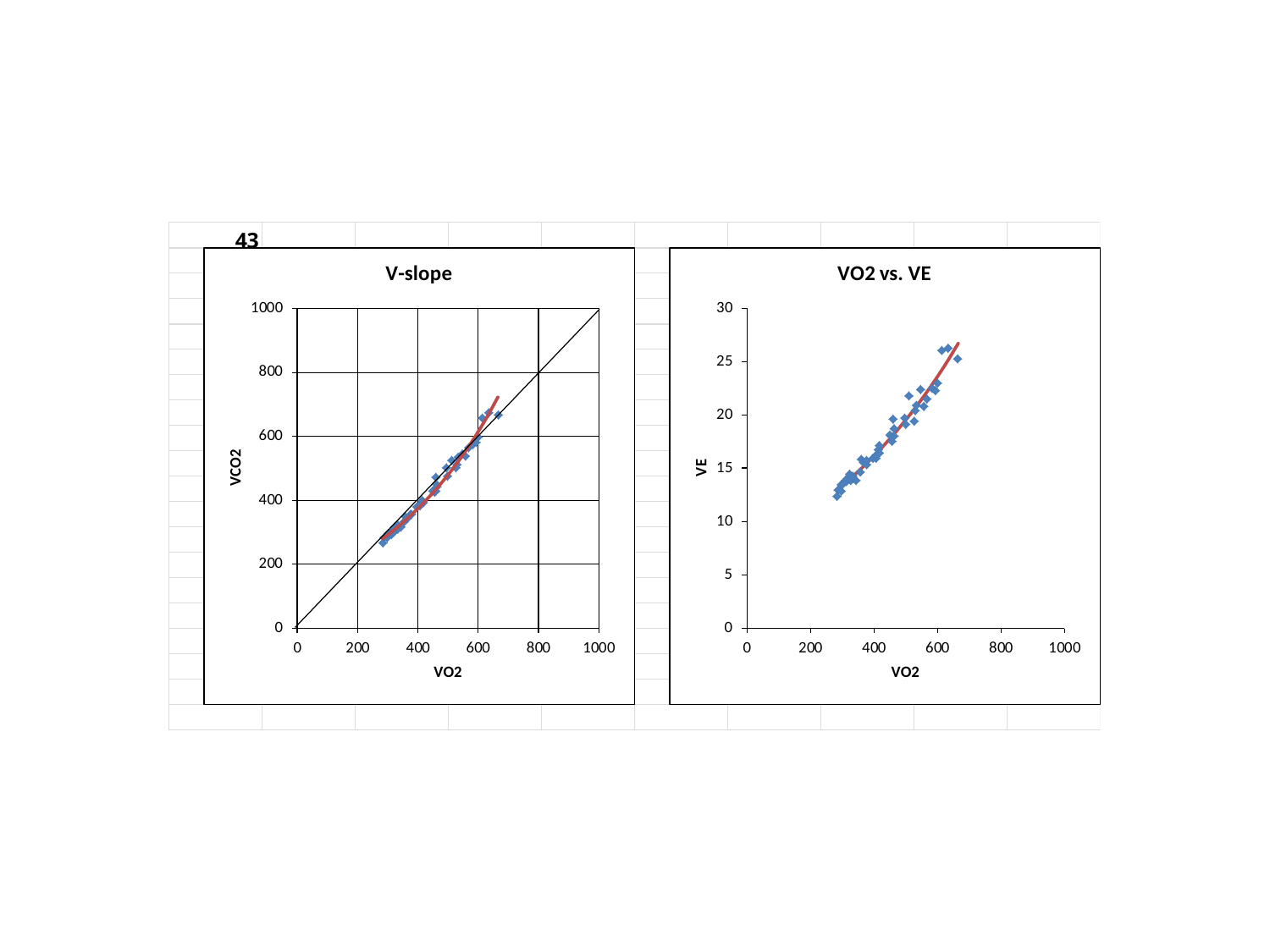

## Slide 45
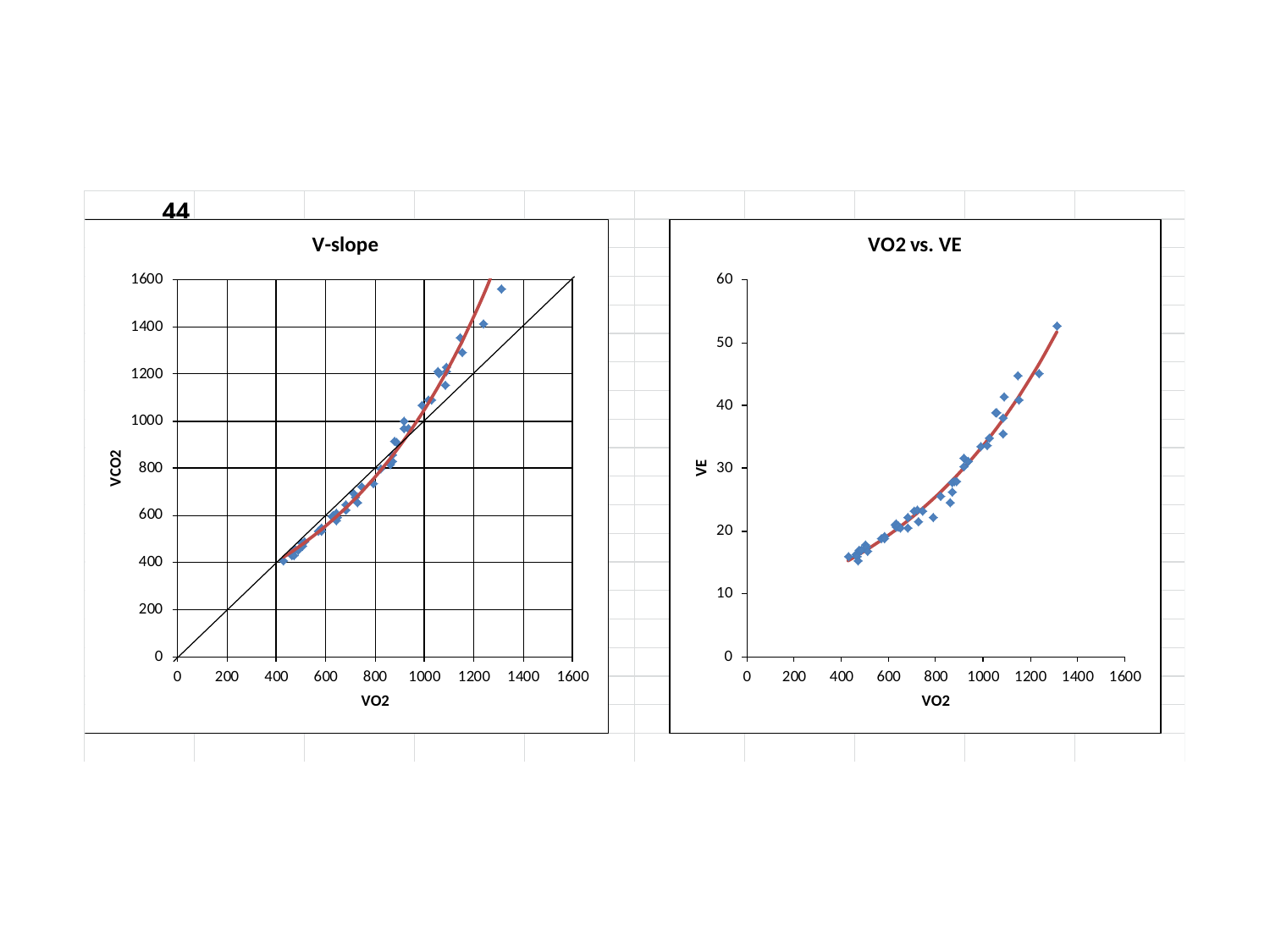

## Slide 46
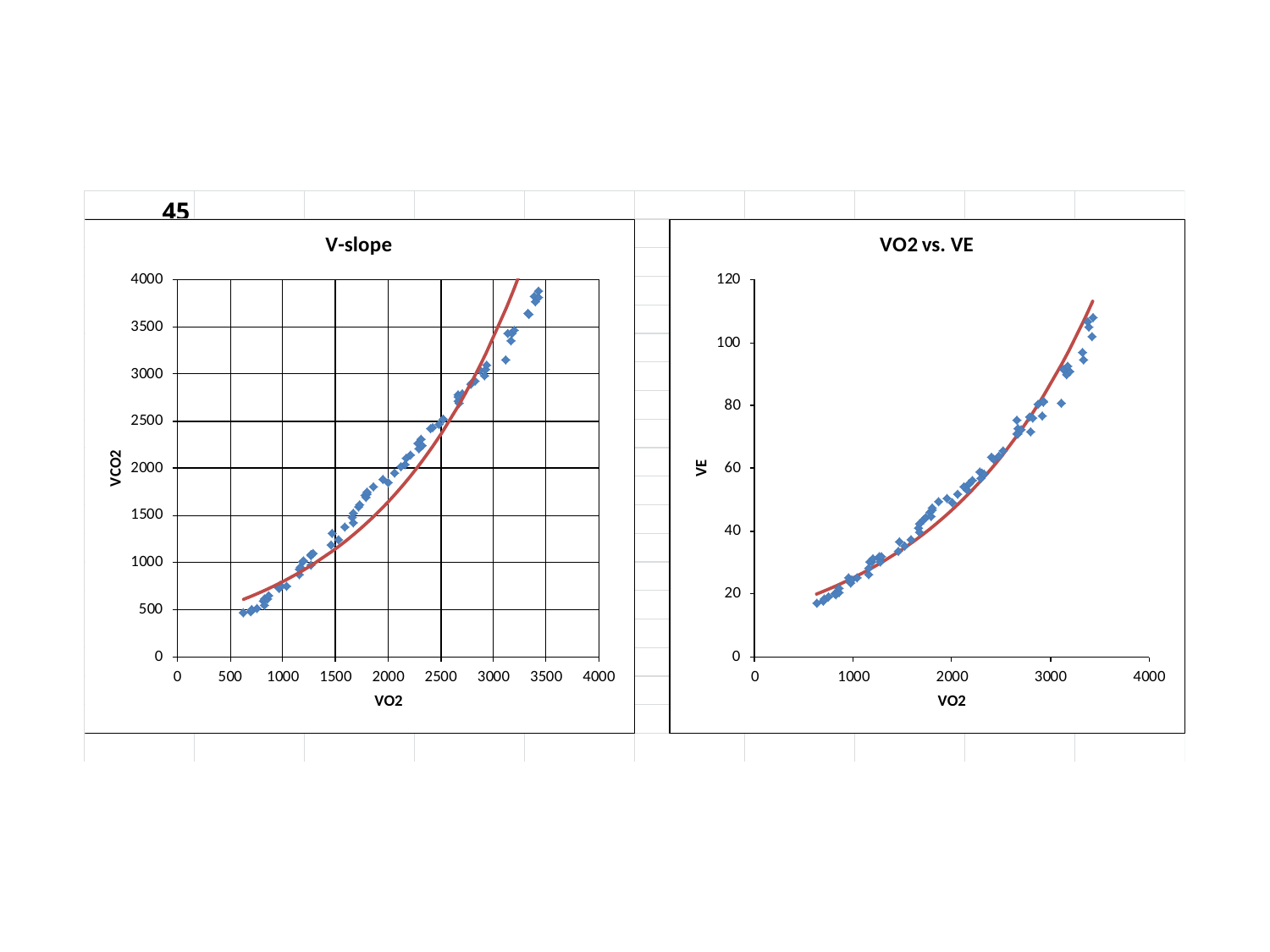

## Slide 47
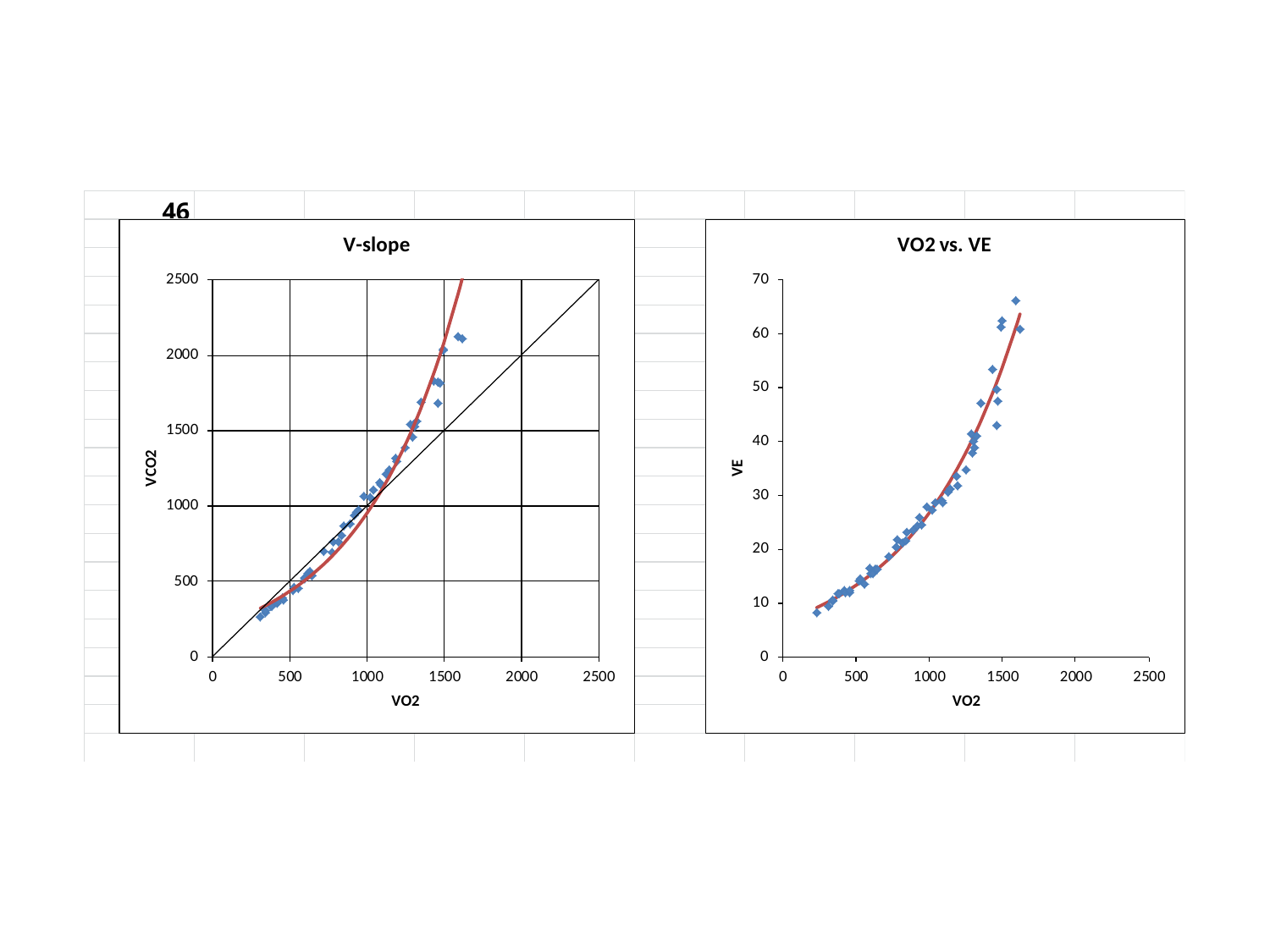

## Slide 48
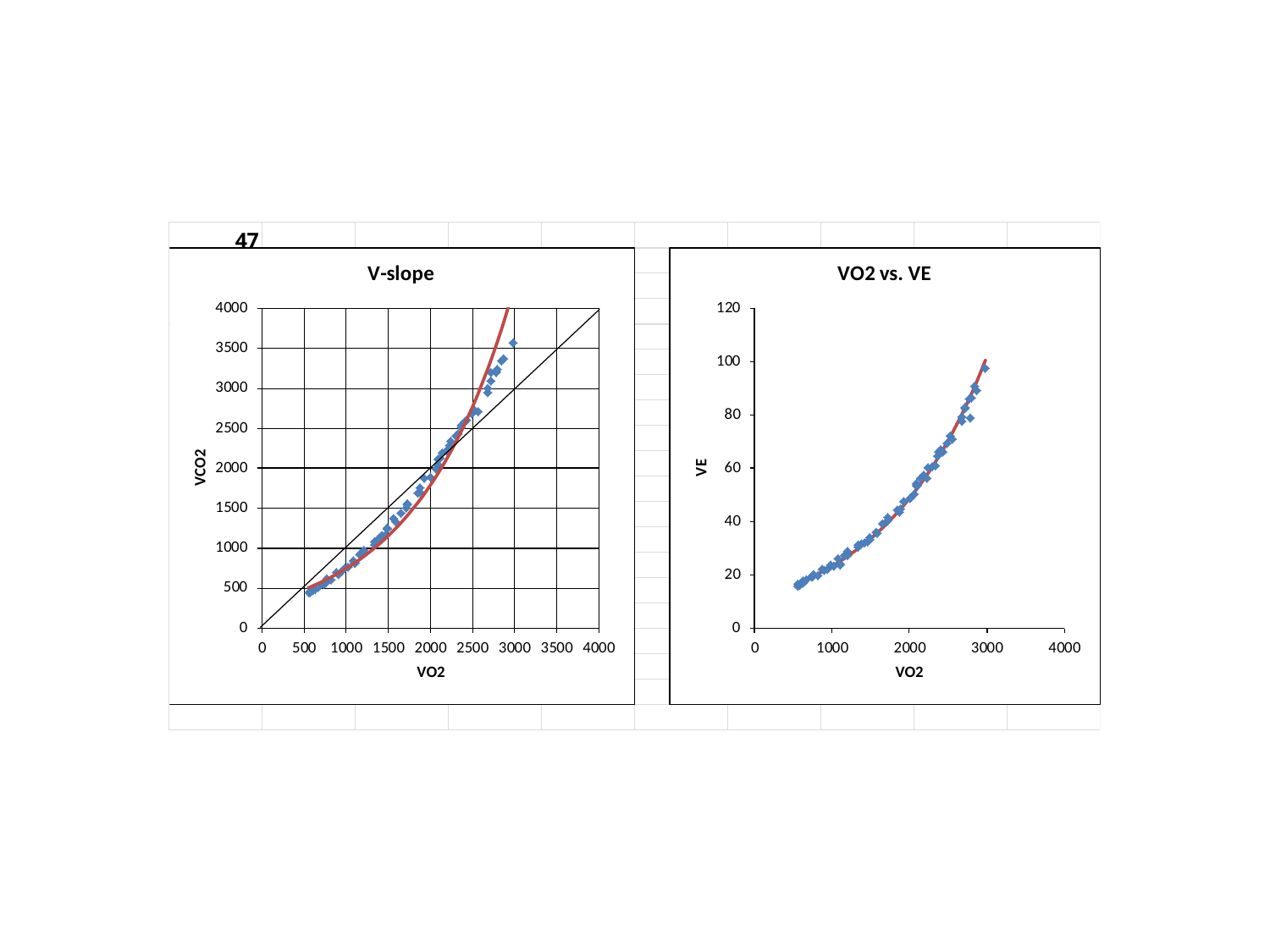

## Slide 49
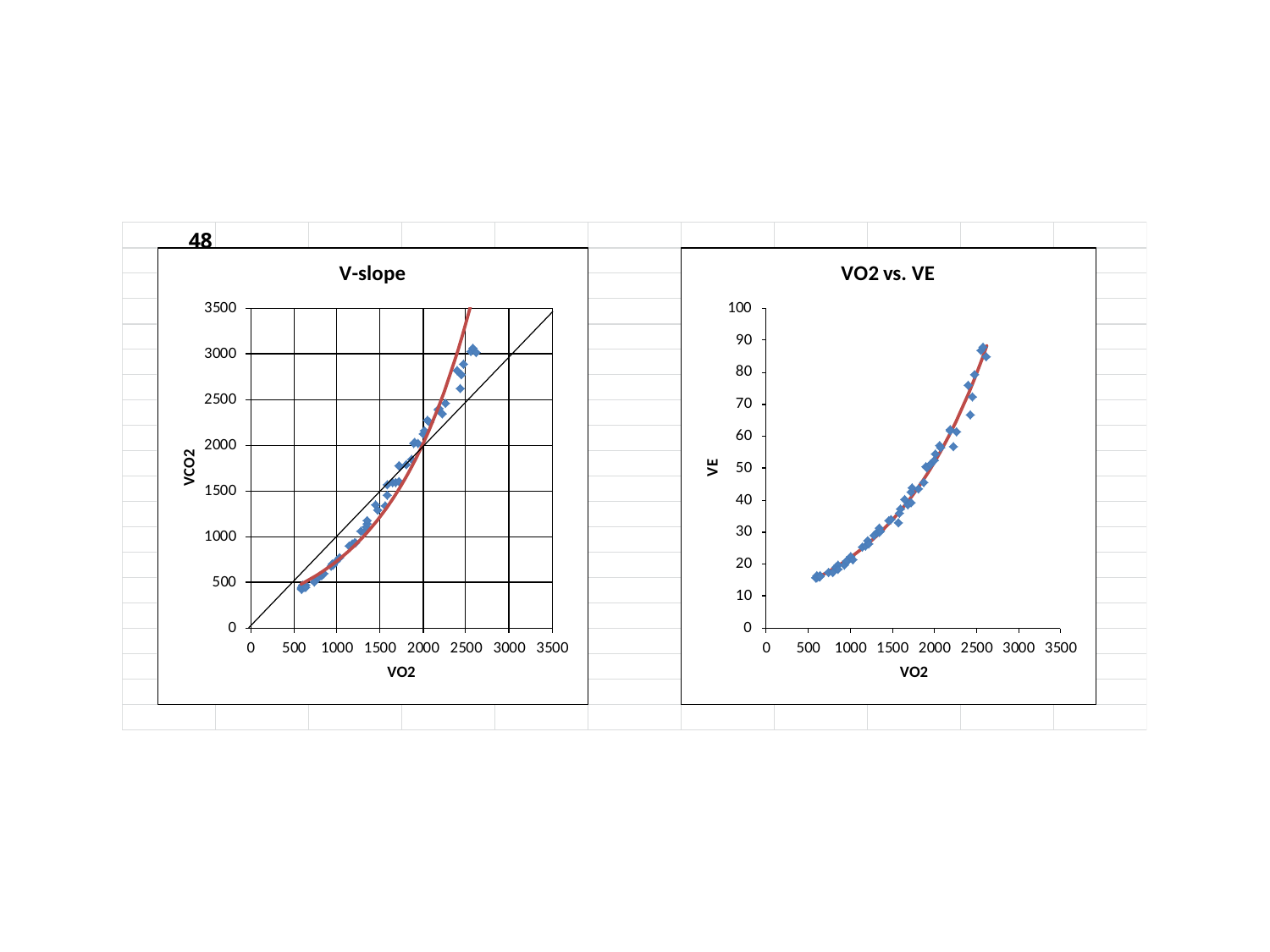

## Slide 50
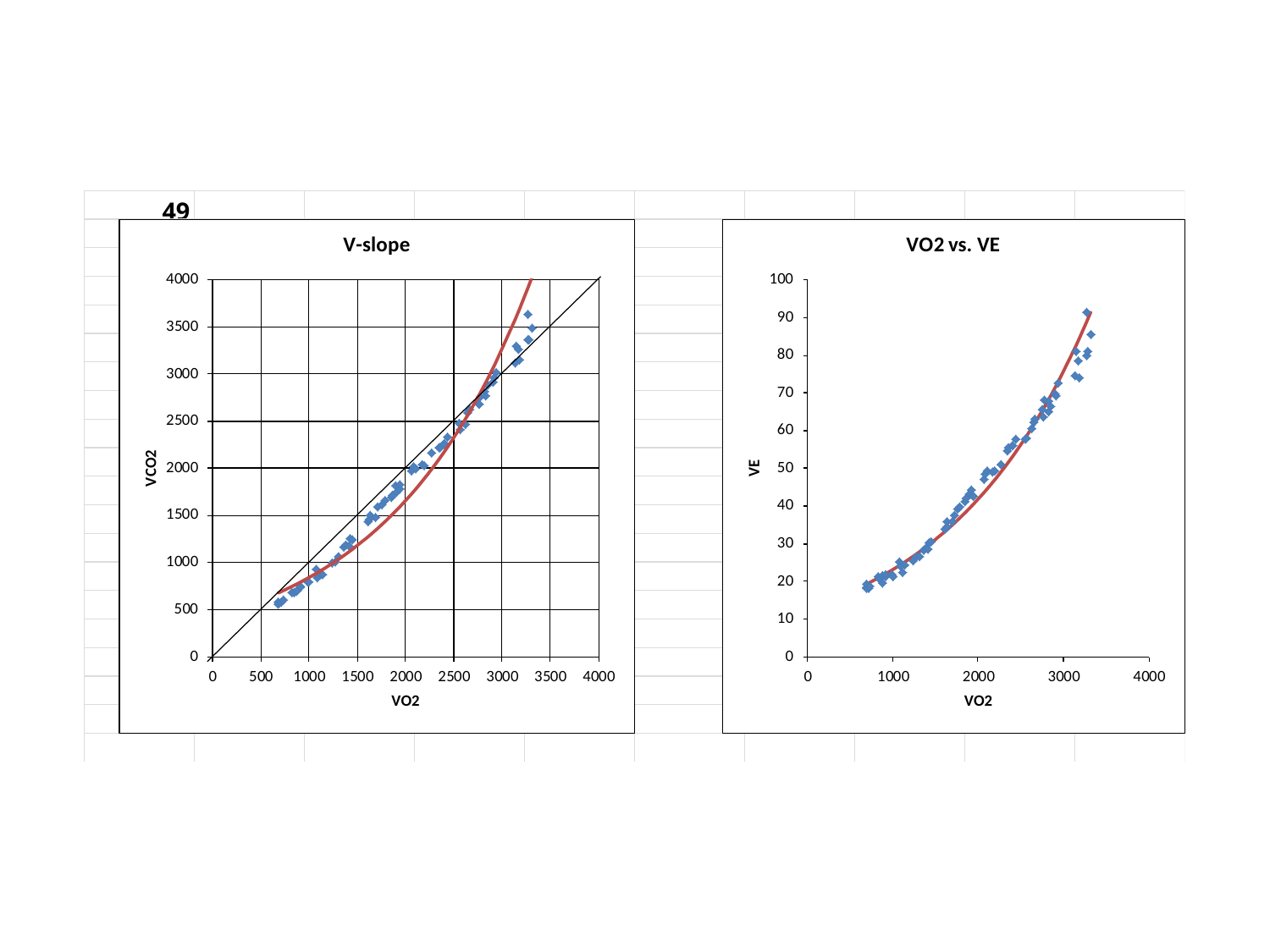

## Slide 51
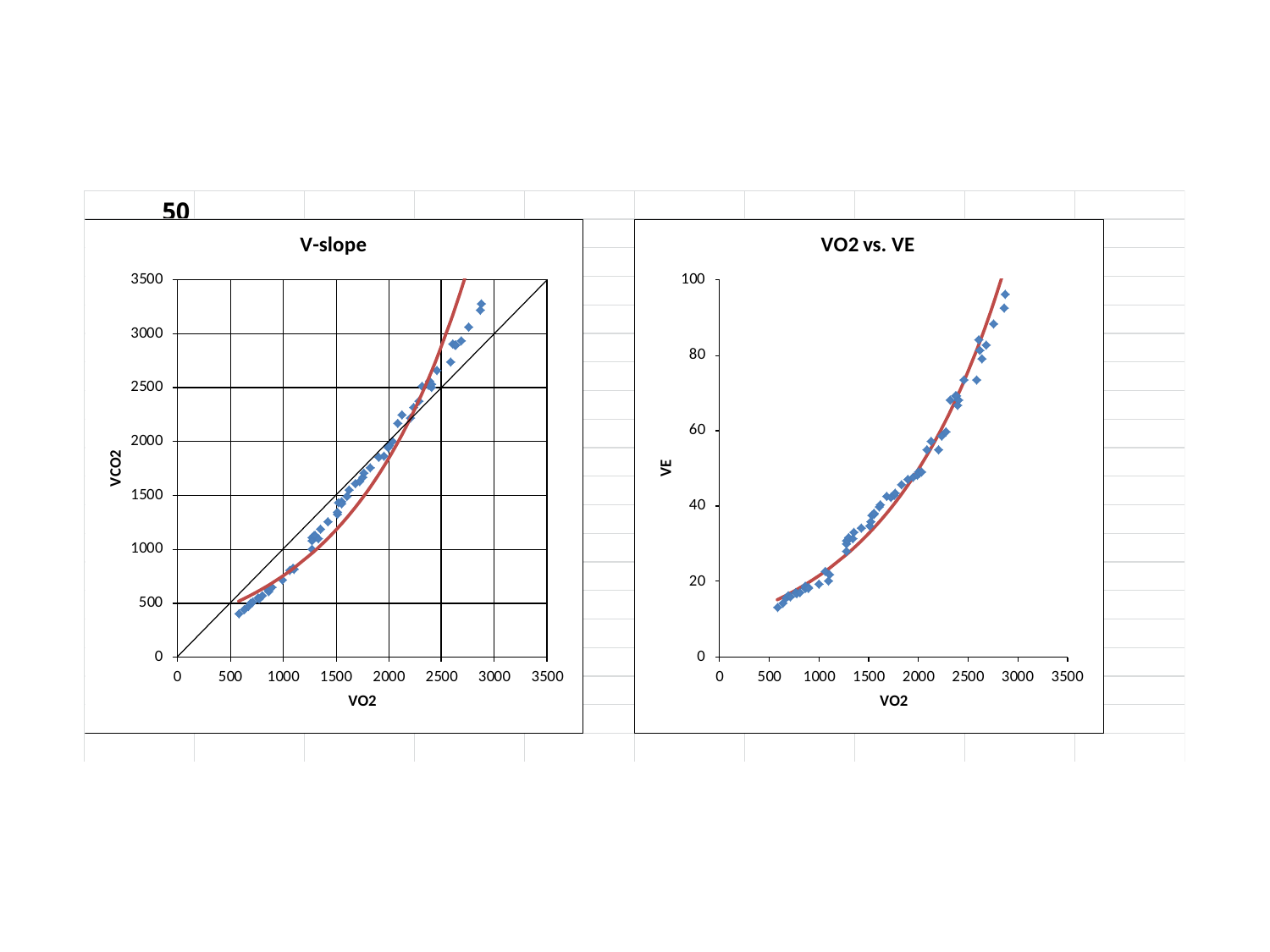

## Slide 52
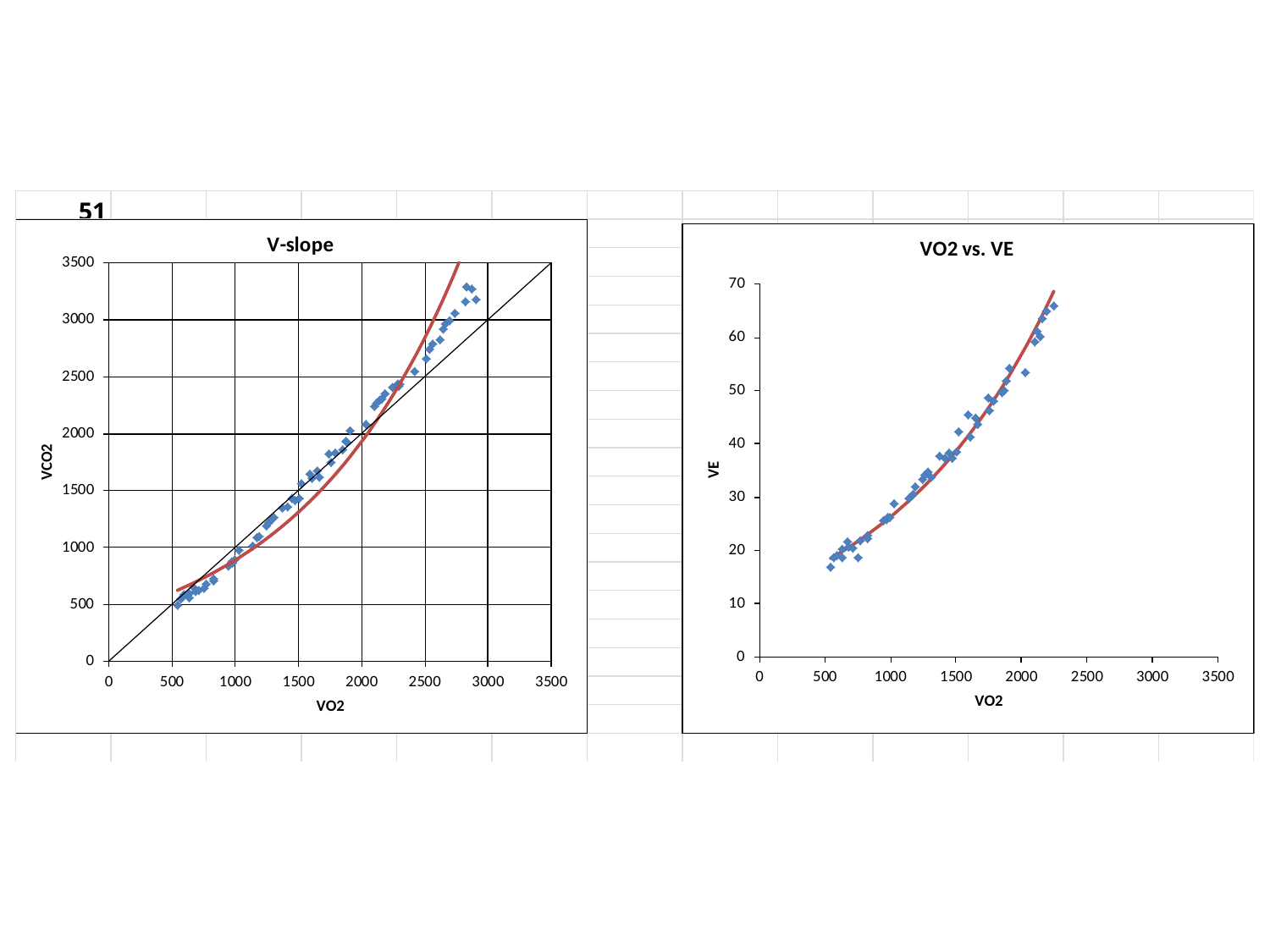

## Slide 53
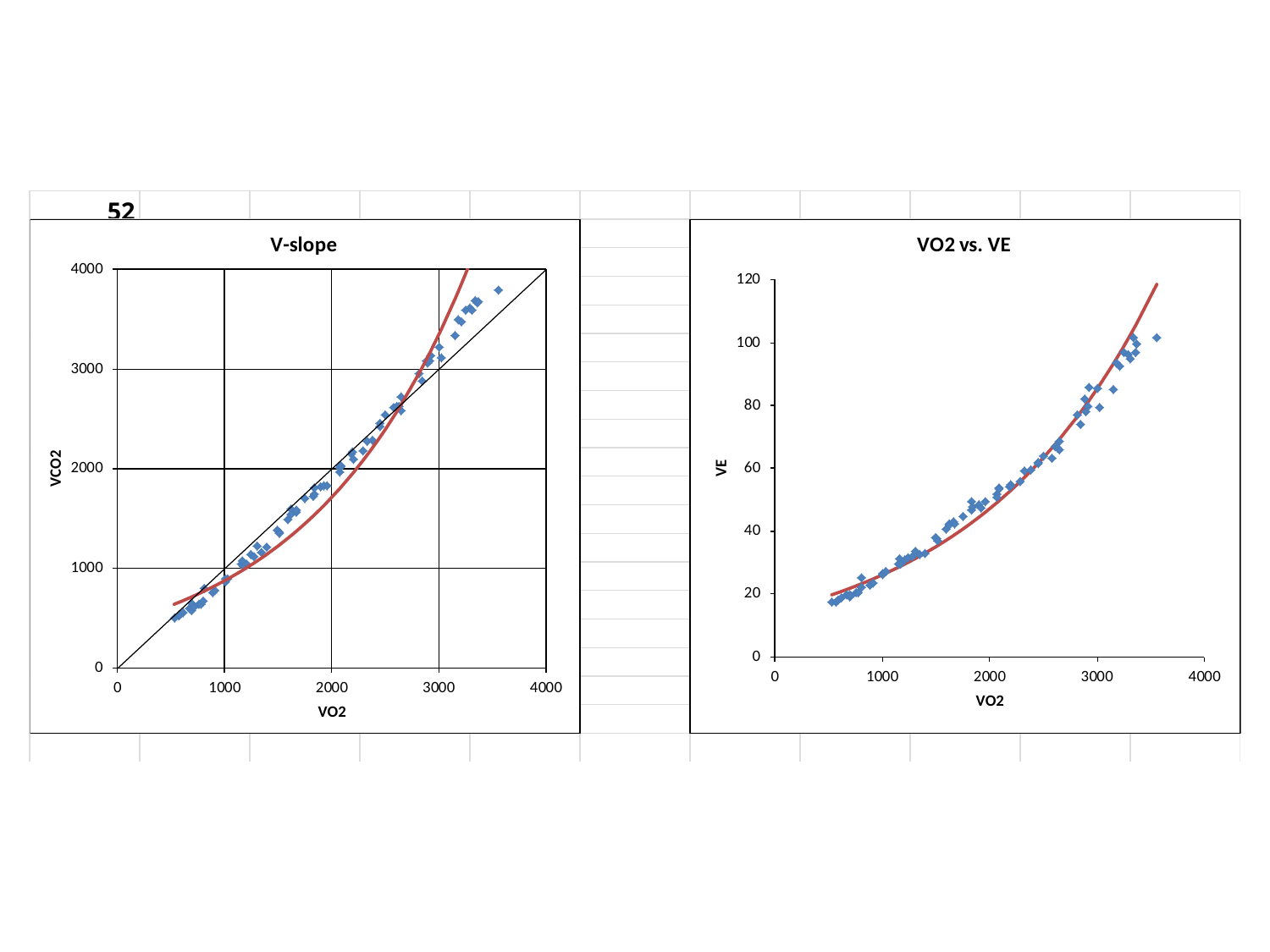

## Slide 54
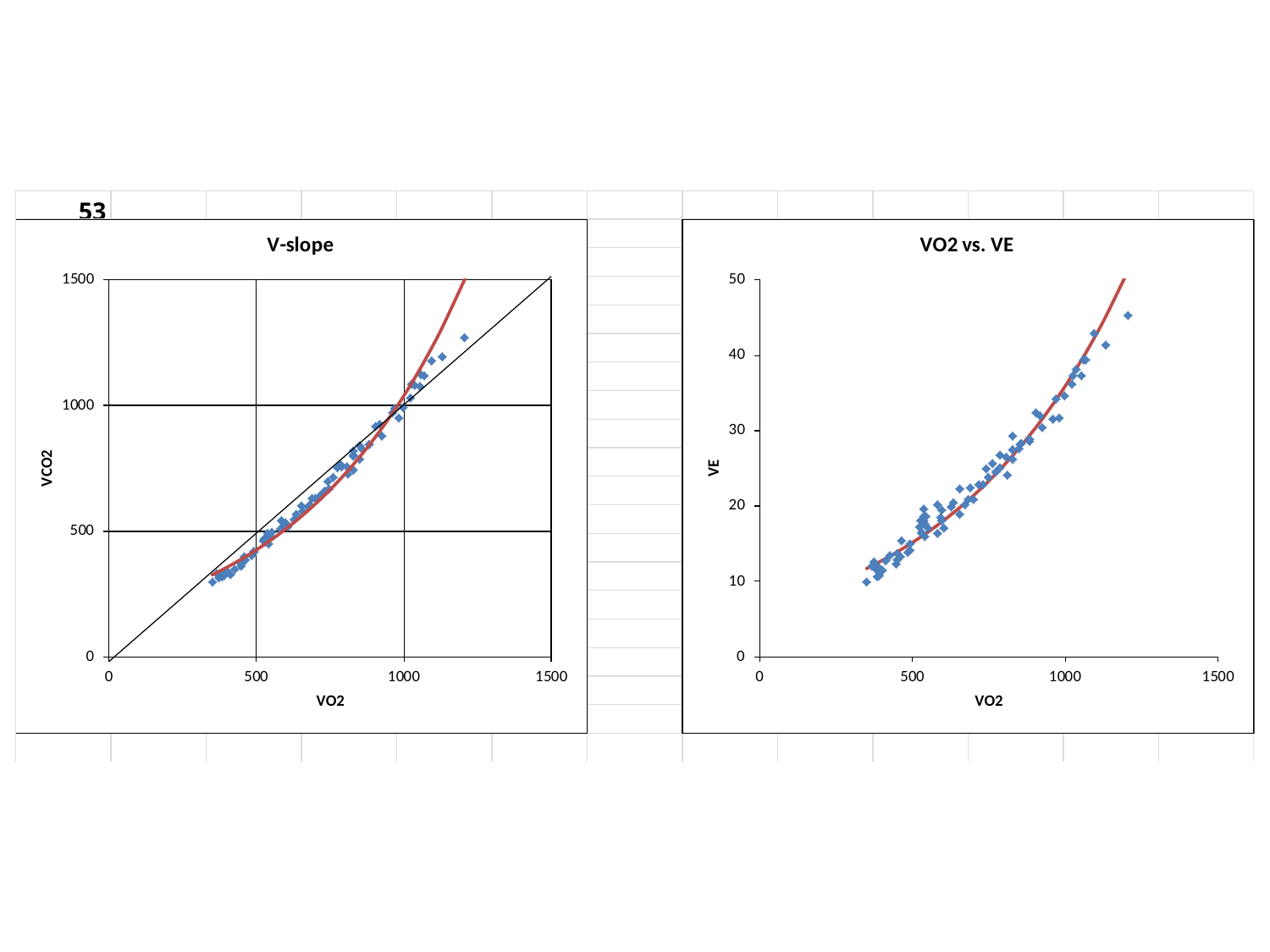

## Slide 55
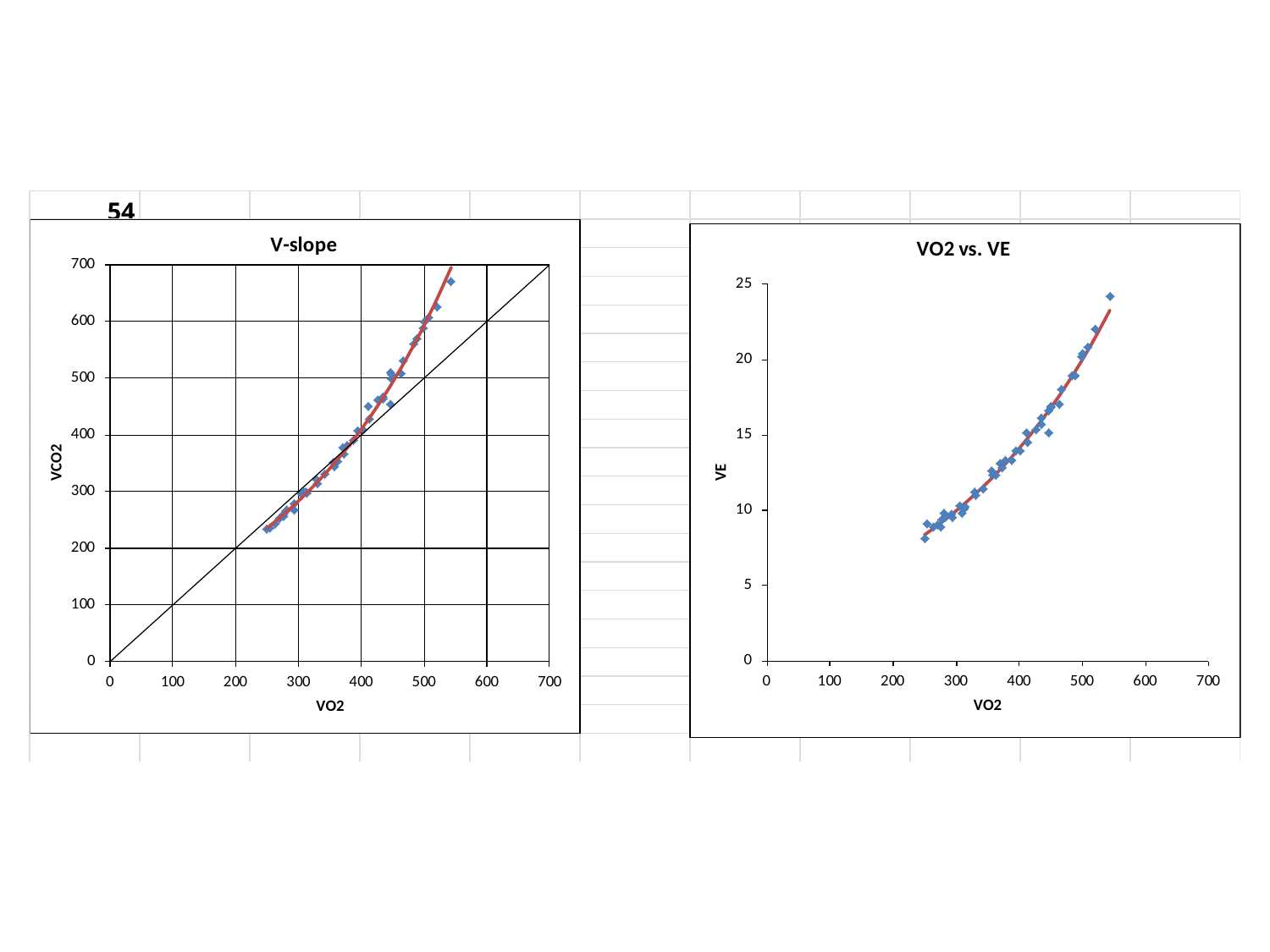

## Slide 56
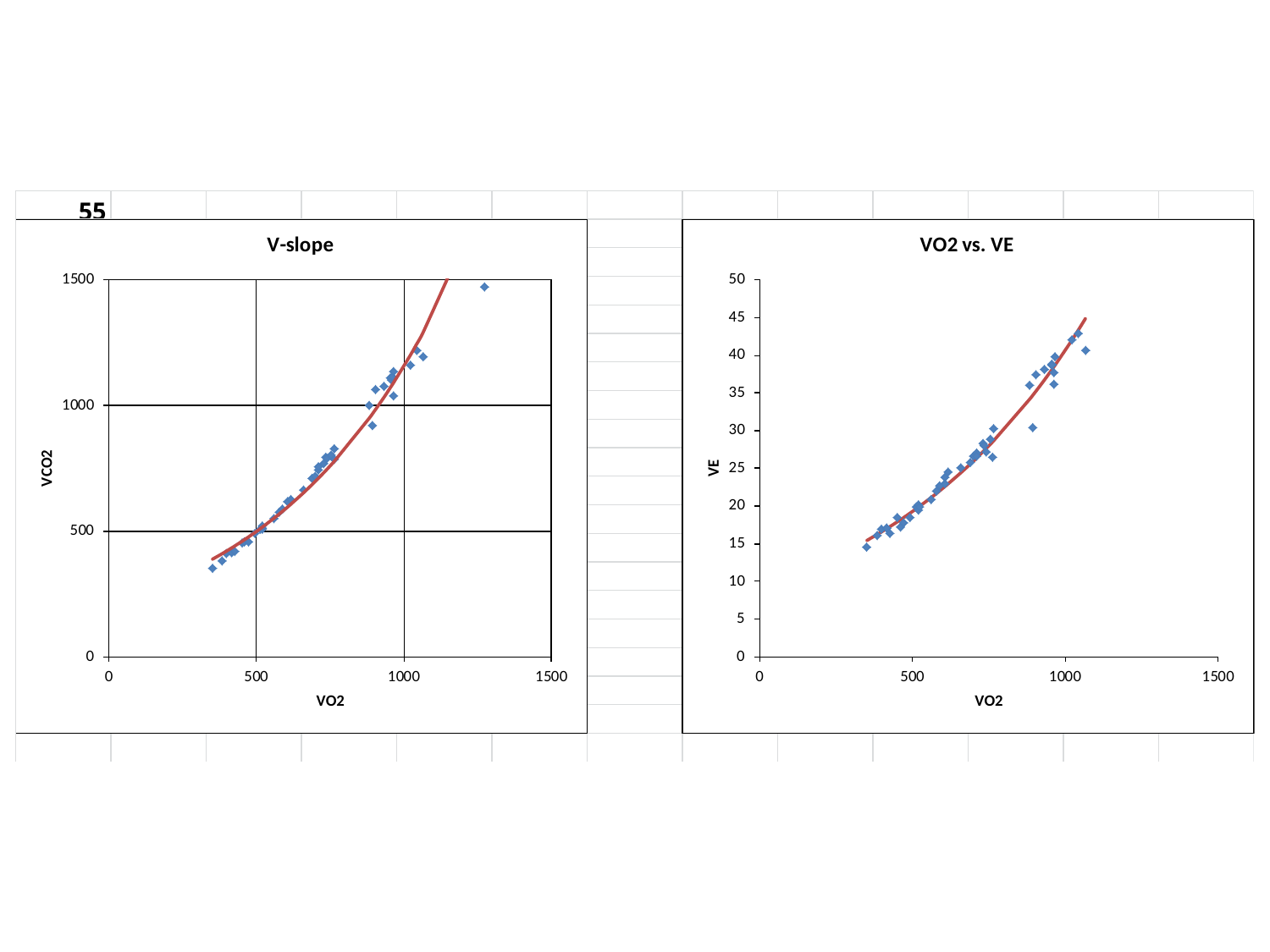

## Slide 57
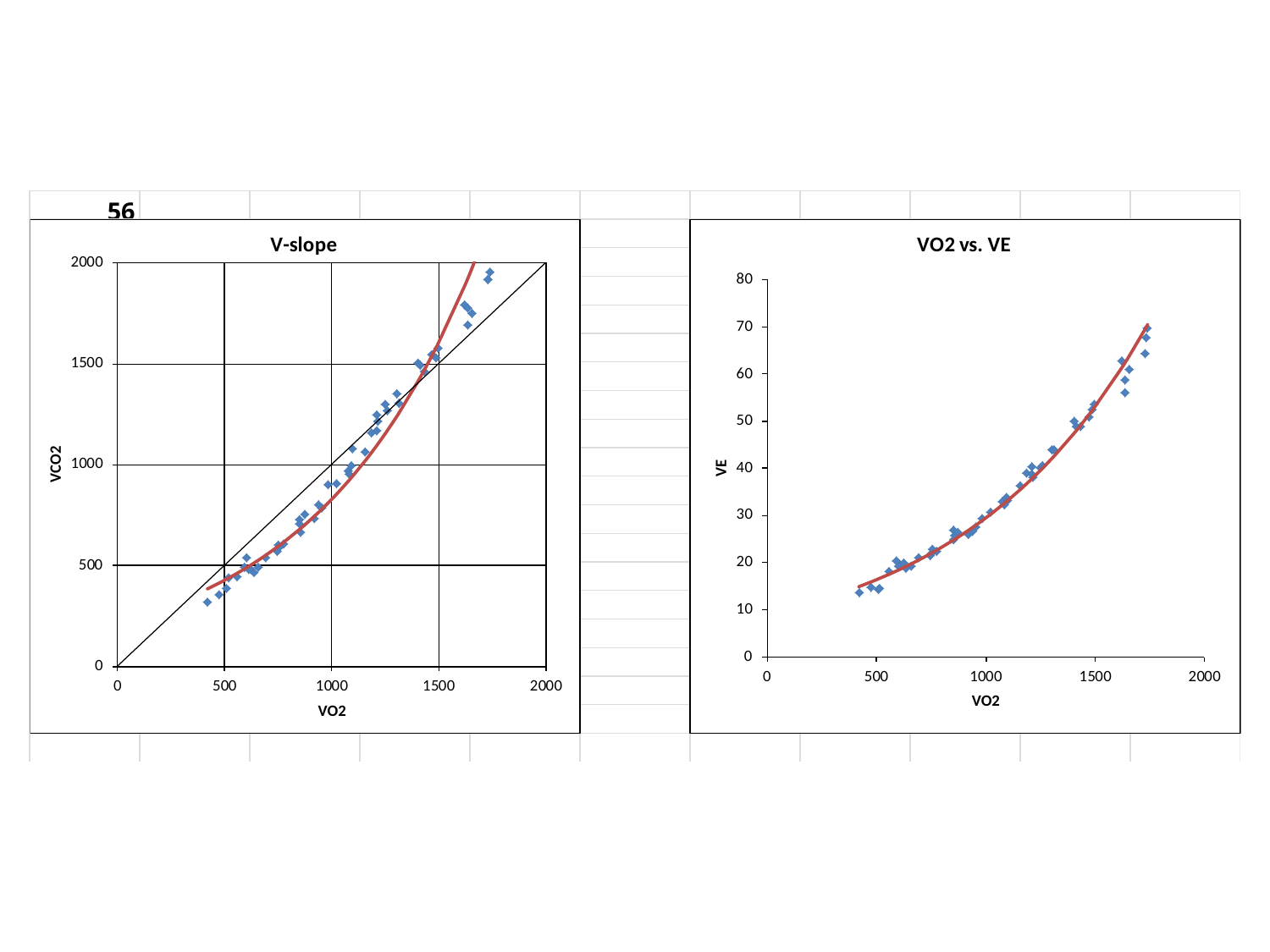

## Slide 58
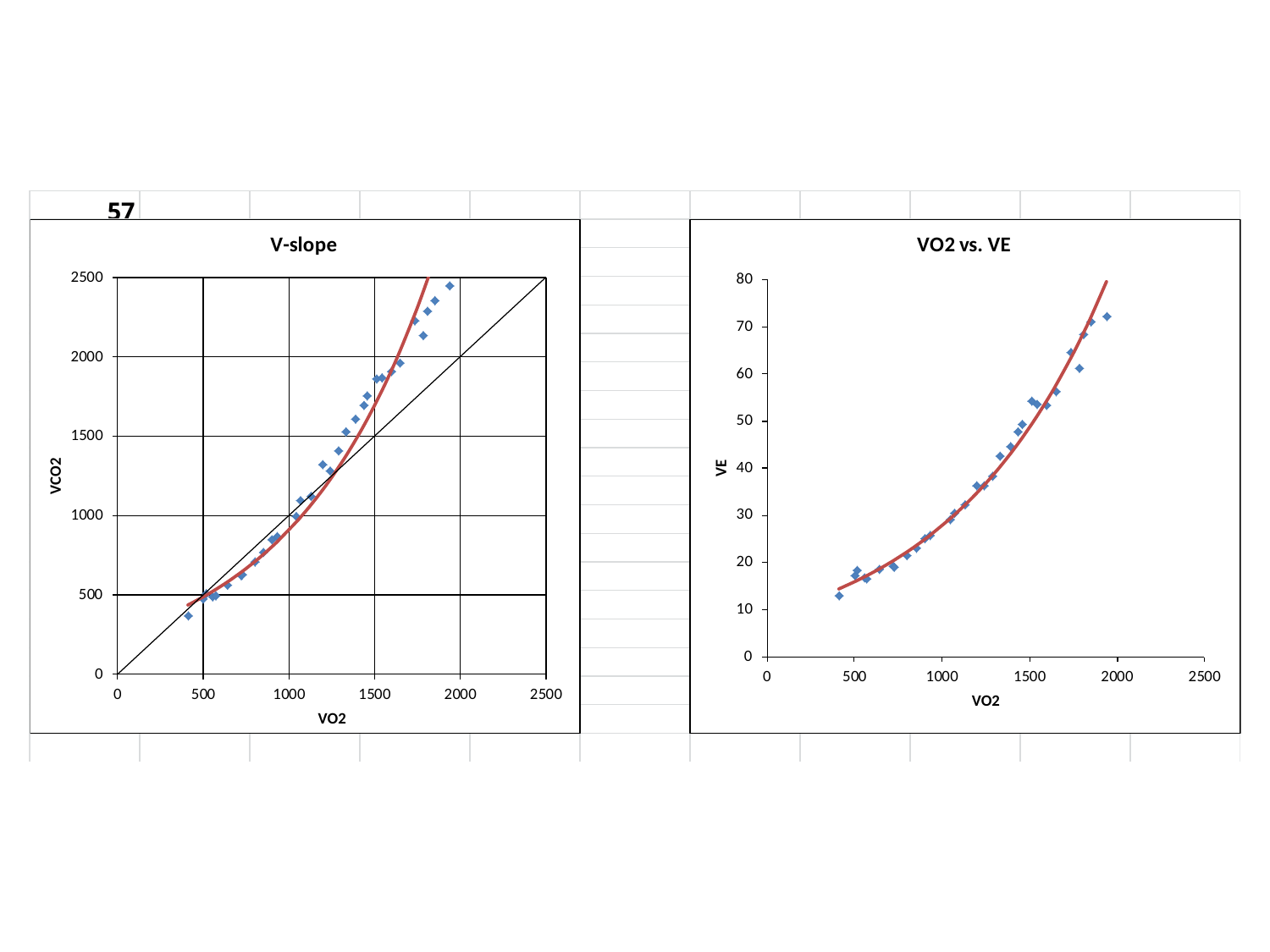

## Slide 59
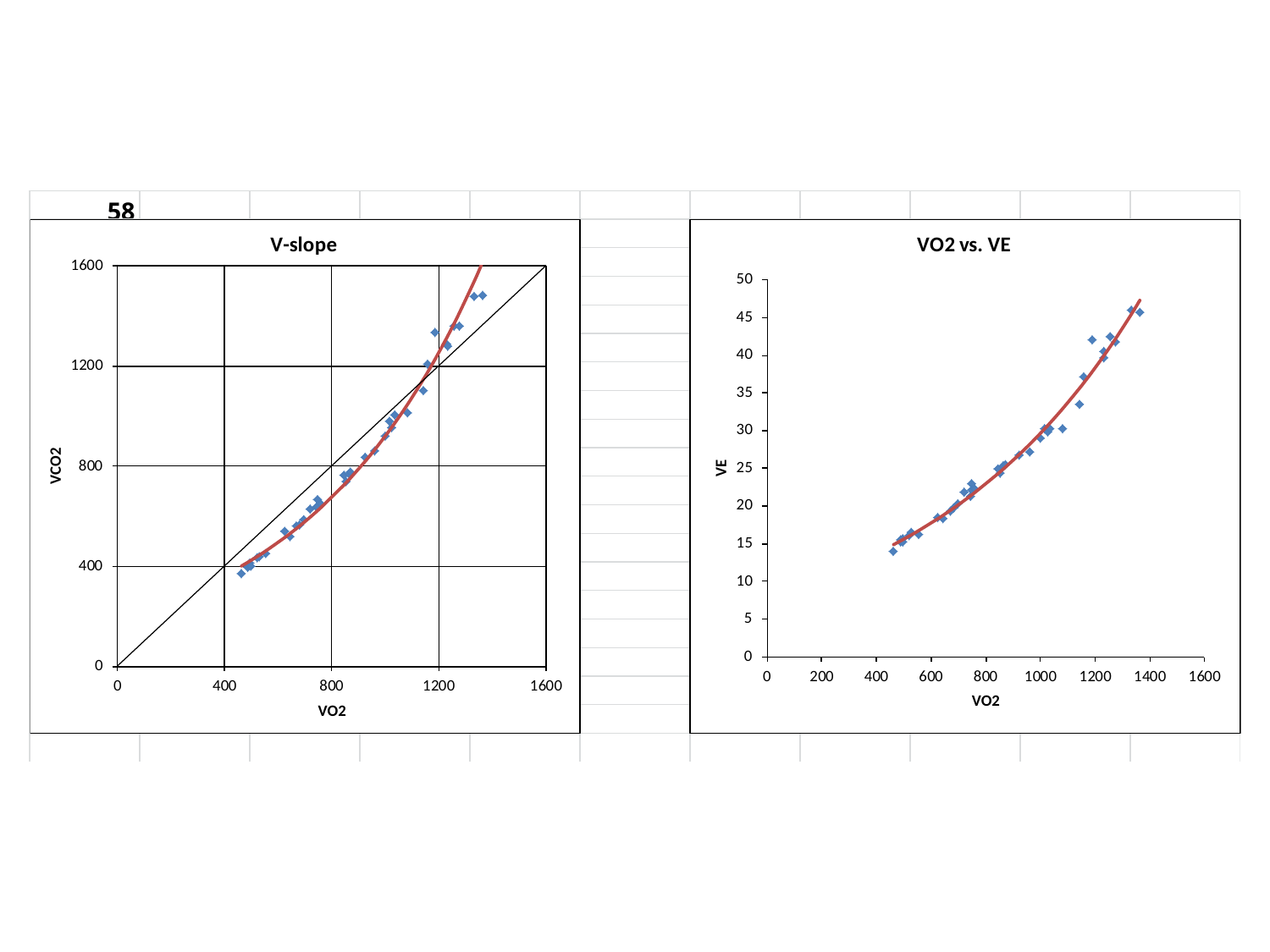

## Slide 60
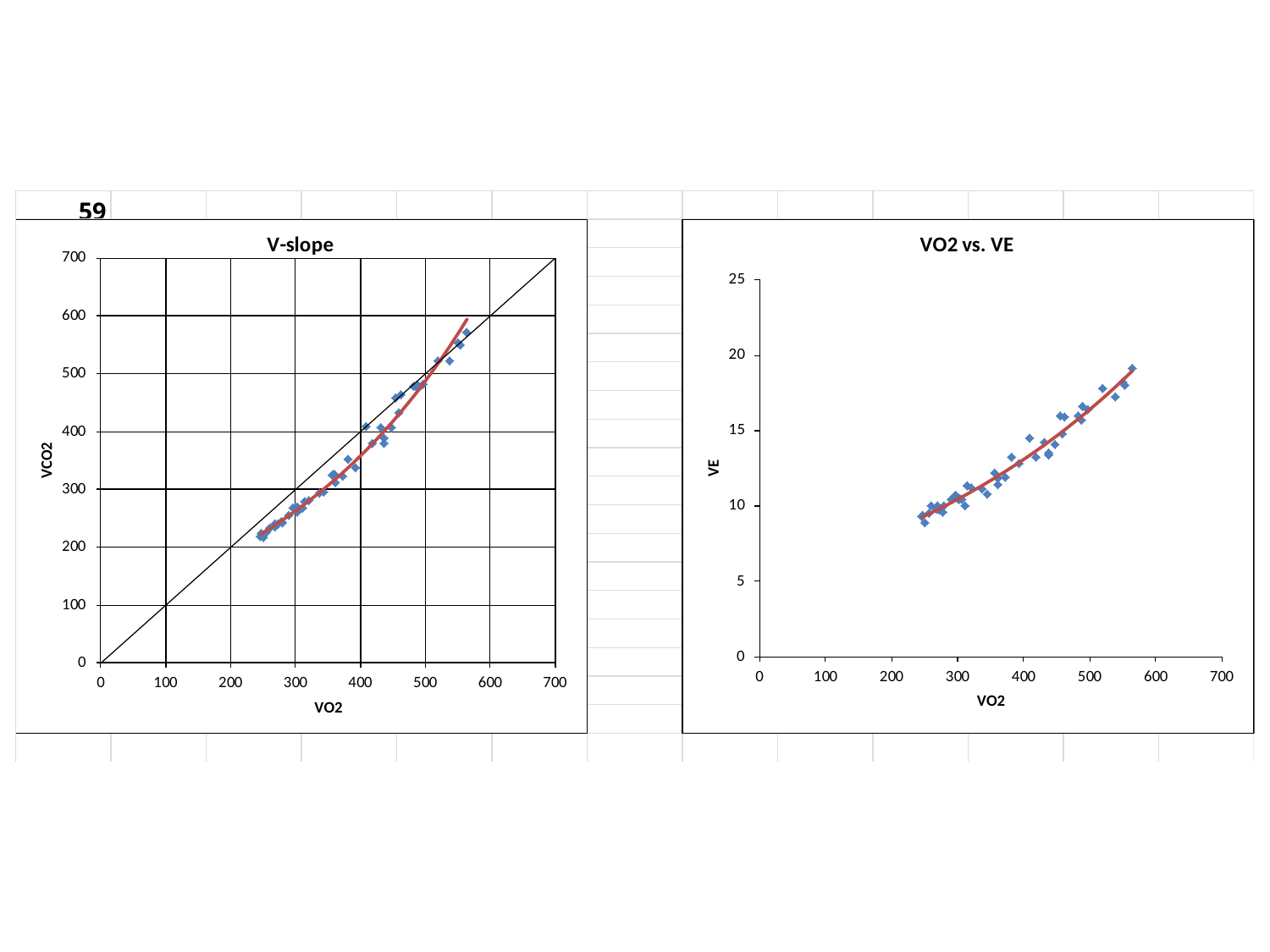

## Slide 61
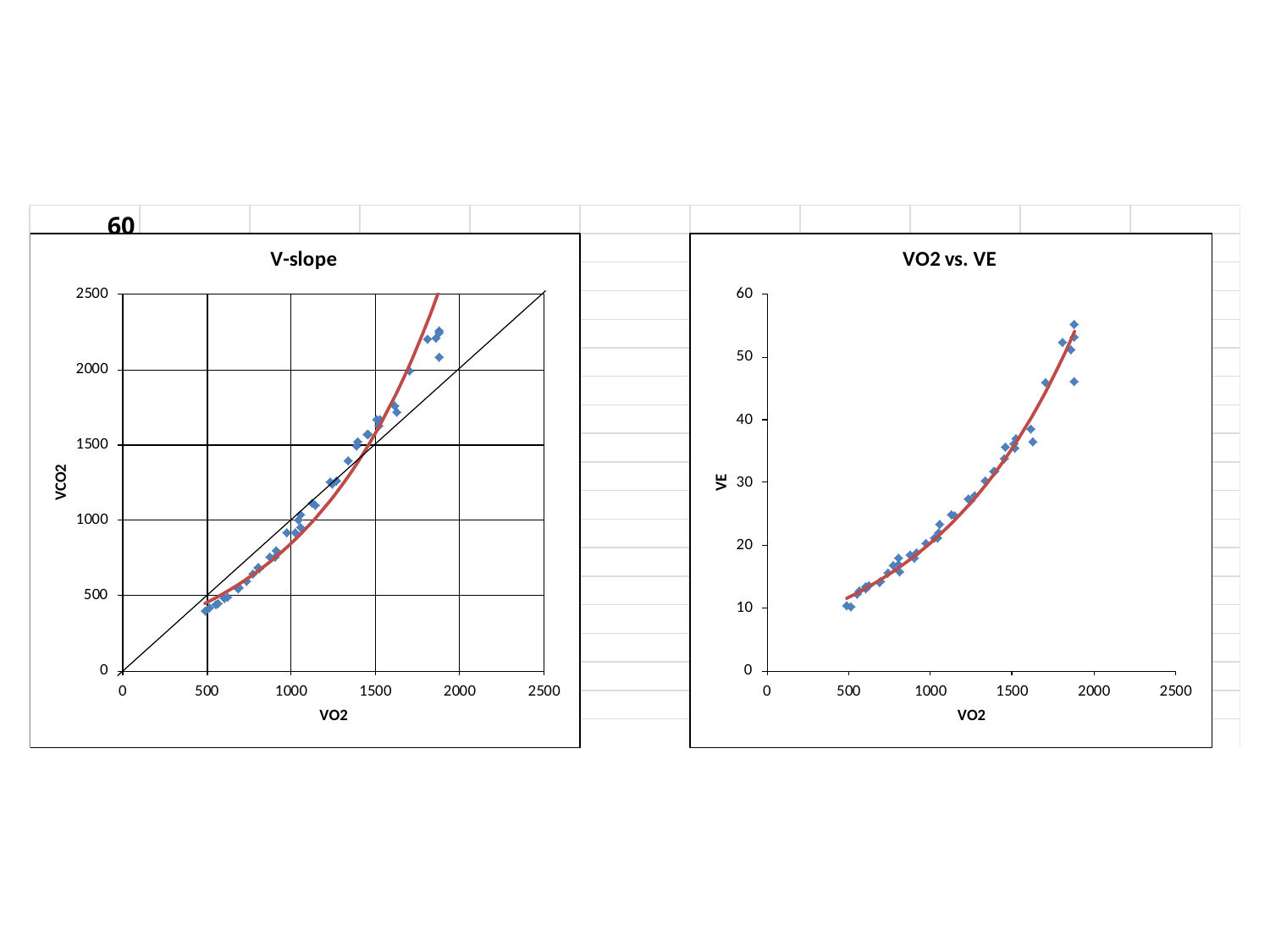

## Slide 62
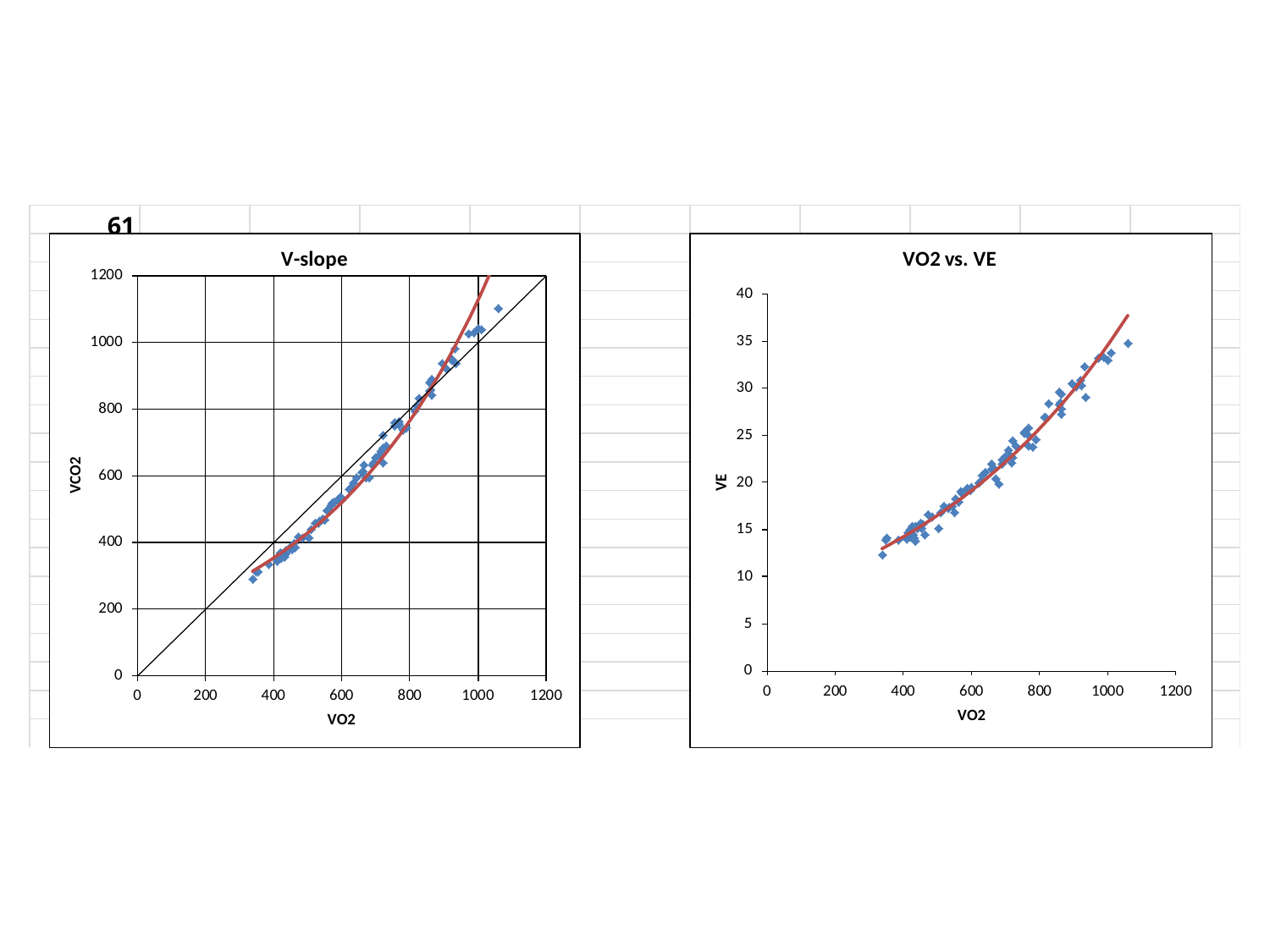

## Slide 63
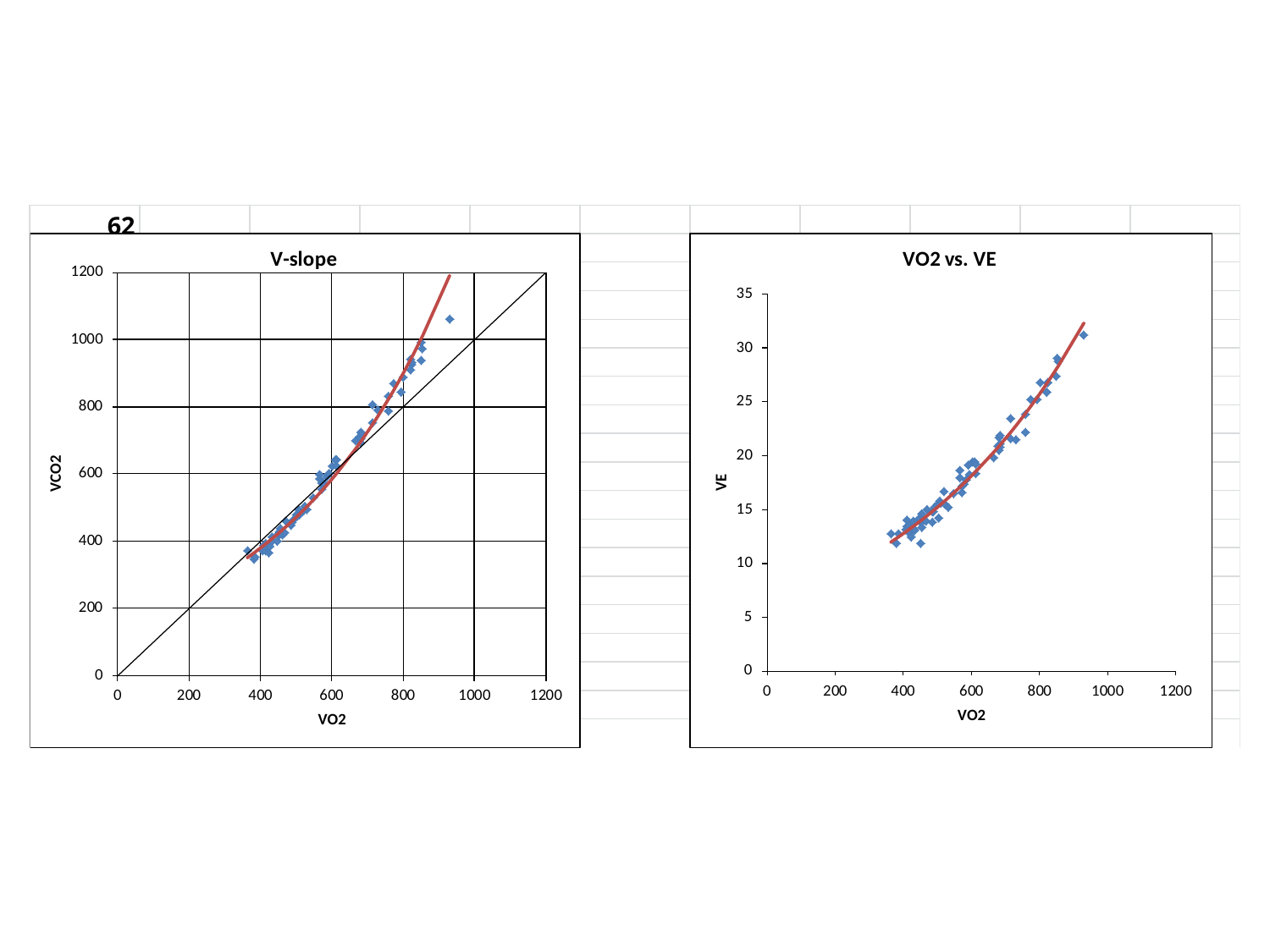

## Slide 64
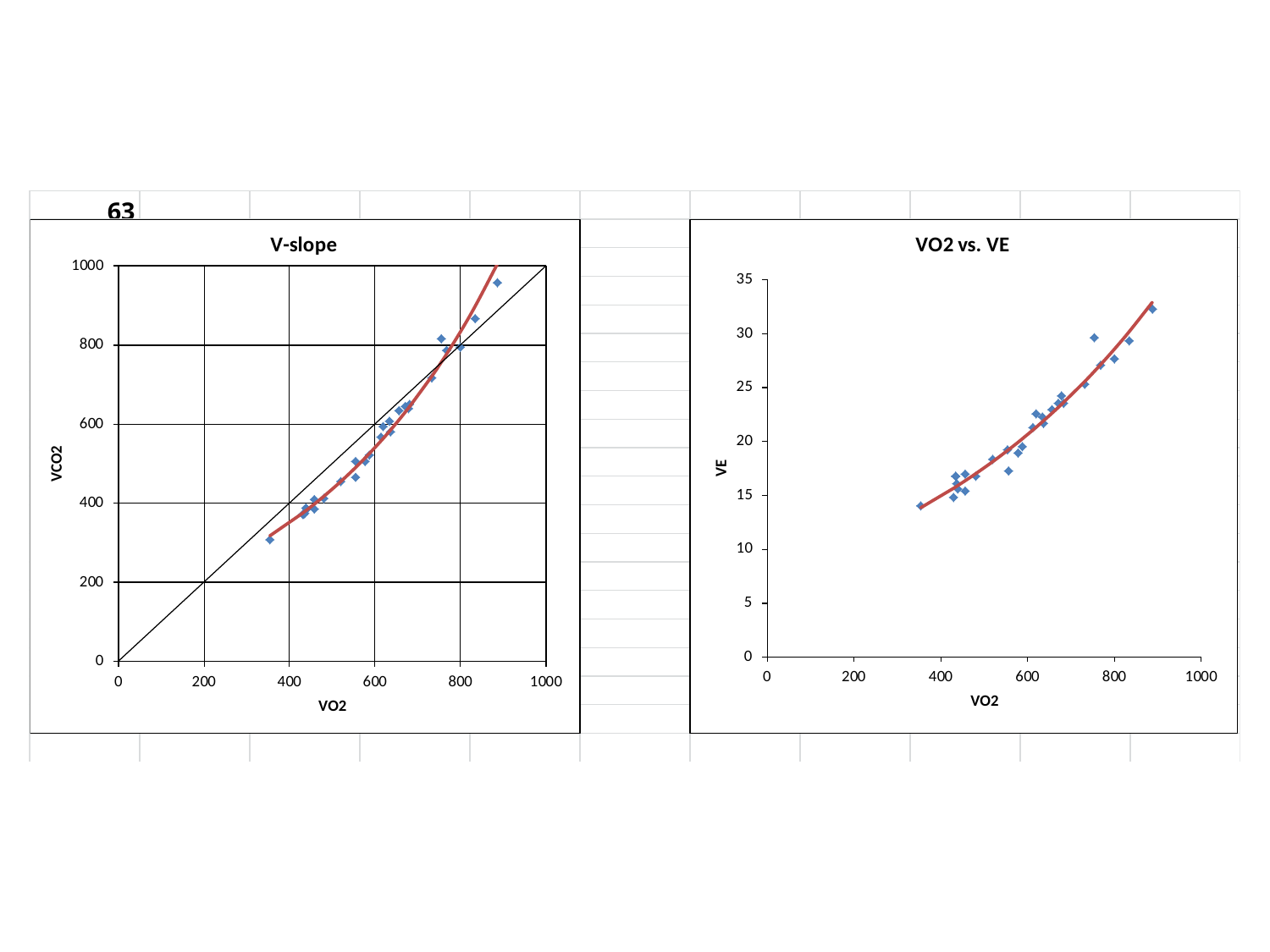

## Slide 65
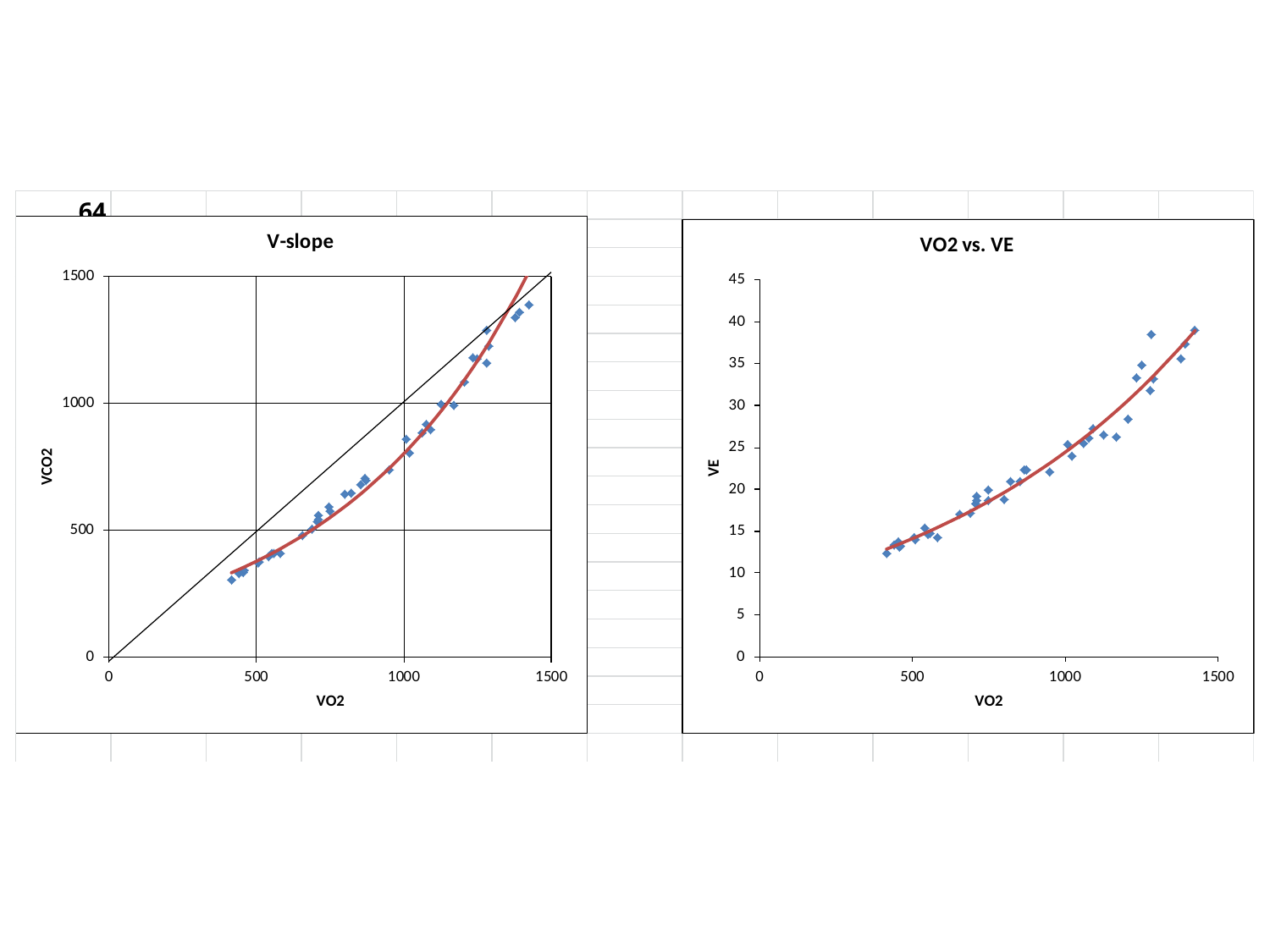

## Slide 66
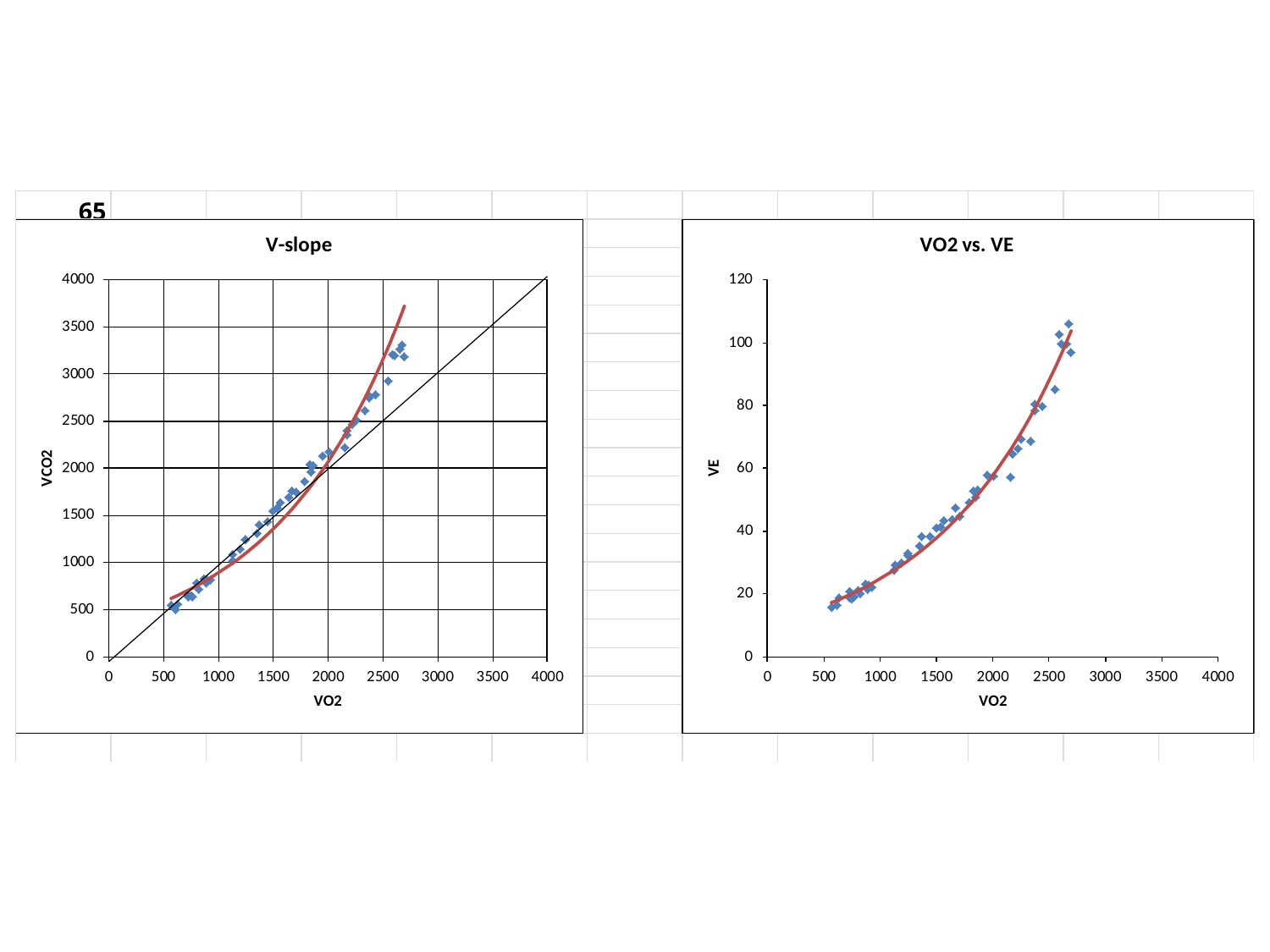

## Slide 67
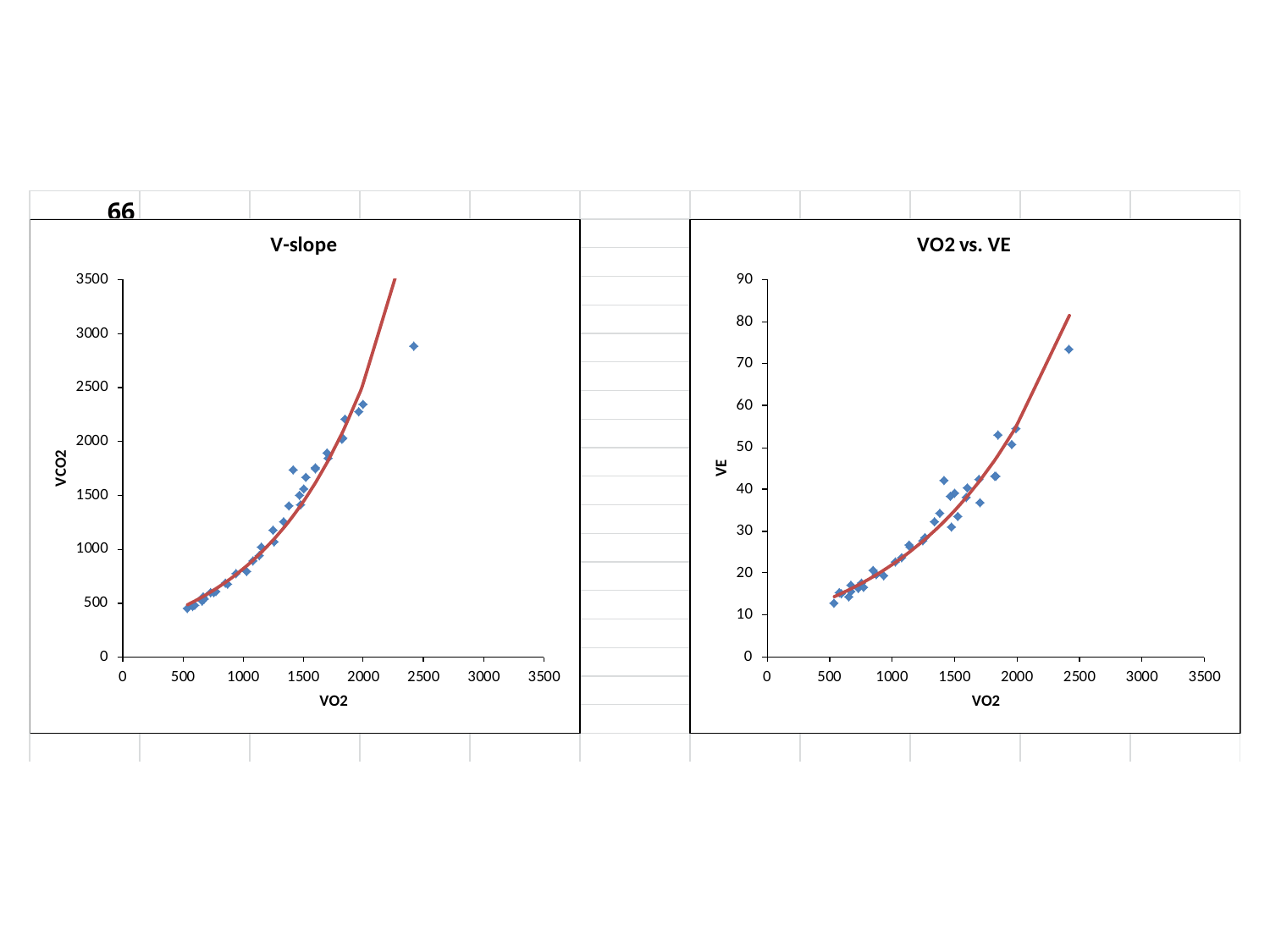

## Slide 68
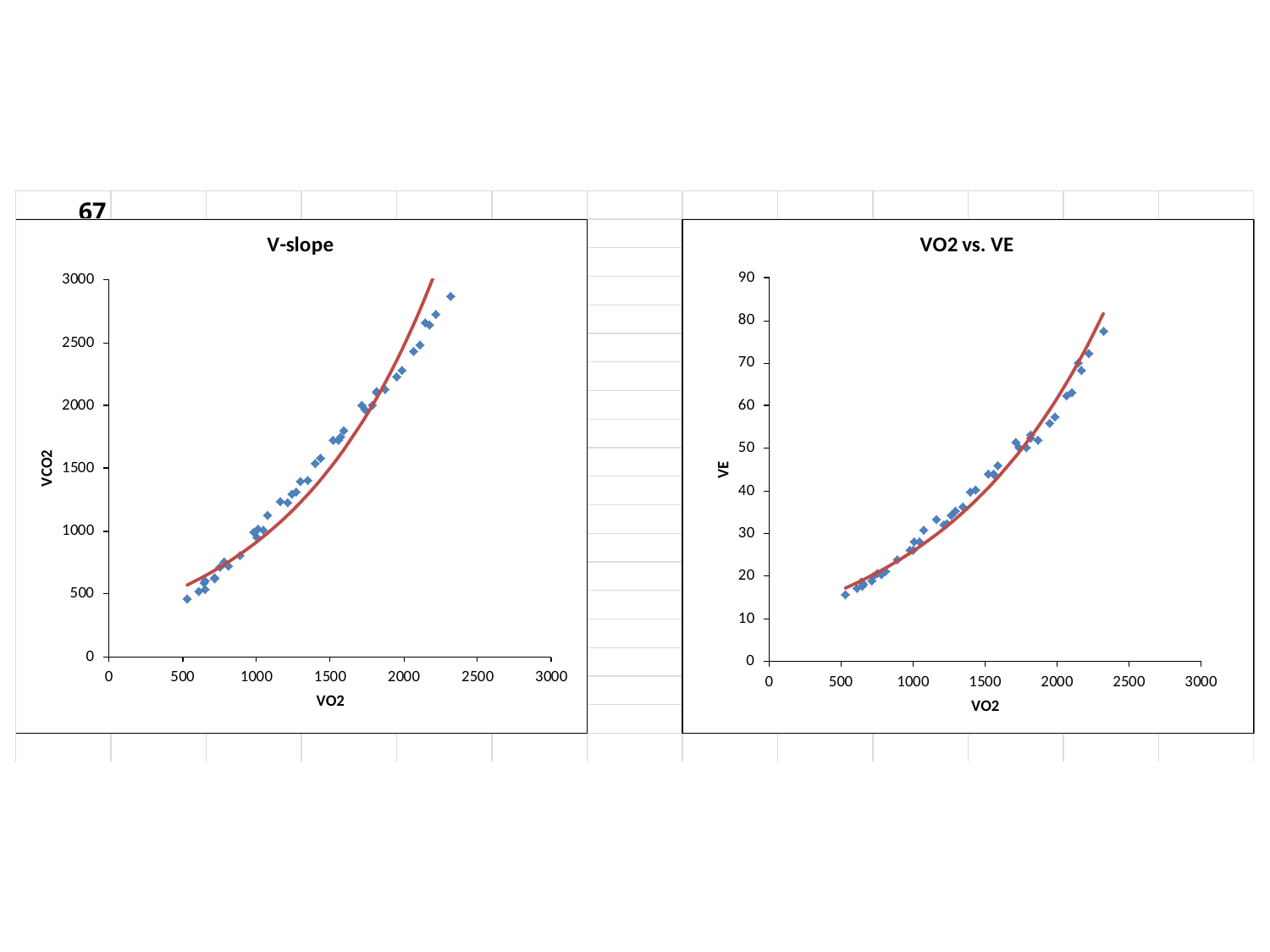

## Slide 69
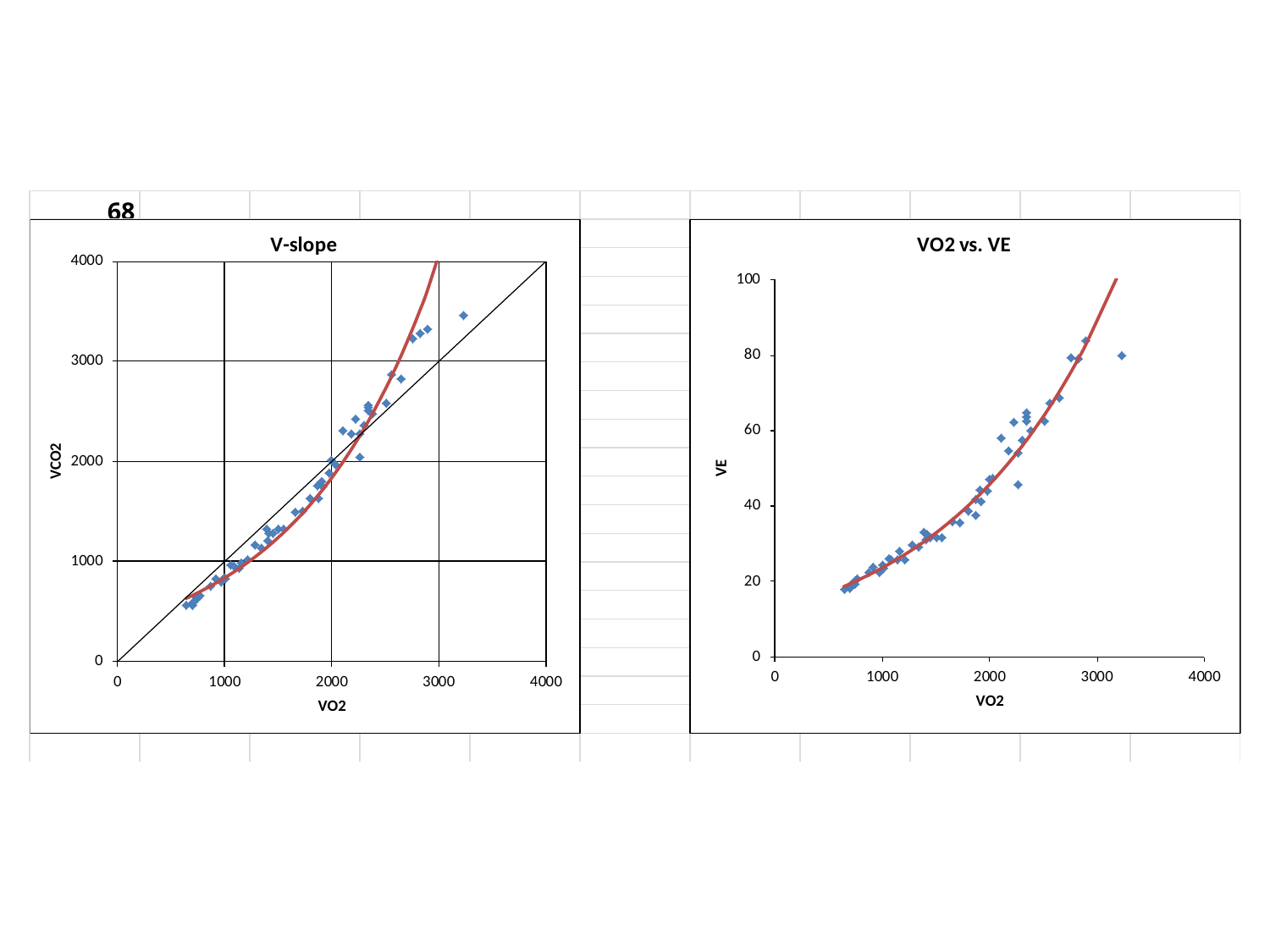

## Slide 70
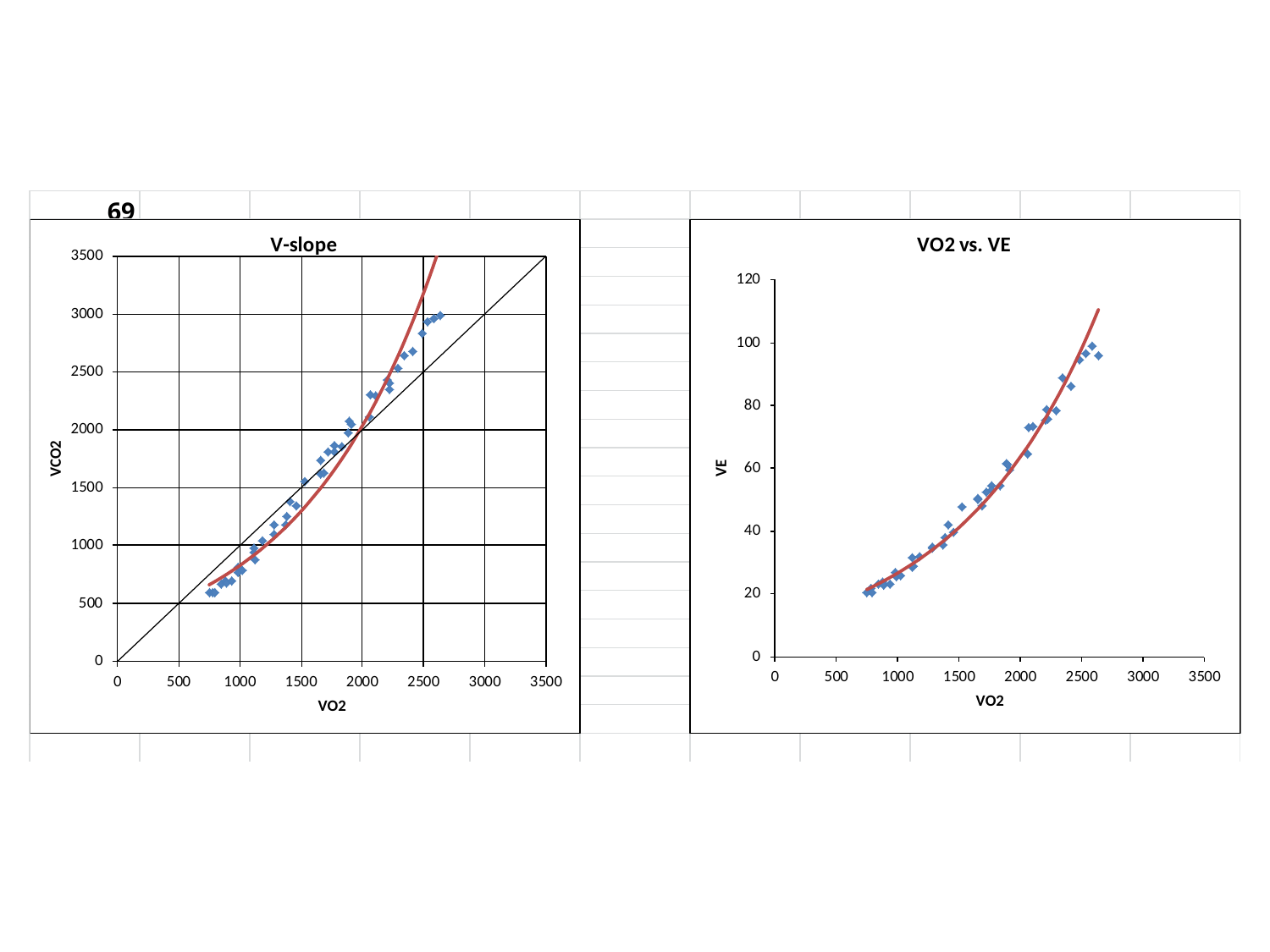

## Slide 71
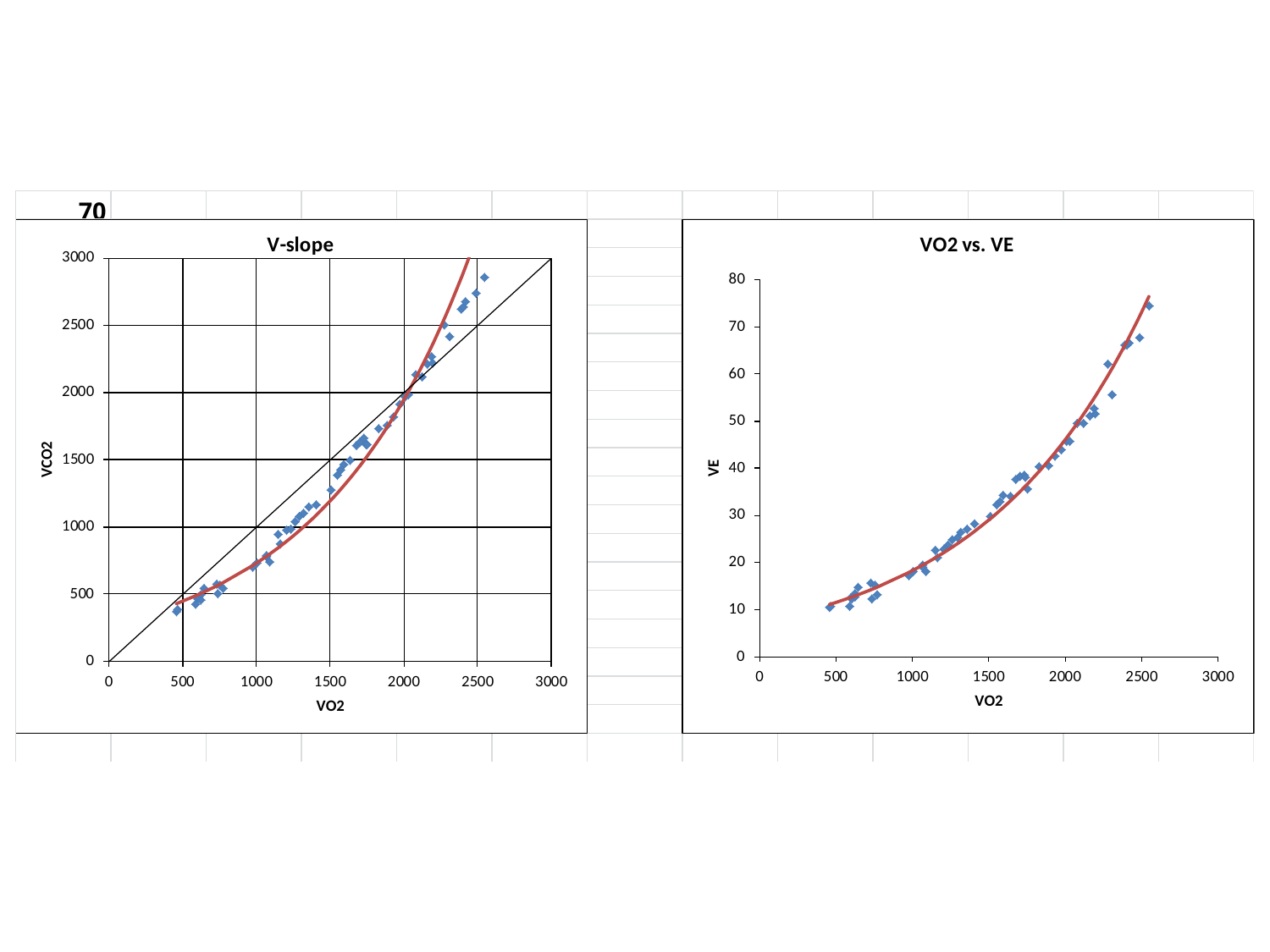

## Slide 72
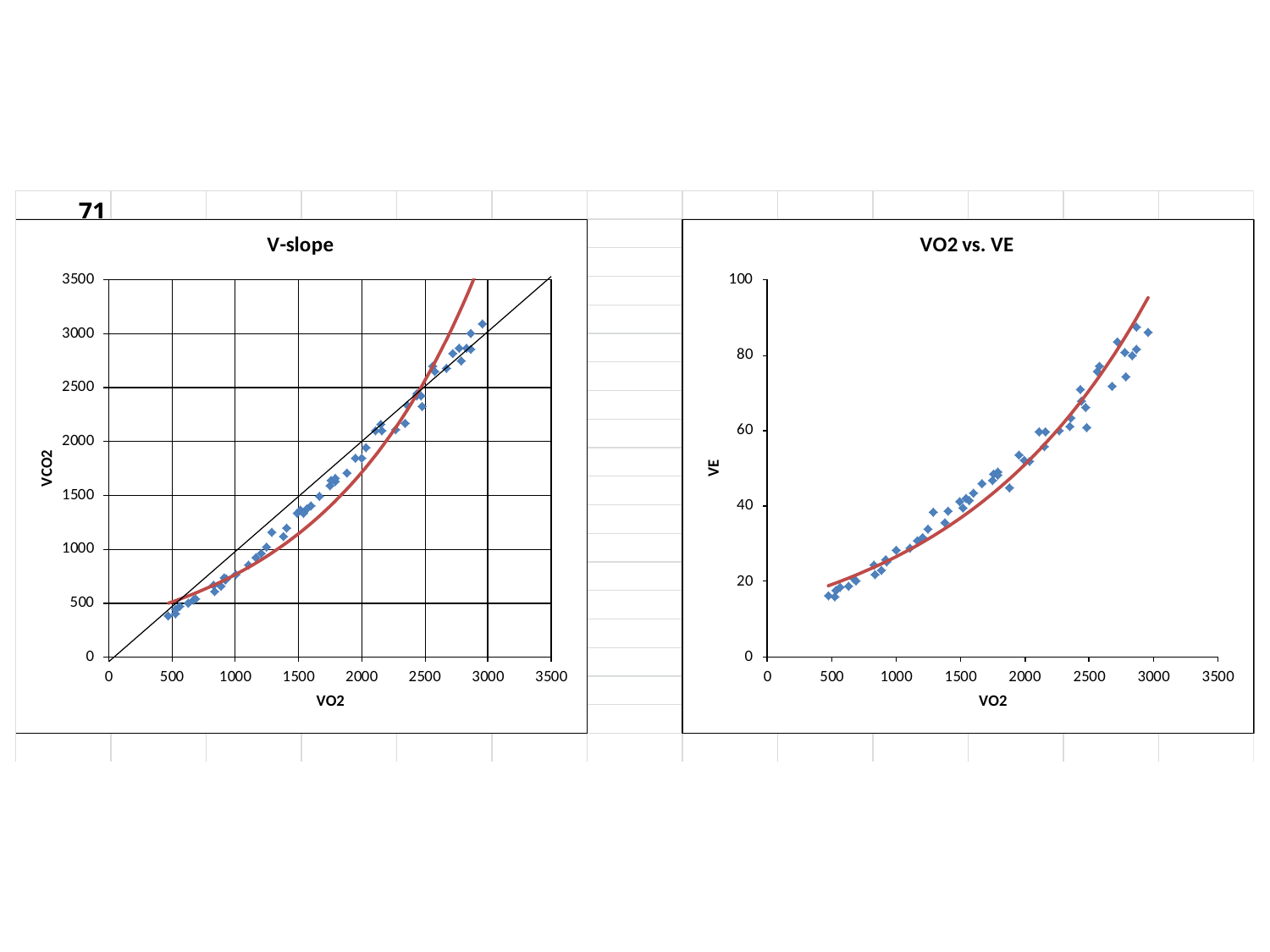

## Slide 73
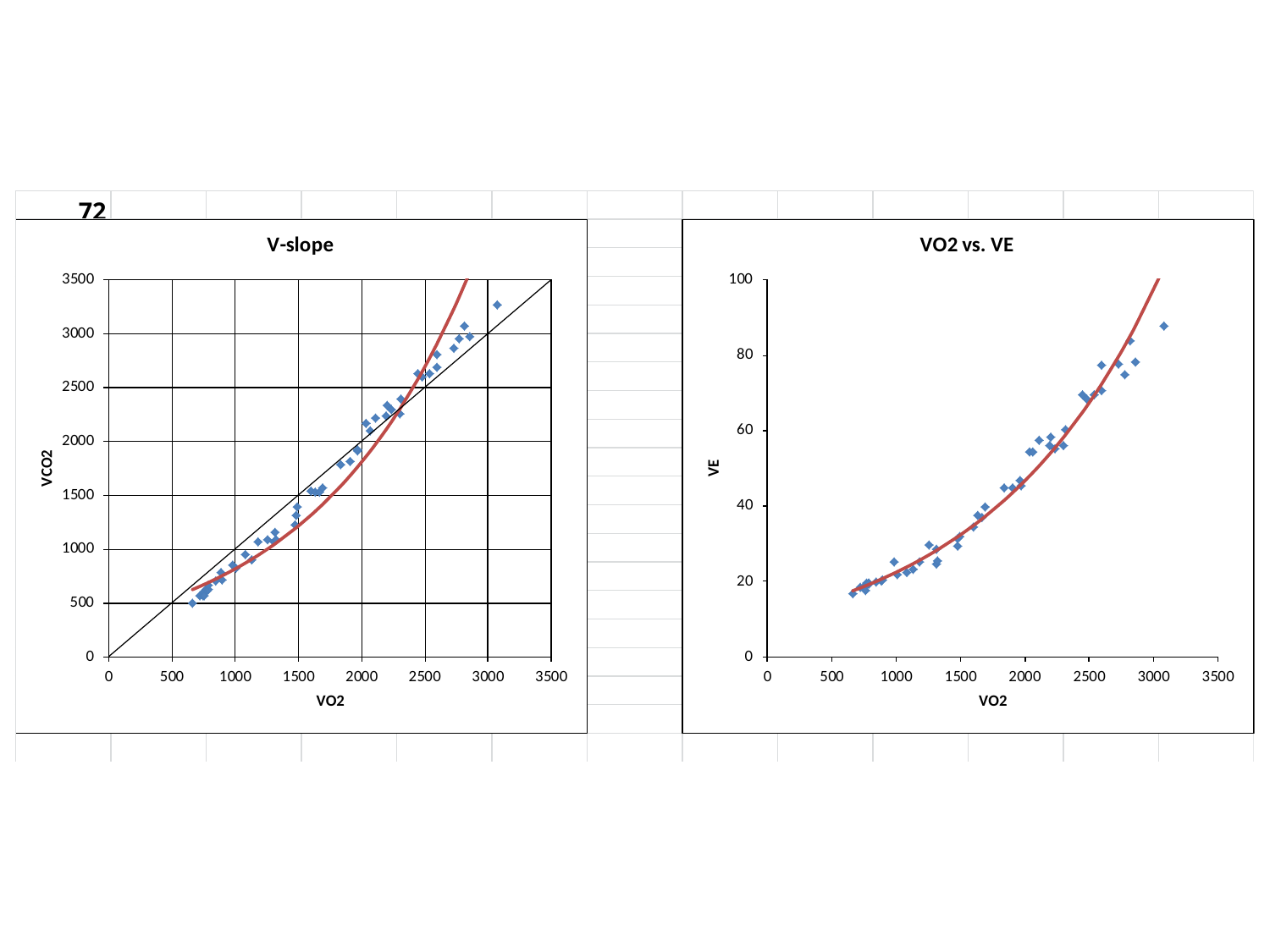

## Slide 74
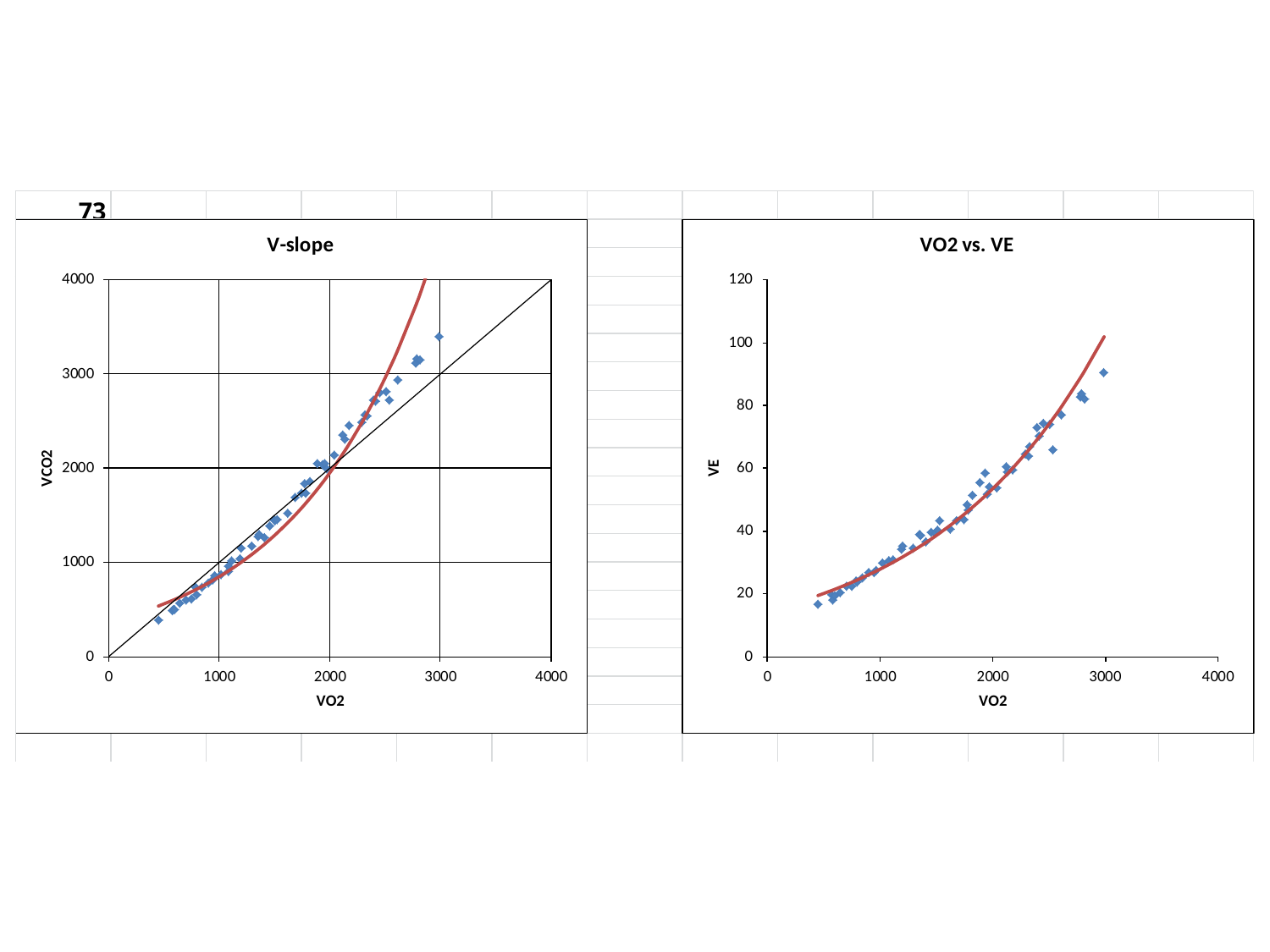

## Slide 75
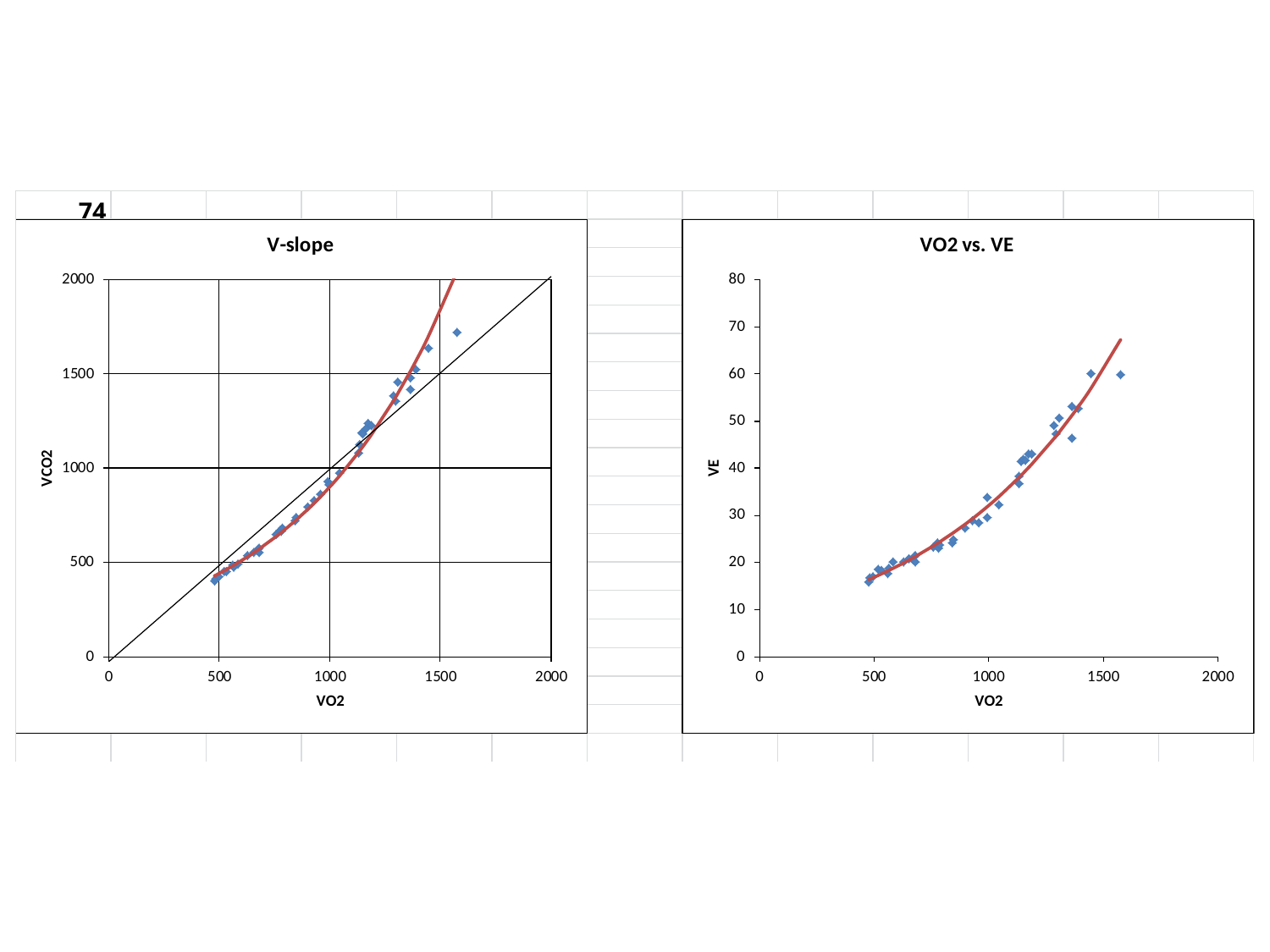

## Slide 76
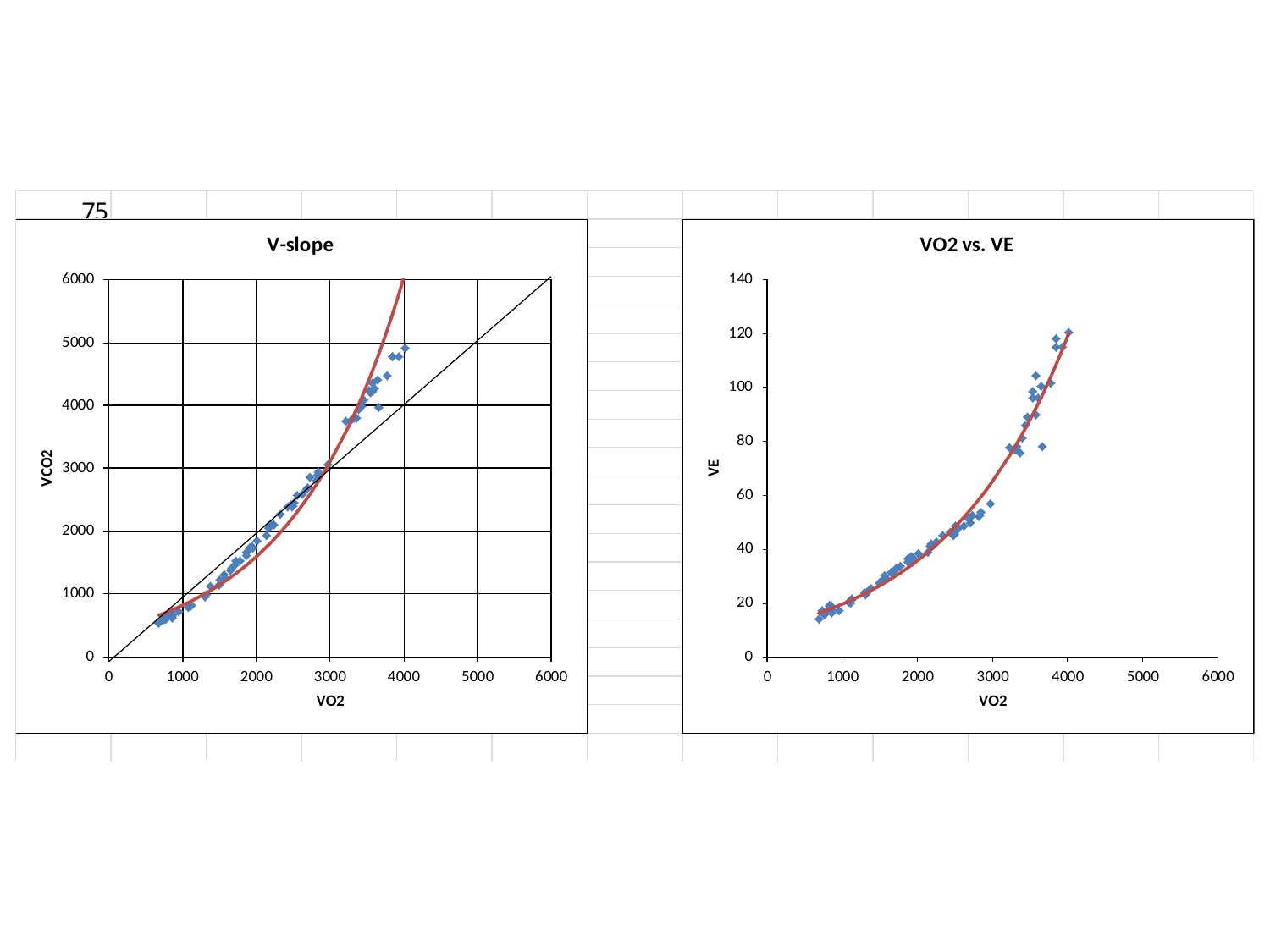

## Slide 77
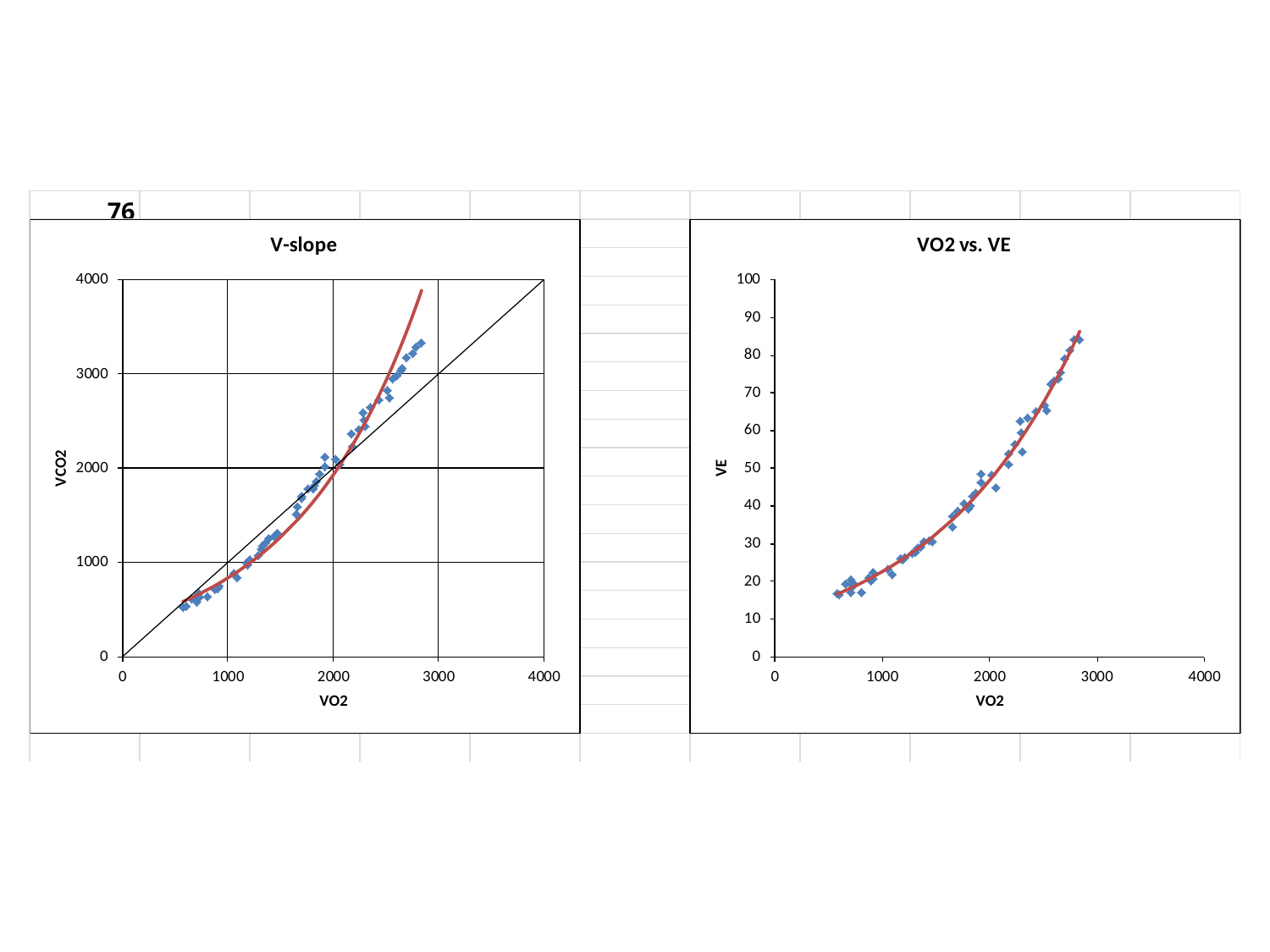

## Slide 78
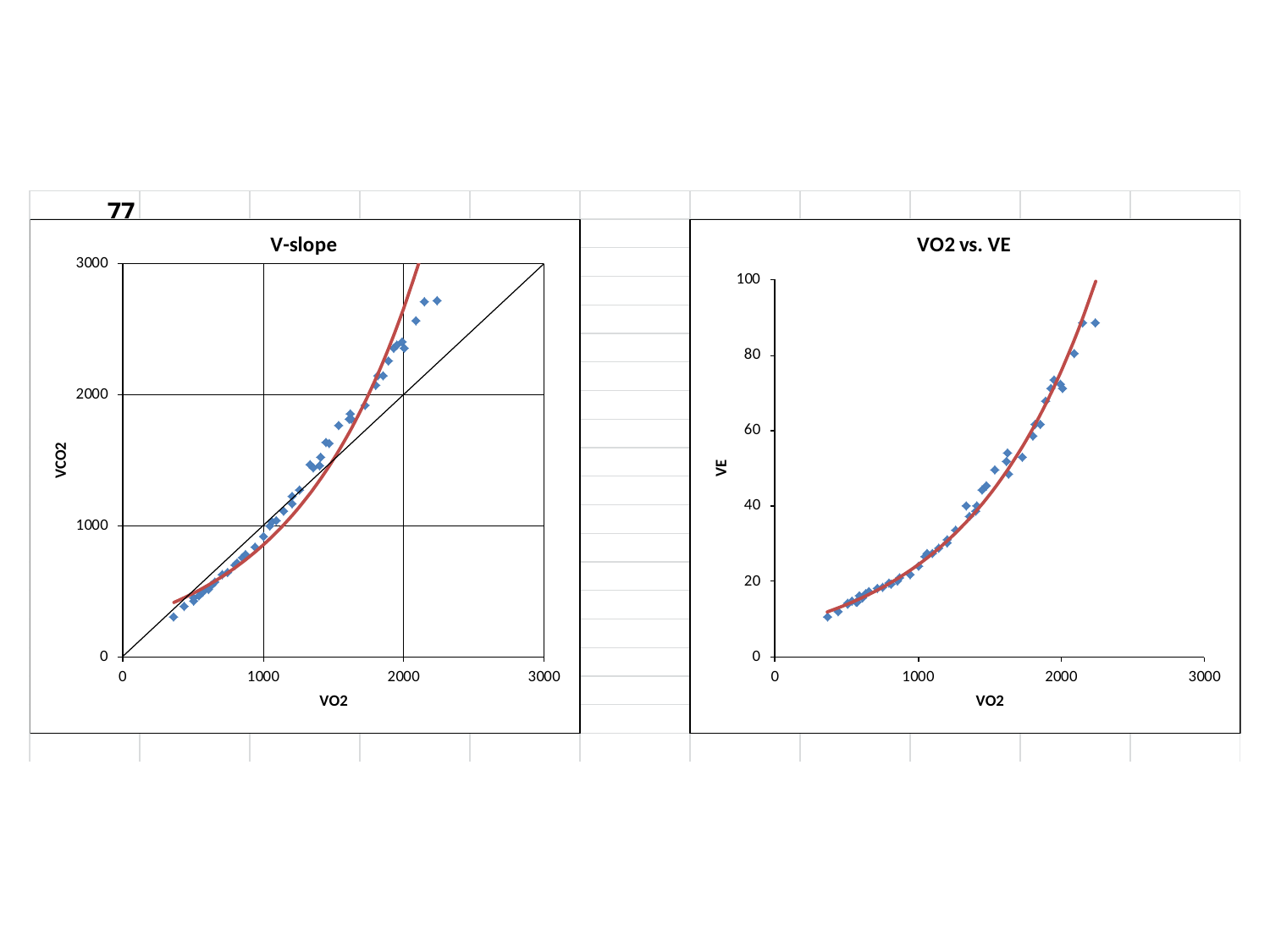

## Slide 79
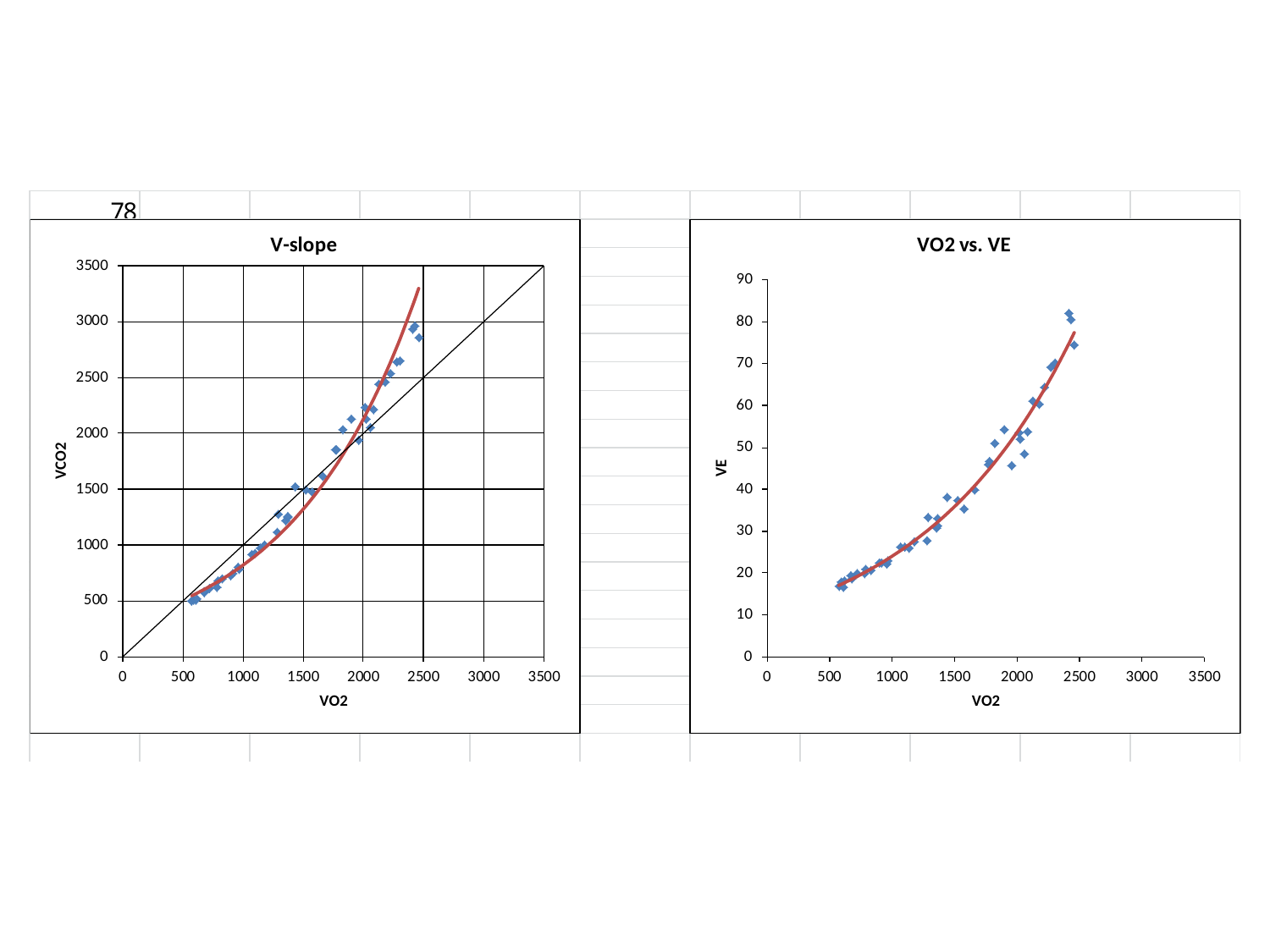

## Slide 80
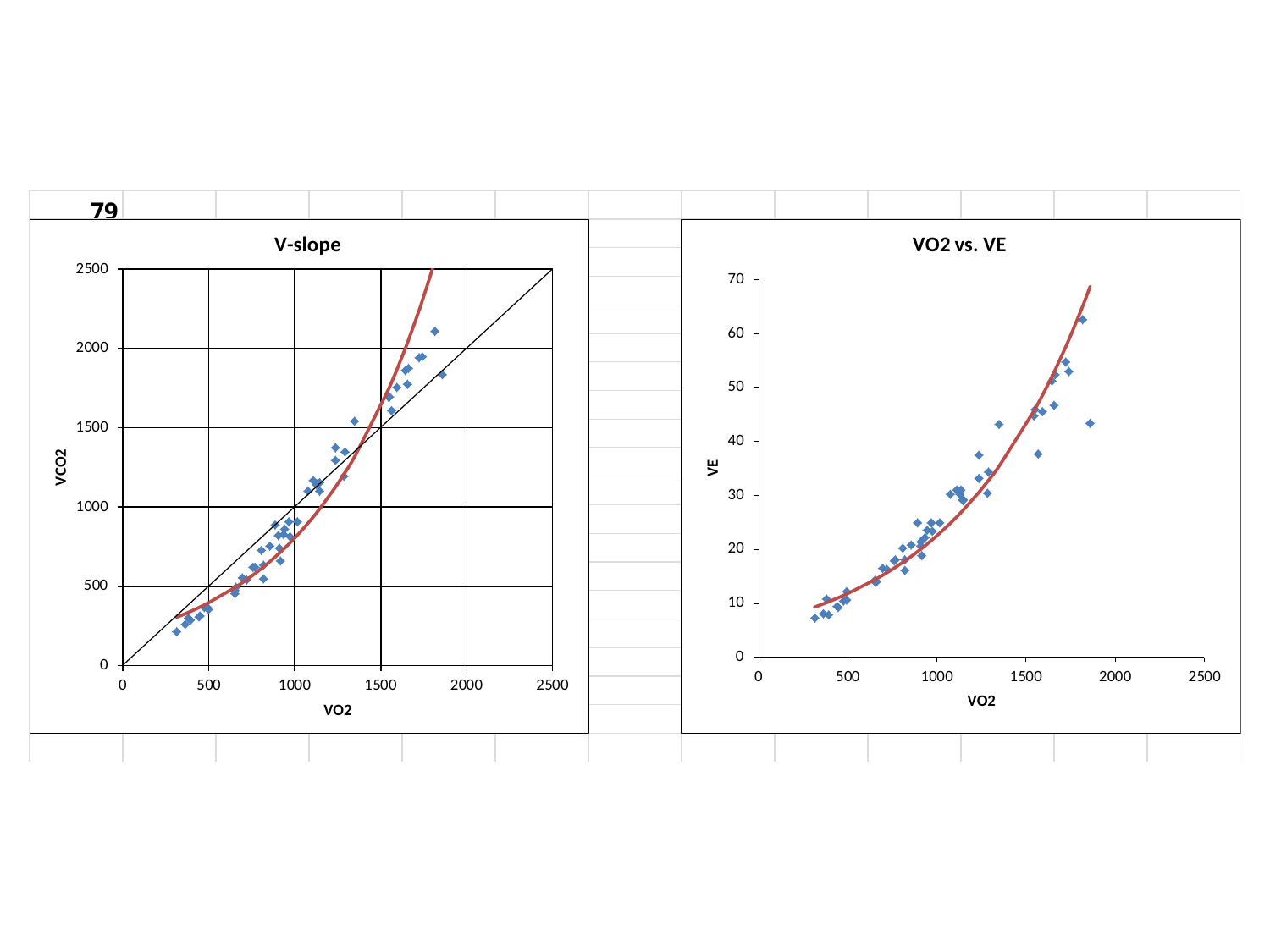

## Slide 81
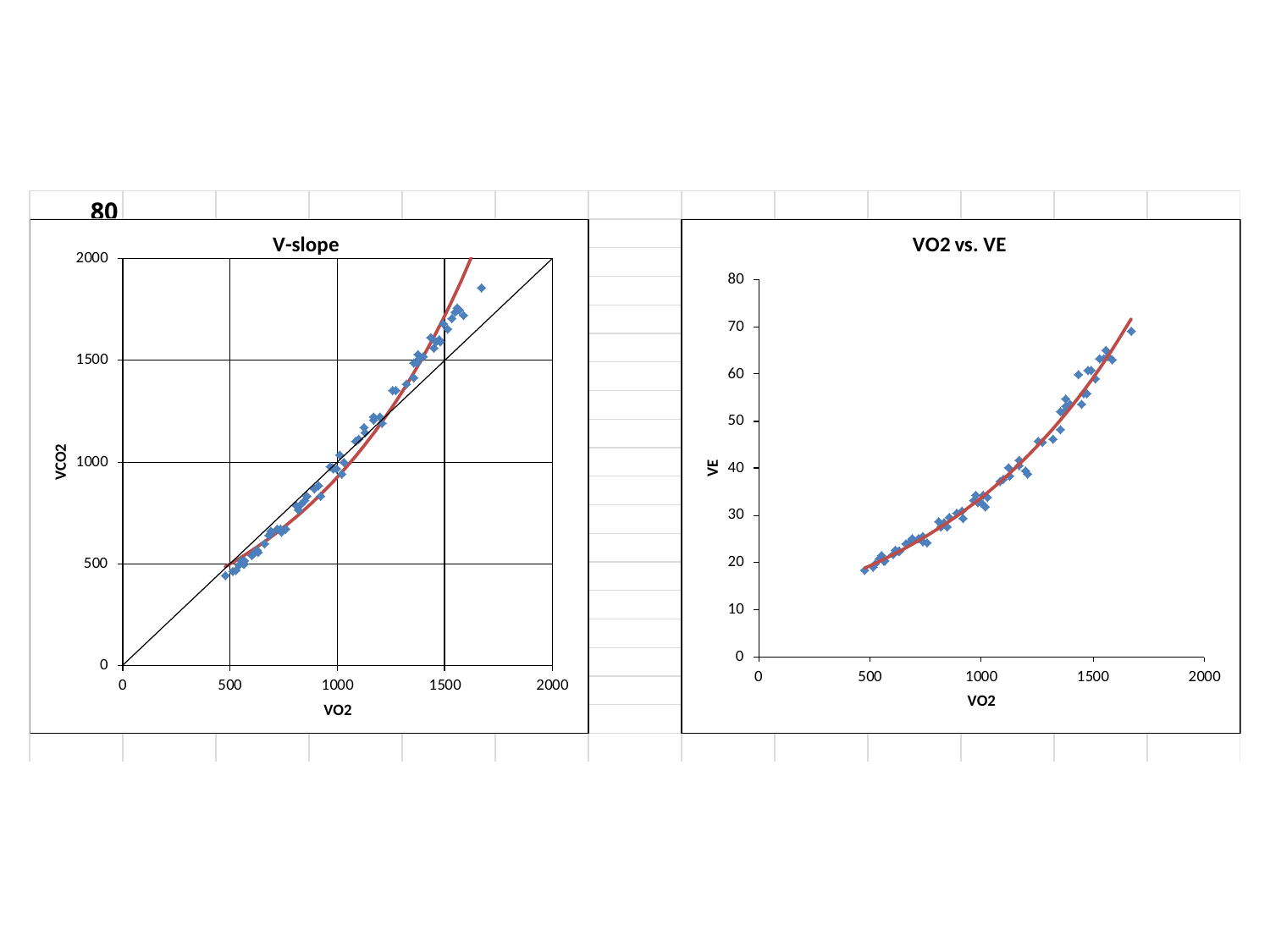

## Slide 82
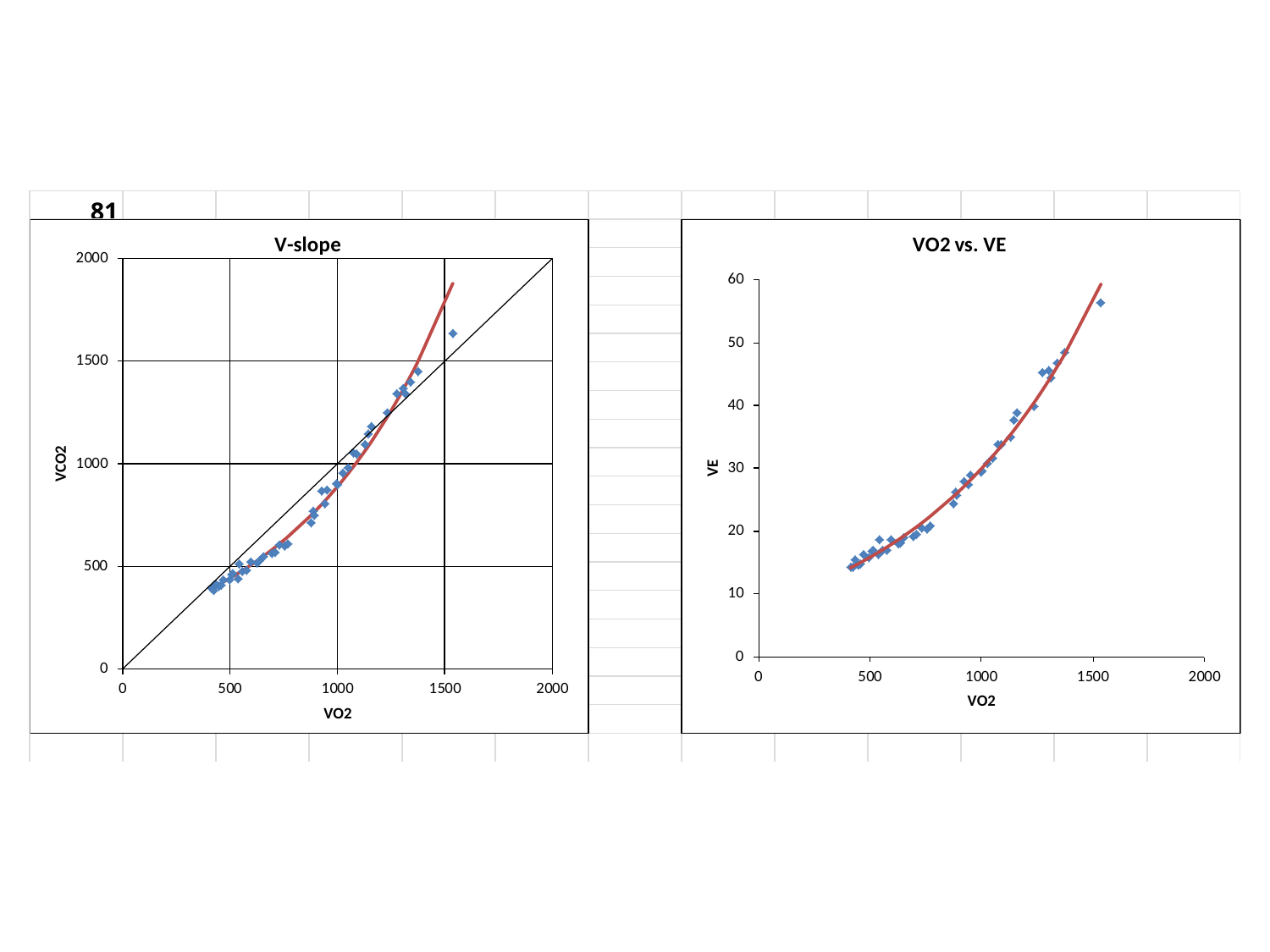

## Slide 83
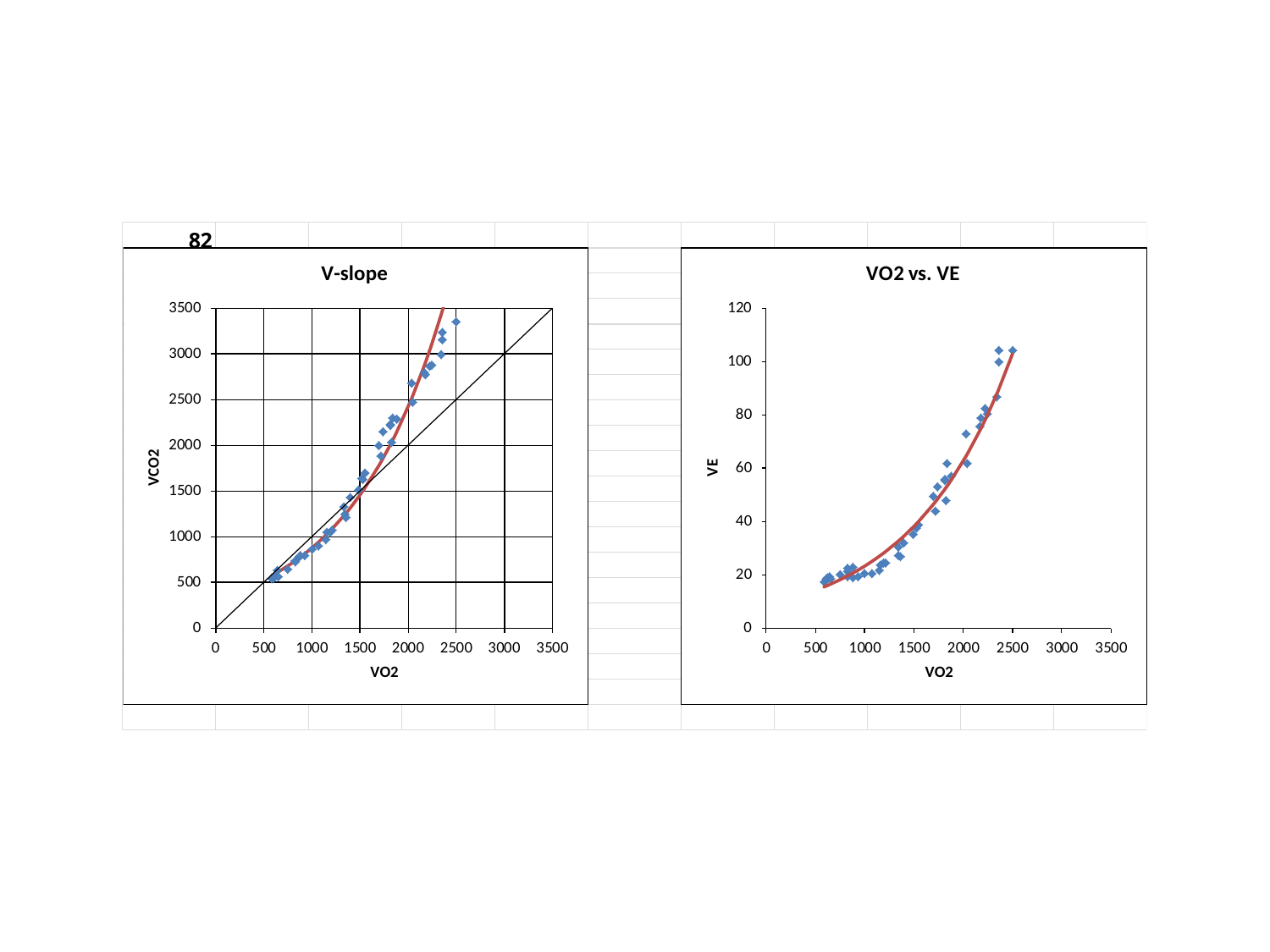

## Slide 84
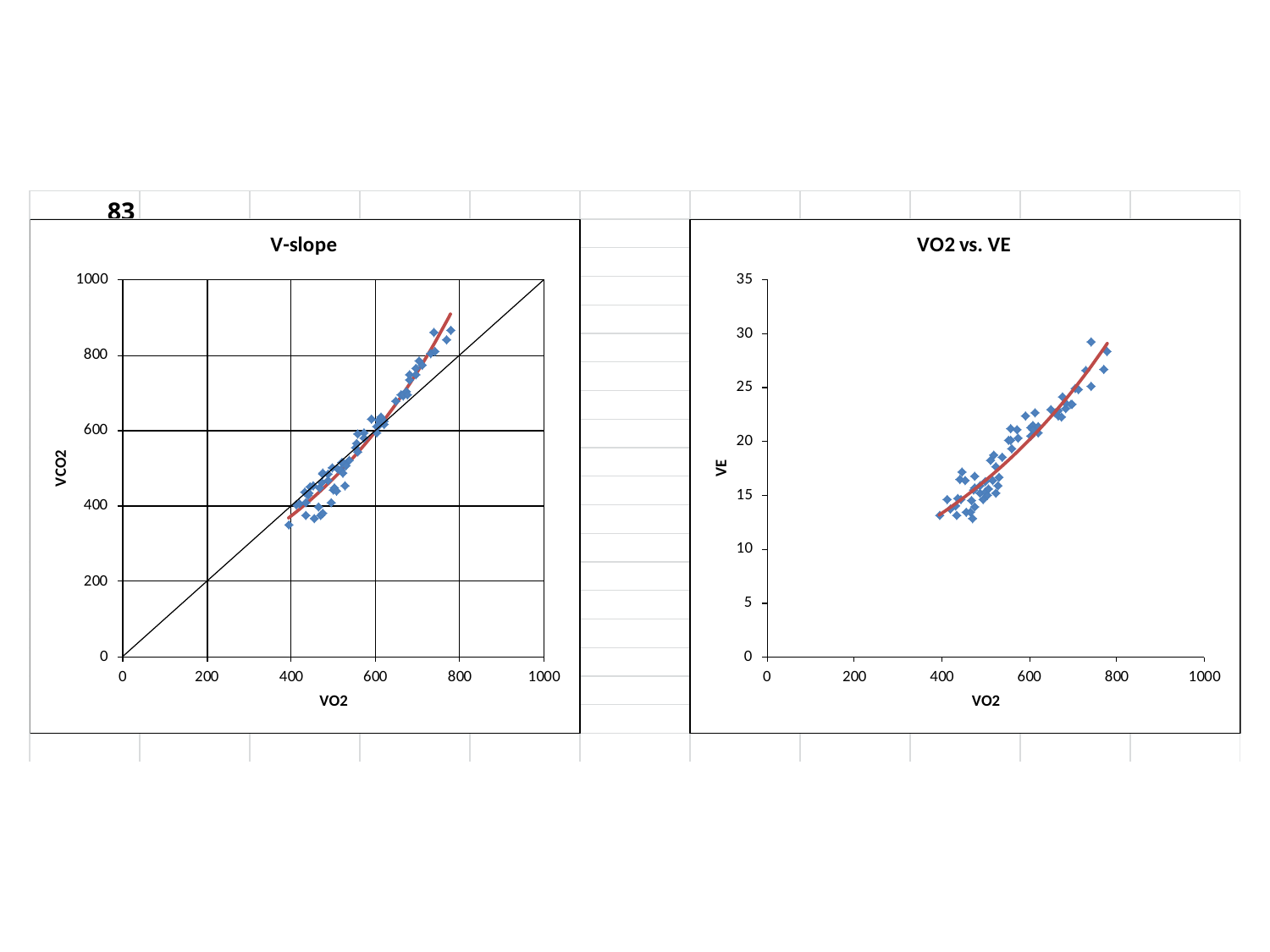

## Slide 85
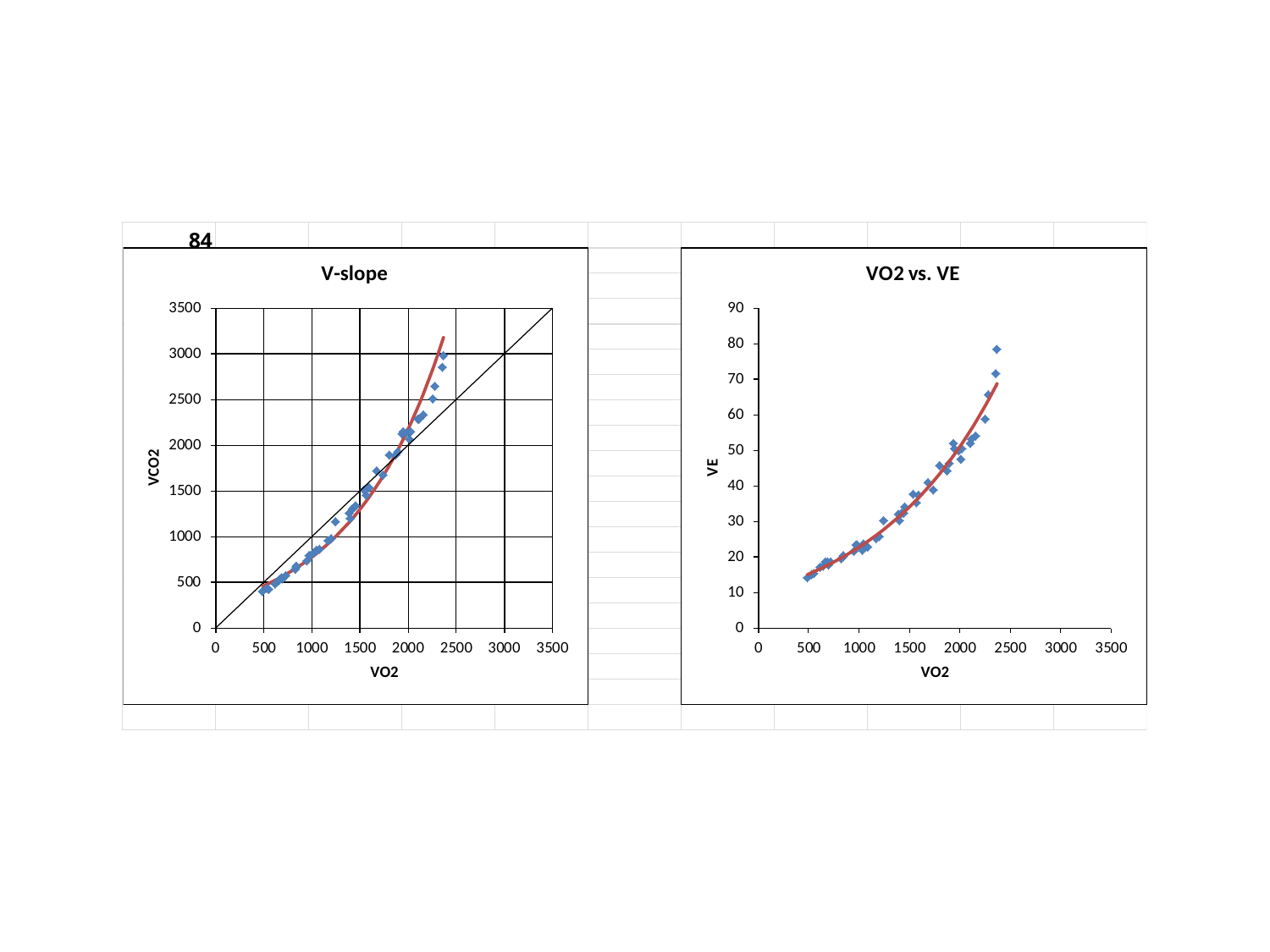

## Slide 86
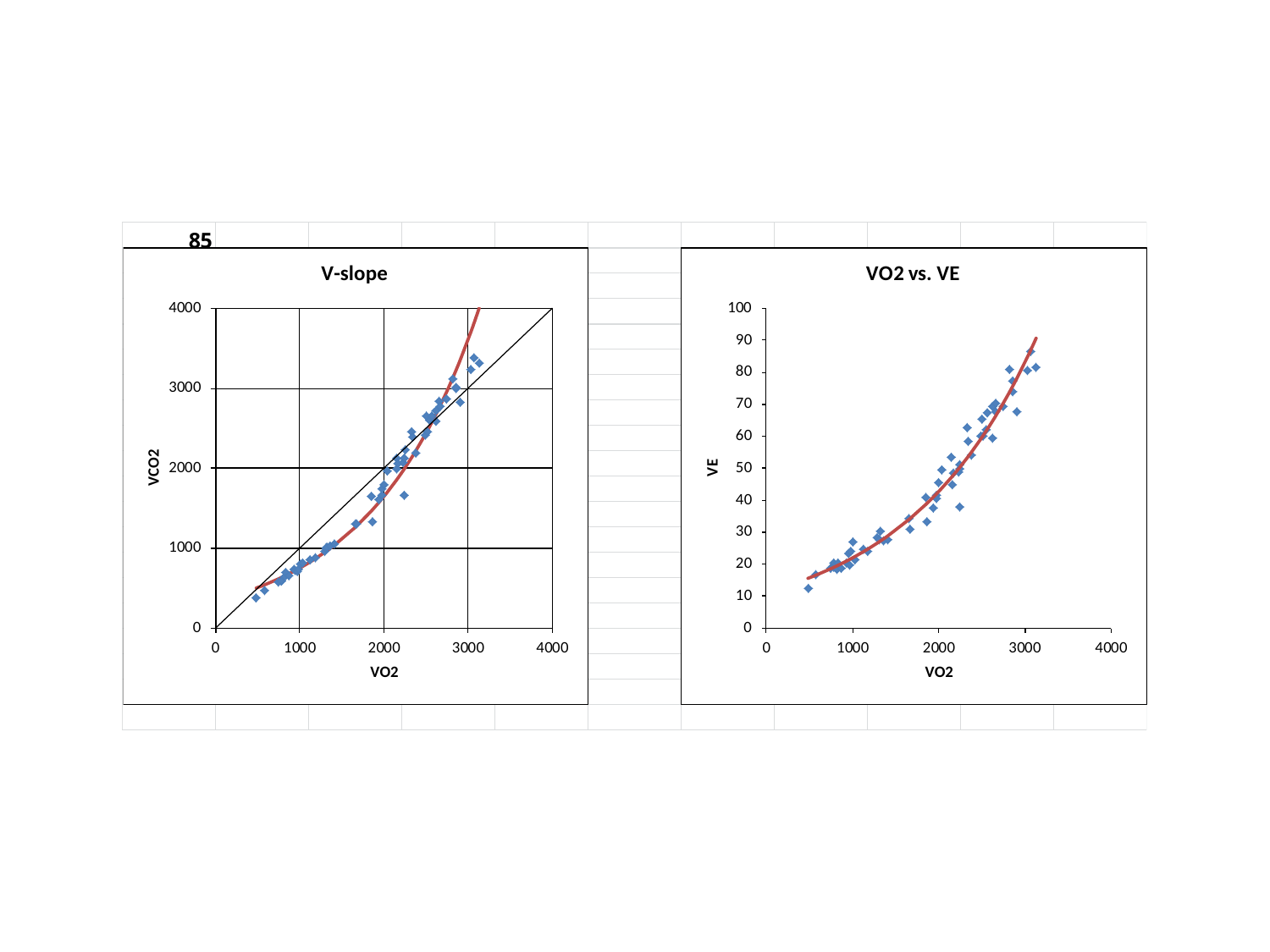

## Slide 87
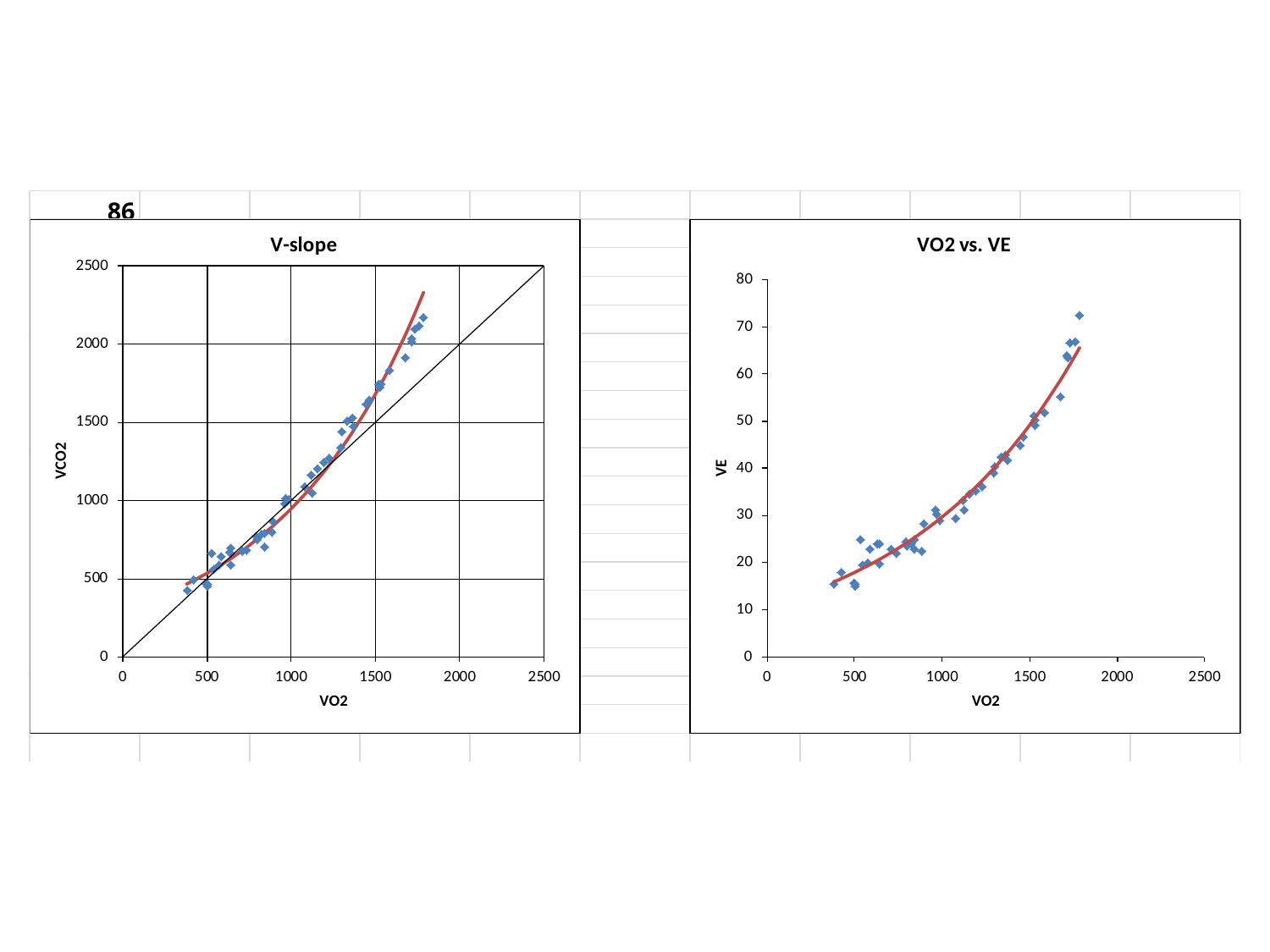

## Slide 88
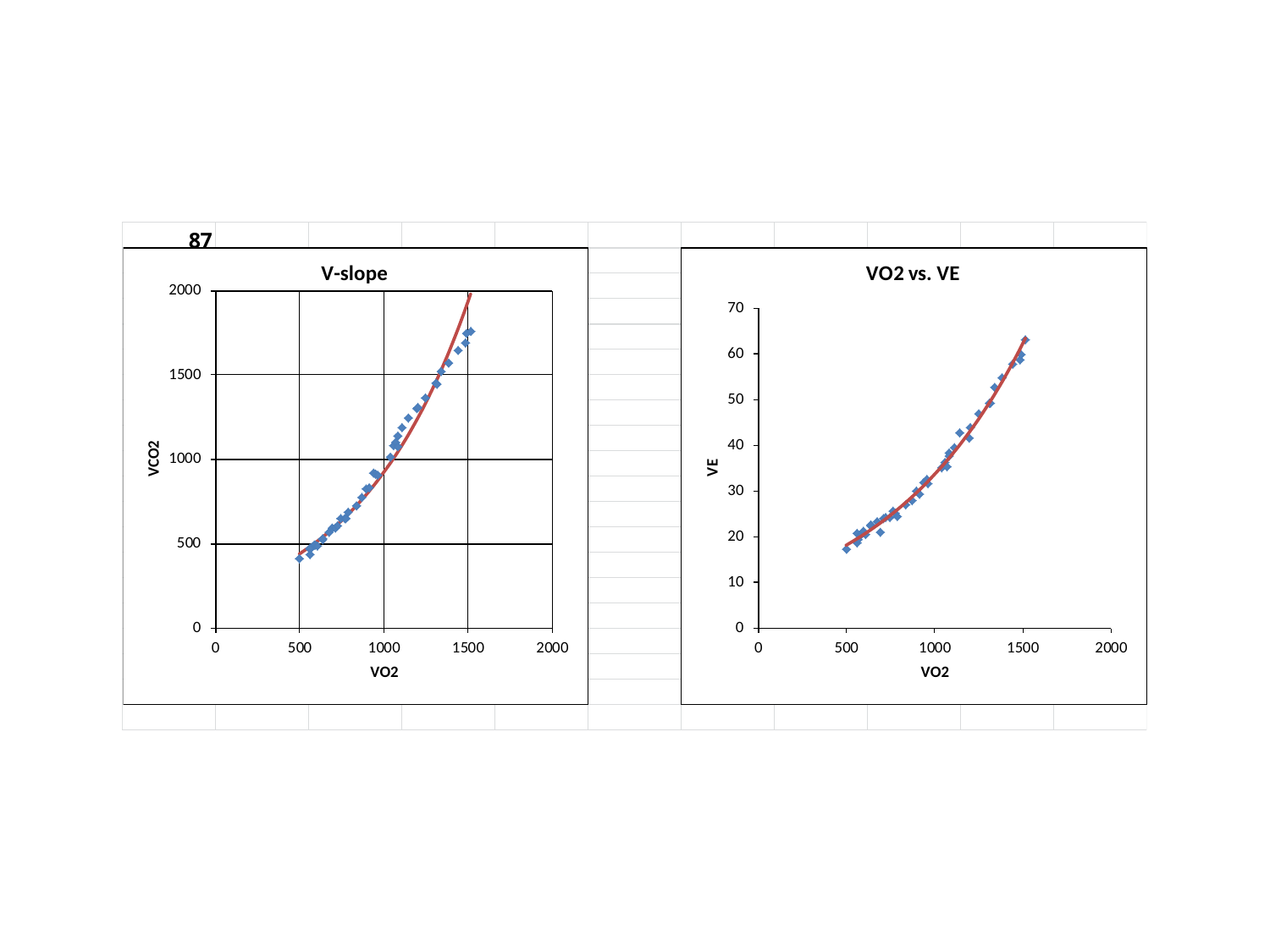

## Slide 89
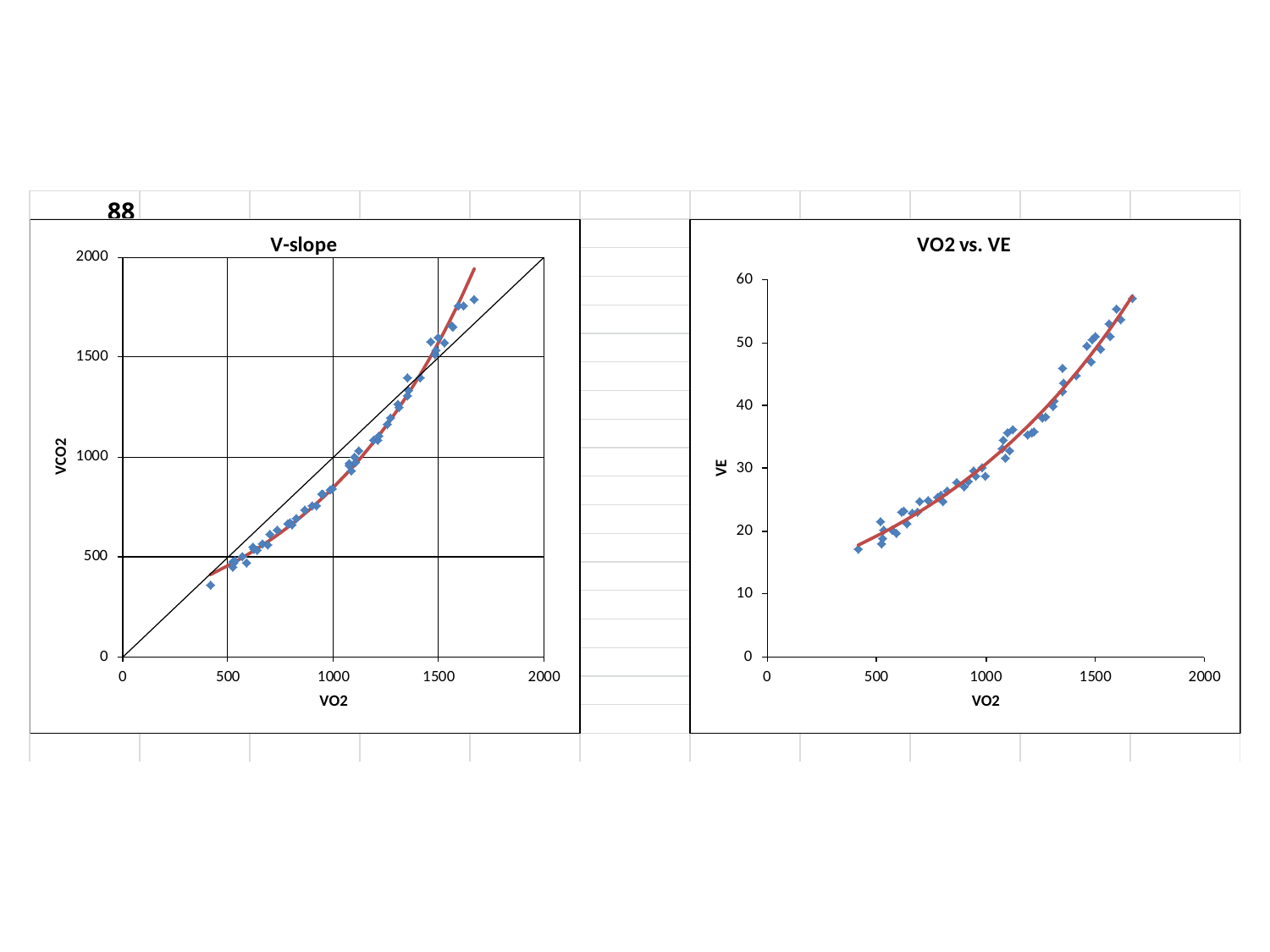

## Slide 90
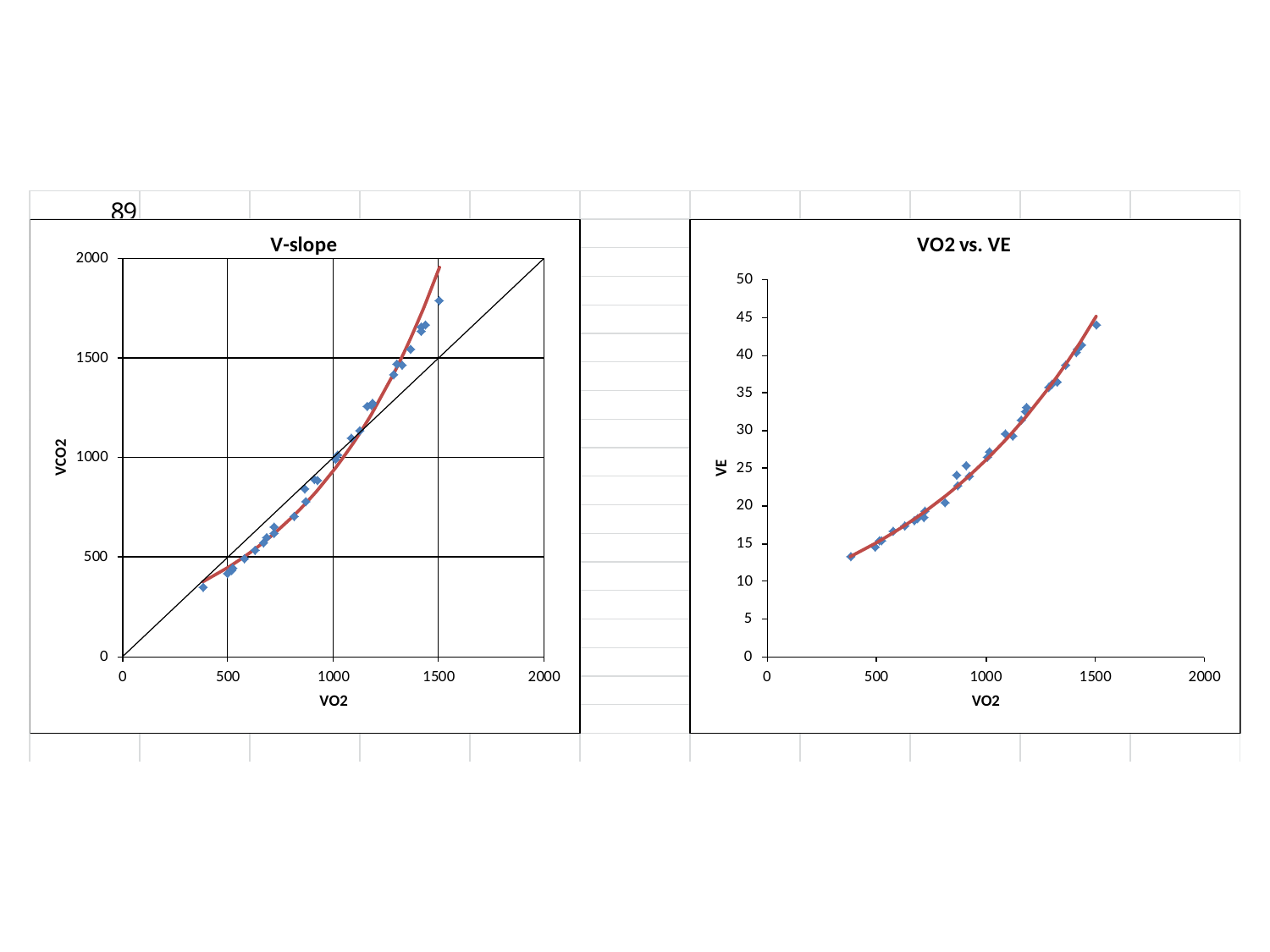

## Slide 91
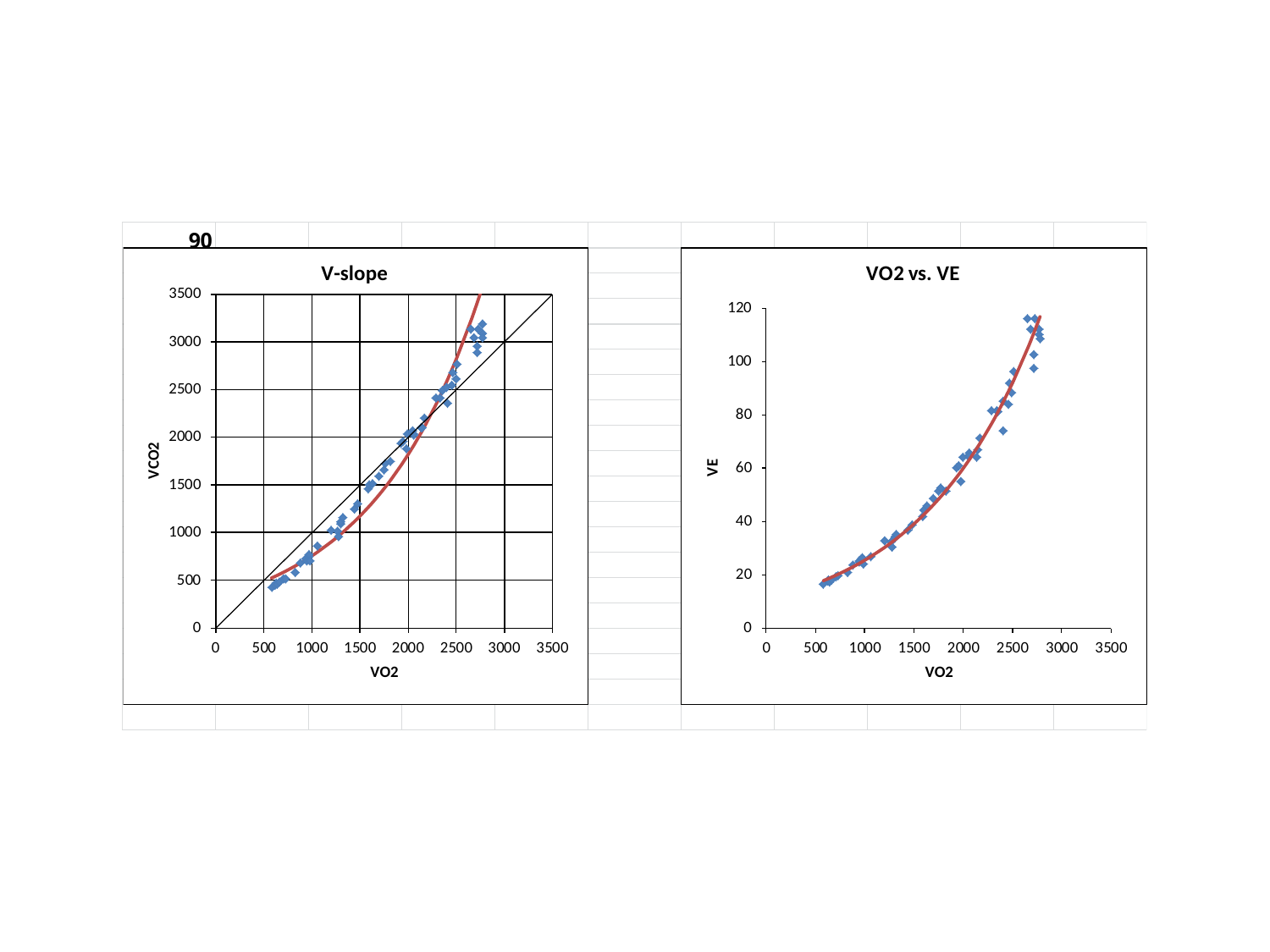

## Slide 92
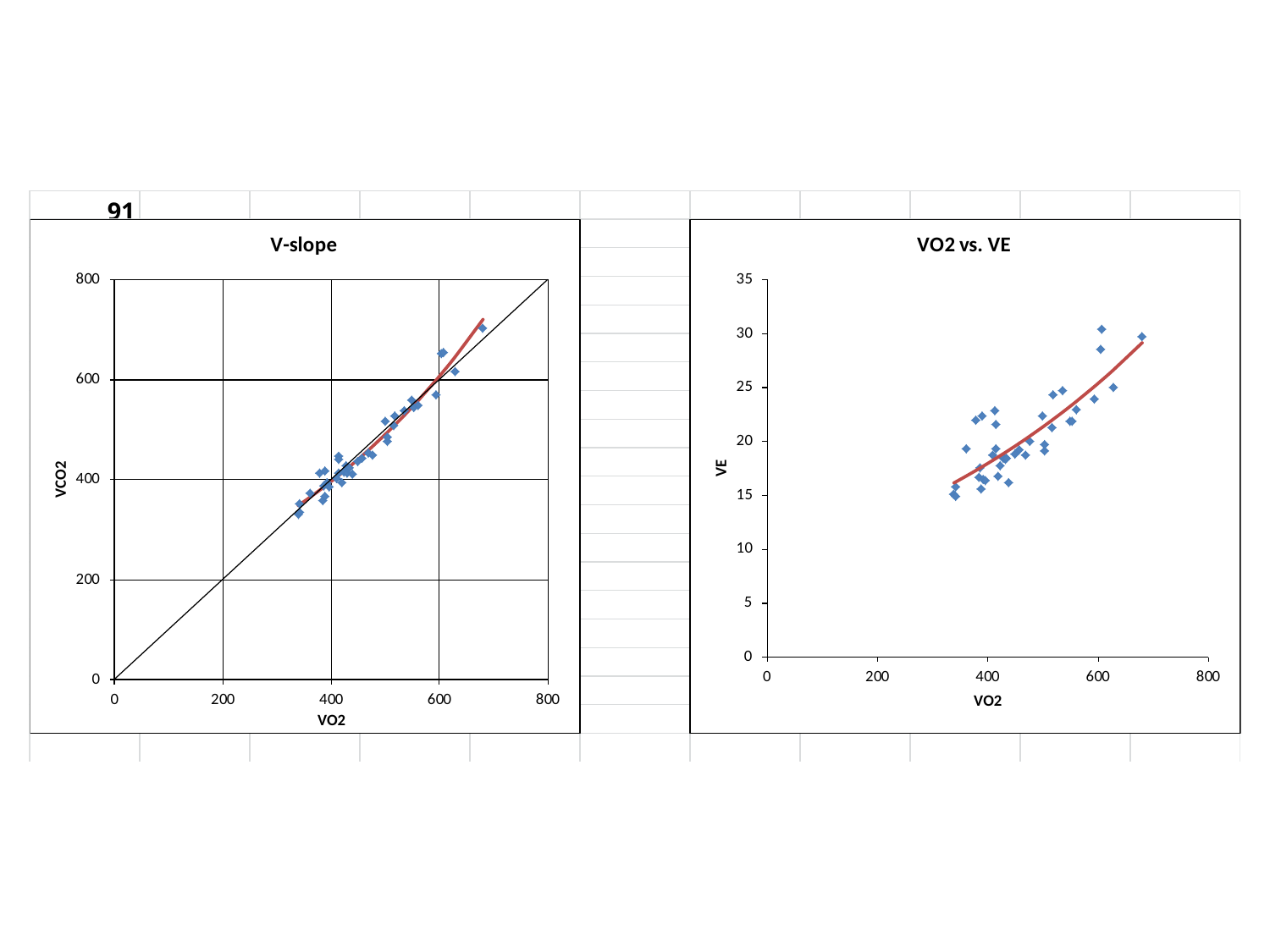

## Slide 93
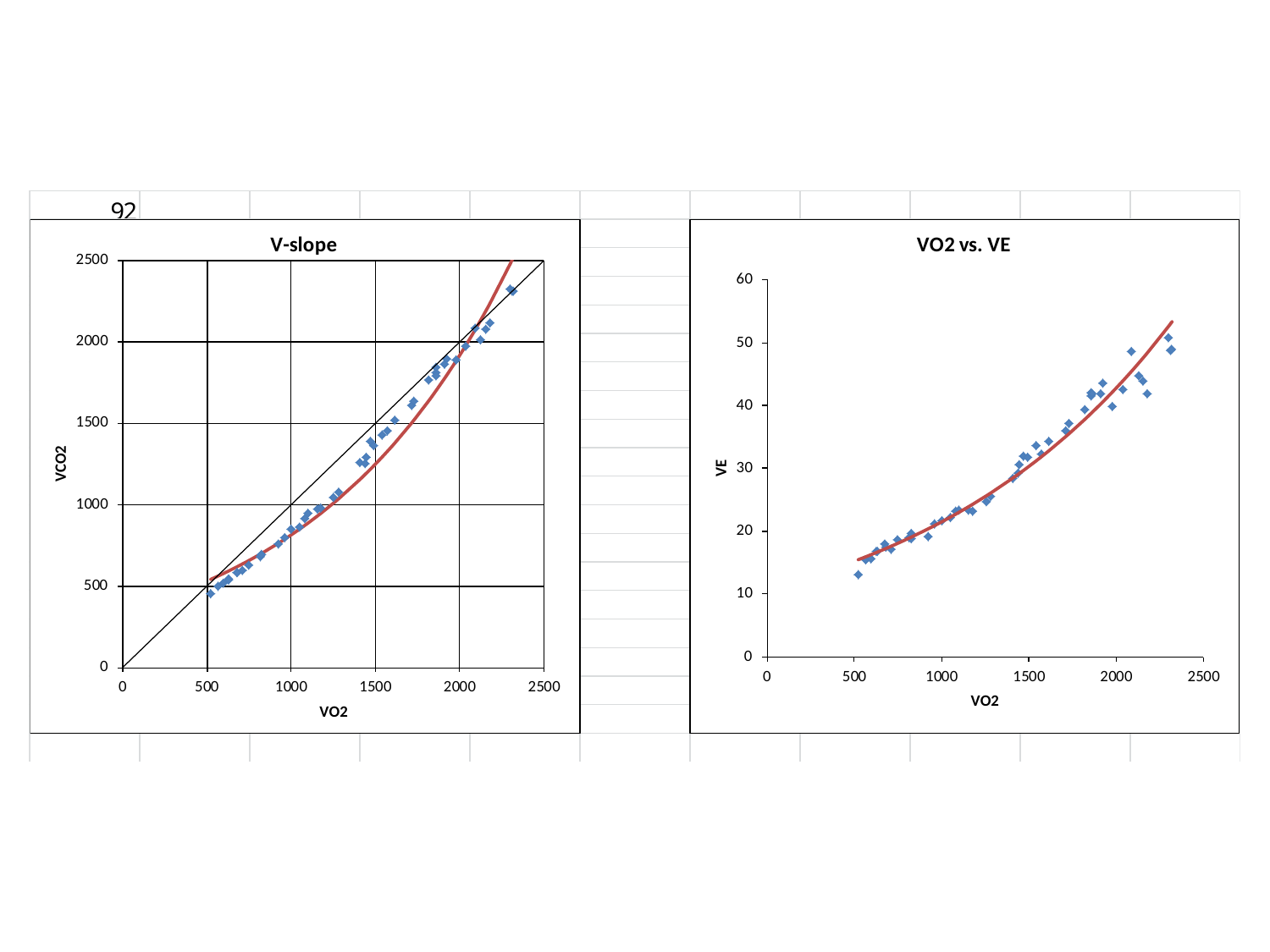

## Slide 94
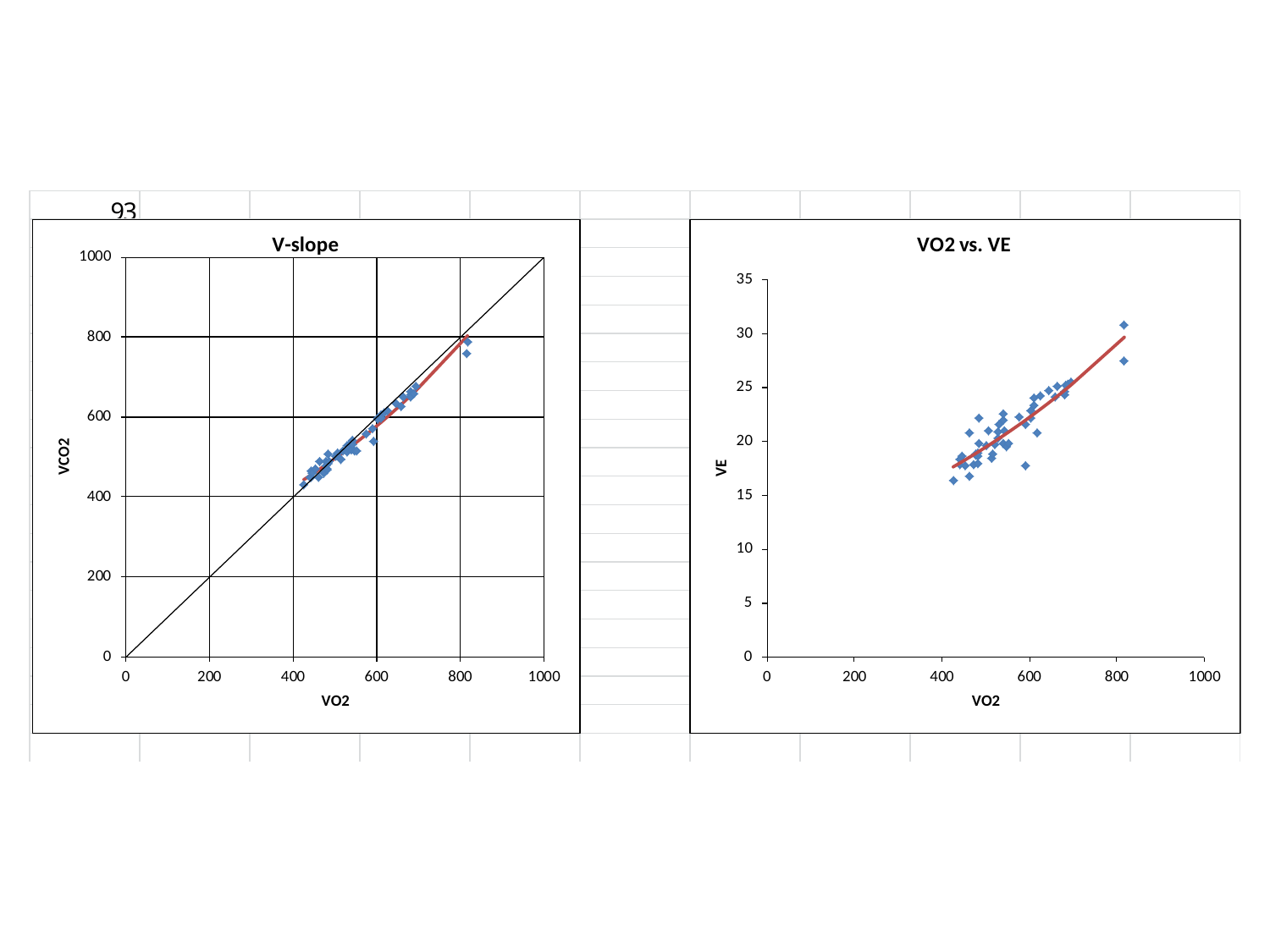

## Slide 95
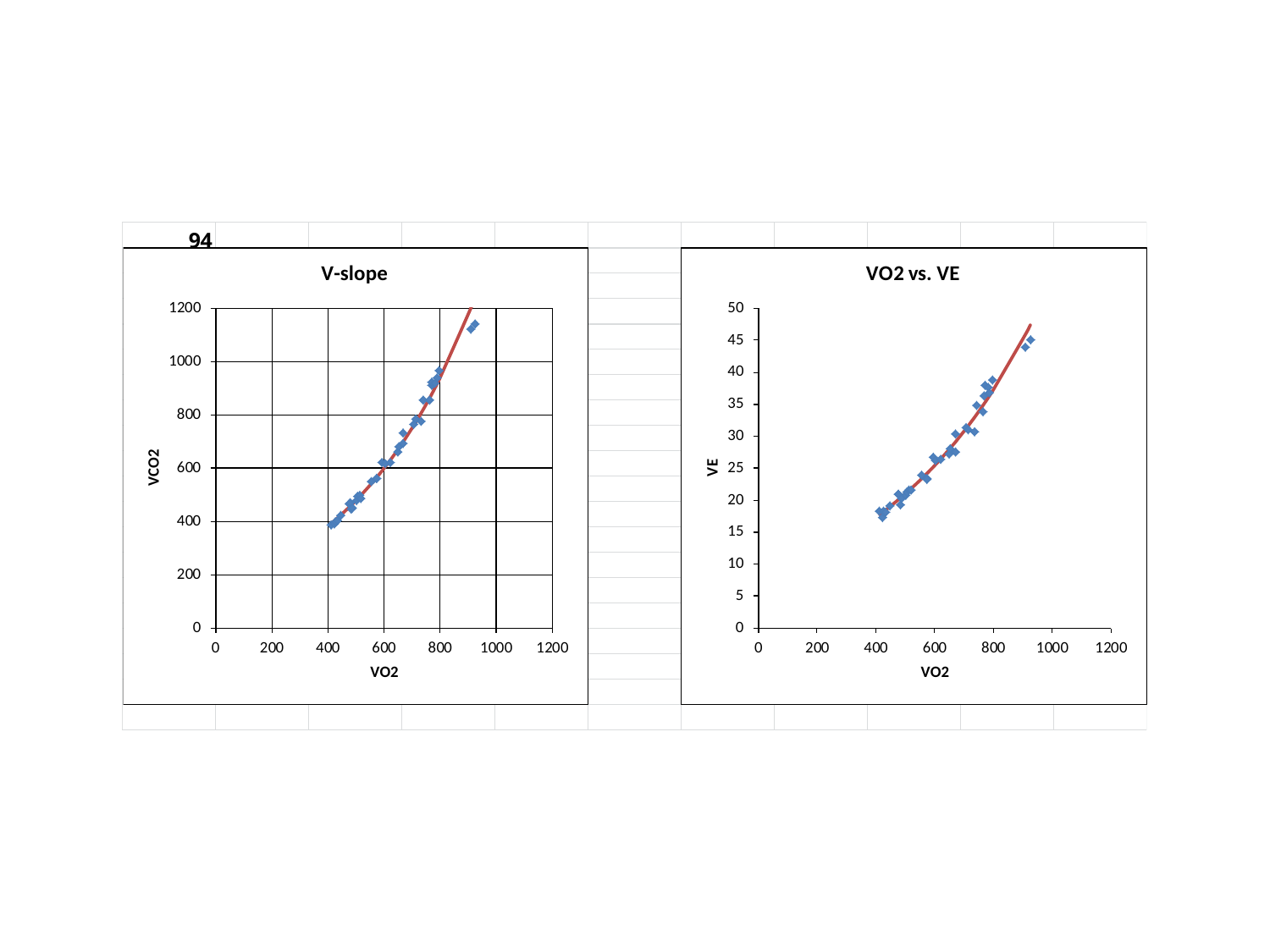

## Slide 96
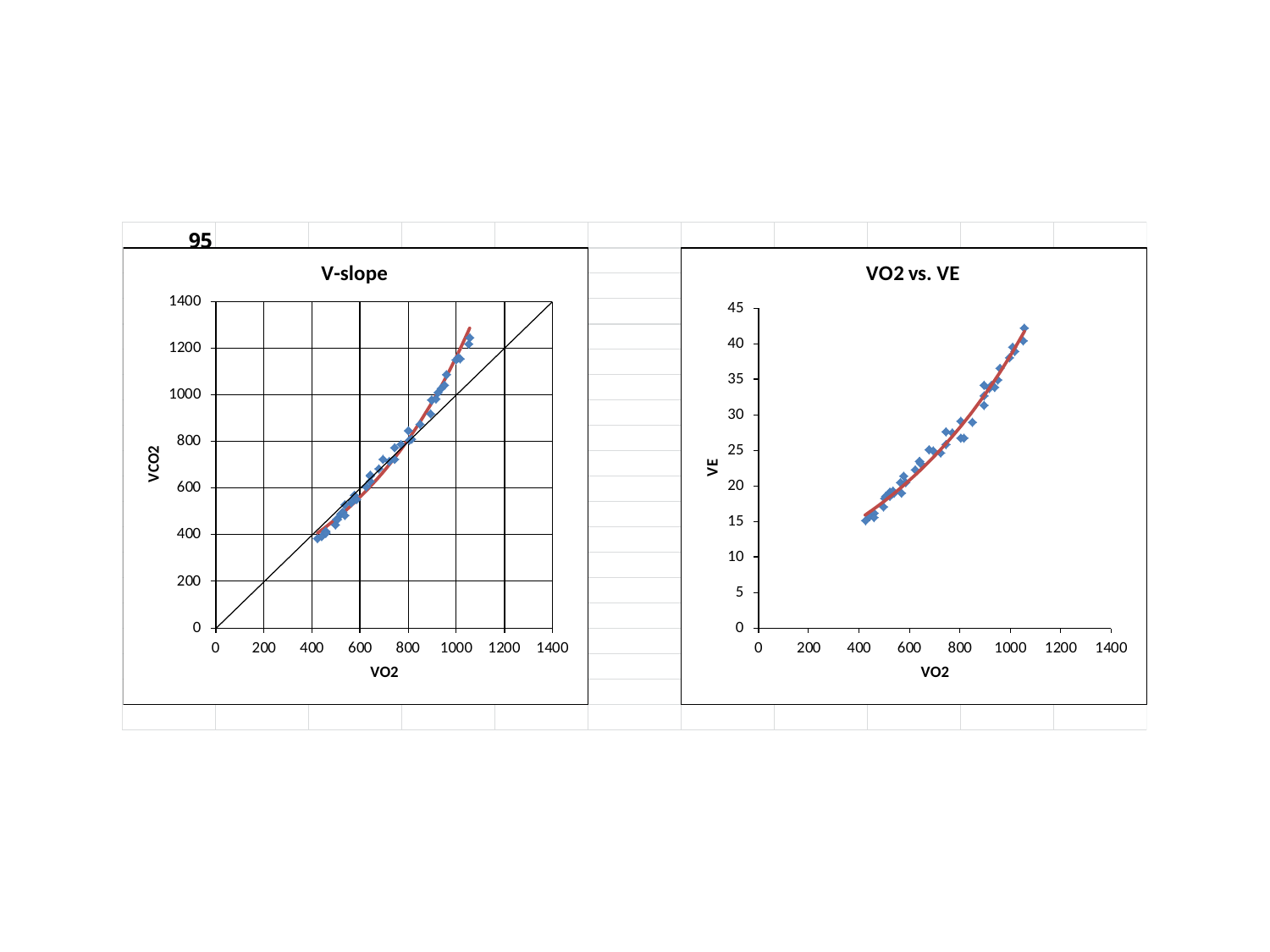

## Slide 97
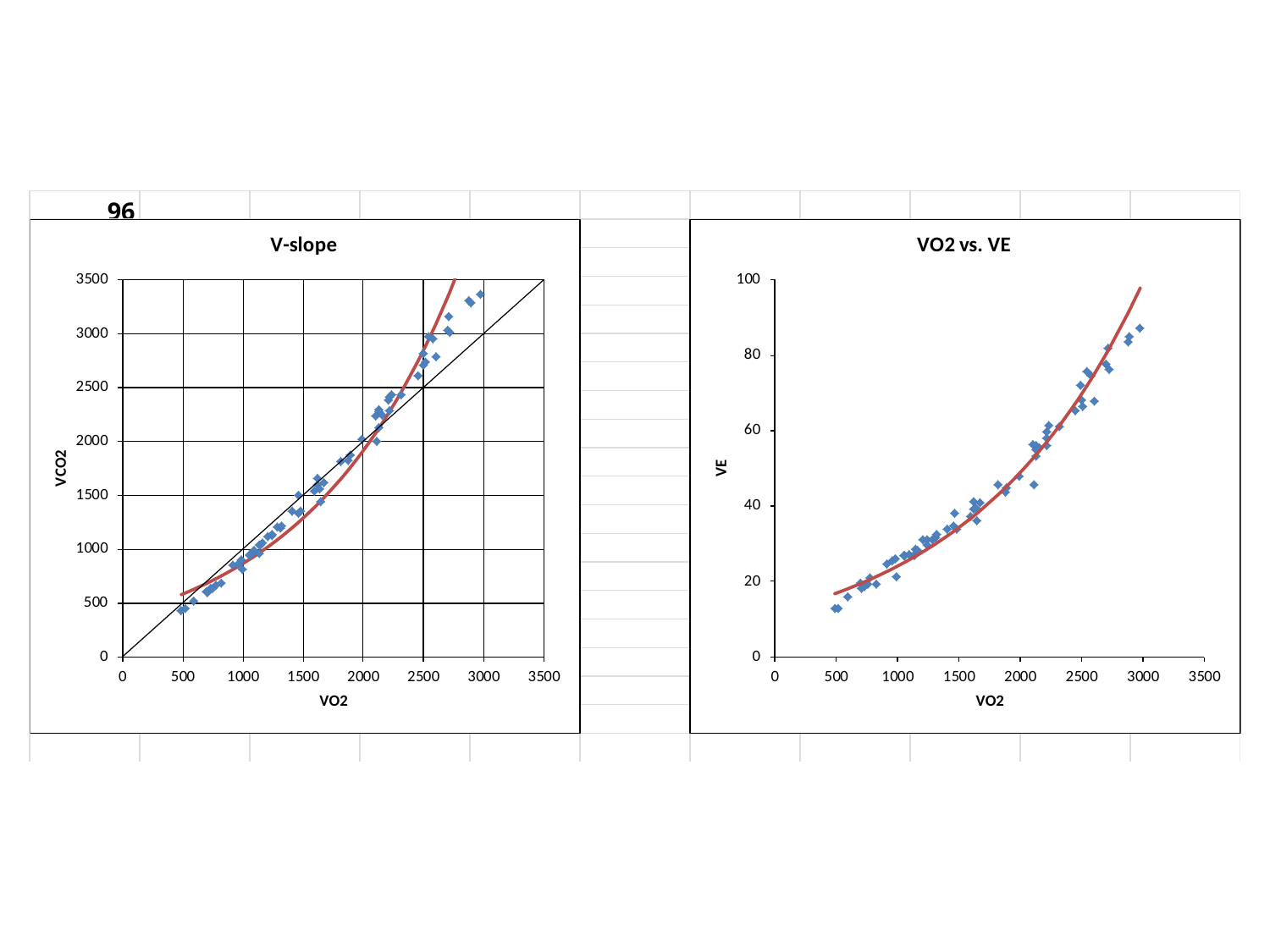

## Slide 98
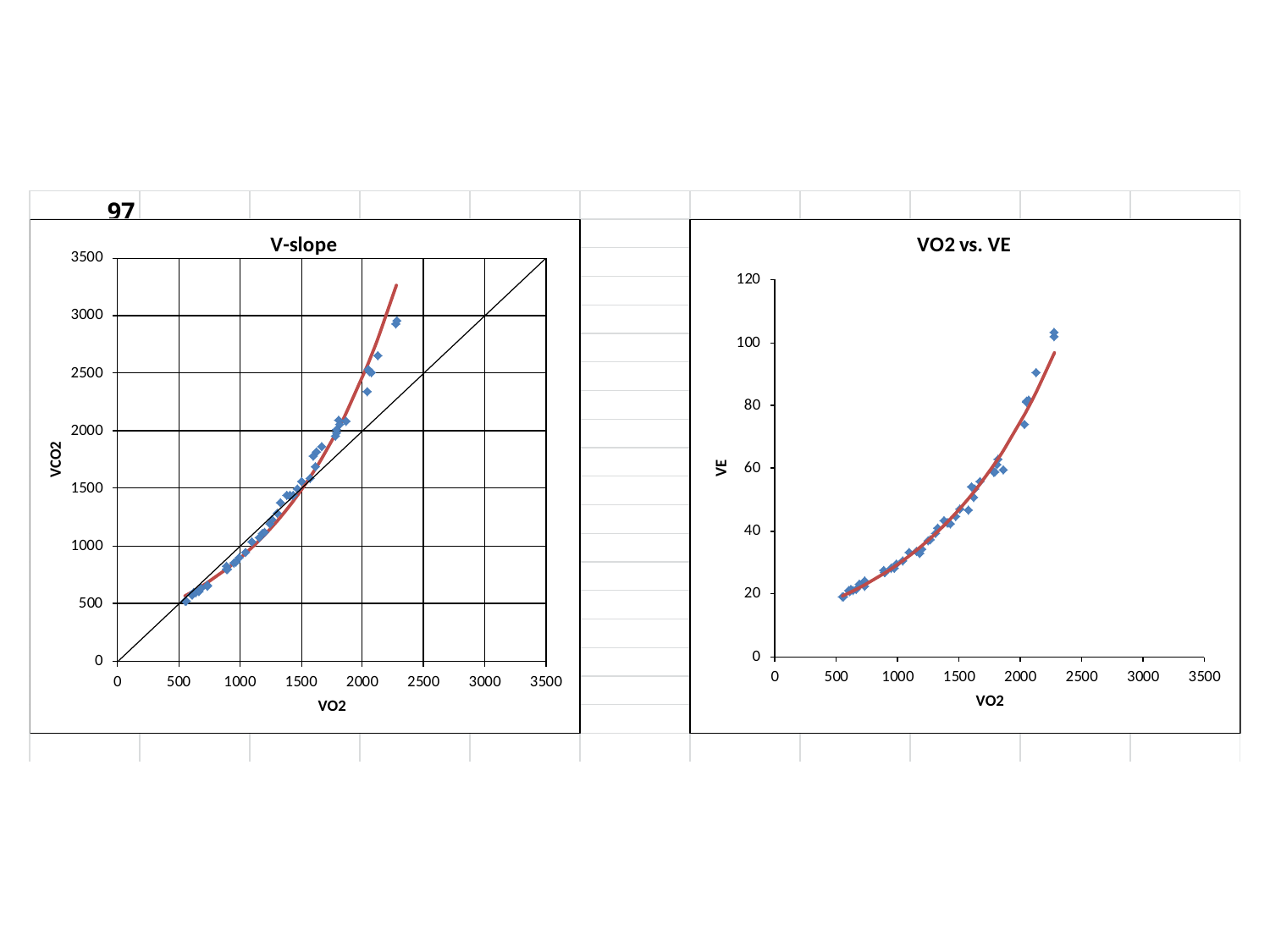

## Slide 99
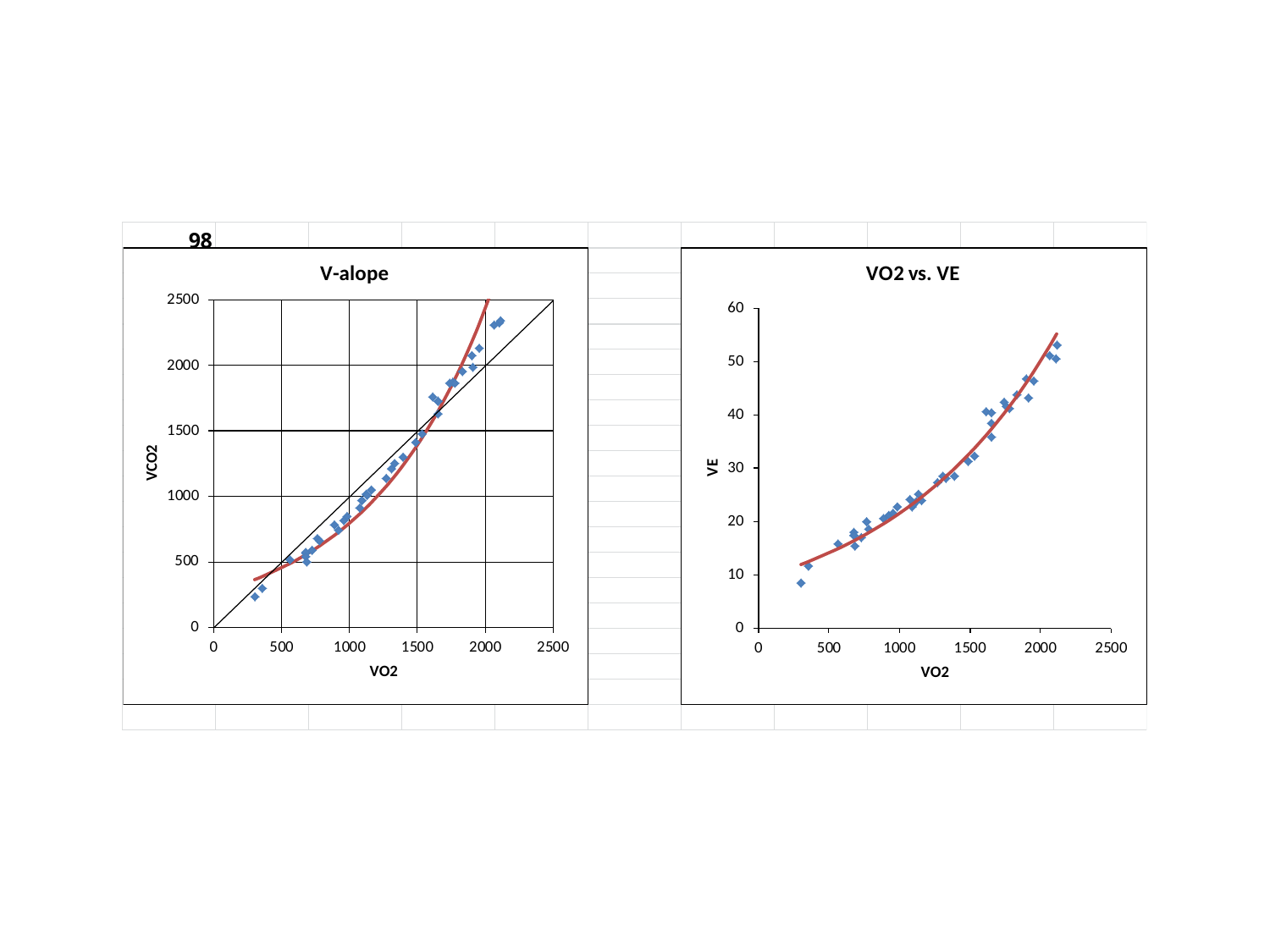

## Slide 100
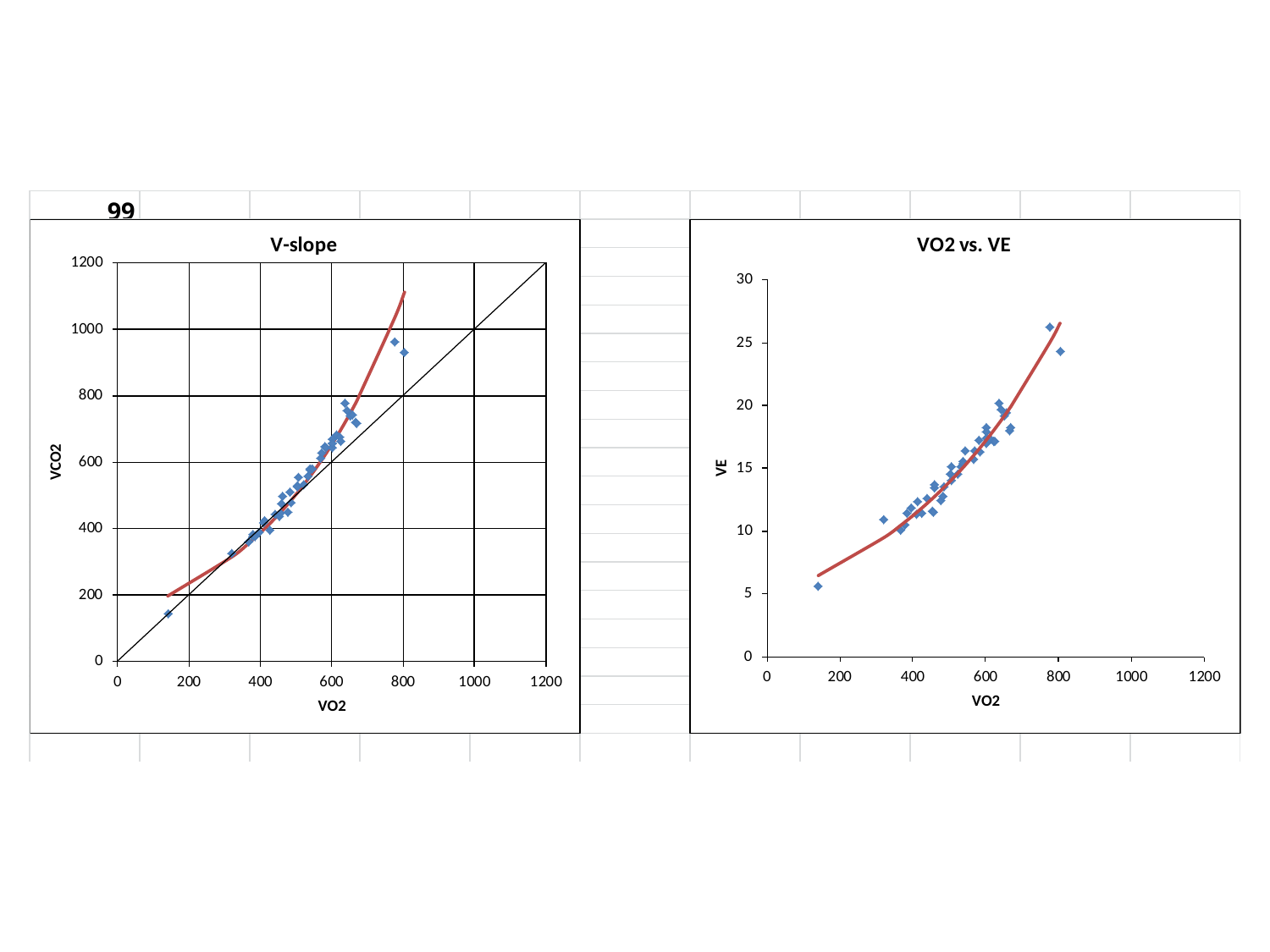

## Slide 101
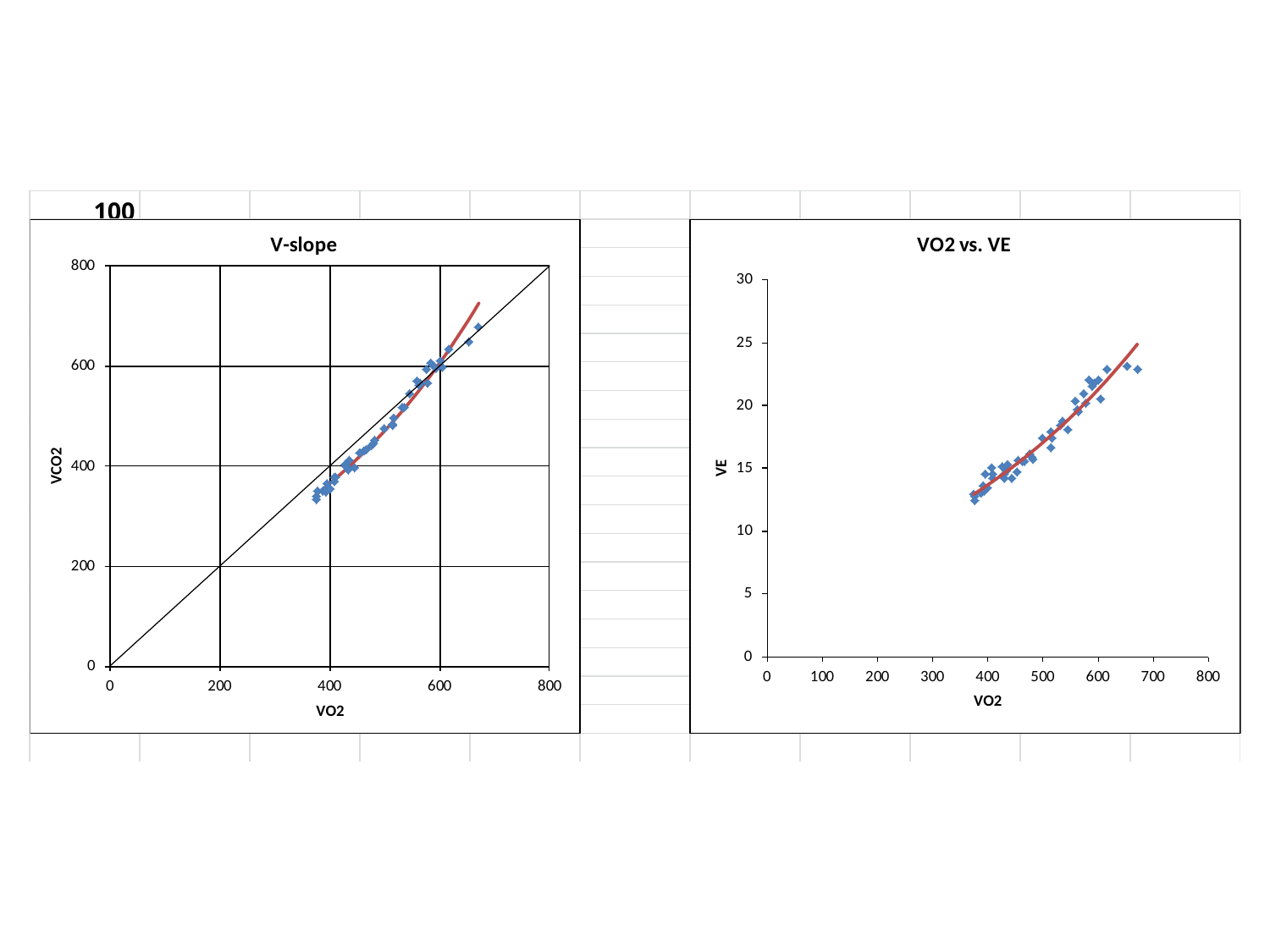

## Slide 102
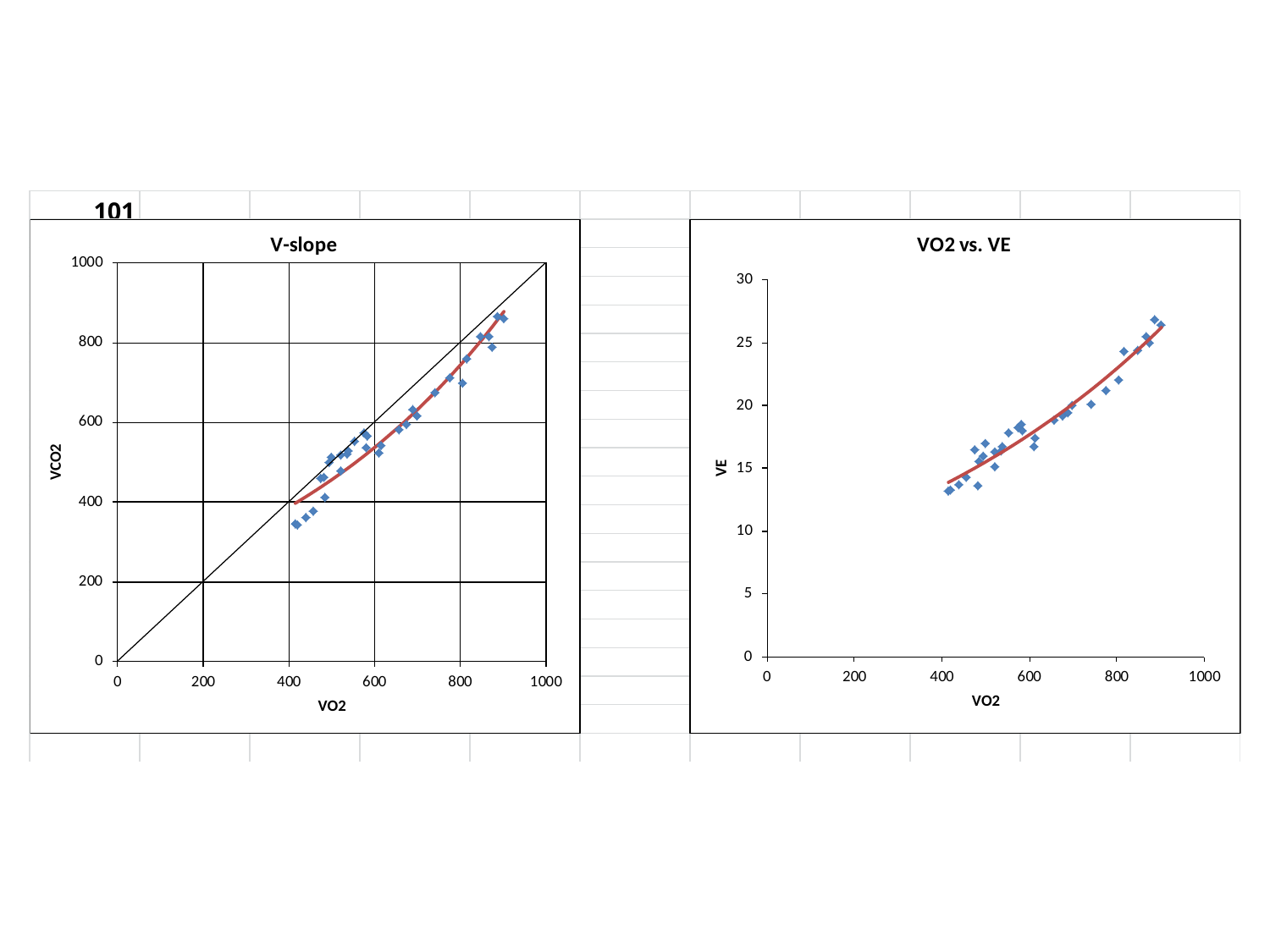

## Slide 103
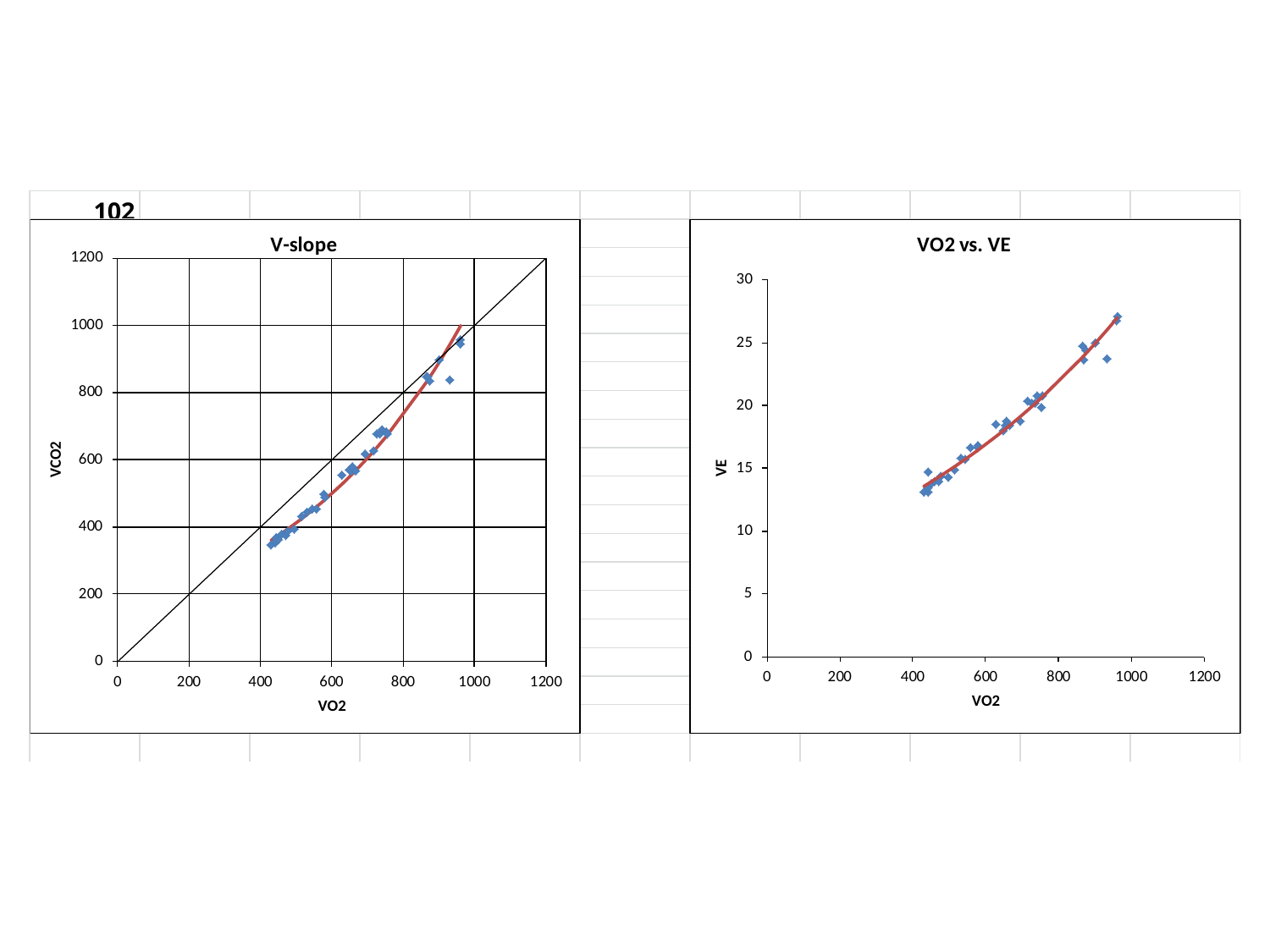

## Slide 104
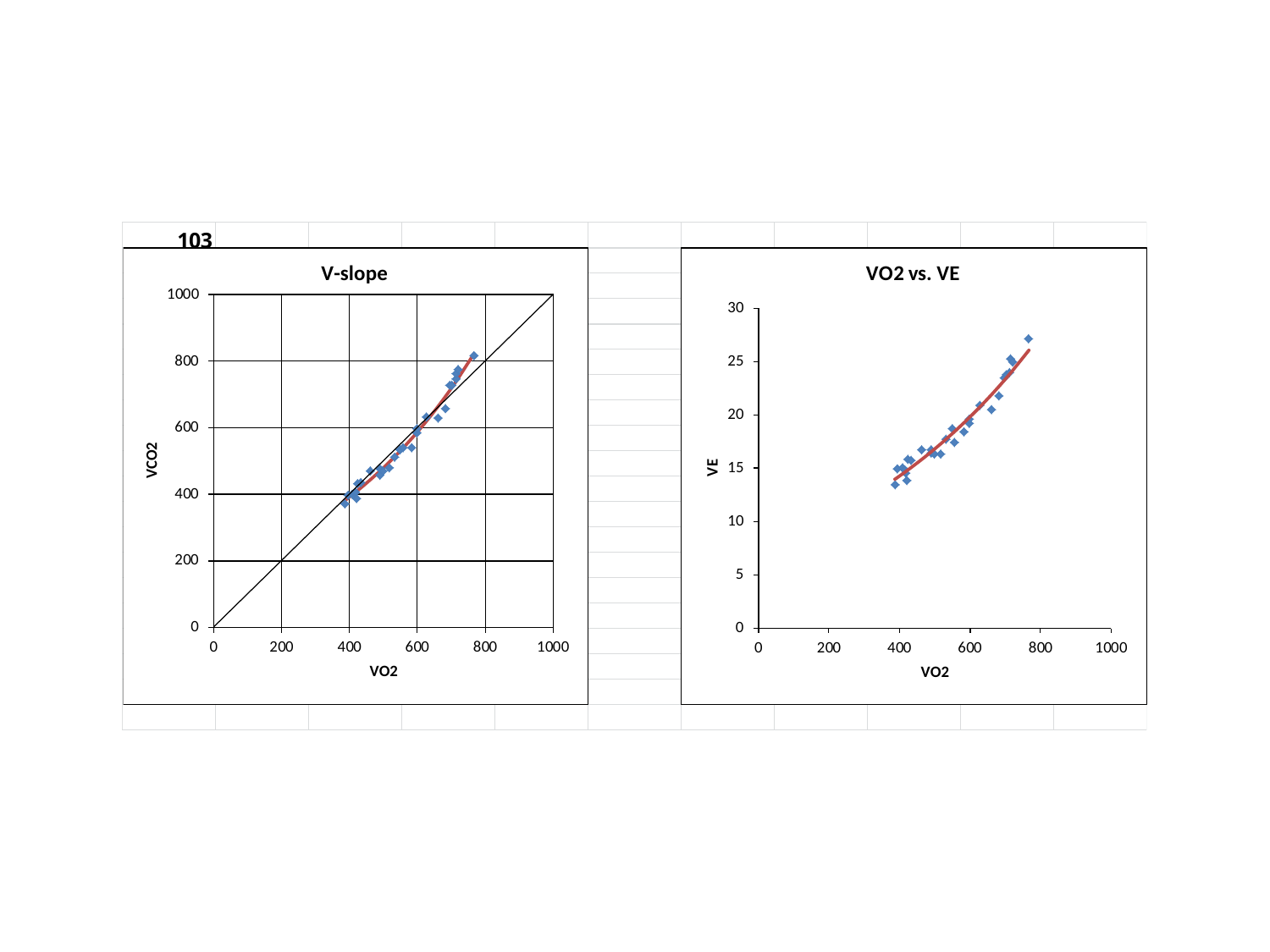

## Slide 105
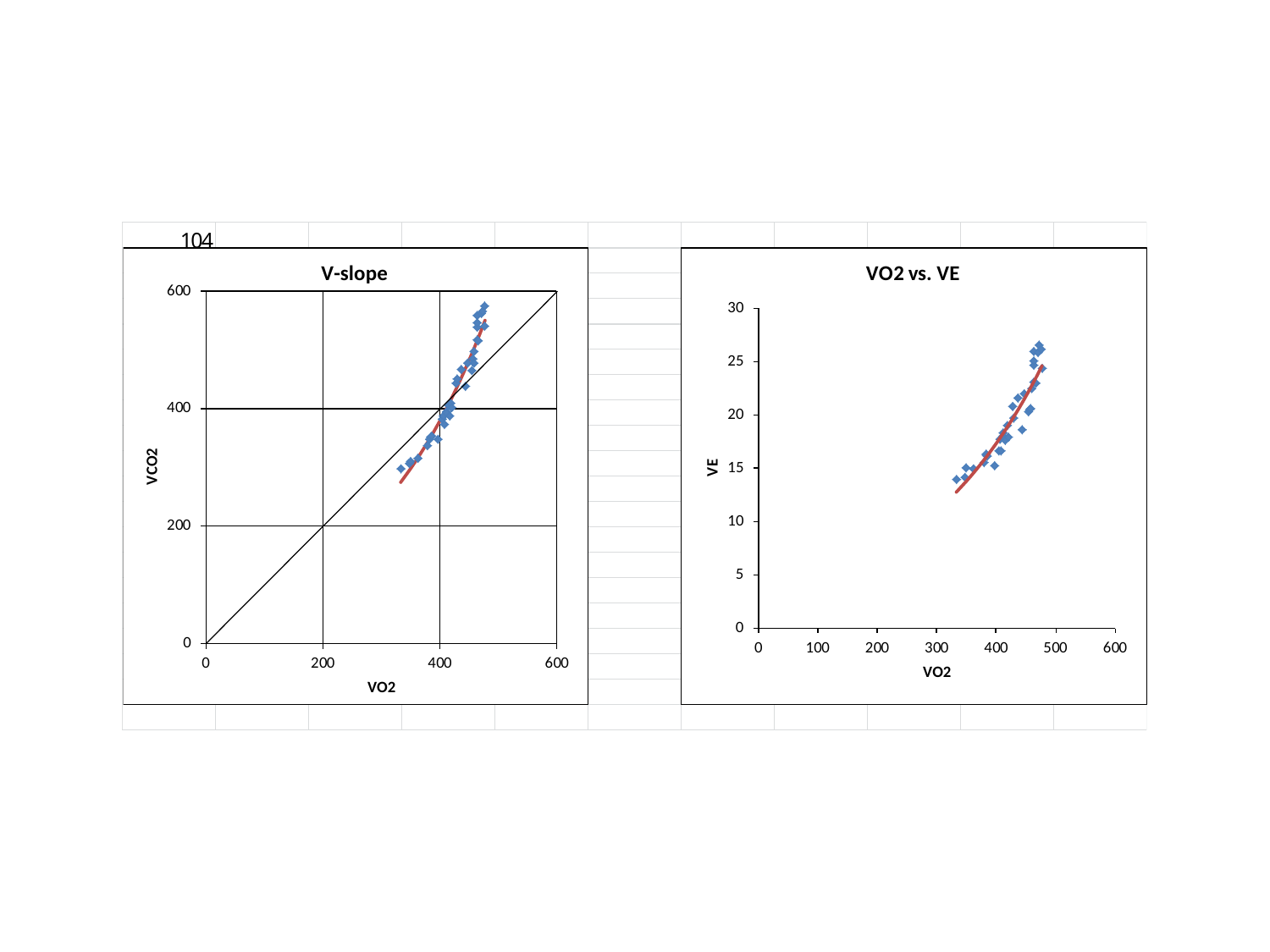

## Slide 106
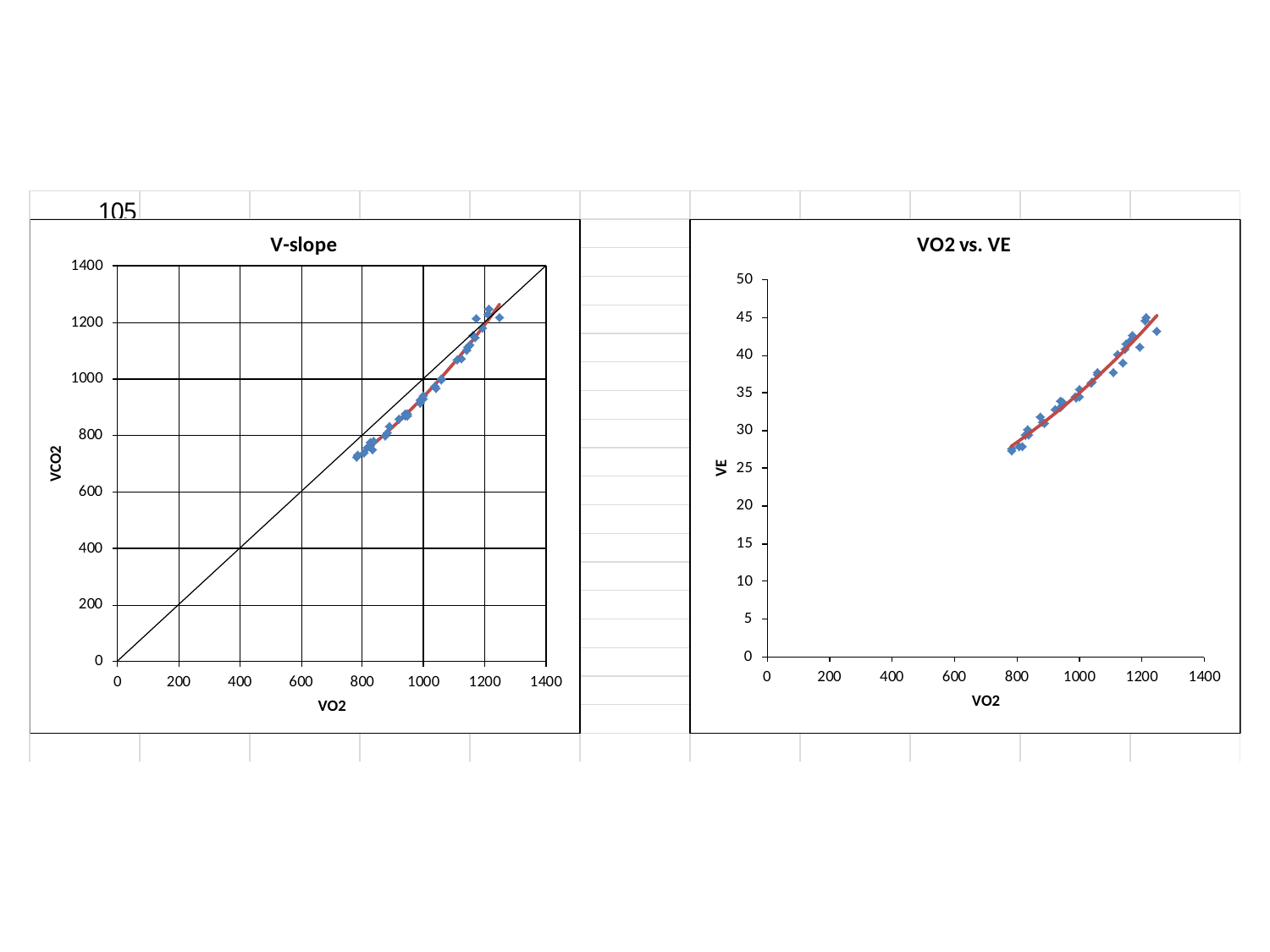

## Slide 107
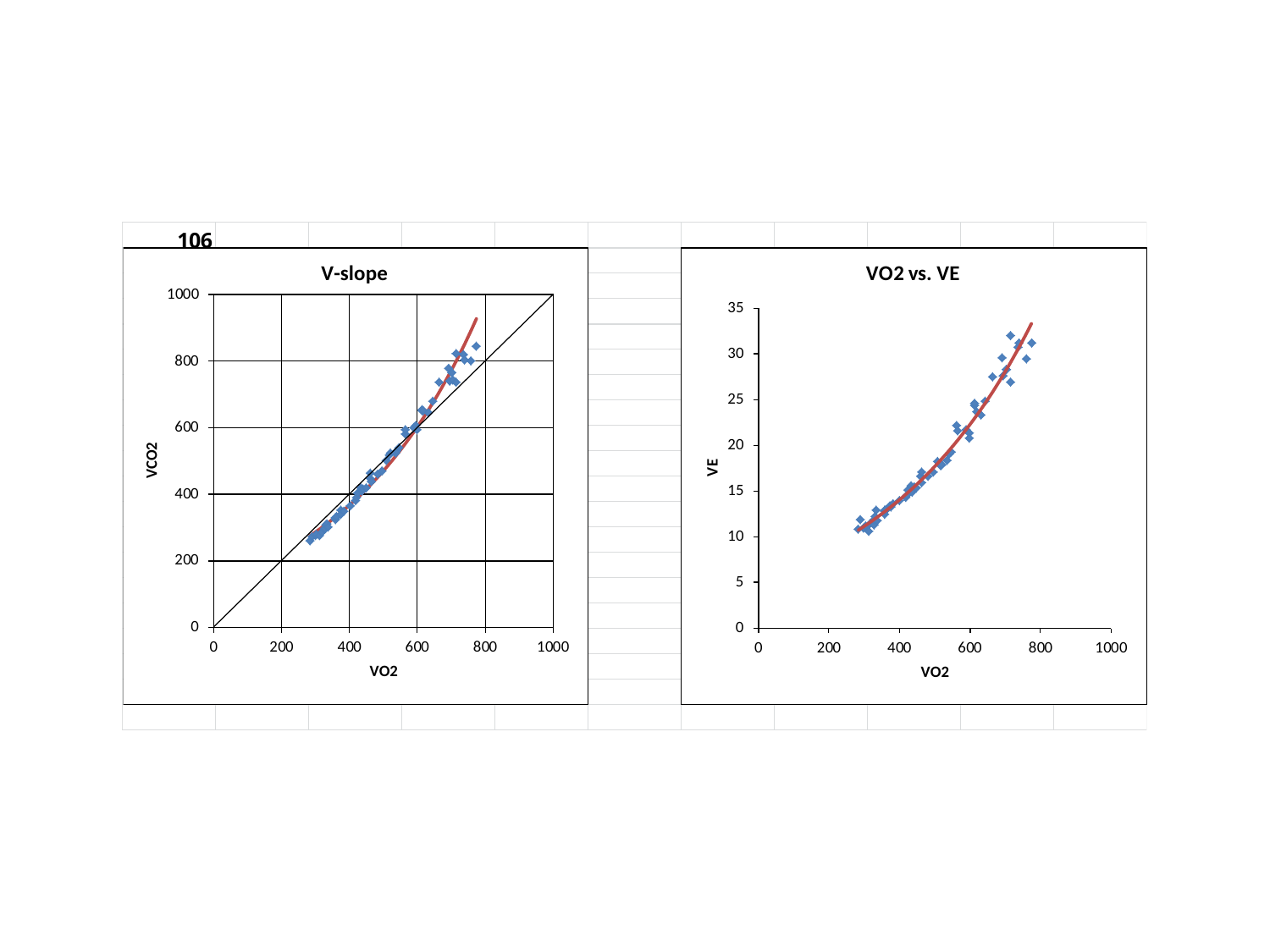

## Slide 108
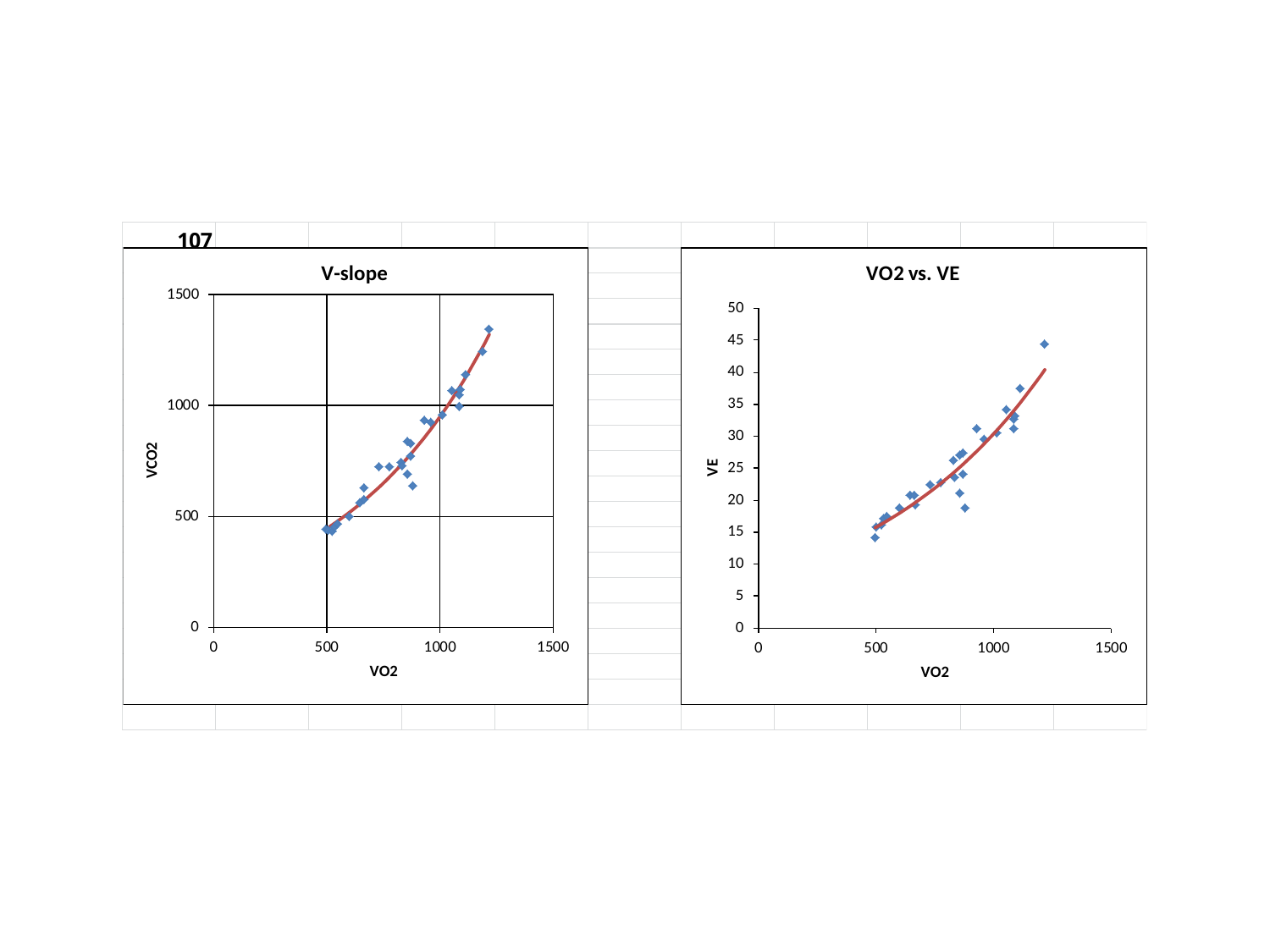

## Slide 109
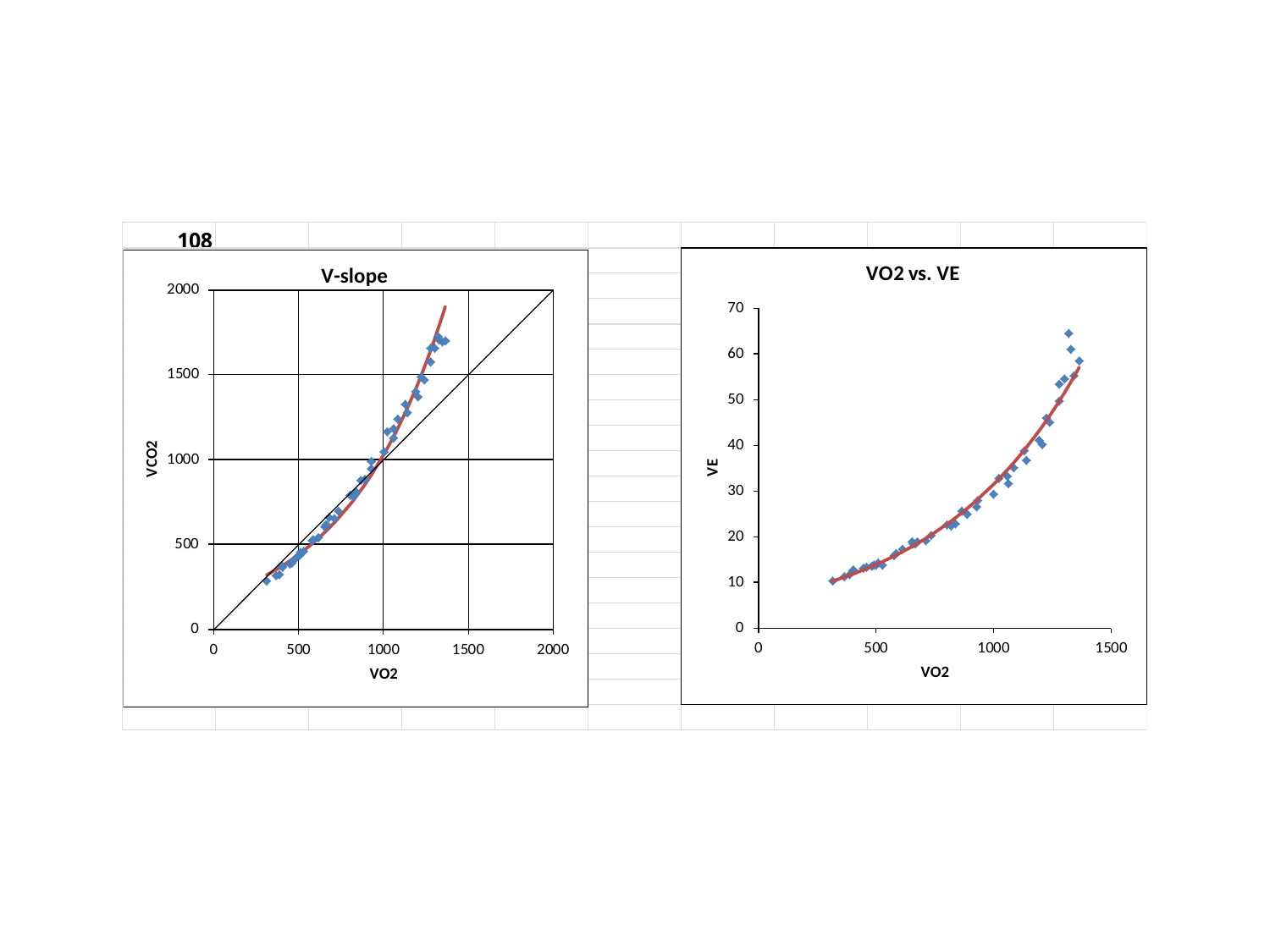

## Slide 110
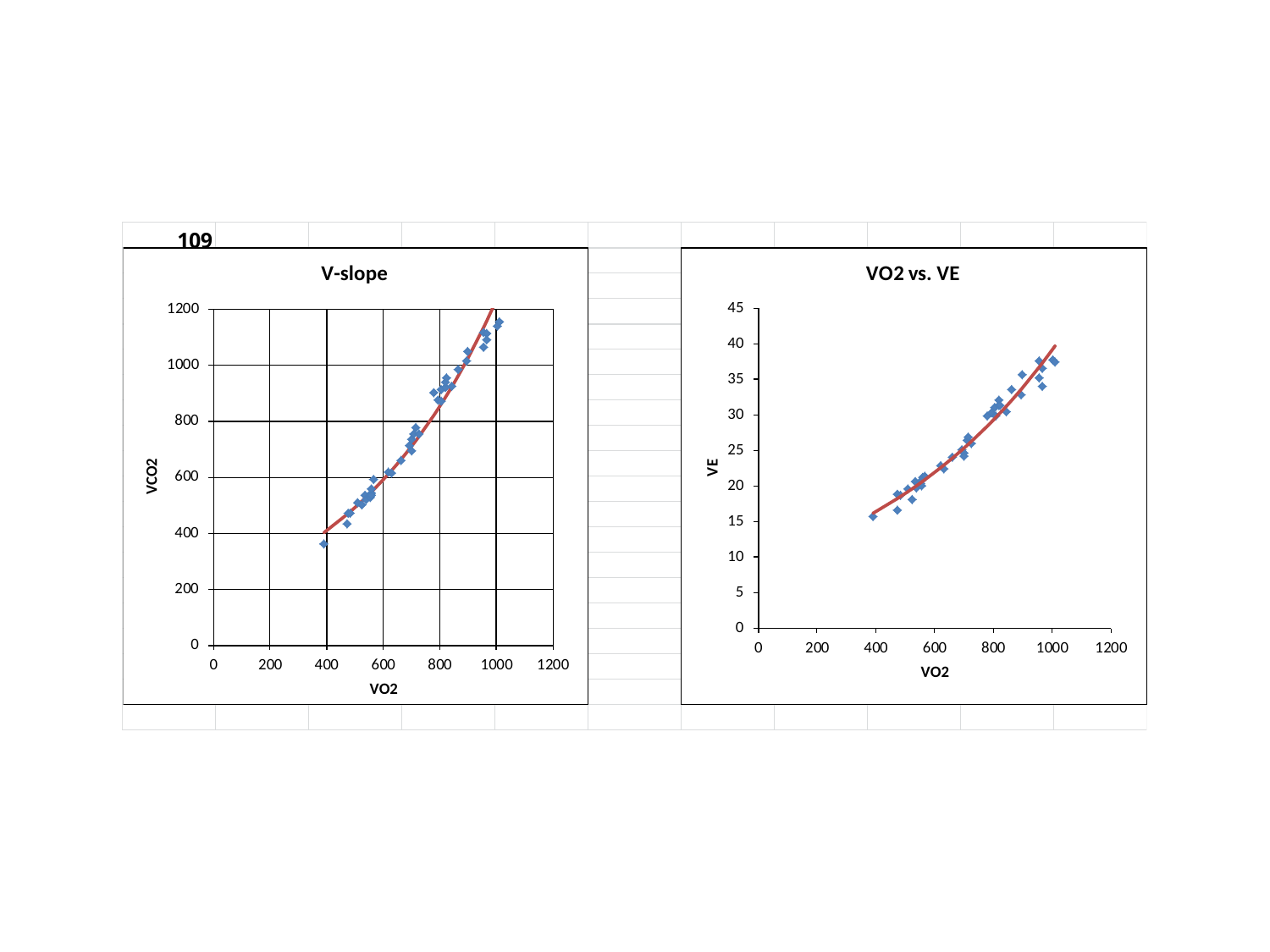

## Slide 111
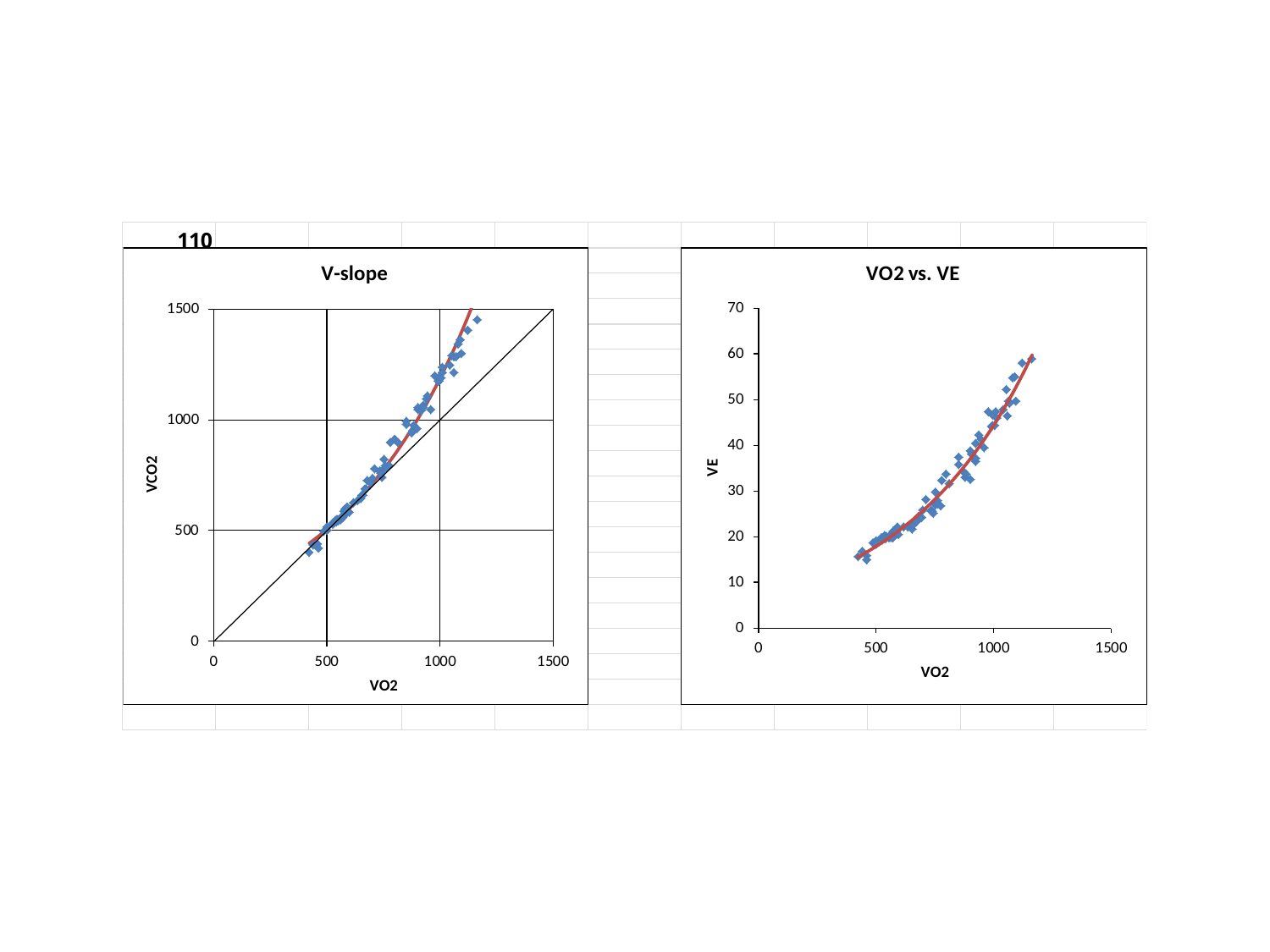

## Slide 112
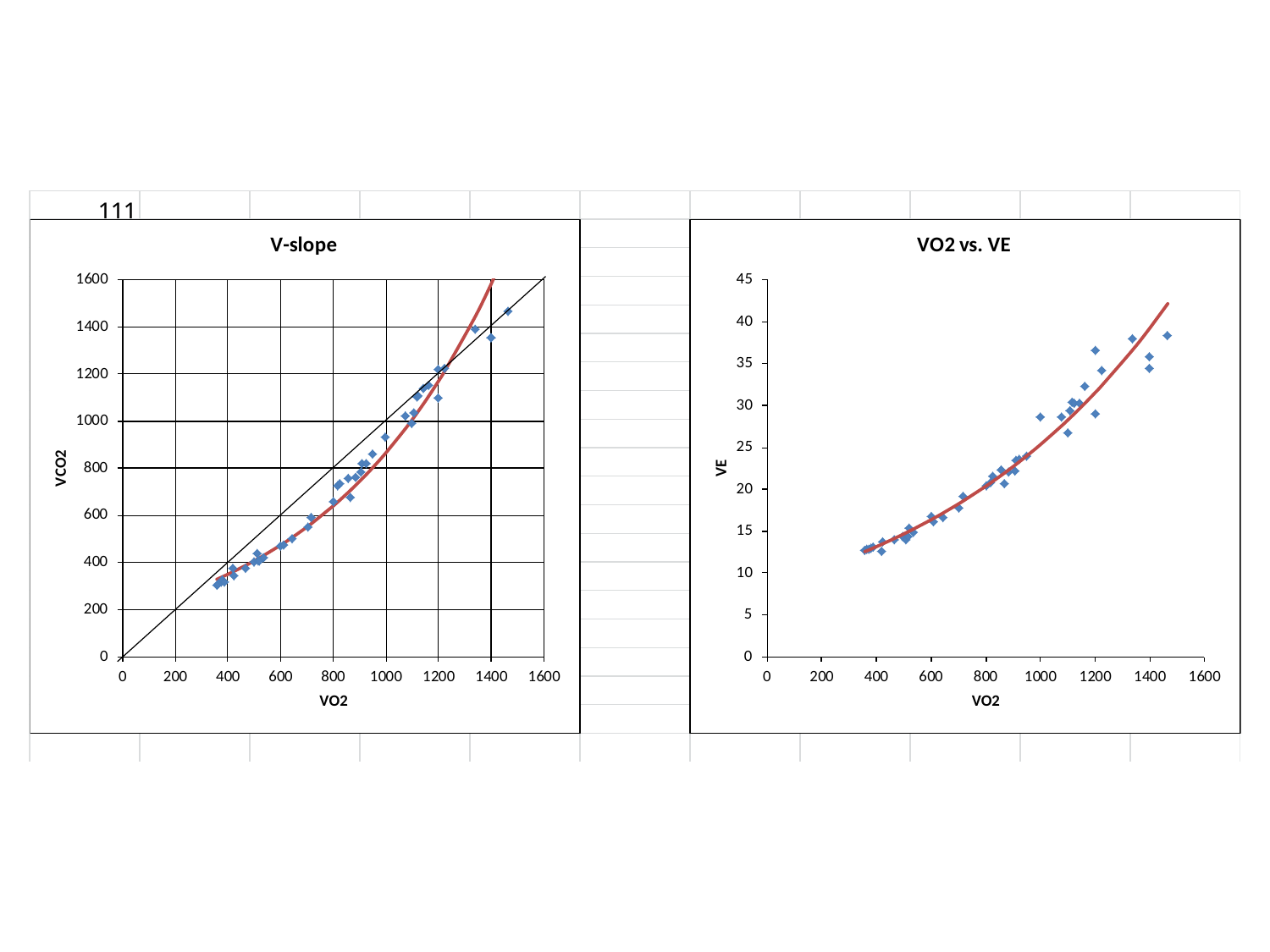

## Slide 113
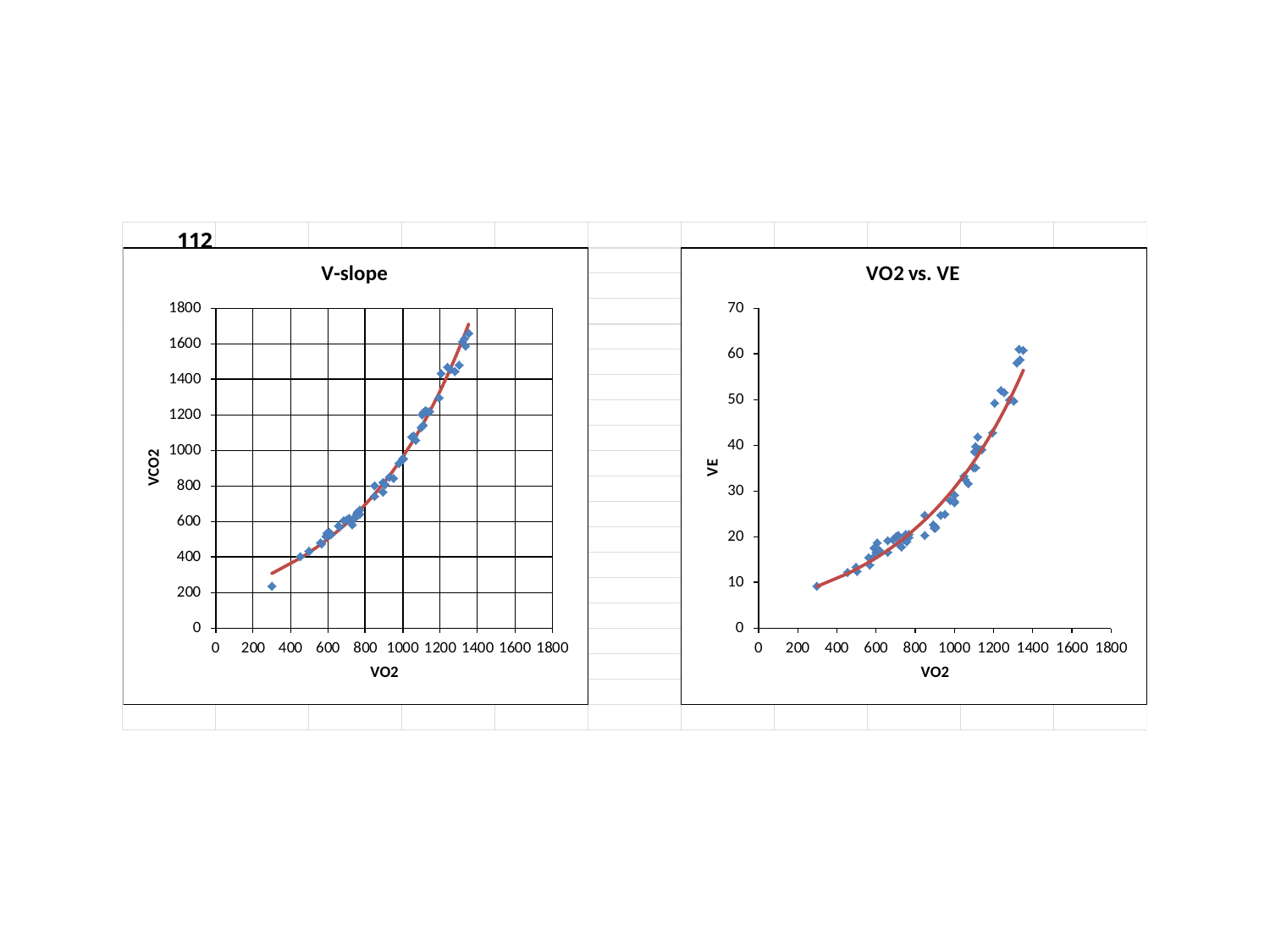

## Slide 114
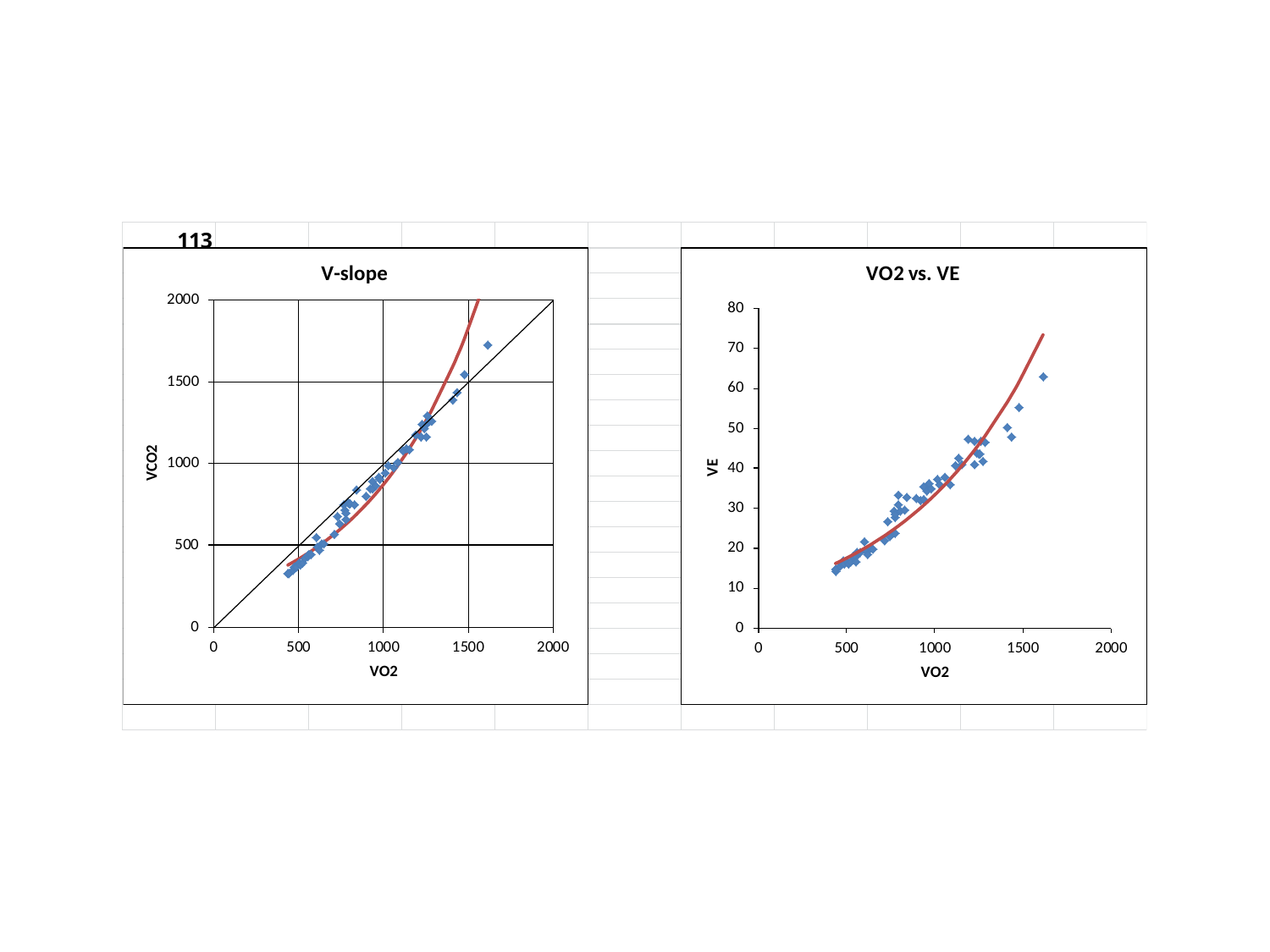

## Slide 115
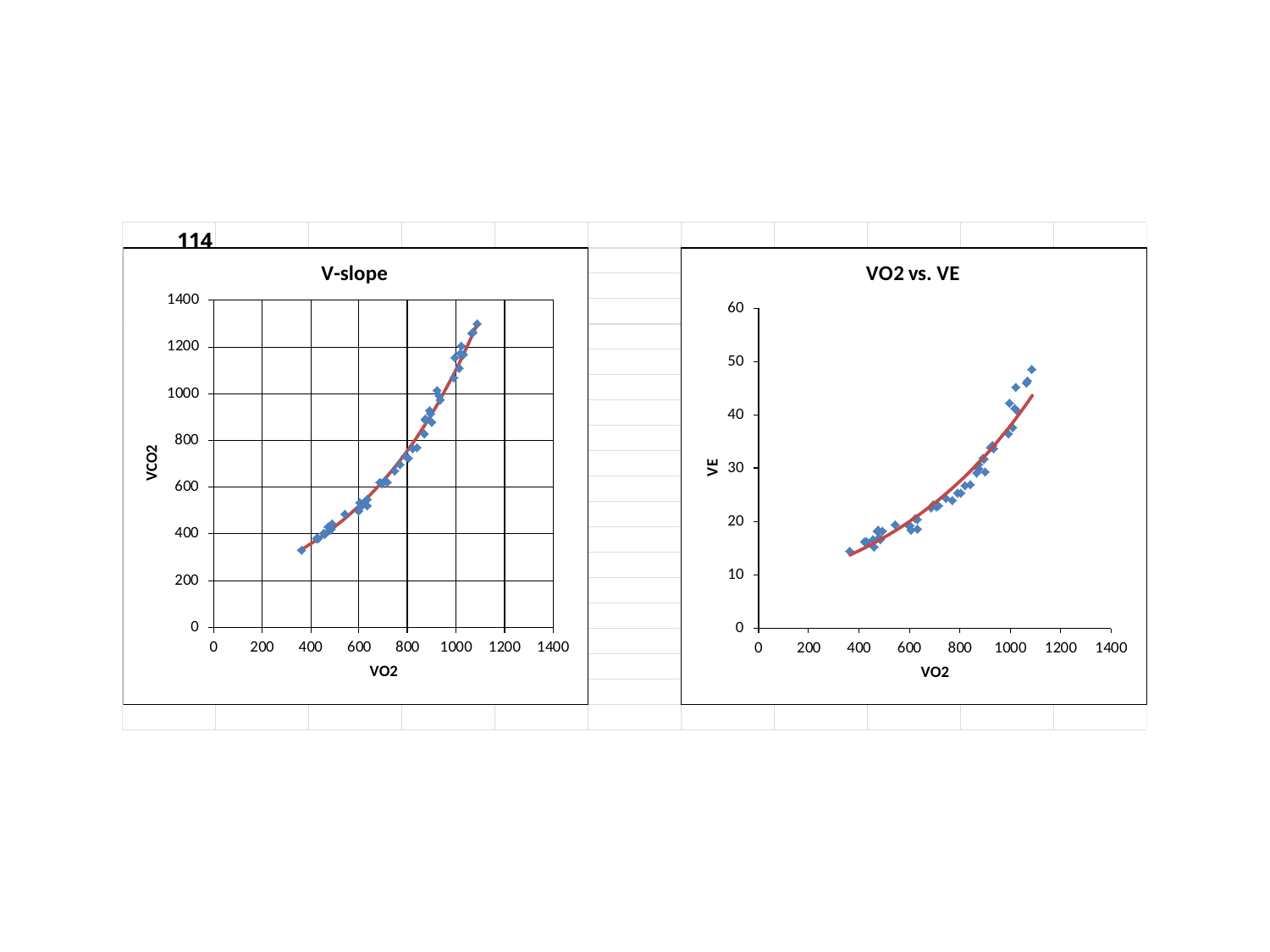

## Slide 116
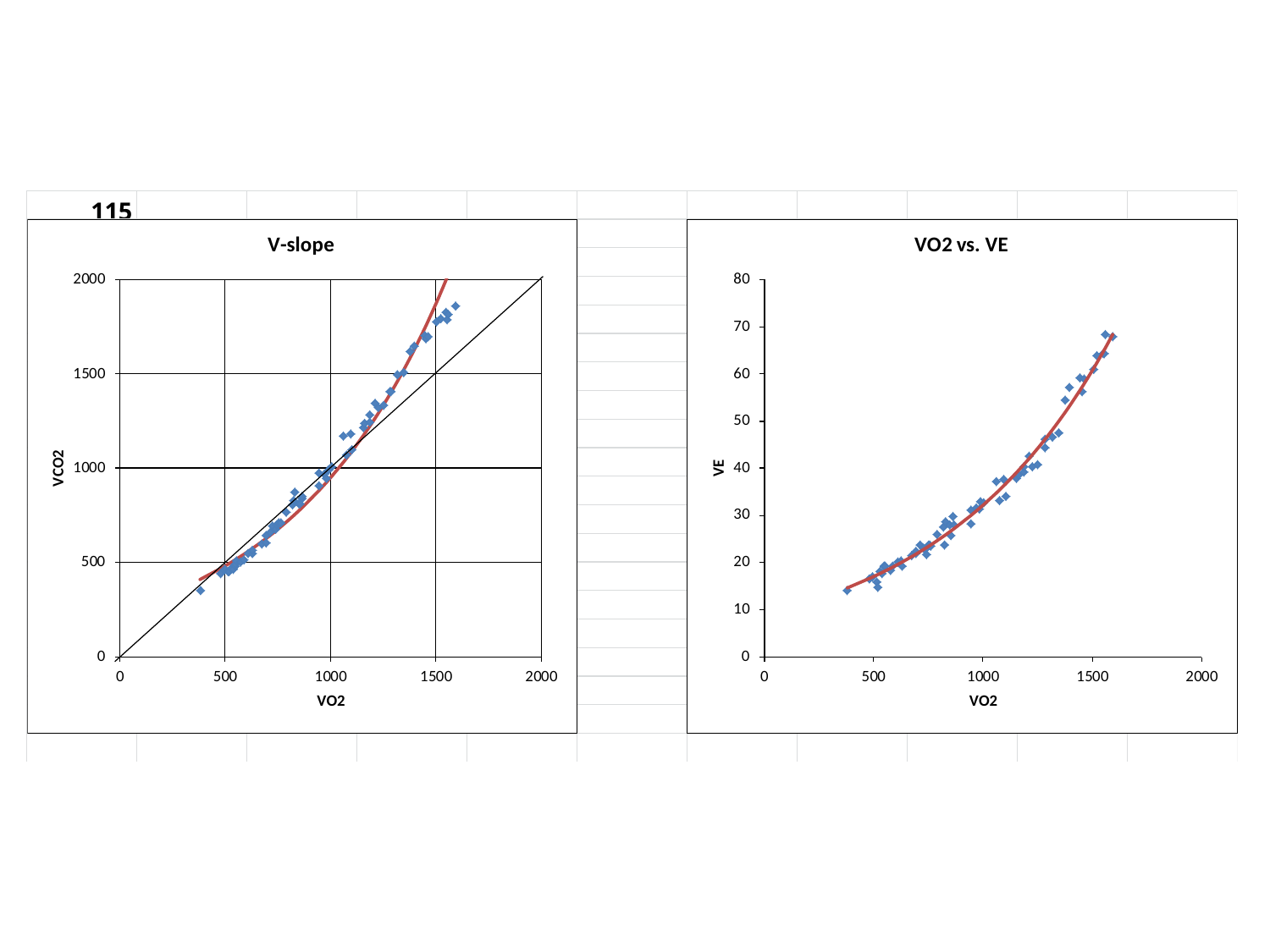

## Slide 117
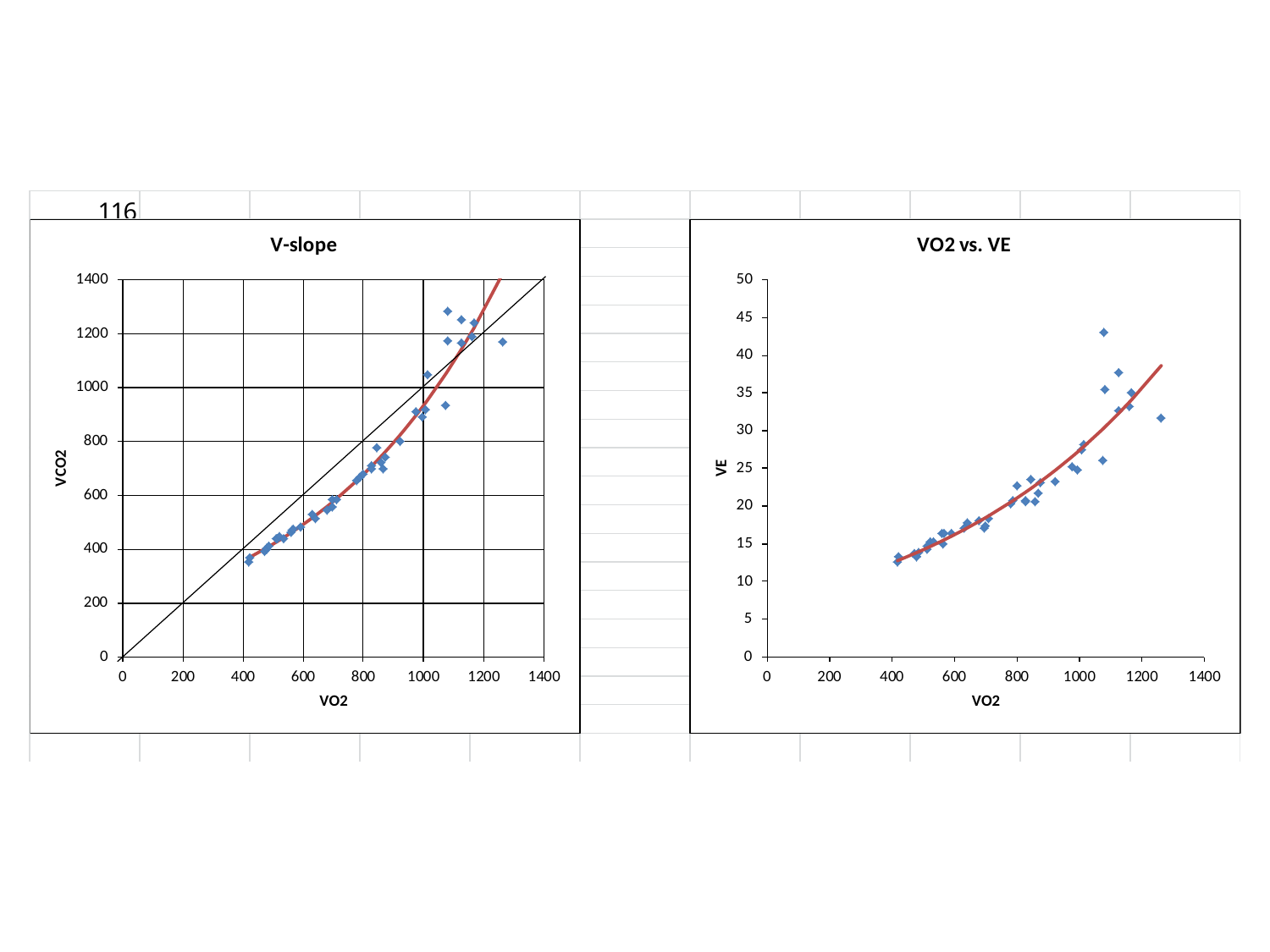

## Slide 118
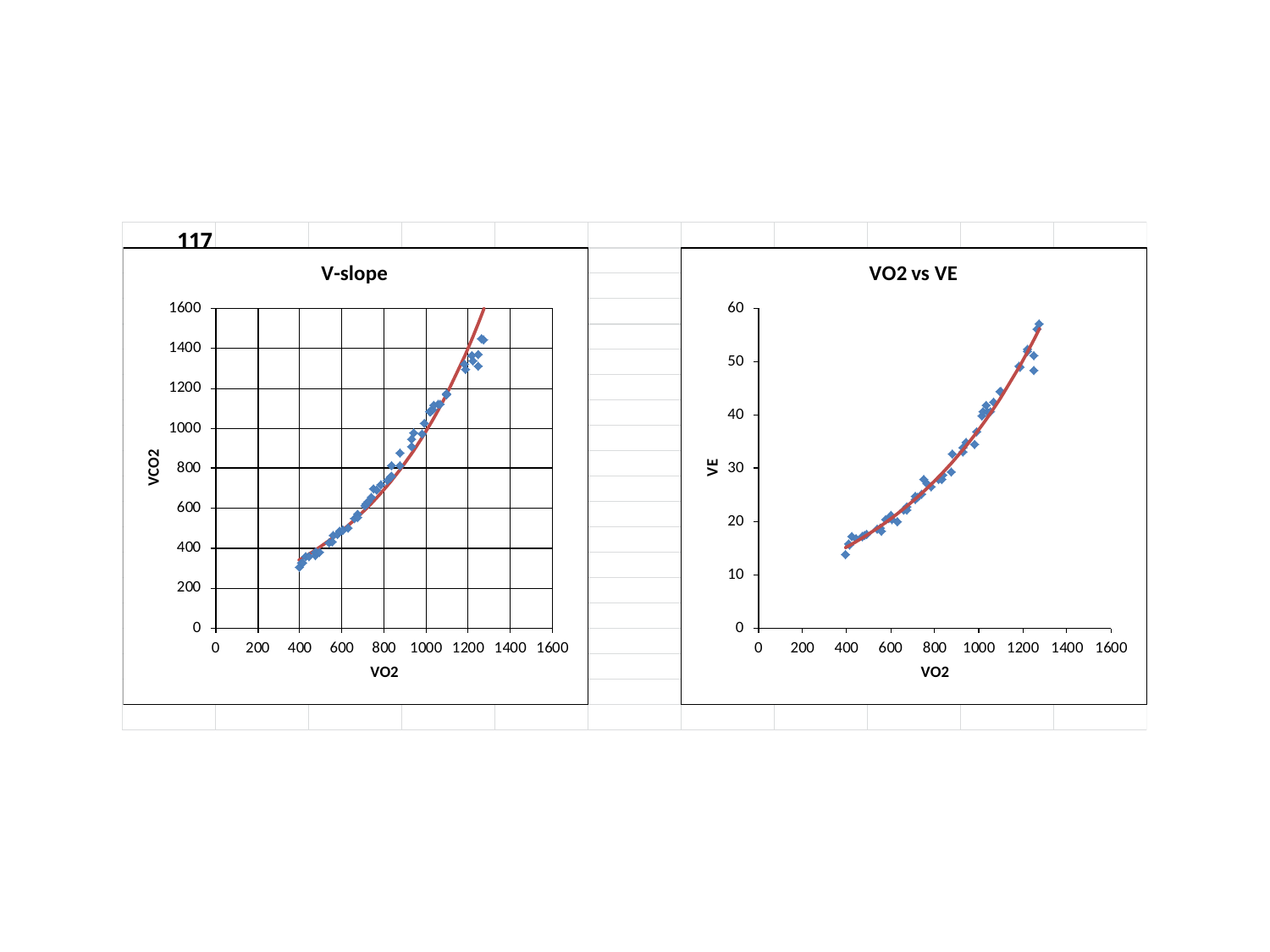

## Slide 119
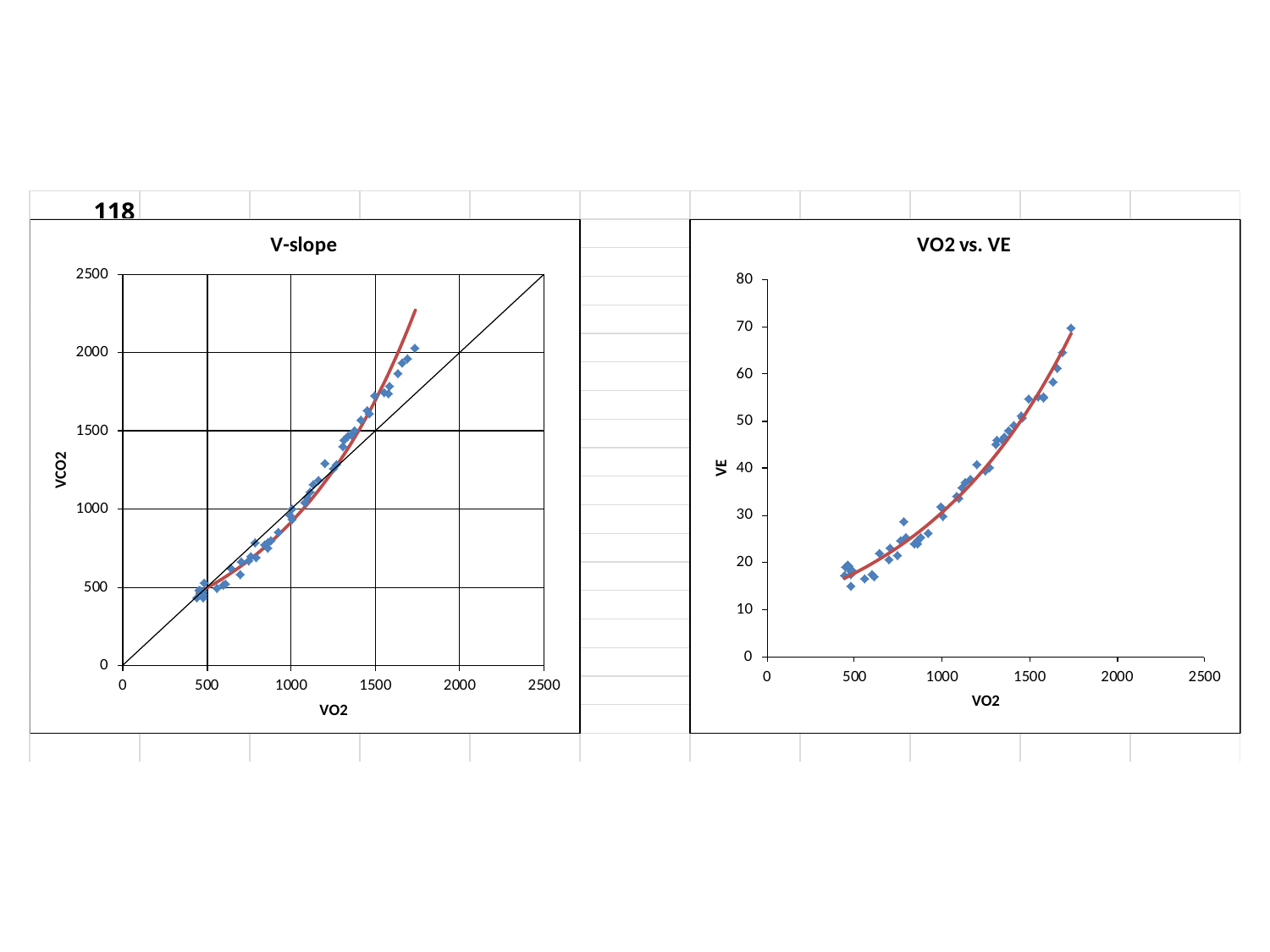

## Slide 120
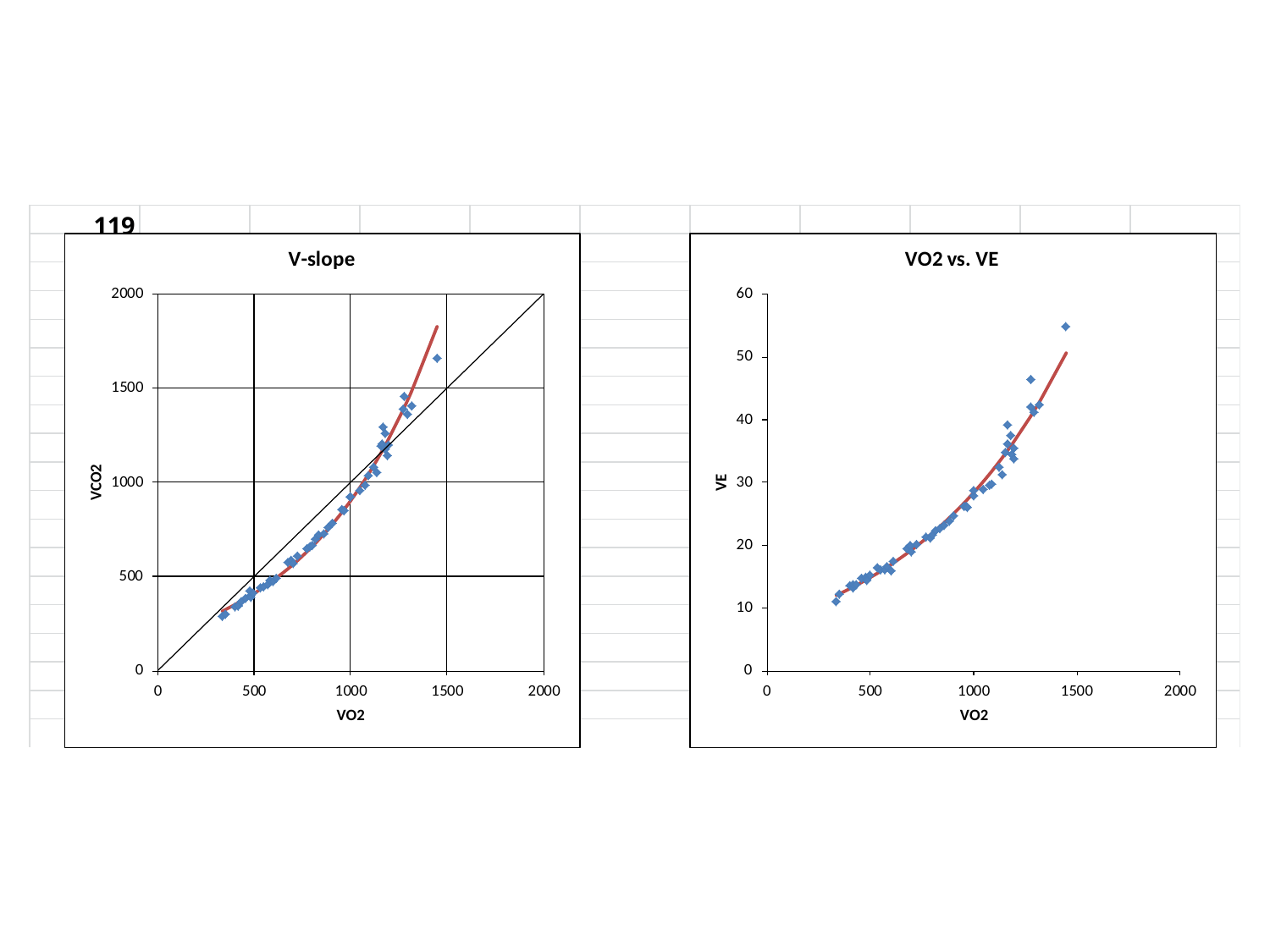

## Slide 121
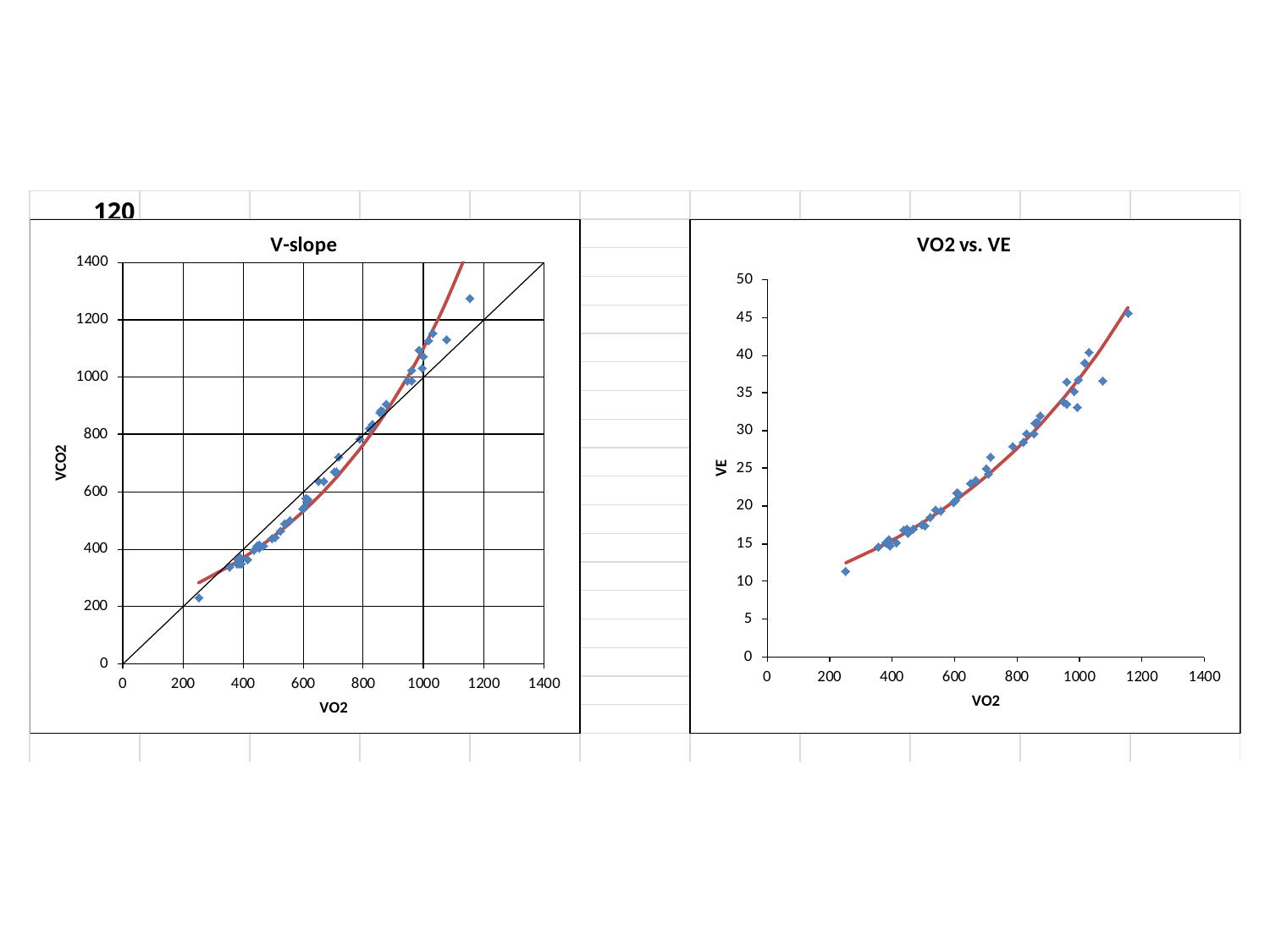

## Slide 122
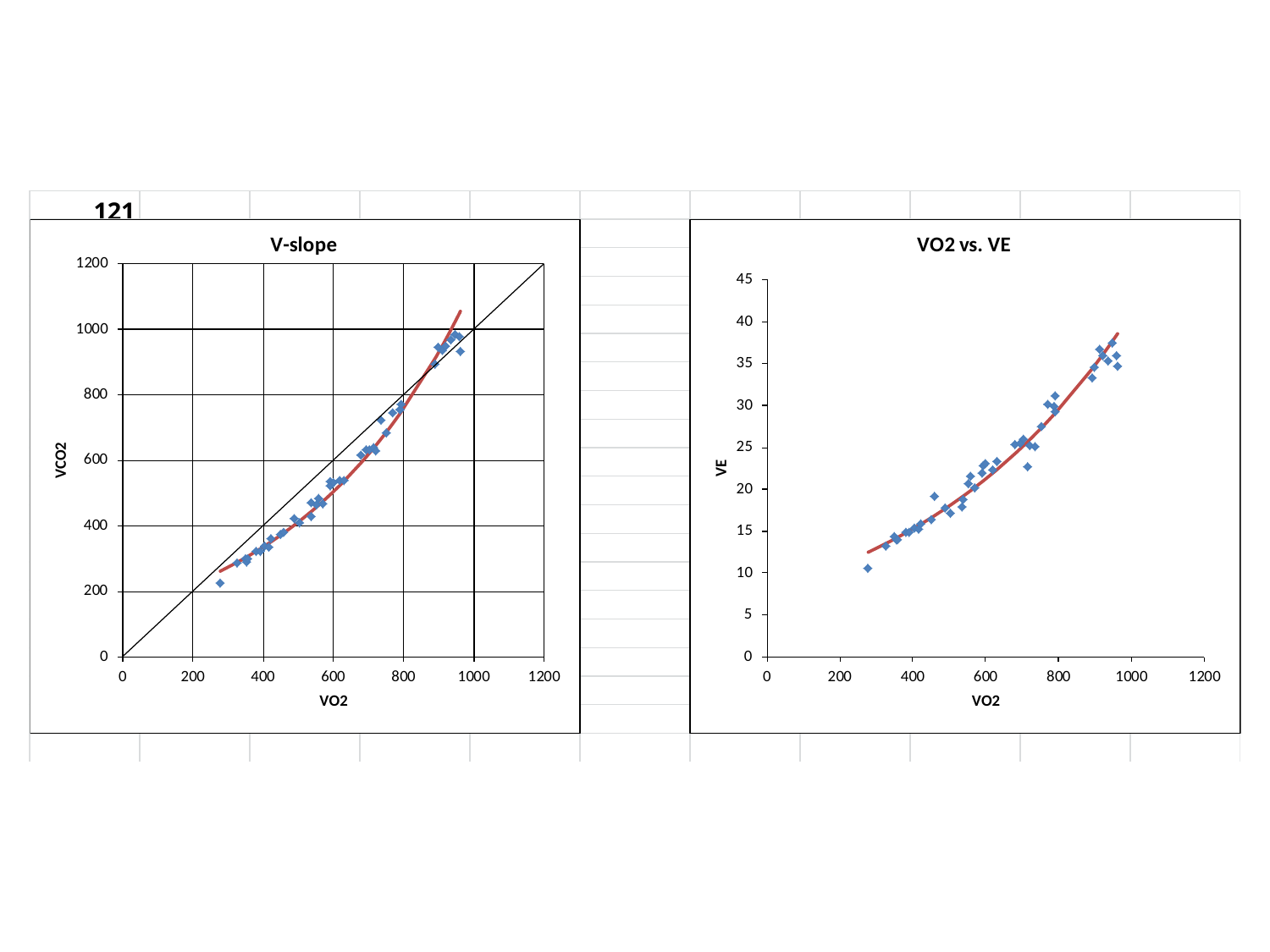

## Slide 123
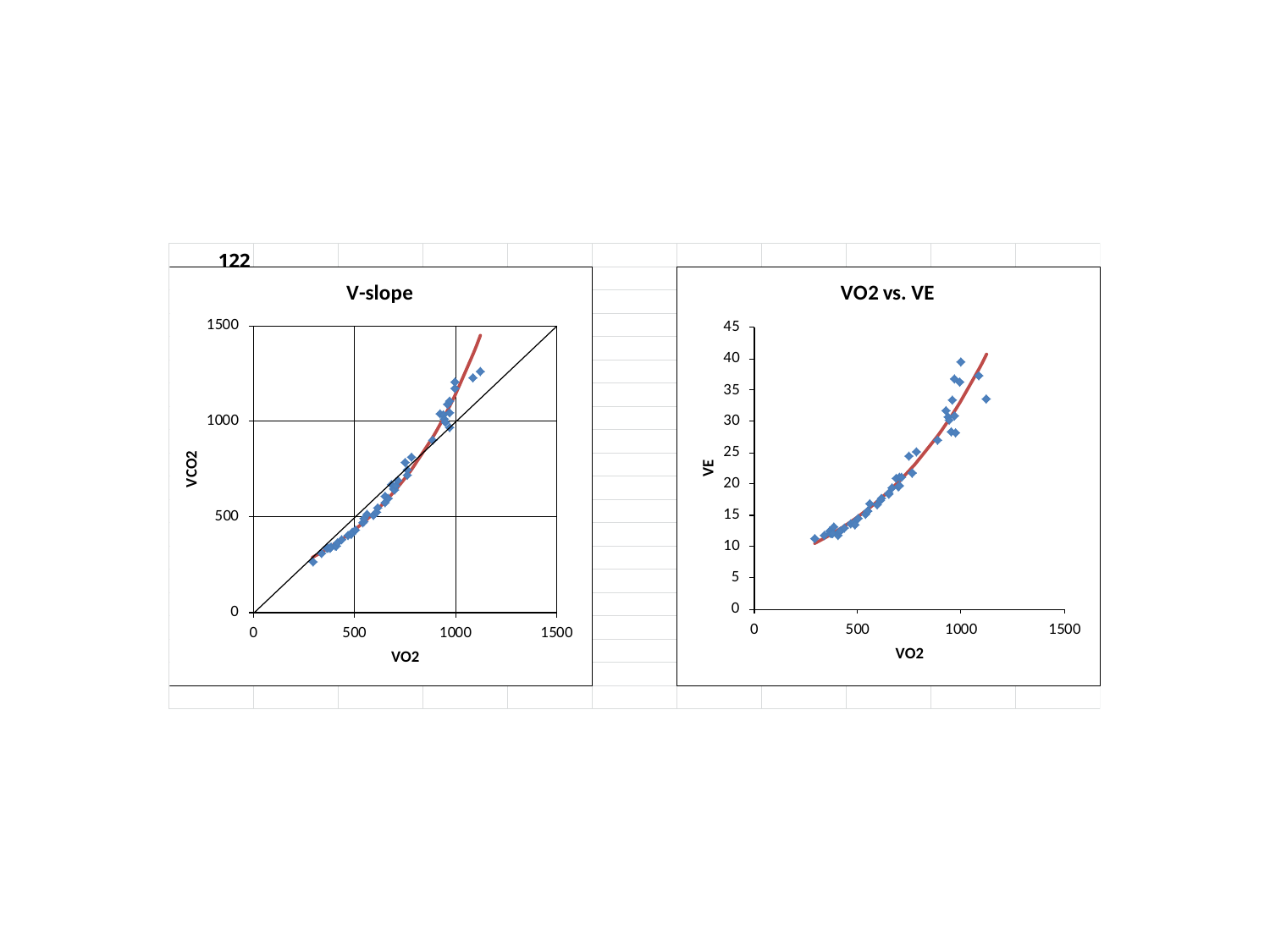

## Slide 124
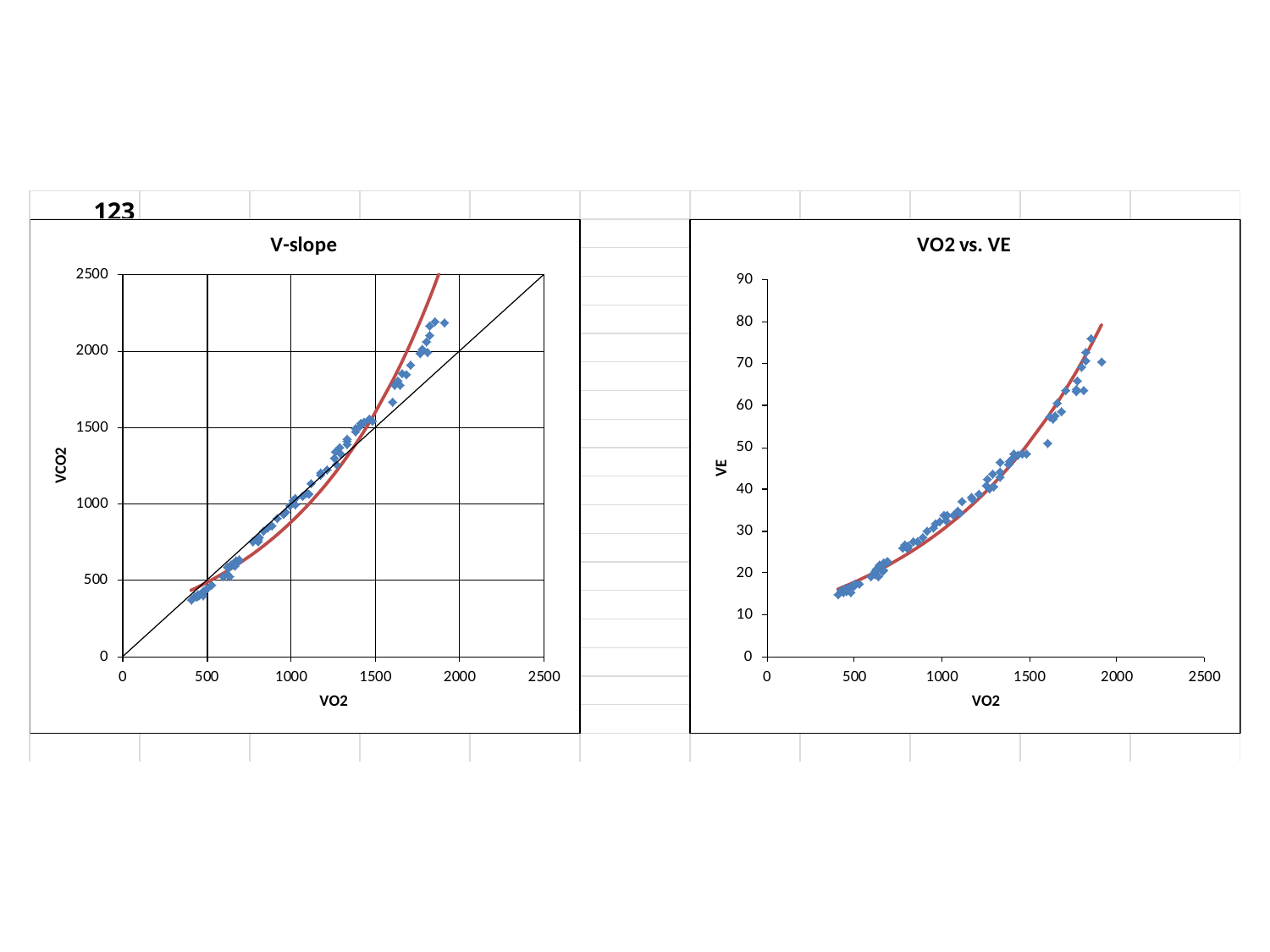

## Slide 125
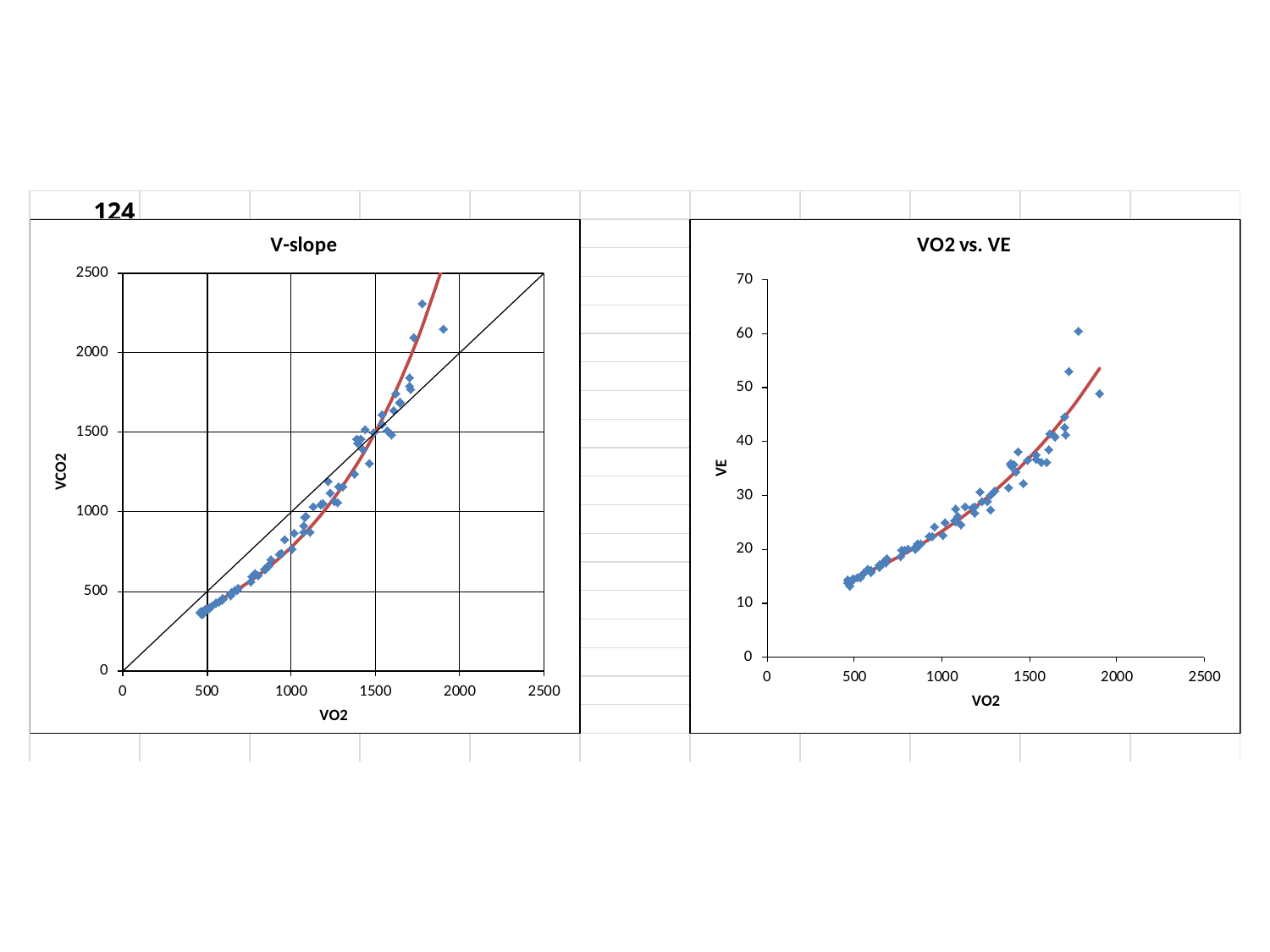

## Slide 126
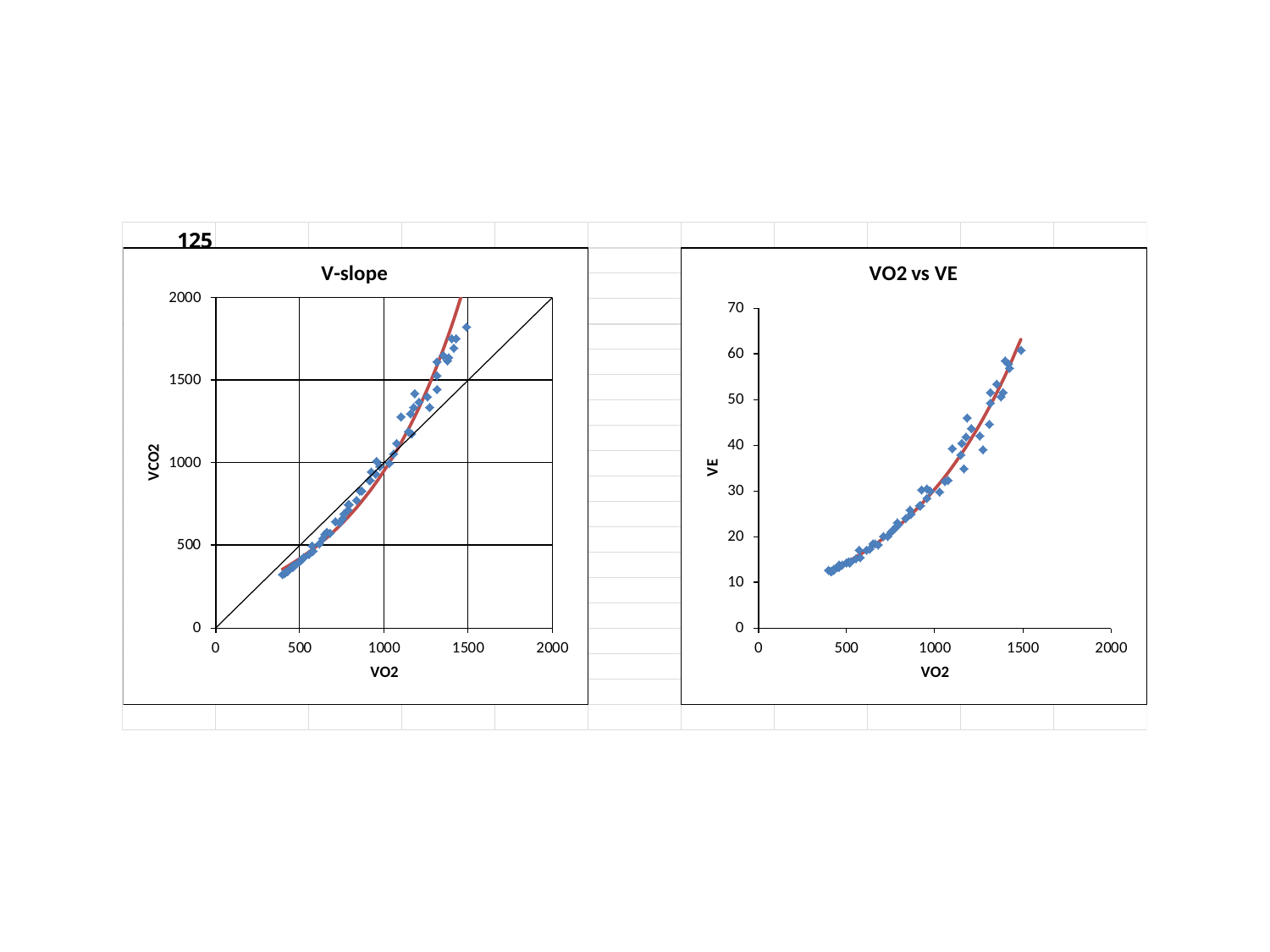

## Slide 127
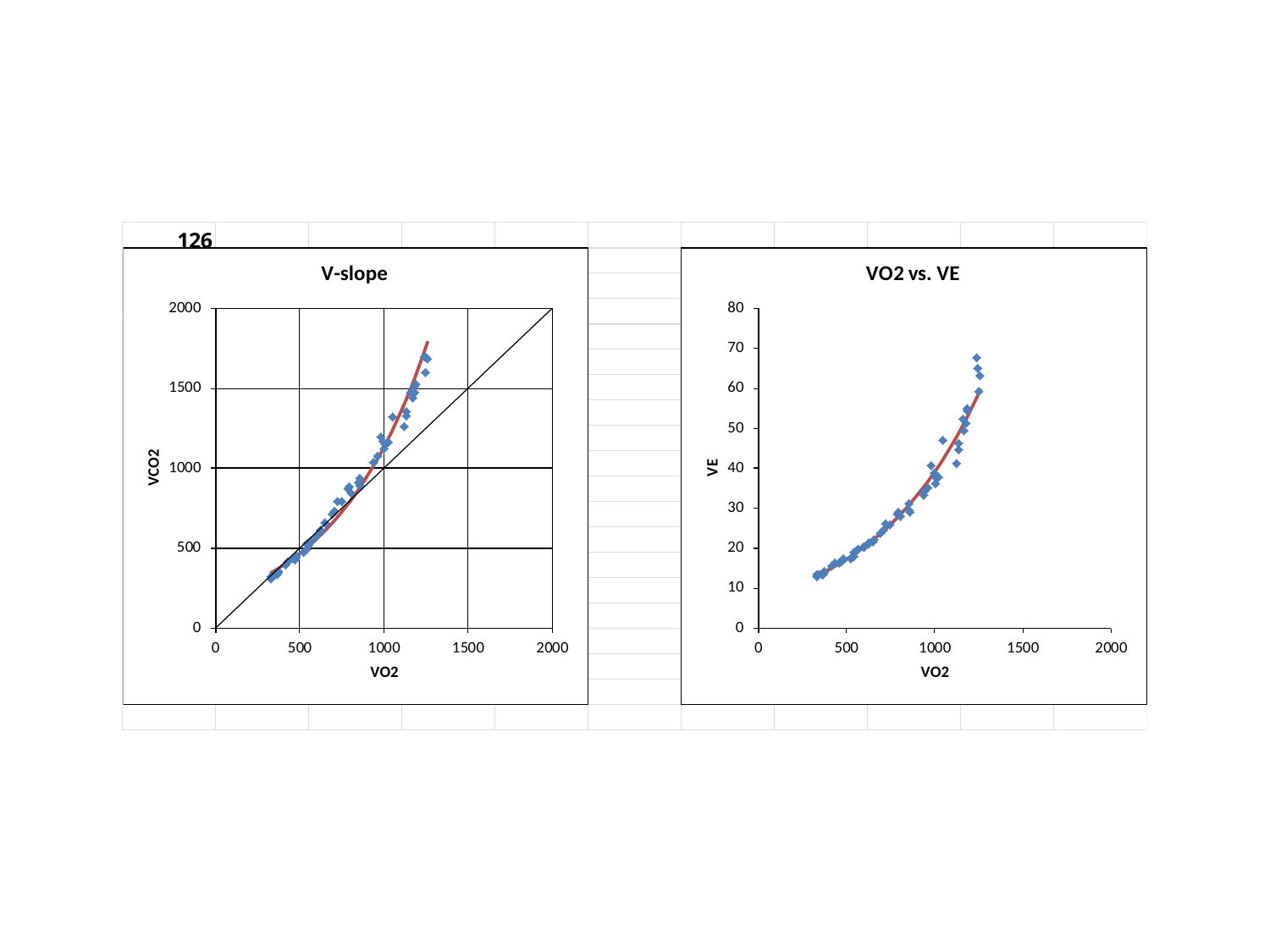

## Slide 128
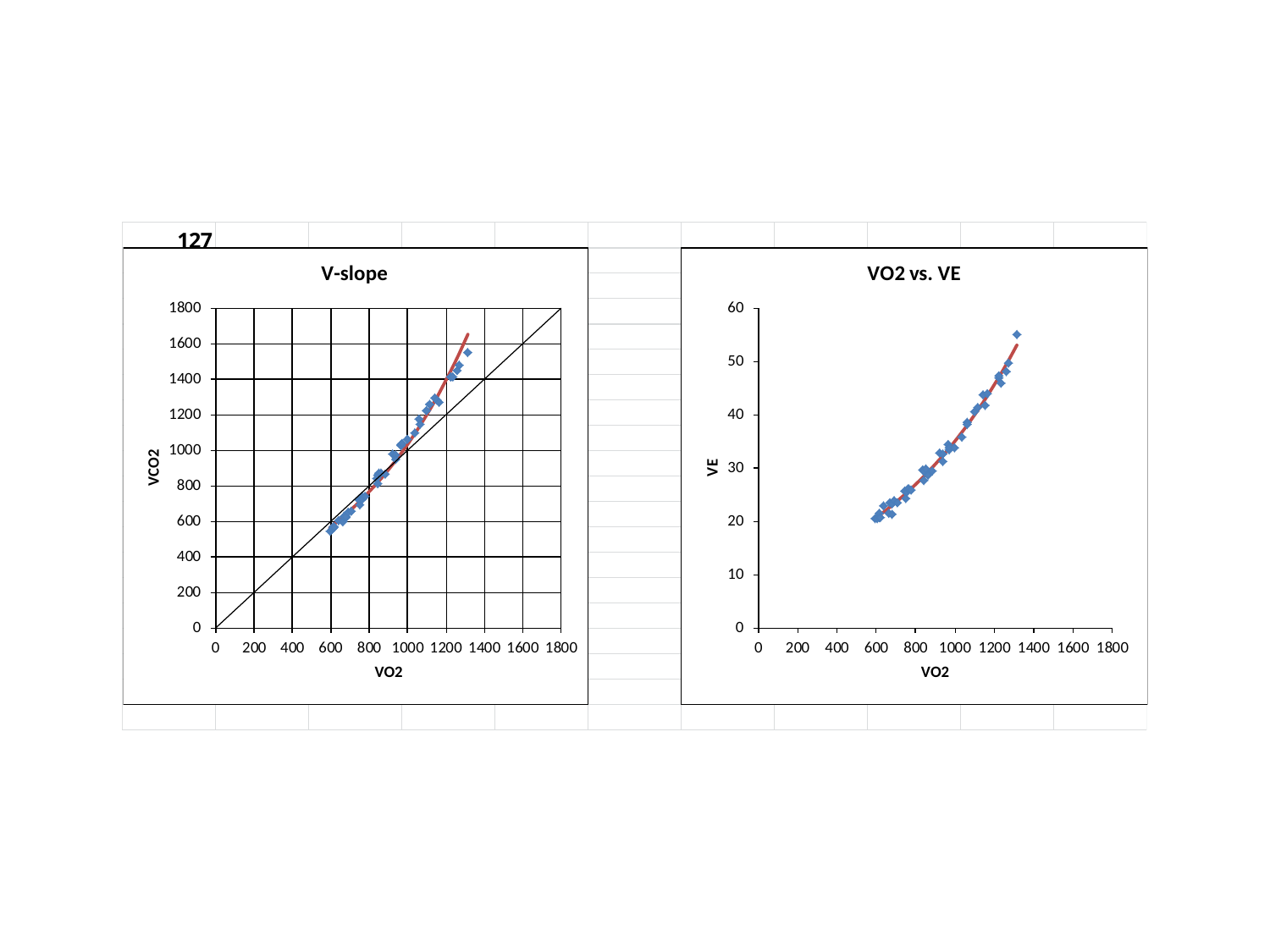

## Slide 129
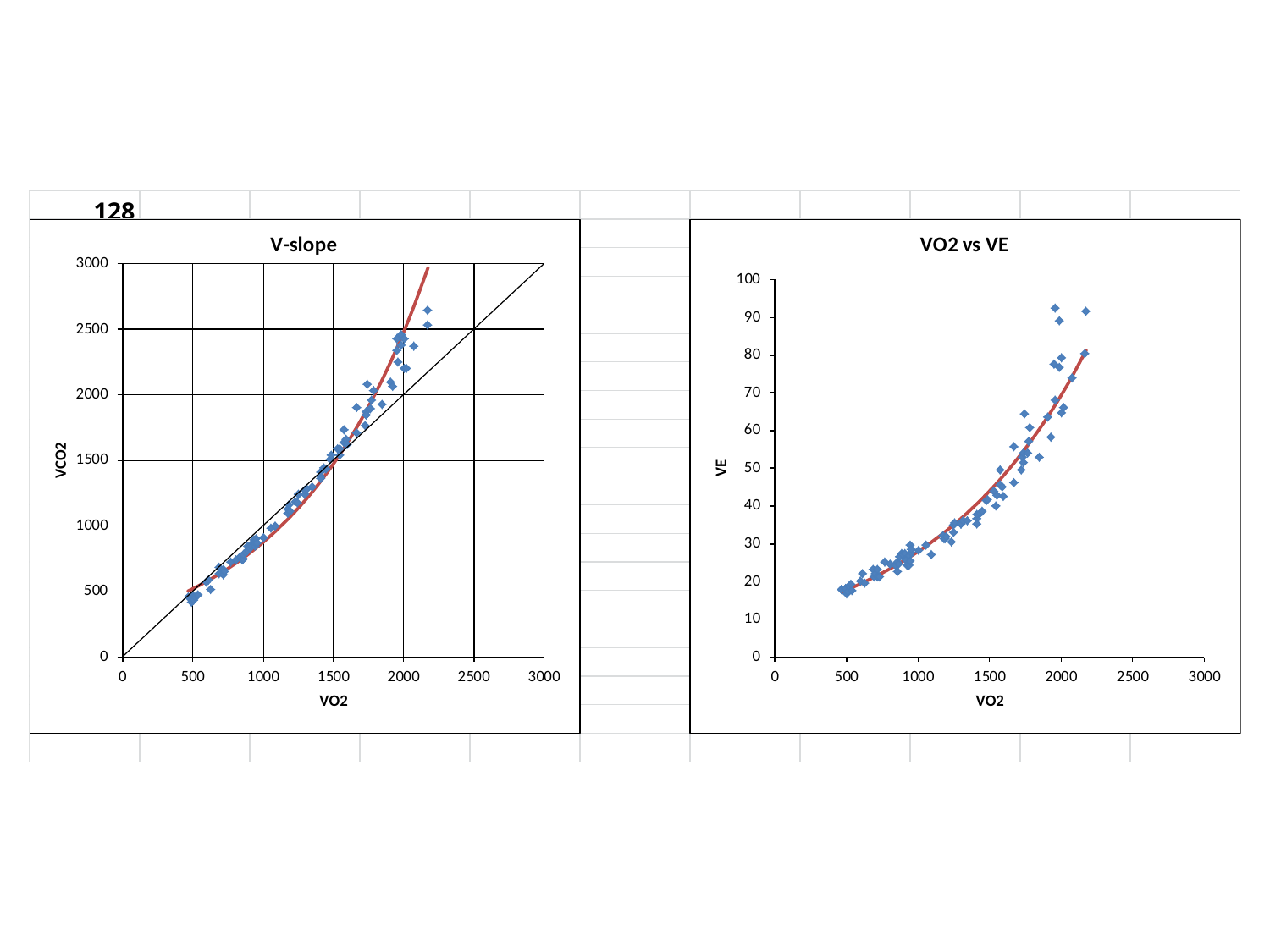

Supplement: Supplementary file 2 — Exponential fitting of all 128 cases. (PPTX 2984 kb) [file 13102_2019_122_MOESM2_ESM.pptx]
