# Supplementary material for: New method for the mathematical derivation of the ventilatory anaerobic threshold: a retrospective study
Source: BMC Sports Sci Med Rehabil. 2019 Jun 24;11:10. doi: 10.1186/s13102-019-0122-z (PMC6592010; doi:10.1186/s13102-019-0122-z)

**Additional file 3:**

**Supplementary Methods 1**

Derivation of computation formula: expVAT(VCO_2_)

The obtained exponential function is *y=ba^x^*_._

A tangent line is obtained by differentiation (based on the basic formula):

*y′=ba ^x^*LN(a)*, where LN(a) is the natural logarithm, *log_e_*.

As the slope must equal 1.0:

*1=ba^x^*LN(a)*,

*a^x^=1/[b*LN(a)].*

Taking the logarithm of both sides:

*LN(a^x^)=LN(1/[(b*LN(a)]),*

*xLN(a)=LN(1/[(b*LN(a)]),*

*x=LN(1/[(b*LN(a)]/LN(a))*.

Derivation of computation formula: expVAT(VE)

The obtained exponential equation is *y=ba^x^* (1).

A tangent line is obtained by differentiation (based on the basic formula):

*y′=ba^x^*LN(a^x^)*, where LN is *log_e_* (2).

Let a contact point *x_1_, f(x_1_)*.

The equation for the line passing through this point is as follows:

*y-f(x_1_)=f′(x_1_)*(x-x_1_)* (based on the formula).

This line must cross the origin (0, 0).

Therefore, *0-f(x_1_)=f′(x_1_)*(0-x_1_)*.

Dividing both sides with *-f(x_1_)*:

*1=[f′(x_1_)*(-x_1_)]/(-f(x_1_))*.

Replacing *f′(x1)* and *f(x1)* with (1) and (2):

*1=[ba^x^*LN(a)*(-x_1_)]/(-ba^x^); therefore, 1=LN(a)*x_1_,*

*x_1_=1/LN(a).*

As the accuracy of the exponential coefficient “a” is critical, an “a” value of at least 6 decimal places, such as 1.001567, is required.

**Supplementary Methods 2**

Detection of outliers on exponential fitting

For institution A data, no plot for VO_2_ vs. VCO_2_ and 4 plots for VO_2_ vs. VE (r: 0.825, -0.921) were excluded as outliers. For institution B data, for the VO_2_ vs. VCO_2_ plot, one plot each for pre- and post-rehabilitation (r: 0.919, 0.950), whereas for the VO_2_ vs. VE plot, 2 plots (r: 0.767, 0.849) for pre-rehabilitation and 3 plots (r: 0.470, 0.891) for post-rehabilitation, were removed from further analyses as outliers. The mean correlation coefficients (r) for institution A data were 0.988±0.007 and 0.987±0.010 for the VO_2_ vs. VCO_2_ relation and for the VO_2_ vs. VE relation, respectively. For institution B, the values were 0.988±0.009 and 0.971±0.022 (pre-rehabilitation) and 0.990±0.006 and 0.979±0.017 (post-rehabilitation) for the VO_2_ vs. VCO_2_ and VO_2_ vs. VE relations, respectively. The correlation coefficients of VO_2_ vs. log-transformed VCO_2_ and VO_2_ vs. log-transformed VCO_2_ were identical to the exponential values.

**Supplementary Figure**

Relation between (left) VO_2_peak vs. %relative vVAT and (right) VO_2_peak vs. %relative expVAT(VCO_2_).

**
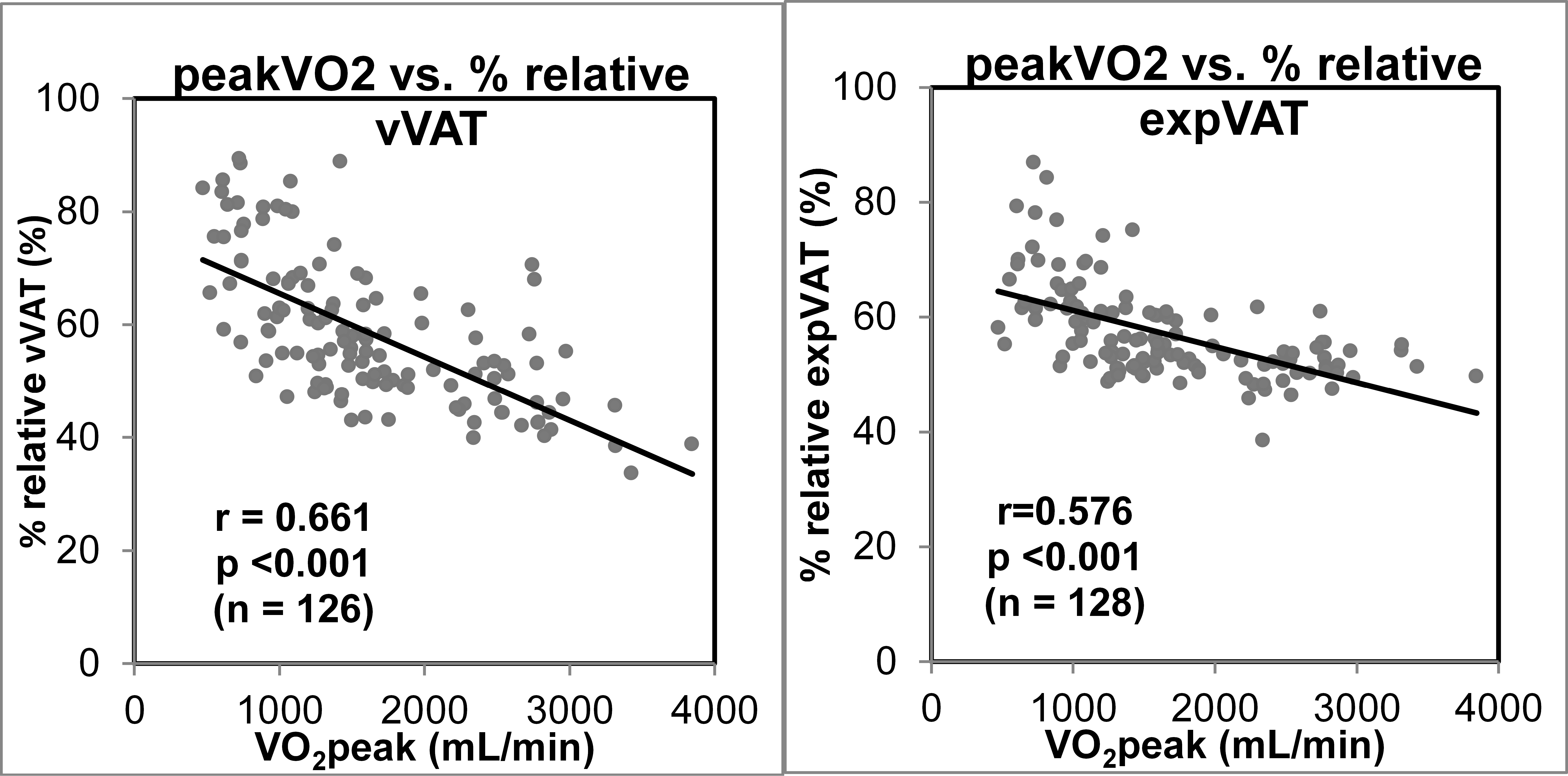
**

**Supplementary Table 1**

Age-/sex-matched comparisons of VO_2_peak, vVAT, and other derived variables of exercise tolerance between healthy and cardiac subjects (population A)

|  | Healthy (n=21) | Cardiac (n=21) | p-Value |
| --- | --- | --- | --- |
| Age, y | 65±8 | 66±8 | 0.790 |
| Male/female | 11/10 | 11/10 |  |
| VO_2_peak, mL/min | 1447±431 | 975±360 | <0.001 |
| vVAT, mL/min | 806±215 | 665±160 | 0.023 |
| expVAT(VCO_2_), mL/min | 798±187 | 633±134 | 0.002 |
| expVAT(VE), mL/min | 801±156 | 683±29 | 0.013 |
| OUES(VCO_2_), mL/min | 1579±377 | 1271±259 | 0.003 |
| OUES(VE), mL/min | 1803±357 | 1520±280 | 0.008 |

**Supplementary Table 2**

Population B/pre-rehabilitation

Correlation matrix of vVAT, expVAT(VCO_2_), VAT(VE), OUES(VCO_2_), and OUES(VE)

|  | vVAT | expVAT  (VCO_2_) | expVAT  (VE) | OUES  (VCO_2_) | OUES  (VE) |
| --- | --- | --- | --- | --- | --- |
| vVAT, mL/min |  | 0.903 | 0.804 | 0.883 | 0.799 |
| expVAT(VCO_2_), mL/min | 0.903 |  | 0.878 | 0.961 | 0.867 |
| expVAT(VE), mL/min | 0.804 | 0.878 |  | 0.906 | 0.976 |
| OUES(VCO_2_), mL/min | 0.883 | 0.961 | 0.906 |  | 0.904 |
| OUES(VE), mL/min | 0.799 | 0.867 | 0.976 | 0.904 |  |

All r values are significant at p<0.001.

Population B/post-rehabilitation

Correlation matrix of vVAT, expVAT(VCO_2_), VAT(VE), OUES(VCO_2_), and OUES(VE)

|  | vVAT | expVAT  (VCO_2_) | expVAT  (VE) | OUES  (VCO_2_) | OUES  (VE) |
| --- | --- | --- | --- | --- | --- |
| vVAT |  | 0.925 | 0.873 | 0.908 | 0.873 |
| expVAT(VCO_2_) | 0.925 |  | 0.923 | 0.973 | 0.930 |
| expVAT(VE) | 0.873 | 0.923 |  | 0.934 | 0.994 |
| OUES(VCO_2_) | 0.908 | 0.973 | 0.934 |  | 0.939 |
| OUES(VE) | 0.873 | 0.930 | 0.994 | 0.939 |  |

All r values are significant at p<0.001.

**Supplementary Table 3.**

Sensitivity analysis: derived expVAT variables before and after cardiac rehabilitation (population B)

|  | Before | After | p-value |
| --- | --- | --- | --- |
| expVAT(VCO2), up to vVAT (mL/min) | 593±146 | 611±154 | 0.124 |
| expVAT(VCO2), up to VAT+100 (mL/min) | 588±140 | 614±150 | 0.013 |
| expVAT(VE), up to vVAT (mL/min) | 718±189 | 723±185 | 0.732 |
| expVAT(VE), up to vVAT+100 (mL/min) | 679±171 | 705±174 | 0.075 |

**Supplementary material**

Comparison of three modes of VAT in each subject group (Population A)

Table 1. Mean VAT values in each subject group

| **Healthy** (n=65) | mean±SD | p: vs. vVAT | p: vs. expVAT(VCO_2_) |
| --- | --- | --- | --- |
| vVAT | 1048±308 |  |  |
| expVAT(VCO_2_) | 1095±353 | 0.038 |  |
| expVAT(VE) | 1054±297 | 1.0 | 0.087 |
| **Those with CV risks** (n=19) | mean±SD | p: vs. vVAT | p: vs. expVAT(VCO_2_) |
| vVAT | 916±237 |  |  |
| expVAT(VCO_2_) | 884±200 | 0.409 |  |
| expVAT(VE) | 884±203 | 0.400 | 1.0 |
| **Cardiac** (n=39) | mean±SD | p: vs. vVAT | p: vs. expVAT(VCO_2_) |
| vVAT | 632±172 |  |  |
| expVAT(VCO_2_) | 591±171 | 0.007 |  |
| expVAT(VE) | 634±176 | 1.0 | 0.004 |

Significant differences are observed in two comparisons:

1. between vVAT and expVAT(VCO_2_) in healthy subjects; and
2. between expVAT(VCO_2_) and expVAT(VE), and between vVAT and expVAT(VCO_2_) in cardiac patients.

**We assess the first problem**. The exponential fitting of the V-slope (VO2 vs. VCO_2_) is less satisfactory than that of the relation VO_2_ vs. VE (shortened as VE slope) in healthy subjects, although the correlation coefficient (r) is excellent in both cases. Furthermore, the degree of fitting is primarily dependent on the level of the VO_2_peak, and therefore, also on the subject group (healthy, those with CV risks, cardiac subjects). The characteristics or the configuration of the V-slope is different depending on the level of the VO_2_peak. This will be explained in the next section using actual examples.

Figure 1(a). Graphs contrasting the configuration characteristics of V-slope and VE slope. Raw data points are shown with exponential fitting superimposed

r = 0.991

r = 0.984

vVAT = 1137 mL/min, expVAT(VCO_2_) = 1343, expVAT(VE) = 1347 mL/min

Figure 1(b). V-slope (raw data points only) showing multiple linear steps after the VAT point (the same case as above).

.

Figure 1(a) shows a good exponential fit of the VE slope; whereas the V-slope manifests less satisfactory exponential curve-fitting. Discrepant parts consist of the mid-section and the top section. The top section represents the phase called metabolic hyperventilation in the presence of metabolic acidosis, in which ventilation is expected to be disproportionally in excess of actual VCO_2_.

The discrepancy in the mid-section is somewhat perplexing. To explain this, we examine Figure 1(b). This V-slope advances in multiple liner steps after the VAT point; therefore, when viewed locally, it does not manifest an exponential increase. However, the whole curve when smoothed out will probably resemble an exponential increase. Positioning a ruler on the Graph(A), the V-slope curve in parallel with the diagonal: R=1 will point to the expVAT(VCO_2_) point, which is clearly beyond vVAT.

Quickly viewing all 128 exponential curves with raw data (Additional file 2) will confirm that when VO_2_peak is relatively low (in this study), the V-slope yields itself much better to exponential fitting; at higher levels of the VO_2_peak, there are more discrepancies. On the other hand, exponential fitting of the VE does not depend on the level of VO_2_peak; it fits better.

The VO_2_peak was positively correlated with the residual VCO_2_ regarding the exponential regression (root mean square deviation (RMSD), calculated using %peak transformed values; see Statistical Analysis of the main text) (r = 0.726, p <0.01), whereas this relation was not significant with the residual of VE (r = 0.01, ns). A larger residual with increasing VO_2_peak was the result of a lesser satisfactory exponential fitting of the V-slope. The mean RMSD (root mean square deviation) in healthy subjects was significantly different between that V-slope and VE slope (4.9±1.4 vs. 3.4±1.5, p <0.001). Whereas in those with CV risks and in cardiac patients, the difference was not significant (3.6±1.7 vs. 3.2±1.4, p=0.249; 2.8±0.8 vs. 3.1±1.0, p=0.069, respectively).

**On the difference between expVAT(VCO_2_) and VAT(VE) in cardiac patients**

In cardiac patients, the mean expVAT(VCO_2_) was significantly less than the mean expVAT(VE). For this assessment, we used %peak transformed VCO_2_ and VE (see statistical methods section of the main text), because of the difference in the unit of measurement of each variable (mL/min vs. L/min).

A typical example of the V-slope and the VE slope depicted on the same graph with exponential fitting in a cardiac patient is shown in Figure 2. The VE slope is clearly less steep than the V-slope and remains above the V-slope, particularly in the early stage of exercise with lower VO_2_, indicating that there is more ventilation per oxygen uptake. A ratio of dead space/ventilation (VD/VT) has been known to be higher in cardiac patients. We speculate that a steeper V-slope tends to generate a smaller VAT value than a less steep VE slope, although the tangent is drawn differently in each case.

The exponential coefficient “*a*”, the slope of the exponential equation in cardiac patients, was significantly steeper in the V-slope than in the VE slope, although the difference was also observed in the whole group (Table 2).

Figure 2. Graph contrasting VCO_2_ (V-slope) and VE (VE slope) against VO2

vVAT = 525 mL/min, expVAT(VCO_2_) = 455 mL/min, expVAT(VE) = 542 mL/min

Table 2. Mean exponential coefficient “a” (of the equation: y = ba^x^)

|  | V-slope | VE slope | p value |
| --- | --- | --- | --- |
| All subjects | 1.0015±0.0006 | 1.0012±0.0005 | <0.001 |
| Healthy | 1.0012±0.0003 | 1.0010±0.0003 | <0.001 |
| Those with CV risks | 1.0014±0.0002 | 1.0012±0.0002 | <0.001 |
| Cardiac | 1.0020±0.0006 | 1.0017±0.0006 | <0.001 |

**On the difference between expVAT(VCO_2_) and vVAT in cardiac patients**

In cardiac patients, the V-slope often takes the shape as shown in Figure 3(a); S1 may be close to R=1 and relatively long. Therefore, the exponential fitting takes the VAT point at a mid-portion of straight S1, whereas the vVAT is at the upper end of S1 (Fig 3(b)).

Figure 3(a)


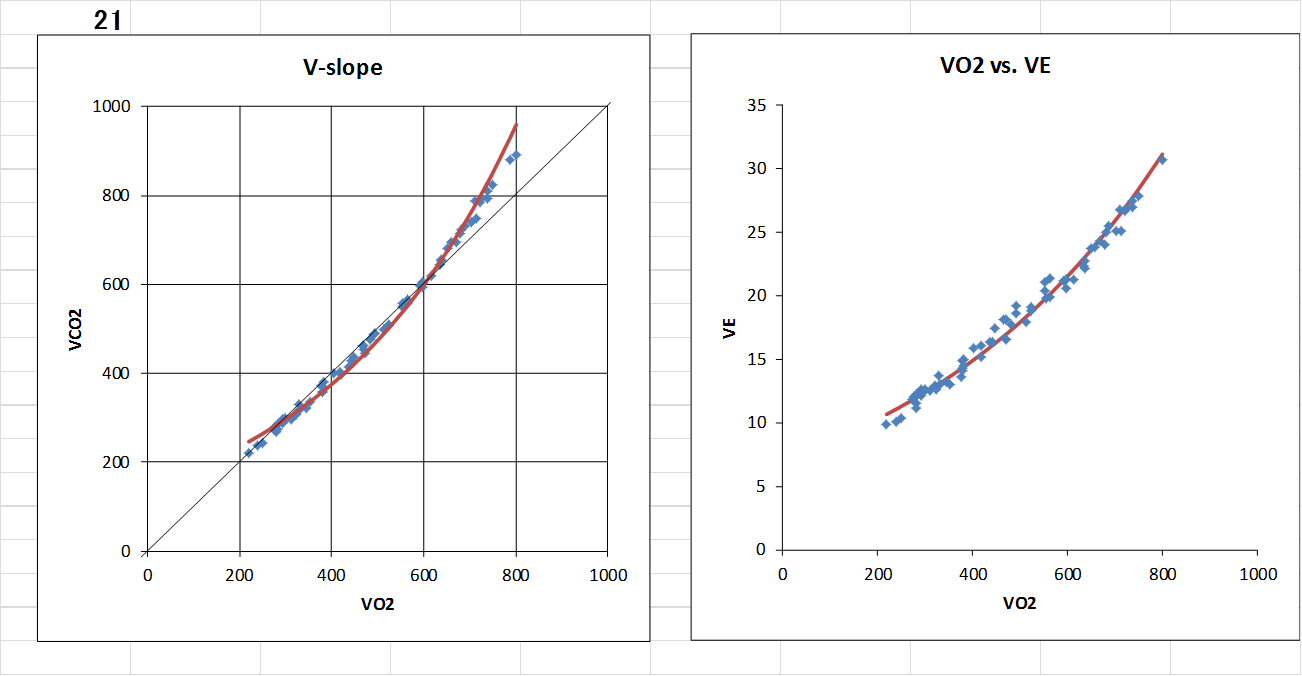


vVAT = 525 mL/min, expVAT(VCO_2_) = 455 mL/min, expVAT(VE) = 542 mL/min

Fig 3(b)


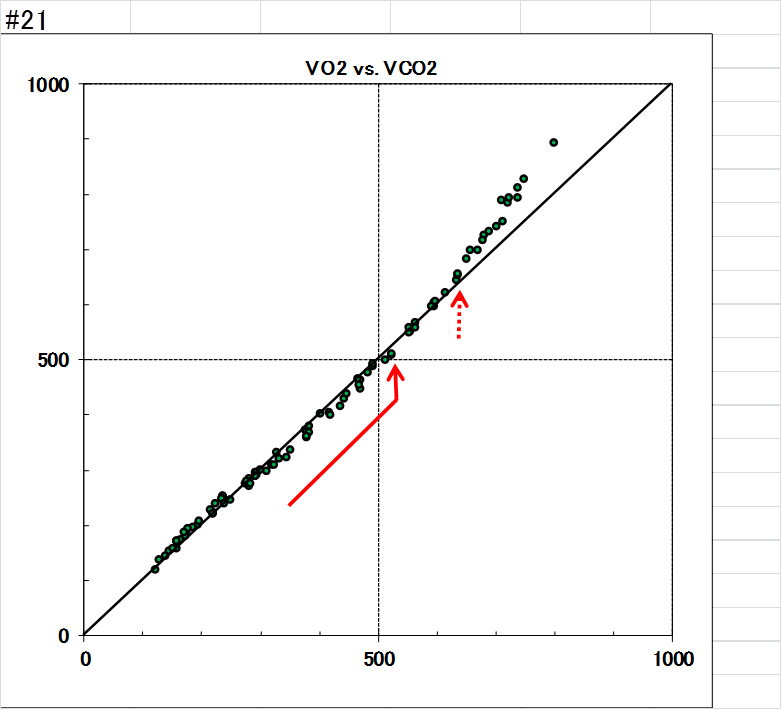

Supplement: Supplementary file 3 — Supplementary Methods-1. Derivation of computation formula of expVAT. Supplementary Methods-2. Detection of outliers on exponential fitting. Supplementary figure. Description of data: Relation of VO2peak vs. %relative expVAT. Table S1. Age−/sex-matched comparisons of CPX variables. Table S2. Correlation matrix of CPX variables in Population B. Table S3. Sensitivity analysis. Supplementary material: Comparison of three modes of VAT in each subject group (Population A). (DOCX 1058 kb) [file 13102_2019_122_MOESM3_ESM.docx]
